# The pivotal role of SFRP2 in promoting glycolysis and progression in the high-risk group based on the glycometabolism prognostic model for colorectal cancer

**DOI:** 10.1007/s00535-025-02281-5

**Published:** 2025-07-29

**Authors:** Feng Du, Xu Ji, Jiayi Su, Chuntao Liu, Junxiong Wang, Tingting Ning, Nan Zhang, Junxuan Xu, Si-an Xie, Si Liu, Li Min, Jing Wu, Shutian Zhang, Shuilong Guo, Shengtao Zhu, Peng Li

**Affiliations:** Department of Gastroenterology, Beijing Friendship Hospital, Capital Medical University, State Key Laboratory of Digestive Health, National Clinical Research Center for Digestive Disease, Beijing Key Laboratory of Early Gastrointestinal Cancer Medicine and Medical Devices, Beijing, 100050 China

**Keywords:** Glycometabolism, Colorectal cancer, Prognostic model, SFRP2, Wnt

## Abstract

**Background:**

Reprogramming glucose metabolism is a hallmark of human cancer during its occurrence and development. However, the comprehensive glycometabolism signature and underlying mechanism in CRC prognosis and immune response remind to be elucidated.

**Methods:**

A prognostic model derived from 297 glycometabolism-related genes (GRGs) was developed using LASSO-Cox and nomogram algorithms. Immune dysfunction between high-risk (Risk^H^) and low-risk (Risk^L^) groups was compared using CIBERSORT, TIMER, and TIDE analyses. The expression and function of key genes, including secreted frizzled-related protein 2 (SFRP2), were validated using PCR, western blotting, immunohistochemistry, transwell assays, and metastatic model in mice. Luciferase reporter and chromatin immunoprecipitation were used to determine the transcription regulation of ENO2 by TCF4.

**Results:**

More than half of the GRGs (152 out of 297) showed differential expression, mainly those associated with glycolysis and biosynthesis. The GRG-risk score outperformed other clinical indicators (AUC = 0.810) and served as an independent risk predictor (*P* < 0.001, HR = 3.180). The Risk^H^ group showed increased infiltration of immune cells and higher immune checkpoint expression. Mechanistically, SFRP2, a key gene in Risk^H^, promoted CRC glycolysis and metastasis via enolase 2 (ENO2) activation through the TCF4/β-catenin axis. Inhibiting ENO2 reversed SFRP2-induced metastasis. Coexpression of SFRP2 and ENO2 correlated with poorer survival and higher recurrence.

**Conclusion:**

The Risk^H^ group is characterized by glycolysis overactivation and immune exclusion. SFRP2 and ENO2 have emerged as promising treatment targets for high-risk CRC patients.

**Supplementary Information:**

The online version contains supplementary material available at 10.1007/s00535-025-02281-5.

## Introduction

Colorectal cancer (CRC) is a prevalent malignant neoplasm that ranks second in global mortality rates [1]. Current methods for treating and assessing the prognosis of CRC patients currently rely on the tumor-node-metastasis (TNM) staging system. However, approximately 15% of patients classified as low risk according to TNM staging still experience tumor recurrence and mortality due to disease progression following surgical intervention [2]. Moreover, the identification of dependable and precise biomarkers for prognostic evaluation in CRC remains a significant challenge. Genetic profiling is a method utilized in the identification of biomarkers that holds clinical significance and has been acknowledged as a crucial advancement in the establishment of efficacious therapeutic interventions through molecular stratification [3]. Given the constraints of TNM staging and the potential for discovering novel biomarkers, there is an opportunity to develop novel prognostic models that can aid clinicians in devising treatment plans in conjunction with TNM staging.

The reprogramming of energy metabolism, commonly referred to as the Warburg effect or aerobic glycolysis, is essential for the proliferation and growth of tumor cells. Changes in glycometabolism throughout the initiation, progression, and resistance to therapy of CRC are widely acknowledged as key characteristics of this disease [4]. Cancer cells exhibit increased glucose consumption and biosynthesis, resulting in an acidic extracellular environment and degradation of the extracellular matrix, which promote tumor advancement and metastasis [5]. In addition, recent research suggests that modulating glucose metabolism may enhance the efficacy of targeted therapy and immunotherapy in the treatment of CRC [6, 7]. Nevertheless, the glycometabolic gene signature of CRC remains poorly understood, and its potential prognostic and therapeutic implications have yet to be elucidated.

The Wnt signaling pathway is recognized as a significant contributor to aerobic glycolysis, serving as a principal route for glucose metabolism and playing a role in the Warburg effect observed in tumors [8]. Still yet, the precise function and mechanism by which Wnt signaling regulates glycolysis remain a topic of debate, contingent upon the specific genes it influences. The secreted frizzled-related protein (SFRP) family is known for its ability to modulate the Wnt signaling pathway through interactions with Wnt ligands or frizzled receptors, thereby impacting glucose metabolism [9]. An increasing number of studies have indicated that the overexpression of SFRP2 contributes to the development and spread of tumors and may serve as a reliable prognostic predictor of human cancers [10]. However, it is unclear whether SFPR2 affects tumor glucose metabolism to facilitate malignant tumor progression in CRC.

In this research, we identified a comprehensive signature of glycometabolic reprogramming and developed a novel prognostic risk model based on glycometabolism-related genes (GRGs) in CRC. We subsequently elucidated the specific mechanism by which SFRP2 functions as a key downstream differentially expressed gene (DEG). Through the inhibition of the Wnt pathway, SFRP2 decreases TCF4 binding to the promoter of enolase 2 (ENO2), leading to enhanced glycolysis in tumor cells, an increased Warburg effect, and the promotion of tumor cell migration in CRC. Our results offer valuable insights into glycometabolic reprogramming in CRC, suggesting that the glycometabolic reprogramming signature may serve as a potential biomarker and provide promising therapeutic targets for CRC.

## Methods and materials

### Construction and validation of a glycometabolism-related signature for CRC

The LASSO regression and glmnet packages (Cox family) were used to construct the risk model based on univariate and multivariate Cox regression analyses of OS (*P* < 0.05). The assessment equation was “regression coefficients × corresponding mRNA expression (Table S4).” The median value of the GRG risk was the cut-off for classifying the patients into the Risk^H^ and Risk^L^ categories. The survival ROC package was used to analyze survival outcomes using Kaplan–Meier (KM) and receiver operating characteristic (ROC) curves. Principal component analysis (PCA), survival curve analysis, pheatmap analysis, and receiver operating characteristic (ROC) curve analysis were conducted, and the results were visualized using R software. The corresponding nomogram calibration curves were drawn based on multivariate Cox regression analysis with the rms R package.

### In vivo metastatic model and bioluminescent imaging

All animal procedures were approved by the Experimental Animal Center, Beijing Friendship Hospital (23–2053) and carried out in accordance with the guidelines of the Beijing Friendship Hospital Animal Care Committee. BALB/C nude mice (6 weeks old) were housed under standard conditions and cared for according to the institutional guidelines for animal care. Mice were randomly assigned into experimental or control groups, blinding was not possible. For the in vivo tail vein metastasis assay, 5 × 10^6^ cells in 100 μl of PBS were injected into the tail veins of nude mice (10 per group). At weekly intervals, anesthetized mice were i.p. injected with D-luciferin (150 mg/kg) and imaged 10 min after injection using an IVIS 100 Imaging System (Xenogen, CA, USA) with an acquisition time of 2 min. The survival of the mice was recorded daily, and 8–9 weeks after tail vein injection, the mice were sacrificed and examined for metastasis through standard histologic examination.

### Serial truncation and site-directed mutagenesis of the luciferase reporter

The wild-type ENO2 promoter sequence and the corresponding mutated sequence were inserted into the pmirGLO reporter vector (KeyGEN BioTECH, Nanjing, Jiangsu, China) to obtain the ENO2-WT and ENO2-MUT luciferase reporter plasmids, respectively. SW480 cells were cotransfected with the reporter plasmids in 12-well plates for 48 h. Finally, using the Dual Luciferase Reporter Gene Assay Kit (Yeasen Biotechnology, Shanghai, China), the cell lysate was collected, and both firefly and Renilla luciferase activities were detected.

### Chromatin immunoprecipitation (ChIP) assays

ChIP assays were carried out using the SimpleChIP Enzymatic Chromatin IP kit following the established protocol. Briefly, CRC cells (1–2 million cells mL^–1^) were fixed with formaldehyde to initiate crosslinking. Subsequently, the samples were lysed, and the chromatin underwent fragmentation. Immunoprecipitation was then performed using an anti-ENO2 antibody and immunoglobulin G (IgG) as a control. Following purification using spin columns, the DNA fragments were subjected to quantitative real-time PCR analysis. The primers used are listed in Table S11. The materials and methods are available in Supplementary material.

## Results

### Identification of glycometabolism-related differentially expressed genes

**Fig. 1:**
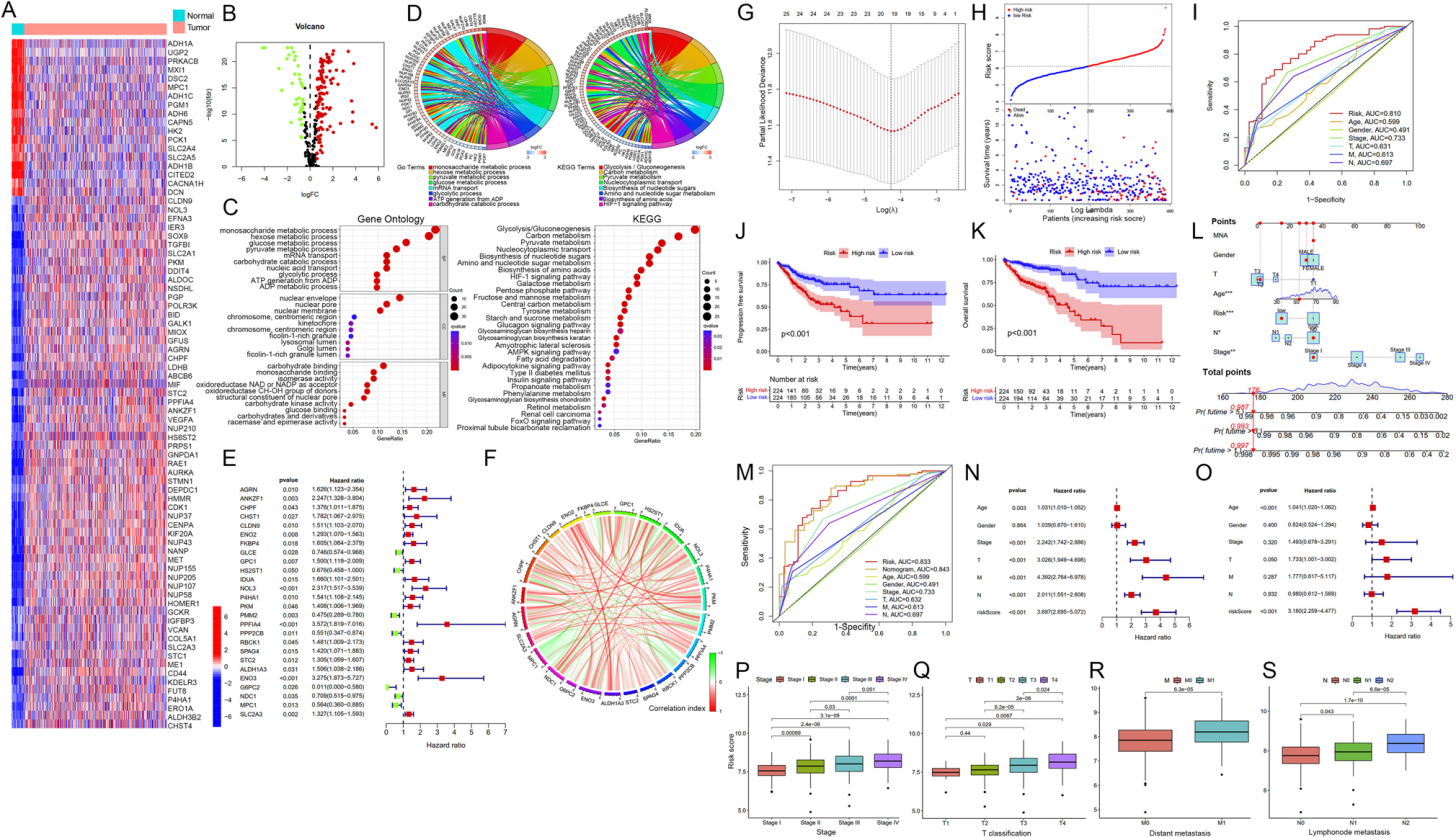
Construction and validation of a glycometabolism-related signature for CRC. **A** Heatmap and **B** volcano plot of the differentially expressed glycometabolic genes (DEGs) found in tumors and normal controls in the TCGA cohort. **C** Gene Ontology (GO, left) and KEGG (right) analyses of the glycometabolism DEGs. **D** Circos plot of GO (left) and KEGG (right) terms with related GRGs. **E** Forest plot showing the univariate Cox analysis of DEGs for overall survival in CRC patients. **F** Gene–gene correlations between the 26 prognostic GRGs. **G** A chart showing the trajectory of each independent variable over time. In this chart, lambda's log value is represented on the horizontal axis, and its coefficient is represented on the vertical axis. **H** Patient outcomes based on the risk score. The risk score for each CRC patient (top). Survival rates and survival times of patients in different groups (bottom). **I** ROC curves showing the prognostic value of the GRG signature and clinicopathologic characteristics. **J-K** K-M survival curves for the OS and PFS of CRC patients in the Risk^H^ and Risk.^L^ groups. **L** The nomogram and the calibration curves for predicting OS at 1, 3, and 5 years. **M** The predictive power of the nomogram, risk score, and clinicopathological factors determined by ROC curve analysis. **N, O** Forest plot showing the univariate and multivariate Cox analyses of the risk model for overall survival in CRC patients. **P-S** Boxplots representing the changes in risk scores with clinicopathological parameters. *P < 0.05; **P < 0.01; ***P < 0.001

We first identified 297 glycolysis-related genes (GRGs) from eight glycometabolism gene sets and analyzed data from 497 individuals (41 controls and 456 Colon Adenocarcinoma patients) from the TCGA-COAD database. Then, we found that 152 (51.2%) of GRGs were differentially expressed between CRC patients and normal controls (*P* < 0.05). Gene expression analysis revealed that glycolytic genes (ENO1, ENO2, LDHA, PGK1, PKM, GAPDH, GPI, ALDH1B1, ALDH3B2, ALDOC, ENO3, LDHB, and PFKM) were significantly upregulated in CRC samples, while ATP-generating genes (HK2, ACSS2, ADH1A, ADH1B, ADH1C, ADH6, G6PC2, GALM, GCK, PCK1, PCK2, and PGM1) were downregulated (Fig. 1A-B, Table S1). Functional enrichment analyses revealed that differentially expressed genes related to glycometabolism are mainly involved in monosaccharide, hexose, pyruvate metabolism, and glycolysis. KEGG analysis highlighted glycolysis as the most enriched pathway and also showed significant enrichment in amino acid biosynthesis, nucleotide sugars, and glycosaminoglycan (Fig. 1C). Circos plots showed a strong link between differentially expressed GRGs and KEGG and GO terms, especially in glycolysis/gluconeogenesis and glycolytic processes (Fig. 1D). This indicates metabolic changes in CRC tumors, highlighting enriched anabolic and biosynthetic pathways that aid tumor growth and metastasis.

### Construction of GRG prognostic models

To identify prognostic GRGs in CRC patients, we analyzed data from 441 patients, excluding 25 with missing outcome information. Univariate Cox regression showed 26 of 297 GRGs were significant (*P* < 0.05) (Fig. 1E). Genes such as AGRN, ANKZF1, and others were identified as risk-related based on their hazard ratio (HR) values, while GLCE, HS2ST1, and several others were deemed protective (Fig. 1E, Table S2). A network illustrating correlations among survival-associated DEGs was created (Fig. 1F). Then, we validated the GRG signature in paired CRC and normal mucosa (Fig.S1A-B) and in the TCGA cohort (Fig.S5A), and analyzed the prognostic value of 19 GRGs in CRC patients (Fig.S2).

Using differentially expressed prognostic GRGs, we developed a LASSO-Cox regression model (Fig. 1G-I). The full names, functions, and coefficients of these genes are listed in Table S3. The risk score, derived from the expression levels of nineteen signature genes and their coefficients, was used to classify CRC patients into high-risk (Risk^H^) and low-risk (Risk^L^) groups based on the median score from the training group. Patients in the GRG Risk^H^ group showed a higher mortality risk (Fig. 1J-K). Nomogram prediction models incorporating clinical parameters and the risk score were constructed to assess the predictive accuracy of the GRG (Fig. 1L). The AUC values for the nomogram and GRG signature risk score were 0.843 and 0.833, respectively, outperforming traditional clinicopathological parameters (Fig. 1M).

### Clinical correlation and model validation

We used univariate and multivariate Cox analyses to assess if the GRG signature's prognostic ability is independent of clinical features like age, sex, tumor size, lymphatic and distant metastasis, and stage. Univariate analysis showed that tumor size, lymphatic and distant metastasis, and GRG risk score (*P* < 0.001, HR = 3.697, 95% CI = 2.695–5.072) were associated with overall survival in CRC patients (Fig. 1N). Even after adjusting for other clinical parameters, the GRG risk score remained a significant independent prognostic indicator (*P* < 0.001, HR = 3.180, 95% CI = 2.259–4.477; Fig. 1O). We then grouped patients by age, sex, T stage, N stage, M stage, and other clinicopathological variables. The analysis revealed a significant increase in the GRG risk score with advancing CRC stage, larger tumor size, lymphatic metastasis, and distant metastasis (Fig. 1P-S). There was a notable correlation between the GRG risk score and the clinical TNM stage, implying that high-risk patients tend to have more advanced CRC compared to low-risk patients (Fig.S3, Table S4).

These findings were confirmed in an independent cohort (GSE39582) with molecular classification data for 566 CRC and 19 non-tumorous colorectal mucosa samples. High-risk individuals showed higher mortality and shorter OS compared to low-risk patients, consistent with the training cohort results (Fig.S4A-B). PCA divided individuals into two distinct risk groups (Fig.S4C). The AUC values of GRG risk score were reached 0.704 in the validate cohort (Fig.S4D). Univariate Cox regression showed the risk score (*P* < 0.001, HR = 3.711, 95% CI = 2.557–5.384) and clinical parameters like T stage, M stage, N stage, and tumor stage were significantly linked to OS (Fig.S4E). Multivariate Cox regression confirmed the risk score (*P* < 0.001, HR = 3.711, 95% CI = 2.557–5.384) as an independent prognostic factor for OS in the GSE39582 cohort (Fig.S4F). The GRG risk score was associated with the progression of CRC stage, larger tumor size, lymphatic metastasis, and distant metastasis (Fig.S4G), yet not with tumor location and chemotherapy type (Fig.S8C, E). A significant relationship was found between the GRG risk score and the clinical TNM stage, indicating that patients with a high risk score are prone to having more advanced CRC compared those with a low risk score (Fig.S4H, Table S5).

### DEG screening and functional enrichment analysis between the Risk^H^ and Risk^L^ groups

**Fig. 2:**
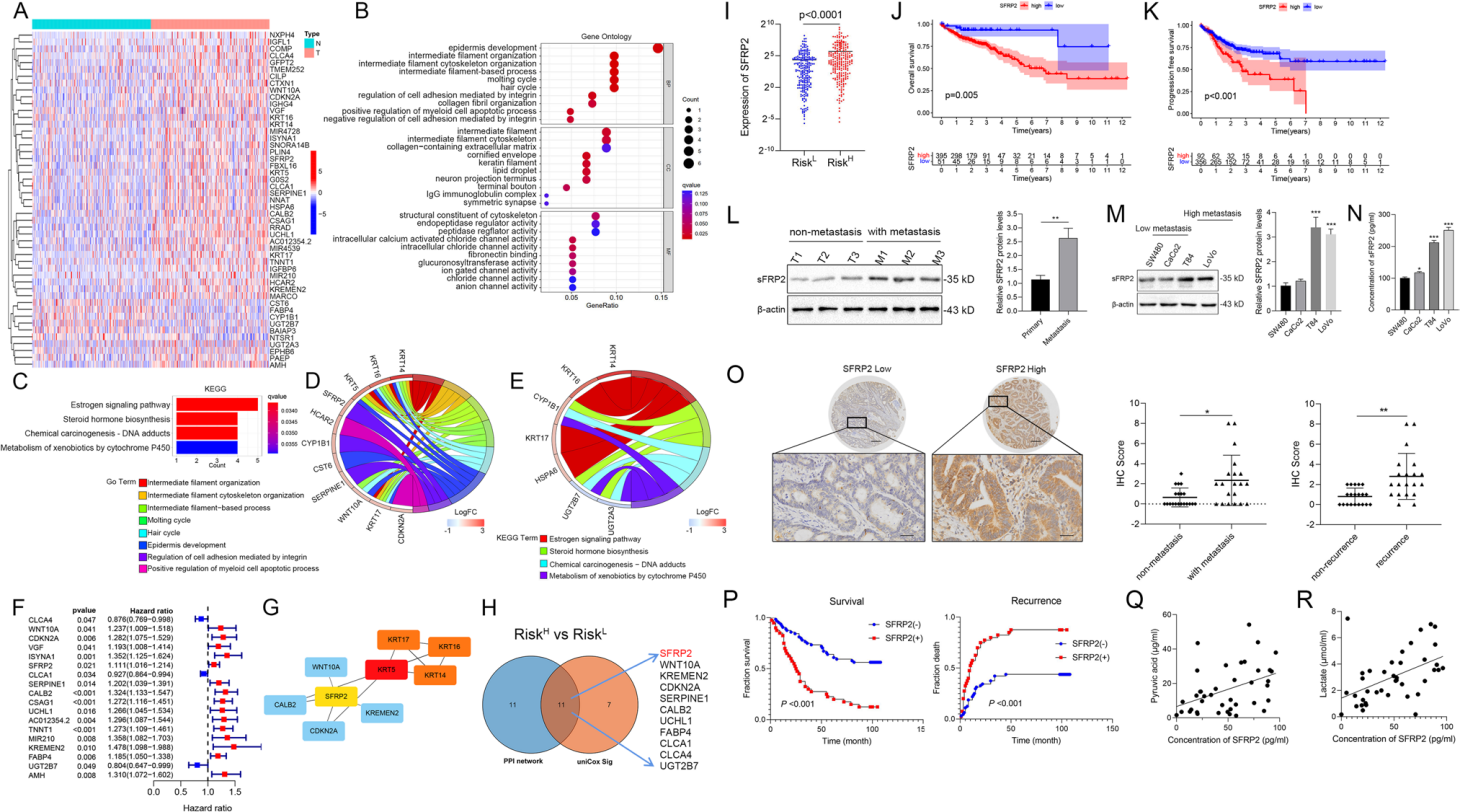
SFRP2 is a core gene between the Risk^H^ and Risk^L^ groups, and its high expression is associated with poor prognosis of CRC. **A** Heatmaps showing the 49 DEGs between Risk^H^ and Risk^L^ CRC patients. **B, C** Functional enrichment analysis of the DEGs between Risk^H^ and Risk^L^ CRC patients. **D, E** Circos plot of GO (**D**) and KEGG (**E**) terms for related genes. **F** Forest plot for the predictors of overall survival. **G** Protein‒protein interaction network analysis identified 22 core candidate genes as downstream targets of GRGs. **H** The intersection between the PPI network and prognosis-associated DEGs between the Risk^H^ and Risk^L^ groups. **I** Expression of SFRP2 in the Risk^H^ versus Risk^L^ groups based on the GRG risk model. **J-K** K-M survival curve analysis suggested that SFRP2 upregulation was associated with shorter overall survival and progression-free survival in patients with CRC. **L, M** SFRP2 was overexpressed in metastatic CRC tissues and cells. **N** ELISA test of the concentration of SFRP2 in supernatant of CRC cells. **O, P** SFRP2 overexpression indicated poor prognosis in CRC patients. **Q, R** The serum concentration of SFRP2 was positively correlated with the levels of pyruvate and lactate

To investigate the molecular basis of the GRG signature, we identified 49 DEGs between the Risk^H^ and Risk^L^ groups (Fig. 2A, Table S6) and conducted GO and KEGG pathway analyses to determine related biologic functions and pathways. GO enrichment analysis showed that DEGs were enriched in cell movement-related processes like intermediate filament organization, cell adhesion regulation, and collagen fibril organization (Fig. 2B). Related cellular components included intermediate filament cytoskeleton, collagen-containing extracellular matrix, and keratin filament, along with molecular functions involving cytoskeletal structure and endopeptidase regulator activity, and fibronectin binding, were significantly enriched in the high-risk group compared with the low-risk group (Fig. 2B). In addition, ion transport functions (such as various chloride channel activities and ion-gated channel activity) and enzyme activities (endopeptidase regulator and glucuronosyltransferase) were significantly increased in the high-risk group (Fig. 2B, P < 0.05). This indicates that highly expressed GRGs may be involved in cell migration, cell–cell communication, and cell–ECM communication. KEGG analysis revealed significant changes in the estrogen signaling, steroid hormone biosynthesis, and chemical carcinogenesis-DNA adducts pathways (Fig. 2C, P < 0.05). Key genes, including KRT14, KRT16, KRT5, KRT17, WNT10A, SFRP2, SERPINE1, and HCAR2, are highlighted in the GO Circos plot (Fig. 2D, P < 0.05, |logFC|> 1). The KEGG Circos plot (Fig. 2E) shows the relationships between these pathways and genes. Gene set variation analysis (GSVA) revealed significant differences in O_GLYCAN_BIOSYNTHESIS, GLYCOSAMINOGLYCAN_BIOSYNTHESIS_CHONDROITIN_SULFATE, and ECM_RECEPTOR_INTERACTION gene sets between the Risk^H^ and Risk^L^ groups (Fig.S5B). These findings suggest that the high risk associated with the GRG signature is linked to motility and cell–cell communication in the tumor microenvironment in CRC.

### SFRP2 is a hub DEG between the Risk^H^ and Risk^L^ groups

Univariate Cox analysis identified 15 risk-related prognostic genes and three protective genes (*P* < 0.05, Fig. 2F, Table S7). PPI analysis using Cytoscape MCODE highlighted SFRP2 as the key hub gene among the DEGs between the Risk^H^ and Risk^L^ groups (Fig. 2G-H). These findings showed that GRGs in Risk^H^ and Risk^L^ groups mainly influenced cell motility and communication. Key core genes among DEGs were identified for further validation.

Then we determined that higher SFRP2 expression in the Risk^H^ group though TCGA data analysis (Fig. 2I, Fig.S6A-C). K–M analysis indicated that higher SFRP2 levels were associated with worse outcomes, including shorter OS and PFS (Fig. 2J-K). SFRP2 protein expression was higher in metastatic CRC samples compared to nonmetastatic ones (Fig. 2L) and was also elevated in high-metastatic potential cell lines like T84 and LoVo, versus lower potential lines like SW480 and Caco-2 (Fig. 2M-N). Immunohistochemical staining further confirmed significantly increased SFRP2 levels in metastatic colorectal cancer (Fig. 2O). Subsequent investigations found that high SFRP2 expression correlates with decreased patient survival (Fig. 2P, Table S8). Univerate and multivariate analysis of CRC cohort data including 106 colon adenocarcinoma patients showed that SFRP2 could be an independent risk prognostic factor for CRC (Table 1). Moreover, serum SFRP2 levels were positively correlated with pyruvate and lactate levels, indicating a connection to glycolysis (Fig. 2Q-R). These findings highlight SFRP2's critical role in colorectal cancer metastasis and its overexpression as a prognostic marker for poor outcomes in CRC patients.

**Table 1:** Univerate and multivariate analysis of factors associated with survival in human CRC

Variables	Survival					
	Univariate analysis			multivariate analysis		
	HR	95% CI	p value	HR	95% CI	p value
Age	0.315	0.028–3.543	0.349			
Sex (female versus male)	0.884	0.532–1.469	0.635			
Tumor size (≤ 5 versus > 5 cm)	0.851	0.510–1.419	0.535			
Tumor differentiation(well/moderate versus poor)	0.551	0.336–0.905	0.019	0.661	0.389–0.121	0.124
Tumor invasion(T1-T3 versus T4)	0.544	0.289–1.023	0.059	0.767	0.392–1.502	0.439
Lymph node metastasis (absent versus present)	0.378	0.228–0.627	< 0.001	0.583	0.326–1.040	0.068
Distant metastasis (absent versus present)	0.071	0.020–0.252	< 0.001	0.153	0.041–0.570	0.005
AJCC stage(I-II versus III-IV)	0.349	0.210–0.579	< 0.001	0.535	0.295–0.972	0.040
SFRP2 expression (negative versus positive)	0.284	0.171–0.471	< 0.001	0.352	0.193–0.640	0.001

### SFRP2 promotes CRC cell migration, metastasis and glycolysis

**Fig. 3:**
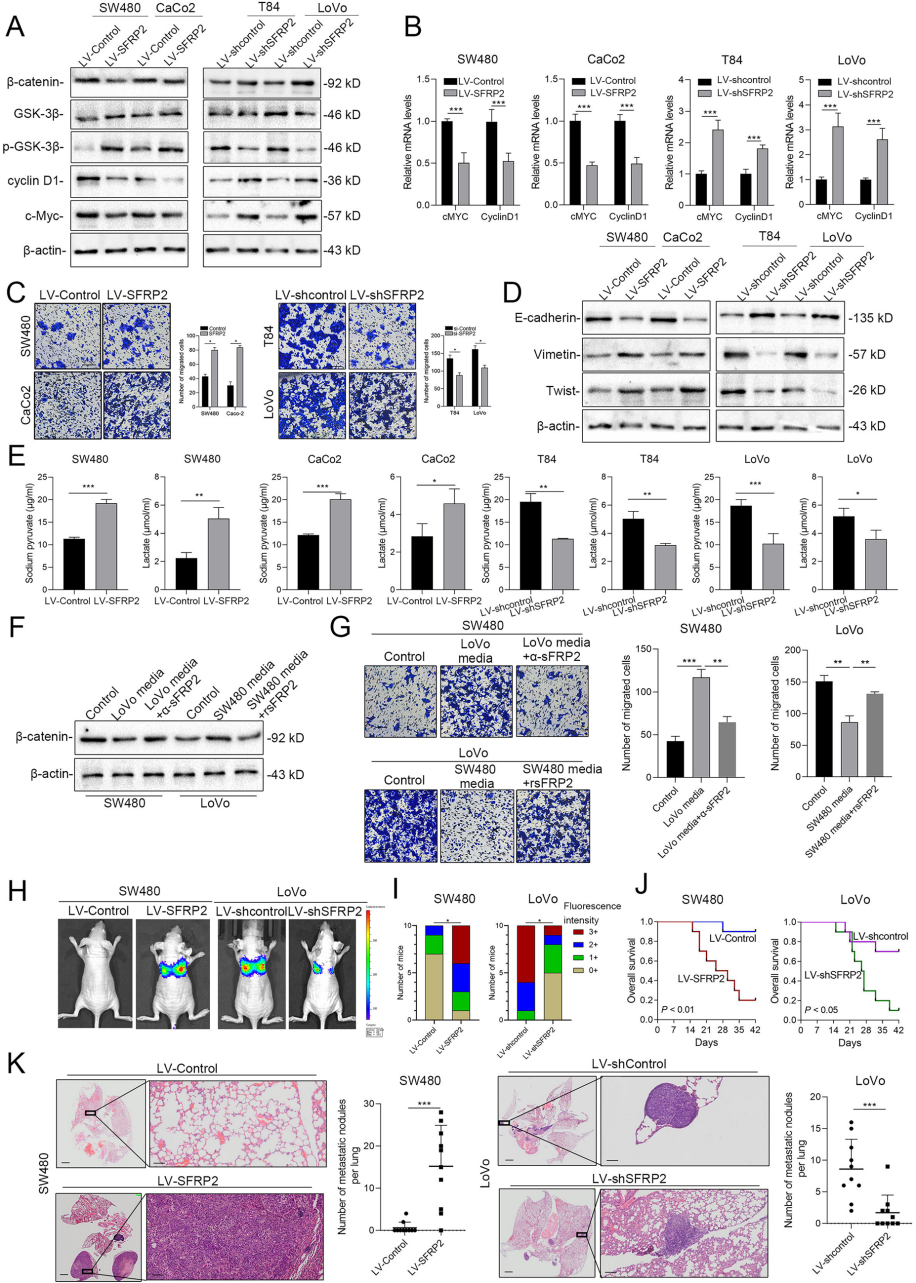
SFRP2 promotes the migration and metastasis of CRC cells in vivo*.* **A** Western blot analysis of the expression of β-catenin, Cyclin D1, C-Myc, and phosphorylated GSK-3B after the knockdown or overexpression of SFRP2 in CRC cells. **B** The mRNA levels of Cyclin D1 in CRC cells with SFRP2 knockdown or overexpression. **C** Cell migration in SFRP2-knockdown and SFRP2-overexpressing cells was determined by Transwell migration assays. **D** Western blot analysis of the expression of E-cadherin, Vimentin and Twist after the knockdown or overexpression of SFRP2 in CRC cells. **E** Measurement of pyruvate and lactate concentrations in the culture media of SFRP2-knockdown and SFRP2-overexpressing cells by ELISA. **F** Western blot analysis of the expression of β-catenin in cells cultured in media containing different concentrations of SFRP2 or blocking agents of SFRP2. **G** Cell migration in cells cultured in media supplemented with different concentrations of SFRP2 or blocking agents of SFRP2 was determined by Transwell migration assays. **H** SW480 cells expressing the scrambled vector or SFRP2 LvRNA and LoVo cells expressing the scrambled vector or SFRP2 shRNA were used to establish the lung metastasis model. Representative image of mice with lung metastasis in the two groups using in vivo bioluminescence imaging and fluorescence intensity. **J** The overall survival of the mice. **K** Representative HE images of metastatic lungs and the number of metastatic nodules. *P < 0.05; **P < 0.01; ***P < 0.001; ns. not significant; *n* = 3

To study SFRP2’s function, SW480 and CaCo2 cells were transfected with a lentivirus for SFRP2 overexpression, while T84 and LoVo cells had SFRP2 inhibited using shRNA. Protein levels in the Wnt pathway were assessed via western blotting. Results showed SFRP2 overexpression in SW480 and CaCo2 cells reduced β-catenin, Cyclin D1, and C-Myc levels, and increased phosphorylated GSK-3B, which promotes β-catenin degradation (Fig. 3A). The changes in Cyclin D1 and C-Myc mRNA levels matched the protein level changes (Fig. 3B). Transwell assays showed that SFRP2 upregulation boosted SW480 and CaCo2 cell migration, while its downregulation hindered T84 and LoVo cell migration (Fig. 3C). In addition, SFRP2 overexpression in SW480 and CaCo2 cells led to decreased E-cadherin expression, and elevated levels of vimentin and Twist (Fig. 3D). Knockdown of SFRP2 in T84 and LoVo cells reduced EMT marker expression (Fig. 3A-D). In addition, analysis of cell culture medium revealed that SW480 and CaCo2 cells overexpressing SFRP2 showed increased pyruvate and lactate production, while T84 and LoVo cells with SFRP2 knockdown exhibited decreased levels (Fig. 3E).

Further, we performed metabolism assays to verify the function of SFRP2. As shown in Fig.S7, when SFRP2 was overexpressed in SW480 and CaCo2 cells, the glycolysis rates, glycolytic capacity and glycolytic reserve were elevated (Fig.S7A, C). In addition, the basal OCR, ATP-linked OCR, maximal OCR, reserve capacity and non-mitochondrial OCR were attenuated by SFRP2 overexpression (Fig.S7B, D), indicating that SFRP2 is able to promote glycolysis and Warburg effect in SW480 and CaCo2 cells indeed. On the contrary, knockdown of SFRP2 not only dramatically depressed the glycolysis (Fig.S7E, G) but also upregulated OCR in T84 and LoVo cells (Fig.S7F, H).

We then conducted coculture experiments using medium from LoVo cells (high SFRP2) and SW480 cells (low SFRP2). Results showed that β-catenin expression in SW480 cells decreased with high SFRP2 medium, while LoVo cells showed increased β-catenin expression with low SFRP2 medium (Fig. 3F). Blocking SFRP2 restored β-catenin levels in SW480 cells, while adding SFRP2 reduced β-catenin in LoVo cells (Fig. 3F). High SFRP2 levels enhanced SW480 cell migration, whereas low SFRP2 levels inhibited LoVo cell migration (Fig. 3G). In vivo, SFRP2 overexpression increased metastatic tumor nodules and decreased mouse survival (*P* < 0.05) (Fig. 3H-J). Knocking down SFRP2 reduced metastatic tumor nodules and increased survival (Fig. 3H-J). HE staining confirmed all lung metastases (Fig. 3K). This suggests SFRP2 promotes CRC cell migration and metastasis in vivo and affects Wnt pathway and cell cycle proteins.

### SFRP2 upregulates ENO2 via Wnt antagonism to promote glycolysis in CRC

**Fig. 4:**
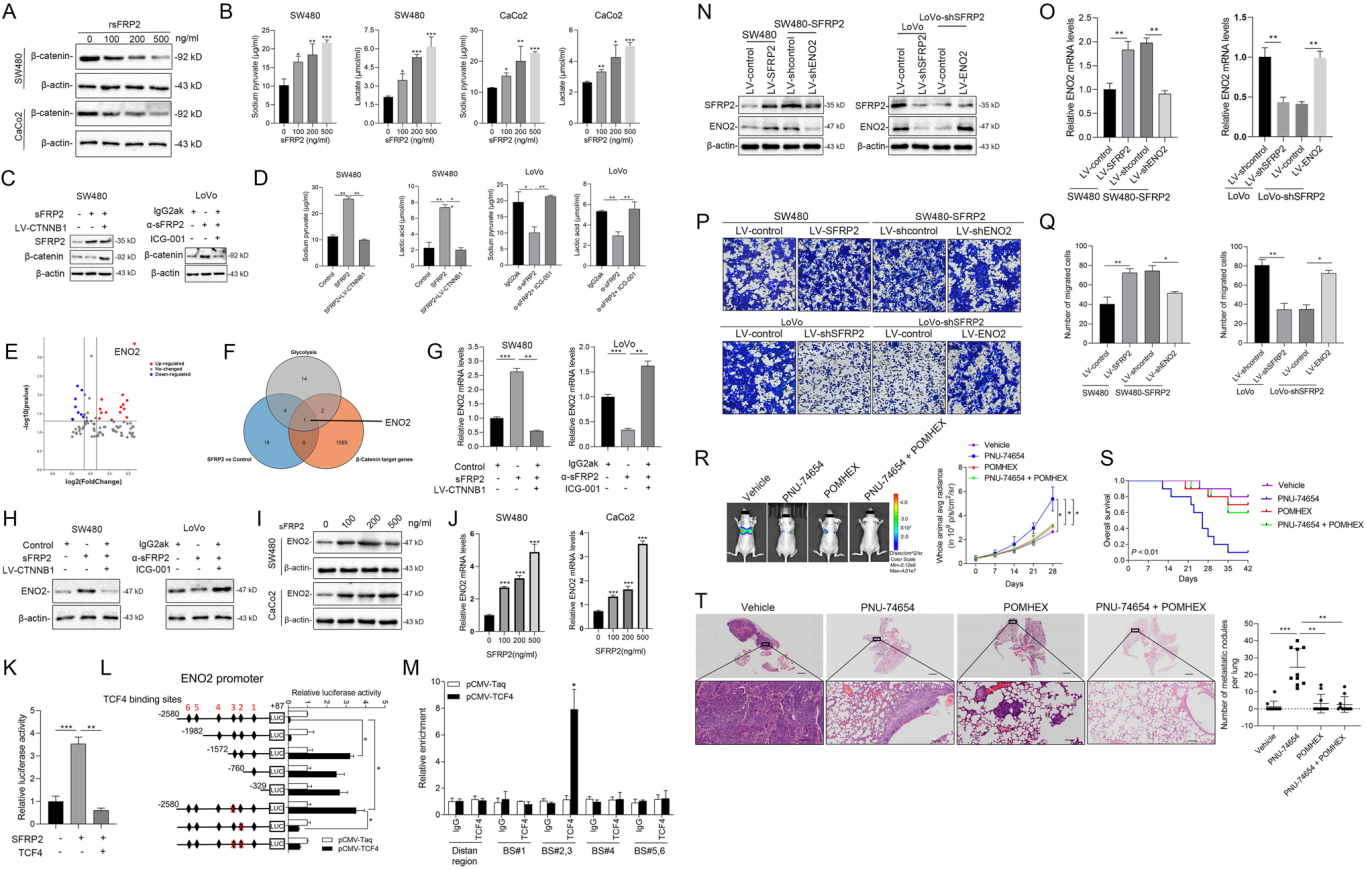
SFRP2 upregulates ENO2 via Wnt antagonism to promote glycolysis. **A** Western blot analysis of the expression of β-catenin in cells cultured in media supplemented with different concentrations of SFRP2. **B** The levels of pyruvic acid and lactate in the media of cells stimulated with different concentrations of SFRP2. **C** Protein expression levels of β-catenin and SFRP2 in cells after overexpressing SFRP2 or CTNNB1 and stimulation with antagonists of β-catenin and SFRP2. **D** The levels of pyruvic acid and lactate in the media of cells after overexpressing SFRP2 or CTNNB1 and stimulated with antagonists of β-catenin and SFRP2. **E** Volcano plots of SFPR2-interacting proteins in SW480 cells stably overexpressing SFPR2 quantified by RNA sequencing. **F** Venn diagram showing one common DEG according to the RNA sequencing results and genes related to glycolysis and β-catenin target genes in the TCGA cohort. **G-H** The mRNA and protein levels of ENO2 in cells after overexpressing SFRP2 or CTNNB1 and stimulation with antagonists of β-catenin and SFRP2. **I-J** The protein and mRNA levels of ENO2 in cells after stimulation with different concentrations of SFRP2. **K** The relative luciferase activity of ENO2 after stimulation with SFRP2 or TCF4. **L** Schematic diagram of the generated luciferase reporter plasmids and the relative luciferase activity after transfection of SW480 cells with or without TCF4 plasmids. **M** A ChIP experiment was used to predict the TCF4 binding site in the ENO2 promoter. *P < 0.05; **P < 0.01; ***P < 0.001; ns. not significant; *n* = 3. **N, O** Western blot and qPCR analysis of the correlation between SFRP2 and ENO2 in CRC cells. **P, Q** Cell migration in ENO2- or SFRP2-knockdown and -overexpressing cells was determined by Transwell migration assays; *n* = 3. **R** The effects of knocking down ENO2, a Wnt/beta-catenin antagonist (PNU-74654) and an ENO2-specific inhibitor (POMHEX), on the metastasis of LoVo cells with high SFRP2 expression in vivo. **S** The overall survival of the mice. **T** Representative HE images of metastatic lungs and the number of metastatic nodules; *n* = 10. *P < 0.05; **P < 0.01; ***P < 0.001; ns. not significant

To investigate SFRP2's effect on the Wnt pathway, SW480 and CaCo2 cells were treated with varying rsFRP2 concentrations, followed by WB and ELISA analyses. Results showed that higher rsFRP2 levels correlated with lower β-catenin expression and higher pyruvate and lactate levels (Fig. 4A-B). Overexpressing CTNNB1 or using an SFRP2 inhibitor increased β-catenin, while the Wnt/β-catenin inhibitor ICG-001 countered α-SFRP2-induced β-catenin expression (Fig. 4C). SFRP2 stimulation boosted pyruvate and lactate production, but CTNNB1 overexpression countered this effect. Inhibiting SFRP2 with α-SFRP2 reduced pyruvate and lactate levels, which was reversed by the Wnt/β-catenin inhibitor ICG-001 (Fig. 4C-D).

Given the important role of SFRP2 in CRC metastasis, a human metastasis PCR array was conducted, which showed a significant increase in ENO2 expression in SW480 cells that overexpress SFRP2 (Fig. 4E). Moreover, we used a Venn diagram to compare DEGs from the metastasis PCR array, glycolysis-related genes, and β-catenin target genes, identifying ENO2 as a common DEG (Fig. 4F). ENO2, part of the enolase family, is essential in glycolysis and gluconeogenesis for synthesizing phosphoenolpyruvate (PEP). The effect of SFRP2 on ENO2 expression was studied by treating cells with sFRP2, leading to increased ENO2 mRNA and protein levels in a dose-dependent manner. While SFRP2 activation boosted ENO2 expression, upregulation of CTNNB1 negated this effect at both mRNA and protein levels. The antagonist α-SFRP2 reduced ENO2 expression, which was reversed by the Wnt/β-catenin inhibitor ICG-001 (Fig. 4G-H). SFRP2 increased ENO2 expression in a dose-dependent manner at both mRNA and protein levels (Fig. 4I-J).

TCF4, part of the Wnt pathway, interacts with the ENO2 promoter to suppress its transcription. Using a luciferase reporter linked to the ENO2 promoter, it was found that TCF4 overexpression negates the luciferase activity boost from SFRP2 overexpression, showing ENO2 is a downstream target of the β-catenin-TCF4 complex (Fig. 4K). Experiments with the luciferase reporter and pCMV-ENO2 promoter vector showed that the binding site 2 at the C-terminal domain of the ENO2 promoter is the funcitonal TCF4 binding site (Fig. 4L-M). SFRP2 upregulates ENO2 in colorectal cancer by inhibiting Wnt pathway activation and blocking the β-catenin-TCF4 complex binding.

### Inhibition of ENO2 reduces the metastatic potential of CRC cells expressing high levels of SFRP2

To confirm SFRP2's role in boosting ENO2 expression, we created cell lines with either reduced or increased ENO2 levels. Lowering ENO2 blocked the SFRP2-induced rise in ENO2, while increasing ENO2 offset the drop caused by SFRP2 suppression (Fig. 4N-O). Reducing ENO2 also hindered SFRP2-induced cell migration, whereas increasing ENO2 restored migration after SFRP2 suppression (Fig. 4P-Q).

Subsequently, we conducted an in vivo study to investigate the impact of ENO2 knockdown, the Wnt/β-catenin antagonist (PNU-74654), and the ENO2-specific inhibitor (POMHEX) on the metastasis of CRC patients exhibiting high SFRP2 expression (Fig. 4R). Our findings revealed that inhibiting ENO2 with a Wnt/β-catenin antagonist (PNU-74654) and an ENO2-specific inhibitor (POMHEX) significantly increased the survival of mice (Fig. 4S), and the combined drugs group exhibited a significantly reduced number of metastatic tumor nodules in lung tissues (Fig. 4T).

### SFRP2 expression is positively correlated with ENO2 expression in human CRC tissues

**Fig. 5:**
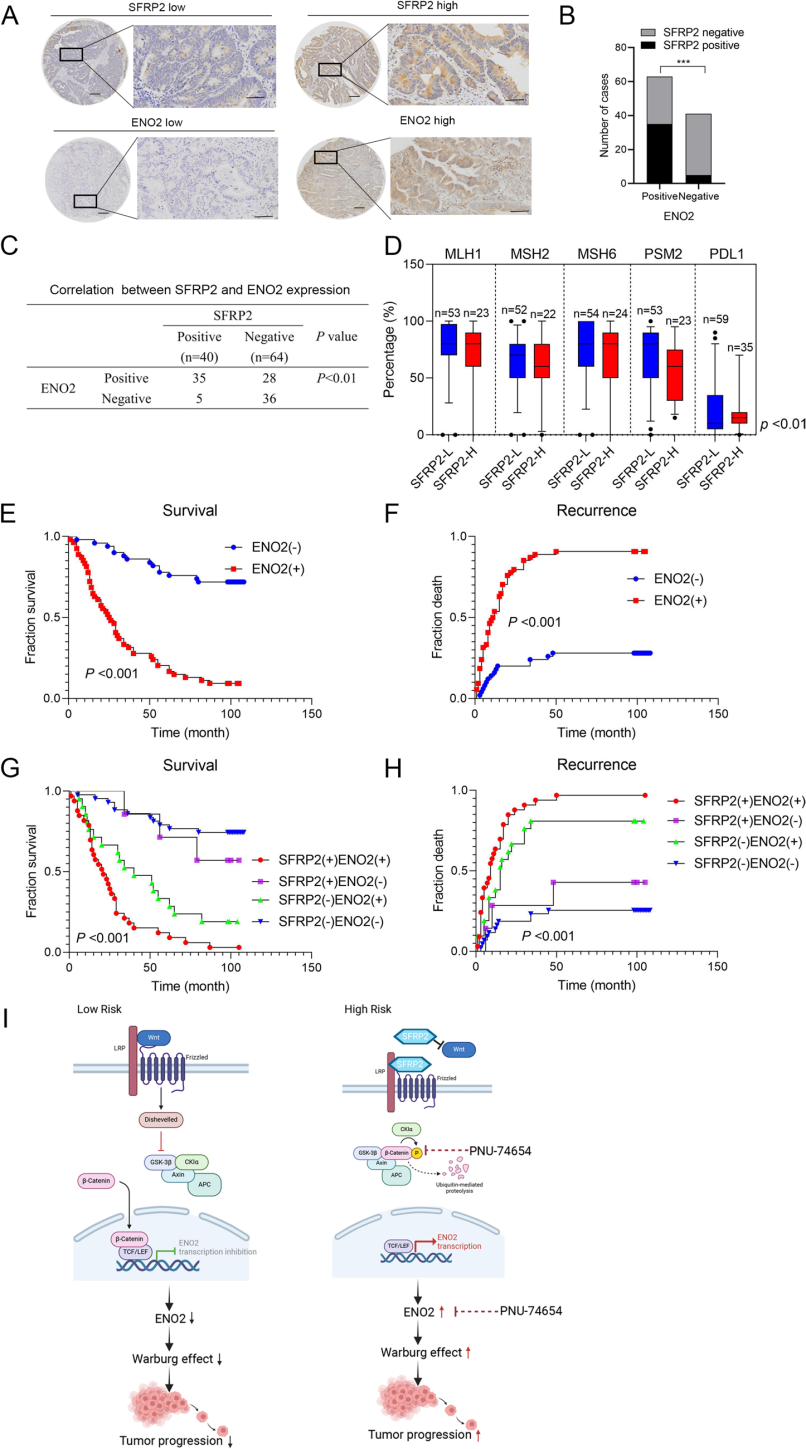
SFRP2 expression is positively correlated with ENO2 expression in human CRC tissues. (A) Representative IHC images of CRC tissues with high or low SFRP2 and ENO2 expression. Scale bars: top, 100 μm; bottom, 20 μm. **B-C** Correlation between SFRP2 and ENO2 expression in CRC patient tissues. ***P < 0.001. **D** Correlations between SFRP2 and MLH1, MSH2, MSH6, PSM2, and PDL1 expression in CRC tissues. **E, F** K–M analysis of the correlations of ENO2 expression with overall survival and recurrence. + High;—Low. **G, H** K–M analysis of the correlations of SFRP2/ENO2 coexpression with overall survival and recurrence. + High;—Low. (I) Graphic illustration of the pivotal role of SFRP2 in promoting glycolysis and progression in the high-risk group based on the glycometabolism prognostic model for CRC

We analyzed primary human CRC tissue samples with different ENO2 expression levels to study the relationship between SFRP2 and ENO2 (Fig. 5A). ENO2 expression was positively correlated with SFRP2 levels (Fig. 5B-C). Moreover, high ENO2 levels were significantly associated with advanced AJCC stage, distant metastasis, lymph node metastasis, and poor tumor differentiation (Table S9). K–M analysis showed that CRC patients with high ENO2 expression had higher recurrence rates and shorter OS times than those with low ENO2 expression (Fig. 5E-F). Furthermore, patients were categorized into four groups based on SFRP2 and ENO2 expression. Those coexpressing SFRP2 and ENO2 had the shortest OS times and highest recurrence rates (Fig. 5G-H). Taken together, these observations above demonstrate the pivotal role of SFRP2 in promoting glycolysis and progression in the high-risk group based on the GRG prognostic model for CRC(Fig. 5I).

### Differences in immune cell infiltration between the Risk^H^ and Risk^L^ groups

**Fig. 6:**
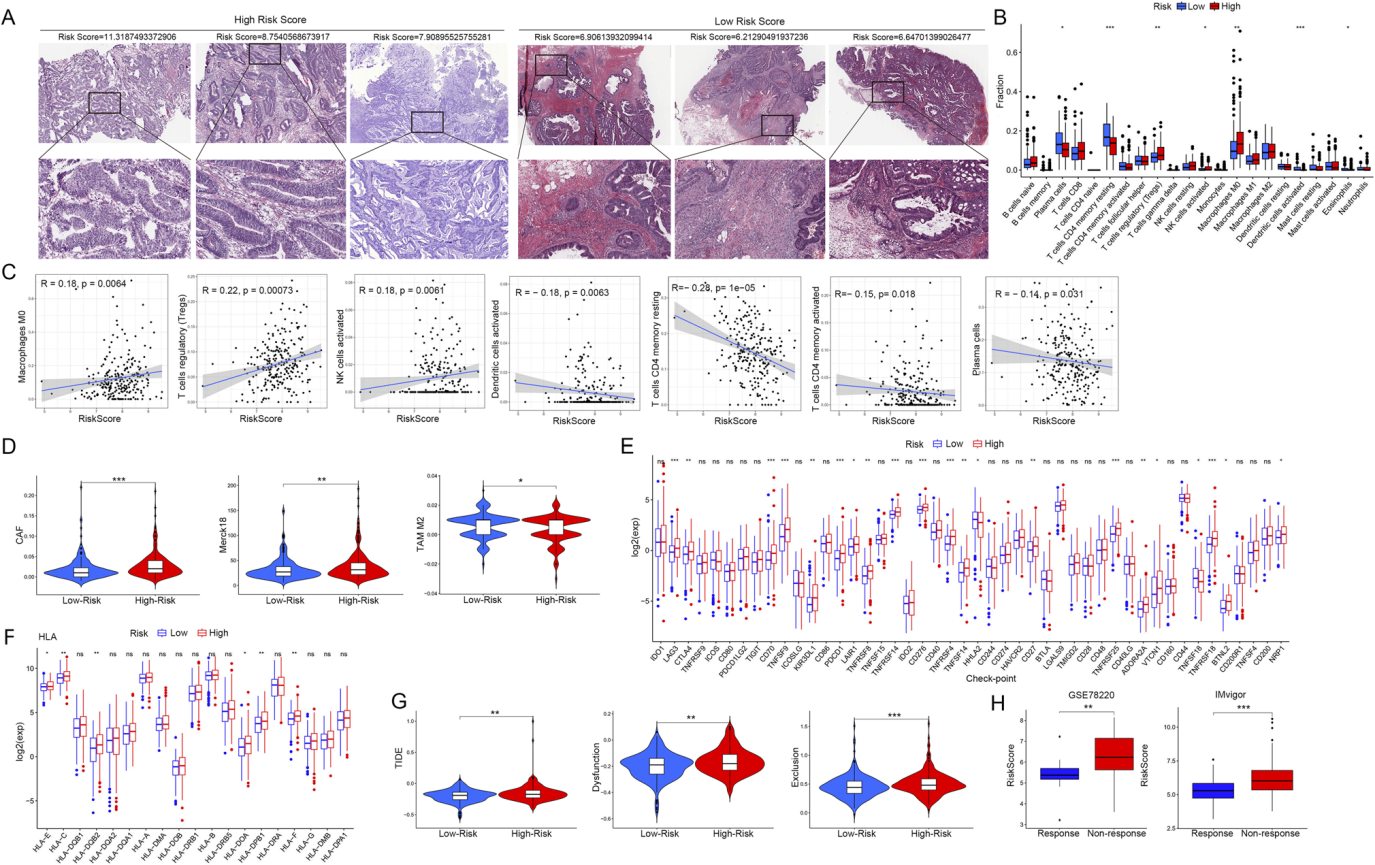
Differences in immunological characteristics between Risk^H^ and Risk^L^ CRC patients. **A** HE staining of histologic sections revealed that Risk^L^ tumors exhibited greater immune cell infiltration than did Risk^H^ tumors. **B** Relative abundance fractions of the immune cell population in the Risk^H^ and Risk^L^ groups determined using the CIBERSORT tool. **C** Correlation analysis of the risk score and immune cell infiltration. **D** Comparison of immune infiltration in the Risk^H^ and Risk^L^ groups using the TIDE score. **E** Boxplots displaying the levels of immune checkpoint regulators in Risk^H^ and Risk^L^ CRC patients (*n* = 224/224). **F** Boxplots display the levels of HLA in Risk^H^ and Risk^L^ CRC. **G** Violin plots showing the differences in TIDE score, immune escape, and dysfunction between the Risk^H^ and Risk^L^ tumors. **H** Correlation analysis of the risk score and immunotherapy response in the GSE78220 and Imvigor datasets

Immune cells infiltrating tumors can either promote or suppress tumor growth, depending on their type and interactions. Pathology slides showed greater immune cell infiltration in low-risk patients’ tumors compared to high-risk patients (Fig. 6A). The CIBERSORT method was used to estimate the scores of 22 immune cell types between high-risk and low-risk groups to understand the impact of GRG signature variation on immune cell infiltration. The results indicated that the Risk^H^ group had significantly higher infiltration of M0 macrophages, regulatory T cells (Tregs), and activated NK cells compared to the Risk^L^ group, while plasma cells, resting memory CD4 T cells, activated dendritic cells, and eosinophils showed decreased infiltration (Fig. 6B). The SFRP2^H^ group had significantly higher infiltration of M0 macrophages and less CD4^+^T memory cells compared to the SFRP2^L^ group (Fig.S6D). Meanwhile, TIMER analysis confirmed that the GRG risk score was positively correlated with M0 macrophages, Tregs, and activated NK cells infiltration, but negatively correlated with the infiltration of resting CD4^+^T memory cells, activated dendritic cells, and eosinophils (Fig. 6C). The expression of SFRP2 was positive correlated with M0 macrophages while negatively correlated with resting dendritic cells and CD4^+^T memory cells (Fig.S6E). Moreover, TIDE analysis showed more cancer-associated fibroblasts (CAFs) and Merk18 cells but fewer TAM M2 macrophages in Risk^H^ group, indicating an immunosuppressive microenvironment in GRG-Risk^H^ CRC (Fig. 6D).

Immune checkpoint blockade therapy, using antibodies like anti-PD-1 and anti-PD-L1, is a key cancer treatment with broad applicability and lasting effects when successful. However, response rates remain low, particularly in cancers with few mutations. To assess the GRG signature's predictive value for immunologic response, researchers compared the expression of several immune checkpoint genes between Risk^H^ and Risk^L^ groups. The Risk^H^ group showed increased expression of various immune checkpoint genes, including PD-1, LAG-3, CTLA-4, and others (Fig. 6E, P < 0.05), indicating potential vulnerability to immune exclusion. Since the GRG risk score was linked to activated NK and dendritic cells, we assessed the expression of human leukocyte antigen (HLA) genes. The results indicated that the high-risk group exhibited elevated expression of HLA-E, HLA-C, HLA-DQB2, HLA-DOA, HLA-DPB1, and HLA-F (Fig. 6F, P < 0.05). The expression of SFRP2 was positively correlated with the levels of TGFB3, GPR68, CD44, and SIGLEC9 (Fig.S6F-G). In addition, TIDE analysis showed higher immune exclusion and dysfunction scores in the GRG Risk^H^ group (Fig. 6G). Immunotherapy analysis showed that patients with immunotherapy resistance had higher GRG risk scores than those with immunotherapy response (Fig. 6H). In another independent cohort (GSE14333), the GRG risk score was associated with external beam radiotherapy (XRT), yet not with Cyclophosphamide (CTX) and tumor location (Fig.S8A, B, D).

### Gene mutations in the glycometabolism-related gene signature

To determine differences in gene mutations between high-risk and low-risk groups, we analyzed TCGA nucleotide variation data. Tumor mutation burden analysis identified APC (71.02%), TP53 (52.26%), TTN (47.98%), KRAS (43.23%), and PIK3CA (29.93%) as the most frequently mutated genes (Table S10). Mutation profiles for 26 prognostic glycometabolism-related genes were also constructed using the maftools algorithm (Fig.S9A). Out of 448 individuals, 102 (22.77%) had altered mutations, with the highest rates in AGRN (6%), PPFIA4 (4%), ALDH1A3 (4%), CHST1 (3%), and NDC1 (2%). Notably, ALDH1A3 mutations, which affect an enzyme using retinal as a substrate, significantly co-occurred with mutations in SPAG4, HS2ST1, ANKZF1, GPC1, GLCE, and NDC1 (Fig.S9B). CHST1 encodes an enzyme that sulfates keratin proteoglycan, regulating glycosaminoglycan metabolism and keratan sulfate biosynthesis, and often coexists with mutations in *PMM2*, *ENO3*, *GPC1*, *STC2*, and *SLC2A3*. *TP53* mutation is crucial for the progression from adenoma to adenocarcinoma in CRC, linked to poor prognosis and chemoresistance. GOF *p53* mutants enhance cell proliferation, migration, and invasion through various mechanisms. The GRG signature risk score was significantly higher in the *TP53* mutation group compared to the wild-type group, suggesting that *TP53* mutations may enhance CRC malignancy via glycometabolism. Similar findings were noted for mutations in *MUC16*, *KMT2D*, *FBXW7*, *USH2A*, *RYR1*, and *RYR3* (Fig.S9C).

## Discussion

Alterations in glycometabolism have been acknowledged as a hallmark of cancer development and progression in recent decades [11]. Warburg effect revealed a distinctive metabolic profile exhibited by cancer cells, whereby glucose was preferentially fermented into lactate, even in the presence of ample oxygen for mitochondrial oxidative phosphorylation [12]. It is evident that glucose and glutamine are the primary sources of carbon, nitrogen, free energy, and reducing equivalents, which are indispensable for producing two viable daughter cells at mitosis [13]. The Warburg effect is an important feature of cancer cells and plays an important role in promoting carcinogenesis, but it cannot be simply regarded as the direct and sole driver of carcinogenesis.

Our investigation demonstrated the clinical significance of the glycometabolic gene profile in colorectal cancer, which aligns with the Warburg effect. A comprehensive analysis of 297 glycolytic regulatory genes (GRGs) in colorectal cancer revealed that a substantial proportion (51.2%) exhibited differential expression, underscoring the probable significance of glycometabolism in the pathogenesis of colorectal cancer. Specifically, the levels of important enzymes in the tricarboxylic acid cycle are decreased in cancer cells. Conversely, the levels of critical regulators of glycolysis and the pentose phosphate pathway are significantly greater in colorectal cancer tissues than in normal tissues, suggesting increased glycolysis and gluconeogenesis. Our identification of a comprehensive glycometabolism reprogramming signature provides a basis for further investigation into potential biomarkers and therapeutic targets.

Enhanced comprehension of gene regulatory networks may facilitate the creation of novel prognostic models for colorectal cancer [14]. This study successfully identified 26 prognosis-associated GRGs and established a 19-gene GRG signature risk model for predicting CRC prognosis. The GRG signature risk score developed in this research serves as a valuable independent prognostic indicator that is associated with tumor size, lymphatic metastasis, distant metastasis, and tumor stage. Li et al. developed a predictive model incorporating preoperative levels of CEA, CA19-9, and CA125 with an area under the receiver operating characteristic curve (AUC) of 0.774. They also found that longitudinal measurements could enhance the accuracy of predicting the prognosis of patients with colorectal cancer, necessitating a minimum of one year of postoperative follow-up [15]. In our study, the AUCs of the GRG prognostic model and the GRG signature risk score achieved values of 0.843 and 0.833, respectively, surpassing those of models based on clinicopathological parameters. Despite the fact that the AUC was much lower in Fig.S4D, it is nonetheless incumbent upon us to re-confirm these findings with a cohort of greater sample size in future. These findings suggests that the GRG prognostic model may offer superior predictive performance.

Moreover, our study sought to elucidate the mechanisms underlying the differential expression of gene regulatory genes within the identified signature. Functional analyses revealed an increase in genes related to cell movement, ion transport functions, and enzyme activity in individuals classified as at high risk (Risk^H^), all of which are closely associated with tumor metastasis. In addition, Sonveaux's research provided compelling evidence supporting the notion that various metabolic alterations, such as shifts in tumor pH, glycolysis, and pentose phosphate synthesis, promote the progression of metastasis [4]. In this study, SFRP2, a secreted glycoprotein known to reduce Wnt activity by diminishing β-catenin levels, was identified as a central gene that serves as a crucial downstream gene in glycometabolism in metastatic colorectal cancer. Furthermore, the function of SFRP2 in cancer is complex, with research suggesting a decrease in its expression at the initiation of gastric cancer, suggesting a potential tumor-suppressive role [16]. Conversely, in breast cancer and hepatocellular cancer, it has been shown to enhance migration and resistance to apoptosis [17]. Our study demonstrated that the overexpression of SFRP2 inhibits the Wnt-induced increase in free β-catenin levels and reduces the expression of cell cycle-related proteins, such as Cyclin D1 and C-Myc. Interestingly, the levels of pyruvate and lactate in the cell culture medium increased under the aforementioned conditions. In addition, our results indicate that cancer cells with heightened malignant potential exhibit increased secretion of SFRP2. Treatment of cancer cells with low malignant potential with high-SFRP2 supernatant enhances cell migration and decreases intracellular β-catenin levels, suggesting a potential reversible mechanism. From these findings, it is clear that Wnt/β-catenin signaling regulates SFRP2 to ensure that CRC cells migrate through glucose metabolism.

Aberrant Wnt signaling is considered to be a driver of metabolic alterations in glycolysis and indirectly affects the immune microenvironment of tumors [18, 19]. Our research showed that an immunosuppressive microenvironment exists for tumor development in GRG-Risk^H^ CRC. The GRG risk score was associated with immune exclusion and dysfunction scores, external beam radiotherapy (XRT), yet not with Cyclophosphamide (CTX). Meanwhile, the expression of enolase 2 (ENO2), a downstream gene of SFRP2 in CRC, was further examined to elucidate the association between SFRP2 and Wnt signaling. Our research revealed that ENO2 expression in tumor tissues is correlated with poor prognosis in CRC patients and promotes the migration, invasion, and metastasis of CRC cells. The upregulation of ENO2 expression following the stimulation of cells with SFRP2 was achieved by inhibiting TCF4 binding to the ENO2 promoter sequence, and inhibition of ENO2 reduced the metastatic potential of CRC cells expressing high levels of SFRP2. More importantly, inhibiting ENO2 with a Wnt/β-catenin antagonist (PNU-74654) and an ENO2-specific inhibitor (POMHEX) significantly increased the survival of mice and lung metastasis. Consequently, targeting the Wnt pathway or the key glycolytic enzyme ENO2 may be a potential therapeutic approach for CRC progression and drug resistance.

In conclusion, we characterized a detailed GRG signature and developed a novel prognostic risk model for patients with CRC. The glycometabolism Risk^H^ group demonstrated a greater likelihood of glycolysis activation and immune exclusion. Specifically, the downstream differential gene SFRP2 was identified as a key player in promoting the Warburg effect and metastasis in high-risk CRC patients. These findings offer valuable insights into the potential of utilizing the GRG signature of glycometabolism as a prognostic tumor marker in cancer and exploring therapeutic approaches to address immune environment alterations induced by abnormal glycometabolism. Like many studies, the current research is limited by factors such as the small size of the human samples. Future research should focus on validating the model established in this study within a larger population.

## Supplementary Information

Below is the link to the electronic supplementary material.

Supplementary file1 (TIF 25539 KB)

Supplementary file2 (TIF 25521 KB)

Supplementary file3 (TIF 24684 KB)

Supplementary file4 (TIF 25522 KB)

Supplementary file5 (TIF 25519 KB)

Supplementary file6 (TIF 25516 KB)

Supplementary file7 (TIF 25517 KB)

Supplementary file8 (TIF 25513 KB)

Supplementary file9 (TIF 25522 KB)

Supplementary file10 (PDF 57 KB)

Supplementary file11 (PDF 44 KB)

Supplementary file12 (PDF 59 KB)

Supplementary file13 (PDF 42 KB)

Supplementary file14 (PDF 43 KB)

Supplementary file15 (PDF 50 KB)

Supplementary file16 (PDF 45 KB)

Supplementary file17 (PDF 67 KB)

Supplementary file18 (PDF 68 KB)

Supplementary file19 (DOCX 1870 KB)

Supplementary file20 (PDF 49 KB)

Supplementary file21 (DOCX 29 KB)
