# Supplementary figures and images for: The pivotal role of SFRP2 in promoting glycolysis and progression in the high-risk group based on the glycometabolism prognostic model for colorectal cancer
Source: J Gastroenterol. 2025 Jul 29;60(11):1400–13. doi: 10.1007/s00535-025-02281-5 (PMC12549743; doi:10.1007/s00535-025-02281-5)

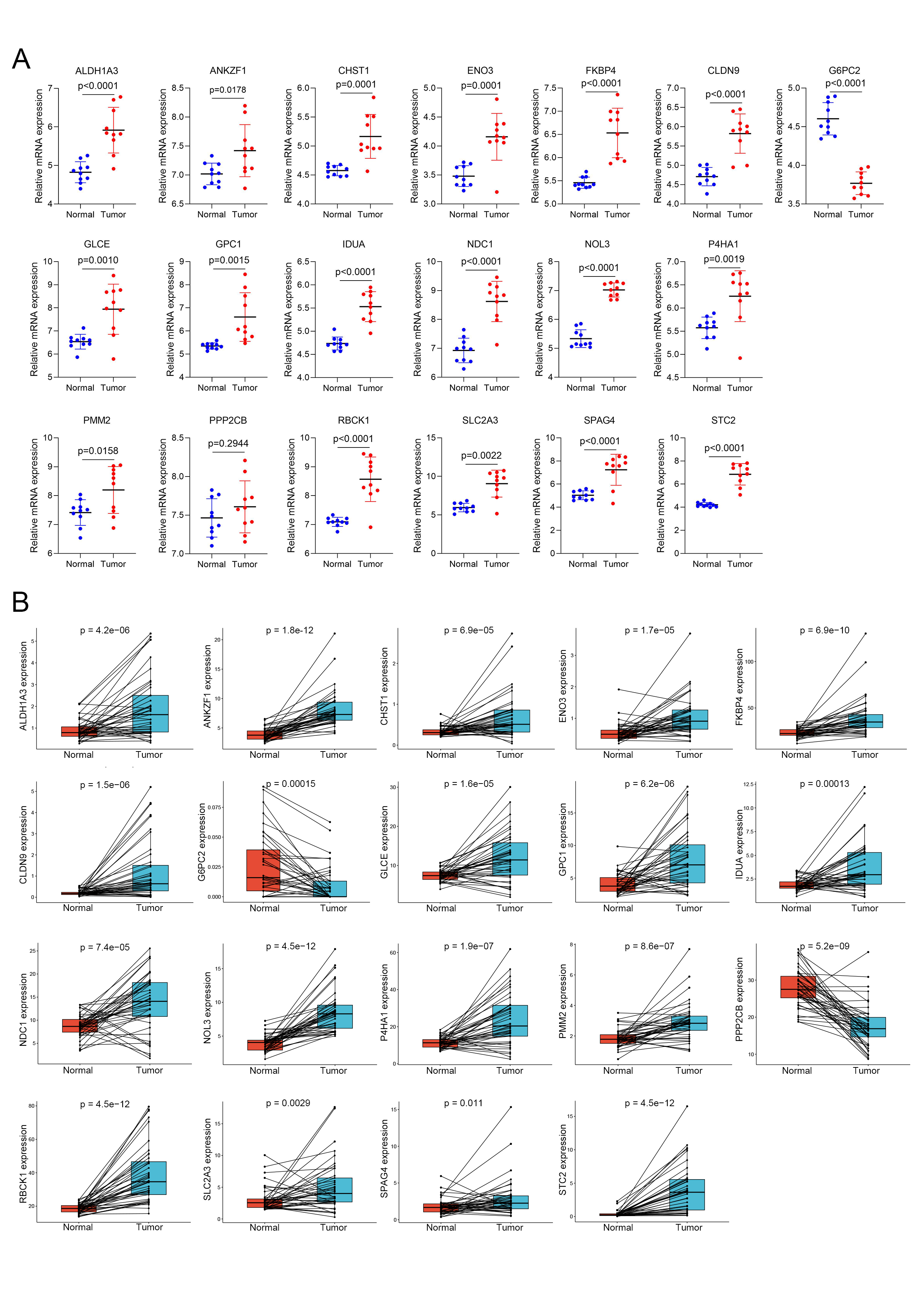

Supplement: Supplementary file 1 — Supplementary file1 (TIF 25539 KB) [file 535_2025_2281_MOESM1_ESM.tif]

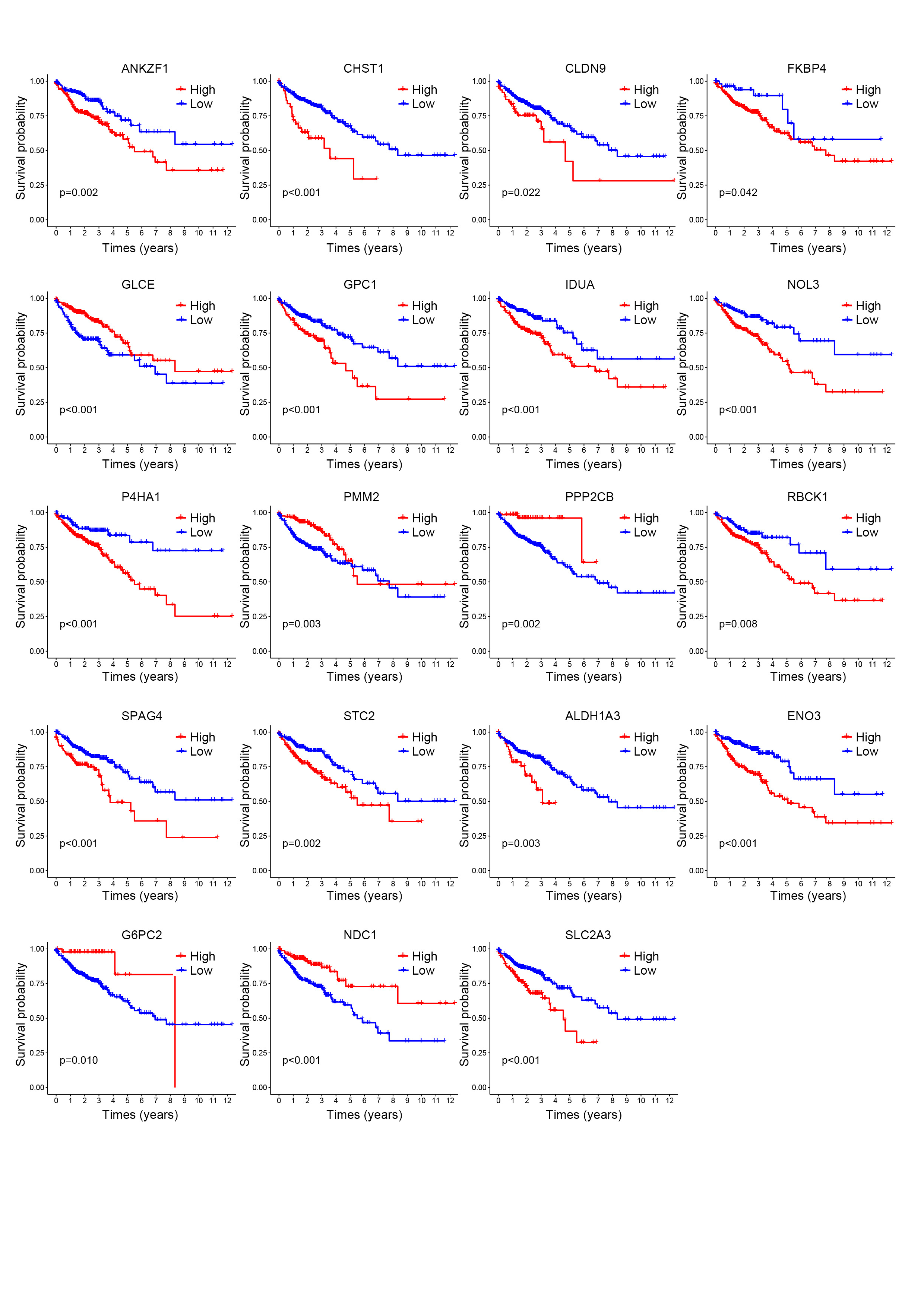

Supplement: Supplementary file 2 — Supplementary file2 (TIF 25521 KB) [file 535_2025_2281_MOESM2_ESM.tif]

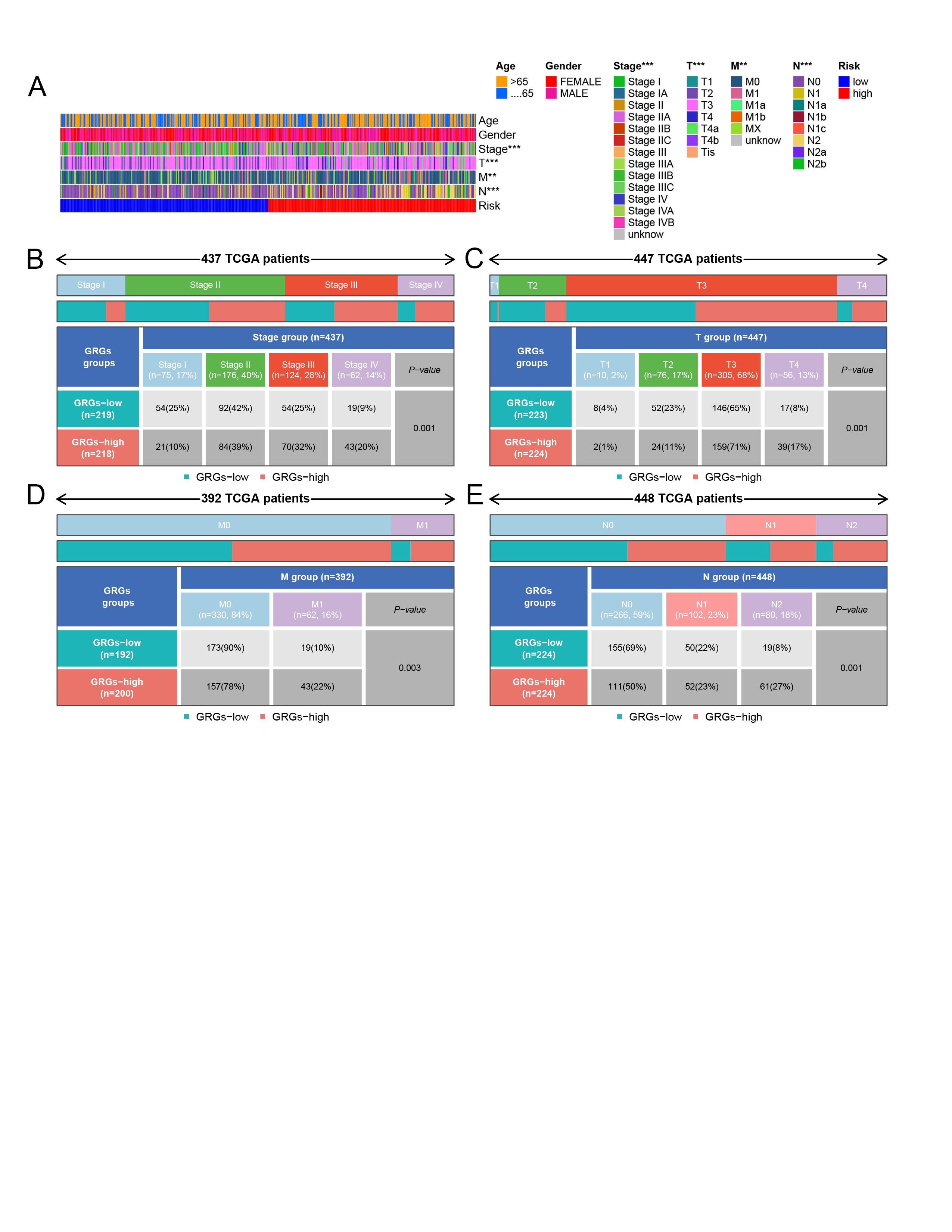

Supplement: Supplementary file 3 — Supplementary file3 (TIF 24684 KB) [file 535_2025_2281_MOESM3_ESM.tif]

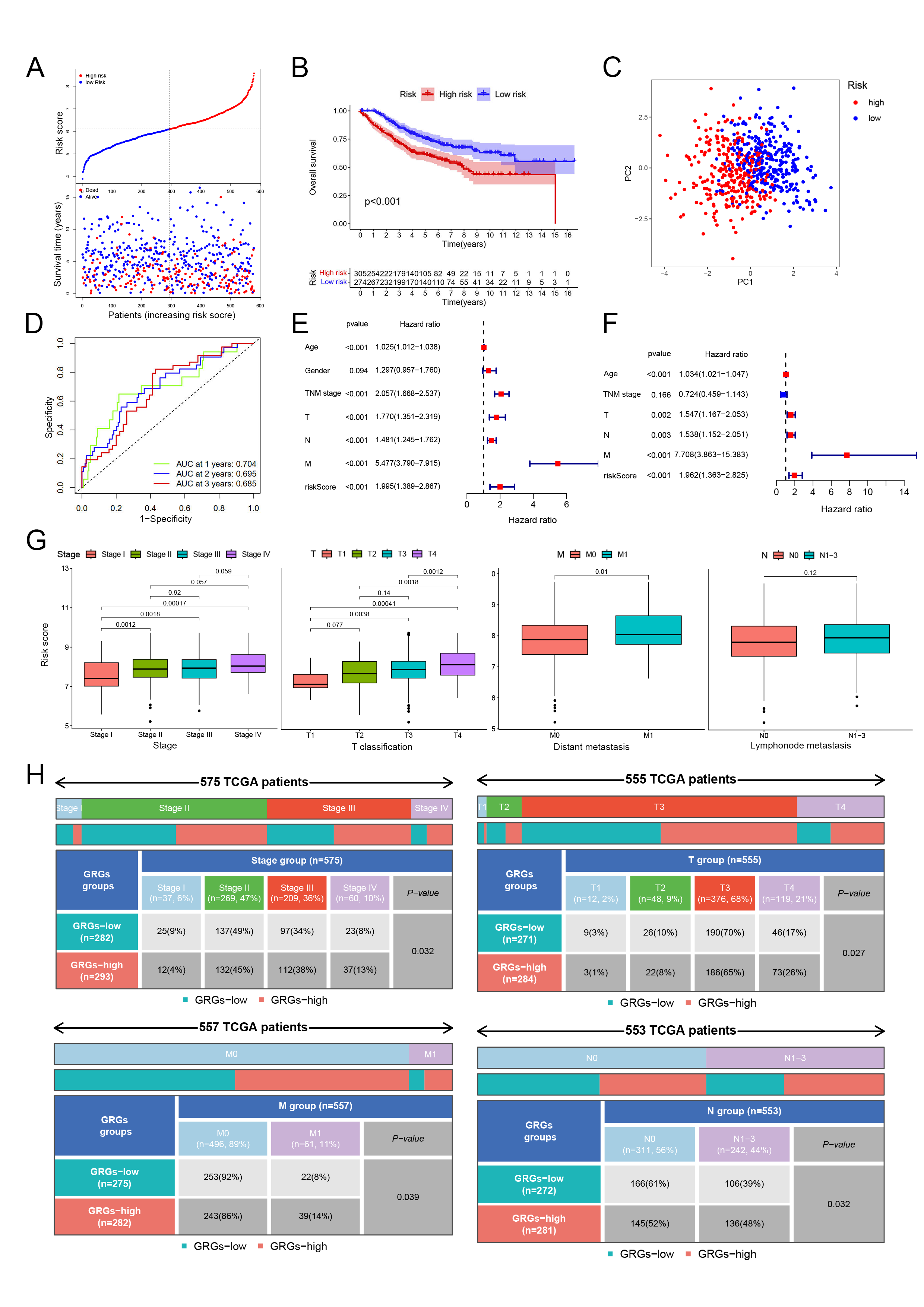

Supplement: Supplementary file 4 — Supplementary file4 (TIF 25522 KB) [file 535_2025_2281_MOESM4_ESM.tif]

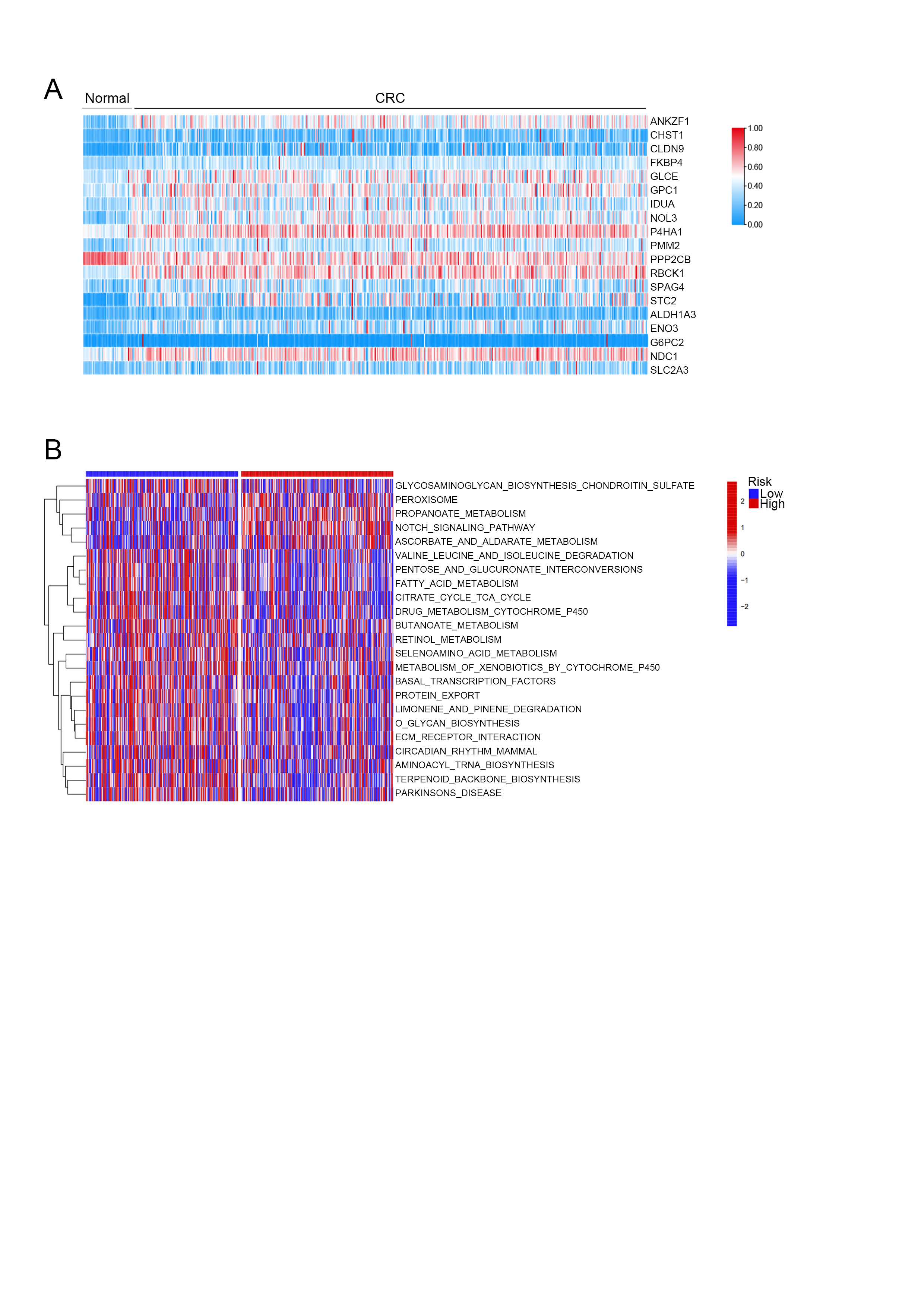

Supplement: Supplementary file 5 — Supplementary file5 (TIF 25519 KB) [file 535_2025_2281_MOESM5_ESM.tif]

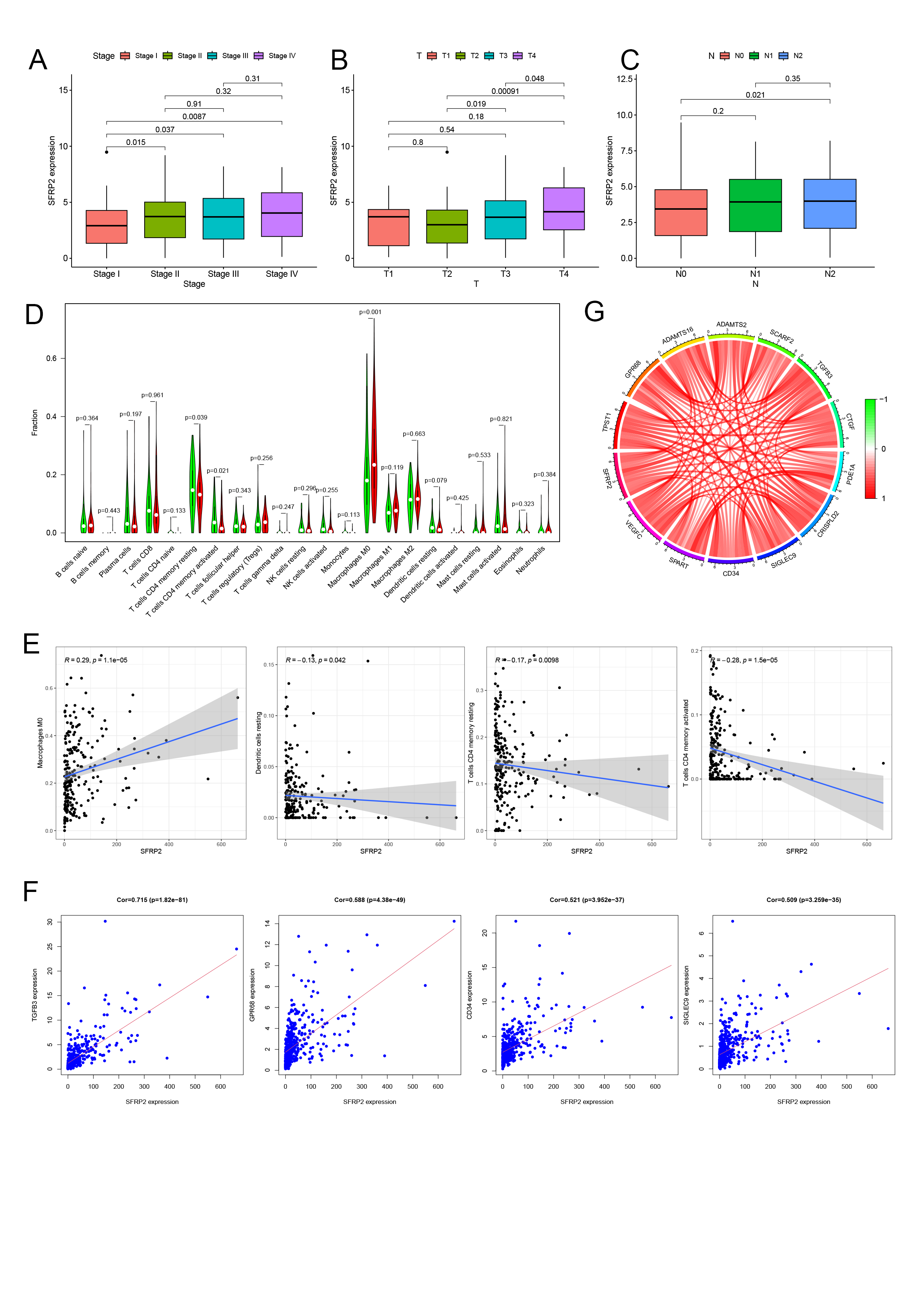

Supplement: Supplementary file 6 — Supplementary file6 (TIF 25516 KB) [file 535_2025_2281_MOESM6_ESM.tif]

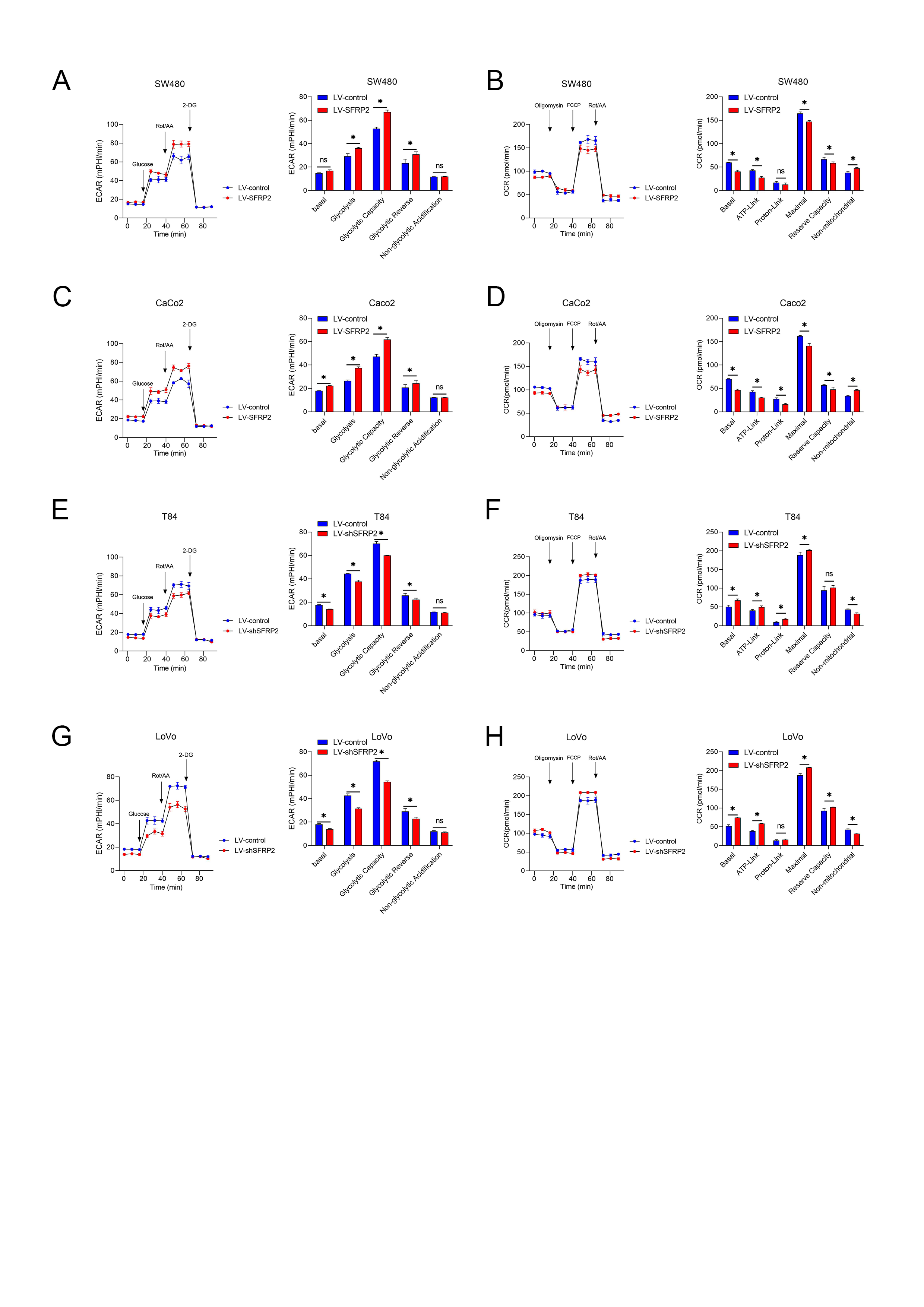

Supplement: Supplementary file 7 — Supplementary file7 (TIF 25517 KB) [file 535_2025_2281_MOESM7_ESM.tif]

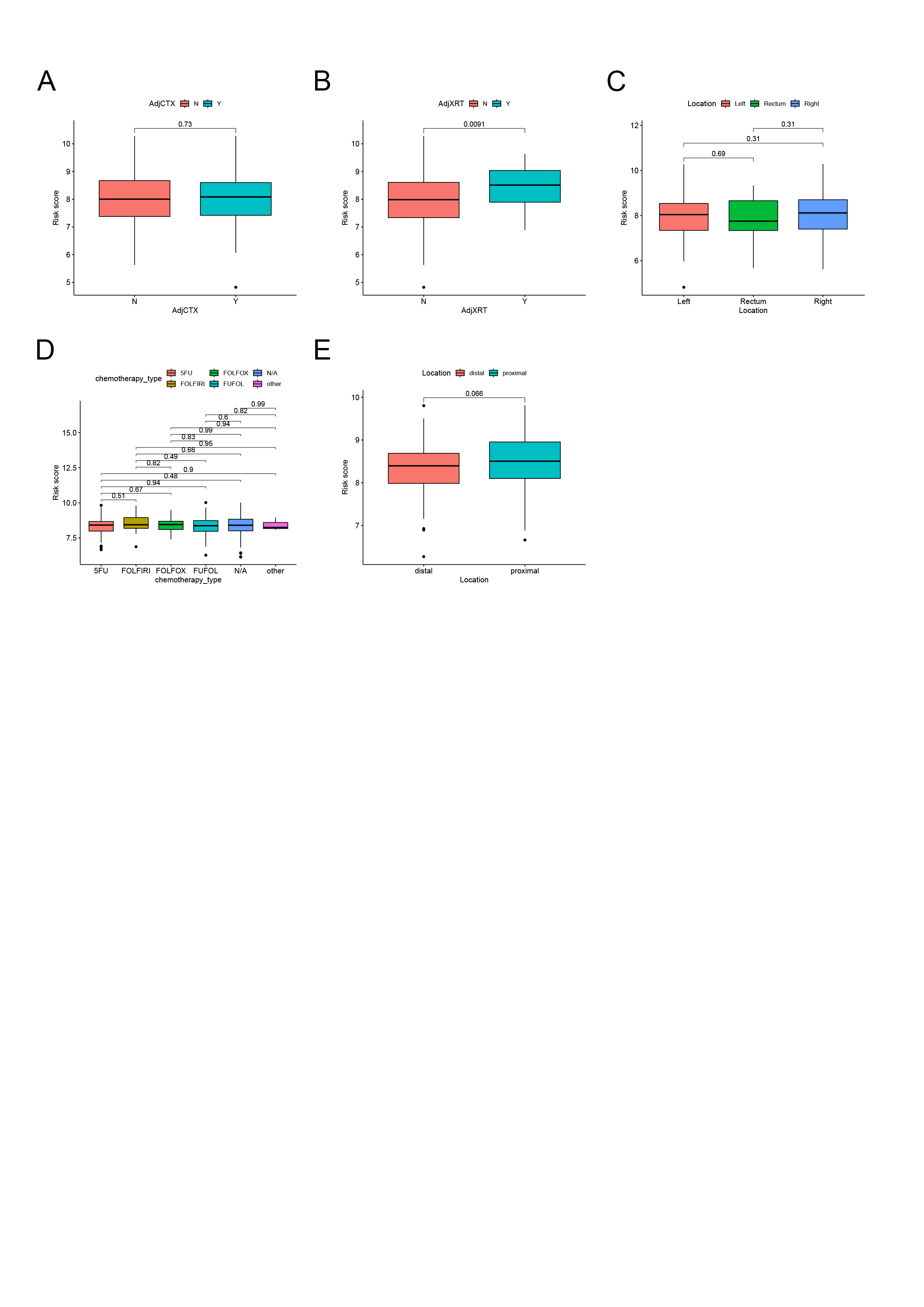

Supplement: Supplementary file 8 — Supplementary file8 (TIF 25513 KB) [file 535_2025_2281_MOESM8_ESM.tif]

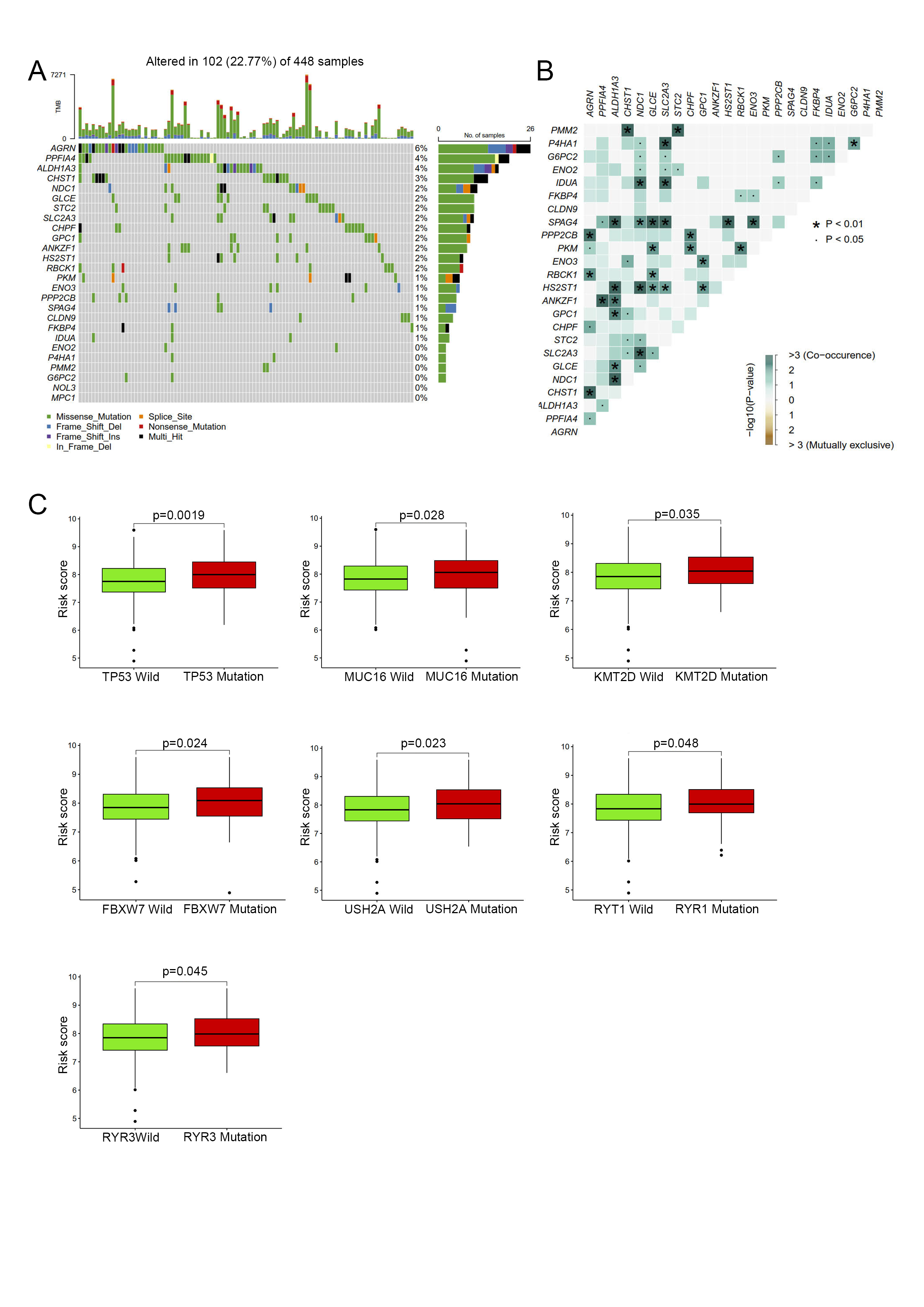

Supplement: Supplementary file 9 — Supplementary file9 (TIF 25522 KB) [file 535_2025_2281_MOESM9_ESM.tif]
