# Supplementary material for: The pivotal role of SFRP2 in promoting glycolysis and progression in the high-risk group based on the glycometabolism prognostic model for colorectal cancer
Source: J Gastroenterol. 2025 Jul 29;60(11):1400–13. doi: 10.1007/s00535-025-02281-5 (PMC12549743; doi:10.1007/s00535-025-02281-5)
Supplement: Supplementary file 10 — Supplementary file10 (PDF 57 KB) [file 535_2025_2281_MOESM10_ESM.pdf]

Table S1. Differentially expressed glycometabolism-related genes in CRC

| Gene | conMean | treatMean | logFC | pValue |
|---|---|---|---|---|
| ABCB6 | 0.038037 | 0.28388 | 2.899822 | 8.71E-20 |
| AGRN | 9.133359 | 21.01634 | 1.202294 | 4.22E-15 |
| ALG1 | 4.212846 | 6.393425 | 0.601794 | 2.81E-12 |
| ANKZF1 | 3.923532 | 8.249893 | 1.072223 | 1.93E-21 |
| ARTN | 0.479585 | 0.817268 | 0.769022 | 1.68E-05 |
| AURKA | 6.42352 | 20.46328 | 1.671602 | 4.36E-22 |
| B3GALT6 | 5.086505 | 10.0409 | 0.981142 | 4.33E-18 |
| B4GALT2 | 7.862717 | 12.3164 | 0.647481 | 2.50E-13 |
| B4GALT7 | 4.784505 | 8.081845 | 0.756315 | 4.72E-16 |
| BIK | 13.70462 | 26.19695 | 0.934737 | 6.63E-07 |
| BPNT1 | 27.67246 | 17.62124 | -0.65114 | 2.36E-12 |
| CACNA1H | 7.309202 | 2.288056 | -1.67559 | 1.86E-09 |
| CAPN5 | 71.13862 | 27.74505 | -1.3584 | 3.27E-22 |
| CD44 | 13.36517 | 38.32984 | 1.519989 | 7.91E-23 |
| CDK1 | 5.720651 | 19.0648 | 1.73666 | 9.11E-20 |
| CENPA | 2.279146 | 6.950611 | 1.608646 | 1.57E-21 |
| CHPF | 16.34387 | 57.94539 | 1.825944 | 8.15E-22 |
| CHST1 | 0.336561 | 0.669943 | 0.993171 | 8.52E-05 |
| CHST4 | 0.041027 | 1.781971 | 5.440762 | 4.25E-09 |
| CITED2 | 34.17846 | 11.25404 | -1.60264 | 3.50E-25 |
| CLDN9 | 0.212441 | 1.347629 | 2.665286 | 3.15E-09 |
| CLN6 | 3.225095 | 5.199652 | 0.689073 | 7.23E-13 |
| COL5A1 | 7.980417 | 27.12084 | 1.764866 | 2.57E-12 |
| DCN | 46.89864 | 15.77504 | -1.5719 | 3.06E-17 |
| DDIT4 | 8.201839 | 28.45664 | 1.794746 | 6.72E-18 |
| DEPDC1 | 1.295651 | 2.78955 | 1.106355 | 8.95E-11 |
| DSC2 | 24.63832 | 9.710886 | -1.34323 | 3.82E-19 |
| EFNA3 | 2.28298 | 9.233244 | 2.015919 | 2.79E-18 |
| EGFR | 9.166893 | 5.807241 | -0.65858 | 1.91E-12 |
| EGLN3 | 14.97254 | 8.64778 | -0.79192 | 1.21E-06 |
| ENO1 | 162.1306 | 306.5424 | 0.91893 | 1.28E-16 |
| ENO2 | 5.136454 | 7.859595 | 0.613682 | 0.025373 |
| ERO1A | 9.224422 | 21.42143 | 1.215524 | 1.16E-17 |
| FAM162A | 38.04927 | 19.55004 | -0.9607 | 4.43E-19 |
| FKBP4 | 23.15479 | 41.04853 | 0.82602 | 3.80E-15 |
| FUT8 | 3.758295 | 7.971394 | 1.084754 | 5.81E-11 |
| G6PD | 9.638224 | 16.44612 | 0.770908 | 8.84E-14 |
| GALK1 | 5.353144 | 14.43784 | 1.431397 | 1.50E-18 |
| GFUS | 23.67246 | 58.60423 | 1.307795 | 1.83E-16 |
| GLCE | 7.478022 | 12.57217 | 0.749505 | 3.31E-08 |
| GNE | 23.55847 | 13.56564 | -0.79629 | 4.34E-10 |
| GNPDA1 | 9.24242 | 19.3367 | 1.064999 | 5.58E-21 |

| | | | | |
|---|---|---|---|---|
| GOT1 | 47.47868 | 30.69441 | -0.6293 | 5.51E-14 |
| GPC1 | 4.076559 | 7.472444 | 0.874228 | 1.62E-05 |
| GPC4 | 10.03648 | 16.11877 | 0.683489 | 1.25E-06 |
| HK2 | 65.3668 | 29.77812 | -1.13431 | 3.90E-13 |
| HMMR | 2.913207 | 8.093919 | 1.47423 | 2.61E-15 |
| HOMER1 | 0.379988 | 1.876734 | 2.304199 | 5.65E-20 |
| HS2ST1 | 3.755837 | 6.550508 | 0.802472 | 5.28E-14 |
| HS6ST2 | 0.30328 | 4.006526 | 3.723627 | 1.45E-09 |
| IDUA | 1.893993 | 3.420046 | 0.852585 | 3.72E-06 |
| IER3 | 28.6445 | 78.54657 | 1.45529 | 2.30E-12 |
| IGFBP3 | 15.57239 | 33.17602 | 1.09115 | 4.59E-08 |
| IRS2 | 6.131351 | 11.16335 | 0.864493 | 3.60E-06 |
| ISG20 | 5.580259 | 3.462529 | -0.68851 | 4.31E-09 |
| KDELR3 | 10.88802 | 23.9371 | 1.136507 | 3.47E-12 |
| KIF20A | 2.548766 | 8.046723 | 1.658603 | 1.87E-22 |
| LDHA | 108.426 | 168.3611 | 0.634848 | 9.73E-11 |
| ME1 | 4.276 | 10.67559 | 1.319981 | 4.31E-11 |
| ME2 | 6.606244 | 4.180816 | -0.66005 | 1.72E-13 |
| MET | 6.6671 | 27.61458 | 2.050299 | 1.78E-24 |
| MIF | 0.008529 | 0.471394 | 5.788368 | 2.09E-08 |
| MIOX | 0.022798 | 0.303955 | 3.736905 | 1.77E-11 |
| MPI | 7.262151 | 4.179335 | -0.79712 | 2.25E-17 |
| MXI1 | 20.55016 | 7.495373 | -1.45508 | 4.41E-24 |
| NANP | 1.407549 | 3.882018 | 1.463622 | 9.35E-22 |
| NASP | 8.7012 | 14.30821 | 0.717557 | 2.17E-13 |
| NOL3 | 3.953527 | 8.092992 | 1.033533 | 2.36E-16 |
| NSDHL | 11.22121 | 24.27735 | 1.113382 | 1.55E-19 |
| P4HA1 | 11.3802 | 24.2312 | 1.090341 | 4.93E-13 |
| PAXIP1 | 1.289041 | 2.308963 | 0.840947 | 9.11E-20 |
| PC | 5.359666 | 2.76547 | -0.95462 | 2.61E-14 |
| PGK1 | 78.99459 | 142.4063 | 0.850187 | 3.77E-15 |
| PKM | 80.08129 | 167.9833 | 1.068781 | 3.86E-18 |
| PKP2 | 32.38963 | 20.67723 | -0.64749 | 7.83E-13 |
| PLOD1 | 16.0612 | 28.20445 | 0.812343 | 8.99E-14 |
| PLOD2 | 18.3626 | 10.70746 | -0.77815 | 6.25E-09 |
| PMM2 | 1.848205 | 3.019475 | 0.708173 | 2.04E-11 |
| POLR3K | 7.461959 | 16.06778 | 1.106545 | 1.38E-17 |
| PPFIA4 | 0.069824 | 0.19868 | 1.508644 | 5.17E-08 |
| PPIA | 43.6024 | 82.88487 | 0.926701 | 1.22E-19 |
| PPP2CB | 27.74237 | 17.73146 | -0.64578 | 1.39E-16 |
| PRPS1 | 6.180322 | 12.58283 | 1.025703 | 1.19E-18 |
| RBCK1 | 18.52425 | 36.94123 | 0.995816 | 1.29E-16 |
| RPE | 8.791227 | 13.32858 | 0.600387 | 1.84E-10 |
| SAP30 | 4.969685 | 8.477744 | 0.770526 | 4.31E-11 |

| | | | | |
|---|---|---|---|---|
| SLC25A10 | 4.830802 | 9.263624 | 0.939314 | 1.97E-08 |
| SOX9 | 18.40873 | 72.66089 | 1.980789 | 1.26E-23 |
| SPAG4 | 1.812085 | 3.225054 | 0.831672 | 5.25E-06 |
| SRD5A3 | 1.704627 | 3.300613 | 0.953278 | 3.72E-11 |
| STC1 | 0.935249 | 5.080574 | 2.441569 | 4.99E-17 |
| STC2 | 0.370234 | 5.254404 | 3.827017 | 2.56E-21 |
| STMN1 | 13.73934 | 28.91397 | 1.073454 | 3.25E-15 |
| TALDO1 | 50.17103 | 78.55536 | 0.646855 | 2.06E-16 |
| TGFA | 9.633146 | 5.551844 | -0.79504 | 3.27E-12 |
| TGFBI | 13.8901 | 141.1416 | 3.345014 | 2.70E-24 |
| UGP2 | 61.24278 | 15.58687 | -1.97421 | 3.62E-25 |
| VCAN | 2.589915 | 8.708128 | 1.749458 | 1.22E-08 |
| VEGFA | 4.231617 | 13.20553 | 1.641861 | 6.51E-23 |
| VLDLR | 1.130402 | 0.591443 | -0.93452 | 4.75E-10 |
| GAPDH | 529.3023 | 1001.181 | 0.919539 | 2.83E-15 |
| GPI | 27.61936 | 48.2463 | 0.804738 | 3.34E-18 |
| ACSS1 | 7.352539 | 11.30082 | 0.620114 | 0.002522 |
| ACSS2 | 59.37887 | 31.61166 | -0.90949 | 4.48E-18 |
| ADH1A | 0.101407 | 0.024696 | -2.03781 | 1.81E-22 |
| ADH1B | 12.15798 | 0.681472 | -4.15711 | 2.90E-25 |
| ADH1C | 258.6312 | 16.80441 | -3.94398 | 1.72E-25 |
| ADH6 | 4.991156 | 2.18429 | -1.19221 | 4.82E-14 |
| ALDH1A3 | 0.930737 | 1.742574 | 0.904775 | 0.000548 |
| ALDH1B1 | 36.65472 | 59.5789 | 0.700802 | 1.37E-06 |
| ALDH3B2 | 0.062422 | 0.772064 | 3.628596 | 1.00E-13 |
| ALDOC | 6.463163 | 13.10895 | 1.02024 | 1.58E-09 |
| ENO3 | 0.545237 | 1.083119 | 0.990238 | 1.18E-10 |
| G6PC2 | 0.025371 | 0.015167 | -0.74219 | 2.09E-06 |
| GALM | 35.8348 | 21.2111 | -0.75654 | 4.83E-17 |
| GCK | 0.073885 | 0.04382 | -0.75369 | 8.02E-12 |
| LDHB | 2.0949 | 6.776002 | 1.693553 | 4.99E-12 |
| PCK1 | 49.1795 | 7.377725 | -2.73681 | 2.55E-20 |
| PCK2 | 47.36876 | 27.43398 | -0.78797 | 4.95E-12 |
| PFKM | 6.349907 | 10.22318 | 0.687036 | 2.44E-10 |
| PGM1 | 42.09198 | 17.2501 | -1.28694 | 4.13E-23 |
| BID | 6.897146 | 14.86593 | 1.107938 | 5.19E-20 |
| CD4 | 15.14482 | 8.227313 | -0.88033 | 1.96E-11 |
| PFKFB3 | 6.757029 | 11.69762 | 0.791754 | 1.19E-10 |
| PFKFB4 | 1.767751 | 2.978976 | 0.752901 | 6.54E-08 |
| PRKACB | 27.42692 | 7.632625 | -1.84534 | 2.07E-23 |
| AAAS | 7.27278 | 11.35753 | 0.64307 | 4.72E-18 |
| GCKR | 0.018085 | 0.074388 | 2.040248 | 1.83E-07 |
| NDC1 | 8.716902 | 15.45894 | 0.826554 | 1.46E-13 |
| NUP107 | 3.10839 | 6.814172 | 1.132371 | 8.50E-22 |

| | | | | |
|---|---|---|---|---|
| NUP155 | 2.7261 | 6.319872 | 1.213057 | 4.85E-22 |
| NUP160 | 5.149571 | 8.371 | 0.700948 | 5.55E-16 |
| NUP188 | 6.093146 | 11.86046 | 0.960901 | 5.03E-19 |
| NUP205 | 6.215898 | 12.51364 | 1.009467 | 1.13E-20 |
| NUP210 | 5.701783 | 15.88084 | 1.477803 | 2.50E-15 |
| NUP35 | 2.261795 | 4.504118 | 0.993776 | 2.68E-18 |
| NUP37 | 4.254229 | 9.243867 | 1.119599 | 7.16E-21 |
| NUP42 | 1.947124 | 3.522388 | 0.855209 | 1.62E-14 |
| NUP43 | 5.696332 | 11.73366 | 1.042548 | 3.76E-22 |
| NUP58 | 3.616583 | 8.330756 | 1.20382 | 2.28E-17 |
| NUP62 | 8.884798 | 14.20324 | 0.676809 | 2.81E-18 |
| NUP85 | 5.68128 | 10.97579 | 0.950037 | 1.65E-22 |
| NUP93 | 3.258093 | 6.096482 | 0.903949 | 1.02E-21 |
| PGM2L1 | 2.008732 | 3.051846 | 0.603397 | 0.001977 |
| PGP | 4.557273 | 9.339294 | 1.035143 | 4.66E-14 |
| RAE1 | 4.743324 | 13.02601 | 1.457425 | 1.44E-23 |
| SEH1L | 2.954444 | 4.846951 | 0.714191 | 6.55E-13 |
| SLC2A1 | 12.73816 | 46.47232 | 1.867214 | 3.00E-17 |
| MPC1 | 50.36293 | 24.36002 | -1.04785 | 7.77E-21 |
| SLC2A3 | 3.074461 | 6.412254 | 1.060498 | 0.001577 |
| SLC2A4 | 4.810339 | 0.863293 | -2.47822 | 8.00E-16 |
| SLC2A5 | 2.610144 | 1.01843 | -1.35778 | 6.32E-13 |
