# Supplementary material for: The pivotal role of SFRP2 in promoting glycolysis and progression in the high-risk group based on the glycometabolism prognostic model for colorectal cancer
Source: J Gastroenterol. 2025 Jul 29;60(11):1400–13. doi: 10.1007/s00535-025-02281-5 (PMC12549743; doi:10.1007/s00535-025-02281-5)
Supplement: Supplementary file 11 — Supplementary file11 (PDF 44 KB) [file 535_2025_2281_MOESM11_ESM.pdf]

Table S2. Univariable Cox regression analysis of the glycometabolism-related genes in overall survival for CRC patients

GeneName	HR	HR.95L	HR.95H	pvalue
AGRN	1.625918	1.123125	2.353798	0.010021
ANKZF1	2.247237	1.327535	3.8041	0.00257
CHPF	1.376359	1.010551	1.874586	0.042709
CHST1	1.781841	1.067389	2.974507	0.027147
CLDN9	1.511318	1.103354	2.070128	0.010092
ENO2	1.293172	1.07003	1.562847	0.007806
FKBP4	1.605425	1.08361	2.378522	0.018259
GLCE	0.745526	0.573959	0.968376	0.027751
GPC1	1.499525	1.119002	2.009447	0.006671
HS2ST1	0.676422	0.45763	0.999817	0.049893
IDUA	1.659502	1.101229	2.500794	0.015486
NOL3	2.316646	1.516609	3.538714	0.000102
P4HA1	1.54147	1.107613	2.14527	0.010287
PKM	1.407688	1.006354	1.969073	0.04583
PMM2	0.475201	0.289399	0.780293	0.003278
PPFIA4	3.571931	1.818546	7.015875	0.000219
PPP2CB	0.550565	0.346641	0.874454	0.011462
RBCK1	1.480751	1.008948	2.173178	0.044912
SPAG4	1.419883	1.070839	1.8827	0.014874
STC2	1.305001	1.059436	1.607486	0.012322
ALDH1A3	1.506438	1.038044	2.186186	0.031047
ENO3	3.275219	1.873114	5.726857	3.16E-05
G6PC2	0.010613	0.000194	0.580033	0.025962
NDC1	0.708707	0.514953	0.975362	0.034595
MPC1	0.564125	0.359634	0.884891	0.012689
SLC2A3	1.326697	1.104901	1.593017	0.002456
