# Supplementary material for: The pivotal role of SFRP2 in promoting glycolysis and progression in the high-risk group based on the glycometabolism prognostic model for colorectal cancer
Source: J Gastroenterol. 2025 Jul 29;60(11):1400–13. doi: 10.1007/s00535-025-02281-5 (PMC12549743; doi:10.1007/s00535-025-02281-5)
Supplement: Supplementary file 12 — Supplementary file12 (PDF 59 KB) [file 535_2025_2281_MOESM12_ESM.pdf]

Table S3. The information and Coefficient of the glycometabolism-related signature in the LASSO Cox regression analysis

No.	Gene	Full Name	Function	Coef
1	ANKZF1	Ankyrin Repeat And Zinc Finger Peptidyl TRNA Hydrolase 1	Plays a role in the cellular response to hydrogen peroxide and in the maintenance of mitochondrial integrity under conditions of cellular stress.<br>Involved in the endoplasmic reticulum (ER)-associated degradation (ERAD) pathway.	0.260479
2	CHST1	Carbohydrate Sulfotransferase 1	Sulfotransferase that utilizes 3'-phospho-5'-adenylyl sulfate (PAPS) as sulfonate donor to catalyze the transfer of sulfate to position 6 of internal galactose (Gal) residues of keratan.<br>May act in a synergistic manner with CHST4 to generate sialyl 6',6-disulfo Lewis X motif, a recognition determinant for immune cell receptors implicated in leukocyte trafficking.<br>Catalyzes sulfation of N-acetyllactosamine (LacNAc) oligosaccharides with highest efficiency for sialylated LacNAc structures .	0.183322
3	CLDN9	Claudin-9	Plays a major role in tight junction-specific obliteration of the intercellular space, through calcium-independent cell-adhesion activity.<br>Acts as a receptor for hepatitis C virus (HCV) entry into hepatic cells.	0.082092
4	FKBP4	FKBP Prolyl Isomerase 4	Immunophilin protein with PPIase and co-chaperone activities.<br>Acts also as a regulator of microtubule dynamics by inhibiting MAPT/TAU ability to promote microtubule assembly.<br>Component of steroid receptors heterocomplexes through interaction with heat-shock protein 90 (HSP90).	0.422546
5	GLCE	Glucuronic Acid Epimerase	Enables calcium ion binding activity, heparosan-N-sulfate-glucuronate 5-epimerase activity, and protein homodimerization activity.<br>Involved in heparan sulfate proteoglycan biosynthetic process.	-0.05152
6	GPC1	Glypican 1	Cell surface proteoglycan that bears heparan sulfate.<br>Required for proper cell differentiation by	0.058003

			sequestering FGF2 in lipid rafts preventing its binding to receptors (FGFRs) and inhibiting the FGF-mediated signaling.	
7	IDUA	Alpha-L-Iduronidas e	Hydrolyzing dermatan sulfate, heparan sulfate,catalyzing the second step of degradation of glucosaminoglycans	0.033317
8	NOL3	Nucleolar Protein 3	Functions as an apoptosis repressor that blocks multiple modes of cell death.<br>Interacting with FAS and FADD upon FAS activation blocking death-inducing signaling complex (DISC) assembly.<br>Interacting with CASP8 in a mitochondria localization- and phosphorylation-dependent manner, limiting the amount of soluble CASP8 available for DISC-mediated activation.	0.456214
9	P4HA1	Prolyl 4-Hydroxylase Subunit Alpha 1	Catalyzes the post-translational formation of 4-hydroxyproline in -Xaa-Pro-Gly- sequences in collagens and other proteins.	0.547072
10	PMM2	Phosphomannomuta se 2	Involved in the synthesis of the GDP-mannose and dolichol-phosphate-mannose required for a number of critical mannosyl transfer reactions.	-0.31426
11	PPP2CB	Protein Phosphatase 2 Catalytic Subunit Beta	Catalytic subunit of protein phosphatase 2A (PP2A), a serine/threonine phosphatase involved in the regulation of the activity of phosphorylase B kinase, casein kinase 2, mitogen-stimulated S6 kinase, and MAP-2 kinase.	-0.324
12	RBCK1	RANBP2-Type And C3HC4-Type Zinc Finger	Functions as an E3 ligase for oxidized IREB2 and both heme and oxygen are necessary for IREB2 ubiquitination.<br>Promotes ubiquitination of TAB2 and IRF3 and their degradation by the proteasome	0.198214
13	SPAG4	Sperm Associated Antigen 4	Involved in maintenance of the nuclear envelope integrity.<br>Required for targeting of SUN3 and probably SYNE1 through a probable SUN1:SYNE3 LINC complex to the nuclear envelope and involved in accurate posterior sperm head localization of the complex.	0.065966
14	STC2	Stanniocalcin 2	Has an anti-hypocalcemic action on calcium and phosphate homeostasis	0.129602
15	ALDH1A 3	Aldehyde Dehydrogenase 1	NAD-dependent aldehyde dehydrogenase that catalyzes the formation of retinoic acid .	0.231511

		Family Member A3	Required for the biosynthesis of normal levels of retinoic acid in the embryonic ocular and nasal regions.	
16	ENO3	Enolase 3	Glycolytic enzyme that catalyzes the conversion of 2-phosphoglycerate to phosphoenolpyruvate.	0.465562
17	G6PC2	Glucose-6-Phosphatase Catalytic Subunit 2	May be responsible for glucose production through glycogenolysis and gluconeogenesis. May hydrolyze glucose-6-phosphate to glucose in the endoplasmic reticulum.	-0.92638
18	NDC1	NDC1 Transmembrane Nucleoporin	Component of the nuclear pore complex (NPC), which plays a key role in de novo assembly and insertion of NPC in the nuclear envelope. Required for NPC and nuclear envelope assembly, possibly by forming a link between the nuclear envelope membrane and soluble nucleoporins, thereby anchoring the NPC in the membrane.	-0.07933
19	SLC2A3	Solute Carrier Family 2 Member 3	Facilitative glucose transporter that can also mediate the uptake of various other monosaccharides across the cell membrane.Mediates the uptake of glucose, 2-deoxyglucose, galactose, mannose, xylose and fucose, and probably also dehydroascorbate.	0.093052
