# Supplementary material for: The pivotal role of SFRP2 in promoting glycolysis and progression in the high-risk group based on the glycometabolism prognostic model for colorectal cancer
Source: J Gastroenterol. 2025 Jul 29;60(11):1400–13. doi: 10.1007/s00535-025-02281-5 (PMC12549743; doi:10.1007/s00535-025-02281-5)
Supplement: Supplementary file 13 — Supplementary file13 (PDF 42 KB) [file 535_2025_2281_MOESM13_ESM.pdf]

Table S4. Correlation between the risk score and clinicopathological features in the TCGA cohort

Clinicopathological variables		Risk Score		p Value
		Low (n=224)	High (n=224)	
Age		67.35(13.05)	66.58(12.71)	0.634
Sex	female	108(48%)	106(47%)	0.925
	male	116(52%)	118(53%)	
Tumor invasion	T1	8(4%)	2(1%)	0.001
	T2	52(23%)	24(11%)	
	T3	146(65%)	159(71%)	
	T4	17(8%)	39(17%)	
Lymph node metastasis	N0	155(69%)	111(50%)	0.001
	N1	50(22%)	52(23%)	
	N2	19(8%)	61(27%)	
Distant metastasis	M0	173(90%)	157(78%)	0.003
	M1	19(10%)	43(22%)	
AJCC stage	Stage I	54(25%)	21(10%)	0.001
	Stage II	92(42%)	84(39%)	
	Stage III	54(25%)	70(32%)	
	Stage IV	19(9%)	43(20%)	
