# Supplementary material for: The pivotal role of SFRP2 in promoting glycolysis and progression in the high-risk group based on the glycometabolism prognostic model for colorectal cancer
Source: J Gastroenterol. 2025 Jul 29;60(11):1400–13. doi: 10.1007/s00535-025-02281-5 (PMC12549743; doi:10.1007/s00535-025-02281-5)
Supplement: Supplementary file 14 — Supplementary file14 (PDF 43 KB) [file 535_2025_2281_MOESM14_ESM.pdf]

Table S5. Correlation between the risk score and clinicopathological features in the GEO (GSE39582) cohort

Clinicopathological variables		Risk Score		p Value
		Low (n=286)	High (n=293)	
Age		66.74(12.18)	65.53(11.32)	0.541
Sex	female	119(42%)	141(48%)	0.136
	male	167(58%)	152(52%)	
Tumor invasion	T1	9(3%)	3(1%)	0.027
	T2	26(10%)	22(8%)	
	T3	190(70%)	186(65%)	
	T4	46(17%)	73(26%)	
Lymph node metastasis	N0	166(61%)	145(52%)	0.032
	N1-2	106(39%)	136(48%)	
Distant metastasis	M0	253(92%)	243(86%)	0.039
	M1	22(8%)	39(14%)	
AJCC stage	Stage I	25(9%)	12(4%)	0.032
	Stage II	137(49%)	132(45%)	
	Stage III	97(34%)	112(38%)	
	Stage IV	23(8%)	37(13%)	
