# Supplementary material for: The pivotal role of SFRP2 in promoting glycolysis and progression in the high-risk group based on the glycometabolism prognostic model for colorectal cancer
Source: J Gastroenterol. 2025 Jul 29;60(11):1400–13. doi: 10.1007/s00535-025-02281-5 (PMC12549743; doi:10.1007/s00535-025-02281-5)
Supplement: Supplementary file 15 — Supplementary file15 (PDF 50 KB) [file 535_2025_2281_MOESM15_ESM.pdf]

Table S6. Comparison of the differential gene expression profile between high-risk and low-risk gruops

Gene	lowMean	highMean	logFC	pValue	fdr
NXPH4	1.712187202	7.527958854	2.136418295	2.49E-12	4.50E-10
IGFL1	0.394873214	1.938533557	2.295504296	0.000431714	0.001726279
COMP	4.730685268	12.16522842	1.362642323	5.75E-07	8.01E-06
CLCA4	13.53599807	4.274328869	-1.663031448	0.000137897	0.000668441
GFPT2	1.305097098	3.178868601	1.284356238	1.64E-07	2.88E-06
TMEM252	3.408231994	1.374359375	-1.310264244	0.000129971	0.000636308
CILP	0.670018527	1.488739732	1.151818664	0.00484548	0.012730843
CTXN1	2.267579092	5.071649926	1.161302294	6.01E-12	9.21E-10
WNT10A	0.81452686	1.748175818	1.101816108	0.000195572	0.000893275
CDKN2A	1.439032292	3.05296942	1.085114172	3.71E-05	0.000231619
IGHG4	58.38391109	134.4388905	1.203307777	0.013234536	0.029030909
VGF	0.994286235	4.685053869	2.236332496	1.36E-07	2.49E-06
KRT16	0.695930208	3.436027307	2.303726965	4.13E-06	3.98E-05
KRT14	0.065680729	4.60818817	6.132585675	0.002368134	0.007074786
MIR4728	0.637401339	1.904229464	1.578933382	0.008446376	0.020050748
ISYNA1	4.728736979	9.8776875	1.062718428	8.79E-12	1.24E-09
SNORA14B	2.88851369	1.1403	-1.340913901	0.000621383	0.00234176
PLIN4	0.76915878	3.128787054	2.024250118	1.26E-05	9.60E-05
SFRP2	20.46944762	51.4476003	1.329631615	4.40E-07	6.50E-06
FBXL16	1.541915997	3.222794122	1.063587859	6.30E-11	5.71E-09
KRT5	1.44347567	5.426184896	1.910391417	0.00404481	0.01096238
G0S2	12.31786734	25.07833415	1.025689024	5.81E-08	1.24E-06
CLCA1	155.318258	64.94059784	-1.25803486	0.001031919	0.003531587
SERPINE1	13.20152567	29.22789844	1.146641431	7.86E-06	6.60E-05
NNAT	1.304617336	6.753212946	2.371947345	0.001624953	0.005161818
HSPA6	1.690766071	3.594701563	1.088194934	5.13E-09	1.73E-07
CALB2	1.218856101	3.551997842	1.543102895	1.68E-05	0.000120986
CSAG1	0.737878497	1.775775298	1.266993859	0.002285629	0.006853602
RRAD	1.793332068	3.691324628	1.041495964	7.61E-05	0.000412546
UCHL1	1.550980878	4.770428348	1.620937916	8.16E-06	6.81E-05
AC012354.2	0.734163839	1.516827083	1.046882667	0.010128117	0.02330833
MIR4539	0.836135938	1.901268006	1.185152494	0.024891392	0.048778143
KRT17	7.758588839	16.61909836	1.098975935	0.012114975	0.027011622
TNNT1	1.581678199	3.862063839	1.287915906	1.51E-09	6.64E-08
IGFBP6	5.769333929	11.59740536	1.007325399	1.65E-06	1.86E-05
MIR210	0.543423214	1.675686086	1.624603806	2.72E-13	8.99E-11
HCAR2	0.692847173	1.719648586	1.311504713	0.002991688	0.008542999
KREMEN2	0.649296354	1.419404092	1.128336357	3.05E-13	8.99E-11
MARCO	1.838425372	4.116081101	1.162800795	1.31E-06	1.54E-05
CST6	1.099342039	2.547123586	1.212228638	0.00034185	0.001416744
FABP4	1.108221429	8.682454836	2.969856833	0.007783079	0.018777723

CYP1B1	0.705286012	1.705683482	1.274069624	0.000262008	0.001137154
UGT2B7	4.207360119	1.977177381	-1.089473	1.40E-05	0.000104676
BAIAP3	0.678286905	1.467718378	1.113607629	2.78E-12	4.95E-10
NTSR1	0.532322098	1.546990774	1.53909323	4.67E-06	4.38E-05
UGT2A3	6.790076637	2.891996503	-1.23136205	2.31E-07	3.83E-06
EPHB6	0.417357813	3.151208333	2.916548455	1.56E-09	6.83E-08
PAEP	0.21367433	12.98404122	5.925182074	2.67E-05	0.000175276
AMH	0.980310491	2.367006548	1.271753029	2.06E-05	0.000143521
