# Supplementary material for: The pivotal role of SFRP2 in promoting glycolysis and progression in the high-risk group based on the glycometabolism prognostic model for colorectal cancer
Source: J Gastroenterol. 2025 Jul 29;60(11):1400–13. doi: 10.1007/s00535-025-02281-5 (PMC12549743; doi:10.1007/s00535-025-02281-5)
Supplement: Supplementary file 16 — Supplementary file16 (PDF 45 KB) [file 535_2025_2281_MOESM16_ESM.pdf]

Table S7. Univariable Cox regression analysis of the differential gene expression profile between high-risk and low-risk gruops in overall survival for CRC patients

Gene	HR	HR.95L	HR.95H	pvalue
CLCA4	0.876134	0.769175	0.997966	0.046523
WNT10A	1.237252	1.008591	1.517754	0.04115
CDKN2A	1.281775	1.074598	1.528895	0.005784
VGF	1.193416	1.007586	1.413518	0.040611
ISYNA1	1.352037	1.125498	1.624173	0.001266
SFRP2	1.110648	1.015988	1.214127	0.020947
CLCA1	0.92715	0.864377	0.994482	0.03446
SERPINE1	1.202128	1.038627	1.391369	0.013585
CALB2	1.323927	1.133031	1.546985	0.000412
CSAG1	1.272312	1.115939	1.450596	0.000319
UCHL1	1.266232	1.045012	1.534282	0.01598
AC012354.2	1.295856	1.087289	1.54443	0.003796
TNNT1	1.273113	1.10939	1.460999	0.000586
MIR210	1.357849	1.082363	1.703451	0.008192
KREMEN2	1.477684	1.098216	1.988271	0.009918
FABP4	1.185065	1.049732	1.337846	0.006062
UGT2B7	0.804338	0.647403	0.999315	0.049282
AMH	1.310129	1.07154	1.601842	0.008448
