# Supplementary material for: The pivotal role of SFRP2 in promoting glycolysis and progression in the high-risk group based on the glycometabolism prognostic model for colorectal cancer
Source: J Gastroenterol. 2025 Jul 29;60(11):1400–13. doi: 10.1007/s00535-025-02281-5 (PMC12549743; doi:10.1007/s00535-025-02281-5)
Supplement: Supplementary file 17 — Supplementary file17 (PDF 67 KB) [file 535_2025_2281_MOESM17_ESM.pdf]

Table S8. Correlation between SFRP2 expression and clinicopathological characteristics of CRC based on immunohistochemical staining

Clinicopathological variables		Tumor SFRP2 expression (n=104)		p Value
		Negative (n=64)	Positive (n=40)	
Age		66.97(11.449)	70.30(10.447)	0.453
Sex	female	25	20	0.225
	male	39	19	
Tumor size	＜5cm	26	14	0.589
	⩾5cm	37	25	
Tumor differentiation	well or moderate	38	19	0.236
	poor	26	21	
Tumor invasion	T1	1	0	0.005
	T2	4	0	
	T3	55	25	
	T4	4	11	
Lymph node metastasis	absent	51	12	<0.001
	present	13	27	
Distant metastasis	absent	64	37	0.026
	present	0	3	
AJCC stage	Stage I	4	0	<0.001
	Stage II	48	12	
	Stage III	11	25	
	Stage IV	1	2	
