# Supplementary material for: The pivotal role of SFRP2 in promoting glycolysis and progression in the high-risk group based on the glycometabolism prognostic model for colorectal cancer
Source: J Gastroenterol. 2025 Jul 29;60(11):1400–13. doi: 10.1007/s00535-025-02281-5 (PMC12549743; doi:10.1007/s00535-025-02281-5)
Supplement: Supplementary file 18 — Supplementary file18 (PDF 68 KB) [file 535_2025_2281_MOESM18_ESM.pdf]

Table S9. Correlation between ENO2 expression and clinicopathological characteristics of CRC based on immunohistochemical staining

Clinicopathological variables		Tumor ENO2 expression (n=104) Negative (n=53)	Positive (n=45)	p Value
Age		68.72(9.908)	67.87(12.495)	0.336
Sex	female	28	17	0.372
	male	31	27	
Tumor size	＜5cm	26	14	0.081
	⩾5cm	37	25	
Tumor differentiation	well or moderate	27	13	0.064
	poor	31	31	
Tumor invasion	T1	1	0	0.152
	T2	3	1	
	T3	49	31	
	T4	5	10	
Lymph node metastasis	absent	48	15	<0.001
	present	11	29	
Distant metastasis	absent	52	42	0.044
	present	0	3	
AJCC stage	Stage I	4	0	<0.001
	Stage II	45	15	
	Stage III	10	26	
	Stage IV	0	3	
