# Supplementary material for: The pivotal role of SFRP2 in promoting glycolysis and progression in the high-risk group based on the glycometabolism prognostic model for colorectal cancer
Source: J Gastroenterol. 2025 Jul 29;60(11):1400–13. doi: 10.1007/s00535-025-02281-5 (PMC12549743; doi:10.1007/s00535-025-02281-5)
Supplement: Supplementary file 19 — Supplementary file19 (DOCX 1870 KB) [file 535_2025_2281_MOESM19_ESM.docx]

Table S10. Tumor Mutation Burden (TMB) and Mutation Annotation Format (MAF) analysis of the somatic mutation in CRC

| Gene Name | Num of Cases with mutation （448 cases in total） | Mutaton Rate |
| --- | --- | --- |
| APC | 299 | 0.710213777 |
| TP53 | 220 | 0.522565321 |
| TTN | 202 | 0.479809976 |
| KRAS | 182 | 0.432304038 |
| PIK3CA | 126 | 0.299287411 |
| MUC16 | 122 | 0.289786223 |
| SYNE1 | 122 | 0.289786223 |
| FAT4 | 99 | 0.235154394 |
| ZFHX4 | 85 | 0.201900238 |
| OBSCN | 84 | 0.199524941 |
| RYR2 | 84 | 0.199524941 |
| DNAH5 | 78 | 0.185273159 |
| CSMD3 | 75 | 0.178147268 |
| LRP1B | 74 | 0.175771971 |
| PCLO | 72 | 0.171021378 |
| FBXW7 | 70 | 0.166270784 |
| RYR3 | 70 | 0.166270784 |
| USH2A | 69 | 0.163895487 |
| DNAH11 | 68 | 0.16152019 |
| ABCA13 | 67 | 0.159144893 |
| CSMD1 | 66 | 0.156769596 |
| ADGRV1 | 65 | 0.154394299 |
| RYR1 | 65 | 0.154394299 |
| FAT3 | 63 | 0.149643705 |
| NEB | 63 | 0.149643705 |
| DST | 61 | 0.144893112 |
| MDN1 | 60 | 0.142517815 |
| LRP2 | 60 | 0.142517815 |
| RNF43 | 60 | 0.142517815 |
| BRAF | 60 | 0.142517815 |
| SOX9 | 60 | 0.142517815 |
| KMT2D | 59 | 0.140142518 |
| DOCK3 | 59 | 0.140142518 |
| SDK1 | 58 | 0.137767221 |
| AMER1 | 57 | 0.135391924 |
| CACNA1E | 57 | 0.135391924 |
| PCDH15 | 56 | 0.133016627 |
| CSMD2 | 56 | 0.133016627 |
| MUC5B | 55 | 0.13064133 |
| ARID1A | 55 | 0.13064133 |
| KIAA1109 | 54 | 0.128266033 |
| DMD | 54 | 0.128266033 |
| FLG | 53 | 0.125890736 |
| ATM | 53 | 0.125890736 |
| ANK3 | 53 | 0.125890736 |
| SACS | 53 | 0.125890736 |
| HMCN1 | 53 | 0.125890736 |
| SYNE2 | 52 | 0.123515439 |
| TRPS1 | 52 | 0.123515439 |
| ANK2 | 52 | 0.123515439 |
| TNXB | 51 | 0.121140143 |
| HYDIN | 51 | 0.121140143 |
| KMT2B | 51 | 0.121140143 |
| SPTA1 | 51 | 0.121140143 |
| ZFHX3 | 50 | 0.118764846 |
| VPS13B | 50 | 0.118764846 |
| XIRP2 | 50 | 0.118764846 |
| DNAH8 | 50 | 0.118764846 |
| BMPR2 | 50 | 0.118764846 |
| DNAH10 | 50 | 0.118764846 |
| KMT2C | 49 | 0.116389549 |
| DSCAM | 49 | 0.116389549 |
| ACVR2A | 49 | 0.116389549 |
| DNAH3 | 48 | 0.114014252 |
| PCDH17 | 48 | 0.114014252 |
| HERC2 | 48 | 0.114014252 |
| UNC13C | 48 | 0.114014252 |
| NBEA | 48 | 0.114014252 |
| MACF1 | 48 | 0.114014252 |
| TENM3 | 47 | 0.111638955 |
| ROBO2 | 47 | 0.111638955 |
| COL12A1 | 47 | 0.111638955 |
| MYCBP2 | 47 | 0.111638955 |
| FAT2 | 46 | 0.109263658 |
| SMAD4 | 45 | 0.106888361 |
| DNAH17 | 45 | 0.106888361 |
| DSCAML1 | 45 | 0.106888361 |
| FRY | 45 | 0.106888361 |
| NRXN1 | 45 | 0.106888361 |
| PCDH9 | 45 | 0.106888361 |
| AHNAK2 | 45 | 0.106888361 |
| COL6A3 | 45 | 0.106888361 |
| ZNF536 | 45 | 0.106888361 |
| PRKDC | 45 | 0.106888361 |
| CUBN | 45 | 0.106888361 |
| FAT1 | 44 | 0.104513064 |
| FCGBP | 44 | 0.104513064 |
| DNAH7 | 44 | 0.104513064 |
| DNAH2 | 44 | 0.104513064 |
| LAMA2 | 44 | 0.104513064 |
| NFASC | 44 | 0.104513064 |
| MXRA5 | 44 | 0.104513064 |
| TSHZ3 | 44 | 0.104513064 |
| RNF213 | 43 | 0.102137767 |
| LRRK2 | 43 | 0.102137767 |
| LAMA1 | 43 | 0.102137767 |
| COL5A1 | 43 | 0.102137767 |
| NAV3 | 43 | 0.102137767 |
| LRP1 | 43 | 0.102137767 |
| FLNC | 43 | 0.102137767 |
| HECTD4 | 43 | 0.102137767 |
| RELN | 43 | 0.102137767 |
| PCDH10 | 43 | 0.102137767 |
| SRCAP | 43 | 0.102137767 |
| SCN1A | 42 | 0.09976247 |
| BSN | 42 | 0.09976247 |
| ASPM | 42 | 0.09976247 |
| ERBB4 | 42 | 0.09976247 |
| UNC79 | 42 | 0.09976247 |
| COL11A1 | 42 | 0.09976247 |
| DNAH9 | 42 | 0.09976247 |
| IGF2R | 42 | 0.09976247 |
| DNAH1 | 42 | 0.09976247 |
| NIPBL | 42 | 0.09976247 |
| TNC | 42 | 0.09976247 |
| ZNF831 | 42 | 0.09976247 |
| FREM2 | 42 | 0.09976247 |
| AKAP9 | 42 | 0.09976247 |
| CHD7 | 41 | 0.097387173 |
| NALCN | 41 | 0.097387173 |
| CACNA1H | 41 | 0.097387173 |
| HECW1 | 41 | 0.097387173 |
| GRIK2 | 41 | 0.097387173 |
| MUC17 | 41 | 0.097387173 |
| PTPRT | 41 | 0.097387173 |
| PLXNA4 | 41 | 0.097387173 |
| DYSF | 41 | 0.097387173 |
| FNDC1 | 41 | 0.097387173 |
| DYNC2H1 | 41 | 0.097387173 |
| TENM1 | 40 | 0.095011876 |
| RIMS1 | 40 | 0.095011876 |
| DCHS1 | 40 | 0.095011876 |
| FRYL | 40 | 0.095011876 |
| PCDH11X | 40 | 0.095011876 |
| PDZD2 | 40 | 0.095011876 |
| GLI3 | 40 | 0.095011876 |
| RIMS2 | 40 | 0.095011876 |
| ASH1L | 40 | 0.095011876 |
| COL6A6 | 40 | 0.095011876 |
| PLEC | 40 | 0.095011876 |
| APOB | 39 | 0.09263658 |
| FRAS1 | 39 | 0.09263658 |
| MTOR | 39 | 0.09263658 |
| IGSF9B | 39 | 0.09263658 |
| ROBO1 | 39 | 0.09263658 |
| PKHD1 | 39 | 0.09263658 |
| MEGF8 | 39 | 0.09263658 |
| KMT2A | 39 | 0.09263658 |
| FBN3 | 39 | 0.09263658 |
| ANKRD11 | 39 | 0.09263658 |
| TAF1L | 39 | 0.09263658 |
| VWF | 39 | 0.09263658 |
| CREBBP | 39 | 0.09263658 |
| PTPRS | 39 | 0.09263658 |
| AHNAK | 39 | 0.09263658 |
| CELSR1 | 39 | 0.09263658 |
| ZDBF2 | 39 | 0.09263658 |
| ABCA1 | 38 | 0.090261283 |
| CDH10 | 38 | 0.090261283 |
| CNOT1 | 38 | 0.090261283 |
| TMEM132D | 38 | 0.090261283 |
| TECTA | 38 | 0.090261283 |
| ATP10A | 38 | 0.090261283 |
| RP1 | 38 | 0.090261283 |
| PXDN | 38 | 0.090261283 |
| CACNA1A | 38 | 0.090261283 |
| LRRIQ1 | 38 | 0.090261283 |
| PAPPA2 | 38 | 0.090261283 |
| COL7A1 | 38 | 0.090261283 |
| TCF7L2 | 38 | 0.090261283 |
| LAMA5 | 38 | 0.090261283 |
| TNR | 37 | 0.087885986 |
| SVIL | 37 | 0.087885986 |
| TRIO | 37 | 0.087885986 |
| SCN9A | 37 | 0.087885986 |
| SPEG | 37 | 0.087885986 |
| GRIN2A | 37 | 0.087885986 |
| VCAN | 37 | 0.087885986 |
| SCN5A | 37 | 0.087885986 |
| TRRAP | 37 | 0.087885986 |
| COL27A1 | 36 | 0.085510689 |
| BCL9L | 36 | 0.085510689 |
| MAP3K4 | 36 | 0.085510689 |
| PKHD1L1 | 36 | 0.085510689 |
| MAP2 | 36 | 0.085510689 |
| BIRC6 | 36 | 0.085510689 |
| FBN2 | 36 | 0.085510689 |
| AKAP12 | 36 | 0.085510689 |
| MBD6 | 36 | 0.085510689 |
| ROS1 | 36 | 0.085510689 |
| TENM2 | 36 | 0.085510689 |
| DOCK2 | 36 | 0.085510689 |
| VPS13A | 36 | 0.085510689 |
| BNC2 | 36 | 0.085510689 |
| BCL9 | 35 | 0.083135392 |
| DYNC1H1 | 35 | 0.083135392 |
| PCDHB8 | 35 | 0.083135392 |
| PCDHA2 | 35 | 0.083135392 |
| MYO15A | 35 | 0.083135392 |
| MAP1B | 35 | 0.083135392 |
| MYO18B | 35 | 0.083135392 |
| RP1L1 | 35 | 0.083135392 |
| VPS13C | 35 | 0.083135392 |
| ITPR3 | 35 | 0.083135392 |
| ABCA12 | 35 | 0.083135392 |
| SDK2 | 35 | 0.083135392 |
| ZBTB20 | 35 | 0.083135392 |
| CNTN6 | 35 | 0.083135392 |
| ADAMTS16 | 35 | 0.083135392 |
| CTNNA2 | 35 | 0.083135392 |
| MAGEC1 | 35 | 0.083135392 |
| FBN1 | 35 | 0.083135392 |
| CMYA5 | 35 | 0.083135392 |
| TEX15 | 35 | 0.083135392 |
| UBR4 | 35 | 0.083135392 |
| EP400 | 34 | 0.080760095 |
| USP34 | 34 | 0.080760095 |
| MINAR1 | 34 | 0.080760095 |
| MYO16 | 34 | 0.080760095 |
| TNS1 | 34 | 0.080760095 |
| RIF1 | 34 | 0.080760095 |
| MKI67 | 34 | 0.080760095 |
| NAV2 | 34 | 0.080760095 |
| CDH23 | 34 | 0.080760095 |
| SPAG17 | 34 | 0.080760095 |
| ZNF292 | 34 | 0.080760095 |
| PCNT | 34 | 0.080760095 |
| BRWD3 | 34 | 0.080760095 |
| NCKAP5 | 34 | 0.080760095 |
| DCC | 34 | 0.080760095 |
| MYO3A | 34 | 0.080760095 |
| HUWE1 | 34 | 0.080760095 |
| BCORL1 | 34 | 0.080760095 |
| CHD8 | 34 | 0.080760095 |
| ADAMTS2 | 34 | 0.080760095 |
| FN1 | 34 | 0.080760095 |
| HERC1 | 34 | 0.080760095 |
| NOS1 | 33 | 0.078384798 |
| COL22A1 | 33 | 0.078384798 |
| UBR5 | 33 | 0.078384798 |
| SPEF2 | 33 | 0.078384798 |
| CACNA1B | 33 | 0.078384798 |
| ZNF423 | 33 | 0.078384798 |
| CNTNAP5 | 33 | 0.078384798 |
| PTPRK | 33 | 0.078384798 |
| F8 | 33 | 0.078384798 |
| A2ML1 | 33 | 0.078384798 |
| SCN11A | 33 | 0.078384798 |
| SCN7A | 33 | 0.078384798 |
| ADGRL3 | 33 | 0.078384798 |
| CDH9 | 33 | 0.078384798 |
| PCDHA12 | 33 | 0.078384798 |
| PXDNL | 33 | 0.078384798 |
| TRIOBP | 33 | 0.078384798 |
| OTOGL | 33 | 0.078384798 |
| KANSL1 | 33 | 0.078384798 |
| TG | 33 | 0.078384798 |
| EVC2 | 33 | 0.078384798 |
| THSD7B | 33 | 0.078384798 |
| KIF21A | 32 | 0.076009501 |
| FHOD3 | 32 | 0.076009501 |
| FMN2 | 32 | 0.076009501 |
| CNTNAP4 | 32 | 0.076009501 |
| PREX2 | 32 | 0.076009501 |
| STAB1 | 32 | 0.076009501 |
| CUX2 | 32 | 0.076009501 |
| ADGRL2 | 32 | 0.076009501 |
| CHD4 | 32 | 0.076009501 |
| ERICH3 | 32 | 0.076009501 |
| PRDM9 | 32 | 0.076009501 |
| SCN3A | 32 | 0.076009501 |
| GCN1 | 32 | 0.076009501 |
| DSEL | 32 | 0.076009501 |
| REV3L | 32 | 0.076009501 |
| NRXN2 | 32 | 0.076009501 |
| FLNA | 32 | 0.076009501 |
| BCOR | 32 | 0.076009501 |
| GALNT17 | 32 | 0.076009501 |
| ABCA4 | 32 | 0.076009501 |
| PKD1 | 32 | 0.076009501 |
| GOLGB1 | 32 | 0.076009501 |
| MAP1A | 32 | 0.076009501 |
| SPEN | 32 | 0.076009501 |
| MYH14 | 32 | 0.076009501 |
| BRCA2 | 32 | 0.076009501 |
| ZNF804B | 32 | 0.076009501 |
| PAPPA | 32 | 0.076009501 |
| DAPK1 | 31 | 0.073634204 |
| FAM47C | 31 | 0.073634204 |
| TIAM1 | 31 | 0.073634204 |
| SETX | 31 | 0.073634204 |
| ADCY8 | 31 | 0.073634204 |
| CHD6 | 31 | 0.073634204 |
| AKAP6 | 31 | 0.073634204 |
| SPTB | 31 | 0.073634204 |
| DMXL2 | 31 | 0.073634204 |
| NCOR2 | 31 | 0.073634204 |
| SCN2A | 31 | 0.073634204 |
| MYH9 | 31 | 0.073634204 |
| KCNB2 | 31 | 0.073634204 |
| ACACB | 31 | 0.073634204 |
| ALMS1 | 31 | 0.073634204 |
| TNRC6B | 31 | 0.073634204 |
| EPHA3 | 31 | 0.073634204 |
| SVEP1 | 31 | 0.073634204 |
| EPHA5 | 31 | 0.073634204 |
| ARFGEF1 | 31 | 0.073634204 |
| PEG3 | 31 | 0.073634204 |
| TCERG1 | 31 | 0.073634204 |
| MIDEAS | 31 | 0.073634204 |
| PCDHA8 | 31 | 0.073634204 |
| KIF26B | 31 | 0.073634204 |
| ARAP2 | 31 | 0.073634204 |
| ITPR1 | 31 | 0.073634204 |
| DLC1 | 31 | 0.073634204 |
| SCN4A | 30 | 0.071258907 |
| DISP3 | 30 | 0.071258907 |
| SLITRK1 | 30 | 0.071258907 |
| CHD9 | 30 | 0.071258907 |
| MYO5A | 30 | 0.071258907 |
| PLXNA1 | 30 | 0.071258907 |
| CPAMD8 | 30 | 0.071258907 |
| GPR179 | 30 | 0.071258907 |
| AFF2 | 30 | 0.071258907 |
| GTF3C1 | 30 | 0.071258907 |
| FASN | 30 | 0.071258907 |
| ATRX | 30 | 0.071258907 |
| TEAD2 | 30 | 0.071258907 |
| HSPG2 | 30 | 0.071258907 |
| SPHKAP | 30 | 0.071258907 |
| MYH7 | 30 | 0.071258907 |
| RIMBP2 | 30 | 0.071258907 |
| LARP4B | 30 | 0.071258907 |
| KALRN | 30 | 0.071258907 |
| SPTBN1 | 30 | 0.071258907 |
| DIDO1 | 30 | 0.071258907 |
| YLPM1 | 30 | 0.071258907 |
| ADAMTS20 | 30 | 0.071258907 |
| HELZ2 | 30 | 0.071258907 |
| CACNA1C | 30 | 0.071258907 |
| MGA | 30 | 0.071258907 |
| DCAF4L2 | 30 | 0.071258907 |
| PLXND1 | 30 | 0.071258907 |
| LAMA3 | 30 | 0.071258907 |
| PCDH18 | 29 | 0.06888361 |
| NLRC5 | 29 | 0.06888361 |
| MGAM | 29 | 0.06888361 |
| ADGRG4 | 29 | 0.06888361 |
| GPR158 | 29 | 0.06888361 |
| PKDREJ | 29 | 0.06888361 |
| AKAP13 | 29 | 0.06888361 |
| ACACA | 29 | 0.06888361 |
| CUL9 | 29 | 0.06888361 |
| MYH8 | 29 | 0.06888361 |
| FAM135B | 29 | 0.06888361 |
| SFMBT2 | 29 | 0.06888361 |
| TCF20 | 29 | 0.06888361 |
| CARD11 | 29 | 0.06888361 |
| LRRC7 | 29 | 0.06888361 |
| LPA | 29 | 0.06888361 |
| ADAMTSL3 | 29 | 0.06888361 |
| PRUNE2 | 29 | 0.06888361 |
| CTNNB1 | 29 | 0.06888361 |
| ACAN | 29 | 0.06888361 |
| MYH2 | 29 | 0.06888361 |
| RGS12 | 29 | 0.06888361 |
| ALK | 29 | 0.06888361 |
| KCNA4 | 29 | 0.06888361 |
| DLG2 | 29 | 0.06888361 |
| POLE | 29 | 0.06888361 |
| SALL1 | 29 | 0.06888361 |
| EPG5 | 29 | 0.06888361 |
| CNTNAP2 | 29 | 0.06888361 |
| ARID2 | 29 | 0.06888361 |
| UTRN | 29 | 0.06888361 |
| CDK12 | 29 | 0.06888361 |
| DMXL1 | 29 | 0.06888361 |
| PTPRZ1 | 29 | 0.06888361 |
| CACNA1G | 29 | 0.06888361 |
| USP9X | 29 | 0.06888361 |
| LYST | 29 | 0.06888361 |
| SLIT1 | 29 | 0.06888361 |
| COL14A1 | 29 | 0.06888361 |
| SIPA1L1 | 29 | 0.06888361 |
| CENPF | 29 | 0.06888361 |
| IGSF10 | 29 | 0.06888361 |
| CEP290 | 29 | 0.06888361 |
| LTBP2 | 29 | 0.06888361 |
| LAMA4 | 29 | 0.06888361 |
| NOTCH3 | 29 | 0.06888361 |
| FLNB | 29 | 0.06888361 |
| DOCK8 | 28 | 0.066508314 |
| COL4A4 | 28 | 0.066508314 |
| ITPR2 | 28 | 0.066508314 |
| PCDHB7 | 28 | 0.066508314 |
| ADAMTSL1 | 28 | 0.066508314 |
| GRM5 | 28 | 0.066508314 |
| KIAA1217 | 28 | 0.066508314 |
| TRPC4 | 28 | 0.066508314 |
| PLCG2 | 28 | 0.066508314 |
| MED12L | 28 | 0.066508314 |
| NPAP1 | 28 | 0.066508314 |
| RANBP2 | 28 | 0.066508314 |
| FLT4 | 28 | 0.066508314 |
| THSD7A | 28 | 0.066508314 |
| MTCL1 | 28 | 0.066508314 |
| NUP205 | 28 | 0.066508314 |
| USF3 | 28 | 0.066508314 |
| TCHH | 28 | 0.066508314 |
| SRRM2 | 28 | 0.066508314 |
| DOCK5 | 28 | 0.066508314 |
| TRPM3 | 28 | 0.066508314 |
| IRS4 | 28 | 0.066508314 |
| CACNA1D | 28 | 0.066508314 |
| MYO7A | 28 | 0.066508314 |
| COL4A1 | 28 | 0.066508314 |
| PKD1L1 | 28 | 0.066508314 |
| ZC3H13 | 28 | 0.066508314 |
| CIC | 28 | 0.066508314 |
| PLXNB3 | 28 | 0.066508314 |
| GREB1 | 28 | 0.066508314 |
| XYLT2 | 28 | 0.066508314 |
| MTUS2 | 28 | 0.066508314 |
| NBEAL2 | 28 | 0.066508314 |
| TRIP12 | 28 | 0.066508314 |
| NUGGC | 28 | 0.066508314 |
| CELSR2 | 28 | 0.066508314 |
| MED12 | 28 | 0.066508314 |
| POLQ | 28 | 0.066508314 |
| PLXNB1 | 28 | 0.066508314 |
| MYH13 | 28 | 0.066508314 |
| ADGRB3 | 28 | 0.066508314 |
| C3 | 27 | 0.064133017 |
| CHD3 | 27 | 0.064133017 |
| ADAMTS9 | 27 | 0.064133017 |
| RPL22 | 27 | 0.064133017 |
| NLRP7 | 27 | 0.064133017 |
| MIA3 | 27 | 0.064133017 |
| DLGAP2 | 27 | 0.064133017 |
| TULP4 | 27 | 0.064133017 |
| ZC3H18 | 27 | 0.064133017 |
| ZAN | 27 | 0.064133017 |
| COL18A1 | 27 | 0.064133017 |
| PTPN13 | 27 | 0.064133017 |
| SYCP2 | 27 | 0.064133017 |
| PCDHGA5 | 27 | 0.064133017 |
| CEP170 | 27 | 0.064133017 |
| NSD1 | 27 | 0.064133017 |
| ARID1B | 27 | 0.064133017 |
| HRNR | 27 | 0.064133017 |
| BRINP3 | 27 | 0.064133017 |
| CDH18 | 27 | 0.064133017 |
| CDH2 | 27 | 0.064133017 |
| NEXMIF | 27 | 0.064133017 |
| KCNH7 | 27 | 0.064133017 |
| ATG2A | 27 | 0.064133017 |
| COL1A2 | 27 | 0.064133017 |
| TET3 | 27 | 0.064133017 |
| CADPS | 27 | 0.064133017 |
| TENM4 | 27 | 0.064133017 |
| DOP1A | 27 | 0.064133017 |
| MYH6 | 27 | 0.064133017 |
| VPS13D | 27 | 0.064133017 |
| ADAMTS12 | 27 | 0.064133017 |
| SHANK1 | 27 | 0.064133017 |
| PCDHB2 | 27 | 0.064133017 |
| PTPRC | 27 | 0.064133017 |
| TANC2 | 27 | 0.064133017 |
| LAMC3 | 27 | 0.064133017 |
| DENND4B | 27 | 0.064133017 |
| WDFY3 | 27 | 0.064133017 |
| STAB2 | 27 | 0.064133017 |
| CLUH | 27 | 0.064133017 |
| MPDZ | 27 | 0.064133017 |
| SCAF4 | 26 | 0.06175772 |
| PHF20L1 | 26 | 0.06175772 |
| SETBP1 | 26 | 0.06175772 |
| PPL | 26 | 0.06175772 |
| GABRA5 | 26 | 0.06175772 |
| DLGAP3 | 26 | 0.06175772 |
| GRIK3 | 26 | 0.06175772 |
| FSTL5 | 26 | 0.06175772 |
| PCDHA3 | 26 | 0.06175772 |
| PCDH19 | 26 | 0.06175772 |
| CFAP65 | 26 | 0.06175772 |
| TRPM6 | 26 | 0.06175772 |
| ELOA2 | 26 | 0.06175772 |
| ZNF835 | 26 | 0.06175772 |
| ANK1 | 26 | 0.06175772 |
| ZNF318 | 26 | 0.06175772 |
| MARF1 | 26 | 0.06175772 |
| SORCS1 | 26 | 0.06175772 |
| COL5A2 | 26 | 0.06175772 |
| AXIN2 | 26 | 0.06175772 |
| GRIA2 | 26 | 0.06175772 |
| ATG2B | 26 | 0.06175772 |
| ZNF804A | 26 | 0.06175772 |
| BICRA | 26 | 0.06175772 |
| SEC16A | 26 | 0.06175772 |
| SMARCAD1 | 26 | 0.06175772 |
| MYLK | 26 | 0.06175772 |
| FILIP1 | 26 | 0.06175772 |
| KIAA1549 | 26 | 0.06175772 |
| SOGA3 | 26 | 0.06175772 |
| CHD5 | 26 | 0.06175772 |
| GRIA1 | 26 | 0.06175772 |
| NCOR1 | 26 | 0.06175772 |
| BDP1 | 26 | 0.06175772 |
| ABCA2 | 26 | 0.06175772 |
| BOD1L1 | 26 | 0.06175772 |
| FSIP2 | 26 | 0.06175772 |
| UTP20 | 26 | 0.06175772 |
| PCDHA13 | 26 | 0.06175772 |
| ZNF518A | 26 | 0.06175772 |
| TRHDE | 26 | 0.06175772 |
| CACNA1F | 26 | 0.06175772 |
| KSR2 | 26 | 0.06175772 |
| TET1 | 26 | 0.06175772 |
| TOGARAM2 | 26 | 0.06175772 |
| DSP | 26 | 0.06175772 |
| PTEN | 26 | 0.06175772 |
| CHL1 | 26 | 0.06175772 |
| CDH4 | 25 | 0.059382423 |
| HDAC9 | 25 | 0.059382423 |
| WWC3 | 25 | 0.059382423 |
| COL4A5 | 25 | 0.059382423 |
| USP35 | 25 | 0.059382423 |
| KIDINS220 | 25 | 0.059382423 |
| DCLK1 | 25 | 0.059382423 |
| TRPC3 | 25 | 0.059382423 |
| SHPRH | 25 | 0.059382423 |
| KDM6B | 25 | 0.059382423 |
| PPFIA2 | 25 | 0.059382423 |
| GRIK4 | 25 | 0.059382423 |
| MON2 | 25 | 0.059382423 |
| MAP7D1 | 25 | 0.059382423 |
| TNKS2 | 25 | 0.059382423 |
| ABCC1 | 25 | 0.059382423 |
| KIF26A | 25 | 0.059382423 |
| EIF3A | 25 | 0.059382423 |
| KATNIP | 25 | 0.059382423 |
| SMARCA4 | 25 | 0.059382423 |
| KCNB1 | 25 | 0.059382423 |
| HNRNPL | 25 | 0.059382423 |
| KCNH1 | 25 | 0.059382423 |
| PCDH7 | 25 | 0.059382423 |
| THBS2 | 25 | 0.059382423 |
| TPO | 25 | 0.059382423 |
| SLIT3 | 25 | 0.059382423 |
| RALGAPA1 | 25 | 0.059382423 |
| CD93 | 25 | 0.059382423 |
| ENAM | 25 | 0.059382423 |
| NYAP2 | 25 | 0.059382423 |
| TTK | 25 | 0.059382423 |
| AMOTL1 | 25 | 0.059382423 |
| ASTN1 | 25 | 0.059382423 |
| ASXL1 | 25 | 0.059382423 |
| EML5 | 25 | 0.059382423 |
| TNN | 25 | 0.059382423 |
| GRM7 | 25 | 0.059382423 |
| ARHGAP21 | 25 | 0.059382423 |
| NOL6 | 25 | 0.059382423 |
| STAG1 | 25 | 0.059382423 |
| TRPA1 | 25 | 0.059382423 |
| ICE1 | 25 | 0.059382423 |
| SPTBN2 | 25 | 0.059382423 |
| CASZ1 | 25 | 0.059382423 |
| CDH8 | 25 | 0.059382423 |
| ALPK2 | 25 | 0.059382423 |
| TOP2B | 25 | 0.059382423 |
| PTPN14 | 25 | 0.059382423 |
| KNL1 | 25 | 0.059382423 |
| PIK3C2G | 25 | 0.059382423 |
| GRM6 | 25 | 0.059382423 |
| SMC2 | 25 | 0.059382423 |
| ACVR1B | 25 | 0.059382423 |
| TRPM2 | 25 | 0.059382423 |
| CALD1 | 25 | 0.059382423 |
| ADCY5 | 25 | 0.059382423 |
| EPB41L3 | 25 | 0.059382423 |
| TNRC6C | 25 | 0.059382423 |
| ARHGAP31 | 25 | 0.059382423 |
| PLXNB2 | 25 | 0.059382423 |
| PCDHA11 | 25 | 0.059382423 |
| PCDHA7 | 25 | 0.059382423 |
| MECOM | 25 | 0.059382423 |
| FRMD4A | 25 | 0.059382423 |
| YTHDC2 | 25 | 0.059382423 |
| NES | 25 | 0.059382423 |
| ARHGAP35 | 25 | 0.059382423 |
| ASCC3 | 25 | 0.059382423 |
| GRM1 | 25 | 0.059382423 |
| NCAM1 | 24 | 0.057007126 |
| ADAMTS18 | 24 | 0.057007126 |
| BAZ1A | 24 | 0.057007126 |
| HCN4 | 24 | 0.057007126 |
| PCDHA1 | 24 | 0.057007126 |
| SBF1 | 24 | 0.057007126 |
| DOCK1 | 24 | 0.057007126 |
| UGGT2 | 24 | 0.057007126 |
| MUC4 | 24 | 0.057007126 |
| KIF13A | 24 | 0.057007126 |
| PLXNA2 | 24 | 0.057007126 |
| ZNF646 | 24 | 0.057007126 |
| CCDC73 | 24 | 0.057007126 |
| SLC4A3 | 24 | 0.057007126 |
| PCDHB9 | 24 | 0.057007126 |
| PLCE1 | 24 | 0.057007126 |
| DOCK10 | 24 | 0.057007126 |
| DDX60L | 24 | 0.057007126 |
| FLRT2 | 24 | 0.057007126 |
| CACHD1 | 24 | 0.057007126 |
| PDGFRA | 24 | 0.057007126 |
| ADAMTS5 | 24 | 0.057007126 |
| CDON | 24 | 0.057007126 |
| PHF2 | 24 | 0.057007126 |
| PRPF8 | 24 | 0.057007126 |
| MYH11 | 24 | 0.057007126 |
| FANCM | 24 | 0.057007126 |
| OSMR | 24 | 0.057007126 |
| DIP2A | 24 | 0.057007126 |
| ABCB1 | 24 | 0.057007126 |
| NOS3 | 24 | 0.057007126 |
| PTCH1 | 24 | 0.057007126 |
| DOCK11 | 24 | 0.057007126 |
| MCF2 | 24 | 0.057007126 |
| CAD | 24 | 0.057007126 |
| NPHS1 | 24 | 0.057007126 |
| ZNF462 | 24 | 0.057007126 |
| PLEKHA6 | 24 | 0.057007126 |
| EP300 | 24 | 0.057007126 |
| LCOR | 24 | 0.057007126 |
| KCNA5 | 24 | 0.057007126 |
| KDM2B | 24 | 0.057007126 |
| WDR17 | 24 | 0.057007126 |
| FER1L6 | 24 | 0.057007126 |
| KDR | 24 | 0.057007126 |
| CTNNA1 | 24 | 0.057007126 |
| ADCY2 | 24 | 0.057007126 |
| BAHCC1 | 24 | 0.057007126 |
| CFH | 24 | 0.057007126 |
| MSH6 | 24 | 0.057007126 |
| PTPRM | 24 | 0.057007126 |
| HTT | 24 | 0.057007126 |
| AFF3 | 24 | 0.057007126 |
| PCDHA4 | 24 | 0.057007126 |
| DIP2B | 24 | 0.057007126 |
| XIRP1 | 24 | 0.057007126 |
| SZT2 | 24 | 0.057007126 |
| CEP162 | 24 | 0.057007126 |
| CROCC | 24 | 0.057007126 |
| PIKFYVE | 24 | 0.057007126 |
| ZFYVE26 | 24 | 0.057007126 |
| NRK | 24 | 0.057007126 |
| PRDM10 | 24 | 0.057007126 |
| USP24 | 24 | 0.057007126 |
| PRR12 | 24 | 0.057007126 |
| PREX1 | 24 | 0.057007126 |
| DAAM2 | 24 | 0.057007126 |
| TMEM132B | 24 | 0.057007126 |
| ARHGAP5 | 24 | 0.057007126 |
| BIVM-ERCC5 | 24 | 0.057007126 |
| COL4A6 | 24 | 0.057007126 |
| KIF1A | 24 | 0.057007126 |
| NLGN4X | 24 | 0.057007126 |
| MYH15 | 24 | 0.057007126 |
| WNK2 | 24 | 0.057007126 |
| RGS22 | 24 | 0.057007126 |
| ABCA7 | 24 | 0.057007126 |
| SULF1 | 24 | 0.057007126 |
| HECW2 | 24 | 0.057007126 |
| PCDHGA8 | 23 | 0.054631829 |
| CCDC88A | 23 | 0.054631829 |
| BRWD1 | 23 | 0.054631829 |
| INSRR | 23 | 0.054631829 |
| PLXNA3 | 23 | 0.054631829 |
| FREM1 | 23 | 0.054631829 |
| MRC1 | 23 | 0.054631829 |
| WNK3 | 23 | 0.054631829 |
| MUC6 | 23 | 0.054631829 |
| RNF17 | 23 | 0.054631829 |
| SLIT2 | 23 | 0.054631829 |
| SAMD9 | 23 | 0.054631829 |
| SPECC1 | 23 | 0.054631829 |
| PCNX1 | 23 | 0.054631829 |
| FRMPD1 | 23 | 0.054631829 |
| USP7 | 23 | 0.054631829 |
| HIVEP2 | 23 | 0.054631829 |
| GRIK5 | 23 | 0.054631829 |
| CACNA2D3 | 23 | 0.054631829 |
| PCDHGA6 | 23 | 0.054631829 |
| RALGAPB | 23 | 0.054631829 |
| PCF11 | 23 | 0.054631829 |
| CNTN5 | 23 | 0.054631829 |
| CNTN4 | 23 | 0.054631829 |
| ATP8A2 | 23 | 0.054631829 |
| ARHGAP20 | 23 | 0.054631829 |
| CHD1 | 23 | 0.054631829 |
| CAMTA1 | 23 | 0.054631829 |
| ADCY1 | 23 | 0.054631829 |
| DPP6 | 23 | 0.054631829 |
| AKAP11 | 23 | 0.054631829 |
| PCDHA6 | 23 | 0.054631829 |
| SLITRK2 | 23 | 0.054631829 |
| CD163 | 23 | 0.054631829 |
| CAMTA2 | 23 | 0.054631829 |
| VWA8 | 23 | 0.054631829 |
| CKAP5 | 23 | 0.054631829 |
| LRRK1 | 23 | 0.054631829 |
| ARMC4 | 23 | 0.054631829 |
| MED13 | 23 | 0.054631829 |
| AHDC1 | 23 | 0.054631829 |
| NCAPD3 | 23 | 0.054631829 |
| GSE1 | 23 | 0.054631829 |
| PDS5B | 23 | 0.054631829 |
| SMCHD1 | 23 | 0.054631829 |
| SETD2 | 23 | 0.054631829 |
| NRAP | 23 | 0.054631829 |
| ATP2B3 | 23 | 0.054631829 |
| KIAA1549L | 23 | 0.054631829 |
| DOCK4 | 23 | 0.054631829 |
| HDLBP | 23 | 0.054631829 |
| ANKHD1-EIF4EBP3 | 23 | 0.054631829 |
| COL19A1 | 23 | 0.054631829 |
| IRS1 | 23 | 0.054631829 |
| TSHZ2 | 23 | 0.054631829 |
| CHRM2 | 23 | 0.054631829 |
| CDH11 | 23 | 0.054631829 |
| ABCA3 | 23 | 0.054631829 |
| SCN8A | 23 | 0.054631829 |
| DNAJC13 | 23 | 0.054631829 |
| SAMD9L | 23 | 0.054631829 |
| PCDHA5 | 23 | 0.054631829 |
| LCT | 23 | 0.054631829 |
| TLE4 | 23 | 0.054631829 |
| MDGA2 | 23 | 0.054631829 |
| CELSR3 | 23 | 0.054631829 |
| ADGRB1 | 23 | 0.054631829 |
| KCNQ5 | 23 | 0.054631829 |
| RASGRF1 | 23 | 0.054631829 |
| CNTRL | 23 | 0.054631829 |
| GLI1 | 23 | 0.054631829 |
| KCNQ3 | 23 | 0.054631829 |
| NUP210L | 23 | 0.054631829 |
| SCN10A | 23 | 0.054631829 |
| ADGRB2 | 23 | 0.054631829 |
| ESPL1 | 23 | 0.054631829 |
| NF1 | 23 | 0.054631829 |
| UNC13A | 23 | 0.054631829 |
| PCDHA9 | 23 | 0.054631829 |
| LRFN5 | 23 | 0.054631829 |
| TACC2 | 23 | 0.054631829 |
| HEATR5B | 23 | 0.054631829 |
| CNTLN | 23 | 0.054631829 |
| ATAD5 | 23 | 0.054631829 |
| A2M | 23 | 0.054631829 |
| CACNA1S | 23 | 0.054631829 |
| PPP1R3A | 23 | 0.054631829 |
| DIP2C | 23 | 0.054631829 |
| HCFC1 | 23 | 0.054631829 |
| NUP98 | 23 | 0.054631829 |
| KCNMA1 | 22 | 0.052256532 |
| TSPOAP1 | 22 | 0.052256532 |
| SEMA5B | 22 | 0.052256532 |
| MAGI2 | 22 | 0.052256532 |
| STK31 | 22 | 0.052256532 |
| FRMPD3 | 22 | 0.052256532 |
| SGSM1 | 22 | 0.052256532 |
| CTTNBP2 | 22 | 0.052256532 |
| FBXL7 | 22 | 0.052256532 |
| CDK5RAP2 | 22 | 0.052256532 |
| TEP1 | 22 | 0.052256532 |
| JMJD1C | 22 | 0.052256532 |
| PCDHGB3 | 22 | 0.052256532 |
| SORBS2 | 22 | 0.052256532 |
| ABI3BP | 22 | 0.052256532 |
| NYNRIN | 22 | 0.052256532 |
| SRGAP1 | 22 | 0.052256532 |
| LMO7 | 22 | 0.052256532 |
| CEP192 | 22 | 0.052256532 |
| ASTN2 | 22 | 0.052256532 |
| SAGE1 | 22 | 0.052256532 |
| ADGRL1 | 22 | 0.052256532 |
| EPHA6 | 22 | 0.052256532 |
| NBAS | 22 | 0.052256532 |
| EFCAB5 | 22 | 0.052256532 |
| FAM47B | 22 | 0.052256532 |
| TNRC18 | 22 | 0.052256532 |
| CRACD | 22 | 0.052256532 |
| ULK2 | 22 | 0.052256532 |
| CFAP43 | 22 | 0.052256532 |
| BOC | 22 | 0.052256532 |
| TOGARAM1 | 22 | 0.052256532 |
| AP2A2 | 22 | 0.052256532 |
| POLRMT | 22 | 0.052256532 |
| CDH12 | 22 | 0.052256532 |
| GRID1 | 22 | 0.052256532 |
| EPHB1 | 22 | 0.052256532 |
| UNC5C | 22 | 0.052256532 |
| CACNA2D1 | 22 | 0.052256532 |
| XPO4 | 22 | 0.052256532 |
| MYO7B | 22 | 0.052256532 |
| SMAD2 | 22 | 0.052256532 |
| PRDM2 | 22 | 0.052256532 |
| MYO18A | 22 | 0.052256532 |
| ATP8B2 | 22 | 0.052256532 |
| ZNF407 | 22 | 0.052256532 |
| LTBP4 | 22 | 0.052256532 |
| POSTN | 22 | 0.052256532 |
| SF3B1 | 22 | 0.052256532 |
| ADAMTS17 | 22 | 0.052256532 |
| MBD5 | 22 | 0.052256532 |
| ARHGEF17 | 22 | 0.052256532 |
| ATP6V1B1 | 22 | 0.052256532 |
| PIK3CG | 22 | 0.052256532 |
| CTNNA3 | 22 | 0.052256532 |
| LRFN2 | 22 | 0.052256532 |
| ARHGEF10 | 22 | 0.052256532 |
| TUBA3C | 22 | 0.052256532 |
| SLC8A2 | 22 | 0.052256532 |
| PRKD1 | 22 | 0.052256532 |
| ARFGEF2 | 22 | 0.052256532 |
| SNX13 | 22 | 0.052256532 |
| ADCY9 | 22 | 0.052256532 |
| DISP1 | 22 | 0.052256532 |
| SPG11 | 22 | 0.052256532 |
| MYO9A | 22 | 0.052256532 |
| NRG1 | 22 | 0.052256532 |
| COL1A1 | 22 | 0.052256532 |
| DCDC1 | 22 | 0.052256532 |
| KCNQ2 | 22 | 0.052256532 |
| TPTE | 22 | 0.052256532 |
| SCAPER | 22 | 0.052256532 |
| COL3A1 | 22 | 0.052256532 |
| KMT2E | 22 | 0.052256532 |
| OTOF | 22 | 0.052256532 |
| ROBO3 | 22 | 0.052256532 |
| POLD1 | 22 | 0.052256532 |
| ALPK3 | 22 | 0.052256532 |
| PCDHGA12 | 22 | 0.052256532 |
| MAP2K4 | 22 | 0.052256532 |
| NRIP1 | 22 | 0.052256532 |
| ARHGEF26 | 22 | 0.052256532 |
| BTBD11 | 22 | 0.052256532 |
| CUX1 | 22 | 0.052256532 |
| ITIH2 | 22 | 0.052256532 |
| SMC1B | 22 | 0.052256532 |
| SETD1A | 22 | 0.052256532 |
| PCDHGA2 | 22 | 0.052256532 |
| MYPN | 22 | 0.052256532 |
| DOCK9 | 22 | 0.052256532 |
| CDC42BPB | 22 | 0.052256532 |
| PTPRU | 22 | 0.052256532 |
| AUTS2 | 22 | 0.052256532 |
| BLM | 22 | 0.052256532 |
| SCAF11 | 22 | 0.052256532 |
| CPS1 | 22 | 0.052256532 |
| LIFR | 22 | 0.052256532 |
| PRKCG | 21 | 0.049881235 |
| OCA2 | 21 | 0.049881235 |
| WWP1 | 21 | 0.049881235 |
| CACNA1I | 21 | 0.049881235 |
| KIT | 21 | 0.049881235 |
| MYOF | 21 | 0.049881235 |
| CCDC180 | 21 | 0.049881235 |
| NLRP2 | 21 | 0.049881235 |
| PARP14 | 21 | 0.049881235 |
| TANC1 | 21 | 0.049881235 |
| HCN1 | 21 | 0.049881235 |
| RET | 21 | 0.049881235 |
| ZNF808 | 21 | 0.049881235 |
| DROSHA | 21 | 0.049881235 |
| MYT1L | 21 | 0.049881235 |
| ARAP1 | 21 | 0.049881235 |
| SALL4 | 21 | 0.049881235 |
| TMEM131 | 21 | 0.049881235 |
| CLASP1 | 21 | 0.049881235 |
| MAGEE1 | 21 | 0.049881235 |
| MYH7B | 21 | 0.049881235 |
| GLUD2 | 21 | 0.049881235 |
| NCKAP5L | 21 | 0.049881235 |
| SV2C | 21 | 0.049881235 |
| KIF1B | 21 | 0.049881235 |
| KCNA1 | 21 | 0.049881235 |
| UHRF1BP1L | 21 | 0.049881235 |
| COL2A1 | 21 | 0.049881235 |
| FAM193A | 21 | 0.049881235 |
| ZNF862 | 21 | 0.049881235 |
| RAI1 | 21 | 0.049881235 |
| PRRC2A | 21 | 0.049881235 |
| ABCB11 | 21 | 0.049881235 |
| EVC | 21 | 0.049881235 |
| PBRM1 | 21 | 0.049881235 |
| PASK | 21 | 0.049881235 |
| USP28 | 21 | 0.049881235 |
| MRTFA | 21 | 0.049881235 |
| MROH2B | 21 | 0.049881235 |
| ABCA6 | 21 | 0.049881235 |
| ERBB3 | 21 | 0.049881235 |
| ITIH6 | 21 | 0.049881235 |
| PCSK5 | 21 | 0.049881235 |
| SPATA31E1 | 21 | 0.049881235 |
| CDC42BPA | 21 | 0.049881235 |
| BRD3 | 21 | 0.049881235 |
| CFAP251 | 21 | 0.049881235 |
| ERC2 | 21 | 0.049881235 |
| TP53BP1 | 21 | 0.049881235 |
| WNK4 | 21 | 0.049881235 |
| WNK1 | 21 | 0.049881235 |
| DENND5A | 21 | 0.049881235 |
| TMPRSS15 | 21 | 0.049881235 |
| ZMYND8 | 21 | 0.049881235 |
| KDM3B | 21 | 0.049881235 |
| PAM | 21 | 0.049881235 |
| ABCA10 | 21 | 0.049881235 |
| SIGLEC1 | 21 | 0.049881235 |
| CTR9 | 21 | 0.049881235 |
| MYO9B | 21 | 0.049881235 |
| JAKMIP2 | 21 | 0.049881235 |
| DICER1 | 21 | 0.049881235 |
| CTNND2 | 21 | 0.049881235 |
| PCDHB4 | 21 | 0.049881235 |
| SRGAP3 | 21 | 0.049881235 |
| CLIP1 | 21 | 0.049881235 |
| OPLAH | 21 | 0.049881235 |
| BNC1 | 21 | 0.049881235 |
| NLRP4 | 21 | 0.049881235 |
| MYH3 | 21 | 0.049881235 |
| HLA-B | 21 | 0.049881235 |
| AR | 21 | 0.049881235 |
| PTPRD | 21 | 0.049881235 |
| SH3PXD2A | 21 | 0.049881235 |
| ARAP3 | 21 | 0.049881235 |
| RADIL | 21 | 0.049881235 |
| KIAA2026 | 21 | 0.049881235 |
| IGSF1 | 21 | 0.049881235 |
| CABIN1 | 21 | 0.049881235 |
| LRRIQ3 | 21 | 0.049881235 |
| SUPT16H | 21 | 0.049881235 |
| ZMYM4 | 21 | 0.049881235 |
| ROCK1 | 21 | 0.049881235 |
| DAB2IP | 21 | 0.049881235 |
| WDR7 | 21 | 0.049881235 |
| EYS | 21 | 0.049881235 |
| NID2 | 21 | 0.049881235 |
| GRID2 | 21 | 0.049881235 |
| PHF3 | 21 | 0.049881235 |
| CCDC39 | 21 | 0.049881235 |
| CHRD | 21 | 0.049881235 |
| FLG2 | 21 | 0.049881235 |
| APBA2 | 21 | 0.049881235 |
| KIF13B | 21 | 0.049881235 |
| TRANK1 | 21 | 0.049881235 |
| ALS2 | 21 | 0.049881235 |
| BRINP1 | 21 | 0.049881235 |
| BPTF | 21 | 0.049881235 |
| RXFP3 | 21 | 0.049881235 |
| ABCA5 | 21 | 0.049881235 |
| MAGI1 | 21 | 0.049881235 |
| ZEB2 | 21 | 0.049881235 |
| NBEAL1 | 21 | 0.049881235 |
| SMTN | 21 | 0.049881235 |
| NLRP1 | 21 | 0.049881235 |
| GPRASP1 | 21 | 0.049881235 |
| ZNF521 | 21 | 0.049881235 |
| NCAM2 | 21 | 0.049881235 |
| KIAA1755 | 21 | 0.049881235 |
| NOTCH1 | 21 | 0.049881235 |
| NUP214 | 21 | 0.049881235 |
| FAM47A | 21 | 0.049881235 |
| CIITA | 21 | 0.049881235 |
| HFM1 | 21 | 0.049881235 |
| PCDHGA7 | 21 | 0.049881235 |
| KIRREL3 | 21 | 0.049881235 |
| JCAD | 21 | 0.049881235 |
| AGRN | 21 | 0.049881235 |
| EXPH5 | 21 | 0.049881235 |
| TNIK | 21 | 0.049881235 |
| NOTCH2 | 21 | 0.049881235 |
| INPPL1 | 21 | 0.049881235 |
| SLC4A7 | 21 | 0.049881235 |
| CNTN1 | 21 | 0.049881235 |
| ZMYM2 | 21 | 0.049881235 |
| ATP1A2 | 21 | 0.049881235 |
| TLL1 | 21 | 0.049881235 |
| PRRC2C | 21 | 0.049881235 |
| CHD2 | 21 | 0.049881235 |
| PARD3B | 21 | 0.049881235 |
| DMBT1 | 21 | 0.049881235 |
| PPP6R2 | 21 | 0.049881235 |
| NINL | 21 | 0.049881235 |
| PCDHGB4 | 21 | 0.049881235 |
| MYH1 | 21 | 0.049881235 |
| NRXN3 | 21 | 0.049881235 |
| ZNFX1 | 21 | 0.049881235 |
| FPGT-TNNI3K | 21 | 0.049881235 |
| TIAM2 | 21 | 0.049881235 |
| KLHL4 | 21 | 0.049881235 |
| KLHL34 | 20 | 0.047505938 |
| MYT1 | 20 | 0.047505938 |
| MYOM2 | 20 | 0.047505938 |
| CD109 | 20 | 0.047505938 |
| ATP12A | 20 | 0.047505938 |
| ARHGAP36 | 20 | 0.047505938 |
| COL11A2 | 20 | 0.047505938 |
| GRIA4 | 20 | 0.047505938 |
| XRN1 | 20 | 0.047505938 |
| HECTD1 | 20 | 0.047505938 |
| PLA2R1 | 20 | 0.047505938 |
| DLEC1 | 20 | 0.047505938 |
| PCDHGB1 | 20 | 0.047505938 |
| KDM5A | 20 | 0.047505938 |
| MAGEL2 | 20 | 0.047505938 |
| DOP1B | 20 | 0.047505938 |
| SRCIN1 | 20 | 0.047505938 |
| SMAD3 | 20 | 0.047505938 |
| ADAM7 | 20 | 0.047505938 |
| SPART | 20 | 0.047505938 |
| ATR | 20 | 0.047505938 |
| IFI16 | 20 | 0.047505938 |
| NUMA1 | 20 | 0.047505938 |
| REST | 20 | 0.047505938 |
| ITSN2 | 20 | 0.047505938 |
| JPH3 | 20 | 0.047505938 |
| LAMB1 | 20 | 0.047505938 |
| ITGAL | 20 | 0.047505938 |
| ZNF208 | 20 | 0.047505938 |
| ARHGAP22 | 20 | 0.047505938 |
| DNM3 | 20 | 0.047505938 |
| RAD51AP2 | 20 | 0.047505938 |
| TRPC5 | 20 | 0.047505938 |
| TNRC6A | 20 | 0.047505938 |
| NCOA6 | 20 | 0.047505938 |
| GIGYF1 | 20 | 0.047505938 |
| SPATA31D1 | 20 | 0.047505938 |
| ACIN1 | 20 | 0.047505938 |
| MVK | 20 | 0.047505938 |
| KIF4B | 20 | 0.047505938 |
| N4BP2 | 20 | 0.047505938 |
| MAST2 | 20 | 0.047505938 |
| PLCH1 | 20 | 0.047505938 |
| PPRC1 | 20 | 0.047505938 |
| MYO1F | 20 | 0.047505938 |
| GRM8 | 20 | 0.047505938 |
| PCDHB12 | 20 | 0.047505938 |
| IFT172 | 20 | 0.047505938 |
| DUOX2 | 20 | 0.047505938 |
| DPP10 | 20 | 0.047505938 |
| PAX6 | 20 | 0.047505938 |
| IGSF9 | 20 | 0.047505938 |
| COL5A3 | 20 | 0.047505938 |
| ITGA7 | 20 | 0.047505938 |
| SALL2 | 20 | 0.047505938 |
| VIRMA | 20 | 0.047505938 |
| CR1 | 20 | 0.047505938 |
| PIK3C2B | 20 | 0.047505938 |
| RPS6KA2 | 20 | 0.047505938 |
| PLG | 20 | 0.047505938 |
| ST6GAL2 | 20 | 0.047505938 |
| FYB1 | 20 | 0.047505938 |
| MAP3K21 | 20 | 0.047505938 |
| LRRN3 | 20 | 0.047505938 |
| DHX30 | 20 | 0.047505938 |
| COBL | 20 | 0.047505938 |
| NOVA1 | 20 | 0.047505938 |
| PCDHB5 | 20 | 0.047505938 |
| PIGO | 20 | 0.047505938 |
| CYLC1 | 20 | 0.047505938 |
| NLRP5 | 20 | 0.047505938 |
| ZNF142 | 20 | 0.047505938 |
| CHST15 | 20 | 0.047505938 |
| ATP2A1 | 20 | 0.047505938 |
| CCDC178 | 20 | 0.047505938 |
| SEZ6L | 20 | 0.047505938 |
| MICAL3 | 20 | 0.047505938 |
| HIRA | 20 | 0.047505938 |
| CFAP47 | 20 | 0.047505938 |
| TMEM94 | 20 | 0.047505938 |
| SLC24A2 | 20 | 0.047505938 |
| CARMIL1 | 20 | 0.047505938 |
| SUPT6H | 20 | 0.047505938 |
| ADAM29 | 20 | 0.047505938 |
| SLX4 | 20 | 0.047505938 |
| LAMB4 | 20 | 0.047505938 |
| COL6A2 | 20 | 0.047505938 |
| RFX7 | 20 | 0.047505938 |
| PLPPR4 | 20 | 0.047505938 |
| PTPRB | 20 | 0.047505938 |
| MAP3K1 | 20 | 0.047505938 |
| KIF21B | 20 | 0.047505938 |
| ADAMTS10 | 20 | 0.047505938 |
| MICAL2 | 20 | 0.047505938 |
| MROH1 | 20 | 0.047505938 |
| COL4A2 | 20 | 0.047505938 |
| MLH3 | 20 | 0.047505938 |
| DNHD1 | 20 | 0.047505938 |
| ZNF609 | 20 | 0.047505938 |
| ATP13A3 | 20 | 0.047505938 |
| ANKRD12 | 20 | 0.047505938 |
| MYO10 | 20 | 0.047505938 |
| CARMIL3 | 20 | 0.047505938 |
| NRCAM | 20 | 0.047505938 |
| FGD6 | 20 | 0.047505938 |
| PDZRN4 | 20 | 0.047505938 |
| ATAD2B | 20 | 0.047505938 |
| SLITRK6 | 20 | 0.047505938 |
| SLC8A1 | 20 | 0.047505938 |
| PIK3R1 | 20 | 0.047505938 |
| CLSTN1 | 20 | 0.047505938 |
| NCOA2 | 20 | 0.047505938 |
| IQCA1 | 20 | 0.047505938 |
| ZBED9 | 20 | 0.047505938 |
| KCNH3 | 20 | 0.047505938 |
| SHANK2 | 20 | 0.047505938 |
| ATP7A | 20 | 0.047505938 |
| CAMSAP1 | 20 | 0.047505938 |
| HEATR1 | 20 | 0.047505938 |
| KCNT2 | 20 | 0.047505938 |
| SPTAN1 | 20 | 0.047505938 |
| TRIP11 | 20 | 0.047505938 |
| ABCA9 | 20 | 0.047505938 |
| TAS1R2 | 20 | 0.047505938 |
| SMARCA2 | 20 | 0.047505938 |
| ABCB4 | 20 | 0.047505938 |
| PCDHGB5 | 20 | 0.047505938 |
| SLITRK5 | 20 | 0.047505938 |
| KCNH5 | 20 | 0.047505938 |
| SLC12A5 | 20 | 0.047505938 |
| CRYBG3 | 20 | 0.047505938 |
| NRAS | 20 | 0.047505938 |
| PHIP | 20 | 0.047505938 |
| TUT7 | 20 | 0.047505938 |
| PCDH12 | 20 | 0.047505938 |
| ARFGEF3 | 20 | 0.047505938 |
| PCNX2 | 20 | 0.047505938 |
| DSPP | 20 | 0.047505938 |
| ITSN1 | 20 | 0.047505938 |
| RBM15 | 20 | 0.047505938 |
| MEGF6 | 20 | 0.047505938 |
| TPR | 20 | 0.047505938 |
| PCDHB3 | 20 | 0.047505938 |
| NAV1 | 20 | 0.047505938 |
| DCAF12L2 | 20 | 0.047505938 |
| SASH1 | 19 | 0.045130641 |
| TRPM1 | 19 | 0.045130641 |
| MDGA1 | 19 | 0.045130641 |
| ANKRD30A | 19 | 0.045130641 |
| SIN3A | 19 | 0.045130641 |
| WHRN | 19 | 0.045130641 |
| ATP2B4 | 19 | 0.045130641 |
| MROH5 | 19 | 0.045130641 |
| PRTG | 19 | 0.045130641 |
| TRERF1 | 19 | 0.045130641 |
| POU4F2 | 19 | 0.045130641 |
| APBB1 | 19 | 0.045130641 |
| C8B | 19 | 0.045130641 |
| GABRG3 | 19 | 0.045130641 |
| TRO | 19 | 0.045130641 |
| SPTBN4 | 19 | 0.045130641 |
| NTRK2 | 19 | 0.045130641 |
| ZNF43 | 19 | 0.045130641 |
| AFDN | 19 | 0.045130641 |
| NID1 | 19 | 0.045130641 |
| TNS3 | 19 | 0.045130641 |
| LRBA | 19 | 0.045130641 |
| GOLGA3 | 19 | 0.045130641 |
| BRD1 | 19 | 0.045130641 |
| BRD4 | 19 | 0.045130641 |
| ABCA8 | 19 | 0.045130641 |
| CDH6 | 19 | 0.045130641 |
| SHROOM3 | 19 | 0.045130641 |
| PSME4 | 19 | 0.045130641 |
| BRPF3 | 19 | 0.045130641 |
| SMC1A | 19 | 0.045130641 |
| DNMBP | 19 | 0.045130641 |
| ATP1A4 | 19 | 0.045130641 |
| ARHGEF40 | 19 | 0.045130641 |
| STOX2 | 19 | 0.045130641 |
| PCM1 | 19 | 0.045130641 |
| CSPG4 | 19 | 0.045130641 |
| NUP210 | 19 | 0.045130641 |
| NTRK3 | 19 | 0.045130641 |
| ZEB1 | 19 | 0.045130641 |
| AHCTF1 | 19 | 0.045130641 |
| PDS5A | 19 | 0.045130641 |
| MAST4 | 19 | 0.045130641 |
| ARHGEF11 | 19 | 0.045130641 |
| ATP10D | 19 | 0.045130641 |
| RLF | 19 | 0.045130641 |
| OR6K2 | 19 | 0.045130641 |
| NHS | 19 | 0.045130641 |
| MAST3 | 19 | 0.045130641 |
| RERE | 19 | 0.045130641 |
| FLT1 | 19 | 0.045130641 |
| STYXL2 | 19 | 0.045130641 |
| DOCK6 | 19 | 0.045130641 |
| CEP350 | 19 | 0.045130641 |
| RBP3 | 19 | 0.045130641 |
| PNISR | 19 | 0.045130641 |
| NLRP3 | 19 | 0.045130641 |
| GNAS | 19 | 0.045130641 |
| PZP | 19 | 0.045130641 |
| SLC3A2 | 19 | 0.045130641 |
| PCDHGA4 | 19 | 0.045130641 |
| ERCC6 | 19 | 0.045130641 |
| ZMYM6 | 19 | 0.045130641 |
| PITPNM3 | 19 | 0.045130641 |
| OBSL1 | 19 | 0.045130641 |
| BACH2 | 19 | 0.045130641 |
| HDAC4 | 19 | 0.045130641 |
| NPHP4 | 19 | 0.045130641 |
| C6 | 19 | 0.045130641 |
| NUAK1 | 19 | 0.045130641 |
| DHX9 | 19 | 0.045130641 |
| POLR1A | 19 | 0.045130641 |
| ITGB4 | 19 | 0.045130641 |
| PSMD1 | 19 | 0.045130641 |
| ELMO1 | 19 | 0.045130641 |
| ABCC12 | 19 | 0.045130641 |
| PPARGC1A | 19 | 0.045130641 |
| TBC1D9 | 19 | 0.045130641 |
| DPYD | 19 | 0.045130641 |
| RTTN | 19 | 0.045130641 |
| PCDHGA1 | 19 | 0.045130641 |
| CNGB1 | 19 | 0.045130641 |
| SOHLH2 | 19 | 0.045130641 |
| TLN1 | 19 | 0.045130641 |
| PRDM16 | 19 | 0.045130641 |
| ABCC5 | 19 | 0.045130641 |
| ATP8B1 | 19 | 0.045130641 |
| PPP1R26 | 19 | 0.045130641 |
| LRRCC1 | 19 | 0.045130641 |
| FKBP15 | 19 | 0.045130641 |
| FRMPD4 | 19 | 0.045130641 |
| TMPO | 19 | 0.045130641 |
| PPFIA4 | 19 | 0.045130641 |
| ZNF658 | 19 | 0.045130641 |
| RPTOR | 19 | 0.045130641 |
| SLC44A5 | 19 | 0.045130641 |
| PLEKHM1 | 19 | 0.045130641 |
| LRP4 | 19 | 0.045130641 |
| NLRP13 | 19 | 0.045130641 |
| LENG8 | 19 | 0.045130641 |
| MUC2 | 19 | 0.045130641 |
| EIF2AK4 | 19 | 0.045130641 |
| SMG1 | 19 | 0.045130641 |
| ADAM12 | 19 | 0.045130641 |
| CLSTN2 | 19 | 0.045130641 |
| PCDHB6 | 19 | 0.045130641 |
| KCNC3 | 19 | 0.045130641 |
| SBNO1 | 19 | 0.045130641 |
| GLIS3 | 19 | 0.045130641 |
| ATP2B2 | 19 | 0.045130641 |
| ARHGAP33 | 19 | 0.045130641 |
| PLCB4 | 19 | 0.045130641 |
| TUT4 | 19 | 0.045130641 |
| PTK2 | 19 | 0.045130641 |
| SP140 | 19 | 0.045130641 |
| KAT6B | 19 | 0.045130641 |
| TRIM71 | 19 | 0.045130641 |
| ITGA10 | 19 | 0.045130641 |
| KDM5B | 19 | 0.045130641 |
| EPRS1 | 19 | 0.045130641 |
| COL15A1 | 19 | 0.045130641 |
| PTPRG | 19 | 0.045130641 |
| PDGFRB | 19 | 0.045130641 |
| ADAMTS7 | 19 | 0.045130641 |
| ABCB5 | 19 | 0.045130641 |
| AFF4 | 19 | 0.045130641 |
| CPSF1 | 19 | 0.045130641 |
| PLEKHG4B | 19 | 0.045130641 |
| BTAF1 | 19 | 0.045130641 |
| TMEM131L | 19 | 0.045130641 |
| APLP1 | 19 | 0.045130641 |
| PCDHB15 | 19 | 0.045130641 |
| MCC | 19 | 0.045130641 |
| RALGAPA2 | 19 | 0.045130641 |
| NEFM | 19 | 0.045130641 |
| TAF1 | 19 | 0.045130641 |
| KNTC1 | 19 | 0.045130641 |
| GON4L | 19 | 0.045130641 |
| ZFP36L2 | 19 | 0.045130641 |
| GABBR1 | 19 | 0.045130641 |
| SI | 19 | 0.045130641 |
| ITGAX | 19 | 0.045130641 |
| PLEKHA5 | 19 | 0.045130641 |
| TSSK1B | 19 | 0.045130641 |
| SPTBN5 | 18 | 0.042755344 |
| AK9 | 18 | 0.042755344 |
| RICTOR | 18 | 0.042755344 |
| NLRP9 | 18 | 0.042755344 |
| SETDB1 | 18 | 0.042755344 |
| TMEM132E | 18 | 0.042755344 |
| GUCY1A1 | 18 | 0.042755344 |
| SV2A | 18 | 0.042755344 |
| SAFB2 | 18 | 0.042755344 |
| AGL | 18 | 0.042755344 |
| GGT5 | 18 | 0.042755344 |
| ADAMTS13 | 18 | 0.042755344 |
| CENPE | 18 | 0.042755344 |
| HIVEP3 | 18 | 0.042755344 |
| GPR149 | 18 | 0.042755344 |
| ELP1 | 18 | 0.042755344 |
| CTCF | 18 | 0.042755344 |
| AGAP1 | 18 | 0.042755344 |
| ECM2 | 18 | 0.042755344 |
| ABCC8 | 18 | 0.042755344 |
| TUBGCP3 | 18 | 0.042755344 |
| KCND2 | 18 | 0.042755344 |
| FCRL5 | 18 | 0.042755344 |
| TRPM8 | 18 | 0.042755344 |
| CNGB3 | 18 | 0.042755344 |
| SORL1 | 18 | 0.042755344 |
| PFKP | 18 | 0.042755344 |
| TGIF1 | 18 | 0.042755344 |
| ASXL3 | 18 | 0.042755344 |
| UHRF1BP1 | 18 | 0.042755344 |
| FCRL3 | 18 | 0.042755344 |
| LTBP1 | 18 | 0.042755344 |
| CD163L1 | 18 | 0.042755344 |
| PDE3A | 18 | 0.042755344 |
| MEI1 | 18 | 0.042755344 |
| KCNH8 | 18 | 0.042755344 |
| SLCO1B3 | 18 | 0.042755344 |
| SORCS3 | 18 | 0.042755344 |
| EXOC1 | 18 | 0.042755344 |
| DZIP1L | 18 | 0.042755344 |
| TBC1D25 | 18 | 0.042755344 |
| PCDHGB7 | 18 | 0.042755344 |
| KCND3 | 18 | 0.042755344 |
| COL20A1 | 18 | 0.042755344 |
| SEMA3C | 18 | 0.042755344 |
| PHRF1 | 18 | 0.042755344 |
| RUNX1T1 | 18 | 0.042755344 |
| B2M | 18 | 0.042755344 |
| LRRTM4 | 18 | 0.042755344 |
| COL24A1 | 18 | 0.042755344 |
| RNF40 | 18 | 0.042755344 |
| MYOCD | 18 | 0.042755344 |
| KIAA0319L | 18 | 0.042755344 |
| NEURL4 | 18 | 0.042755344 |
| TNKS | 18 | 0.042755344 |
| MAP4K4 | 18 | 0.042755344 |
| CRMP1 | 18 | 0.042755344 |
| ROR2 | 18 | 0.042755344 |
| ASXL2 | 18 | 0.042755344 |
| ATRNL1 | 18 | 0.042755344 |
| CEP85L | 18 | 0.042755344 |
| HSPA2 | 18 | 0.042755344 |
| ANKRD26 | 18 | 0.042755344 |
| PPP4R4 | 18 | 0.042755344 |
| TSHZ1 | 18 | 0.042755344 |
| UNC45B | 18 | 0.042755344 |
| ZNF516 | 18 | 0.042755344 |
| MAP1S | 18 | 0.042755344 |
| MCM3AP | 18 | 0.042755344 |
| KANSL1L | 18 | 0.042755344 |
| CSF3R | 18 | 0.042755344 |
| C2orf16 | 18 | 0.042755344 |
| DYNC2I1 | 18 | 0.042755344 |
| WSCD2 | 18 | 0.042755344 |
| LZTR1 | 18 | 0.042755344 |
| BTBD7 | 18 | 0.042755344 |
| VRTN | 18 | 0.042755344 |
| KCNV2 | 18 | 0.042755344 |
| CNNM1 | 18 | 0.042755344 |
| KIAA0586 | 18 | 0.042755344 |
| COL6A1 | 18 | 0.042755344 |
| PCNX3 | 18 | 0.042755344 |
| ABCC4 | 18 | 0.042755344 |
| NOD2 | 18 | 0.042755344 |
| ZNF540 | 18 | 0.042755344 |
| AL592490.1 | 18 | 0.042755344 |
| OVCH1 | 18 | 0.042755344 |
| ARHGEF1 | 18 | 0.042755344 |
| ATAD2 | 18 | 0.042755344 |
| B4GALNT1 | 18 | 0.042755344 |
| JARID2 | 18 | 0.042755344 |
| ZNF236 | 18 | 0.042755344 |
| TFAP2D | 18 | 0.042755344 |
| MPRIP | 18 | 0.042755344 |
| NOS2 | 18 | 0.042755344 |
| PER3 | 18 | 0.042755344 |
| F5 | 18 | 0.042755344 |
| NPAT | 18 | 0.042755344 |
| RAPGEF2 | 18 | 0.042755344 |
| MGAT3 | 18 | 0.042755344 |
| NCKAP1L | 18 | 0.042755344 |
| DDX58 | 18 | 0.042755344 |
| SCAF8 | 18 | 0.042755344 |
| SH3BP4 | 18 | 0.042755344 |
| TTC3 | 18 | 0.042755344 |
| JHY | 18 | 0.042755344 |
| DDX17 | 18 | 0.042755344 |
| SNTG1 | 18 | 0.042755344 |
| SEMA3D | 18 | 0.042755344 |
| RASGRF2 | 18 | 0.042755344 |
| ITIH5 | 18 | 0.042755344 |
| DCTN1 | 18 | 0.042755344 |
| PRRC2B | 18 | 0.042755344 |
| C5 | 18 | 0.042755344 |
| MADD | 18 | 0.042755344 |
| ACE | 18 | 0.042755344 |
| ZZEF1 | 18 | 0.042755344 |
| PSD2 | 18 | 0.042755344 |
| PCDHGA3 | 18 | 0.042755344 |
| JADE1 | 18 | 0.042755344 |
| CCDC141 | 18 | 0.042755344 |
| TIE1 | 18 | 0.042755344 |
| PCDHGB6 | 18 | 0.042755344 |
| CAMSAP2 | 18 | 0.042755344 |
| LINGO2 | 18 | 0.042755344 |
| CAND1 | 18 | 0.042755344 |
| FANCA | 18 | 0.042755344 |
| ITGAD | 18 | 0.042755344 |
| HELZ | 18 | 0.042755344 |
| KIF2B | 18 | 0.042755344 |
| PLXNC1 | 18 | 0.042755344 |
| EPHA4 | 18 | 0.042755344 |
| SIPA1L2 | 18 | 0.042755344 |
| SCAF1 | 18 | 0.042755344 |
| ANKRD50 | 18 | 0.042755344 |
| ZBTB7C | 18 | 0.042755344 |
| SLCO6A1 | 18 | 0.042755344 |
| PCDH8 | 18 | 0.042755344 |
| INTS1 | 18 | 0.042755344 |
| PCDHB1 | 18 | 0.042755344 |
| TRPC6 | 18 | 0.042755344 |
| PHLPP1 | 18 | 0.042755344 |
| EIF4G3 | 18 | 0.042755344 |
| TDRD6 | 18 | 0.042755344 |
| CHRM3 | 18 | 0.042755344 |
| CEMIP | 18 | 0.042755344 |
| CDC14A | 18 | 0.042755344 |
| MIA2 | 18 | 0.042755344 |
| MYOM1 | 18 | 0.042755344 |
| MMP9 | 18 | 0.042755344 |
| TRDN | 18 | 0.042755344 |
| GPATCH8 | 18 | 0.042755344 |
| ATXN2L | 18 | 0.042755344 |
| TEK | 18 | 0.042755344 |
| ATP9A | 18 | 0.042755344 |
| ZNF98 | 18 | 0.042755344 |
| QSER1 | 18 | 0.042755344 |
| RBM10 | 18 | 0.042755344 |
| CRYBG1 | 18 | 0.042755344 |
| PTPRN2 | 18 | 0.042755344 |
| NLRP8 | 18 | 0.042755344 |
| SYCP2L | 18 | 0.042755344 |
| WASF3 | 18 | 0.042755344 |
| CASP8 | 18 | 0.042755344 |
| SETD5 | 18 | 0.042755344 |
| CASKIN2 | 18 | 0.042755344 |
| UBE4B | 18 | 0.042755344 |
| TEX14 | 18 | 0.042755344 |
| MAPK8IP3 | 18 | 0.042755344 |
| TRAPPC9 | 18 | 0.042755344 |
| IMPG1 | 18 | 0.042755344 |
| PHLDB2 | 18 | 0.042755344 |
| MAGEC3 | 18 | 0.042755344 |
| FAM214A | 18 | 0.042755344 |
| ARHGAP28 | 18 | 0.042755344 |
| TSC1 | 18 | 0.042755344 |
| SHROOM4 | 18 | 0.042755344 |
| EFEMP1 | 18 | 0.042755344 |
| CKAP2 | 18 | 0.042755344 |
| UNC80 | 18 | 0.042755344 |
| SRRM4 | 18 | 0.042755344 |
| ZBTB40 | 18 | 0.042755344 |
| RPGRIP1L | 18 | 0.042755344 |
| CLCN4 | 18 | 0.042755344 |
| SNRNP200 | 18 | 0.042755344 |
| ZNF91 | 18 | 0.042755344 |
| TLN2 | 17 | 0.040380048 |
| EPB41L5 | 17 | 0.040380048 |
| PLEKHH2 | 17 | 0.040380048 |
| ZGRF1 | 17 | 0.040380048 |
| ESRP1 | 17 | 0.040380048 |
| BCHE | 17 | 0.040380048 |
| PAX3 | 17 | 0.040380048 |
| ASAP2 | 17 | 0.040380048 |
| NELL2 | 17 | 0.040380048 |
| PIP5K1C | 17 | 0.040380048 |
| STARD8 | 17 | 0.040380048 |
| UPF2 | 17 | 0.040380048 |
| WDTC1 | 17 | 0.040380048 |
| MSH2 | 17 | 0.040380048 |
| NEDD9 | 17 | 0.040380048 |
| ARHGAP30 | 17 | 0.040380048 |
| PAPLN | 17 | 0.040380048 |
| PTCHD3 | 17 | 0.040380048 |
| ATP11C | 17 | 0.040380048 |
| PI4KA | 17 | 0.040380048 |
| SGO2 | 17 | 0.040380048 |
| KAT6A | 17 | 0.040380048 |
| ABL2 | 17 | 0.040380048 |
| RFX5 | 17 | 0.040380048 |
| CLEC16A | 17 | 0.040380048 |
| RTL9 | 17 | 0.040380048 |
| GIGYF2 | 17 | 0.040380048 |
| USP19 | 17 | 0.040380048 |
| FCSK | 17 | 0.040380048 |
| GABRG1 | 17 | 0.040380048 |
| ERICH6 | 17 | 0.040380048 |
| LHCGR | 17 | 0.040380048 |
| FSTL4 | 17 | 0.040380048 |
| GRIN2B | 17 | 0.040380048 |
| IGSF3 | 17 | 0.040380048 |
| BEND3 | 17 | 0.040380048 |
| MAP3K5 | 17 | 0.040380048 |
| ARPP21 | 17 | 0.040380048 |
| PPP1R9A | 17 | 0.040380048 |
| JAK1 | 17 | 0.040380048 |
| CPED1 | 17 | 0.040380048 |
| NAALAD2 | 17 | 0.040380048 |
| HIVEP1 | 17 | 0.040380048 |
| CCDC158 | 17 | 0.040380048 |
| PTBP2 | 17 | 0.040380048 |
| ZNF626 | 17 | 0.040380048 |
| SMC4 | 17 | 0.040380048 |
| ATP13A5 | 17 | 0.040380048 |
| BAZ2B | 17 | 0.040380048 |
| INF2 | 17 | 0.040380048 |
| RFC1 | 17 | 0.040380048 |
| HDX | 17 | 0.040380048 |
| ARHGEF15 | 17 | 0.040380048 |
| SLC26A7 | 17 | 0.040380048 |
| SLC12A7 | 17 | 0.040380048 |
| EIF2AK1 | 17 | 0.040380048 |
| ZHX2 | 17 | 0.040380048 |
| MAP3K13 | 17 | 0.040380048 |
| PCDHGA11 | 17 | 0.040380048 |
| TBX3 | 17 | 0.040380048 |
| HEG1 | 17 | 0.040380048 |
| TNKS1BP1 | 17 | 0.040380048 |
| MTTP | 17 | 0.040380048 |
| CASP8AP2 | 17 | 0.040380048 |
| STAT1 | 17 | 0.040380048 |
| CCNA1 | 17 | 0.040380048 |
| SYNPO2 | 17 | 0.040380048 |
| GUF1 | 17 | 0.040380048 |
| PAXIP1 | 17 | 0.040380048 |
| FAM214B | 17 | 0.040380048 |
| NKTR | 17 | 0.040380048 |
| CTNND1 | 17 | 0.040380048 |
| CEP164 | 17 | 0.040380048 |
| ERN2 | 17 | 0.040380048 |
| ADAMTS4 | 17 | 0.040380048 |
| LRRC41 | 17 | 0.040380048 |
| KIAA0100 | 17 | 0.040380048 |
| PIWIL1 | 17 | 0.040380048 |
| RGS3 | 17 | 0.040380048 |
| SAFB | 17 | 0.040380048 |
| NEDD4 | 17 | 0.040380048 |
| HPSE2 | 17 | 0.040380048 |
| MCHR2 | 17 | 0.040380048 |
| INTS2 | 17 | 0.040380048 |
| DDI1 | 17 | 0.040380048 |
| RNF6 | 17 | 0.040380048 |
| LRP5 | 17 | 0.040380048 |
| SOX5 | 17 | 0.040380048 |
| DGKD | 17 | 0.040380048 |
| USP6 | 17 | 0.040380048 |
| SBNO2 | 17 | 0.040380048 |
| HEATR5A | 17 | 0.040380048 |
| FASTKD3 | 17 | 0.040380048 |
| DIS3L | 17 | 0.040380048 |
| DNER | 17 | 0.040380048 |
| HIF3A | 17 | 0.040380048 |
| ZDHHC8 | 17 | 0.040380048 |
| E2F7 | 17 | 0.040380048 |
| DENND2C | 17 | 0.040380048 |
| IQGAP3 | 17 | 0.040380048 |
| SH3TC1 | 17 | 0.040380048 |
| MASP1 | 17 | 0.040380048 |
| RBM33 | 17 | 0.040380048 |
| GRM3 | 17 | 0.040380048 |
| ATG9B | 17 | 0.040380048 |
| CENPJ | 17 | 0.040380048 |
| PELP1 | 17 | 0.040380048 |
| CLCA4 | 17 | 0.040380048 |
| XKR4 | 17 | 0.040380048 |
| PLCG1 | 17 | 0.040380048 |
| ADAMTS1 | 17 | 0.040380048 |
| ARMC3 | 17 | 0.040380048 |
| PCARE | 17 | 0.040380048 |
| TICRR | 17 | 0.040380048 |
| COL17A1 | 17 | 0.040380048 |
| FZD10 | 17 | 0.040380048 |
| TTBK1 | 17 | 0.040380048 |
| AC011462.1 | 17 | 0.040380048 |
| FGD1 | 17 | 0.040380048 |
| SFSWAP | 17 | 0.040380048 |
| FXR1 | 17 | 0.040380048 |
| GEMIN5 | 17 | 0.040380048 |
| CEP250 | 17 | 0.040380048 |
| HIP1R | 17 | 0.040380048 |
| THBS1 | 17 | 0.040380048 |
| TRIM9 | 17 | 0.040380048 |
| ATP8B3 | 17 | 0.040380048 |
| MAP10 | 17 | 0.040380048 |
| IQGAP2 | 17 | 0.040380048 |
| ATP4A | 17 | 0.040380048 |
| FHOD1 | 17 | 0.040380048 |
| SLC12A6 | 17 | 0.040380048 |
| GOLGA4 | 17 | 0.040380048 |
| TBX18 | 17 | 0.040380048 |
| USP29 | 17 | 0.040380048 |
| PLIN4 | 17 | 0.040380048 |
| SYNJ2 | 17 | 0.040380048 |
| ZNF334 | 17 | 0.040380048 |
| PEAR1 | 17 | 0.040380048 |
| NWD1 | 17 | 0.040380048 |
| NAP1L3 | 17 | 0.040380048 |
| EPS8L1 | 17 | 0.040380048 |
| NPAS4 | 17 | 0.040380048 |
| NCAPG | 17 | 0.040380048 |
| MYBPC3 | 17 | 0.040380048 |
| WDR33 | 17 | 0.040380048 |
| RNF111 | 17 | 0.040380048 |
| FBXO10 | 17 | 0.040380048 |
| FAM83B | 17 | 0.040380048 |
| FMNL3 | 17 | 0.040380048 |
| EGFLAM | 17 | 0.040380048 |
| NRG3 | 17 | 0.040380048 |
| NCOA5 | 17 | 0.040380048 |
| EPHA2 | 17 | 0.040380048 |
| CCDC110 | 17 | 0.040380048 |
| USP48 | 17 | 0.040380048 |
| IRX1 | 17 | 0.040380048 |
| HTR2C | 17 | 0.040380048 |
| TRAPPC8 | 17 | 0.040380048 |
| C10orf90 | 17 | 0.040380048 |
| TAOK2 | 17 | 0.040380048 |
| ZBTB41 | 17 | 0.040380048 |
| OTUD4 | 17 | 0.040380048 |
| PTPRF | 17 | 0.040380048 |
| ATP11A | 17 | 0.040380048 |
| ANKIB1 | 17 | 0.040380048 |
| ECPAS | 17 | 0.040380048 |
| CECR2 | 17 | 0.040380048 |
| NR4A2 | 17 | 0.040380048 |
| CFTR | 17 | 0.040380048 |
| RXFP2 | 17 | 0.040380048 |
| SLFN13 | 17 | 0.040380048 |
| CFAP54 | 17 | 0.040380048 |
| ZNF629 | 17 | 0.040380048 |
| CRB1 | 17 | 0.040380048 |
| EGR2 | 17 | 0.040380048 |
| EDNRB | 17 | 0.040380048 |
| LVRN | 17 | 0.040380048 |
| CEP170B | 17 | 0.040380048 |
| TGFBR2 | 17 | 0.040380048 |
| PIK3R6 | 17 | 0.040380048 |
| CDC42BPG | 17 | 0.040380048 |
| FLCN | 17 | 0.040380048 |
| NFATC1 | 17 | 0.040380048 |
| SNAP91 | 17 | 0.040380048 |
| SRFBP1 | 17 | 0.040380048 |
| RPRD2 | 17 | 0.040380048 |
| CCDC80 | 17 | 0.040380048 |
| SHANK3 | 17 | 0.040380048 |
| TRIM46 | 17 | 0.040380048 |
| SLC9C1 | 17 | 0.040380048 |
| LAMB2 | 17 | 0.040380048 |
| CAMSAP3 | 17 | 0.040380048 |
| PLCL2 | 17 | 0.040380048 |
| ZBBX | 17 | 0.040380048 |
| NDST3 | 17 | 0.040380048 |
| COLEC12 | 17 | 0.040380048 |
| SIGLEC8 | 17 | 0.040380048 |
| ST18 | 17 | 0.040380048 |
| ZNF644 | 17 | 0.040380048 |
| ATP10B | 17 | 0.040380048 |
| CADPS2 | 17 | 0.040380048 |
| EDC4 | 17 | 0.040380048 |
| CNTNAP1 | 17 | 0.040380048 |
| STAT5B | 17 | 0.040380048 |
| EIF4G1 | 17 | 0.040380048 |
| DENND5B | 17 | 0.040380048 |
| HSPA12A | 17 | 0.040380048 |
| TASOR2 | 17 | 0.040380048 |
| MYH4 | 17 | 0.040380048 |
| ZSCAN20 | 17 | 0.040380048 |
| PCDHB10 | 17 | 0.040380048 |
| INHBA | 17 | 0.040380048 |
| DHX38 | 17 | 0.040380048 |
| ANO4 | 17 | 0.040380048 |
| NIN | 17 | 0.040380048 |
| ZNF106 | 16 | 0.038004751 |
| CDH1 | 16 | 0.038004751 |
| FILIP1L | 16 | 0.038004751 |
| PRDM5 | 16 | 0.038004751 |
| SYNGAP1 | 16 | 0.038004751 |
| HSPA1L | 16 | 0.038004751 |
| DAB1 | 16 | 0.038004751 |
| EXOC6 | 16 | 0.038004751 |
| SLFN5 | 16 | 0.038004751 |
| KLHL6 | 16 | 0.038004751 |
| INPP4A | 16 | 0.038004751 |
| EXT1 | 16 | 0.038004751 |
| TBC1D9B | 16 | 0.038004751 |
| IARS2 | 16 | 0.038004751 |
| SCUBE3 | 16 | 0.038004751 |
| MXRA8 | 16 | 0.038004751 |
| MAP7D3 | 16 | 0.038004751 |
| SNED1 | 16 | 0.038004751 |
| ITGB2 | 16 | 0.038004751 |
| SLC4A1 | 16 | 0.038004751 |
| PCDHB13 | 16 | 0.038004751 |
| CWF19L2 | 16 | 0.038004751 |
| MCF2L2 | 16 | 0.038004751 |
| DDX27 | 16 | 0.038004751 |
| LRGUK | 16 | 0.038004751 |
| SLC12A1 | 16 | 0.038004751 |
| JAKMIP1 | 16 | 0.038004751 |
| MAP3K12 | 16 | 0.038004751 |
| PLK1 | 16 | 0.038004751 |
| RBM46 | 16 | 0.038004751 |
| NFE2L1 | 16 | 0.038004751 |
| CHAT | 16 | 0.038004751 |
| ZFR2 | 16 | 0.038004751 |
| ZNF418 | 16 | 0.038004751 |
| ARAF | 16 | 0.038004751 |
| DHX15 | 16 | 0.038004751 |
| FIGN | 16 | 0.038004751 |
| VAV3 | 16 | 0.038004751 |
| CR2 | 16 | 0.038004751 |
| MED13L | 16 | 0.038004751 |
| CADM1 | 16 | 0.038004751 |
| ZNF385D | 16 | 0.038004751 |
| INPP5D | 16 | 0.038004751 |
| EVPL | 16 | 0.038004751 |
| OPCML | 16 | 0.038004751 |
| DNMT3B | 16 | 0.038004751 |
| SLC6A2 | 16 | 0.038004751 |
| IFT140 | 16 | 0.038004751 |
| PCDHA10 | 16 | 0.038004751 |
| MDC1 | 16 | 0.038004751 |
| GRIP2 | 16 | 0.038004751 |
| LUZP1 | 16 | 0.038004751 |
| OAS2 | 16 | 0.038004751 |
| GCC2 | 16 | 0.038004751 |
| OR2M4 | 16 | 0.038004751 |
| CRTC1 | 16 | 0.038004751 |
| CUL1 | 16 | 0.038004751 |
| IBTK | 16 | 0.038004751 |
| FOLH1 | 16 | 0.038004751 |
| SLC4A10 | 16 | 0.038004751 |
| RBBP6 | 16 | 0.038004751 |
| RTN1 | 16 | 0.038004751 |
| AP3D1 | 16 | 0.038004751 |
| ZNF99 | 16 | 0.038004751 |
| SRRM1 | 16 | 0.038004751 |
| KAT14 | 16 | 0.038004751 |
| SLC39A10 | 16 | 0.038004751 |
| MMS22L | 16 | 0.038004751 |
| RAPGEF6 | 16 | 0.038004751 |
| PEAK1 | 16 | 0.038004751 |
| GATA3 | 16 | 0.038004751 |
| ENC1 | 16 | 0.038004751 |
| KLHL1 | 16 | 0.038004751 |
| TJP2 | 16 | 0.038004751 |
| CLTCL1 | 16 | 0.038004751 |
| MYBBP1A | 16 | 0.038004751 |
| PRDM15 | 16 | 0.038004751 |
| SOS2 | 16 | 0.038004751 |
| GANAB | 16 | 0.038004751 |
| ZNF615 | 16 | 0.038004751 |
| LGR4 | 16 | 0.038004751 |
| TRIM24 | 16 | 0.038004751 |
| IQSEC2 | 16 | 0.038004751 |
| LEPR | 16 | 0.038004751 |
| NFATC4 | 16 | 0.038004751 |
| LIPE | 16 | 0.038004751 |
| KIAA0319 | 16 | 0.038004751 |
| IQSEC1 | 16 | 0.038004751 |
| CIT | 16 | 0.038004751 |
| CCDC88C | 16 | 0.038004751 |
| EOMES | 16 | 0.038004751 |
| SLC32A1 | 16 | 0.038004751 |
| DHX34 | 16 | 0.038004751 |
| NUP133 | 16 | 0.038004751 |
| EEF1AKMT4-ECE2 | 16 | 0.038004751 |
| RESF1 | 16 | 0.038004751 |
| SLC1A6 | 16 | 0.038004751 |
| MYO3B | 16 | 0.038004751 |
| OGDH | 16 | 0.038004751 |
| SEMA6A | 16 | 0.038004751 |
| NMUR2 | 16 | 0.038004751 |
| SPON1 | 16 | 0.038004751 |
| CFAP69 | 16 | 0.038004751 |
| ATP2A2 | 16 | 0.038004751 |
| MINK1 | 16 | 0.038004751 |
| BCL11A | 16 | 0.038004751 |
| KCNU1 | 16 | 0.038004751 |
| BRIP1 | 16 | 0.038004751 |
| KCTD8 | 16 | 0.038004751 |
| RFTN1 | 16 | 0.038004751 |
| GUCY1A2 | 16 | 0.038004751 |
| NCOA1 | 16 | 0.038004751 |
| CCAR1 | 16 | 0.038004751 |
| AXL | 16 | 0.038004751 |
| TRPC7 | 16 | 0.038004751 |
| PYHIN1 | 16 | 0.038004751 |
| TMTC2 | 16 | 0.038004751 |
| TBXT | 16 | 0.038004751 |
| TRPV6 | 16 | 0.038004751 |
| GAA | 16 | 0.038004751 |
| TRIM37 | 16 | 0.038004751 |
| LARGE1 | 16 | 0.038004751 |
| SUPT5H | 16 | 0.038004751 |
| DENND4A | 16 | 0.038004751 |
| TPP2 | 16 | 0.038004751 |
| ITGA8 | 16 | 0.038004751 |
| WASHC2C | 16 | 0.038004751 |
| DUOX1 | 16 | 0.038004751 |
| SYCP1 | 16 | 0.038004751 |
| FBH1 | 16 | 0.038004751 |
| MN1 | 16 | 0.038004751 |
| ANKS1B | 16 | 0.038004751 |
| MAP2K7 | 16 | 0.038004751 |
| P3H3 | 16 | 0.038004751 |
| ADAMTSL4 | 16 | 0.038004751 |
| PDE4DIP | 16 | 0.038004751 |
| CLCN2 | 16 | 0.038004751 |
| TRPV5 | 16 | 0.038004751 |
| MYO5C | 16 | 0.038004751 |
| IPO11 | 16 | 0.038004751 |
| HBS1L | 16 | 0.038004751 |
| GJA8 | 16 | 0.038004751 |
| HEPHL1 | 16 | 0.038004751 |
| KAZN | 16 | 0.038004751 |
| CILP | 16 | 0.038004751 |
| ZFAT | 16 | 0.038004751 |
| WIZ | 16 | 0.038004751 |
| LRP6 | 16 | 0.038004751 |
| ANKRD17 | 16 | 0.038004751 |
| CFAP61 | 16 | 0.038004751 |
| PRAM1 | 16 | 0.038004751 |
| SEMA5A | 16 | 0.038004751 |
| FAN1 | 16 | 0.038004751 |
| ITGAM | 16 | 0.038004751 |
| VPS50 | 16 | 0.038004751 |
| ZFC3H1 | 16 | 0.038004751 |
| NOL4 | 16 | 0.038004751 |
| ENTPD4 | 16 | 0.038004751 |
| SHOC1 | 16 | 0.038004751 |
| PATJ | 16 | 0.038004751 |
| TIMELESS | 16 | 0.038004751 |
| BICC1 | 16 | 0.038004751 |
| TAOK3 | 16 | 0.038004751 |
| PLCB3 | 16 | 0.038004751 |
| PTPRN | 16 | 0.038004751 |
| MGAT5B | 16 | 0.038004751 |
| CDHR3 | 16 | 0.038004751 |
| MAP3K10 | 16 | 0.038004751 |
| WDR49 | 16 | 0.038004751 |
| WDR90 | 16 | 0.038004751 |
| DNMT1 | 16 | 0.038004751 |
| KNDC1 | 16 | 0.038004751 |
| FHDC1 | 16 | 0.038004751 |
| GIMAP8 | 16 | 0.038004751 |
| CC2D2A | 16 | 0.038004751 |
| FKBP9 | 16 | 0.038004751 |
| PTGER3 | 16 | 0.038004751 |
| PAK5 | 16 | 0.038004751 |
| SEC31A | 16 | 0.038004751 |
| IRAG1 | 16 | 0.038004751 |
| TXLNB | 16 | 0.038004751 |
| SIM1 | 16 | 0.038004751 |
| FOXK1 | 16 | 0.038004751 |
| SMARCA1 | 16 | 0.038004751 |
| BCLAF1 | 16 | 0.038004751 |
| CORIN | 16 | 0.038004751 |
| LRP12 | 16 | 0.038004751 |
| TTF1 | 16 | 0.038004751 |
| MORC1 | 16 | 0.038004751 |
| CNKSR2 | 16 | 0.038004751 |
| DIAPH3 | 16 | 0.038004751 |
| BRSK1 | 16 | 0.038004751 |
| IL16 | 16 | 0.038004751 |
| TRIM3 | 16 | 0.038004751 |
| SOS1 | 16 | 0.038004751 |
| TTC37 | 16 | 0.038004751 |
| LRRN1 | 16 | 0.038004751 |
| PCBP1 | 16 | 0.038004751 |
| REV1 | 16 | 0.038004751 |
| DGKB | 16 | 0.038004751 |
| PCDHAC1 | 16 | 0.038004751 |
| CACNA2D4 | 16 | 0.038004751 |
| COL9A1 | 16 | 0.038004751 |
| TBC1D32 | 16 | 0.038004751 |
| RASAL3 | 16 | 0.038004751 |
| DOCK7 | 16 | 0.038004751 |
| ROBO4 | 16 | 0.038004751 |
| RAB11FIP1 | 16 | 0.038004751 |
| ADGRD1 | 16 | 0.038004751 |
| CNKSR1 | 16 | 0.038004751 |
| ACTA1 | 16 | 0.038004751 |
| CCT8L2 | 16 | 0.038004751 |
| ULK1 | 16 | 0.038004751 |
| AMPH | 16 | 0.038004751 |
| MLH1 | 16 | 0.038004751 |
| HTR1A | 16 | 0.038004751 |
| HK3 | 16 | 0.038004751 |
| GRIN3A | 16 | 0.038004751 |
| CGNL1 | 16 | 0.038004751 |
| GBF1 | 16 | 0.038004751 |
| CBX8 | 16 | 0.038004751 |
| NELL1 | 16 | 0.038004751 |
| MYH10 | 16 | 0.038004751 |
| MGAT4C | 16 | 0.038004751 |
| DHX29 | 16 | 0.038004751 |
| SLC4A11 | 16 | 0.038004751 |
| DIAPH2 | 16 | 0.038004751 |
| KIF4A | 16 | 0.038004751 |
| G3BP1 | 16 | 0.038004751 |
| KCTD19 | 16 | 0.038004751 |
| DDX60 | 16 | 0.038004751 |
| JAG2 | 16 | 0.038004751 |
| CDH16 | 16 | 0.038004751 |
| ADGRG6 | 16 | 0.038004751 |
| CCDC28A | 16 | 0.038004751 |
| CASR | 16 | 0.038004751 |
| LIG3 | 16 | 0.038004751 |
| PHC1 | 16 | 0.038004751 |
| IFT80 | 16 | 0.038004751 |
| ITGA11 | 16 | 0.038004751 |
| SPAG9 | 16 | 0.038004751 |
| MAST1 | 16 | 0.038004751 |
| SLC9C2 | 16 | 0.038004751 |
| TRPM7 | 16 | 0.038004751 |
| KLHL5 | 16 | 0.038004751 |
| MST1R | 16 | 0.038004751 |
| MET | 16 | 0.038004751 |
| EPHA7 | 16 | 0.038004751 |
| TMEM168 | 16 | 0.038004751 |
| CENPC | 16 | 0.038004751 |
| SPPL2B | 16 | 0.038004751 |
| POP1 | 16 | 0.038004751 |
| NAALADL1 | 16 | 0.038004751 |
| CYFIP2 | 16 | 0.038004751 |
| ZFYVE28 | 16 | 0.038004751 |
| NLRC4 | 15 | 0.035629454 |
| PSD | 15 | 0.035629454 |
| DDX54 | 15 | 0.035629454 |
| SON | 15 | 0.035629454 |
| CD1E | 15 | 0.035629454 |
| PC | 15 | 0.035629454 |
| PLCB1 | 15 | 0.035629454 |
| KCNJ3 | 15 | 0.035629454 |
| SCRIB | 15 | 0.035629454 |
| ADCY10 | 15 | 0.035629454 |
| KANK1 | 15 | 0.035629454 |
| ARHGEF18 | 15 | 0.035629454 |
| TICAM1 | 15 | 0.035629454 |
| ISLR2 | 15 | 0.035629454 |
| ITIH1 | 15 | 0.035629454 |
| SLC6A15 | 15 | 0.035629454 |
| ITGA5 | 15 | 0.035629454 |
| AEBP1 | 15 | 0.035629454 |
| SPINK5 | 15 | 0.035629454 |
| RAD54L2 | 15 | 0.035629454 |
| RASGRP3 | 15 | 0.035629454 |
| ZNF619 | 15 | 0.035629454 |
| SLC27A6 | 15 | 0.035629454 |
| ITGA4 | 15 | 0.035629454 |
| RASA1 | 15 | 0.035629454 |
| ZNF471 | 15 | 0.035629454 |
| IL1RL1 | 15 | 0.035629454 |
| ZIC4 | 15 | 0.035629454 |
| KIF20B | 15 | 0.035629454 |
| SLC16A6 | 15 | 0.035629454 |
| FURIN | 15 | 0.035629454 |
| ANO5 | 15 | 0.035629454 |
| GAK | 15 | 0.035629454 |
| B4GALNT4 | 15 | 0.035629454 |
| USP26 | 15 | 0.035629454 |
| ZIM2 | 15 | 0.035629454 |
| MARK4 | 15 | 0.035629454 |
| NPC1 | 15 | 0.035629454 |
| MACC1 | 15 | 0.035629454 |
| CDRT1 | 15 | 0.035629454 |
| DLGAP1 | 15 | 0.035629454 |
| CYP7B1 | 15 | 0.035629454 |
| AGO3 | 15 | 0.035629454 |
| ARHGEF6 | 15 | 0.035629454 |
| TAP2 | 15 | 0.035629454 |
| SLITRK3 | 15 | 0.035629454 |
| FANCD2 | 15 | 0.035629454 |
| CEP152 | 15 | 0.035629454 |
| PAXBP1 | 15 | 0.035629454 |
| HCFC2 | 15 | 0.035629454 |
| JUP | 15 | 0.035629454 |
| AARS2 | 15 | 0.035629454 |
| ADAR | 15 | 0.035629454 |
| RELCH | 15 | 0.035629454 |
| SH3PXD2B | 15 | 0.035629454 |
| AASS | 15 | 0.035629454 |
| NEMF | 15 | 0.035629454 |
| C15orf39 | 15 | 0.035629454 |
| RSF1 | 15 | 0.035629454 |
| EPPK1 | 15 | 0.035629454 |
| IL18RAP | 15 | 0.035629454 |
| RTN4 | 15 | 0.035629454 |
| AXDND1 | 15 | 0.035629454 |
| IMPG2 | 15 | 0.035629454 |
| HHIP | 15 | 0.035629454 |
| CASS4 | 15 | 0.035629454 |
| XDH | 15 | 0.035629454 |
| ZNF793 | 15 | 0.035629454 |
| CNTN3 | 15 | 0.035629454 |
| MAN2A1 | 15 | 0.035629454 |
| DDR2 | 15 | 0.035629454 |
| RNF123 | 15 | 0.035629454 |
| USP43 | 15 | 0.035629454 |
| UBE3A | 15 | 0.035629454 |
| SLC9A3 | 15 | 0.035629454 |
| SNX29 | 15 | 0.035629454 |
| IL1RAPL1 | 15 | 0.035629454 |
| ZNF7 | 15 | 0.035629454 |
| ZFP28 | 15 | 0.035629454 |
| PRKCB | 15 | 0.035629454 |
| BMP1 | 15 | 0.035629454 |
| IL7R | 15 | 0.035629454 |
| SLCO5A1 | 15 | 0.035629454 |
| KDM2A | 15 | 0.035629454 |
| CHST2 | 15 | 0.035629454 |
| GLDC | 15 | 0.035629454 |
| POTEE | 15 | 0.035629454 |
| MIS18BP1 | 15 | 0.035629454 |
| MCM4 | 15 | 0.035629454 |
| WRN | 15 | 0.035629454 |
| EEF1AKNMT | 15 | 0.035629454 |
| ARID4A | 15 | 0.035629454 |
| PDHA2 | 15 | 0.035629454 |
| SRGAP2 | 15 | 0.035629454 |
| GABRQ | 15 | 0.035629454 |
| HLA-A | 15 | 0.035629454 |
| ABR | 15 | 0.035629454 |
| AGO4 | 15 | 0.035629454 |
| USP44 | 15 | 0.035629454 |
| MARK2 | 15 | 0.035629454 |
| NPC1L1 | 15 | 0.035629454 |
| ACTL9 | 15 | 0.035629454 |
| PTK2B | 15 | 0.035629454 |
| MPO | 15 | 0.035629454 |
| CC2D1A | 15 | 0.035629454 |
| FYCO1 | 15 | 0.035629454 |
| NAA15 | 15 | 0.035629454 |
| TLR7 | 15 | 0.035629454 |
| ZNF451 | 15 | 0.035629454 |
| ARHGAP32 | 15 | 0.035629454 |
| ZC3H4 | 15 | 0.035629454 |
| TNPO1 | 15 | 0.035629454 |
| CDH19 | 15 | 0.035629454 |
| ITGAV | 15 | 0.035629454 |
| TUBGCP2 | 15 | 0.035629454 |
| FOXG1 | 15 | 0.035629454 |
| LNPEP | 15 | 0.035629454 |
| CHSY1 | 15 | 0.035629454 |
| IFFO1 | 15 | 0.035629454 |
| EIF2AK3 | 15 | 0.035629454 |
| CEP120 | 15 | 0.035629454 |
| LONRF2 | 15 | 0.035629454 |
| ALPK1 | 15 | 0.035629454 |
| TTC21A | 15 | 0.035629454 |
| EMSY | 15 | 0.035629454 |
| PTGFRN | 15 | 0.035629454 |
| NTNG2 | 15 | 0.035629454 |
| UNC13B | 15 | 0.035629454 |
| UBN1 | 15 | 0.035629454 |
| WASL | 15 | 0.035629454 |
| NR0B1 | 15 | 0.035629454 |
| CASP5 | 15 | 0.035629454 |
| PDE6A | 15 | 0.035629454 |
| CARD6 | 15 | 0.035629454 |
| SYNRG | 15 | 0.035629454 |
| CATSPERE | 15 | 0.035629454 |
| AASDH | 15 | 0.035629454 |
| DNAI4 | 15 | 0.035629454 |
| FNIP1 | 15 | 0.035629454 |
| CEP112 | 15 | 0.035629454 |
| GRIK1 | 15 | 0.035629454 |
| RABGAP1 | 15 | 0.035629454 |
| IGF2BP1 | 15 | 0.035629454 |
| SPAG5 | 15 | 0.035629454 |
| CASKIN1 | 15 | 0.035629454 |
| MED1 | 15 | 0.035629454 |
| MGAT5 | 15 | 0.035629454 |
| FLRT1 | 15 | 0.035629454 |
| KCNJ8 | 15 | 0.035629454 |
| ESR1 | 15 | 0.035629454 |
| SSH2 | 15 | 0.035629454 |
| SPRTN | 15 | 0.035629454 |
| ZNF366 | 15 | 0.035629454 |
| DHX57 | 15 | 0.035629454 |
| PTPN23 | 15 | 0.035629454 |
| AMBRA1 | 15 | 0.035629454 |
| MTA2 | 15 | 0.035629454 |
| MEIS1 | 15 | 0.035629454 |
| ANO3 | 15 | 0.035629454 |
| ZNF638 | 15 | 0.035629454 |
| ZNF616 | 15 | 0.035629454 |
| ATP6V0A4 | 15 | 0.035629454 |
| MAMLD1 | 15 | 0.035629454 |
| MARK1 | 15 | 0.035629454 |
| ABL1 | 15 | 0.035629454 |
| ZNF585A | 15 | 0.035629454 |
| SGO1 | 15 | 0.035629454 |
| INSR | 15 | 0.035629454 |
| ADGRG2 | 15 | 0.035629454 |
| AC004922.1 | 15 | 0.035629454 |
| SOX6 | 15 | 0.035629454 |
| ANKAR | 15 | 0.035629454 |
| EFCAB6 | 15 | 0.035629454 |
| DGKI | 15 | 0.035629454 |
| BICDL1 | 15 | 0.035629454 |
| UPF1 | 15 | 0.035629454 |
| KCNA3 | 15 | 0.035629454 |
| IPO8 | 15 | 0.035629454 |
| EHD3 | 15 | 0.035629454 |
| ZNF512B | 15 | 0.035629454 |
| VPS33A | 15 | 0.035629454 |
| PTCHD4 | 15 | 0.035629454 |
| CSDE1 | 15 | 0.035629454 |
| PCDHGC5 | 15 | 0.035629454 |
| GPR37 | 15 | 0.035629454 |
| PLEKHG1 | 15 | 0.035629454 |
| TRIM42 | 15 | 0.035629454 |
| ZC3H6 | 15 | 0.035629454 |
| GUCY1B1 | 15 | 0.035629454 |
| FER | 15 | 0.035629454 |
| BRINP2 | 15 | 0.035629454 |
| PCDHAC2 | 15 | 0.035629454 |
| ZDHHC7 | 15 | 0.035629454 |
| VCAM1 | 15 | 0.035629454 |
| EMILIN1 | 15 | 0.035629454 |
| PGR | 15 | 0.035629454 |
| LINGO1 | 15 | 0.035629454 |
| DENND6A | 15 | 0.035629454 |
| SYMPK | 15 | 0.035629454 |
| AGTPBP1 | 15 | 0.035629454 |
| ARHGEF10L | 15 | 0.035629454 |
| NEK10 | 15 | 0.035629454 |
| LOXL2 | 15 | 0.035629454 |
| MYO1E | 15 | 0.035629454 |
| CTC1 | 15 | 0.035629454 |
| WDR35 | 15 | 0.035629454 |
| RBL2 | 15 | 0.035629454 |
| ADAMTS3 | 15 | 0.035629454 |
| CFAP58 | 15 | 0.035629454 |
| SELP | 15 | 0.035629454 |
| EVI5 | 15 | 0.035629454 |
| IVL | 15 | 0.035629454 |
| FGFR2 | 15 | 0.035629454 |
| MARCHF10 | 15 | 0.035629454 |
| C6orf118 | 15 | 0.035629454 |
| TNK2 | 15 | 0.035629454 |
| CLIP2 | 15 | 0.035629454 |
| WDR3 | 15 | 0.035629454 |
| PROKR1 | 15 | 0.035629454 |
| CCKBR | 15 | 0.035629454 |
| ZFR | 15 | 0.035629454 |
| RIMBP3 | 15 | 0.035629454 |
| SLC12A9 | 15 | 0.035629454 |
| GFRA1 | 15 | 0.035629454 |
| GDF5 | 15 | 0.035629454 |
| FBXW10 | 15 | 0.035629454 |
| CYLC2 | 15 | 0.035629454 |
| RB1CC1 | 15 | 0.035629454 |
| XKR6 | 15 | 0.035629454 |
| ZNF416 | 15 | 0.035629454 |
| SEL1L3 | 15 | 0.035629454 |
| MCTP1 | 15 | 0.035629454 |
| CFAP45 | 15 | 0.035629454 |
| AGBL5 | 15 | 0.035629454 |
| RFX6 | 15 | 0.035629454 |
| KIF17 | 15 | 0.035629454 |
| ADD3 | 15 | 0.035629454 |
| STRIP2 | 15 | 0.035629454 |
| IL1RAPL2 | 15 | 0.035629454 |
| ADAM8 | 15 | 0.035629454 |
| HELQ | 15 | 0.035629454 |
| PCDHB14 | 15 | 0.035629454 |
| COPA | 15 | 0.035629454 |
| SLC24A3 | 15 | 0.035629454 |
| MYO1A | 15 | 0.035629454 |
| MAP9 | 15 | 0.035629454 |
| PARD3 | 15 | 0.035629454 |
| RAPH1 | 15 | 0.035629454 |
| TNS2 | 15 | 0.035629454 |
| SND1 | 15 | 0.035629454 |
| ZSWIM8 | 15 | 0.035629454 |
| RAG1 | 15 | 0.035629454 |
| KCNG1 | 15 | 0.035629454 |
| DLG5 | 15 | 0.035629454 |
| HGF | 15 | 0.035629454 |
| CFAP100 | 15 | 0.035629454 |
| PDZD4 | 15 | 0.035629454 |
| OR6N1 | 15 | 0.035629454 |
| DEPDC5 | 15 | 0.035629454 |
| KCNH4 | 15 | 0.035629454 |
| SLC18A3 | 15 | 0.035629454 |
| ERF | 15 | 0.035629454 |
| GAPVD1 | 15 | 0.035629454 |
| C10orf120 | 15 | 0.035629454 |
| RBFOX1 | 15 | 0.035629454 |
| ZFYVE16 | 15 | 0.035629454 |
| PRG4 | 15 | 0.035629454 |
| ITGB8 | 15 | 0.035629454 |
| SYDE2 | 15 | 0.035629454 |
| KPRP | 15 | 0.035629454 |
| VPS8 | 15 | 0.035629454 |
| TOP3A | 15 | 0.035629454 |
| PHLDB1 | 15 | 0.035629454 |
| STON1 | 15 | 0.035629454 |
| C2CD5 | 15 | 0.035629454 |
| TEX10 | 15 | 0.035629454 |
| DZIP1 | 15 | 0.035629454 |
| NUP155 | 15 | 0.035629454 |
| NAA25 | 15 | 0.035629454 |
| TTC21B | 15 | 0.035629454 |
| CDH22 | 15 | 0.035629454 |
| MSH3 | 15 | 0.035629454 |
| HELB | 15 | 0.035629454 |
| KCTD3 | 15 | 0.035629454 |
| CPQ | 15 | 0.035629454 |
| DHX16 | 15 | 0.035629454 |
| THOC2 | 15 | 0.035629454 |
| SYNPO2L | 15 | 0.035629454 |
| GUCY2C | 15 | 0.035629454 |
| C1R | 15 | 0.035629454 |
| TMF1 | 15 | 0.035629454 |
| ATP1A1 | 15 | 0.035629454 |
| TTLL2 | 15 | 0.035629454 |
| DIAPH1 | 15 | 0.035629454 |
| CCDC171 | 15 | 0.035629454 |
| SLITRK4 | 15 | 0.035629454 |
| KCNS2 | 15 | 0.035629454 |
| MME | 15 | 0.035629454 |
| ALS2CL | 15 | 0.035629454 |
| UBN2 | 15 | 0.035629454 |
| LRRC66 | 15 | 0.035629454 |
| KIAA0825 | 15 | 0.035629454 |
| ZNF677 | 15 | 0.035629454 |
| NSD2 | 15 | 0.035629454 |
| RAPGEF1 | 15 | 0.035629454 |
| ADGRL4 | 15 | 0.035629454 |
| NDST4 | 15 | 0.035629454 |
| ABCC9 | 15 | 0.035629454 |
| ACVR2B | 14 | 0.033254157 |
| TRAK1 | 14 | 0.033254157 |
| CSF1R | 14 | 0.033254157 |
| LDB3 | 14 | 0.033254157 |
| HIPK2 | 14 | 0.033254157 |
| TIMP3 | 14 | 0.033254157 |
| ANKRD52 | 14 | 0.033254157 |
| NLGN3 | 14 | 0.033254157 |
| EFHB | 14 | 0.033254157 |
| ATRN | 14 | 0.033254157 |
| PRAMEF17 | 14 | 0.033254157 |
| MYO1B | 14 | 0.033254157 |
| PCSK7 | 14 | 0.033254157 |
| GRIA3 | 14 | 0.033254157 |
| STAG3 | 14 | 0.033254157 |
| ITK | 14 | 0.033254157 |
| CATSPERD | 14 | 0.033254157 |
| MON1B | 14 | 0.033254157 |
| PLA2G6 | 14 | 0.033254157 |
| NLRC3 | 14 | 0.033254157 |
| KIAA1210 | 14 | 0.033254157 |
| SEC61A1 | 14 | 0.033254157 |
| ASNSD1 | 14 | 0.033254157 |
| OTOA | 14 | 0.033254157 |
| LZTS3 | 14 | 0.033254157 |
| OLFML2B | 14 | 0.033254157 |
| DCX | 14 | 0.033254157 |
| UROC1 | 14 | 0.033254157 |
| TOP2A | 14 | 0.033254157 |
| KDM4B | 14 | 0.033254157 |
| POLM | 14 | 0.033254157 |
| NPR2 | 14 | 0.033254157 |
| ATP8B4 | 14 | 0.033254157 |
| UNC5B | 14 | 0.033254157 |
| ADGRA3 | 14 | 0.033254157 |
| DVL2 | 14 | 0.033254157 |
| EHBP1 | 14 | 0.033254157 |
| SYNPO | 14 | 0.033254157 |
| NOL8 | 14 | 0.033254157 |
| NLRP14 | 14 | 0.033254157 |
| COL16A1 | 14 | 0.033254157 |
| MEX3B | 14 | 0.033254157 |
| RBM27 | 14 | 0.033254157 |
| TDRD3 | 14 | 0.033254157 |
| PRPF40B | 14 | 0.033254157 |
| TRIM45 | 14 | 0.033254157 |
| CLASP2 | 14 | 0.033254157 |
| PSD4 | 14 | 0.033254157 |
| POM121L12 | 14 | 0.033254157 |
| ADCY6 | 14 | 0.033254157 |
| POGZ | 14 | 0.033254157 |
| EPHB6 | 14 | 0.033254157 |
| PDE8B | 14 | 0.033254157 |
| RUSC2 | 14 | 0.033254157 |
| CNTN2 | 14 | 0.033254157 |
| CTSC | 14 | 0.033254157 |
| ZMYM1 | 14 | 0.033254157 |
| EXD3 | 14 | 0.033254157 |
| NEO1 | 14 | 0.033254157 |
| PHACTR1 | 14 | 0.033254157 |
| HNF1A | 14 | 0.033254157 |
| PTCHD1 | 14 | 0.033254157 |
| POLR2B | 14 | 0.033254157 |
| UGT3A2 | 14 | 0.033254157 |
| PFAS | 14 | 0.033254157 |
| SLCO2A1 | 14 | 0.033254157 |
| PNPLA8 | 14 | 0.033254157 |
| GLB1L2 | 14 | 0.033254157 |
| EFCAB13 | 14 | 0.033254157 |
| EPX | 14 | 0.033254157 |
| CEP126 | 14 | 0.033254157 |
| PKN1 | 14 | 0.033254157 |
| PRPF6 | 14 | 0.033254157 |
| SALL3 | 14 | 0.033254157 |
| SPATA31A1 | 14 | 0.033254157 |
| SNX25 | 14 | 0.033254157 |
| PKD2L1 | 14 | 0.033254157 |
| RAD50 | 14 | 0.033254157 |
| ANKRD35 | 14 | 0.033254157 |
| RPGRIP1 | 14 | 0.033254157 |
| CLTC | 14 | 0.033254157 |
| CDAN1 | 14 | 0.033254157 |
| UNC5D | 14 | 0.033254157 |
| DIS3L2 | 14 | 0.033254157 |
| AMPD2 | 14 | 0.033254157 |
| OGDHL | 14 | 0.033254157 |
| EML1 | 14 | 0.033254157 |
| FNDC3B | 14 | 0.033254157 |
| WNT9A | 14 | 0.033254157 |
| SIGLEC10 | 14 | 0.033254157 |
| CCDC9B | 14 | 0.033254157 |
| OR2M2 | 14 | 0.033254157 |
| PHKA2 | 14 | 0.033254157 |
| ZNF184 | 14 | 0.033254157 |
| GUCY2F | 14 | 0.033254157 |
| ETV1 | 14 | 0.033254157 |
| NOM1 | 14 | 0.033254157 |
| SAP130 | 14 | 0.033254157 |
| PKD2 | 14 | 0.033254157 |
| ATP2A3 | 14 | 0.033254157 |
| CPZ | 14 | 0.033254157 |
| PODN | 14 | 0.033254157 |
| FNBP4 | 14 | 0.033254157 |
| VARS1 | 14 | 0.033254157 |
| AMER2 | 14 | 0.033254157 |
| FGD5 | 14 | 0.033254157 |
| CASD1 | 14 | 0.033254157 |
| LYN | 14 | 0.033254157 |
| MCF2L | 14 | 0.033254157 |
| KIF16B | 14 | 0.033254157 |
| PITPNM1 | 14 | 0.033254157 |
| RASGRP2 | 14 | 0.033254157 |
| KSR1 | 14 | 0.033254157 |
| GLI2 | 14 | 0.033254157 |
| BUB1 | 14 | 0.033254157 |
| USP15 | 14 | 0.033254157 |
| MLLT6 | 14 | 0.033254157 |
| FOXN3 | 14 | 0.033254157 |
| ACSS2 | 14 | 0.033254157 |
| MMP16 | 14 | 0.033254157 |
| ZNF443 | 14 | 0.033254157 |
| ZNF557 | 14 | 0.033254157 |
| EGR1 | 14 | 0.033254157 |
| ROCK2 | 14 | 0.033254157 |
| MYO1G | 14 | 0.033254157 |
| CCDC15 | 14 | 0.033254157 |
| FER1L5 | 14 | 0.033254157 |
| MRGPRX1 | 14 | 0.033254157 |
| CNBD1 | 14 | 0.033254157 |
| SLCO3A1 | 14 | 0.033254157 |
| MBD1 | 14 | 0.033254157 |
| E4F1 | 14 | 0.033254157 |
| PLCZ1 | 14 | 0.033254157 |
| DRD5 | 14 | 0.033254157 |
| ABCC10 | 14 | 0.033254157 |
| LATS1 | 14 | 0.033254157 |
| VWA7 | 14 | 0.033254157 |
| FAM184A | 14 | 0.033254157 |
| VEPH1 | 14 | 0.033254157 |
| XPO6 | 14 | 0.033254157 |
| EHD2 | 14 | 0.033254157 |
| GLRB | 14 | 0.033254157 |
| MAP3K9 | 14 | 0.033254157 |
| RBM25 | 14 | 0.033254157 |
| DNAI3 | 14 | 0.033254157 |
| STON1-GTF2A1L | 14 | 0.033254157 |
| EEA1 | 14 | 0.033254157 |
| MAD1L1 | 14 | 0.033254157 |
| PYGB | 14 | 0.033254157 |
| SORCS2 | 14 | 0.033254157 |
| ABCC11 | 14 | 0.033254157 |
| ENPP2 | 14 | 0.033254157 |
| ADNP | 14 | 0.033254157 |
| MAPRE3 | 14 | 0.033254157 |
| EPHA10 | 14 | 0.033254157 |
| PLEKHH1 | 14 | 0.033254157 |
| TSC22D1 | 14 | 0.033254157 |
| CTNNBL1 | 14 | 0.033254157 |
| IQSEC3 | 14 | 0.033254157 |
| GABRB2 | 14 | 0.033254157 |
| DCAF1 | 14 | 0.033254157 |
| ADAM21 | 14 | 0.033254157 |
| GRM2 | 14 | 0.033254157 |
| OR1C1 | 14 | 0.033254157 |
| ANO2 | 14 | 0.033254157 |
| FAM83H | 14 | 0.033254157 |
| LARS1 | 14 | 0.033254157 |
| GP2 | 14 | 0.033254157 |
| EBF1 | 14 | 0.033254157 |
| R3HDM1 | 14 | 0.033254157 |
| ETV6 | 14 | 0.033254157 |
| PCDH11Y | 14 | 0.033254157 |
| NOTCH4 | 14 | 0.033254157 |
| MASTL | 14 | 0.033254157 |
| SHROOM2 | 14 | 0.033254157 |
| HEATR4 | 14 | 0.033254157 |
| HDAC5 | 14 | 0.033254157 |
| PCDHGB2 | 14 | 0.033254157 |
| APBA1 | 14 | 0.033254157 |
| PHC2 | 14 | 0.033254157 |
| FAM151A | 14 | 0.033254157 |
| ZXDA | 14 | 0.033254157 |
| LAMC1 | 14 | 0.033254157 |
| FGF13 | 14 | 0.033254157 |
| DCAF12L1 | 14 | 0.033254157 |
| CAND2 | 14 | 0.033254157 |
| MAPKBP1 | 14 | 0.033254157 |
| STAMBPL1 | 14 | 0.033254157 |
| IL18R1 | 14 | 0.033254157 |
| CEP78 | 14 | 0.033254157 |
| STS | 14 | 0.033254157 |
| RGS20 | 14 | 0.033254157 |
| QRICH2 | 14 | 0.033254157 |
| MITF | 14 | 0.033254157 |
| ANKRD27 | 14 | 0.033254157 |
| DUS4L-BCAP29 | 14 | 0.033254157 |
| MEFV | 14 | 0.033254157 |
| EIF4ENIF1 | 14 | 0.033254157 |
| PTPN3 | 14 | 0.033254157 |
| PPP1R13B | 14 | 0.033254157 |
| TUB | 14 | 0.033254157 |
| TAF3 | 14 | 0.033254157 |
| SEMA3A | 14 | 0.033254157 |
| APOBR | 14 | 0.033254157 |
| FCHO2 | 14 | 0.033254157 |
| F13A1 | 14 | 0.033254157 |
| PUS7 | 14 | 0.033254157 |
| LHX1 | 14 | 0.033254157 |
| C3orf20 | 14 | 0.033254157 |
| ZMYM3 | 14 | 0.033254157 |
| AP3B1 | 14 | 0.033254157 |
| SMC5 | 14 | 0.033254157 |
| GBP1 | 14 | 0.033254157 |
| CEP128 | 14 | 0.033254157 |
| DGKZ | 14 | 0.033254157 |
| NLRP11 | 14 | 0.033254157 |
| USP31 | 14 | 0.033254157 |
| PRRG1 | 14 | 0.033254157 |
| NAA16 | 14 | 0.033254157 |
| WDR62 | 14 | 0.033254157 |
| BRPF1 | 14 | 0.033254157 |
| LRRC49 | 14 | 0.033254157 |
| SENP7 | 14 | 0.033254157 |
| SLC16A1 | 14 | 0.033254157 |
| BAZ2A | 14 | 0.033254157 |
| KBTBD6 | 14 | 0.033254157 |
| PRX | 14 | 0.033254157 |
| KIRREL2 | 14 | 0.033254157 |
| CLSPN | 14 | 0.033254157 |
| MRC2 | 14 | 0.033254157 |
| UBA6 | 14 | 0.033254157 |
| ABCC3 | 14 | 0.033254157 |
| WDR47 | 14 | 0.033254157 |
| TOPBP1 | 14 | 0.033254157 |
| SLC4A5 | 14 | 0.033254157 |
| ZNF836 | 14 | 0.033254157 |
| MARS1 | 14 | 0.033254157 |
| SLC27A1 | 14 | 0.033254157 |
| ZBTB46 | 14 | 0.033254157 |
| NPAS3 | 14 | 0.033254157 |
| BCAS3 | 14 | 0.033254157 |
| ALOX5 | 14 | 0.033254157 |
| CATSPERB | 14 | 0.033254157 |
| CCDC116 | 14 | 0.033254157 |
| PCDHGA10 | 14 | 0.033254157 |
| DDX42 | 14 | 0.033254157 |
| ZRANB3 | 14 | 0.033254157 |
| OTUD7B | 14 | 0.033254157 |
| WDR19 | 14 | 0.033254157 |
| NFRKB | 14 | 0.033254157 |
| WNT16 | 14 | 0.033254157 |
| ZHX3 | 14 | 0.033254157 |
| PTPRH | 14 | 0.033254157 |
| LY75 | 14 | 0.033254157 |
| ADAMTSL2 | 14 | 0.033254157 |
| ZNF335 | 14 | 0.033254157 |
| ZNF304 | 14 | 0.033254157 |
| NCAPD2 | 14 | 0.033254157 |
| TGM4 | 14 | 0.033254157 |
| LRP10 | 14 | 0.033254157 |
| MAP4 | 14 | 0.033254157 |
| TSGA10 | 14 | 0.033254157 |
| OR5T2 | 14 | 0.033254157 |
| ZNF607 | 14 | 0.033254157 |
| STXBP5L | 14 | 0.033254157 |
| POLR2A | 14 | 0.033254157 |
| DYNC1I1 | 14 | 0.033254157 |
| NOL4L | 14 | 0.033254157 |
| POTEF | 14 | 0.033254157 |
| SDHA | 14 | 0.033254157 |
| DHX8 | 14 | 0.033254157 |
| MKRN3 | 14 | 0.033254157 |
| GABRA1 | 14 | 0.033254157 |
| ZBED4 | 14 | 0.033254157 |
| FAM83E | 14 | 0.033254157 |
| MOV10L1 | 14 | 0.033254157 |
| SEMG2 | 14 | 0.033254157 |
| TUBGCP6 | 14 | 0.033254157 |
| MELTF | 14 | 0.033254157 |
| KLKB1 | 14 | 0.033254157 |
| ADARB1 | 14 | 0.033254157 |
| URB2 | 14 | 0.033254157 |
| NEDD4L | 14 | 0.033254157 |
| ZNF483 | 14 | 0.033254157 |
| KTN1 | 14 | 0.033254157 |
| DCAF5 | 14 | 0.033254157 |
| EPB41L1 | 14 | 0.033254157 |
| FAM135A | 14 | 0.033254157 |
| TUBGCP5 | 14 | 0.033254157 |
| TNPO3 | 14 | 0.033254157 |
| WDR72 | 14 | 0.033254157 |
| LOXL3 | 14 | 0.033254157 |
| CLIP4 | 14 | 0.033254157 |
| HTR7 | 14 | 0.033254157 |
| NOMO1 | 14 | 0.033254157 |
| TJP1 | 14 | 0.033254157 |
| GALNT13 | 14 | 0.033254157 |
| PLEKHM2 | 14 | 0.033254157 |
| ALDH1A3 | 14 | 0.033254157 |
| SLC5A2 | 14 | 0.033254157 |
| PRAG1 | 14 | 0.033254157 |
| RTKN2 | 14 | 0.033254157 |
| LMTK3 | 14 | 0.033254157 |
| COL28A1 | 14 | 0.033254157 |
| TTLL7 | 14 | 0.033254157 |
| CPD | 14 | 0.033254157 |
| NRDC | 14 | 0.033254157 |
| MAN2C1 | 14 | 0.033254157 |
| JRK | 14 | 0.033254157 |
| CNTNAP3 | 14 | 0.033254157 |
| ZNF592 | 14 | 0.033254157 |
| NCOA3 | 14 | 0.033254157 |
| MTMR4 | 14 | 0.033254157 |
| TBC1D10B | 14 | 0.033254157 |
| SLC26A5 | 14 | 0.033254157 |
| UBE3C | 14 | 0.033254157 |
| RORB | 14 | 0.033254157 |
| SNAPC4 | 14 | 0.033254157 |
| HKDC1 | 14 | 0.033254157 |
| PDE1C | 14 | 0.033254157 |
| RNF20 | 14 | 0.033254157 |
| ZNF479 | 14 | 0.033254157 |
| TOPORS | 14 | 0.033254157 |
| CFAP44 | 14 | 0.033254157 |
| GMEB2 | 14 | 0.033254157 |
| LOXL4 | 14 | 0.033254157 |
| IGDCC4 | 14 | 0.033254157 |
| ZC3H12C | 14 | 0.033254157 |
| SEL1L2 | 14 | 0.033254157 |
| DENND3 | 14 | 0.033254157 |
| LRIG1 | 14 | 0.033254157 |
| PROS1 | 14 | 0.033254157 |
| ZNF354C | 14 | 0.033254157 |
| ANPEP | 14 | 0.033254157 |
| YTHDC1 | 14 | 0.033254157 |
| LNX1 | 14 | 0.033254157 |
| PCSK1 | 14 | 0.033254157 |
| ITGA6 | 14 | 0.033254157 |
| GTF2IRD1 | 14 | 0.033254157 |
| LRRTM1 | 14 | 0.033254157 |
| ZBTB33 | 14 | 0.033254157 |
| ANXA6 | 14 | 0.033254157 |
| KCNN3 | 14 | 0.033254157 |
| KIF5B | 14 | 0.033254157 |
| AP5Z1 | 14 | 0.033254157 |
| GCNA | 14 | 0.033254157 |
| PRPF4B | 14 | 0.033254157 |
| FYB2 | 14 | 0.033254157 |
| SPOCK3 | 14 | 0.033254157 |
| RASA2 | 14 | 0.033254157 |
| CSNK1A1L | 14 | 0.033254157 |
| COL21A1 | 14 | 0.033254157 |
| AMPD1 | 14 | 0.033254157 |
| TBX5 | 14 | 0.033254157 |
| UBR1 | 14 | 0.033254157 |
| KDM6A | 14 | 0.033254157 |
| RRBP1 | 14 | 0.033254157 |
| IGF1R | 14 | 0.033254157 |
| VPS41 | 14 | 0.033254157 |
| KIAA0232 | 14 | 0.033254157 |
| TVP23A | 14 | 0.033254157 |
| XPR1 | 14 | 0.033254157 |
| NFATC2 | 14 | 0.033254157 |
| PLEKHG2 | 14 | 0.033254157 |
| TCF4 | 14 | 0.033254157 |
| C2CD3 | 14 | 0.033254157 |
| OSBPL6 | 14 | 0.033254157 |
| SIPA1L3 | 14 | 0.033254157 |
| EXOC4 | 14 | 0.033254157 |
| CELF4 | 14 | 0.033254157 |
| NISCH | 14 | 0.033254157 |
| F9 | 14 | 0.033254157 |
| CD58 | 14 | 0.033254157 |
| XAB2 | 14 | 0.033254157 |
| ZBED1 | 13 | 0.03087886 |
| ZNF492 | 13 | 0.03087886 |
| LTN1 | 13 | 0.03087886 |
| ZNF528 | 13 | 0.03087886 |
| FZD2 | 13 | 0.03087886 |
| EBF3 | 13 | 0.03087886 |
| MTA1 | 13 | 0.03087886 |
| CPXCR1 | 13 | 0.03087886 |
| MED23 | 13 | 0.03087886 |
| ITGA3 | 13 | 0.03087886 |
| NSD3 | 13 | 0.03087886 |
| CDH13 | 13 | 0.03087886 |
| GABRA4 | 13 | 0.03087886 |
| YEATS2 | 13 | 0.03087886 |
| MIER3 | 13 | 0.03087886 |
| ALG13 | 13 | 0.03087886 |
| DTX4 | 13 | 0.03087886 |
| ADNP2 | 13 | 0.03087886 |
| MOGS | 13 | 0.03087886 |
| TRAPPC12 | 13 | 0.03087886 |
| FGD2 | 13 | 0.03087886 |
| OR4A15 | 13 | 0.03087886 |
| NETO1 | 13 | 0.03087886 |
| PNPLA7 | 13 | 0.03087886 |
| ITGAE | 13 | 0.03087886 |
| ESF1 | 13 | 0.03087886 |
| MED25 | 13 | 0.03087886 |
| IL21R | 13 | 0.03087886 |
| MCOLN1 | 13 | 0.03087886 |
| TMEM200A | 13 | 0.03087886 |
| MTDH | 13 | 0.03087886 |
| EZH2 | 13 | 0.03087886 |
| ZBTB38 | 13 | 0.03087886 |
| COBLL1 | 13 | 0.03087886 |
| PPP1R16B | 13 | 0.03087886 |
| SPARCL1 | 13 | 0.03087886 |
| MTUS1 | 13 | 0.03087886 |
| GPR162 | 13 | 0.03087886 |
| TAF1C | 13 | 0.03087886 |
| ERG | 13 | 0.03087886 |
| MAG | 13 | 0.03087886 |
| SEMA3E | 13 | 0.03087886 |
| KBTBD7 | 13 | 0.03087886 |
| TGM2 | 13 | 0.03087886 |
| SENP6 | 13 | 0.03087886 |
| KMT5B | 13 | 0.03087886 |
| PLEKHG6 | 13 | 0.03087886 |
| KCNH6 | 13 | 0.03087886 |
| TBX4 | 13 | 0.03087886 |
| POGK | 13 | 0.03087886 |
| FOCAD | 13 | 0.03087886 |
| SEMA3G | 13 | 0.03087886 |
| EEF2 | 13 | 0.03087886 |
| KEL | 13 | 0.03087886 |
| PCDHGA9 | 13 | 0.03087886 |
| CPXM2 | 13 | 0.03087886 |
| USP42 | 13 | 0.03087886 |
| PDZRN3 | 13 | 0.03087886 |
| SHOX | 13 | 0.03087886 |
| DOC2A | 13 | 0.03087886 |
| POU6F2 | 13 | 0.03087886 |
| FAM9A | 13 | 0.03087886 |
| PLCL1 | 13 | 0.03087886 |
| AVPR1A | 13 | 0.03087886 |
| KLC2 | 13 | 0.03087886 |
| RAI14 | 13 | 0.03087886 |
| RTL3 | 13 | 0.03087886 |
| DYRK3 | 13 | 0.03087886 |
| TRIM55 | 13 | 0.03087886 |
| OTOP2 | 13 | 0.03087886 |
| MCM7 | 13 | 0.03087886 |
| UGT2B15 | 13 | 0.03087886 |
| VILL | 13 | 0.03087886 |
| ZNF45 | 13 | 0.03087886 |
| GLG1 | 13 | 0.03087886 |
| PDE9A | 13 | 0.03087886 |
| TDRD5 | 13 | 0.03087886 |
| KRT85 | 13 | 0.03087886 |
| TP53BP2 | 13 | 0.03087886 |
| NEUROD4 | 13 | 0.03087886 |
| CLCA1 | 13 | 0.03087886 |
| SWT1 | 13 | 0.03087886 |
| GJA10 | 13 | 0.03087886 |
| USP3 | 13 | 0.03087886 |
| KIAA1586 | 13 | 0.03087886 |
| ZNF578 | 13 | 0.03087886 |
| RMC1 | 13 | 0.03087886 |
| GPC6 | 13 | 0.03087886 |
| CCDC18 | 13 | 0.03087886 |
| EFL1 | 13 | 0.03087886 |
| ERBB2 | 13 | 0.03087886 |
| FETUB | 13 | 0.03087886 |
| EIF5B | 13 | 0.03087886 |
| LIMA1 | 13 | 0.03087886 |
| SEMA4G | 13 | 0.03087886 |
| OSBPL5 | 13 | 0.03087886 |
| ARNTL2 | 13 | 0.03087886 |
| ZNF574 | 13 | 0.03087886 |
| AP3B2 | 13 | 0.03087886 |
| WNT1 | 13 | 0.03087886 |
| PKN3 | 13 | 0.03087886 |
| ADAMDEC1 | 13 | 0.03087886 |
| EML4 | 13 | 0.03087886 |
| SEMA6D | 13 | 0.03087886 |
| POLR3E | 13 | 0.03087886 |
| OR2T12 | 13 | 0.03087886 |
| POLR3B | 13 | 0.03087886 |
| GPLD1 | 13 | 0.03087886 |
| ZAP70 | 13 | 0.03087886 |
| VCPIP1 | 13 | 0.03087886 |
| DDX23 | 13 | 0.03087886 |
| FSHR | 13 | 0.03087886 |
| TRIM60 | 13 | 0.03087886 |
| ADAM23 | 13 | 0.03087886 |
| FBXO21 | 13 | 0.03087886 |
| MCM10 | 13 | 0.03087886 |
| AL136295.1 | 13 | 0.03087886 |
| PHKB | 13 | 0.03087886 |
| IPO7 | 13 | 0.03087886 |
| ATF5 | 13 | 0.03087886 |
| ABCB8 | 13 | 0.03087886 |
| PDE4A | 13 | 0.03087886 |
| AATK | 13 | 0.03087886 |
| PWP1 | 13 | 0.03087886 |
| ZNF667 | 13 | 0.03087886 |
| DENND2B | 13 | 0.03087886 |
| INTS7 | 13 | 0.03087886 |
| FBXL18 | 13 | 0.03087886 |
| SSTR4 | 13 | 0.03087886 |
| ZNF365 | 13 | 0.03087886 |
| CNGA3 | 13 | 0.03087886 |
| CDYL | 13 | 0.03087886 |
| PDZD8 | 13 | 0.03087886 |
| GABRB3 | 13 | 0.03087886 |
| CRISPLD1 | 13 | 0.03087886 |
| TMC2 | 13 | 0.03087886 |
| UHRF2 | 13 | 0.03087886 |
| EXOSC10 | 13 | 0.03087886 |
| AOC2 | 13 | 0.03087886 |
| RDX | 13 | 0.03087886 |
| UTP4 | 13 | 0.03087886 |
| RECQL5 | 13 | 0.03087886 |
| TTBK2 | 13 | 0.03087886 |
| VAV1 | 13 | 0.03087886 |
| PNPLA5 | 13 | 0.03087886 |
| ANGPT4 | 13 | 0.03087886 |
| CUL7 | 13 | 0.03087886 |
| UBQLN2 | 13 | 0.03087886 |
| CARD10 | 13 | 0.03087886 |
| SLC13A3 | 13 | 0.03087886 |
| OPTN | 13 | 0.03087886 |
| AGO1 | 13 | 0.03087886 |
| COL25A1 | 13 | 0.03087886 |
| BEND2 | 13 | 0.03087886 |
| MX1 | 13 | 0.03087886 |
| OLFM1 | 13 | 0.03087886 |
| IFIH1 | 13 | 0.03087886 |
| SYNJ1 | 13 | 0.03087886 |
| GRIN2C | 13 | 0.03087886 |
| TRAPPC10 | 13 | 0.03087886 |
| WWC1 | 13 | 0.03087886 |
| UACA | 13 | 0.03087886 |
| PTPN12 | 13 | 0.03087886 |
| PLEKHA7 | 13 | 0.03087886 |
| ARSJ | 13 | 0.03087886 |
| MYCBPAP | 13 | 0.03087886 |
| GTF2IRD2 | 13 | 0.03087886 |
| SRRT | 13 | 0.03087886 |
| IQCN | 13 | 0.03087886 |
| DCST2 | 13 | 0.03087886 |
| IDE | 13 | 0.03087886 |
| DOT1L | 13 | 0.03087886 |
| SEC24B | 13 | 0.03087886 |
| PLD1 | 13 | 0.03087886 |
| ST8SIA6 | 13 | 0.03087886 |
| JAG1 | 13 | 0.03087886 |
| ESYT3 | 13 | 0.03087886 |
| INO80 | 13 | 0.03087886 |
| INPP5F | 13 | 0.03087886 |
| SOCS5 | 13 | 0.03087886 |
| NDUFS1 | 13 | 0.03087886 |
| MARVELD2 | 13 | 0.03087886 |
| GPSM2 | 13 | 0.03087886 |
| MYOM3 | 13 | 0.03087886 |
| KLHL25 | 13 | 0.03087886 |
| AGBL1 | 13 | 0.03087886 |
| GZF1 | 13 | 0.03087886 |
| TFIP11 | 13 | 0.03087886 |
| MAP3K11 | 13 | 0.03087886 |
| TLR3 | 13 | 0.03087886 |
| NR3C2 | 13 | 0.03087886 |
| ZFYVE9 | 13 | 0.03087886 |
| OR5D14 | 13 | 0.03087886 |
| XRCC5 | 13 | 0.03087886 |
| CNR1 | 13 | 0.03087886 |
| TBC1D14 | 13 | 0.03087886 |
| ZNF431 | 13 | 0.03087886 |
| LZTS2 | 13 | 0.03087886 |
| TTYH1 | 13 | 0.03087886 |
| SLC7A2 | 13 | 0.03087886 |
| SYT16 | 13 | 0.03087886 |
| KCNQ1 | 13 | 0.03087886 |
| PRKCH | 13 | 0.03087886 |
| TLR9 | 13 | 0.03087886 |
| DKK2 | 13 | 0.03087886 |
| TTLL12 | 13 | 0.03087886 |
| TSC2 | 13 | 0.03087886 |
| SHC1 | 13 | 0.03087886 |
| ESCO2 | 13 | 0.03087886 |
| WDR59 | 13 | 0.03087886 |
| CEBPZ | 13 | 0.03087886 |
| CCDC40 | 13 | 0.03087886 |
| PHTF1 | 13 | 0.03087886 |
| DPY19L2 | 13 | 0.03087886 |
| L1TD1 | 13 | 0.03087886 |
| TBC1D1 | 13 | 0.03087886 |
| OSBPL7 | 13 | 0.03087886 |
| FGFR1 | 13 | 0.03087886 |
| SECISBP2L | 13 | 0.03087886 |
| SLC34A2 | 13 | 0.03087886 |
| TAF2 | 13 | 0.03087886 |
| PABPC5 | 13 | 0.03087886 |
| GTF3C2 | 13 | 0.03087886 |
| ABCF3 | 13 | 0.03087886 |
| PCDHGC3 | 13 | 0.03087886 |
| TRIM51 | 13 | 0.03087886 |
| TTLL4 | 13 | 0.03087886 |
| KDM5C | 13 | 0.03087886 |
| LARP4 | 13 | 0.03087886 |
| SCARA5 | 13 | 0.03087886 |
| NUTM1 | 13 | 0.03087886 |
| ITGB6 | 13 | 0.03087886 |
| OR5R1 | 13 | 0.03087886 |
| ZNF256 | 13 | 0.03087886 |
| UBR3 | 13 | 0.03087886 |
| RBBP8NL | 13 | 0.03087886 |
| RARB | 13 | 0.03087886 |
| KLHL9 | 13 | 0.03087886 |
| CLOCK | 13 | 0.03087886 |
| PHEX | 13 | 0.03087886 |
| ADAM10 | 13 | 0.03087886 |
| STOX1 | 13 | 0.03087886 |
| ARHGAP29 | 13 | 0.03087886 |
| RPS6KA6 | 13 | 0.03087886 |
| CLSTN3 | 13 | 0.03087886 |
| OLFML2A | 13 | 0.03087886 |
| SLC9A9 | 13 | 0.03087886 |
| RBBP7 | 13 | 0.03087886 |
| DSTYK | 13 | 0.03087886 |
| NOBOX | 13 | 0.03087886 |
| LATS2 | 13 | 0.03087886 |
| KIF3A | 13 | 0.03087886 |
| KRT4 | 13 | 0.03087886 |
| PNPLA6 | 13 | 0.03087886 |
| ACKR3 | 13 | 0.03087886 |
| SLCO4C1 | 13 | 0.03087886 |
| ZNF174 | 13 | 0.03087886 |
| ASIC2 | 13 | 0.03087886 |
| DENND1C | 13 | 0.03087886 |
| EPN1 | 13 | 0.03087886 |
| EEF1A2 | 13 | 0.03087886 |
| ERBIN | 13 | 0.03087886 |
| CCDC88B | 13 | 0.03087886 |
| SMG6 | 13 | 0.03087886 |
| NLRX1 | 13 | 0.03087886 |
| UBE3B | 13 | 0.03087886 |
| NAP1L2 | 13 | 0.03087886 |
| DHX36 | 13 | 0.03087886 |
| CEP135 | 13 | 0.03087886 |
| GFPT2 | 13 | 0.03087886 |
| MYBPC2 | 13 | 0.03087886 |
| C1S | 13 | 0.03087886 |
| RBM19 | 13 | 0.03087886 |
| DDX47 | 13 | 0.03087886 |
| FGA | 13 | 0.03087886 |
| ADCY7 | 13 | 0.03087886 |
| LIG1 | 13 | 0.03087886 |
| BRD2 | 13 | 0.03087886 |
| NLRP10 | 13 | 0.03087886 |
| KIF5C | 13 | 0.03087886 |
| CPSF6 | 13 | 0.03087886 |
| PRR14 | 13 | 0.03087886 |
| ITGA1 | 13 | 0.03087886 |
| SBF2 | 13 | 0.03087886 |
| TMC3 | 13 | 0.03087886 |
| CAPRIN2 | 13 | 0.03087886 |
| TNPO2 | 13 | 0.03087886 |
| DCST1 | 13 | 0.03087886 |
| NEXN | 13 | 0.03087886 |
| ZNF567 | 13 | 0.03087886 |
| CIZ1 | 13 | 0.03087886 |
| GALNTL6 | 13 | 0.03087886 |
| LPIN3 | 13 | 0.03087886 |
| ANAPC1 | 13 | 0.03087886 |
| TTC17 | 13 | 0.03087886 |
| AFP | 13 | 0.03087886 |
| FOXI1 | 13 | 0.03087886 |
| CD248 | 13 | 0.03087886 |
| PAPOLG | 13 | 0.03087886 |
| SATB2 | 13 | 0.03087886 |
| TMTC1 | 13 | 0.03087886 |
| RC3H1 | 13 | 0.03087886 |
| SLK | 13 | 0.03087886 |
| TBC1D8 | 13 | 0.03087886 |
| SLC25A12 | 13 | 0.03087886 |
| FTSJ3 | 13 | 0.03087886 |
| NYAP1 | 13 | 0.03087886 |
| ZNF569 | 13 | 0.03087886 |
| ALG8 | 13 | 0.03087886 |
| PUM1 | 13 | 0.03087886 |
| TRPM4 | 13 | 0.03087886 |
| TECPR2 | 13 | 0.03087886 |
| NKAPL | 13 | 0.03087886 |
| PKN2 | 13 | 0.03087886 |
| PCDHGC4 | 13 | 0.03087886 |
| CCAR2 | 13 | 0.03087886 |
| NEK1 | 13 | 0.03087886 |
| ADAM11 | 13 | 0.03087886 |
| KLHL42 | 13 | 0.03087886 |
| DLL1 | 13 | 0.03087886 |
| HTR1E | 13 | 0.03087886 |
| ZNF445 | 13 | 0.03087886 |
| FAM71B | 13 | 0.03087886 |
| KDM4A | 13 | 0.03087886 |
| HNRNPDL | 13 | 0.03087886 |
| ARHGAP39 | 13 | 0.03087886 |
| SEZ6L2 | 13 | 0.03087886 |
| ASAP1 | 13 | 0.03087886 |
| TRAM1L1 | 13 | 0.03087886 |
| KCNQ4 | 13 | 0.03087886 |
| ATP1A3 | 13 | 0.03087886 |
| FBLN2 | 13 | 0.03087886 |
| CDK14 | 13 | 0.03087886 |
| LMBRD1 | 13 | 0.03087886 |
| THAP12 | 13 | 0.03087886 |
| APAF1 | 13 | 0.03087886 |
| KCNC2 | 13 | 0.03087886 |
| HIPK1 | 13 | 0.03087886 |
| KIFC3 | 13 | 0.03087886 |
| SLC4A8 | 13 | 0.03087886 |
| MICALL2 | 13 | 0.03087886 |
| TGFBR1 | 13 | 0.03087886 |
| ISL1 | 13 | 0.03087886 |
| RBM15B | 13 | 0.03087886 |
| HRC | 13 | 0.03087886 |
| TECRL | 13 | 0.03087886 |
| ADCY4 | 13 | 0.03087886 |
| NFXL1 | 13 | 0.03087886 |
| GALNTL5 | 13 | 0.03087886 |
| MAP7 | 13 | 0.03087886 |
| SYVN1 | 13 | 0.03087886 |
| STK10 | 13 | 0.03087886 |
| PHLPP2 | 13 | 0.03087886 |
| SIN3B | 13 | 0.03087886 |
| DACH2 | 13 | 0.03087886 |
| HIC2 | 13 | 0.03087886 |
| ZNF454 | 13 | 0.03087886 |
| ROR1 | 13 | 0.03087886 |
| ZNF546 | 13 | 0.03087886 |
| NFATC3 | 13 | 0.03087886 |
| DNM2 | 13 | 0.03087886 |
| TPTE2 | 13 | 0.03087886 |
| ABCD1 | 13 | 0.03087886 |
| RAVER1 | 13 | 0.03087886 |
| AFF1 | 13 | 0.03087886 |
| MAF | 13 | 0.03087886 |
| SERPINE3 | 13 | 0.03087886 |
| UGT8 | 13 | 0.03087886 |
| LRRC37A2 | 13 | 0.03087886 |
| CCDC102B | 13 | 0.03087886 |
| SLC11A1 | 13 | 0.03087886 |
| ADGRE5 | 13 | 0.03087886 |
| CEP83 | 13 | 0.03087886 |
| APPL1 | 13 | 0.03087886 |
| SARDH | 13 | 0.03087886 |
| CP | 13 | 0.03087886 |
| CHST10 | 13 | 0.03087886 |
| CHAF1A | 13 | 0.03087886 |
| PER1 | 13 | 0.03087886 |
| BANK1 | 13 | 0.03087886 |
| SLFN11 | 13 | 0.03087886 |
| CARMIL2 | 13 | 0.03087886 |
| EIF4G2 | 13 | 0.03087886 |
| PRKAA2 | 13 | 0.03087886 |
| GDF2 | 13 | 0.03087886 |
| CPXM1 | 13 | 0.03087886 |
| PABPC3 | 13 | 0.03087886 |
| DPP8 | 13 | 0.03087886 |
| MTREX | 13 | 0.03087886 |
| UGGT1 | 13 | 0.03087886 |
| GABBR2 | 13 | 0.03087886 |
| PEX1 | 13 | 0.03087886 |
| TTI1 | 13 | 0.03087886 |
| WNT5A | 13 | 0.03087886 |
| ACAP1 | 13 | 0.03087886 |
| CD180 | 13 | 0.03087886 |
| HDGFL2 | 13 | 0.03087886 |
| GBE1 | 13 | 0.03087886 |
| USP8 | 13 | 0.03087886 |
| CILP2 | 13 | 0.03087886 |
| FNBP1 | 13 | 0.03087886 |
| FAM83C | 13 | 0.03087886 |
| WDR6 | 13 | 0.03087886 |
| PROX1 | 13 | 0.03087886 |
| RIN2 | 13 | 0.03087886 |
| KRT34 | 13 | 0.03087886 |
| ZNF761 | 13 | 0.03087886 |
| PARP1 | 13 | 0.03087886 |
| DGKH | 13 | 0.03087886 |
| KRT73 | 13 | 0.03087886 |
| OTOL1 | 13 | 0.03087886 |
| FYN | 13 | 0.03087886 |
| KLF3 | 13 | 0.03087886 |
| ASB5 | 13 | 0.03087886 |
| XKR3 | 13 | 0.03087886 |
| MOV10 | 13 | 0.03087886 |
| UNC5A | 13 | 0.03087886 |
| ZFPM2 | 13 | 0.03087886 |
| ELL | 12 | 0.028503563 |
| MARCO | 12 | 0.028503563 |
| REPS1 | 12 | 0.028503563 |
| ADGRA2 | 12 | 0.028503563 |
| ZNF331 | 12 | 0.028503563 |
| ARNTL | 12 | 0.028503563 |
| GUCY2D | 12 | 0.028503563 |
| MPP4 | 12 | 0.028503563 |
| HTR1B | 12 | 0.028503563 |
| HRH2 | 12 | 0.028503563 |
| DDX50 | 12 | 0.028503563 |
| KCNT1 | 12 | 0.028503563 |
| ADAMTS8 | 12 | 0.028503563 |
| ZNRF3 | 12 | 0.028503563 |
| PASD1 | 12 | 0.028503563 |
| ZKSCAN5 | 12 | 0.028503563 |
| FBXO40 | 12 | 0.028503563 |
| KIF24 | 12 | 0.028503563 |
| ZNF326 | 12 | 0.028503563 |
| ESYT2 | 12 | 0.028503563 |
| COPB1 | 12 | 0.028503563 |
| NCKAP1 | 12 | 0.028503563 |
| ARHGAP24 | 12 | 0.028503563 |
| SSH1 | 12 | 0.028503563 |
| CORO7 | 12 | 0.028503563 |
| MEF2C | 12 | 0.028503563 |
| TMEM63B | 12 | 0.028503563 |
| TAX1BP1 | 12 | 0.028503563 |
| ETAA1 | 12 | 0.028503563 |
| GALNT14 | 12 | 0.028503563 |
| SUN2 | 12 | 0.028503563 |
| INTS5 | 12 | 0.028503563 |
| ZSCAN31 | 12 | 0.028503563 |
| ITPRID1 | 12 | 0.028503563 |
| GOT1L1 | 12 | 0.028503563 |
| MCTP2 | 12 | 0.028503563 |
| SOX11 | 12 | 0.028503563 |
| PPP2R2C | 12 | 0.028503563 |
| OR5D18 | 12 | 0.028503563 |
| RGL2 | 12 | 0.028503563 |
| ABLIM2 | 12 | 0.028503563 |
| SUGP2 | 12 | 0.028503563 |
| EMC1 | 12 | 0.028503563 |
| USP47 | 12 | 0.028503563 |
| FXR2 | 12 | 0.028503563 |
| CFAP70 | 12 | 0.028503563 |
| TNFRSF19 | 12 | 0.028503563 |
| DUSP16 | 12 | 0.028503563 |
| TRAF7 | 12 | 0.028503563 |
| DAAM1 | 12 | 0.028503563 |
| PKP3 | 12 | 0.028503563 |
| STARD13 | 12 | 0.028503563 |
| PLAT | 12 | 0.028503563 |
| GPR139 | 12 | 0.028503563 |
| ABCD2 | 12 | 0.028503563 |
| TMTC3 | 12 | 0.028503563 |
| HECTD2 | 12 | 0.028503563 |
| LRRC36 | 12 | 0.028503563 |
| ZBTB16 | 12 | 0.028503563 |
| ARHGAP17 | 12 | 0.028503563 |
| L3MBTL3 | 12 | 0.028503563 |
| ATP7B | 12 | 0.028503563 |
| PIK3R5 | 12 | 0.028503563 |
| KCNG2 | 12 | 0.028503563 |
| NRROS | 12 | 0.028503563 |
| NUP188 | 12 | 0.028503563 |
| CEP70 | 12 | 0.028503563 |
| PGBD5 | 12 | 0.028503563 |
| DDX31 | 12 | 0.028503563 |
| SRPK1 | 12 | 0.028503563 |
| PMS1 | 12 | 0.028503563 |
| CCDC92 | 12 | 0.028503563 |
| ZIC1 | 12 | 0.028503563 |
| MCOLN3 | 12 | 0.028503563 |
| ARHGEF25 | 12 | 0.028503563 |
| PDYN | 12 | 0.028503563 |
| SCG2 | 12 | 0.028503563 |
| MCPH1 | 12 | 0.028503563 |
| PLB1 | 12 | 0.028503563 |
| EXOC6B | 12 | 0.028503563 |
| PPP5C | 12 | 0.028503563 |
| ADAM2 | 12 | 0.028503563 |
| DENND4C | 12 | 0.028503563 |
| PDE11A | 12 | 0.028503563 |
| WFS1 | 12 | 0.028503563 |
| ZNF419 | 12 | 0.028503563 |
| EEF2K | 12 | 0.028503563 |
| ZNF687 | 12 | 0.028503563 |
| COL6A5 | 12 | 0.028503563 |
| CDYL2 | 12 | 0.028503563 |
| ZNF28 | 12 | 0.028503563 |
| SCUBE2 | 12 | 0.028503563 |
| MTMR6 | 12 | 0.028503563 |
| PCDHB11 | 12 | 0.028503563 |
| TFAP2B | 12 | 0.028503563 |
| PTK7 | 12 | 0.028503563 |
| SLC38A9 | 12 | 0.028503563 |
| ZNF420 | 12 | 0.028503563 |
| PKD2L2 | 12 | 0.028503563 |
| SNX2 | 12 | 0.028503563 |
| ABCG2 | 12 | 0.028503563 |
| WWOX | 12 | 0.028503563 |
| DCAF6 | 12 | 0.028503563 |
| CCP110 | 12 | 0.028503563 |
| ADAMTS6 | 12 | 0.028503563 |
| KIF18B | 12 | 0.028503563 |
| MARK3 | 12 | 0.028503563 |
| SLC26A3 | 12 | 0.028503563 |
| IQCE | 12 | 0.028503563 |
| KNOP1 | 12 | 0.028503563 |
| CSF2RA | 12 | 0.028503563 |
| SOGA1 | 12 | 0.028503563 |
| TLE2 | 12 | 0.028503563 |
| BANP | 12 | 0.028503563 |
| FIG4 | 12 | 0.028503563 |
| DARS2 | 12 | 0.028503563 |
| BBS2 | 12 | 0.028503563 |
| NPY2R | 12 | 0.028503563 |
| CYLD | 12 | 0.028503563 |
| FAHD2B | 12 | 0.028503563 |
| SFI1 | 12 | 0.028503563 |
| LARP1 | 12 | 0.028503563 |
| TARBP2 | 12 | 0.028503563 |
| TOX4 | 12 | 0.028503563 |
| CCDC87 | 12 | 0.028503563 |
| JAKMIP3 | 12 | 0.028503563 |
| GABRG2 | 12 | 0.028503563 |
| DSG3 | 12 | 0.028503563 |
| EFHC2 | 12 | 0.028503563 |
| SF1 | 12 | 0.028503563 |
| PALM2AKAP2 | 12 | 0.028503563 |
| LRRC17 | 12 | 0.028503563 |
| TBC1D8B | 12 | 0.028503563 |
| SLC6A1 | 12 | 0.028503563 |
| THOC1 | 12 | 0.028503563 |
| OTUD7A | 12 | 0.028503563 |
| ADD2 | 12 | 0.028503563 |
| JAK3 | 12 | 0.028503563 |
| PPP2R1A | 12 | 0.028503563 |
| SMO | 12 | 0.028503563 |
| ZNF19 | 12 | 0.028503563 |
| IL12RB2 | 12 | 0.028503563 |
| MEIS2 | 12 | 0.028503563 |
| ZNF518B | 12 | 0.028503563 |
| ERCC6L2 | 12 | 0.028503563 |
| ME1 | 12 | 0.028503563 |
| RABGAP1L | 12 | 0.028503563 |
| CLCN1 | 12 | 0.028503563 |
| ANO8 | 12 | 0.028503563 |
| PHACTR2 | 12 | 0.028503563 |
| RHOBTB1 | 12 | 0.028503563 |
| DNAJC10 | 12 | 0.028503563 |
| DSG1 | 12 | 0.028503563 |
| NPBWR1 | 12 | 0.028503563 |
| DSG4 | 12 | 0.028503563 |
| DZIP3 | 12 | 0.028503563 |
| ZNF205 | 12 | 0.028503563 |
| ZNF827 | 12 | 0.028503563 |
| DDX18 | 12 | 0.028503563 |
| SIMC1 | 12 | 0.028503563 |
| DBF4B | 12 | 0.028503563 |
| HERC5 | 12 | 0.028503563 |
| ZNF26 | 12 | 0.028503563 |
| MTHFD1L | 12 | 0.028503563 |
| CYP1B1 | 12 | 0.028503563 |
| BCL6 | 12 | 0.028503563 |
| KRT31 | 12 | 0.028503563 |
| ZNF34 | 12 | 0.028503563 |
| TM9SF3 | 12 | 0.028503563 |
| CYP4B1 | 12 | 0.028503563 |
| TET2 | 12 | 0.028503563 |
| LRRC4C | 12 | 0.028503563 |
| CSPP1 | 12 | 0.028503563 |
| MAB21L2 | 12 | 0.028503563 |
| ZIC3 | 12 | 0.028503563 |
| PRAMEF20 | 12 | 0.028503563 |
| MROH7 | 12 | 0.028503563 |
| TCERG1L | 12 | 0.028503563 |
| NRBP1 | 12 | 0.028503563 |
| GARNL3 | 12 | 0.028503563 |
| IPO9 | 12 | 0.028503563 |
| IGDCC3 | 12 | 0.028503563 |
| USP40 | 12 | 0.028503563 |
| RNF216 | 12 | 0.028503563 |
| DNM1 | 12 | 0.028503563 |
| RASEF | 12 | 0.028503563 |
| USP10 | 12 | 0.028503563 |
| ABCG4 | 12 | 0.028503563 |
| ALX4 | 12 | 0.028503563 |
| THRB | 12 | 0.028503563 |
| HHIPL2 | 12 | 0.028503563 |
| OR8H2 | 12 | 0.028503563 |
| KRT71 | 12 | 0.028503563 |
| OR4A5 | 12 | 0.028503563 |
| OXR1 | 12 | 0.028503563 |
| SLC35F3 | 12 | 0.028503563 |
| RNASEL | 12 | 0.028503563 |
| E2F8 | 12 | 0.028503563 |
| BAIAP2L1 | 12 | 0.028503563 |
| BMP10 | 12 | 0.028503563 |
| GTF3C3 | 12 | 0.028503563 |
| RING1 | 12 | 0.028503563 |
| BMPR1B | 12 | 0.028503563 |
| LIMCH1 | 12 | 0.028503563 |
| CDH7 | 12 | 0.028503563 |
| SLC27A5 | 12 | 0.028503563 |
| CCDC85A | 12 | 0.028503563 |
| WASHC4 | 12 | 0.028503563 |
| PABPC4 | 12 | 0.028503563 |
| MCM6 | 12 | 0.028503563 |
| URGCP | 12 | 0.028503563 |
| ITGB3 | 12 | 0.028503563 |
| PKP4 | 12 | 0.028503563 |
| HS6ST3 | 12 | 0.028503563 |
| LONRF1 | 12 | 0.028503563 |
| TLL2 | 12 | 0.028503563 |
| SLTM | 12 | 0.028503563 |
| GNB4 | 12 | 0.028503563 |
| TMEM132A | 12 | 0.028503563 |
| ZNF229 | 12 | 0.028503563 |
| WDR11 | 12 | 0.028503563 |
| ACLY | 12 | 0.028503563 |
| ZNF595 | 12 | 0.028503563 |
| NPHP3 | 12 | 0.028503563 |
| RIPK4 | 12 | 0.028503563 |
| NEK5 | 12 | 0.028503563 |
| GPR50 | 12 | 0.028503563 |
| ARHGAP27 | 12 | 0.028503563 |
| OR5I1 | 12 | 0.028503563 |
| LRRC31 | 12 | 0.028503563 |
| NUP153 | 12 | 0.028503563 |
| MOS | 12 | 0.028503563 |
| ENOX2 | 12 | 0.028503563 |
| TEX2 | 12 | 0.028503563 |
| ADAM18 | 12 | 0.028503563 |
| ADAMTS19 | 12 | 0.028503563 |
| MLLT10 | 12 | 0.028503563 |
| CYP20A1 | 12 | 0.028503563 |
| KIAA0408 | 12 | 0.028503563 |
| KIF1C | 12 | 0.028503563 |
| ENPEP | 12 | 0.028503563 |
| ZNF442 | 12 | 0.028503563 |
| SLC13A1 | 12 | 0.028503563 |
| ZNF227 | 12 | 0.028503563 |
| PDCD11 | 12 | 0.028503563 |
| MTR | 12 | 0.028503563 |
| SPG7 | 12 | 0.028503563 |
| LRPPRC | 12 | 0.028503563 |
| SMG5 | 12 | 0.028503563 |
| KCNA6 | 12 | 0.028503563 |
| ZNF721 | 12 | 0.028503563 |
| CAMK1D | 12 | 0.028503563 |
| LETM1 | 12 | 0.028503563 |
| SLC12A2 | 12 | 0.028503563 |
| SATB1 | 12 | 0.028503563 |
| ATXN2 | 12 | 0.028503563 |
| TGM5 | 12 | 0.028503563 |
| GPHN | 12 | 0.028503563 |
| ABCC6 | 12 | 0.028503563 |
| SYNE3 | 12 | 0.028503563 |
| RXRG | 12 | 0.028503563 |
| CHST1 | 12 | 0.028503563 |
| AKAP3 | 12 | 0.028503563 |
| COL4A3 | 12 | 0.028503563 |
| MYO6 | 12 | 0.028503563 |
| THEMIS | 12 | 0.028503563 |
| DNTTIP2 | 12 | 0.028503563 |
| EPB41 | 12 | 0.028503563 |
| RASA3 | 12 | 0.028503563 |
| SEC14L1 | 12 | 0.028503563 |
| FSCB | 12 | 0.028503563 |
| SIX4 | 12 | 0.028503563 |
| CEMIP2 | 12 | 0.028503563 |
| RTL5 | 12 | 0.028503563 |
| VN1R2 | 12 | 0.028503563 |
| TTLL5 | 12 | 0.028503563 |
| LAMB3 | 12 | 0.028503563 |
| MFGE8 | 12 | 0.028503563 |
| RADX | 12 | 0.028503563 |
| ETNPPL | 12 | 0.028503563 |
| DCAF4L1 | 12 | 0.028503563 |
| HDAC6 | 12 | 0.028503563 |
| NDST2 | 12 | 0.028503563 |
| OSBPL1A | 12 | 0.028503563 |
| PDE4D | 12 | 0.028503563 |
| THOC5 | 12 | 0.028503563 |
| MPP2 | 12 | 0.028503563 |
| ZNF568 | 12 | 0.028503563 |
| RTEL1 | 12 | 0.028503563 |
| MINDY4 | 12 | 0.028503563 |
| ZSCAN29 | 12 | 0.028503563 |
| ACP7 | 12 | 0.028503563 |
| PCOLCE2 | 12 | 0.028503563 |
| GPR156 | 12 | 0.028503563 |
| UPF3A | 12 | 0.028503563 |
| SH3TC2 | 12 | 0.028503563 |
| KHSRP | 12 | 0.028503563 |
| NPR1 | 12 | 0.028503563 |
| BRD8 | 12 | 0.028503563 |
| TCF12 | 12 | 0.028503563 |
| FARP1 | 12 | 0.028503563 |
| EHMT2 | 12 | 0.028503563 |
| MED14 | 12 | 0.028503563 |
| ARHGAP11A | 12 | 0.028503563 |
| C14orf39 | 12 | 0.028503563 |
| ZNF329 | 12 | 0.028503563 |
| OR6C75 | 12 | 0.028503563 |
| MAP3K14 | 12 | 0.028503563 |
| P2RY8 | 12 | 0.028503563 |
| CHRNA4 | 12 | 0.028503563 |
| QRICH1 | 12 | 0.028503563 |
| ZNF648 | 12 | 0.028503563 |
| HAUS6 | 12 | 0.028503563 |
| ERCC4 | 12 | 0.028503563 |
| PDE1B | 12 | 0.028503563 |
| TBC1D10C | 12 | 0.028503563 |
| ZIK1 | 12 | 0.028503563 |
| EIF3B | 12 | 0.028503563 |
| MYBL1 | 12 | 0.028503563 |
| NTN4 | 12 | 0.028503563 |
| ANKRD44 | 12 | 0.028503563 |
| AKAP4 | 12 | 0.028503563 |
| CEP104 | 12 | 0.028503563 |
| ETV5 | 12 | 0.028503563 |
| FBXO34 | 12 | 0.028503563 |
| ANGPTL2 | 12 | 0.028503563 |
| PTOV1 | 12 | 0.028503563 |
| CDH20 | 12 | 0.028503563 |
| FAM13A | 12 | 0.028503563 |
| RNF31 | 12 | 0.028503563 |
| SIPA1 | 12 | 0.028503563 |
| ZBTB4 | 12 | 0.028503563 |
| INTS8 | 12 | 0.028503563 |
| GBA2 | 12 | 0.028503563 |
| INPP4B | 12 | 0.028503563 |
| POLK | 12 | 0.028503563 |
| LTBP3 | 12 | 0.028503563 |
| KLHL40 | 12 | 0.028503563 |
| ZMYM5 | 12 | 0.028503563 |
| TGM6 | 12 | 0.028503563 |
| PTCH2 | 12 | 0.028503563 |
| GPRASP2 | 12 | 0.028503563 |
| TYK2 | 12 | 0.028503563 |
| LMTK2 | 12 | 0.028503563 |
| TUSC3 | 12 | 0.028503563 |
| FAM114A1 | 12 | 0.028503563 |
| KLHL14 | 12 | 0.028503563 |
| FAM120B | 12 | 0.028503563 |
| CFAP94 | 12 | 0.028503563 |
| MYBPH | 12 | 0.028503563 |
| NUP107 | 12 | 0.028503563 |
| ACTN1 | 12 | 0.028503563 |
| TRIM33 | 12 | 0.028503563 |
| ZNF148 | 12 | 0.028503563 |
| NPTX2 | 12 | 0.028503563 |
| CES3 | 12 | 0.028503563 |
| ZNF398 | 12 | 0.028503563 |
| FAM171B | 12 | 0.028503563 |
| KIF18A | 12 | 0.028503563 |
| SKIV2L | 12 | 0.028503563 |
| ZKSCAN2 | 12 | 0.028503563 |
| MBTPS1 | 12 | 0.028503563 |
| GABRA2 | 12 | 0.028503563 |
| GATAD2A | 12 | 0.028503563 |
| CSF2RB | 12 | 0.028503563 |
| TECPR1 | 12 | 0.028503563 |
| AP2A1 | 12 | 0.028503563 |
| CBFA2T3 | 12 | 0.028503563 |
| LEMD3 | 12 | 0.028503563 |
| NLGN1 | 12 | 0.028503563 |
| KCNH2 | 12 | 0.028503563 |
| HAPLN1 | 12 | 0.028503563 |
| PDILT | 12 | 0.028503563 |
| IQGAP1 | 12 | 0.028503563 |
| KCNV1 | 12 | 0.028503563 |
| DNA2 | 12 | 0.028503563 |
| MAPK8IP1 | 12 | 0.028503563 |
| LAMC2 | 12 | 0.028503563 |
| NRP2 | 12 | 0.028503563 |
| ATXN1 | 12 | 0.028503563 |
| ESCO1 | 12 | 0.028503563 |
| EYA4 | 12 | 0.028503563 |
| LSR | 12 | 0.028503563 |
| SLC26A9 | 12 | 0.028503563 |
| ADGRG3 | 12 | 0.028503563 |
| CD22 | 12 | 0.028503563 |
| MICAL1 | 12 | 0.028503563 |
| BCR | 12 | 0.028503563 |
| LEO1 | 12 | 0.028503563 |
| ZNF780A | 12 | 0.028503563 |
| TBX20 | 12 | 0.028503563 |
| MMP13 | 12 | 0.028503563 |
| LZTS1 | 12 | 0.028503563 |
| DGCR2 | 12 | 0.028503563 |
| ZNF37A | 12 | 0.028503563 |
| KIAA1522 | 12 | 0.028503563 |
| APBB3 | 12 | 0.028503563 |
| NRDE2 | 12 | 0.028503563 |
| RNF128 | 12 | 0.028503563 |
| JAK2 | 12 | 0.028503563 |
| TLR8 | 12 | 0.028503563 |
| NEK4 | 12 | 0.028503563 |
| F2 | 12 | 0.028503563 |
| XPO5 | 12 | 0.028503563 |
| DOK5 | 12 | 0.028503563 |
| PTPN21 | 12 | 0.028503563 |
| FZD6 | 12 | 0.028503563 |
| GMPS | 12 | 0.028503563 |
| ITGB1 | 12 | 0.028503563 |
| GCNT4 | 12 | 0.028503563 |
| RIPOR1 | 12 | 0.028503563 |
| TBC1D22A | 12 | 0.028503563 |
| TMEM63C | 12 | 0.028503563 |
| ZNF16 | 12 | 0.028503563 |
| SOX7 | 12 | 0.028503563 |
| HSP90B1 | 12 | 0.028503563 |
| ZCCHC2 | 12 | 0.028503563 |
| PDE2A | 12 | 0.028503563 |
| MTO1 | 12 | 0.028503563 |
| CDHR2 | 12 | 0.028503563 |
| BRF1 | 12 | 0.028503563 |
| STAC | 12 | 0.028503563 |
| PPP2R2B | 12 | 0.028503563 |
| NIBAN1 | 12 | 0.028503563 |
| ADCY3 | 12 | 0.028503563 |
| BBS9 | 12 | 0.028503563 |
| DDR1 | 12 | 0.028503563 |
| POU3F4 | 12 | 0.028503563 |
| DDX43 | 12 | 0.028503563 |
| PACS2 | 12 | 0.028503563 |
| ANO6 | 12 | 0.028503563 |
| SUSD5 | 12 | 0.028503563 |
| MAN1A1 | 12 | 0.028503563 |
| PHF14 | 12 | 0.028503563 |
| RBPJL | 12 | 0.028503563 |
| PRICKLE2 | 12 | 0.028503563 |
| TBC1D4 | 12 | 0.028503563 |
| TGM7 | 12 | 0.028503563 |
| SPATA5 | 12 | 0.028503563 |
| TRAK2 | 12 | 0.028503563 |
| MAP3K19 | 12 | 0.028503563 |
| GABRA6 | 12 | 0.028503563 |
| IL6ST | 12 | 0.028503563 |
| LMBRD2 | 12 | 0.028503563 |
| AGAP2 | 12 | 0.028503563 |
| SLC6A11 | 12 | 0.028503563 |
| TBC1D5 | 12 | 0.028503563 |
| PLA2G4A | 12 | 0.028503563 |
| AKAP7 | 12 | 0.028503563 |
| AKT1 | 12 | 0.028503563 |
| OR2L3 | 12 | 0.028503563 |
| TP63 | 12 | 0.028503563 |
| SPATA17 | 12 | 0.028503563 |
| ULK4 | 12 | 0.028503563 |
| ZNF404 | 12 | 0.028503563 |
| POLR3A | 12 | 0.028503563 |
| AOC1 | 12 | 0.028503563 |
| CCPG1 | 12 | 0.028503563 |
| MYO19 | 12 | 0.028503563 |
| WASHC2A | 12 | 0.028503563 |
| BPIFB1 | 12 | 0.028503563 |
| MPHOSPH8 | 12 | 0.028503563 |
| HLX | 12 | 0.028503563 |
| CDK13 | 12 | 0.028503563 |
| FBXO38 | 12 | 0.028503563 |
| FAM78B | 12 | 0.028503563 |
| PPP1R10 | 12 | 0.028503563 |
| CRAT | 12 | 0.028503563 |
| CATSPER1 | 12 | 0.028503563 |
| SEMA3F | 12 | 0.028503563 |
| ANKS6 | 12 | 0.028503563 |
| CLCN3 | 12 | 0.028503563 |
| SYNM | 12 | 0.028503563 |
| COLGALT1 | 12 | 0.028503563 |
| ASIC5 | 12 | 0.028503563 |
| CTDP1 | 12 | 0.028503563 |
| MAPT | 12 | 0.028503563 |
| KRT75 | 12 | 0.028503563 |
| ATIC | 12 | 0.028503563 |
| RGL3 | 12 | 0.028503563 |
| FZD7 | 12 | 0.028503563 |
| KCND1 | 12 | 0.028503563 |
| CNGA1 | 12 | 0.028503563 |
| ATP13A2 | 12 | 0.028503563 |
| CACNA2D2 | 12 | 0.028503563 |
| RASAL2 | 12 | 0.028503563 |
| BBX | 12 | 0.028503563 |
| ZNF749 | 12 | 0.028503563 |
| TAF4 | 12 | 0.028503563 |
| ANGPT2 | 12 | 0.028503563 |
| PKLR | 12 | 0.028503563 |
| AMOT | 12 | 0.028503563 |
| RANBP17 | 12 | 0.028503563 |
| ADAMTS14 | 12 | 0.028503563 |
| SLC23A1 | 12 | 0.028503563 |
| SH2D3C | 12 | 0.028503563 |
| GPR101 | 12 | 0.028503563 |
| TRPV2 | 12 | 0.028503563 |
| N4BP1 | 12 | 0.028503563 |
| FZD3 | 12 | 0.028503563 |
| CRACDL | 12 | 0.028503563 |
| RECK | 12 | 0.028503563 |
| MYORG | 12 | 0.028503563 |
| CC2D1B | 12 | 0.028503563 |
| GRM4 | 12 | 0.028503563 |
| ARHGAP4 | 12 | 0.028503563 |
| BMS1 | 12 | 0.028503563 |
| ABTB1 | 12 | 0.028503563 |
| GRIP1 | 12 | 0.028503563 |
| VIT | 12 | 0.028503563 |
| RRP12 | 12 | 0.028503563 |
| TGFBR3 | 12 | 0.028503563 |
| PALB2 | 12 | 0.028503563 |
| SEMA6B | 12 | 0.028503563 |
| TTC14 | 12 | 0.028503563 |
| USHBP1 | 11 | 0.026128266 |
| PTPRO | 11 | 0.026128266 |
| NEK8 | 11 | 0.026128266 |
| ACE2 | 11 | 0.026128266 |
| THBS3 | 11 | 0.026128266 |
| CAMKK2 | 11 | 0.026128266 |
| LANCL2 | 11 | 0.026128266 |
| POR | 11 | 0.026128266 |
| TYR | 11 | 0.026128266 |
| TMPRSS9 | 11 | 0.026128266 |
| RHBG | 11 | 0.026128266 |
| PRKD3 | 11 | 0.026128266 |
| CFAP46 | 11 | 0.026128266 |
| ATP9B | 11 | 0.026128266 |
| WDHD1 | 11 | 0.026128266 |
| L1CAM | 11 | 0.026128266 |
| TAOK1 | 11 | 0.026128266 |
| SMARCC2 | 11 | 0.026128266 |
| FOXP2 | 11 | 0.026128266 |
| LRRC37A3 | 11 | 0.026128266 |
| ZNF714 | 11 | 0.026128266 |
| PPFIBP2 | 11 | 0.026128266 |
| NEFL | 11 | 0.026128266 |
| PIK3CD | 11 | 0.026128266 |
| COL8A1 | 11 | 0.026128266 |
| DNAI2 | 11 | 0.026128266 |
| NAPEPLD | 11 | 0.026128266 |
| HPSE | 11 | 0.026128266 |
| FKBP10 | 11 | 0.026128266 |
| TSPAN10 | 11 | 0.026128266 |
| PHACTR3 | 11 | 0.026128266 |
| CEP95 | 11 | 0.026128266 |
| TMC5 | 11 | 0.026128266 |
| LIMK1 | 11 | 0.026128266 |
| NTM | 11 | 0.026128266 |
| RCBTB2 | 11 | 0.026128266 |
| SMOX | 11 | 0.026128266 |
| MATK | 11 | 0.026128266 |
| TMCO6 | 11 | 0.026128266 |
| N4BP2L2 | 11 | 0.026128266 |
| ODF2L | 11 | 0.026128266 |
| DCAF8L1 | 11 | 0.026128266 |
| TM7SF3 | 11 | 0.026128266 |
| AKNA | 11 | 0.026128266 |
| ZNF583 | 11 | 0.026128266 |
| ECE1 | 11 | 0.026128266 |
| ARSG | 11 | 0.026128266 |
| MPL | 11 | 0.026128266 |
| SLC5A8 | 11 | 0.026128266 |
| ANKFN1 | 11 | 0.026128266 |
| FAM186B | 11 | 0.026128266 |
| OR2L13 | 11 | 0.026128266 |
| ITPKB | 11 | 0.026128266 |
| SKIDA1 | 11 | 0.026128266 |
| RAPGEF4 | 11 | 0.026128266 |
| VEGFC | 11 | 0.026128266 |
| PPFIA3 | 11 | 0.026128266 |
| ADA2 | 11 | 0.026128266 |
| SLC4A2 | 11 | 0.026128266 |
| ILDR1 | 11 | 0.026128266 |
| TTLL8 | 11 | 0.026128266 |
| SMARCAL1 | 11 | 0.026128266 |
| TNFAIP3 | 11 | 0.026128266 |
| BCAM | 11 | 0.026128266 |
| GOLGA1 | 11 | 0.026128266 |
| SLC25A31 | 11 | 0.026128266 |
| OR4P4 | 11 | 0.026128266 |
| UGP2 | 11 | 0.026128266 |
| FBXO30 | 11 | 0.026128266 |
| CHERP | 11 | 0.026128266 |
| KCNJ4 | 11 | 0.026128266 |
| EPHX1 | 11 | 0.026128266 |
| GLT8D1 | 11 | 0.026128266 |
| ANKS1A | 11 | 0.026128266 |
| VTN | 11 | 0.026128266 |
| ACAD11 | 11 | 0.026128266 |
| SMARCB1 | 11 | 0.026128266 |
| ZNF493 | 11 | 0.026128266 |
| POMT2 | 11 | 0.026128266 |
| TP73 | 11 | 0.026128266 |
| ARV1 | 11 | 0.026128266 |
| NOP2 | 11 | 0.026128266 |
| BEND5 | 11 | 0.026128266 |
| PREP | 11 | 0.026128266 |
| UBASH3A | 11 | 0.026128266 |
| OR4C15 | 11 | 0.026128266 |
| TREH | 11 | 0.026128266 |
| SREBF1 | 11 | 0.026128266 |
| HRG | 11 | 0.026128266 |
| ZNF350 | 11 | 0.026128266 |
| CRAMP1 | 11 | 0.026128266 |
| IPO4 | 11 | 0.026128266 |
| RUNX1 | 11 | 0.026128266 |
| ZFP90 | 11 | 0.026128266 |
| SORBS1 | 11 | 0.026128266 |
| REXO5 | 11 | 0.026128266 |
| ACSM1 | 11 | 0.026128266 |
| ELFN2 | 11 | 0.026128266 |
| TMPRSS11A | 11 | 0.026128266 |
| SNX18 | 11 | 0.026128266 |
| CYP4F3 | 11 | 0.026128266 |
| RGS6 | 11 | 0.026128266 |
| QKI | 11 | 0.026128266 |
| ZNF248 | 11 | 0.026128266 |
| NFE2L3 | 11 | 0.026128266 |
| VLDLR | 11 | 0.026128266 |
| ELAPOR2 | 11 | 0.026128266 |
| ADAM30 | 11 | 0.026128266 |
| EGR3 | 11 | 0.026128266 |
| MAP7D2 | 11 | 0.026128266 |
| TBC1D16 | 11 | 0.026128266 |
| OR2M3 | 11 | 0.026128266 |
| DPP3 | 11 | 0.026128266 |
| IPO13 | 11 | 0.026128266 |
| PIAS4 | 11 | 0.026128266 |
| GRAMD1B | 11 | 0.026128266 |
| SPATA16 | 11 | 0.026128266 |
| ABLIM3 | 11 | 0.026128266 |
| GALNT3 | 11 | 0.026128266 |
| SREBF2 | 11 | 0.026128266 |
| ST8SIA4 | 11 | 0.026128266 |
| LCA5 | 11 | 0.026128266 |
| PARP9 | 11 | 0.026128266 |
| FAM120A | 11 | 0.026128266 |
| ACSS3 | 11 | 0.026128266 |
| PIAS3 | 11 | 0.026128266 |
| TTC30A | 11 | 0.026128266 |
| LRRFIP1 | 11 | 0.026128266 |
| SLC12A3 | 11 | 0.026128266 |
| ARHGAP45 | 11 | 0.026128266 |
| UGT3A1 | 11 | 0.026128266 |
| PARVB | 11 | 0.026128266 |
| SMPD1 | 11 | 0.026128266 |
| SLC49A3 | 11 | 0.026128266 |
| RRP1B | 11 | 0.026128266 |
| PITPNM2 | 11 | 0.026128266 |
| FRMPD2 | 11 | 0.026128266 |
| ARMCX1 | 11 | 0.026128266 |
| CYP4Z1 | 11 | 0.026128266 |
| TRIM35 | 11 | 0.026128266 |
| CTCFL | 11 | 0.026128266 |
| PHF21A | 11 | 0.026128266 |
| IQUB | 11 | 0.026128266 |
| MELK | 11 | 0.026128266 |
| ARNT2 | 11 | 0.026128266 |
| MTF2 | 11 | 0.026128266 |
| ZMYND11 | 11 | 0.026128266 |
| POLG | 11 | 0.026128266 |
| MAK | 11 | 0.026128266 |
| LRIG3 | 11 | 0.026128266 |
| SLCO4A1 | 11 | 0.026128266 |
| MYEF2 | 11 | 0.026128266 |
| PAMR1 | 11 | 0.026128266 |
| SEC14L5 | 11 | 0.026128266 |
| HERC4 | 11 | 0.026128266 |
| THRAP3 | 11 | 0.026128266 |
| SYN3 | 11 | 0.026128266 |
| ATXN7 | 11 | 0.026128266 |
| FCHSD1 | 11 | 0.026128266 |
| CCDC14 | 11 | 0.026128266 |
| KRT82 | 11 | 0.026128266 |
| ZNF559 | 11 | 0.026128266 |
| CPEB2 | 11 | 0.026128266 |
| MASP2 | 11 | 0.026128266 |
| PRSS35 | 11 | 0.026128266 |
| HTATSF1 | 11 | 0.026128266 |
| PRICKLE1 | 11 | 0.026128266 |
| PARP4 | 11 | 0.026128266 |
| ARHGEF5 | 11 | 0.026128266 |
| IQCH | 11 | 0.026128266 |
| PNMA8A | 11 | 0.026128266 |
| CNNM2 | 11 | 0.026128266 |
| CUL3 | 11 | 0.026128266 |
| FAM13C | 11 | 0.026128266 |
| CRNKL1 | 11 | 0.026128266 |
| EDRF1 | 11 | 0.026128266 |
| USP6NL | 11 | 0.026128266 |
| PROM1 | 11 | 0.026128266 |
| CEP63 | 11 | 0.026128266 |
| EFNB3 | 11 | 0.026128266 |
| PRSS12 | 11 | 0.026128266 |
| FUBP1 | 11 | 0.026128266 |
| NT5C1B | 11 | 0.026128266 |
| ANKRD28 | 11 | 0.026128266 |
| CCR3 | 11 | 0.026128266 |
| FRMD3 | 11 | 0.026128266 |
| ZNF507 | 11 | 0.026128266 |
| OR4N2 | 11 | 0.026128266 |
| ZNF878 | 11 | 0.026128266 |
| KRT33A | 11 | 0.026128266 |
| C12orf40 | 11 | 0.026128266 |
| TEX11 | 11 | 0.026128266 |
| SH3RF3 | 11 | 0.026128266 |
| SIX5 | 11 | 0.026128266 |
| TMPRSS11D | 11 | 0.026128266 |
| SMYD1 | 11 | 0.026128266 |
| OR4M1 | 11 | 0.026128266 |
| PHF23 | 11 | 0.026128266 |
| ZNF320 | 11 | 0.026128266 |
| ZNF74 | 11 | 0.026128266 |
| STK36 | 11 | 0.026128266 |
| RBBP8 | 11 | 0.026128266 |
| DPP4 | 11 | 0.026128266 |
| MBD4 | 11 | 0.026128266 |
| ZNF93 | 11 | 0.026128266 |
| DPYS | 11 | 0.026128266 |
| AMIGO3 | 11 | 0.026128266 |
| MACO1 | 11 | 0.026128266 |
| FARP2 | 11 | 0.026128266 |
| CCNT1 | 11 | 0.026128266 |
| EHMT1 | 11 | 0.026128266 |
| DDX24 | 11 | 0.026128266 |
| PLOD2 | 11 | 0.026128266 |
| USP32 | 11 | 0.026128266 |
| SLAIN1 | 11 | 0.026128266 |
| WDR36 | 11 | 0.026128266 |
| JMJD7-PLA2G4B | 11 | 0.026128266 |
| CDK11B | 11 | 0.026128266 |
| CDH17 | 11 | 0.026128266 |
| C20orf194 | 11 | 0.026128266 |
| AP1M1 | 11 | 0.026128266 |
| GNG12 | 11 | 0.026128266 |
| ZNF653 | 11 | 0.026128266 |
| DISP2 | 11 | 0.026128266 |
| TFAP2C | 11 | 0.026128266 |
| MYRIP | 11 | 0.026128266 |
| ZNF746 | 11 | 0.026128266 |
| CLEC4F | 11 | 0.026128266 |
| ITPRID2 | 11 | 0.026128266 |
| GIMAP1 | 11 | 0.026128266 |
| UTP6 | 11 | 0.026128266 |
| NIM1K | 11 | 0.026128266 |
| RGS9 | 11 | 0.026128266 |
| JADE2 | 11 | 0.026128266 |
| S1PR4 | 11 | 0.026128266 |
| ESPNL | 11 | 0.026128266 |
| MEPCE | 11 | 0.026128266 |
| PRKD2 | 11 | 0.026128266 |
| EGFR | 11 | 0.026128266 |
| SMCR8 | 11 | 0.026128266 |
| OR4K5 | 11 | 0.026128266 |
| FADS2 | 11 | 0.026128266 |
| CCDC186 | 11 | 0.026128266 |
| CCDC150 | 11 | 0.026128266 |
| NEUROD6 | 11 | 0.026128266 |
| RXRA | 11 | 0.026128266 |
| EPS15 | 11 | 0.026128266 |
| C7orf31 | 11 | 0.026128266 |
| NFE2 | 11 | 0.026128266 |
| IFT88 | 11 | 0.026128266 |
| ZNF282 | 11 | 0.026128266 |
| ZNF668 | 11 | 0.026128266 |
| KCNG4 | 11 | 0.026128266 |
| R3HDM2 | 11 | 0.026128266 |
| SV2B | 11 | 0.026128266 |
| SLC9A1 | 11 | 0.026128266 |
| ZNF560 | 11 | 0.026128266 |
| NDN | 11 | 0.026128266 |
| JAM3 | 11 | 0.026128266 |
| TDRD7 | 11 | 0.026128266 |
| RB1 | 11 | 0.026128266 |
| SCFD1 | 11 | 0.026128266 |
| RANBP10 | 11 | 0.026128266 |
| DEPDC1 | 11 | 0.026128266 |
| TACR3 | 11 | 0.026128266 |
| ZYG11B | 11 | 0.026128266 |
| ZNF791 | 11 | 0.026128266 |
| NKAPD1 | 11 | 0.026128266 |
| DACT1 | 11 | 0.026128266 |
| UBQLN3 | 11 | 0.026128266 |
| MOCOS | 11 | 0.026128266 |
| MRGPRX3 | 11 | 0.026128266 |
| FBXL20 | 11 | 0.026128266 |
| ZER1 | 11 | 0.026128266 |
| OR2W1 | 11 | 0.026128266 |
| MARCHF4 | 11 | 0.026128266 |
| CGN | 11 | 0.026128266 |
| FOXO4 | 11 | 0.026128266 |
| KAT2B | 11 | 0.026128266 |
| GAD1 | 11 | 0.026128266 |
| OPRD1 | 11 | 0.026128266 |
| IFT122 | 11 | 0.026128266 |
| PSPC1 | 11 | 0.026128266 |
| STAG2 | 11 | 0.026128266 |
| TRAP1 | 11 | 0.026128266 |
| NAALADL2 | 11 | 0.026128266 |
| CEL | 11 | 0.026128266 |
| ZC3H3 | 11 | 0.026128266 |
| ALDH1A2 | 11 | 0.026128266 |
| DPYSL5 | 11 | 0.026128266 |
| SMPD4 | 11 | 0.026128266 |
| DCLK3 | 11 | 0.026128266 |
| KIF15 | 11 | 0.026128266 |
| TMEM60 | 11 | 0.026128266 |
| OR52A5 | 11 | 0.026128266 |
| MIGA1 | 11 | 0.026128266 |
| PLPPR5 | 11 | 0.026128266 |
| BCO2 | 11 | 0.026128266 |
| NLRP12 | 11 | 0.026128266 |
| SLC44A4 | 11 | 0.026128266 |
| ZNF107 | 11 | 0.026128266 |
| OTUD6A | 11 | 0.026128266 |
| ENG | 11 | 0.026128266 |
| MEIOC | 11 | 0.026128266 |
| RHBDF2 | 11 | 0.026128266 |
| NLGN4Y | 11 | 0.026128266 |
| TTC12 | 11 | 0.026128266 |
| ZNF543 | 11 | 0.026128266 |
| PRRT2 | 11 | 0.026128266 |
| GNPTAB | 11 | 0.026128266 |
| SLC2A14 | 11 | 0.026128266 |
| FBXO11 | 11 | 0.026128266 |
| AGGF1 | 11 | 0.026128266 |
| PIP4K2A | 11 | 0.026128266 |
| MYBPC1 | 11 | 0.026128266 |
| U2SURP | 11 | 0.026128266 |
| DAB2 | 11 | 0.026128266 |
| ADAD1 | 11 | 0.026128266 |
| SCAP | 11 | 0.026128266 |
| CSNK2A2 | 11 | 0.026128266 |
| ERCC3 | 11 | 0.026128266 |
| SLC45A4 | 11 | 0.026128266 |
| OR9Q2 | 11 | 0.026128266 |
| BICD2 | 11 | 0.026128266 |
| RUBCN | 11 | 0.026128266 |
| USP54 | 11 | 0.026128266 |
| SPECC1L | 11 | 0.026128266 |
| ZRANB1 | 11 | 0.026128266 |
| THSD4 | 11 | 0.026128266 |
| SLC18A2 | 11 | 0.026128266 |
| GAS2L1 | 11 | 0.026128266 |
| CCDC136 | 11 | 0.026128266 |
| CSE1L | 11 | 0.026128266 |
| MRTFB | 11 | 0.026128266 |
| ATCAY | 11 | 0.026128266 |
| RBM26 | 11 | 0.026128266 |
| SUCO | 11 | 0.026128266 |
| ZNF608 | 11 | 0.026128266 |
| ECEL1 | 11 | 0.026128266 |
| VWA5A | 11 | 0.026128266 |
| UMOD | 11 | 0.026128266 |
| CFAP74 | 11 | 0.026128266 |
| ZC3H11A | 11 | 0.026128266 |
| ZNF382 | 11 | 0.026128266 |
| ZBTB5 | 11 | 0.026128266 |
| PRORP | 11 | 0.026128266 |
| OR2B2 | 11 | 0.026128266 |
| BMPER | 11 | 0.026128266 |
| OR8H1 | 11 | 0.026128266 |
| TCF7 | 11 | 0.026128266 |
| CDC5L | 11 | 0.026128266 |
| ANKK1 | 11 | 0.026128266 |
| ZNF273 | 11 | 0.026128266 |
| VWCE | 11 | 0.026128266 |
| PDE10A | 11 | 0.026128266 |
| CUL4B | 11 | 0.026128266 |
| MFN1 | 11 | 0.026128266 |
| CYP4F12 | 11 | 0.026128266 |
| PCDH1 | 11 | 0.026128266 |
| TESPA1 | 11 | 0.026128266 |
| FGD4 | 11 | 0.026128266 |
| NVL | 11 | 0.026128266 |
| FLII | 11 | 0.026128266 |
| FTO | 11 | 0.026128266 |
| PROM2 | 11 | 0.026128266 |
| LRCH2 | 11 | 0.026128266 |
| ZKSCAN7 | 11 | 0.026128266 |
| ZNF83 | 11 | 0.026128266 |
| LIG4 | 11 | 0.026128266 |
| USP5 | 11 | 0.026128266 |
| QRFPR | 11 | 0.026128266 |
| ARSB | 11 | 0.026128266 |
| HK2 | 11 | 0.026128266 |
| HID1 | 11 | 0.026128266 |
| CCDC62 | 11 | 0.026128266 |
| ATP2B1 | 11 | 0.026128266 |
| GBP6 | 11 | 0.026128266 |
| PLAGL2 | 11 | 0.026128266 |
| RPE65 | 11 | 0.026128266 |
| TEX47 | 11 | 0.026128266 |
| AADACL3 | 11 | 0.026128266 |
| SLC22A16 | 11 | 0.026128266 |
| ARHGAP6 | 11 | 0.026128266 |
| OSBPL3 | 11 | 0.026128266 |
| GYS2 | 11 | 0.026128266 |
| GPR32 | 11 | 0.026128266 |
| TBC1D31 | 11 | 0.026128266 |
| MUSK | 11 | 0.026128266 |
| WDR26 | 11 | 0.026128266 |
| EPB41L2 | 11 | 0.026128266 |
| ORC1 | 11 | 0.026128266 |
| CCDC160 | 11 | 0.026128266 |
| ELL2 | 11 | 0.026128266 |
| RNF145 | 11 | 0.026128266 |
| TRIM2 | 11 | 0.026128266 |
| FEZF2 | 11 | 0.026128266 |
| ZBTB22 | 11 | 0.026128266 |
| RNF180 | 11 | 0.026128266 |
| PCCA | 11 | 0.026128266 |
| KIF6 | 11 | 0.026128266 |
| PAX5 | 11 | 0.026128266 |
| DEUP1 | 11 | 0.026128266 |
| ZNF777 | 11 | 0.026128266 |
| AHRR | 11 | 0.026128266 |
| EPS8L2 | 11 | 0.026128266 |
| AOC3 | 11 | 0.026128266 |
| IGSF8 | 11 | 0.026128266 |
| DGKA | 11 | 0.026128266 |
| AL121899.2 | 11 | 0.026128266 |
| GTDC1 | 11 | 0.026128266 |
| SOX10 | 11 | 0.026128266 |
| SEMA6C | 11 | 0.026128266 |
| PYGM | 11 | 0.026128266 |
| SPZ1 | 11 | 0.026128266 |
| LDLRAP1 | 11 | 0.026128266 |
| VPS16 | 11 | 0.026128266 |
| ZNF532 | 11 | 0.026128266 |
| CHTF18 | 11 | 0.026128266 |
| QARS1 | 11 | 0.026128266 |
| PRMT2 | 11 | 0.026128266 |
| GASK1B | 11 | 0.026128266 |
| TMEM74 | 11 | 0.026128266 |
| SLC30A9 | 11 | 0.026128266 |
| SPPL2C | 11 | 0.026128266 |
| PARP6 | 11 | 0.026128266 |
| ZNF425 | 11 | 0.026128266 |
| ZNF345 | 11 | 0.026128266 |
| BPIFB6 | 11 | 0.026128266 |
| CUL2 | 11 | 0.026128266 |
| SERAC1 | 11 | 0.026128266 |
| TASOR | 11 | 0.026128266 |
| KANK4 | 11 | 0.026128266 |
| ZSWIM5 | 11 | 0.026128266 |
| SEMA4D | 11 | 0.026128266 |
| EXO1 | 11 | 0.026128266 |
| CDHR5 | 11 | 0.026128266 |
| PANK4 | 11 | 0.026128266 |
| ELAVL4 | 11 | 0.026128266 |
| TJP3 | 11 | 0.026128266 |
| SLC4A4 | 11 | 0.026128266 |
| ZBTB21 | 11 | 0.026128266 |
| HUNK | 11 | 0.026128266 |
| HERC6 | 11 | 0.026128266 |
| AXIN1 | 11 | 0.026128266 |
| EIF2A | 11 | 0.026128266 |
| IKBKE | 11 | 0.026128266 |
| PML | 11 | 0.026128266 |
| AGAP3 | 11 | 0.026128266 |
| SELE | 11 | 0.026128266 |
| SLC22A7 | 11 | 0.026128266 |
| OR2T33 | 11 | 0.026128266 |
| SLC9A4 | 11 | 0.026128266 |
| ZNF112 | 11 | 0.026128266 |
| MYO5B | 11 | 0.026128266 |
| IARS1 | 11 | 0.026128266 |
| ZNF180 | 11 | 0.026128266 |
| KANK2 | 11 | 0.026128266 |
| ANKRD13A | 11 | 0.026128266 |
| SLC16A7 | 11 | 0.026128266 |
| DZANK1 | 11 | 0.026128266 |
| USP2 | 11 | 0.026128266 |
| ARID5B | 11 | 0.026128266 |
| SPTY2D1 | 11 | 0.026128266 |
| TAS1R1 | 11 | 0.026128266 |
| STAU2 | 11 | 0.026128266 |
| PNLDC1 | 11 | 0.026128266 |
| DNAI1 | 11 | 0.026128266 |
| RBAK | 11 | 0.026128266 |
| AP2B1 | 11 | 0.026128266 |
| KLHL22 | 11 | 0.026128266 |
| SNX19 | 11 | 0.026128266 |
| YTHDF3 | 11 | 0.026128266 |
| PLCB2 | 11 | 0.026128266 |
| BAZ1B | 11 | 0.026128266 |
| BPIFB2 | 11 | 0.026128266 |
| TBC1D17 | 11 | 0.026128266 |
| IQCJ-SCHIP1 | 11 | 0.026128266 |
| TLE3 | 11 | 0.026128266 |
| FUT1 | 11 | 0.026128266 |
| BARHL2 | 11 | 0.026128266 |
| HROB | 11 | 0.026128266 |
| OR5W2 | 11 | 0.026128266 |
| CLASRP | 11 | 0.026128266 |
| C10orf71 | 11 | 0.026128266 |
| LRP3 | 11 | 0.026128266 |
| GPR26 | 11 | 0.026128266 |
| DYNC1I2 | 11 | 0.026128266 |
| IRF2 | 11 | 0.026128266 |
| SENP5 | 11 | 0.026128266 |
| ZNF610 | 11 | 0.026128266 |
| GIT2 | 11 | 0.026128266 |
| MEGF10 | 11 | 0.026128266 |
| ARHGEF12 | 11 | 0.026128266 |
| MAGEA12 | 11 | 0.026128266 |
| TPCN1 | 11 | 0.026128266 |
| AQR | 11 | 0.026128266 |
| PPP4R1 | 11 | 0.026128266 |
| CDC25B | 11 | 0.026128266 |
| PNMA8B | 11 | 0.026128266 |
| PRPF3 | 11 | 0.026128266 |
| MMP12 | 11 | 0.026128266 |
| AGO2 | 11 | 0.026128266 |
| OR4A16 | 11 | 0.026128266 |
| ARHGEF7 | 11 | 0.026128266 |
| PRKCI | 11 | 0.026128266 |
| SEC24D | 11 | 0.026128266 |
| HECA | 11 | 0.026128266 |
| GPC5 | 11 | 0.026128266 |
| FNDC3A | 11 | 0.026128266 |
| OR4K1 | 11 | 0.026128266 |
| ZNF135 | 11 | 0.026128266 |
| ZNF699 | 11 | 0.026128266 |
| USP25 | 11 | 0.026128266 |
| NHLRC3 | 11 | 0.026128266 |
| RAPGEF3 | 11 | 0.026128266 |
| COL9A2 | 11 | 0.026128266 |
| MSL3 | 11 | 0.026128266 |
| SNX33 | 11 | 0.026128266 |
| NONO | 11 | 0.026128266 |
| INTS13 | 11 | 0.026128266 |
| TRIM17 | 11 | 0.026128266 |
| L3MBTL4 | 11 | 0.026128266 |
| CLIP3 | 11 | 0.026128266 |
| CYP11B1 | 11 | 0.026128266 |
| SPIDR | 11 | 0.026128266 |
| GPR19 | 11 | 0.026128266 |
| HK1 | 11 | 0.026128266 |
| WDR64 | 11 | 0.026128266 |
| RNF139 | 11 | 0.026128266 |
| TCOF1 | 11 | 0.026128266 |
| TMOD1 | 11 | 0.026128266 |
| ZNF611 | 11 | 0.026128266 |
| PPM1E | 11 | 0.026128266 |
| PAQR9 | 11 | 0.026128266 |
| SERPINB2 | 11 | 0.026128266 |
| KIF9 | 11 | 0.026128266 |
| ZNF572 | 11 | 0.026128266 |
| NFIB | 11 | 0.026128266 |
| ZNF429 | 11 | 0.026128266 |
| RBMXL2 | 11 | 0.026128266 |
| FAM160A2 | 11 | 0.026128266 |
| ACSBG1 | 11 | 0.026128266 |
| PLD2 | 11 | 0.026128266 |
| MSN | 11 | 0.026128266 |
| CYFIP1 | 11 | 0.026128266 |
| DTNA | 11 | 0.026128266 |
| ATL1 | 11 | 0.026128266 |
| RUNX2 | 11 | 0.026128266 |
| KCNF1 | 11 | 0.026128266 |
| ZNF517 | 11 | 0.026128266 |
| YTHDF2 | 11 | 0.026128266 |
| FAP | 11 | 0.026128266 |
| TRIM22 | 11 | 0.026128266 |
| CUL5 | 11 | 0.026128266 |
| DCLK2 | 11 | 0.026128266 |
| WWC2 | 11 | 0.026128266 |
| TOP1 | 11 | 0.026128266 |
| MATR3 | 11 | 0.026128266 |
| EIF4B | 11 | 0.026128266 |
| LCP2 | 11 | 0.026128266 |
| ZNF337 | 11 | 0.026128266 |
| SLC24A1 | 11 | 0.026128266 |
| GRIPAP1 | 11 | 0.026128266 |
| ZNF33A | 11 | 0.026128266 |
| C8orf34 | 11 | 0.026128266 |
| ATP2C1 | 11 | 0.026128266 |
| POMT1 | 11 | 0.026128266 |
| SGCZ | 11 | 0.026128266 |
| VANGL1 | 11 | 0.026128266 |
| APCDD1L | 11 | 0.026128266 |
| TBL1XR1 | 11 | 0.026128266 |
| TAS1R3 | 11 | 0.026128266 |
| TMCO3 | 11 | 0.026128266 |
| FANCI | 11 | 0.026128266 |
| GAD2 | 11 | 0.026128266 |
| SLC4A9 | 11 | 0.026128266 |
| CYP7A1 | 11 | 0.026128266 |
| ABCC2 | 11 | 0.026128266 |
| SIK3 | 11 | 0.026128266 |
| IPO5 | 11 | 0.026128266 |
| NELFE | 11 | 0.026128266 |
| IRX6 | 11 | 0.026128266 |
| PLCH2 | 11 | 0.026128266 |
| PDE8A | 11 | 0.026128266 |
| CDCA7L | 11 | 0.026128266 |
| CRYBG2 | 11 | 0.026128266 |
| PADI1 | 11 | 0.026128266 |
| STK32B | 11 | 0.026128266 |
| FBXO24 | 11 | 0.026128266 |
| ZNF709 | 11 | 0.026128266 |
| NSUN2 | 11 | 0.026128266 |
| POLH | 11 | 0.026128266 |
| NDUFA10 | 11 | 0.026128266 |
| AKNAD1 | 11 | 0.026128266 |
| CLEC14A | 11 | 0.026128266 |
| ATP2C2 | 11 | 0.026128266 |
| OR5H14 | 11 | 0.026128266 |
| UBE2O | 11 | 0.026128266 |
| MAEL | 11 | 0.026128266 |
| LUZP2 | 11 | 0.026128266 |
| DNAAF1 | 11 | 0.026128266 |
| SHLD2 | 11 | 0.026128266 |
| HAS1 | 11 | 0.026128266 |
| CCBE1 | 11 | 0.026128266 |
| OCRL | 11 | 0.026128266 |
| STPG2 | 11 | 0.026128266 |
| KIF12 | 11 | 0.026128266 |
| FMO3 | 11 | 0.026128266 |
| UBXN4 | 11 | 0.026128266 |
| ASPH | 11 | 0.026128266 |
| SLC4A1AP | 10 | 0.023752969 |
| ZNF585B | 10 | 0.023752969 |
| SLC15A2 | 10 | 0.023752969 |
| TRIM29 | 10 | 0.023752969 |
| PANK1 | 10 | 0.023752969 |
| IGSF22 | 10 | 0.023752969 |
| C4orf50 | 10 | 0.023752969 |
| CYP4A22 | 10 | 0.023752969 |
| CAPN6 | 10 | 0.023752969 |
| LILRA5 | 10 | 0.023752969 |
| CACNG3 | 10 | 0.023752969 |
| CDC7 | 10 | 0.023752969 |
| SGCD | 10 | 0.023752969 |
| INTS4 | 10 | 0.023752969 |
| POLA1 | 10 | 0.023752969 |
| CLGN | 10 | 0.023752969 |
| ZNF257 | 10 | 0.023752969 |
| SUPT20H | 10 | 0.023752969 |
| FCAMR | 10 | 0.023752969 |
| PLXDC2 | 10 | 0.023752969 |
| TTC16 | 10 | 0.023752969 |
| B3GAT1 | 10 | 0.023752969 |
| FAM222B | 10 | 0.023752969 |
| TTC29 | 10 | 0.023752969 |
| ADAM32 | 10 | 0.023752969 |
| RGS14 | 10 | 0.023752969 |
| SLC6A3 | 10 | 0.023752969 |
| UGDH | 10 | 0.023752969 |
| PURG | 10 | 0.023752969 |
| FBLN1 | 10 | 0.023752969 |
| TUBB4A | 10 | 0.023752969 |
| CDC73 | 10 | 0.023752969 |
| RTCB | 10 | 0.023752969 |
| HIF1A | 10 | 0.023752969 |
| PRDM4 | 10 | 0.023752969 |
| WDR55 | 10 | 0.023752969 |
| GOLGA2 | 10 | 0.023752969 |
| P2RY4 | 10 | 0.023752969 |
| BRSK2 | 10 | 0.023752969 |
| NOX3 | 10 | 0.023752969 |
| PNPT1 | 10 | 0.023752969 |
| GP5 | 10 | 0.023752969 |
| ING1 | 10 | 0.023752969 |
| ART5 | 10 | 0.023752969 |
| ALAS2 | 10 | 0.023752969 |
| DOK7 | 10 | 0.023752969 |
| XPO7 | 10 | 0.023752969 |
| SPAG16 | 10 | 0.023752969 |
| POT1 | 10 | 0.023752969 |
| ECT2 | 10 | 0.023752969 |
| FAM170A | 10 | 0.023752969 |
| GK2 | 10 | 0.023752969 |
| HCRTR2 | 10 | 0.023752969 |
| G2E3 | 10 | 0.023752969 |
| ALG10 | 10 | 0.023752969 |
| KLHL11 | 10 | 0.023752969 |
| PLTP | 10 | 0.023752969 |
| SAMHD1 | 10 | 0.023752969 |
| PRR23B | 10 | 0.023752969 |
| TLR10 | 10 | 0.023752969 |
| PAPOLA | 10 | 0.023752969 |
| WDR5 | 10 | 0.023752969 |
| PTGS2 | 10 | 0.023752969 |
| ADGRA1 | 10 | 0.023752969 |
| TENT4B | 10 | 0.023752969 |
| MLLT1 | 10 | 0.023752969 |
| COG6 | 10 | 0.023752969 |
| IRX5 | 10 | 0.023752969 |
| CFAP91 | 10 | 0.023752969 |
| ZNF473 | 10 | 0.023752969 |
| OPRM1 | 10 | 0.023752969 |
| CPT1B | 10 | 0.023752969 |
| ACOT12 | 10 | 0.023752969 |
| ZCCHC8 | 10 | 0.023752969 |
| LMCD1 | 10 | 0.023752969 |
| TOX | 10 | 0.023752969 |
| FAM81B | 10 | 0.023752969 |
| ACTN4 | 10 | 0.023752969 |
| MMRN2 | 10 | 0.023752969 |
| C7 | 10 | 0.023752969 |
| LRRC4B | 10 | 0.023752969 |
| CASK | 10 | 0.023752969 |
| PEX5L | 10 | 0.023752969 |
| CCDC148 | 10 | 0.023752969 |
| TBK1 | 10 | 0.023752969 |
| MYO1H | 10 | 0.023752969 |
| CHST3 | 10 | 0.023752969 |
| AMER3 | 10 | 0.023752969 |
| OR4K15 | 10 | 0.023752969 |
| RBL1 | 10 | 0.023752969 |
| MGRN1 | 10 | 0.023752969 |
| KLHL10 | 10 | 0.023752969 |
| IKZF1 | 10 | 0.023752969 |
| HR | 10 | 0.023752969 |
| NGLY1 | 10 | 0.023752969 |
| FLRT3 | 10 | 0.023752969 |
| C12orf42 | 10 | 0.023752969 |
| BRCA1 | 10 | 0.023752969 |
| STON2 | 10 | 0.023752969 |
| SEC24C | 10 | 0.023752969 |
| PTPN1 | 10 | 0.023752969 |
| COG1 | 10 | 0.023752969 |
| SLC22A6 | 10 | 0.023752969 |
| RANBP6 | 10 | 0.023752969 |
| ZNF597 | 10 | 0.023752969 |
| CPSF2 | 10 | 0.023752969 |
| PBXIP1 | 10 | 0.023752969 |
| ZFP42 | 10 | 0.023752969 |
| CCNL1 | 10 | 0.023752969 |
| ARSF | 10 | 0.023752969 |
| OR10AG1 | 10 | 0.023752969 |
| CPB2 | 10 | 0.023752969 |
| TUBB8 | 10 | 0.023752969 |
| TBR1 | 10 | 0.023752969 |
| RAB3C | 10 | 0.023752969 |
| BMP7 | 10 | 0.023752969 |
| DDX46 | 10 | 0.023752969 |
| POM121 | 10 | 0.023752969 |
| HEPACAM2 | 10 | 0.023752969 |
| WIPF3 | 10 | 0.023752969 |
| ZFP30 | 10 | 0.023752969 |
| DSC2 | 10 | 0.023752969 |
| TXNRD1 | 10 | 0.023752969 |
| DHX37 | 10 | 0.023752969 |
| LRIG2 | 10 | 0.023752969 |
| EPHA8 | 10 | 0.023752969 |
| PIBF1 | 10 | 0.023752969 |
| EXTL3 | 10 | 0.023752969 |
| ELF3 | 10 | 0.023752969 |
| TRAF2 | 10 | 0.023752969 |
| CCDC170 | 10 | 0.023752969 |
| LRIT1 | 10 | 0.023752969 |
| SF3B4 | 10 | 0.023752969 |
| ASB15 | 10 | 0.023752969 |
| USP33 | 10 | 0.023752969 |
| KIAA0753 | 10 | 0.023752969 |
| ZNF692 | 10 | 0.023752969 |
| GAB4 | 10 | 0.023752969 |
| ACTR1B | 10 | 0.023752969 |
| ZNF384 | 10 | 0.023752969 |
| ZNF513 | 10 | 0.023752969 |
| SPAG1 | 10 | 0.023752969 |
| KHDRBS2 | 10 | 0.023752969 |
| SLC39A12 | 10 | 0.023752969 |
| GJB3 | 10 | 0.023752969 |
| DAGLA | 10 | 0.023752969 |
| CAMK2G | 10 | 0.023752969 |
| ANOS1 | 10 | 0.023752969 |
| EML3 | 10 | 0.023752969 |
| ODF2 | 10 | 0.023752969 |
| CD96 | 10 | 0.023752969 |
| VWA3A | 10 | 0.023752969 |
| PUM2 | 10 | 0.023752969 |
| RNF103 | 10 | 0.023752969 |
| NR2C2 | 10 | 0.023752969 |
| POTEA | 10 | 0.023752969 |
| CEP131 | 10 | 0.023752969 |
| ELOA | 10 | 0.023752969 |
| FOXM1 | 10 | 0.023752969 |
| TSHR | 10 | 0.023752969 |
| ZNF385A | 10 | 0.023752969 |
| RPS6KA1 | 10 | 0.023752969 |
| IL1RL2 | 10 | 0.023752969 |
| KIF5A | 10 | 0.023752969 |
| SLC1A3 | 10 | 0.023752969 |
| ZNF417 | 10 | 0.023752969 |
| KRT222 | 10 | 0.023752969 |
| ZNF117 | 10 | 0.023752969 |
| TRPV1 | 10 | 0.023752969 |
| DPYSL2 | 10 | 0.023752969 |
| LMOD1 | 10 | 0.023752969 |
| RNF150 | 10 | 0.023752969 |
| TSPYL6 | 10 | 0.023752969 |
| ZNF44 | 10 | 0.023752969 |
| ME2 | 10 | 0.023752969 |
| SERPINB8 | 10 | 0.023752969 |
| BZW1 | 10 | 0.023752969 |
| NHSL2 | 10 | 0.023752969 |
| SH3BP2 | 10 | 0.023752969 |
| LTF | 10 | 0.023752969 |
| HDAC7 | 10 | 0.023752969 |
| KRT84 | 10 | 0.023752969 |
| ELAVL3 | 10 | 0.023752969 |
| ZNF700 | 10 | 0.023752969 |
| MMP2 | 10 | 0.023752969 |
| GFM1 | 10 | 0.023752969 |
| ZNF133 | 10 | 0.023752969 |
| SPATA20 | 10 | 0.023752969 |
| TENT2 | 10 | 0.023752969 |
| ADAM19 | 10 | 0.023752969 |
| DMGDH | 10 | 0.023752969 |
| GNB1L | 10 | 0.023752969 |
| ZNF790 | 10 | 0.023752969 |
| ZMAT1 | 10 | 0.023752969 |
| SYT5 | 10 | 0.023752969 |
| ZDHHC14 | 10 | 0.023752969 |
| MATN4 | 10 | 0.023752969 |
| OR2T6 | 10 | 0.023752969 |
| CHST9 | 10 | 0.023752969 |
| HS6ST2 | 10 | 0.023752969 |
| ZNF484 | 10 | 0.023752969 |
| LIPI | 10 | 0.023752969 |
| ADGRE3 | 10 | 0.023752969 |
| F13B | 10 | 0.023752969 |
| PWWP3A | 10 | 0.023752969 |
| ERICH1 | 10 | 0.023752969 |
| SDSL | 10 | 0.023752969 |
| ALDH16A1 | 10 | 0.023752969 |
| RTN3 | 10 | 0.023752969 |
| ANKRD6 | 10 | 0.023752969 |
| ITGA2B | 10 | 0.023752969 |
| DAXX | 10 | 0.023752969 |
| OR11H1 | 10 | 0.023752969 |
| INO80E | 10 | 0.023752969 |
| MPHOSPH10 | 10 | 0.023752969 |
| CPLANE1 | 10 | 0.023752969 |
| ZNF234 | 10 | 0.023752969 |
| KIF27 | 10 | 0.023752969 |
| ALDH3A1 | 10 | 0.023752969 |
| MIGA2 | 10 | 0.023752969 |
| TTC39A | 10 | 0.023752969 |
| DRC1 | 10 | 0.023752969 |
| ADAMTS15 | 10 | 0.023752969 |
| PITRM1 | 10 | 0.023752969 |
| USP4 | 10 | 0.023752969 |
| FOXD4L4 | 10 | 0.023752969 |
| MACROH2A2 | 10 | 0.023752969 |
| ZNF212 | 10 | 0.023752969 |
| KRT6C | 10 | 0.023752969 |
| COL23A1 | 10 | 0.023752969 |
| PDIA5 | 10 | 0.023752969 |
| ATP8A1 | 10 | 0.023752969 |
| NR1H3 | 10 | 0.023752969 |
| DNAAF4 | 10 | 0.023752969 |
| UBAP2L | 10 | 0.023752969 |
| ZNF283 | 10 | 0.023752969 |
| HPS1 | 10 | 0.023752969 |
| SLC2A13 | 10 | 0.023752969 |
| VCL | 10 | 0.023752969 |
| BAHD1 | 10 | 0.023752969 |
| HNRNPUL1 | 10 | 0.023752969 |
| WDPCP | 10 | 0.023752969 |
| LUM | 10 | 0.023752969 |
| CDR2 | 10 | 0.023752969 |
| RREB1 | 10 | 0.023752969 |
| OR8J1 | 10 | 0.023752969 |
| SIRPB1 | 10 | 0.023752969 |
| ADAM17 | 10 | 0.023752969 |
| TMEM26 | 10 | 0.023752969 |
| JPH1 | 10 | 0.023752969 |
| INTS6L | 10 | 0.023752969 |
| RABGGTA | 10 | 0.023752969 |
| AMPD3 | 10 | 0.023752969 |
| ATP11B | 10 | 0.023752969 |
| MAN2A2 | 10 | 0.023752969 |
| TPX2 | 10 | 0.023752969 |
| PRKCD | 10 | 0.023752969 |
| CNGA4 | 10 | 0.023752969 |
| PM20D1 | 10 | 0.023752969 |
| ZNF614 | 10 | 0.023752969 |
| CLMP | 10 | 0.023752969 |
| FMO5 | 10 | 0.023752969 |
| FAM71A | 10 | 0.023752969 |
| SRP68 | 10 | 0.023752969 |
| SLC7A10 | 10 | 0.023752969 |
| DIS3 | 10 | 0.023752969 |
| KCNA10 | 10 | 0.023752969 |
| ELF1 | 10 | 0.023752969 |
| GDF6 | 10 | 0.023752969 |
| CBY2 | 10 | 0.023752969 |
| ELF4 | 10 | 0.023752969 |
| SLC43A3 | 10 | 0.023752969 |
| ZNF140 | 10 | 0.023752969 |
| ZNF211 | 10 | 0.023752969 |
| OR4D10 | 10 | 0.023752969 |
| RUFY3 | 10 | 0.023752969 |
| UXS1 | 10 | 0.023752969 |
| VMP1 | 10 | 0.023752969 |
| CSNK1E | 10 | 0.023752969 |
| OR5H6 | 10 | 0.023752969 |
| GPR22 | 10 | 0.023752969 |
| MYO1C | 10 | 0.023752969 |
| LRRFIP2 | 10 | 0.023752969 |
| FASTKD1 | 10 | 0.023752969 |
| TNS4 | 10 | 0.023752969 |
| LCP1 | 10 | 0.023752969 |
| ZNF132 | 10 | 0.023752969 |
| SLC44A1 | 10 | 0.023752969 |
| NDE1 | 10 | 0.023752969 |
| PPFIBP1 | 10 | 0.023752969 |
| IGHE | 10 | 0.023752969 |
| CCDC9 | 10 | 0.023752969 |
| CCDC144A | 10 | 0.023752969 |
| MROH9 | 10 | 0.023752969 |
| SCYL3 | 10 | 0.023752969 |
| MAGI3 | 10 | 0.023752969 |
| TGFB2 | 10 | 0.023752969 |
| SRMS | 10 | 0.023752969 |
| CD2BP2 | 10 | 0.023752969 |
| PPEF1 | 10 | 0.023752969 |
| MAP2K1 | 10 | 0.023752969 |
| SNAPC1 | 10 | 0.023752969 |
| SLC25A13 | 10 | 0.023752969 |
| OR10H1 | 10 | 0.023752969 |
| CSRNP3 | 10 | 0.023752969 |
| PHAX | 10 | 0.023752969 |
| LILRB2 | 10 | 0.023752969 |
| INSC | 10 | 0.023752969 |
| SLCO1B1 | 10 | 0.023752969 |
| TXLNA | 10 | 0.023752969 |
| SLC45A1 | 10 | 0.023752969 |
| CACNB2 | 10 | 0.023752969 |
| SLC15A1 | 10 | 0.023752969 |
| HOMEZ | 10 | 0.023752969 |
| DDX11 | 10 | 0.023752969 |
| GRN | 10 | 0.023752969 |
| MED16 | 10 | 0.023752969 |
| FAM110B | 10 | 0.023752969 |
| PGM5 | 10 | 0.023752969 |
| CLMN | 10 | 0.023752969 |
| FOXD4L5 | 10 | 0.023752969 |
| DHX35 | 10 | 0.023752969 |
| ZNF101 | 10 | 0.023752969 |
| MAGEB6B | 10 | 0.023752969 |
| DYM | 10 | 0.023752969 |
| TYW1 | 10 | 0.023752969 |
| ZFP64 | 10 | 0.023752969 |
| KL | 10 | 0.023752969 |
| TKFC | 10 | 0.023752969 |
| KLHL24 | 10 | 0.023752969 |
| SLC2A7 | 10 | 0.023752969 |
| SLC8B1 | 10 | 0.023752969 |
| AOX1 | 10 | 0.023752969 |
| DOK1 | 10 | 0.023752969 |
| HOOK2 | 10 | 0.023752969 |
| ARC | 10 | 0.023752969 |
| NPFFR2 | 10 | 0.023752969 |
| MTMR1 | 10 | 0.023752969 |
| ZNF600 | 10 | 0.023752969 |
| TM9SF2 | 10 | 0.023752969 |
| NCAN | 10 | 0.023752969 |
| POM121C | 10 | 0.023752969 |
| ALDH1B1 | 10 | 0.023752969 |
| DBT | 10 | 0.023752969 |
| FAM169A | 10 | 0.023752969 |
| PINK1 | 10 | 0.023752969 |
| ADGRE1 | 10 | 0.023752969 |
| ZNF266 | 10 | 0.023752969 |
| OSR2 | 10 | 0.023752969 |
| MAML3 | 10 | 0.023752969 |
| CA6 | 10 | 0.023752969 |
| ZNF131 | 10 | 0.023752969 |
| PPP2R3A | 10 | 0.023752969 |
| OR13C3 | 10 | 0.023752969 |
| ZNF594 | 10 | 0.023752969 |
| HAUS3 | 10 | 0.023752969 |
| FAM160B2 | 10 | 0.023752969 |
| SMARCA5 | 10 | 0.023752969 |
| EHHADH | 10 | 0.023752969 |
| MORC4 | 10 | 0.023752969 |
| NOX4 | 10 | 0.023752969 |
| ZNF676 | 10 | 0.023752969 |
| ZNF467 | 10 | 0.023752969 |
| PIWIL2 | 10 | 0.023752969 |
| OTC | 10 | 0.023752969 |
| PAK4 | 10 | 0.023752969 |
| SREK1 | 10 | 0.023752969 |
| APP | 10 | 0.023752969 |
| VCP | 10 | 0.023752969 |
| POMGNT1 | 10 | 0.023752969 |
| GDF10 | 10 | 0.023752969 |
| NR5A2 | 10 | 0.023752969 |
| GEMIN4 | 10 | 0.023752969 |
| MYCN | 10 | 0.023752969 |
| MAGED2 | 10 | 0.023752969 |
| SHKBP1 | 10 | 0.023752969 |
| RNF214 | 10 | 0.023752969 |
| ZZZ3 | 10 | 0.023752969 |
| FAF1 | 10 | 0.023752969 |
| TCF3 | 10 | 0.023752969 |
| GNAO1 | 10 | 0.023752969 |
| INSM2 | 10 | 0.023752969 |
| VPS53 | 10 | 0.023752969 |
| POU2F1 | 10 | 0.023752969 |
| KRT40 | 10 | 0.023752969 |
| GAREM1 | 10 | 0.023752969 |
| KCNN2 | 10 | 0.023752969 |
| ATP13A1 | 10 | 0.023752969 |
| CBLB | 10 | 0.023752969 |
| DSE | 10 | 0.023752969 |
| FAM83G | 10 | 0.023752969 |
| ARHGAP44 | 10 | 0.023752969 |
| CADM3 | 10 | 0.023752969 |
| KLHL30 | 10 | 0.023752969 |
| ZNF778 | 10 | 0.023752969 |
| MYF6 | 10 | 0.023752969 |
| DAO | 10 | 0.023752969 |
| NEIL1 | 10 | 0.023752969 |
| EXOC2 | 10 | 0.023752969 |
| PAPOLB | 10 | 0.023752969 |
| PDXDC1 | 10 | 0.023752969 |
| PAN3 | 10 | 0.023752969 |
| CTAGE1 | 10 | 0.023752969 |
| HDGF | 10 | 0.023752969 |
| NCSTN | 10 | 0.023752969 |
| FMO1 | 10 | 0.023752969 |
| KIF14 | 10 | 0.023752969 |
| ILF3 | 10 | 0.023752969 |
| ADSL | 10 | 0.023752969 |
| ATN1 | 10 | 0.023752969 |
| PAH | 10 | 0.023752969 |
| PTH2R | 10 | 0.023752969 |
| OR2A5 | 10 | 0.023752969 |
| RINT1 | 10 | 0.023752969 |
| BARD1 | 10 | 0.023752969 |
| ZNF530 | 10 | 0.023752969 |
| GRIN2D | 10 | 0.023752969 |
| RBM42 | 10 | 0.023752969 |
| PMS2 | 10 | 0.023752969 |
| PCDHB16 | 10 | 0.023752969 |
| KRT1 | 10 | 0.023752969 |
| CYP2F1 | 10 | 0.023752969 |
| NCBP1 | 10 | 0.023752969 |
| SLC17A6 | 10 | 0.023752969 |
| TRAPPC11 | 10 | 0.023752969 |
| ZFP36L1 | 10 | 0.023752969 |
| PTPN5 | 10 | 0.023752969 |
| ZNF221 | 10 | 0.023752969 |
| TSPAN5 | 10 | 0.023752969 |
| PIAS1 | 10 | 0.023752969 |
| ZFP82 | 10 | 0.023752969 |
| TNFRSF10A | 10 | 0.023752969 |
| OR6T1 | 10 | 0.023752969 |
| SRPX | 10 | 0.023752969 |
| PDE4B | 10 | 0.023752969 |
| PARP10 | 10 | 0.023752969 |
| ALDH1L2 | 10 | 0.023752969 |
| USP53 | 10 | 0.023752969 |
| PROSER1 | 10 | 0.023752969 |
| MAGED1 | 10 | 0.023752969 |
| PIEZO2 | 10 | 0.023752969 |
| PDE5A | 10 | 0.023752969 |
| ADGRF1 | 10 | 0.023752969 |
| POLI | 10 | 0.023752969 |
| MYLK3 | 10 | 0.023752969 |
| KRT79 | 10 | 0.023752969 |
| FSCN3 | 10 | 0.023752969 |
| ANO7 | 10 | 0.023752969 |
| OR4C6 | 10 | 0.023752969 |
| KCNS3 | 10 | 0.023752969 |
| AJAP1 | 10 | 0.023752969 |
| DPYSL4 | 10 | 0.023752969 |
| PPP1R3F | 10 | 0.023752969 |
| TRIM67 | 10 | 0.023752969 |
| SLC26A8 | 10 | 0.023752969 |
| PTPN4 | 10 | 0.023752969 |
| IKZF4 | 10 | 0.023752969 |
| FCN1 | 10 | 0.023752969 |
| MAN2B1 | 10 | 0.023752969 |
| UST | 10 | 0.023752969 |
| LGR5 | 10 | 0.023752969 |
| PRKG1 | 10 | 0.023752969 |
| DLGAP4 | 10 | 0.023752969 |
| IDH2 | 10 | 0.023752969 |
| KDM7A | 10 | 0.023752969 |
| RAD17 | 10 | 0.023752969 |
| ALPL | 10 | 0.023752969 |
| ASB18 | 10 | 0.023752969 |
| CBL | 10 | 0.023752969 |
| ANKRD55 | 10 | 0.023752969 |
| SNX1 | 10 | 0.023752969 |
| PTPRR | 10 | 0.023752969 |
| KLC1 | 10 | 0.023752969 |
| IL4I1 | 10 | 0.023752969 |
| DSC1 | 10 | 0.023752969 |
| TENT5D | 10 | 0.023752969 |
| PIK3R2 | 10 | 0.023752969 |
| PTPRJ | 10 | 0.023752969 |
| CCNB3 | 10 | 0.023752969 |
| SYTL5 | 10 | 0.023752969 |
| CNOT4 | 10 | 0.023752969 |
| EPHB2 | 10 | 0.023752969 |
| IGFALS | 10 | 0.023752969 |
| KLHL32 | 10 | 0.023752969 |
| CAB39L | 10 | 0.023752969 |
| ANKMY1 | 10 | 0.023752969 |
| ATF6 | 10 | 0.023752969 |
| OSBPL10 | 10 | 0.023752969 |
| SHCBP1L | 10 | 0.023752969 |
| HASPIN | 10 | 0.023752969 |
| DRP2 | 10 | 0.023752969 |
| LMF2 | 10 | 0.023752969 |
| DUSP22 | 10 | 0.023752969 |
| XPNPEP3 | 10 | 0.023752969 |
| ABCB9 | 10 | 0.023752969 |
| CRYGA | 10 | 0.023752969 |
| MDM1 | 10 | 0.023752969 |
| TGFBRAP1 | 10 | 0.023752969 |
| CSNK1G3 | 10 | 0.023752969 |
| FAM91A1 | 10 | 0.023752969 |
| TBX2 | 10 | 0.023752969 |
| CWC27 | 10 | 0.023752969 |
| DYRK1B | 10 | 0.023752969 |
| PON1 | 10 | 0.023752969 |
| SOX13 | 10 | 0.023752969 |
| TEKT3 | 10 | 0.023752969 |
| ALG11 | 10 | 0.023752969 |
| ARHGEF3 | 10 | 0.023752969 |
| PRDM1 | 10 | 0.023752969 |
| RCC2 | 10 | 0.023752969 |
| IL17RA | 10 | 0.023752969 |
| HCLS1 | 10 | 0.023752969 |
| TMEM39A | 10 | 0.023752969 |
| ZNF20 | 10 | 0.023752969 |
| RBM12B | 10 | 0.023752969 |
| USP38 | 10 | 0.023752969 |
| RCOR3 | 10 | 0.023752969 |
| PSMD2 | 10 | 0.023752969 |
| SOBP | 10 | 0.023752969 |
| SPDL1 | 10 | 0.023752969 |
| FNIP2 | 10 | 0.023752969 |
| FRMD6 | 10 | 0.023752969 |
| APC2 | 10 | 0.023752969 |
| SLC22A10 | 10 | 0.023752969 |
| ATP6V0D2 | 10 | 0.023752969 |
| GOLGA5 | 10 | 0.023752969 |
| CAPN7 | 10 | 0.023752969 |
| GCNT2 | 10 | 0.023752969 |
| AHI1 | 10 | 0.023752969 |
| EYA1 | 10 | 0.023752969 |
| ELK3 | 10 | 0.023752969 |
| TRAIP | 10 | 0.023752969 |
| CCDC151 | 10 | 0.023752969 |
| PLCD1 | 10 | 0.023752969 |
| CYP2D6 | 10 | 0.023752969 |
| ANAPC2 | 10 | 0.023752969 |
| OR5K2 | 10 | 0.023752969 |
| SFRP4 | 10 | 0.023752969 |
| ARHGAP9 | 10 | 0.023752969 |
| TLE1 | 10 | 0.023752969 |
| HSPA4L | 10 | 0.023752969 |
| WDR75 | 10 | 0.023752969 |
| KIF20A | 10 | 0.023752969 |
| EPB41L4B | 10 | 0.023752969 |
| PGM1 | 10 | 0.023752969 |
| OR4C13 | 10 | 0.023752969 |
| NTNG1 | 10 | 0.023752969 |
| ZNF354A | 10 | 0.023752969 |
| LEF1 | 10 | 0.023752969 |
| BACH1 | 10 | 0.023752969 |
| ZNF675 | 10 | 0.023752969 |
| APMAP | 10 | 0.023752969 |
| SSBP3 | 10 | 0.023752969 |
| UNK | 10 | 0.023752969 |
| CD101 | 10 | 0.023752969 |
| GRIN1 | 10 | 0.023752969 |
| ZFP57 | 10 | 0.023752969 |
| UGT2A1 | 10 | 0.023752969 |
| TBCK | 10 | 0.023752969 |
| ZNF134 | 10 | 0.023752969 |
| KMO | 10 | 0.023752969 |
| KBTBD3 | 10 | 0.023752969 |
| IL2RG | 10 | 0.023752969 |
| BMP2 | 10 | 0.023752969 |
| ZNF573 | 10 | 0.023752969 |
| CERKL | 10 | 0.023752969 |
| ANKFY1 | 10 | 0.023752969 |
| RBM6 | 10 | 0.023752969 |
| OR51E1 | 10 | 0.023752969 |
| STIM2 | 10 | 0.023752969 |
| DSG2 | 10 | 0.023752969 |
| ANXA13 | 10 | 0.023752969 |
| HACE1 | 10 | 0.023752969 |
| TTC23L | 10 | 0.023752969 |
| ALOXE3 | 10 | 0.023752969 |
| VRK2 | 10 | 0.023752969 |
| STK4 | 10 | 0.023752969 |
| FOXP1 | 10 | 0.023752969 |
| PHACTR4 | 10 | 0.023752969 |
| RNF220 | 10 | 0.023752969 |
| IGHG2 | 10 | 0.023752969 |
| COPB2 | 10 | 0.023752969 |
| EPHB3 | 10 | 0.023752969 |
| TKTL2 | 10 | 0.023752969 |
| SIDT2 | 10 | 0.023752969 |
| SMG7 | 10 | 0.023752969 |
| ESYT1 | 10 | 0.023752969 |
| WDR13 | 10 | 0.023752969 |
| SP3 | 10 | 0.023752969 |
| NUF2 | 10 | 0.023752969 |
| DNAAF3 | 10 | 0.023752969 |
| CD4 | 10 | 0.023752969 |
| FBF1 | 10 | 0.023752969 |
| TIAL1 | 10 | 0.023752969 |
| MYPOP | 10 | 0.023752969 |
| ZNF395 | 10 | 0.023752969 |
| MTMR3 | 10 | 0.023752969 |
| C9 | 10 | 0.023752969 |
| ESRRG | 10 | 0.023752969 |
| EDIL3 | 10 | 0.023752969 |
| PUM3 | 10 | 0.023752969 |
| LARP7 | 10 | 0.023752969 |
| IRAK3 | 10 | 0.023752969 |
| CHSY3 | 10 | 0.023752969 |
| NPHP1 | 10 | 0.023752969 |
| VPS18 | 10 | 0.023752969 |
| RPS6KA4 | 10 | 0.023752969 |
| KLF17 | 10 | 0.023752969 |
| EPHB4 | 10 | 0.023752969 |
| HNRNPUL2 | 10 | 0.023752969 |
| PABPC1 | 10 | 0.023752969 |
| BBS12 | 10 | 0.023752969 |
| CYP2A13 | 10 | 0.023752969 |
| KCTD18 | 10 | 0.023752969 |
| DISC1 | 10 | 0.023752969 |
| MMRN1 | 10 | 0.023752969 |
| STRN3 | 10 | 0.023752969 |
| GPR12 | 10 | 0.023752969 |
| KIZ | 10 | 0.023752969 |
| CCSER2 | 10 | 0.023752969 |
| CCDC120 | 10 | 0.023752969 |
| CPNE8 | 10 | 0.023752969 |
| OR5K3 | 10 | 0.023752969 |
| FARSB | 10 | 0.023752969 |
| ZSCAN1 | 10 | 0.023752969 |
| ST6GALNAC3 | 10 | 0.023752969 |
| PPIP5K2 | 10 | 0.023752969 |
| ADARB2 | 10 | 0.023752969 |
| TFE3 | 10 | 0.023752969 |
| USP20 | 10 | 0.023752969 |
| CCDC173 | 10 | 0.023752969 |
| WDR81 | 10 | 0.023752969 |
| PLEKHA4 | 10 | 0.023752969 |
| ELOVL2 | 10 | 0.023752969 |
| CHRDL1 | 10 | 0.023752969 |
| KLHL36 | 10 | 0.023752969 |
| JRKL | 10 | 0.023752969 |
| WDR45 | 10 | 0.023752969 |
| EFR3A | 10 | 0.023752969 |
| OR2W3 | 10 | 0.023752969 |
| POTEH | 10 | 0.023752969 |
| TRIP6 | 10 | 0.023752969 |
| PEX6 | 10 | 0.023752969 |
| PPM1L | 10 | 0.023752969 |
| OXCT2 | 10 | 0.023752969 |
| PIGG | 10 | 0.023752969 |
| BARHL1 | 10 | 0.023752969 |
| TAB2 | 10 | 0.023752969 |
| PICALM | 10 | 0.023752969 |
| CCDC66 | 10 | 0.023752969 |
| S1PR1 | 10 | 0.023752969 |
| TMC4 | 10 | 0.023752969 |
| ORC3 | 10 | 0.023752969 |
| GLE1 | 10 | 0.023752969 |
| PIWIL4 | 10 | 0.023752969 |
| PRKCSH | 10 | 0.023752969 |
| SAMD4A | 10 | 0.023752969 |
| ARHGAP15 | 10 | 0.023752969 |
| PWWP3B | 10 | 0.023752969 |
| ALPI | 10 | 0.023752969 |
| NIBAN2 | 10 | 0.023752969 |
| BLK | 10 | 0.023752969 |
| LILRB5 | 10 | 0.023752969 |
| ALOX12B | 10 | 0.023752969 |
| SLC9A5 | 10 | 0.023752969 |
| KRT74 | 10 | 0.023752969 |
| RPH3A | 10 | 0.023752969 |
| ATF7IP | 10 | 0.023752969 |
| CDKL5 | 10 | 0.023752969 |
| SLC22A14 | 10 | 0.023752969 |
| POLR1B | 10 | 0.023752969 |
| ASIC4 | 10 | 0.023752969 |
| RPS6KC1 | 10 | 0.023752969 |
| NUB1 | 10 | 0.023752969 |
| SLC1A7 | 10 | 0.023752969 |
| ERCC2 | 10 | 0.023752969 |
| KLRK1 | 10 | 0.023752969 |
| ALDH18A1 | 10 | 0.023752969 |
| EXOC3 | 10 | 0.023752969 |
| SPTLC2 | 10 | 0.023752969 |
| CDC23 | 10 | 0.023752969 |
| CNTROB | 10 | 0.023752969 |
| MUC5AC | 10 | 0.023752969 |
| ARHGEF2 | 10 | 0.023752969 |
| KRT3 | 10 | 0.023752969 |
| RSPH4A | 10 | 0.023752969 |
| PGAP4 | 10 | 0.023752969 |
| TMEM200C | 10 | 0.023752969 |
| SYT3 | 10 | 0.023752969 |
| ZDHHC5 | 10 | 0.023752969 |
| SCLT1 | 10 | 0.023752969 |
| PRRT3 | 10 | 0.023752969 |
| VANGL2 | 10 | 0.023752969 |
| TTC13 | 10 | 0.023752969 |
| OR4C16 | 10 | 0.023752969 |
| PPP2R3B | 10 | 0.023752969 |
| VWA3B | 10 | 0.023752969 |
| FGD3 | 10 | 0.023752969 |
| KLHL23 | 10 | 0.023752969 |
| ZNF214 | 10 | 0.023752969 |
| F2R | 10 | 0.023752969 |
| INVS | 10 | 0.023752969 |
| NR2E1 | 10 | 0.023752969 |
| TRIM56 | 10 | 0.023752969 |
| NOC4L | 10 | 0.023752969 |
| CYP27C1 | 10 | 0.023752969 |
| SLC5A3 | 10 | 0.023752969 |
| IKZF2 | 10 | 0.023752969 |
| PKP2 | 10 | 0.023752969 |
| SP100 | 10 | 0.023752969 |
| TMCC2 | 10 | 0.023752969 |
| CHRNB2 | 10 | 0.023752969 |
| EGF | 10 | 0.023752969 |
| OR52D1 | 10 | 0.023752969 |
| PANX2 | 10 | 0.023752969 |
| CARS1 | 10 | 0.023752969 |
| OR2Y1 | 10 | 0.023752969 |
| NUMB | 10 | 0.023752969 |
| ZNF775 | 10 | 0.023752969 |
| SLC17A5 | 10 | 0.023752969 |
| VPS52 | 10 | 0.023752969 |
| SEC63 | 10 | 0.023752969 |
| NTRK1 | 10 | 0.023752969 |
| XKR7 | 10 | 0.023752969 |
| EHD4 | 10 | 0.023752969 |
| CYP2A7 | 10 | 0.023752969 |
| TTC7A | 10 | 0.023752969 |
| GSK3B | 10 | 0.023752969 |
| ANTXR1 | 10 | 0.023752969 |
| SLC6A13 | 10 | 0.023752969 |
| FUBP3 | 10 | 0.023752969 |
| PAX2 | 10 | 0.023752969 |
| SPRY3 | 10 | 0.023752969 |
| IGHM | 10 | 0.023752969 |
| AP1G2 | 10 | 0.023752969 |
| RBM28 | 10 | 0.023752969 |
| ANGPTL1 | 10 | 0.023752969 |
| ZNF571 | 10 | 0.023752969 |
| EFHC1 | 10 | 0.023752969 |
| KIRREL1 | 10 | 0.023752969 |
| FAM227B | 10 | 0.023752969 |
| CAPN5 | 10 | 0.023752969 |
| ZNF680 | 10 | 0.023752969 |
| ZBTB7B | 10 | 0.023752969 |
| WRAP53 | 10 | 0.023752969 |
| INO80D | 10 | 0.023752969 |
| IFIT2 | 10 | 0.023752969 |
| MAP3K6 | 10 | 0.023752969 |
| PDPR | 10 | 0.023752969 |
| PPIG | 10 | 0.023752969 |
| STIP1 | 10 | 0.023752969 |
| OR5M1 | 10 | 0.023752969 |
| TFEC | 10 | 0.023752969 |
| SMARCD2 | 10 | 0.023752969 |
| ZNF564 | 10 | 0.023752969 |
| OR14A16 | 10 | 0.023752969 |
| ST8SIA3 | 10 | 0.023752969 |
| RASAL1 | 10 | 0.023752969 |
| DNAJB8 | 10 | 0.023752969 |
| CLCA2 | 10 | 0.023752969 |
| ASIC3 | 10 | 0.023752969 |
| SOX17 | 10 | 0.023752969 |
| SEMA4F | 10 | 0.023752969 |
| OR8K1 | 10 | 0.023752969 |
| PRLHR | 10 | 0.023752969 |
| RNF8 | 10 | 0.023752969 |
| XK | 10 | 0.023752969 |
| NIBAN3 | 10 | 0.023752969 |
| FRK | 10 | 0.023752969 |
| ZNF226 | 10 | 0.023752969 |
| TMPRSS12 | 10 | 0.023752969 |
| ZNF275 | 9 | 0.021377672 |
| CCDC93 | 9 | 0.021377672 |
| LYAR | 9 | 0.021377672 |
| ANGEL1 | 9 | 0.021377672 |
| MUC21 | 9 | 0.021377672 |
| RNF169 | 9 | 0.021377672 |
| GPBP1 | 9 | 0.021377672 |
| LSAMP | 9 | 0.021377672 |
| MPP1 | 9 | 0.021377672 |
| AKAP8 | 9 | 0.021377672 |
| KIF3C | 9 | 0.021377672 |
| IBSP | 9 | 0.021377672 |
| LGALS4 | 9 | 0.021377672 |
| ENPP3 | 9 | 0.021377672 |
| ENTHD1 | 9 | 0.021377672 |
| CRYZ | 9 | 0.021377672 |
| CLCN6 | 9 | 0.021377672 |
| IGHG1 | 9 | 0.021377672 |
| CLPTM1 | 9 | 0.021377672 |
| PIK3R4 | 9 | 0.021377672 |
| DLG1 | 9 | 0.021377672 |
| FBXO43 | 9 | 0.021377672 |
| FAM20A | 9 | 0.021377672 |
| LRRIQ4 | 9 | 0.021377672 |
| SLC44A2 | 9 | 0.021377672 |
| PAK3 | 9 | 0.021377672 |
| SLC24A4 | 9 | 0.021377672 |
| ZC3H7B | 9 | 0.021377672 |
| RIN3 | 9 | 0.021377672 |
| ESR2 | 9 | 0.021377672 |
| SIK2 | 9 | 0.021377672 |
| BACE2 | 9 | 0.021377672 |
| SLC14A2 | 9 | 0.021377672 |
| CYP3A7 | 9 | 0.021377672 |
| CDHR1 | 9 | 0.021377672 |
| ZNF701 | 9 | 0.021377672 |
| OR4Q3 | 9 | 0.021377672 |
| GPC4 | 9 | 0.021377672 |
| HAO1 | 9 | 0.021377672 |
| TPP1 | 9 | 0.021377672 |
| SLC10A2 | 9 | 0.021377672 |
| SLC25A23 | 9 | 0.021377672 |
| HMMR | 9 | 0.021377672 |
| MORC2 | 9 | 0.021377672 |
| MYO1D | 9 | 0.021377672 |
| TLK1 | 9 | 0.021377672 |
| PARP15 | 9 | 0.021377672 |
| PODXL | 9 | 0.021377672 |
| PSKH2 | 9 | 0.021377672 |
| MAPK6 | 9 | 0.021377672 |
| PIPOX | 9 | 0.021377672 |
| INPP5A | 9 | 0.021377672 |
| DNAJC28 | 9 | 0.021377672 |
| SCYL2 | 9 | 0.021377672 |
| ZBTB24 | 9 | 0.021377672 |
| GLRA3 | 9 | 0.021377672 |
| TMTC4 | 9 | 0.021377672 |
| SLC1A1 | 9 | 0.021377672 |
| DLD | 9 | 0.021377672 |
| NAA35 | 9 | 0.021377672 |
| EFCAB7 | 9 | 0.021377672 |
| TCP11 | 9 | 0.021377672 |
| CIB3 | 9 | 0.021377672 |
| FCN2 | 9 | 0.021377672 |
| MR1 | 9 | 0.021377672 |
| CORO2B | 9 | 0.021377672 |
| GALNT2 | 9 | 0.021377672 |
| PRAMEF1 | 9 | 0.021377672 |
| DNTT | 9 | 0.021377672 |
| ADAM15 | 9 | 0.021377672 |
| CCDC174 | 9 | 0.021377672 |
| OOEP | 9 | 0.021377672 |
| HMX2 | 9 | 0.021377672 |
| FMR1 | 9 | 0.021377672 |
| SLC34A1 | 9 | 0.021377672 |
| RAF1 | 9 | 0.021377672 |
| RABGEF1 | 9 | 0.021377672 |
| CRTAC1 | 9 | 0.021377672 |
| PGBD4 | 9 | 0.021377672 |
| SMG8 | 9 | 0.021377672 |
| WDR91 | 9 | 0.021377672 |
| INCENP | 9 | 0.021377672 |
| ZC3H12B | 9 | 0.021377672 |
| POF1B | 9 | 0.021377672 |
| MFN2 | 9 | 0.021377672 |
| CHPF2 | 9 | 0.021377672 |
| BABAM2 | 9 | 0.021377672 |
| OR6N2 | 9 | 0.021377672 |
| MFSD2A | 9 | 0.021377672 |
| MZF1 | 9 | 0.021377672 |
| APBB1IP | 9 | 0.021377672 |
| RXFP1 | 9 | 0.021377672 |
| ASAH2 | 9 | 0.021377672 |
| FAM161B | 9 | 0.021377672 |
| TMPRSS6 | 9 | 0.021377672 |
| AP4B1 | 9 | 0.021377672 |
| CLDN18 | 9 | 0.021377672 |
| GRK5 | 9 | 0.021377672 |
| CPA5 | 9 | 0.021377672 |
| DACH1 | 9 | 0.021377672 |
| RSPH6A | 9 | 0.021377672 |
| GLRX3 | 9 | 0.021377672 |
| GNA14 | 9 | 0.021377672 |
| NAT10 | 9 | 0.021377672 |
| RAD54B | 9 | 0.021377672 |
| ELAVL2 | 9 | 0.021377672 |
| HELLS | 9 | 0.021377672 |
| ANKLE2 | 9 | 0.021377672 |
| KRT2 | 9 | 0.021377672 |
| WHAMM | 9 | 0.021377672 |
| PCID2 | 9 | 0.021377672 |
| SEC16B | 9 | 0.021377672 |
| TAF5 | 9 | 0.021377672 |
| MTX3 | 9 | 0.021377672 |
| GGNBP2 | 9 | 0.021377672 |
| LDOC1 | 9 | 0.021377672 |
| TAB3 | 9 | 0.021377672 |
| TMCC1 | 9 | 0.021377672 |
| OR5AR1 | 9 | 0.021377672 |
| DSTN | 9 | 0.021377672 |
| TRIP4 | 9 | 0.021377672 |
| GGA2 | 9 | 0.021377672 |
| CNGA2 | 9 | 0.021377672 |
| INA | 9 | 0.021377672 |
| ZNF490 | 9 | 0.021377672 |
| CACNG2 | 9 | 0.021377672 |
| COPS6 | 9 | 0.021377672 |
| CIAO3 | 9 | 0.021377672 |
| PTPRE | 9 | 0.021377672 |
| TLCD3B | 9 | 0.021377672 |
| TRIM6 | 9 | 0.021377672 |
| RPAP2 | 9 | 0.021377672 |
| DPP9 | 9 | 0.021377672 |
| CRIM1 | 9 | 0.021377672 |
| PYGO2 | 9 | 0.021377672 |
| CCR1 | 9 | 0.021377672 |
| KLHL13 | 9 | 0.021377672 |
| COG4 | 9 | 0.021377672 |
| LDLRAD3 | 9 | 0.021377672 |
| ECT2L | 9 | 0.021377672 |
| CAMKV | 9 | 0.021377672 |
| GNAQ | 9 | 0.021377672 |
| SF3B3 | 9 | 0.021377672 |
| TRMT12 | 9 | 0.021377672 |
| ACRBP | 9 | 0.021377672 |
| MIB1 | 9 | 0.021377672 |
| ADAM33 | 9 | 0.021377672 |
| MCCC1 | 9 | 0.021377672 |
| TRMT6 | 9 | 0.021377672 |
| ZNF780B | 9 | 0.021377672 |
| GYS1 | 9 | 0.021377672 |
| ADGRF4 | 9 | 0.021377672 |
| AHR | 9 | 0.021377672 |
| IRS2 | 9 | 0.021377672 |
| LPCAT1 | 9 | 0.021377672 |
| DRC7 | 9 | 0.021377672 |
| FLT3 | 9 | 0.021377672 |
| ARSD | 9 | 0.021377672 |
| ZNF618 | 9 | 0.021377672 |
| PFKL | 9 | 0.021377672 |
| FLVCR2 | 9 | 0.021377672 |
| MTMR12 | 9 | 0.021377672 |
| GPR20 | 9 | 0.021377672 |
| MAPK10 | 9 | 0.021377672 |
| ARHGEF9 | 9 | 0.021377672 |
| PPFIA1 | 9 | 0.021377672 |
| DNAJC16 | 9 | 0.021377672 |
| SLC17A1 | 9 | 0.021377672 |
| FKBP6 | 9 | 0.021377672 |
| UBP1 | 9 | 0.021377672 |
| KAT7 | 9 | 0.021377672 |
| BAIAP3 | 9 | 0.021377672 |
| ANKRD34A | 9 | 0.021377672 |
| GAB1 | 9 | 0.021377672 |
| FBXO41 | 9 | 0.021377672 |
| UBE4A | 9 | 0.021377672 |
| EDEM3 | 9 | 0.021377672 |
| PLA1A | 9 | 0.021377672 |
| CFHR4 | 9 | 0.021377672 |
| TRIM54 | 9 | 0.021377672 |
| ARL13B | 9 | 0.021377672 |
| ABTB2 | 9 | 0.021377672 |
| PDIA2 | 9 | 0.021377672 |
| SLC2A4 | 9 | 0.021377672 |
| CHST5 | 9 | 0.021377672 |
| ZNF510 | 9 | 0.021377672 |
| ZFPM1 | 9 | 0.021377672 |
| RAB11FIP2 | 9 | 0.021377672 |
| LGSN | 9 | 0.021377672 |
| KLF8 | 9 | 0.021377672 |
| ZBTB49 | 9 | 0.021377672 |
| MTIF2 | 9 | 0.021377672 |
| SULF2 | 9 | 0.021377672 |
| ZNF529 | 9 | 0.021377672 |
| MGAT1 | 9 | 0.021377672 |
| TBL1X | 9 | 0.021377672 |
| XPOT | 9 | 0.021377672 |
| PRRG3 | 9 | 0.021377672 |
| MATN2 | 9 | 0.021377672 |
| ADGRF5 | 9 | 0.021377672 |
| MAGEB4 | 9 | 0.021377672 |
| HECTD3 | 9 | 0.021377672 |
| L3MBTL1 | 9 | 0.021377672 |
| CD19 | 9 | 0.021377672 |
| USP17L2 | 9 | 0.021377672 |
| OR6K3 | 9 | 0.021377672 |
| OPA1 | 9 | 0.021377672 |
| GGCX | 9 | 0.021377672 |
| CPNE2 | 9 | 0.021377672 |
| HIP1 | 9 | 0.021377672 |
| CLK3 | 9 | 0.021377672 |
| RASSF2 | 9 | 0.021377672 |
| TOP3B | 9 | 0.021377672 |
| TXNRD2 | 9 | 0.021377672 |
| MTMR11 | 9 | 0.021377672 |
| SCUBE1 | 9 | 0.021377672 |
| ZNF14 | 9 | 0.021377672 |
| AP1G1 | 9 | 0.021377672 |
| CPEB4 | 9 | 0.021377672 |
| NRP1 | 9 | 0.021377672 |
| MAB21L1 | 9 | 0.021377672 |
| PTGFR | 9 | 0.021377672 |
| TNK1 | 9 | 0.021377672 |
| AAK1 | 9 | 0.021377672 |
| RMI1 | 9 | 0.021377672 |
| RAPGEF5 | 9 | 0.021377672 |
| C19orf44 | 9 | 0.021377672 |
| BMP3 | 9 | 0.021377672 |
| TEPSIN | 9 | 0.021377672 |
| RRNAD1 | 9 | 0.021377672 |
| ALB | 9 | 0.021377672 |
| ZNF549 | 9 | 0.021377672 |
| INTU | 9 | 0.021377672 |
| PPIL4 | 9 | 0.021377672 |
| ZNF652 | 9 | 0.021377672 |
| GPD2 | 9 | 0.021377672 |
| ACTRT1 | 9 | 0.021377672 |
| FTMT | 9 | 0.021377672 |
| GORASP2 | 9 | 0.021377672 |
| BICD1 | 9 | 0.021377672 |
| DGKG | 9 | 0.021377672 |
| KRT5 | 9 | 0.021377672 |
| LIPC | 9 | 0.021377672 |
| ELAPOR1 | 9 | 0.021377672 |
| MTBP | 9 | 0.021377672 |
| CNDP2 | 9 | 0.021377672 |
| TRAF3 | 9 | 0.021377672 |
| PFKFB1 | 9 | 0.021377672 |
| XIAP | 9 | 0.021377672 |
| USP13 | 9 | 0.021377672 |
| MAP4K5 | 9 | 0.021377672 |
| AK5 | 9 | 0.021377672 |
| THAP9 | 9 | 0.021377672 |
| AP002748.5 | 9 | 0.021377672 |
| EPSTI1 | 9 | 0.021377672 |
| LILRB3 | 9 | 0.021377672 |
| PHF20 | 9 | 0.021377672 |
| GJB1 | 9 | 0.021377672 |
| BNIP5 | 9 | 0.021377672 |
| ZP4 | 9 | 0.021377672 |
| ACTL7B | 9 | 0.021377672 |
| RIC1 | 9 | 0.021377672 |
| GABRR2 | 9 | 0.021377672 |
| RGMB | 9 | 0.021377672 |
| ZNF30 | 9 | 0.021377672 |
| SLC45A3 | 9 | 0.021377672 |
| CNOT11 | 9 | 0.021377672 |
| AQP7 | 9 | 0.021377672 |
| RECQL4 | 9 | 0.021377672 |
| CXorf66 | 9 | 0.021377672 |
| CDC25A | 9 | 0.021377672 |
| IFT81 | 9 | 0.021377672 |
| G6PD | 9 | 0.021377672 |
| LSP1 | 9 | 0.021377672 |
| ZNF317 | 9 | 0.021377672 |
| USP1 | 9 | 0.021377672 |
| ACAP3 | 9 | 0.021377672 |
| PLEKHH3 | 9 | 0.021377672 |
| CHGB | 9 | 0.021377672 |
| DYTN | 9 | 0.021377672 |
| TOX3 | 9 | 0.021377672 |
| TTF2 | 9 | 0.021377672 |
| PI15 | 9 | 0.021377672 |
| CHIT1 | 9 | 0.021377672 |
| HOXC10 | 9 | 0.021377672 |
| TAF4B | 9 | 0.021377672 |
| RNF165 | 9 | 0.021377672 |
| CBX6 | 9 | 0.021377672 |
| PRKG2 | 9 | 0.021377672 |
| ZNF562 | 9 | 0.021377672 |
| ALDH4A1 | 9 | 0.021377672 |
| GP1BA | 9 | 0.021377672 |
| VPS37B | 9 | 0.021377672 |
| GRHL2 | 9 | 0.021377672 |
| USP45 | 9 | 0.021377672 |
| ITPRIP | 9 | 0.021377672 |
| OR7E24 | 9 | 0.021377672 |
| PADI3 | 9 | 0.021377672 |
| HTR1F | 9 | 0.021377672 |
| MYBL2 | 9 | 0.021377672 |
| CPT1C | 9 | 0.021377672 |
| ZCCHC12 | 9 | 0.021377672 |
| PELI3 | 9 | 0.021377672 |
| MMEL1 | 9 | 0.021377672 |
| WDCP | 9 | 0.021377672 |
| ICA1 | 9 | 0.021377672 |
| PLEKHA2 | 9 | 0.021377672 |
| SSRP1 | 9 | 0.021377672 |
| PRDM14 | 9 | 0.021377672 |
| SLC7A3 | 9 | 0.021377672 |
| THYN1 | 9 | 0.021377672 |
| NUFIP1 | 9 | 0.021377672 |
| STIL | 9 | 0.021377672 |
| GSAP | 9 | 0.021377672 |
| FAM133A | 9 | 0.021377672 |
| PTER | 9 | 0.021377672 |
| MEP1A | 9 | 0.021377672 |
| PIWIL3 | 9 | 0.021377672 |
| PDLIM5 | 9 | 0.021377672 |
| VNN1 | 9 | 0.021377672 |
| OR4C46 | 9 | 0.021377672 |
| ARID5A | 9 | 0.021377672 |
| RSPO3 | 9 | 0.021377672 |
| EDEM2 | 9 | 0.021377672 |
| CCDC96 | 9 | 0.021377672 |
| OTOP3 | 9 | 0.021377672 |
| TWNK | 9 | 0.021377672 |
| PRCC | 9 | 0.021377672 |
| PDIA3 | 9 | 0.021377672 |
| ZNF813 | 9 | 0.021377672 |
| ZNF432 | 9 | 0.021377672 |
| NOLC1 | 9 | 0.021377672 |
| OTX2 | 9 | 0.021377672 |
| AADACL4 | 9 | 0.021377672 |
| MEPE | 9 | 0.021377672 |
| NR1D1 | 9 | 0.021377672 |
| NASP | 9 | 0.021377672 |
| EHBP1L1 | 9 | 0.021377672 |
| MORC3 | 9 | 0.021377672 |
| SEC14L4 | 9 | 0.021377672 |
| PKP1 | 9 | 0.021377672 |
| CDCA2 | 9 | 0.021377672 |
| COMP | 9 | 0.021377672 |
| KIFC1 | 9 | 0.021377672 |
| SNCAIP | 9 | 0.021377672 |
| ARHGEF28 | 9 | 0.021377672 |
| ITGA9 | 9 | 0.021377672 |
| ADAM22 | 9 | 0.021377672 |
| ADGRE2 | 9 | 0.021377672 |
| PTPN6 | 9 | 0.021377672 |
| FAM98A | 9 | 0.021377672 |
| TBC1D10A | 9 | 0.021377672 |
| MTM1 | 9 | 0.021377672 |
| RHOBTB3 | 9 | 0.021377672 |
| COLGALT2 | 9 | 0.021377672 |
| ZFP37 | 9 | 0.021377672 |
| BCAR3 | 9 | 0.021377672 |
| GABRB1 | 9 | 0.021377672 |
| ALOX15B | 9 | 0.021377672 |
| HAUS5 | 9 | 0.021377672 |
| SLC9A2 | 9 | 0.021377672 |
| FGF14 | 9 | 0.021377672 |
| IL17RC | 9 | 0.021377672 |
| FAM102A | 9 | 0.021377672 |
| ACTN2 | 9 | 0.021377672 |
| C11orf87 | 9 | 0.021377672 |
| SCEL | 9 | 0.021377672 |
| GABRR3 | 9 | 0.021377672 |
| FAM126A | 9 | 0.021377672 |
| RTP1 | 9 | 0.021377672 |
| LNX2 | 9 | 0.021377672 |
| RPA1 | 9 | 0.021377672 |
| POFUT2 | 9 | 0.021377672 |
| KCNK13 | 9 | 0.021377672 |
| FAM120C | 9 | 0.021377672 |
| IMMT | 9 | 0.021377672 |
| OR10J1 | 9 | 0.021377672 |
| FBXO5 | 9 | 0.021377672 |
| COX10 | 9 | 0.021377672 |
| OR7C1 | 9 | 0.021377672 |
| PLAG1 | 9 | 0.021377672 |
| CAVIN1 | 9 | 0.021377672 |
| S1PR5 | 9 | 0.021377672 |
| ZNF141 | 9 | 0.021377672 |
| SLC5A4 | 9 | 0.021377672 |
| ADSS1 | 9 | 0.021377672 |
| SLC7A1 | 9 | 0.021377672 |
| SMARCC1 | 9 | 0.021377672 |
| STX11 | 9 | 0.021377672 |
| SLFNL1 | 9 | 0.021377672 |
| LILRB1 | 9 | 0.021377672 |
| HTR3B | 9 | 0.021377672 |
| MANEAL | 9 | 0.021377672 |
| SPAG6 | 9 | 0.021377672 |
| MALT1 | 9 | 0.021377672 |
| DLG3 | 9 | 0.021377672 |
| HADHB | 9 | 0.021377672 |
| PTPN11 | 9 | 0.021377672 |
| ZBTB11 | 9 | 0.021377672 |
| MTHFSD | 9 | 0.021377672 |
| TADA3 | 9 | 0.021377672 |
| DPEP2 | 9 | 0.021377672 |
| RANBP3 | 9 | 0.021377672 |
| ZNF773 | 9 | 0.021377672 |
| C9orf131 | 9 | 0.021377672 |
| RGS7 | 9 | 0.021377672 |
| SFPQ | 9 | 0.021377672 |
| GFRA2 | 9 | 0.021377672 |
| C12orf4 | 9 | 0.021377672 |
| KNG1 | 9 | 0.021377672 |
| RC3H2 | 9 | 0.021377672 |
| ARFIP1 | 9 | 0.021377672 |
| RFX2 | 9 | 0.021377672 |
| SLC2A9 | 9 | 0.021377672 |
| RBM48 | 9 | 0.021377672 |
| GPC3 | 9 | 0.021377672 |
| WASHC5 | 9 | 0.021377672 |
| DNM1L | 9 | 0.021377672 |
| CFAP206 | 9 | 0.021377672 |
| NUP93 | 9 | 0.021377672 |
| PRL | 9 | 0.021377672 |
| RCN3 | 9 | 0.021377672 |
| SIRT1 | 9 | 0.021377672 |
| CACNB1 | 9 | 0.021377672 |
| UBR2 | 9 | 0.021377672 |
| PAX1 | 9 | 0.021377672 |
| TMCC3 | 9 | 0.021377672 |
| TACC3 | 9 | 0.021377672 |
| ZMYND15 | 9 | 0.021377672 |
| SUN1 | 9 | 0.021377672 |
| TLK2 | 9 | 0.021377672 |
| CCDC8 | 9 | 0.021377672 |
| XRN2 | 9 | 0.021377672 |
| SLC27A3 | 9 | 0.021377672 |
| OR5B3 | 9 | 0.021377672 |
| SLC7A11 | 9 | 0.021377672 |
| XRCC6 | 9 | 0.021377672 |
| GPC2 | 9 | 0.021377672 |
| ZC3H12A | 9 | 0.021377672 |
| LRRC1 | 9 | 0.021377672 |
| SLC8A3 | 9 | 0.021377672 |
| CYP1A2 | 9 | 0.021377672 |
| DBH | 9 | 0.021377672 |
| H1-2 | 9 | 0.021377672 |
| KRT10 | 9 | 0.021377672 |
| SLF2 | 9 | 0.021377672 |
| GJD2 | 9 | 0.021377672 |
| ZNF84 | 9 | 0.021377672 |
| RARS2 | 9 | 0.021377672 |
| PHC3 | 9 | 0.021377672 |
| PNLIP | 9 | 0.021377672 |
| OSBPL2 | 9 | 0.021377672 |
| OLFML1 | 9 | 0.021377672 |
| KIFAP3 | 9 | 0.021377672 |
| CPEB1 | 9 | 0.021377672 |
| MEP1B | 9 | 0.021377672 |
| C21orf58 | 9 | 0.021377672 |
| STXBP1 | 9 | 0.021377672 |
| MAP3K2 | 9 | 0.021377672 |
| CELF2 | 9 | 0.021377672 |
| BTN2A1 | 9 | 0.021377672 |
| OMD | 9 | 0.021377672 |
| SCML2 | 9 | 0.021377672 |
| DLX5 | 9 | 0.021377672 |
| PHKA1 | 9 | 0.021377672 |
| FMO4 | 9 | 0.021377672 |
| KERA | 9 | 0.021377672 |
| NSRP1 | 9 | 0.021377672 |
| ADCK1 | 9 | 0.021377672 |
| OR1A2 | 9 | 0.021377672 |
| GABRA3 | 9 | 0.021377672 |
| KRT39 | 9 | 0.021377672 |
| PPP1R12C | 9 | 0.021377672 |
| FAM161A | 9 | 0.021377672 |
| ZNF175 | 9 | 0.021377672 |
| DRD1 | 9 | 0.021377672 |
| LINS1 | 9 | 0.021377672 |
| WIPF1 | 9 | 0.021377672 |
| ZNF85 | 9 | 0.021377672 |
| TCHP | 9 | 0.021377672 |
| SLC6A5 | 9 | 0.021377672 |
| FBL | 9 | 0.021377672 |
| TIGD2 | 9 | 0.021377672 |
| SNIP1 | 9 | 0.021377672 |
| ASCC2 | 9 | 0.021377672 |
| ZNF660 | 9 | 0.021377672 |
| ACTR3B | 9 | 0.021377672 |
| CCER1 | 9 | 0.021377672 |
| RIOK1 | 9 | 0.021377672 |
| GLT6D1 | 9 | 0.021377672 |
| TFR2 | 9 | 0.021377672 |
| PRAMEF18 | 9 | 0.021377672 |
| EDEM1 | 9 | 0.021377672 |
| BSX | 9 | 0.021377672 |
| KCNK18 | 9 | 0.021377672 |
| OR5F1 | 9 | 0.021377672 |
| IL17RD | 9 | 0.021377672 |
| HOXD12 | 9 | 0.021377672 |
| SLCO1A2 | 9 | 0.021377672 |
| CWC22 | 9 | 0.021377672 |
| SLC2A2 | 9 | 0.021377672 |
| CARF | 9 | 0.021377672 |
| INAVA | 9 | 0.021377672 |
| CX3CR1 | 9 | 0.021377672 |
| AFAP1 | 9 | 0.021377672 |
| BMP5 | 9 | 0.021377672 |
| DMRTB1 | 9 | 0.021377672 |
| RELA | 9 | 0.021377672 |
| LPCAT4 | 9 | 0.021377672 |
| YIF1A | 9 | 0.021377672 |
| MSLNL | 9 | 0.021377672 |
| MMD2 | 9 | 0.021377672 |
| PDZD7 | 9 | 0.021377672 |
| ADRA1D | 9 | 0.021377672 |
| CLNK | 9 | 0.021377672 |
| SULT6B1 | 9 | 0.021377672 |
| ICAM5 | 9 | 0.021377672 |
| KCNJ16 | 9 | 0.021377672 |
| BEST1 | 9 | 0.021377672 |
| TRPV3 | 9 | 0.021377672 |
| VPS54 | 9 | 0.021377672 |
| SLC30A8 | 9 | 0.021377672 |
| CACNG5 | 9 | 0.021377672 |
| MLIP | 9 | 0.021377672 |
| TMEM63A | 9 | 0.021377672 |
| TTC39C | 9 | 0.021377672 |
| ZP2 | 9 | 0.021377672 |
| SLC9A7 | 9 | 0.021377672 |
| FIZ1 | 9 | 0.021377672 |
| CSTF3 | 9 | 0.021377672 |
| ACSM4 | 9 | 0.021377672 |
| MAGEB2 | 9 | 0.021377672 |
| BTN2A2 | 9 | 0.021377672 |
| KIAA1958 | 9 | 0.021377672 |
| RIMKLB | 9 | 0.021377672 |
| SEMA4A | 9 | 0.021377672 |
| SLC23A2 | 9 | 0.021377672 |
| NF2 | 9 | 0.021377672 |
| POLDIP3 | 9 | 0.021377672 |
| AKT3 | 9 | 0.021377672 |
| EPC1 | 9 | 0.021377672 |
| TPSD1 | 9 | 0.021377672 |
| ZNF526 | 9 | 0.021377672 |
| SLC38A10 | 9 | 0.021377672 |
| IRAK2 | 9 | 0.021377672 |
| KIR3DL3 | 9 | 0.021377672 |
| ZNHIT6 | 9 | 0.021377672 |
| NSMAF | 9 | 0.021377672 |
| TBC1D23 | 9 | 0.021377672 |
| USH1G | 9 | 0.021377672 |
| MIB2 | 9 | 0.021377672 |
| ENPP1 | 9 | 0.021377672 |
| ONECUT2 | 9 | 0.021377672 |
| CSGALNACT2 | 9 | 0.021377672 |
| SEZ6 | 9 | 0.021377672 |
| ZMIZ1 | 9 | 0.021377672 |
| TMEM108 | 9 | 0.021377672 |
| WAC | 9 | 0.021377672 |
| WBP4 | 9 | 0.021377672 |
| NUDT7 | 9 | 0.021377672 |
| AC013489.1 | 9 | 0.021377672 |
| RTKN | 9 | 0.021377672 |
| COG2 | 9 | 0.021377672 |
| ZSWIM4 | 9 | 0.021377672 |
| ZNF264 | 9 | 0.021377672 |
| SH3GL3 | 9 | 0.021377672 |
| RNF112 | 9 | 0.021377672 |
| KIFC2 | 9 | 0.021377672 |
| GAN | 9 | 0.021377672 |
| CCDC57 | 9 | 0.021377672 |
| NOX5 | 9 | 0.021377672 |
| UGT2B4 | 9 | 0.021377672 |
| SIGLEC5 | 9 | 0.021377672 |
| PUS7L | 9 | 0.021377672 |
| MERTK | 9 | 0.021377672 |
| TNIP3 | 9 | 0.021377672 |
| C2orf78 | 9 | 0.021377672 |
| NR1D2 | 9 | 0.021377672 |
| TGM1 | 9 | 0.021377672 |
| ACOT7 | 9 | 0.021377672 |
| FPGT | 9 | 0.021377672 |
| FMR1NB | 9 | 0.021377672 |
| SNX14 | 9 | 0.021377672 |
| TMEM87B | 9 | 0.021377672 |
| ERRFI1 | 9 | 0.021377672 |
| CLCN7 | 9 | 0.021377672 |
| KATNB1 | 9 | 0.021377672 |
| CD276 | 9 | 0.021377672 |
| ADD1 | 9 | 0.021377672 |
| ARSL | 9 | 0.021377672 |
| VEZT | 9 | 0.021377672 |
| MAP3K15 | 9 | 0.021377672 |
| SIGLEC11 | 9 | 0.021377672 |
| SLC11A2 | 9 | 0.021377672 |
| GPRC6A | 9 | 0.021377672 |
| CDC27 | 9 | 0.021377672 |
| CHRND | 9 | 0.021377672 |
| PADI6 | 9 | 0.021377672 |
| AVPR1B | 9 | 0.021377672 |
| SLC5A6 | 9 | 0.021377672 |
| ZNF217 | 9 | 0.021377672 |
| ARHGAP25 | 9 | 0.021377672 |
| CYP11B2 | 9 | 0.021377672 |
| PRPF40A | 9 | 0.021377672 |
| LPO | 9 | 0.021377672 |
| GPAT3 | 9 | 0.021377672 |
| CATSPER3 | 9 | 0.021377672 |
| ZNF704 | 9 | 0.021377672 |
| PHF12 | 9 | 0.021377672 |
| SNX15 | 9 | 0.021377672 |
| RTP5 | 9 | 0.021377672 |
| LCMT2 | 9 | 0.021377672 |
| OR51V1 | 9 | 0.021377672 |
| OR9A4 | 9 | 0.021377672 |
| KRT37 | 9 | 0.021377672 |
| CAPN15 | 9 | 0.021377672 |
| SLC46A3 | 9 | 0.021377672 |
| GCC1 | 9 | 0.021377672 |
| AFAP1L2 | 9 | 0.021377672 |
| CARNS1 | 9 | 0.021377672 |
| PLEKHG3 | 9 | 0.021377672 |
| MUC20 | 9 | 0.021377672 |
| UVSSA | 9 | 0.021377672 |
| NT5C2 | 9 | 0.021377672 |
| SCFD2 | 9 | 0.021377672 |
| OR10A3 | 9 | 0.021377672 |
| MSLN | 9 | 0.021377672 |
| TUT1 | 9 | 0.021377672 |
| ITCH | 9 | 0.021377672 |
| STXBP3 | 9 | 0.021377672 |
| TEKT5 | 9 | 0.021377672 |
| ADRA1A | 9 | 0.021377672 |
| CHUK | 9 | 0.021377672 |
| NKX2-2 | 9 | 0.021377672 |
| OR5L2 | 9 | 0.021377672 |
| WDR1 | 9 | 0.021377672 |
| ERCC6L | 9 | 0.021377672 |
| ZNF763 | 9 | 0.021377672 |
| LIMK2 | 9 | 0.021377672 |
| ISG20L2 | 9 | 0.021377672 |
| SUSD4 | 9 | 0.021377672 |
| ZFP2 | 9 | 0.021377672 |
| PRPF38B | 9 | 0.021377672 |
| COL13A1 | 9 | 0.021377672 |
| ZNF480 | 9 | 0.021377672 |
| NCAPH | 9 | 0.021377672 |
| MMP3 | 9 | 0.021377672 |
| CPT1A | 9 | 0.021377672 |
| UNC13D | 9 | 0.021377672 |
| NBN | 9 | 0.021377672 |
| CHIA | 9 | 0.021377672 |
| ZNF587 | 9 | 0.021377672 |
| OAS3 | 9 | 0.021377672 |
| TBX22 | 9 | 0.021377672 |
| GREM2 | 9 | 0.021377672 |
| NT5C1A | 9 | 0.021377672 |
| OR10H5 | 9 | 0.021377672 |
| TMEM104 | 9 | 0.021377672 |
| P4HB | 9 | 0.021377672 |
| YME1L1 | 9 | 0.021377672 |
| HLA-C | 9 | 0.021377672 |
| KCNK9 | 9 | 0.021377672 |
| NCOA4 | 9 | 0.021377672 |
| ZNF284 | 9 | 0.021377672 |
| ALDH8A1 | 9 | 0.021377672 |
| FIP1L1 | 9 | 0.021377672 |
| GML | 9 | 0.021377672 |
| CFAP77 | 9 | 0.021377672 |
| IHH | 9 | 0.021377672 |
| DTL | 9 | 0.021377672 |
| CACNB4 | 9 | 0.021377672 |
| FSD2 | 9 | 0.021377672 |
| TBXAS1 | 9 | 0.021377672 |
| PPP1R12B | 9 | 0.021377672 |
| GSPT2 | 9 | 0.021377672 |
| GJC1 | 9 | 0.021377672 |
| PIK3C2A | 9 | 0.021377672 |
| PPARGC1B | 9 | 0.021377672 |
| WAPL | 9 | 0.021377672 |
| CPA1 | 9 | 0.021377672 |
| CBLL2 | 9 | 0.021377672 |
| SERPINI2 | 9 | 0.021377672 |
| CHRDL2 | 9 | 0.021377672 |
| NECTIN1 | 9 | 0.021377672 |
| MAML1 | 9 | 0.021377672 |
| AP1S1 | 9 | 0.021377672 |
| USP36 | 9 | 0.021377672 |
| TMEM181 | 9 | 0.021377672 |
| LRRTM3 | 9 | 0.021377672 |
| CCDC181 | 9 | 0.021377672 |
| NOP9 | 9 | 0.021377672 |
| OFD1 | 9 | 0.021377672 |
| STRIP1 | 9 | 0.021377672 |
| RALGDS | 9 | 0.021377672 |
| KANSL3 | 9 | 0.021377672 |
| TUFM | 9 | 0.021377672 |
| TRPC4AP | 9 | 0.021377672 |
| HYOU1 | 9 | 0.021377672 |
| OR51E2 | 9 | 0.021377672 |
| MFSD5 | 9 | 0.021377672 |
| PPP6R3 | 9 | 0.021377672 |
| ERC1 | 9 | 0.021377672 |
| CARNMT1 | 9 | 0.021377672 |
| GAL3ST4 | 9 | 0.021377672 |
| RNF25 | 9 | 0.021377672 |
| NEDD1 | 9 | 0.021377672 |
| ST8SIA1 | 9 | 0.021377672 |
| RHOT1 | 9 | 0.021377672 |
| PARPBP | 9 | 0.021377672 |
| IGFN1 | 9 | 0.021377672 |
| TLR5 | 9 | 0.021377672 |
| P2RY13 | 9 | 0.021377672 |
| DSC3 | 9 | 0.021377672 |
| PDGFD | 9 | 0.021377672 |
| ERMP1 | 9 | 0.021377672 |
| RAB34 | 9 | 0.021377672 |
| ILDR2 | 9 | 0.021377672 |
| CDC20B | 9 | 0.021377672 |
| SIRPA | 9 | 0.021377672 |
| SSBP4 | 9 | 0.021377672 |
| CABS1 | 9 | 0.021377672 |
| MMP19 | 9 | 0.021377672 |
| ZCCHC14 | 9 | 0.021377672 |
| SPOPL | 9 | 0.021377672 |
| CERS3 | 9 | 0.021377672 |
| RAD21 | 9 | 0.021377672 |
| CYC1 | 9 | 0.021377672 |
| STK39 | 9 | 0.021377672 |
| ADORA1 | 9 | 0.021377672 |
| MAP4K3 | 9 | 0.021377672 |
| POLN | 9 | 0.021377672 |
| TRIM4 | 9 | 0.021377672 |
| TMC1 | 9 | 0.021377672 |
| RHPN1 | 9 | 0.021377672 |
| CNOT9 | 9 | 0.021377672 |
| PHF1 | 9 | 0.021377672 |
| MTHFD1 | 9 | 0.021377672 |
| LSS | 9 | 0.021377672 |
| KRT6A | 9 | 0.021377672 |
| OR1J1 | 9 | 0.021377672 |
| ACSL1 | 9 | 0.021377672 |
| QSOX2 | 9 | 0.021377672 |
| CLDN10 | 9 | 0.021377672 |
| INHA | 9 | 0.021377672 |
| GP6 | 9 | 0.021377672 |
| ASZ1 | 9 | 0.021377672 |
| HMG20A | 9 | 0.021377672 |
| GATA1 | 9 | 0.021377672 |
| SNW1 | 9 | 0.021377672 |
| OPRL1 | 9 | 0.021377672 |
| TOMM70 | 9 | 0.021377672 |
| OR10G9 | 9 | 0.021377672 |
| AGTR1 | 9 | 0.021377672 |
| CAPN3 | 9 | 0.021377672 |
| CSNK1D | 9 | 0.021377672 |
| ZNF800 | 9 | 0.021377672 |
| PDE3B | 9 | 0.021377672 |
| TUBGCP4 | 9 | 0.021377672 |
| TFDP1 | 9 | 0.021377672 |
| PAF1 | 9 | 0.021377672 |
| SCARB2 | 9 | 0.021377672 |
| CBFA2T2 | 9 | 0.021377672 |
| TAF6L | 9 | 0.021377672 |
| NPR3 | 9 | 0.021377672 |
| SH3D21 | 9 | 0.021377672 |
| USP11 | 9 | 0.021377672 |
| MCCC2 | 9 | 0.021377672 |
| IGF2 | 9 | 0.021377672 |
| ANTXR2 | 9 | 0.021377672 |
| GARRE1 | 9 | 0.021377672 |
| GDAP2 | 9 | 0.021377672 |
| ZNF251 | 9 | 0.021377672 |
| PDE6C | 9 | 0.021377672 |
| IL31RA | 9 | 0.021377672 |
| ALDH1A1 | 9 | 0.021377672 |
| DMP1 | 9 | 0.021377672 |
| GALNT11 | 9 | 0.021377672 |
| DNAH12 | 9 | 0.021377672 |
| FOXRED2 | 9 | 0.021377672 |
| WNT11 | 9 | 0.021377672 |
| FIBCD1 | 9 | 0.021377672 |
| VIPR2 | 9 | 0.021377672 |
| MAP4K1 | 9 | 0.021377672 |
| CLU | 9 | 0.021377672 |
| TUBA3E | 9 | 0.021377672 |
| DES | 9 | 0.021377672 |
| OR9G1 | 9 | 0.021377672 |
| STT3A | 9 | 0.021377672 |
| TMC6 | 9 | 0.021377672 |
| SPICE1 | 9 | 0.021377672 |
| DBF4 | 9 | 0.021377672 |
| SCNN1B | 9 | 0.021377672 |
| PPP2R5A | 9 | 0.021377672 |
| HSPA6 | 9 | 0.021377672 |
| ATG4D | 9 | 0.021377672 |
| RYK | 9 | 0.021377672 |
| KRIT1 | 9 | 0.021377672 |
| ACSBG2 | 9 | 0.021377672 |
| ZNF683 | 9 | 0.021377672 |
| OR10G7 | 9 | 0.021377672 |
| ZSCAN5B | 9 | 0.021377672 |
| PIGR | 9 | 0.021377672 |
| RFX4 | 9 | 0.021377672 |
| UGT2B11 | 9 | 0.021377672 |
| BAG6 | 9 | 0.021377672 |
| BTRC | 9 | 0.021377672 |
| GDPD2 | 9 | 0.021377672 |
| PALMD | 9 | 0.021377672 |
| CYP8B1 | 9 | 0.021377672 |
| SPATC1 | 9 | 0.021377672 |
| PDLIM1 | 9 | 0.021377672 |
| PIK3C3 | 9 | 0.021377672 |
| GIPC3 | 9 | 0.021377672 |
| ZNF267 | 9 | 0.021377672 |
| NOP58 | 9 | 0.021377672 |
| SLFN12 | 9 | 0.021377672 |
| SYT12 | 9 | 0.021377672 |
| ZNF347 | 9 | 0.021377672 |
| CFAP20DC | 9 | 0.021377672 |
| SCAI | 9 | 0.021377672 |
| SRL | 9 | 0.021377672 |
| TAAR9 | 9 | 0.021377672 |
| INSL6 | 9 | 0.021377672 |
| IL1R1 | 9 | 0.021377672 |
| BMPR1A | 9 | 0.021377672 |
| KIF2C | 9 | 0.021377672 |
| SLC18A1 | 9 | 0.021377672 |
| ARMH3 | 9 | 0.021377672 |
| CYP2R1 | 9 | 0.021377672 |
| TTLL9 | 9 | 0.021377672 |
| KIF7 | 9 | 0.021377672 |
| MTSS1 | 9 | 0.021377672 |
| AARS1 | 9 | 0.021377672 |
| PLEK | 9 | 0.021377672 |
| TULP1 | 9 | 0.021377672 |
| CHRNB1 | 9 | 0.021377672 |
| OR4X1 | 9 | 0.021377672 |
| NHLRC2 | 9 | 0.021377672 |
| SYNCRIP | 9 | 0.021377672 |
| NBPF11 | 9 | 0.021377672 |
| COL9A3 | 9 | 0.021377672 |
| ARMC2 | 9 | 0.021377672 |
| CAPS2 | 9 | 0.021377672 |
| SNRK | 9 | 0.021377672 |
| GNAT1 | 9 | 0.021377672 |
| OTOP1 | 9 | 0.021377672 |
| MTNR1B | 9 | 0.021377672 |
| INPP5J | 9 | 0.021377672 |
| GRK6 | 9 | 0.021377672 |
| ALDH1L1 | 9 | 0.021377672 |
| HEATR3 | 9 | 0.021377672 |
| RUFY1 | 9 | 0.021377672 |
| TSPYL5 | 9 | 0.021377672 |
| PUS10 | 9 | 0.021377672 |
| PTH1R | 8 | 0.019002375 |
| OR51S1 | 8 | 0.019002375 |
| FLYWCH1 | 8 | 0.019002375 |
| SLC45A2 | 8 | 0.019002375 |
| ZBTB48 | 8 | 0.019002375 |
| KRT6B | 8 | 0.019002375 |
| KRI1 | 8 | 0.019002375 |
| CMTR1 | 8 | 0.019002375 |
| LSM14A | 8 | 0.019002375 |
| HOOK3 | 8 | 0.019002375 |
| CAPN12 | 8 | 0.019002375 |
| EDNRA | 8 | 0.019002375 |
| VPS33B | 8 | 0.019002375 |
| CYP46A1 | 8 | 0.019002375 |
| IKBKB | 8 | 0.019002375 |
| FMO2 | 8 | 0.019002375 |
| ZNF554 | 8 | 0.019002375 |
| DLG4 | 8 | 0.019002375 |
| IL1RAP | 8 | 0.019002375 |
| SIDT1 | 8 | 0.019002375 |
| TLR4 | 8 | 0.019002375 |
| SERPINE2 | 8 | 0.019002375 |
| EPB41L4A | 8 | 0.019002375 |
| DDX55 | 8 | 0.019002375 |
| KCNC1 | 8 | 0.019002375 |
| FARSA | 8 | 0.019002375 |
| NOD1 | 8 | 0.019002375 |
| XRCC2 | 8 | 0.019002375 |
| PSG1 | 8 | 0.019002375 |
| AIFM3 | 8 | 0.019002375 |
| FBP2 | 8 | 0.019002375 |
| CYP4F2 | 8 | 0.019002375 |
| AKR1B1 | 8 | 0.019002375 |
| LDLR | 8 | 0.019002375 |
| SRSF6 | 8 | 0.019002375 |
| DMTN | 8 | 0.019002375 |
| GPC1 | 8 | 0.019002375 |
| OR1L1 | 8 | 0.019002375 |
| HTR5A | 8 | 0.019002375 |
| ZNF189 | 8 | 0.019002375 |
| PLS3 | 8 | 0.019002375 |
| LPIN1 | 8 | 0.019002375 |
| ZGPAT | 8 | 0.019002375 |
| EMID1 | 8 | 0.019002375 |
| GAS7 | 8 | 0.019002375 |
| TNIP1 | 8 | 0.019002375 |
| RARG | 8 | 0.019002375 |
| PRIMPOL | 8 | 0.019002375 |
| KLB | 8 | 0.019002375 |
| ZNF544 | 8 | 0.019002375 |
| SLC25A28 | 8 | 0.019002375 |
| PDK2 | 8 | 0.019002375 |
| LMX1B | 8 | 0.019002375 |
| MAN1B1 | 8 | 0.019002375 |
| OR51A4 | 8 | 0.019002375 |
| HJV | 8 | 0.019002375 |
| QSOX1 | 8 | 0.019002375 |
| SRBD1 | 8 | 0.019002375 |
| SLC13A2 | 8 | 0.019002375 |
| MOK | 8 | 0.019002375 |
| EME1 | 8 | 0.019002375 |
| GLB1L3 | 8 | 0.019002375 |
| DHX40 | 8 | 0.019002375 |
| EVL | 8 | 0.019002375 |
| CYP26B1 | 8 | 0.019002375 |
| OR13D1 | 8 | 0.019002375 |
| CLEC18B | 8 | 0.019002375 |
| CREB3L1 | 8 | 0.019002375 |
| ENPP5 | 8 | 0.019002375 |
| LCA5L | 8 | 0.019002375 |
| IL3RA | 8 | 0.019002375 |
| ZNF470 | 8 | 0.019002375 |
| TINAG | 8 | 0.019002375 |
| ZNF491 | 8 | 0.019002375 |
| GRAMD4 | 8 | 0.019002375 |
| FHL5 | 8 | 0.019002375 |
| KCNJ5 | 8 | 0.019002375 |
| OR4K2 | 8 | 0.019002375 |
| KLK4 | 8 | 0.019002375 |
| POMGNT2 | 8 | 0.019002375 |
| BOP1 | 8 | 0.019002375 |
| AC006059.2 | 8 | 0.019002375 |
| OR2B6 | 8 | 0.019002375 |
| C2CD6 | 8 | 0.019002375 |
| OR10C1 | 8 | 0.019002375 |
| DPYSL3 | 8 | 0.019002375 |
| THBS4 | 8 | 0.019002375 |
| GHR | 8 | 0.019002375 |
| SASS6 | 8 | 0.019002375 |
| PLK4 | 8 | 0.019002375 |
| SLC16A13 | 8 | 0.019002375 |
| LARP1B | 8 | 0.019002375 |
| ZXDB | 8 | 0.019002375 |
| ALCAM | 8 | 0.019002375 |
| CCNF | 8 | 0.019002375 |
| MED24 | 8 | 0.019002375 |
| FMNL1 | 8 | 0.019002375 |
| INTS3 | 8 | 0.019002375 |
| KIF23 | 8 | 0.019002375 |
| HAPLN4 | 8 | 0.019002375 |
| ZNF623 | 8 | 0.019002375 |
| FLOT1 | 8 | 0.019002375 |
| OR5H1 | 8 | 0.019002375 |
| LILRA4 | 8 | 0.019002375 |
| SNRPN | 8 | 0.019002375 |
| ZNF577 | 8 | 0.019002375 |
| AP2M1 | 8 | 0.019002375 |
| VIL1 | 8 | 0.019002375 |
| SKA3 | 8 | 0.019002375 |
| SLC6A20 | 8 | 0.019002375 |
| SEMA3B | 8 | 0.019002375 |
| CST1 | 8 | 0.019002375 |
| CLEC4D | 8 | 0.019002375 |
| SH2D3A | 8 | 0.019002375 |
| RAB6B | 8 | 0.019002375 |
| CAPN10 | 8 | 0.019002375 |
| IL13RA2 | 8 | 0.019002375 |
| NME8 | 8 | 0.019002375 |
| TACC1 | 8 | 0.019002375 |
| FANCC | 8 | 0.019002375 |
| MBOAT1 | 8 | 0.019002375 |
| TM4SF4 | 8 | 0.019002375 |
| EIF3E | 8 | 0.019002375 |
| ENTPD3 | 8 | 0.019002375 |
| BCKDK | 8 | 0.019002375 |
| MNDA | 8 | 0.019002375 |
| PRF1 | 8 | 0.019002375 |
| LRRC71 | 8 | 0.019002375 |
| OR2D2 | 8 | 0.019002375 |
| TBCD | 8 | 0.019002375 |
| STXBP5 | 8 | 0.019002375 |
| RSBN1 | 8 | 0.019002375 |
| GLP1R | 8 | 0.019002375 |
| OR10Z1 | 8 | 0.019002375 |
| ABHD18 | 8 | 0.019002375 |
| TSKS | 8 | 0.019002375 |
| ZNF224 | 8 | 0.019002375 |
| ACO2 | 8 | 0.019002375 |
| BEND4 | 8 | 0.019002375 |
| SEC31B | 8 | 0.019002375 |
| IL17RB | 8 | 0.019002375 |
| CD244 | 8 | 0.019002375 |
| GORASP1 | 8 | 0.019002375 |
| SAMD7 | 8 | 0.019002375 |
| LMF1 | 8 | 0.019002375 |
| CENPI | 8 | 0.019002375 |
| HLCS | 8 | 0.019002375 |
| ZNF354B | 8 | 0.019002375 |
| ZNF300 | 8 | 0.019002375 |
| IFT74 | 8 | 0.019002375 |
| ADAM9 | 8 | 0.019002375 |
| BTBD3 | 8 | 0.019002375 |
| BICRAL | 8 | 0.019002375 |
| PDE7A | 8 | 0.019002375 |
| LMBR1L | 8 | 0.019002375 |
| ZNF776 | 8 | 0.019002375 |
| PRKACG | 8 | 0.019002375 |
| TTC7B | 8 | 0.019002375 |
| PDSS1 | 8 | 0.019002375 |
| KPNA3 | 8 | 0.019002375 |
| GATB | 8 | 0.019002375 |
| GPRIN1 | 8 | 0.019002375 |
| TMOD2 | 8 | 0.019002375 |
| GJA1 | 8 | 0.019002375 |
| SYDE1 | 8 | 0.019002375 |
| HGS | 8 | 0.019002375 |
| F7 | 8 | 0.019002375 |
| NMD3 | 8 | 0.019002375 |
| GATA2 | 8 | 0.019002375 |
| OGT | 8 | 0.019002375 |
| SLC2A5 | 8 | 0.019002375 |
| EWSR1 | 8 | 0.019002375 |
| EPC2 | 8 | 0.019002375 |
| AMHR2 | 8 | 0.019002375 |
| EPS15L1 | 8 | 0.019002375 |
| MUC13 | 8 | 0.019002375 |
| CHRNA2 | 8 | 0.019002375 |
| ZNF606 | 8 | 0.019002375 |
| SGIP1 | 8 | 0.019002375 |
| GALNT10 | 8 | 0.019002375 |
| KIF11 | 8 | 0.019002375 |
| RSPO2 | 8 | 0.019002375 |
| SLC38A6 | 8 | 0.019002375 |
| HS3ST4 | 8 | 0.019002375 |
| STT3B | 8 | 0.019002375 |
| ALG10B | 8 | 0.019002375 |
| ANGPTL5 | 8 | 0.019002375 |
| ZNF750 | 8 | 0.019002375 |
| MRGPRX2 | 8 | 0.019002375 |
| PACSIN1 | 8 | 0.019002375 |
| RRAGB | 8 | 0.019002375 |
| KPNA4 | 8 | 0.019002375 |
| ARHGAP10 | 8 | 0.019002375 |
| CPNE9 | 8 | 0.019002375 |
| EFTUD2 | 8 | 0.019002375 |
| DYRK1A | 8 | 0.019002375 |
| TMEM175 | 8 | 0.019002375 |
| SHCBP1 | 8 | 0.019002375 |
| SLC39A7 | 8 | 0.019002375 |
| ENGASE | 8 | 0.019002375 |
| IL1R2 | 8 | 0.019002375 |
| DYRK4 | 8 | 0.019002375 |
| ZNF330 | 8 | 0.019002375 |
| SMC3 | 8 | 0.019002375 |
| KLHDC9 | 8 | 0.019002375 |
| RNF168 | 8 | 0.019002375 |
| ZFP3 | 8 | 0.019002375 |
| ZNF599 | 8 | 0.019002375 |
| KCNS1 | 8 | 0.019002375 |
| RAE1 | 8 | 0.019002375 |
| PPIE | 8 | 0.019002375 |
| BEST3 | 8 | 0.019002375 |
| CABYR | 8 | 0.019002375 |
| ICE2 | 8 | 0.019002375 |
| TBX15 | 8 | 0.019002375 |
| HPS3 | 8 | 0.019002375 |
| CHST4 | 8 | 0.019002375 |
| ZNF689 | 8 | 0.019002375 |
| PPP3CA | 8 | 0.019002375 |
| PCGF1 | 8 | 0.019002375 |
| FOXN2 | 8 | 0.019002375 |
| FMOD | 8 | 0.019002375 |
| B4GAT1 | 8 | 0.019002375 |
| RIC3 | 8 | 0.019002375 |
| RGS21 | 8 | 0.019002375 |
| GATAD2B | 8 | 0.019002375 |
| SERPINA10 | 8 | 0.019002375 |
| CDK16 | 8 | 0.019002375 |
| ZNF10 | 8 | 0.019002375 |
| GPR160 | 8 | 0.019002375 |
| RANGAP1 | 8 | 0.019002375 |
| NXF1 | 8 | 0.019002375 |
| SPANXN2 | 8 | 0.019002375 |
| ZNF768 | 8 | 0.019002375 |
| ANKRD24 | 8 | 0.019002375 |
| OR2M5 | 8 | 0.019002375 |
| IGSF5 | 8 | 0.019002375 |
| ARMT1 | 8 | 0.019002375 |
| ZNF433 | 8 | 0.019002375 |
| ERMN | 8 | 0.019002375 |
| MPEG1 | 8 | 0.019002375 |
| CATSPER4 | 8 | 0.019002375 |
| EXOC3L4 | 8 | 0.019002375 |
| WIPF2 | 8 | 0.019002375 |
| CLIC6 | 8 | 0.019002375 |
| THOP1 | 8 | 0.019002375 |
| GRK2 | 8 | 0.019002375 |
| VPS45 | 8 | 0.019002375 |
| PABPC1L | 8 | 0.019002375 |
| SFMBT1 | 8 | 0.019002375 |
| PPM1D | 8 | 0.019002375 |
| CCT6A | 8 | 0.019002375 |
| FUT2 | 8 | 0.019002375 |
| PHF24 | 8 | 0.019002375 |
| ZCRB1 | 8 | 0.019002375 |
| SPEM2 | 8 | 0.019002375 |
| GBP5 | 8 | 0.019002375 |
| GRIN3B | 8 | 0.019002375 |
| INSYN2A | 8 | 0.019002375 |
| NDST1 | 8 | 0.019002375 |
| GCDH | 8 | 0.019002375 |
| GAB3 | 8 | 0.019002375 |
| OR6C68 | 8 | 0.019002375 |
| SF3B2 | 8 | 0.019002375 |
| CSPG5 | 8 | 0.019002375 |
| PPARA | 8 | 0.019002375 |
| CCR7 | 8 | 0.019002375 |
| ZNF35 | 8 | 0.019002375 |
| FCHO1 | 8 | 0.019002375 |
| HSD3B1 | 8 | 0.019002375 |
| CD2AP | 8 | 0.019002375 |
| SDAD1 | 8 | 0.019002375 |
| TSPYL2 | 8 | 0.019002375 |
| SEMA4C | 8 | 0.019002375 |
| GPNMB | 8 | 0.019002375 |
| CEP97 | 8 | 0.019002375 |
| SLC2A8 | 8 | 0.019002375 |
| MAMDC2 | 8 | 0.019002375 |
| BRF2 | 8 | 0.019002375 |
| NYX | 8 | 0.019002375 |
| PYGL | 8 | 0.019002375 |
| TAS2R1 | 8 | 0.019002375 |
| SPIN1 | 8 | 0.019002375 |
| DNAJC3 | 8 | 0.019002375 |
| TFRC | 8 | 0.019002375 |
| PRAMEF7 | 8 | 0.019002375 |
| CD207 | 8 | 0.019002375 |
| ASPG | 8 | 0.019002375 |
| TBL2 | 8 | 0.019002375 |
| WNT3A | 8 | 0.019002375 |
| PLA2G3 | 8 | 0.019002375 |
| PDE1A | 8 | 0.019002375 |
| MOG | 8 | 0.019002375 |
| CPNE4 | 8 | 0.019002375 |
| BRD7 | 8 | 0.019002375 |
| NDOR1 | 8 | 0.019002375 |
| SDS | 8 | 0.019002375 |
| STEAP2 | 8 | 0.019002375 |
| CIR1 | 8 | 0.019002375 |
| FAM199X | 8 | 0.019002375 |
| RIT2 | 8 | 0.019002375 |
| TBC1D15 | 8 | 0.019002375 |
| LRCH1 | 8 | 0.019002375 |
| SHTN1 | 8 | 0.019002375 |
| VSIG10 | 8 | 0.019002375 |
| SEC23IP | 8 | 0.019002375 |
| L3MBTL2 | 8 | 0.019002375 |
| PIGB | 8 | 0.019002375 |
| KDM4C | 8 | 0.019002375 |
| SATL1 | 8 | 0.019002375 |
| MACROD2 | 8 | 0.019002375 |
| CSRNP1 | 8 | 0.019002375 |
| PSD3 | 8 | 0.019002375 |
| ELL3 | 8 | 0.019002375 |
| TRAF5 | 8 | 0.019002375 |
| FIGNL1 | 8 | 0.019002375 |
| QRSL1 | 8 | 0.019002375 |
| KCNAB3 | 8 | 0.019002375 |
| ST14 | 8 | 0.019002375 |
| PARP3 | 8 | 0.019002375 |
| FDPS | 8 | 0.019002375 |
| GPR87 | 8 | 0.019002375 |
| TAB1 | 8 | 0.019002375 |
| OR5M3 | 8 | 0.019002375 |
| GPR155 | 8 | 0.019002375 |
| PFKFB2 | 8 | 0.019002375 |
| DGCR8 | 8 | 0.019002375 |
| ABT1 | 8 | 0.019002375 |
| SCIN | 8 | 0.019002375 |
| PPP2R2A | 8 | 0.019002375 |
| NR1H4 | 8 | 0.019002375 |
| SH3KBP1 | 8 | 0.019002375 |
| ZNF280A | 8 | 0.019002375 |
| ACTRT2 | 8 | 0.019002375 |
| ZNF235 | 8 | 0.019002375 |
| AFM | 8 | 0.019002375 |
| GNAZ | 8 | 0.019002375 |
| NFX1 | 8 | 0.019002375 |
| LHX5 | 8 | 0.019002375 |
| TDRKH | 8 | 0.019002375 |
| ZNF185 | 8 | 0.019002375 |
| SP4 | 8 | 0.019002375 |
| OSBPL8 | 8 | 0.019002375 |
| TNFRSF21 | 8 | 0.019002375 |
| CDX2 | 8 | 0.019002375 |
| PSAPL1 | 8 | 0.019002375 |
| STK24 | 8 | 0.019002375 |
| AP4E1 | 8 | 0.019002375 |
| PACSIN2 | 8 | 0.019002375 |
| MTHFD2L | 8 | 0.019002375 |
| ZNF829 | 8 | 0.019002375 |
| AIDA | 8 | 0.019002375 |
| TMEM67 | 8 | 0.019002375 |
| ASMTL | 8 | 0.019002375 |
| OR10J3 | 8 | 0.019002375 |
| CHEK1 | 8 | 0.019002375 |
| RTL1 | 8 | 0.019002375 |
| F11 | 8 | 0.019002375 |
| GAS6 | 8 | 0.019002375 |
| DHX58 | 8 | 0.019002375 |
| EVX2 | 8 | 0.019002375 |
| ZNF319 | 8 | 0.019002375 |
| SPARC | 8 | 0.019002375 |
| PCSK4 | 8 | 0.019002375 |
| WNT7A | 8 | 0.019002375 |
| PGAP1 | 8 | 0.019002375 |
| GABRR1 | 8 | 0.019002375 |
| DNAJA4 | 8 | 0.019002375 |
| C17orf97 | 8 | 0.019002375 |
| FAM111B | 8 | 0.019002375 |
| SORT1 | 8 | 0.019002375 |
| GPR180 | 8 | 0.019002375 |
| DAG1 | 8 | 0.019002375 |
| PGLYRP4 | 8 | 0.019002375 |
| PHF8 | 8 | 0.019002375 |
| OR2T10 | 8 | 0.019002375 |
| AHCYL2 | 8 | 0.019002375 |
| ASAP3 | 8 | 0.019002375 |
| TBC1D24 | 8 | 0.019002375 |
| NACC1 | 8 | 0.019002375 |
| ACP2 | 8 | 0.019002375 |
| APOBEC4 | 8 | 0.019002375 |
| ATP13A4 | 8 | 0.019002375 |
| ABCD4 | 8 | 0.019002375 |
| APOA5 | 8 | 0.019002375 |
| NDC1 | 8 | 0.019002375 |
| FGF10 | 8 | 0.019002375 |
| CCDC30 | 8 | 0.019002375 |
| PRKRA | 8 | 0.019002375 |
| ZSCAN4 | 8 | 0.019002375 |
| FEZF1 | 8 | 0.019002375 |
| TANGO6 | 8 | 0.019002375 |
| OR5AS1 | 8 | 0.019002375 |
| LRRC32 | 8 | 0.019002375 |
| GABPA | 8 | 0.019002375 |
| TNFRSF8 | 8 | 0.019002375 |
| CALR3 | 8 | 0.019002375 |
| HIPK3 | 8 | 0.019002375 |
| SYNE4 | 8 | 0.019002375 |
| PPP1R32 | 8 | 0.019002375 |
| PIDD1 | 8 | 0.019002375 |
| METTL22 | 8 | 0.019002375 |
| PRAMEF14 | 8 | 0.019002375 |
| MAT2B | 8 | 0.019002375 |
| NBR1 | 8 | 0.019002375 |
| NKX6-1 | 8 | 0.019002375 |
| HTRA1 | 8 | 0.019002375 |
| ZMIZ2 | 8 | 0.019002375 |
| KCNK10 | 8 | 0.019002375 |
| RHAG | 8 | 0.019002375 |
| PSG7 | 8 | 0.019002375 |
| BTNL9 | 8 | 0.019002375 |
| FBXW5 | 8 | 0.019002375 |
| ARHGEF4 | 8 | 0.019002375 |
| MC3R | 8 | 0.019002375 |
| ISLR | 8 | 0.019002375 |
| KCNJ2 | 8 | 0.019002375 |
| SPANXD | 8 | 0.019002375 |
| TBC1D2 | 8 | 0.019002375 |
| CSTF2T | 8 | 0.019002375 |
| SPOCD1 | 8 | 0.019002375 |
| APOA4 | 8 | 0.019002375 |
| DCTN2 | 8 | 0.019002375 |
| SLC38A4 | 8 | 0.019002375 |
| B3GNT2 | 8 | 0.019002375 |
| PER2 | 8 | 0.019002375 |
| MST1 | 8 | 0.019002375 |
| CEP72 | 8 | 0.019002375 |
| ACSL5 | 8 | 0.019002375 |
| TF | 8 | 0.019002375 |
| HLTF | 8 | 0.019002375 |
| NUP42 | 8 | 0.019002375 |
| CD5L | 8 | 0.019002375 |
| ADGRG7 | 8 | 0.019002375 |
| PDCD6IP | 8 | 0.019002375 |
| MAU2 | 8 | 0.019002375 |
| XPO1 | 8 | 0.019002375 |
| SUPT3H | 8 | 0.019002375 |
| BPIFB4 | 8 | 0.019002375 |
| SHOC2 | 8 | 0.019002375 |
| UBTF | 8 | 0.019002375 |
| SP140L | 8 | 0.019002375 |
| LDHAL6B | 8 | 0.019002375 |
| HOXD4 | 8 | 0.019002375 |
| CNTFR | 8 | 0.019002375 |
| CASP4 | 8 | 0.019002375 |
| CHPF | 8 | 0.019002375 |
| SESN2 | 8 | 0.019002375 |
| NSFL1C | 8 | 0.019002375 |
| NTSR1 | 8 | 0.019002375 |
| AGFG1 | 8 | 0.019002375 |
| MIEF2 | 8 | 0.019002375 |
| CES5A | 8 | 0.019002375 |
| MAP2K2 | 8 | 0.019002375 |
| FBXL3 | 8 | 0.019002375 |
| SLC17A7 | 8 | 0.019002375 |
| ZNF17 | 8 | 0.019002375 |
| ZNF548 | 8 | 0.019002375 |
| MUL1 | 8 | 0.019002375 |
| CD70 | 8 | 0.019002375 |
| ARFGAP3 | 8 | 0.019002375 |
| TRIM49 | 8 | 0.019002375 |
| SNAP47 | 8 | 0.019002375 |
| MPP3 | 8 | 0.019002375 |
| KBTBD12 | 8 | 0.019002375 |
| ZNF33B | 8 | 0.019002375 |
| CYP2A6 | 8 | 0.019002375 |
| RAD18 | 8 | 0.019002375 |
| SLC28A1 | 8 | 0.019002375 |
| SMURF1 | 8 | 0.019002375 |
| OVGP1 | 8 | 0.019002375 |
| AURKC | 8 | 0.019002375 |
| ABAT | 8 | 0.019002375 |
| PLEKHG4 | 8 | 0.019002375 |
| CWF19L1 | 8 | 0.019002375 |
| KHDRBS3 | 8 | 0.019002375 |
| ZBP1 | 8 | 0.019002375 |
| CAVIN2 | 8 | 0.019002375 |
| PRAMEF2 | 8 | 0.019002375 |
| KLHL38 | 8 | 0.019002375 |
| OR6F1 | 8 | 0.019002375 |
| AJM1 | 8 | 0.019002375 |
| OR3A1 | 8 | 0.019002375 |
| GCNT1 | 8 | 0.019002375 |
| PAK1 | 8 | 0.019002375 |
| AMIGO2 | 8 | 0.019002375 |
| ACTBL2 | 8 | 0.019002375 |
| CRNN | 8 | 0.019002375 |
| XRCC4 | 8 | 0.019002375 |
| ZNF496 | 8 | 0.019002375 |
| DBR1 | 8 | 0.019002375 |
| FBXL19 | 8 | 0.019002375 |
| DNAJC1 | 8 | 0.019002375 |
| CIP2A | 8 | 0.019002375 |
| MFAP5 | 8 | 0.019002375 |
| TUBB2A | 8 | 0.019002375 |
| TAS2R42 | 8 | 0.019002375 |
| CNOT10 | 8 | 0.019002375 |
| SAMD3 | 8 | 0.019002375 |
| HPS6 | 8 | 0.019002375 |
| PPP1R12A | 8 | 0.019002375 |
| SNX31 | 8 | 0.019002375 |
| SYT14 | 8 | 0.019002375 |
| MAP6 | 8 | 0.019002375 |
| TRPC1 | 8 | 0.019002375 |
| PRPF31 | 8 | 0.019002375 |
| FAM189B | 8 | 0.019002375 |
| TRAM1 | 8 | 0.019002375 |
| ASB11 | 8 | 0.019002375 |
| PACRG | 8 | 0.019002375 |
| SLC20A2 | 8 | 0.019002375 |
| MROH8 | 8 | 0.019002375 |
| PLEKHM3 | 8 | 0.019002375 |
| TXNDC11 | 8 | 0.019002375 |
| FOXRED1 | 8 | 0.019002375 |
| TPH2 | 8 | 0.019002375 |
| AGPAT1 | 8 | 0.019002375 |
| TNNT2 | 8 | 0.019002375 |
| NFIA | 8 | 0.019002375 |
| NCOA7 | 8 | 0.019002375 |
| ZNF625 | 8 | 0.019002375 |
| TSPEAR | 8 | 0.019002375 |
| ANO9 | 8 | 0.019002375 |
| DNAJC21 | 8 | 0.019002375 |
| SERPINE1 | 8 | 0.019002375 |
| TRUB1 | 8 | 0.019002375 |
| RPUSD4 | 8 | 0.019002375 |
| TAS2R13 | 8 | 0.019002375 |
| LRRC47 | 8 | 0.019002375 |
| TRIM32 | 8 | 0.019002375 |
| SUPT20HL2 | 8 | 0.019002375 |
| APOBEC1 | 8 | 0.019002375 |
| LRRC8E | 8 | 0.019002375 |
| ZRANB2 | 8 | 0.019002375 |
| IWS1 | 8 | 0.019002375 |
| TPST2 | 8 | 0.019002375 |
| PSG2 | 8 | 0.019002375 |
| OR13C8 | 8 | 0.019002375 |
| CHRNA5 | 8 | 0.019002375 |
| CEACAM18 | 8 | 0.019002375 |
| C1orf127 | 8 | 0.019002375 |
| GABRE | 8 | 0.019002375 |
| VSTM2A | 8 | 0.019002375 |
| PRMT7 | 8 | 0.019002375 |
| SLC6A17 | 8 | 0.019002375 |
| MAN2B2 | 8 | 0.019002375 |
| RPL3L | 8 | 0.019002375 |
| TRIM41 | 8 | 0.019002375 |
| KLHDC8B | 8 | 0.019002375 |
| BTNL8 | 8 | 0.019002375 |
| TDO2 | 8 | 0.019002375 |
| HTR2B | 8 | 0.019002375 |
| BTK | 8 | 0.019002375 |
| MKX | 8 | 0.019002375 |
| CYP26A1 | 8 | 0.019002375 |
| PSMA8 | 8 | 0.019002375 |
| SPEM1 | 8 | 0.019002375 |
| EPAS1 | 8 | 0.019002375 |
| CYP4F22 | 8 | 0.019002375 |
| SLC6A4 | 8 | 0.019002375 |
| SLC17A9 | 8 | 0.019002375 |
| SLC38A1 | 8 | 0.019002375 |
| FBXW11 | 8 | 0.019002375 |
| EPS8L3 | 8 | 0.019002375 |
| ESX1 | 8 | 0.019002375 |
| GZMK | 8 | 0.019002375 |
| SLC25A2 | 8 | 0.019002375 |
| NIPA2 | 8 | 0.019002375 |
| OR4D5 | 8 | 0.019002375 |
| HSPA8 | 8 | 0.019002375 |
| GEM | 8 | 0.019002375 |
| MFSD2B | 8 | 0.019002375 |
| DGKE | 8 | 0.019002375 |
| KPNA1 | 8 | 0.019002375 |
| GMPPA | 8 | 0.019002375 |
| METTL6 | 8 | 0.019002375 |
| SYT4 | 8 | 0.019002375 |
| RBM12 | 8 | 0.019002375 |
| AL645922.1 | 8 | 0.019002375 |
| POGLUT2 | 8 | 0.019002375 |
| PLA2G7 | 8 | 0.019002375 |
| FCRL1 | 8 | 0.019002375 |
| PSEN2 | 8 | 0.019002375 |
| CCNT2 | 8 | 0.019002375 |
| ARMCX5 | 8 | 0.019002375 |
| DNAJC11 | 8 | 0.019002375 |
| DNAJC7 | 8 | 0.019002375 |
| RNF19A | 8 | 0.019002375 |
| OR2G6 | 8 | 0.019002375 |
| GTPBP3 | 8 | 0.019002375 |
| PLOD1 | 8 | 0.019002375 |
| TMEM25 | 8 | 0.019002375 |
| ZNF287 | 8 | 0.019002375 |
| INPP5E | 8 | 0.019002375 |
| ROPN1B | 8 | 0.019002375 |
| KRT28 | 8 | 0.019002375 |
| HCN2 | 8 | 0.019002375 |
| EVI5L | 8 | 0.019002375 |
| FFAR3 | 8 | 0.019002375 |
| EIF2B5 | 8 | 0.019002375 |
| SMOC1 | 8 | 0.019002375 |
| ZNF624 | 8 | 0.019002375 |
| NTN1 | 8 | 0.019002375 |
| TTLL3 | 8 | 0.019002375 |
| RBMS3 | 8 | 0.019002375 |
| RAB3GAP2 | 8 | 0.019002375 |
| MEF2A | 8 | 0.019002375 |
| FAAP100 | 8 | 0.019002375 |
| DHTKD1 | 8 | 0.019002375 |
| GUSB | 8 | 0.019002375 |
| VPS35L | 8 | 0.019002375 |
| GLCE | 8 | 0.019002375 |
| MTMR7 | 8 | 0.019002375 |
| ARHGAP18 | 8 | 0.019002375 |
| ILVBL | 8 | 0.019002375 |
| TRIM15 | 8 | 0.019002375 |
| SOCS6 | 8 | 0.019002375 |
| CDKL2 | 8 | 0.019002375 |
| PPP2R5E | 8 | 0.019002375 |
| PCNX4 | 8 | 0.019002375 |
| UHRF1 | 8 | 0.019002375 |
| FAR2 | 8 | 0.019002375 |
| TRAF3IP1 | 8 | 0.019002375 |
| CDH24 | 8 | 0.019002375 |
| MYRF | 8 | 0.019002375 |
| ABCB7 | 8 | 0.019002375 |
| NOC3L | 8 | 0.019002375 |
| DGKK | 8 | 0.019002375 |
| SLC35G3 | 8 | 0.019002375 |
| PUS3 | 8 | 0.019002375 |
| CCDC38 | 8 | 0.019002375 |
| HOOK1 | 8 | 0.019002375 |
| UGT1A7 | 8 | 0.019002375 |
| IGF2BP3 | 8 | 0.019002375 |
| ZNF311 | 8 | 0.019002375 |
| MTMR2 | 8 | 0.019002375 |
| CEP55 | 8 | 0.019002375 |
| LRP2BP | 8 | 0.019002375 |
| EIF3J | 8 | 0.019002375 |
| HEATR6 | 8 | 0.019002375 |
| TMEM8B | 8 | 0.019002375 |
| ZNF408 | 8 | 0.019002375 |
| SASH3 | 8 | 0.019002375 |
| ZNF649 | 8 | 0.019002375 |
| PPP3CB | 8 | 0.019002375 |
| LTK | 8 | 0.019002375 |
| CTBP1 | 8 | 0.019002375 |
| SLC17A8 | 8 | 0.019002375 |
| IFRD2 | 8 | 0.019002375 |
| OR56A3 | 8 | 0.019002375 |
| CYP27B1 | 8 | 0.019002375 |
| USP16 | 8 | 0.019002375 |
| MMP10 | 8 | 0.019002375 |
| ISYNA1 | 8 | 0.019002375 |
| JMY | 8 | 0.019002375 |
| CCDC138 | 8 | 0.019002375 |
| SEC14L3 | 8 | 0.019002375 |
| ALPP | 8 | 0.019002375 |
| MPHOSPH9 | 8 | 0.019002375 |
| PLA2G4E | 8 | 0.019002375 |
| ASB2 | 8 | 0.019002375 |
| AMMECR1L | 8 | 0.019002375 |
| PDE6B | 8 | 0.019002375 |
| RAP1GDS1 | 8 | 0.019002375 |
| SKOR1 | 8 | 0.019002375 |
| SUCLA2 | 8 | 0.019002375 |
| VGLL4 | 8 | 0.019002375 |
| WIPI2 | 8 | 0.019002375 |
| ZNF385B | 8 | 0.019002375 |
| ZNF219 | 8 | 0.019002375 |
| MYOD1 | 8 | 0.019002375 |
| GALNT5 | 8 | 0.019002375 |
| MMP15 | 8 | 0.019002375 |
| ACO1 | 8 | 0.019002375 |
| ZNF124 | 8 | 0.019002375 |
| AIFM1 | 8 | 0.019002375 |
| METTL16 | 8 | 0.019002375 |
| HLA-DRA | 8 | 0.019002375 |
| PPEF2 | 8 | 0.019002375 |
| ATAT1 | 8 | 0.019002375 |
| TIGD7 | 8 | 0.019002375 |
| RBM47 | 8 | 0.019002375 |
| GSX2 | 8 | 0.019002375 |
| TNFRSF11B | 8 | 0.019002375 |
| HHAT | 8 | 0.019002375 |
| HTR3A | 8 | 0.019002375 |
| FAM217A | 8 | 0.019002375 |
| SERPINB10 | 8 | 0.019002375 |
| SLC39A5 | 8 | 0.019002375 |
| MTMR9 | 8 | 0.019002375 |
| ARHGEF37 | 8 | 0.019002375 |
| ALDOB | 8 | 0.019002375 |
| STAT2 | 8 | 0.019002375 |
| COP1 | 8 | 0.019002375 |
| NECAB3 | 8 | 0.019002375 |
| DUXA | 8 | 0.019002375 |
| ADGRF3 | 8 | 0.019002375 |
| STRADA | 8 | 0.019002375 |
| TTC30B | 8 | 0.019002375 |
| HADHA | 8 | 0.019002375 |
| NACC2 | 8 | 0.019002375 |
| UBXN7 | 8 | 0.019002375 |
| SERPINA12 | 8 | 0.019002375 |
| VWA2 | 8 | 0.019002375 |
| PLA2G4F | 8 | 0.019002375 |
| MYC | 8 | 0.019002375 |
| NR5A1 | 8 | 0.019002375 |
| RAB11FIP4 | 8 | 0.019002375 |
| SESTD1 | 8 | 0.019002375 |
| SNX32 | 8 | 0.019002375 |
| DNMT3A | 8 | 0.019002375 |
| ITIH3 | 8 | 0.019002375 |
| NFIC | 8 | 0.019002375 |
| ABI3 | 8 | 0.019002375 |
| TBRG4 | 8 | 0.019002375 |
| VSIR | 8 | 0.019002375 |
| FAM124A | 8 | 0.019002375 |
| PPP1R18 | 8 | 0.019002375 |
| PPP1R36 | 8 | 0.019002375 |
| STK3 | 8 | 0.019002375 |
| IDH3G | 8 | 0.019002375 |
| UBASH3B | 8 | 0.019002375 |
| FUT9 | 8 | 0.019002375 |
| TRPM5 | 8 | 0.019002375 |
| HESX1 | 8 | 0.019002375 |
| WDR70 | 8 | 0.019002375 |
| PSG8 | 8 | 0.019002375 |
| ZNF514 | 8 | 0.019002375 |
| MAEA | 8 | 0.019002375 |
| KLHL26 | 8 | 0.019002375 |
| UBQLNL | 8 | 0.019002375 |
| PROSER3 | 8 | 0.019002375 |
| FBXL13 | 8 | 0.019002375 |
| TRIM50 | 8 | 0.019002375 |
| CACTIN | 8 | 0.019002375 |
| SLC2A3 | 8 | 0.019002375 |
| BAG3 | 8 | 0.019002375 |
| TTYH2 | 8 | 0.019002375 |
| CETP | 8 | 0.019002375 |
| SLC2A10 | 8 | 0.019002375 |
| OSGEPL1 | 8 | 0.019002375 |
| PIK3CB | 8 | 0.019002375 |
| DHODH | 8 | 0.019002375 |
| SAXO2 | 8 | 0.019002375 |
| MMUT | 8 | 0.019002375 |
| SLC9A8 | 8 | 0.019002375 |
| SERPINB7 | 8 | 0.019002375 |
| ZW10 | 8 | 0.019002375 |
| KRTAP10-6 | 8 | 0.019002375 |
| LHX6 | 8 | 0.019002375 |
| GCLC | 8 | 0.019002375 |
| SLC6A12 | 8 | 0.019002375 |
| GPT | 8 | 0.019002375 |
| GRK3 | 8 | 0.019002375 |
| STK33 | 8 | 0.019002375 |
| OR2L2 | 8 | 0.019002375 |
| CNOT3 | 8 | 0.019002375 |
| HCK | 8 | 0.019002375 |
| DMPK | 8 | 0.019002375 |
| ZFX | 8 | 0.019002375 |
| CEP89 | 8 | 0.019002375 |
| GPR171 | 8 | 0.019002375 |
| PHF7 | 8 | 0.019002375 |
| C1orf112 | 8 | 0.019002375 |
| SRPK3 | 8 | 0.019002375 |
| NR4A3 | 8 | 0.019002375 |
| ATXN3L | 8 | 0.019002375 |
| RANBP9 | 8 | 0.019002375 |
| ZNF438 | 8 | 0.019002375 |
| GZMA | 8 | 0.019002375 |
| FERMT2 | 8 | 0.019002375 |
| MRAP2 | 8 | 0.019002375 |
| MCHR1 | 8 | 0.019002375 |
| TAP1 | 8 | 0.019002375 |
| SCYL1 | 8 | 0.019002375 |
| CLCNKB | 8 | 0.019002375 |
| CCDC82 | 8 | 0.019002375 |
| UNC5CL | 8 | 0.019002375 |
| LPL | 8 | 0.019002375 |
| TCIRG1 | 8 | 0.019002375 |
| FEM1A | 8 | 0.019002375 |
| CAPRIN1 | 8 | 0.019002375 |
| MFAP3L | 8 | 0.019002375 |
| CYP2C19 | 8 | 0.019002375 |
| UTP25 | 8 | 0.019002375 |
| OSCP1 | 8 | 0.019002375 |
| CHST6 | 8 | 0.019002375 |
| LLGL2 | 8 | 0.019002375 |
| CEP41 | 8 | 0.019002375 |
| URI1 | 8 | 0.019002375 |
| TXK | 8 | 0.019002375 |
| DDX1 | 8 | 0.019002375 |
| GPR3 | 8 | 0.019002375 |
| ADRB3 | 8 | 0.019002375 |
| PPAT | 8 | 0.019002375 |
| TCFL5 | 8 | 0.019002375 |
| PIK3AP1 | 8 | 0.019002375 |
| OR10J5 | 8 | 0.019002375 |
| SIRT4 | 8 | 0.019002375 |
| AADAC | 8 | 0.019002375 |
| HOXB3 | 8 | 0.019002375 |
| OR8G2P | 8 | 0.019002375 |
| CENPU | 8 | 0.019002375 |
| CCSER1 | 8 | 0.019002375 |
| KCNJ10 | 8 | 0.019002375 |
| MARCHF7 | 8 | 0.019002375 |
| THNSL1 | 8 | 0.019002375 |
| ZNF280B | 8 | 0.019002375 |
| ACSM2B | 8 | 0.019002375 |
| AMZ1 | 8 | 0.019002375 |
| NEK3 | 8 | 0.019002375 |
| THADA | 8 | 0.019002375 |
| PLEKHN1 | 8 | 0.019002375 |
| RAB40C | 8 | 0.019002375 |
| TMPRSS2 | 8 | 0.019002375 |
| UNKL | 8 | 0.019002375 |
| HTRA3 | 8 | 0.019002375 |
| USPL1 | 8 | 0.019002375 |
| TRIM23 | 8 | 0.019002375 |
| DDIAS | 8 | 0.019002375 |
| HTR3E | 8 | 0.019002375 |
| SBSN | 8 | 0.019002375 |
| NDC80 | 8 | 0.019002375 |
| PLA2G4D | 8 | 0.019002375 |
| GPCPD1 | 8 | 0.019002375 |
| FAM200A | 8 | 0.019002375 |
| BMP2K | 8 | 0.019002375 |
| ZC2HC1C | 8 | 0.019002375 |
| KPNA6 | 8 | 0.019002375 |
| LMX1A | 8 | 0.019002375 |
| CHRNB3 | 8 | 0.019002375 |
| KRT33B | 8 | 0.019002375 |
| METTL3 | 8 | 0.019002375 |
| ARID4B | 8 | 0.019002375 |
| CCDC125 | 8 | 0.019002375 |
| EML2 | 8 | 0.019002375 |
| SIGLEC9 | 8 | 0.019002375 |
| RNF148 | 8 | 0.019002375 |
| NPAS2 | 8 | 0.019002375 |
| ONECUT1 | 8 | 0.019002375 |
| EMILIN2 | 8 | 0.019002375 |
| PNPLA3 | 8 | 0.019002375 |
| FRS3 | 8 | 0.019002375 |
| NEUROD1 | 8 | 0.019002375 |
| CRY2 | 8 | 0.019002375 |
| SIGLEC6 | 8 | 0.019002375 |
| NADSYN1 | 8 | 0.019002375 |
| PTDSS1 | 8 | 0.019002375 |
| OR51B5 | 8 | 0.019002375 |
| CDH5 | 8 | 0.019002375 |
| MAGEC2 | 8 | 0.019002375 |
| PRDM13 | 8 | 0.019002375 |
| CHST11 | 8 | 0.019002375 |
| NR2F2 | 8 | 0.019002375 |
| CDK1 | 8 | 0.019002375 |
| SLC35G2 | 8 | 0.019002375 |
| RIPK1 | 8 | 0.019002375 |
| DYNC1LI1 | 8 | 0.019002375 |
| CXCR2 | 8 | 0.019002375 |
| ZNF154 | 8 | 0.019002375 |
| SPATS2 | 8 | 0.019002375 |
| SCNN1G | 8 | 0.019002375 |
| BBS7 | 8 | 0.019002375 |
| LBR | 8 | 0.019002375 |
| DCBLD2 | 8 | 0.019002375 |
| CDRT15 | 8 | 0.019002375 |
| HJURP | 8 | 0.019002375 |
| RUVBL1 | 8 | 0.019002375 |
| OR14K1 | 8 | 0.019002375 |
| LUC7L3 | 8 | 0.019002375 |
| IRAK4 | 8 | 0.019002375 |
| ZBTB26 | 8 | 0.019002375 |
| CACNG7 | 8 | 0.019002375 |
| SCG3 | 8 | 0.019002375 |
| VAV2 | 8 | 0.019002375 |
| OR6K6 | 8 | 0.019002375 |
| ENTR1 | 8 | 0.019002375 |
| IGSF11 | 8 | 0.019002375 |
| BCAN | 8 | 0.019002375 |
| AFTPH | 8 | 0.019002375 |
| GGA3 | 8 | 0.019002375 |
| PON3 | 8 | 0.019002375 |
| ZNF678 | 8 | 0.019002375 |
| REL | 8 | 0.019002375 |
| TRIM6-TRIM34 | 8 | 0.019002375 |
| A4GALT | 8 | 0.019002375 |
| MAN1A2 | 8 | 0.019002375 |
| STAT4 | 8 | 0.019002375 |
| CACUL1 | 8 | 0.019002375 |
| JPH2 | 8 | 0.019002375 |
| SUGP1 | 8 | 0.019002375 |
| PPP1R13L | 8 | 0.019002375 |
| PTGS1 | 8 | 0.019002375 |
| SLC7A4 | 8 | 0.019002375 |
| MSC | 8 | 0.019002375 |
| DNAJB7 | 8 | 0.019002375 |
| SLMAP | 8 | 0.019002375 |
| ART1 | 8 | 0.019002375 |
| ZNF556 | 8 | 0.019002375 |
| CES1 | 8 | 0.019002375 |
| CHRM4 | 8 | 0.019002375 |
| PRMT3 | 8 | 0.019002375 |
| EZHIP | 8 | 0.019002375 |
| GPR37L1 | 8 | 0.019002375 |
| CAVIN4 | 8 | 0.019002375 |
| AC008397.2 | 8 | 0.019002375 |
| TBC1D2B | 8 | 0.019002375 |
| GXYLT1 | 8 | 0.019002375 |
| MIOS | 8 | 0.019002375 |
| ARHGAP19 | 8 | 0.019002375 |
| SLA | 8 | 0.019002375 |
| DAZAP1 | 8 | 0.019002375 |
| LONP1 | 8 | 0.019002375 |
| ABCG1 | 8 | 0.019002375 |
| CLUAP1 | 8 | 0.019002375 |
| HS3ST3B1 | 8 | 0.019002375 |
| GALR1 | 8 | 0.019002375 |
| SPRED1 | 8 | 0.019002375 |
| KHNYN | 8 | 0.019002375 |
| TSPYL4 | 8 | 0.019002375 |
| DEAF1 | 8 | 0.019002375 |
| OSBP | 8 | 0.019002375 |
| PTCD1 | 8 | 0.019002375 |
| KRTAP27-1 | 8 | 0.019002375 |
| HEPH | 8 | 0.019002375 |
| ZNF628 | 8 | 0.019002375 |
| RARA | 8 | 0.019002375 |
| GPR142 | 8 | 0.019002375 |
| FAM171A1 | 8 | 0.019002375 |
| LIPT1 | 8 | 0.019002375 |
| PTPN9 | 8 | 0.019002375 |
| ACSF3 | 8 | 0.019002375 |
| OLFM4 | 8 | 0.019002375 |
| CCDC34 | 8 | 0.019002375 |
| FKTN | 8 | 0.019002375 |
| ZNF654 | 8 | 0.019002375 |
| PHOX2B | 8 | 0.019002375 |
| ANGPT1 | 8 | 0.019002375 |
| RANBP3L | 8 | 0.019002375 |
| SRP72 | 8 | 0.019002375 |
| CCNL2 | 8 | 0.019002375 |
| LPAR4 | 8 | 0.019002375 |
| KCNJ12 | 8 | 0.019002375 |
| CSNK2A1 | 8 | 0.019002375 |
| MLXIPL | 8 | 0.019002375 |
| KLHL3 | 8 | 0.019002375 |
| FANCL | 8 | 0.019002375 |
| HNRNPM | 8 | 0.019002375 |
| NLN | 8 | 0.019002375 |
| INTS11 | 8 | 0.019002375 |
| LIMD1 | 8 | 0.019002375 |
| STEAP4 | 8 | 0.019002375 |
| RPAP1 | 8 | 0.019002375 |
| CADM2 | 8 | 0.019002375 |
| OR4A47 | 8 | 0.019002375 |
| TAF15 | 8 | 0.019002375 |
| CNOT7 | 8 | 0.019002375 |
| GSTCD | 8 | 0.019002375 |
| CCDC105 | 8 | 0.019002375 |
| DNAJC22 | 8 | 0.019002375 |
| BCL6B | 8 | 0.019002375 |
| SF3A1 | 8 | 0.019002375 |
| TMEM161B | 8 | 0.019002375 |
| MXD3 | 8 | 0.019002375 |
| DNAJA1 | 8 | 0.019002375 |
| AACS | 8 | 0.019002375 |
| ANO10 | 8 | 0.019002375 |
| KRT9 | 8 | 0.019002375 |
| AC098582.1 | 8 | 0.019002375 |
| ADHFE1 | 8 | 0.019002375 |
| AADACL2 | 8 | 0.019002375 |
| OPN4 | 8 | 0.019002375 |
| LRFN4 | 8 | 0.019002375 |
| GBP7 | 8 | 0.019002375 |
| SGCE | 8 | 0.019002375 |
| SEC24A | 8 | 0.019002375 |
| YY1AP1 | 8 | 0.019002375 |
| HNF1B | 8 | 0.019002375 |
| KIF19 | 8 | 0.019002375 |
| NEPRO | 8 | 0.019002375 |
| NPSR1 | 8 | 0.019002375 |
| EFNB1 | 8 | 0.019002375 |
| SLC22A2 | 8 | 0.019002375 |
| NTAN1 | 8 | 0.019002375 |
| PRKAA1 | 8 | 0.019002375 |
| SPTLC1 | 8 | 0.019002375 |
| OR13C4 | 8 | 0.019002375 |
| GPR141 | 8 | 0.019002375 |
| SLC12A4 | 8 | 0.019002375 |
| LGI1 | 8 | 0.019002375 |
| FSCN2 | 8 | 0.019002375 |
| CD300LG | 8 | 0.019002375 |
| LHX9 | 8 | 0.019002375 |
| COG5 | 8 | 0.019002375 |
| BCL11B | 8 | 0.019002375 |
| GNRHR | 8 | 0.019002375 |
| SMPD2 | 8 | 0.019002375 |
| GHSR | 8 | 0.019002375 |
| SPRED2 | 8 | 0.019002375 |
| ZNF605 | 8 | 0.019002375 |
| HSPH1 | 8 | 0.019002375 |
| UGT2A3 | 8 | 0.019002375 |
| PCGF2 | 8 | 0.019002375 |
| FOXN1 | 8 | 0.019002375 |
| CCR5 | 8 | 0.019002375 |
| ACTG2 | 8 | 0.019002375 |
| RIPOR3 | 8 | 0.019002375 |
| LRFN3 | 8 | 0.019002375 |
| SSTR1 | 8 | 0.019002375 |
| SETD3 | 8 | 0.019002375 |
| SLC41A3 | 8 | 0.019002375 |
| HGSNAT | 8 | 0.019002375 |
| PRLR | 8 | 0.019002375 |
| EBF2 | 8 | 0.019002375 |
| ARSA | 8 | 0.019002375 |
| NCAPG2 | 8 | 0.019002375 |
| OR52E4 | 8 | 0.019002375 |
| HDGFL1 | 8 | 0.019002375 |
| SFXN2 | 8 | 0.019002375 |
| GALNT9 | 8 | 0.019002375 |
| ZNF497 | 8 | 0.019002375 |
| ANO1 | 8 | 0.019002375 |
| RALYL | 8 | 0.019002375 |
| ZNF197 | 8 | 0.019002375 |
| INTS6 | 8 | 0.019002375 |
| MTPAP | 8 | 0.019002375 |
| TMEM44 | 8 | 0.019002375 |
| RBM43 | 8 | 0.019002375 |
| OR52J3 | 8 | 0.019002375 |
| VASN | 8 | 0.019002375 |
| MRPL39 | 8 | 0.019002375 |
| RELB | 8 | 0.019002375 |
| FBLN7 | 8 | 0.019002375 |
| LRIT2 | 8 | 0.019002375 |
| DDB1 | 8 | 0.019002375 |
| OR12D2 | 8 | 0.019002375 |
| UTP14A | 8 | 0.019002375 |
| PPHLN1 | 8 | 0.019002375 |
| STC2 | 8 | 0.019002375 |
| MSI1 | 8 | 0.019002375 |
| FAM83D | 7 | 0.016627078 |
| ANAPC5 | 7 | 0.016627078 |
| GPR52 | 7 | 0.016627078 |
| KDM4D | 7 | 0.016627078 |
| SLC25A4 | 7 | 0.016627078 |
| RNF113B | 7 | 0.016627078 |
| GTF2A1 | 7 | 0.016627078 |
| HOXA1 | 7 | 0.016627078 |
| ZNF816 | 7 | 0.016627078 |
| CD200R1L | 7 | 0.016627078 |
| LAMP1 | 7 | 0.016627078 |
| OR6B2 | 7 | 0.016627078 |
| ENTPD2 | 7 | 0.016627078 |
| CAPZB | 7 | 0.016627078 |
| CYTIP | 7 | 0.016627078 |
| BTN1A1 | 7 | 0.016627078 |
| AHCY | 7 | 0.016627078 |
| LHX2 | 7 | 0.016627078 |
| PGK2 | 7 | 0.016627078 |
| OR10X1 | 7 | 0.016627078 |
| PLCXD3 | 7 | 0.016627078 |
| FADS3 | 7 | 0.016627078 |
| YARS2 | 7 | 0.016627078 |
| TLR1 | 7 | 0.016627078 |
| FOLR3 | 7 | 0.016627078 |
| THEMIS2 | 7 | 0.016627078 |
| CLCC1 | 7 | 0.016627078 |
| CCNB1 | 7 | 0.016627078 |
| UBXN6 | 7 | 0.016627078 |
| ATF6B | 7 | 0.016627078 |
| SEMA4B | 7 | 0.016627078 |
| PIGK | 7 | 0.016627078 |
| ZNF665 | 7 | 0.016627078 |
| ZNF302 | 7 | 0.016627078 |
| TYRO3 | 7 | 0.016627078 |
| STAU1 | 7 | 0.016627078 |
| ANKLE1 | 7 | 0.016627078 |
| HMGCS2 | 7 | 0.016627078 |
| LRRC56 | 7 | 0.016627078 |
| ST6GALNAC5 | 7 | 0.016627078 |
| CPE | 7 | 0.016627078 |
| ATOH1 | 7 | 0.016627078 |
| NMT1 | 7 | 0.016627078 |
| MECP2 | 7 | 0.016627078 |
| ZNF286A | 7 | 0.016627078 |
| CASQ2 | 7 | 0.016627078 |
| GJD4 | 7 | 0.016627078 |
| ABCB10 | 7 | 0.016627078 |
| MTG1 | 7 | 0.016627078 |
| SLC36A4 | 7 | 0.016627078 |
| PRSS37 | 7 | 0.016627078 |
| BCL3 | 7 | 0.016627078 |
| BTNL3 | 7 | 0.016627078 |
| SPNS1 | 7 | 0.016627078 |
| SLF1 | 7 | 0.016627078 |
| DMRT2 | 7 | 0.016627078 |
| GPR161 | 7 | 0.016627078 |
| DCSTAMP | 7 | 0.016627078 |
| CAMK2B | 7 | 0.016627078 |
| USO1 | 7 | 0.016627078 |
| EPHA1 | 7 | 0.016627078 |
| HMGCR | 7 | 0.016627078 |
| MFSD14B | 7 | 0.016627078 |
| ERLIN2 | 7 | 0.016627078 |
| TARS3 | 7 | 0.016627078 |
| PLCD4 | 7 | 0.016627078 |
| SHMT1 | 7 | 0.016627078 |
| ZNF598 | 7 | 0.016627078 |
| SLC17A3 | 7 | 0.016627078 |
| EGFL6 | 7 | 0.016627078 |
| OR6C70 | 7 | 0.016627078 |
| AAAS | 7 | 0.016627078 |
| GPR68 | 7 | 0.016627078 |
| SPIRE2 | 7 | 0.016627078 |
| NADK | 7 | 0.016627078 |
| ZSWIM2 | 7 | 0.016627078 |
| CLEC5A | 7 | 0.016627078 |
| CHRNA3 | 7 | 0.016627078 |
| TRMT10A | 7 | 0.016627078 |
| ZNF671 | 7 | 0.016627078 |
| CTU2 | 7 | 0.016627078 |
| NANOS2 | 7 | 0.016627078 |
| NUDCD1 | 7 | 0.016627078 |
| TGFB1I1 | 7 | 0.016627078 |
| PACSIN3 | 7 | 0.016627078 |
| BLZF1 | 7 | 0.016627078 |
| UBE2U | 7 | 0.016627078 |
| CLINT1 | 7 | 0.016627078 |
| REC8 | 7 | 0.016627078 |
| FGF12 | 7 | 0.016627078 |
| DDX19A | 7 | 0.016627078 |
| RHPN2 | 7 | 0.016627078 |
| GDF9 | 7 | 0.016627078 |
| IREB2 | 7 | 0.016627078 |
| ST8SIA5 | 7 | 0.016627078 |
| FAM90A1 | 7 | 0.016627078 |
| IGSF21 | 7 | 0.016627078 |
| SIGLEC14 | 7 | 0.016627078 |
| SYTL1 | 7 | 0.016627078 |
| NAB1 | 7 | 0.016627078 |
| AMBN | 7 | 0.016627078 |
| CCDC146 | 7 | 0.016627078 |
| ELP4 | 7 | 0.016627078 |
| BFAR | 7 | 0.016627078 |
| PDGFA | 7 | 0.016627078 |
| ZNF534 | 7 | 0.016627078 |
| C1orf100 | 7 | 0.016627078 |
| LRRC18 | 7 | 0.016627078 |
| IVNS1ABP | 7 | 0.016627078 |
| KRT76 | 7 | 0.016627078 |
| GEN1 | 7 | 0.016627078 |
| SLC22A13 | 7 | 0.016627078 |
| PNMA2 | 7 | 0.016627078 |
| CCDC17 | 7 | 0.016627078 |
| NAAA | 7 | 0.016627078 |
| ZDHHC16 | 7 | 0.016627078 |
| ACTB | 7 | 0.016627078 |
| BIN3 | 7 | 0.016627078 |
| KCTD11 | 7 | 0.016627078 |
| GPRIN3 | 7 | 0.016627078 |
| DYNC2LI1 | 7 | 0.016627078 |
| SP6 | 7 | 0.016627078 |
| CXCR1 | 7 | 0.016627078 |
| EPN2 | 7 | 0.016627078 |
| GLA | 7 | 0.016627078 |
| ZNF341 | 7 | 0.016627078 |
| OR51Q1 | 7 | 0.016627078 |
| IFNLR1 | 7 | 0.016627078 |
| MMP11 | 7 | 0.016627078 |
| SRSF11 | 7 | 0.016627078 |
| XYLT1 | 7 | 0.016627078 |
| KDM1B | 7 | 0.016627078 |
| PCYT1B | 7 | 0.016627078 |
| VPS37C | 7 | 0.016627078 |
| ZNF136 | 7 | 0.016627078 |
| SESN3 | 7 | 0.016627078 |
| TMX3 | 7 | 0.016627078 |
| PGAP6 | 7 | 0.016627078 |
| TPCN2 | 7 | 0.016627078 |
| AC092143.1 | 7 | 0.016627078 |
| LTBR | 7 | 0.016627078 |
| ZNF436 | 7 | 0.016627078 |
| PLD3 | 7 | 0.016627078 |
| SLC28A2 | 7 | 0.016627078 |
| MAML2 | 7 | 0.016627078 |
| SPOUT1 | 7 | 0.016627078 |
| COL8A2 | 7 | 0.016627078 |
| GDF3 | 7 | 0.016627078 |
| HORMAD1 | 7 | 0.016627078 |
| LRRC8A | 7 | 0.016627078 |
| PAX7 | 7 | 0.016627078 |
| PDLIM7 | 7 | 0.016627078 |
| KLK13 | 7 | 0.016627078 |
| MFHAS1 | 7 | 0.016627078 |
| NAGPA | 7 | 0.016627078 |
| ACAP2 | 7 | 0.016627078 |
| SLC5A9 | 7 | 0.016627078 |
| MX2 | 7 | 0.016627078 |
| MARCHF6 | 7 | 0.016627078 |
| GALNS | 7 | 0.016627078 |
| STRA8 | 7 | 0.016627078 |
| RUBCNL | 7 | 0.016627078 |
| SERPINB12 | 7 | 0.016627078 |
| OR56A4 | 7 | 0.016627078 |
| FBLIM1 | 7 | 0.016627078 |
| TMPRSS13 | 7 | 0.016627078 |
| ACADSB | 7 | 0.016627078 |
| OR2T8 | 7 | 0.016627078 |
| ZBED8 | 7 | 0.016627078 |
| MFSD12 | 7 | 0.016627078 |
| CARS2 | 7 | 0.016627078 |
| STAT6 | 7 | 0.016627078 |
| MFSD6 | 7 | 0.016627078 |
| OR6C65 | 7 | 0.016627078 |
| OR5AN1 | 7 | 0.016627078 |
| KRTAP10-7 | 7 | 0.016627078 |
| FBXO15 | 7 | 0.016627078 |
| MPIG6B | 7 | 0.016627078 |
| PRAME | 7 | 0.016627078 |
| TATDN2 | 7 | 0.016627078 |
| RALBP1 | 7 | 0.016627078 |
| MAGEE2 | 7 | 0.016627078 |
| MSL2 | 7 | 0.016627078 |
| DCAF11 | 7 | 0.016627078 |
| PCSK9 | 7 | 0.016627078 |
| PTHLH | 7 | 0.016627078 |
| CHRNA1 | 7 | 0.016627078 |
| VPS4A | 7 | 0.016627078 |
| ORC2 | 7 | 0.016627078 |
| SHISAL1 | 7 | 0.016627078 |
| OBI1 | 7 | 0.016627078 |
| MMP25 | 7 | 0.016627078 |
| CBLIF | 7 | 0.016627078 |
| FBXO42 | 7 | 0.016627078 |
| UGT2B10 | 7 | 0.016627078 |
| CHFR | 7 | 0.016627078 |
| UTP14C | 7 | 0.016627078 |
| JPT2 | 7 | 0.016627078 |
| OR10S1 | 7 | 0.016627078 |
| FBXO31 | 7 | 0.016627078 |
| PSMD11 | 7 | 0.016627078 |
| PPAN-P2RY11 | 7 | 0.016627078 |
| SNTB1 | 7 | 0.016627078 |
| GALNT15 | 7 | 0.016627078 |
| REM1 | 7 | 0.016627078 |
| FCRL4 | 7 | 0.016627078 |
| THSD1 | 7 | 0.016627078 |
| FAM163B | 7 | 0.016627078 |
| PSAP | 7 | 0.016627078 |
| DCLRE1A | 7 | 0.016627078 |
| PCSK2 | 7 | 0.016627078 |
| AGTR2 | 7 | 0.016627078 |
| NKPD1 | 7 | 0.016627078 |
| C7orf50 | 7 | 0.016627078 |
| GCK | 7 | 0.016627078 |
| ZBTB39 | 7 | 0.016627078 |
| GPR61 | 7 | 0.016627078 |
| GSK3A | 7 | 0.016627078 |
| CABP4 | 7 | 0.016627078 |
| MEIS3 | 7 | 0.016627078 |
| FOXB2 | 7 | 0.016627078 |
| VAC14 | 7 | 0.016627078 |
| IDH1 | 7 | 0.016627078 |
| PRKCQ | 7 | 0.016627078 |
| ZFYVE27 | 7 | 0.016627078 |
| MOXD1 | 7 | 0.016627078 |
| LONP2 | 7 | 0.016627078 |
| CPO | 7 | 0.016627078 |
| E2F5 | 7 | 0.016627078 |
| KYAT3 | 7 | 0.016627078 |
| IP6K1 | 7 | 0.016627078 |
| MNX1 | 7 | 0.016627078 |
| FBXL14 | 7 | 0.016627078 |
| PLCXD1 | 7 | 0.016627078 |
| AGPAT4 | 7 | 0.016627078 |
| KIAA0895 | 7 | 0.016627078 |
| ZNF92 | 7 | 0.016627078 |
| TBC1D19 | 7 | 0.016627078 |
| NDUFA9 | 7 | 0.016627078 |
| GNL2 | 7 | 0.016627078 |
| MPP6 | 7 | 0.016627078 |
| IGHV1OR21-1 | 7 | 0.016627078 |
| IPPK | 7 | 0.016627078 |
| MBOAT2 | 7 | 0.016627078 |
| RPUSD1 | 7 | 0.016627078 |
| HEATR9 | 7 | 0.016627078 |
| CACNG1 | 7 | 0.016627078 |
| TRIL | 7 | 0.016627078 |
| PUF60 | 7 | 0.016627078 |
| ZNF333 | 7 | 0.016627078 |
| MFSD11 | 7 | 0.016627078 |
| COQ8B | 7 | 0.016627078 |
| PARD6G | 7 | 0.016627078 |
| ERMARD | 7 | 0.016627078 |
| TNFSF13B | 7 | 0.016627078 |
| ACVRL1 | 7 | 0.016627078 |
| ZNF839 | 7 | 0.016627078 |
| CFHR5 | 7 | 0.016627078 |
| CCDC74B | 7 | 0.016627078 |
| ZNF785 | 7 | 0.016627078 |
| ATP1B2 | 7 | 0.016627078 |
| HCFC1R1 | 7 | 0.016627078 |
| CDS2 | 7 | 0.016627078 |
| ADCK5 | 7 | 0.016627078 |
| SLC16A9 | 7 | 0.016627078 |
| MAT1A | 7 | 0.016627078 |
| IFIT1 | 7 | 0.016627078 |
| TTLL1 | 7 | 0.016627078 |
| DENND11 | 7 | 0.016627078 |
| SAMD14 | 7 | 0.016627078 |
| BCAT1 | 7 | 0.016627078 |
| SGCG | 7 | 0.016627078 |
| MYSM1 | 7 | 0.016627078 |
| ZNF622 | 7 | 0.016627078 |
| WARS2 | 7 | 0.016627078 |
| DCT | 7 | 0.016627078 |
| HSD17B2 | 7 | 0.016627078 |
| IRF7 | 7 | 0.016627078 |
| DRC3 | 7 | 0.016627078 |
| ECSIT | 7 | 0.016627078 |
| GPN1 | 7 | 0.016627078 |
| PNMT | 7 | 0.016627078 |
| GPBP1L1 | 7 | 0.016627078 |
| KRBA1 | 7 | 0.016627078 |
| MAX | 7 | 0.016627078 |
| KBTBD4 | 7 | 0.016627078 |
| TMEM117 | 7 | 0.016627078 |
| MFSD8 | 7 | 0.016627078 |
| ZNF430 | 7 | 0.016627078 |
| RMDN1 | 7 | 0.016627078 |
| RHOA | 7 | 0.016627078 |
| TRABD | 7 | 0.016627078 |
| SLC44A3 | 7 | 0.016627078 |
| FSD1 | 7 | 0.016627078 |
| UVRAG | 7 | 0.016627078 |
| ANAPC7 | 7 | 0.016627078 |
| CNST | 7 | 0.016627078 |
| MVD | 7 | 0.016627078 |
| PRDM7 | 7 | 0.016627078 |
| CERT1 | 7 | 0.016627078 |
| SSX2IP | 7 | 0.016627078 |
| CTSH | 7 | 0.016627078 |
| ADCK2 | 7 | 0.016627078 |
| ZACN | 7 | 0.016627078 |
| RFC4 | 7 | 0.016627078 |
| HLF | 7 | 0.016627078 |
| CYP2C8 | 7 | 0.016627078 |
| SAMD4B | 7 | 0.016627078 |
| BTBD2 | 7 | 0.016627078 |
| SCRN1 | 7 | 0.016627078 |
| BTN3A3 | 7 | 0.016627078 |
| CREB5 | 7 | 0.016627078 |
| EN1 | 7 | 0.016627078 |
| TROAP | 7 | 0.016627078 |
| ETV4 | 7 | 0.016627078 |
| NFKBIB | 7 | 0.016627078 |
| CYSLTR1 | 7 | 0.016627078 |
| TRIM58 | 7 | 0.016627078 |
| TAGAP | 7 | 0.016627078 |
| BRS3 | 7 | 0.016627078 |
| PIP5K1A | 7 | 0.016627078 |
| KCNN1 | 7 | 0.016627078 |
| BMP4 | 7 | 0.016627078 |
| MAPRE2 | 7 | 0.016627078 |
| MSH5 | 7 | 0.016627078 |
| OR52K2 | 7 | 0.016627078 |
| SLC15A3 | 7 | 0.016627078 |
| SNPH | 7 | 0.016627078 |
| TAF1D | 7 | 0.016627078 |
| OR51M1 | 7 | 0.016627078 |
| TCEANC | 7 | 0.016627078 |
| PRODH2 | 7 | 0.016627078 |
| MCMDC2 | 7 | 0.016627078 |
| PRAMEF12 | 7 | 0.016627078 |
| EPB42 | 7 | 0.016627078 |
| PAK6 | 7 | 0.016627078 |
| TUBG2 | 7 | 0.016627078 |
| CHRNA6 | 7 | 0.016627078 |
| UBQLN1 | 7 | 0.016627078 |
| RUNDC3B | 7 | 0.016627078 |
| ZNF586 | 7 | 0.016627078 |
| OR51A7 | 7 | 0.016627078 |
| TUBE1 | 7 | 0.016627078 |
| MOCS1 | 7 | 0.016627078 |
| FCGRT | 7 | 0.016627078 |
| NOSIP | 7 | 0.016627078 |
| UPRT | 7 | 0.016627078 |
| RINL | 7 | 0.016627078 |
| MNS1 | 7 | 0.016627078 |
| ACSM2A | 7 | 0.016627078 |
| ATG9A | 7 | 0.016627078 |
| CFAP53 | 7 | 0.016627078 |
| ASPRV1 | 7 | 0.016627078 |
| PLA2G15 | 7 | 0.016627078 |
| TNFAIP2 | 7 | 0.016627078 |
| HNRNPD | 7 | 0.016627078 |
| LONRF3 | 7 | 0.016627078 |
| SUN5 | 7 | 0.016627078 |
| GJA9 | 7 | 0.016627078 |
| SEPTIN3 | 7 | 0.016627078 |
| PTBP3 | 7 | 0.016627078 |
| FBXO22 | 7 | 0.016627078 |
| TUBA3D | 7 | 0.016627078 |
| RNF44 | 7 | 0.016627078 |
| RNF32 | 7 | 0.016627078 |
| HS6ST1 | 7 | 0.016627078 |
| RSPH10B2 | 7 | 0.016627078 |
| UBE2Q1 | 7 | 0.016627078 |
| OR14C36 | 7 | 0.016627078 |
| TBATA | 7 | 0.016627078 |
| IFNAR1 | 7 | 0.016627078 |
| ABCG5 | 7 | 0.016627078 |
| TRIM25 | 7 | 0.016627078 |
| SLC38A11 | 7 | 0.016627078 |
| MAP2K3 | 7 | 0.016627078 |
| TMEM41A | 7 | 0.016627078 |
| TINAGL1 | 7 | 0.016627078 |
| CCDC7 | 7 | 0.016627078 |
| CYP3A4 | 7 | 0.016627078 |
| GFPT1 | 7 | 0.016627078 |
| SLC28A3 | 7 | 0.016627078 |
| CREB3 | 7 | 0.016627078 |
| ZNF772 | 7 | 0.016627078 |
| B4GALT5 | 7 | 0.016627078 |
| GRXCR1 | 7 | 0.016627078 |
| SGPP1 | 7 | 0.016627078 |
| CR1L | 7 | 0.016627078 |
| SMOC2 | 7 | 0.016627078 |
| GIPC1 | 7 | 0.016627078 |
| OR2L8 | 7 | 0.016627078 |
| PXN | 7 | 0.016627078 |
| GLB1 | 7 | 0.016627078 |
| PIANP | 7 | 0.016627078 |
| CLCNKA | 7 | 0.016627078 |
| ITPKC | 7 | 0.016627078 |
| CABP2 | 7 | 0.016627078 |
| EXD1 | 7 | 0.016627078 |
| GJA3 | 7 | 0.016627078 |
| SLC5A12 | 7 | 0.016627078 |
| CCDC42 | 7 | 0.016627078 |
| AVPR2 | 7 | 0.016627078 |
| DLK1 | 7 | 0.016627078 |
| CTSW | 7 | 0.016627078 |
| TELO2 | 7 | 0.016627078 |
| ASIC1 | 7 | 0.016627078 |
| ZNF254 | 7 | 0.016627078 |
| SOD2 | 7 | 0.016627078 |
| ZNF669 | 7 | 0.016627078 |
| OGA | 7 | 0.016627078 |
| SLC37A1 | 7 | 0.016627078 |
| GAL3ST2 | 7 | 0.016627078 |
| HAL | 7 | 0.016627078 |
| C22orf23 | 7 | 0.016627078 |
| OXNAD1 | 7 | 0.016627078 |
| NKRF | 7 | 0.016627078 |
| LRRC43 | 7 | 0.016627078 |
| FAS | 7 | 0.016627078 |
| SHE | 7 | 0.016627078 |
| ZNF100 | 7 | 0.016627078 |
| RNF39 | 7 | 0.016627078 |
| OR8K3 | 7 | 0.016627078 |
| PLEKHG5 | 7 | 0.016627078 |
| AK7 | 7 | 0.016627078 |
| MAN1C1 | 7 | 0.016627078 |
| GPR78 | 7 | 0.016627078 |
| RAG2 | 7 | 0.016627078 |
| OR13F1 | 7 | 0.016627078 |
| LRRC40 | 7 | 0.016627078 |
| SLC22A15 | 7 | 0.016627078 |
| DOK6 | 7 | 0.016627078 |
| C1QTNF3 | 7 | 0.016627078 |
| SPATA1 | 7 | 0.016627078 |
| PARN | 7 | 0.016627078 |
| ESS2 | 7 | 0.016627078 |
| APCDD1 | 7 | 0.016627078 |
| UCP1 | 7 | 0.016627078 |
| TCF25 | 7 | 0.016627078 |
| SH3RF1 | 7 | 0.016627078 |
| TARS2 | 7 | 0.016627078 |
| MMP27 | 7 | 0.016627078 |
| OGFRL1 | 7 | 0.016627078 |
| PLAAT4 | 7 | 0.016627078 |
| ZSCAN5A | 7 | 0.016627078 |
| ZNF391 | 7 | 0.016627078 |
| ST3GAL6 | 7 | 0.016627078 |
| GBA3 | 7 | 0.016627078 |
| MYCT1 | 7 | 0.016627078 |
| GAMT | 7 | 0.016627078 |
| ACSL4 | 7 | 0.016627078 |
| PRKAG1 | 7 | 0.016627078 |
| ZDHHC15 | 7 | 0.016627078 |
| FBXL5 | 7 | 0.016627078 |
| ADAT1 | 7 | 0.016627078 |
| CHRM1 | 7 | 0.016627078 |
| ZBTB6 | 7 | 0.016627078 |
| PEX2 | 7 | 0.016627078 |
| GTF2E1 | 7 | 0.016627078 |
| DMRT3 | 7 | 0.016627078 |
| CDH26 | 7 | 0.016627078 |
| PSIP1 | 7 | 0.016627078 |
| ACTL6A | 7 | 0.016627078 |
| TMEM260 | 7 | 0.016627078 |
| GNA12 | 7 | 0.016627078 |
| ITGA2 | 7 | 0.016627078 |
| DAPP1 | 7 | 0.016627078 |
| CRPPA | 7 | 0.016627078 |
| USP21 | 7 | 0.016627078 |
| WFIKKN2 | 7 | 0.016627078 |
| TRMT1 | 7 | 0.016627078 |
| ASH2L | 7 | 0.016627078 |
| KIF2A | 7 | 0.016627078 |
| ZNF789 | 7 | 0.016627078 |
| TMEM145 | 7 | 0.016627078 |
| MEX3C | 7 | 0.016627078 |
| AMIGO1 | 7 | 0.016627078 |
| STAT5A | 7 | 0.016627078 |
| WDR41 | 7 | 0.016627078 |
| MAPK8 | 7 | 0.016627078 |
| GALNT6 | 7 | 0.016627078 |
| TUBA4A | 7 | 0.016627078 |
| OSBPL9 | 7 | 0.016627078 |
| DGAT1 | 7 | 0.016627078 |
| NDUFAF5 | 7 | 0.016627078 |
| RTCA | 7 | 0.016627078 |
| TRIM39 | 7 | 0.016627078 |
| TFG | 7 | 0.016627078 |
| SLC2A11 | 7 | 0.016627078 |
| THEG | 7 | 0.016627078 |
| TPBG | 7 | 0.016627078 |
| OR6B3 | 7 | 0.016627078 |
| IRF6 | 7 | 0.016627078 |
| SNTG2 | 7 | 0.016627078 |
| MARS2 | 7 | 0.016627078 |
| TBC1D22B | 7 | 0.016627078 |
| CCDC112 | 7 | 0.016627078 |
| C12orf50 | 7 | 0.016627078 |
| SLC19A2 | 7 | 0.016627078 |
| KCTD16 | 7 | 0.016627078 |
| SEMG1 | 7 | 0.016627078 |
| FUT8 | 7 | 0.016627078 |
| MEGF9 | 7 | 0.016627078 |
| C8A | 7 | 0.016627078 |
| R3HCC1L | 7 | 0.016627078 |
| SLC29A4 | 7 | 0.016627078 |
| EMC7 | 7 | 0.016627078 |
| LDB1 | 7 | 0.016627078 |
| MAP3K7 | 7 | 0.016627078 |
| OR51T1 | 7 | 0.016627078 |
| SHF | 7 | 0.016627078 |
| OR10H2 | 7 | 0.016627078 |
| POLD3 | 7 | 0.016627078 |
| SLC5A5 | 7 | 0.016627078 |
| HAS2 | 7 | 0.016627078 |
| EIF4A2 | 7 | 0.016627078 |
| CYP24A1 | 7 | 0.016627078 |
| OR2G3 | 7 | 0.016627078 |
| P3H2 | 7 | 0.016627078 |
| SPANXN3 | 7 | 0.016627078 |
| BRDT | 7 | 0.016627078 |
| HSP90AA1 | 7 | 0.016627078 |
| ACOX1 | 7 | 0.016627078 |
| P2RX1 | 7 | 0.016627078 |
| SLU7 | 7 | 0.016627078 |
| OPN1SW | 7 | 0.016627078 |
| TAAR6 | 7 | 0.016627078 |
| RABL6 | 7 | 0.016627078 |
| PSG9 | 7 | 0.016627078 |
| CDC40 | 7 | 0.016627078 |
| OR5C1 | 7 | 0.016627078 |
| HOXD3 | 7 | 0.016627078 |
| LHPP | 7 | 0.016627078 |
| CDR2L | 7 | 0.016627078 |
| OR2T27 | 7 | 0.016627078 |
| HLA-DPA1 | 7 | 0.016627078 |
| SHFL | 7 | 0.016627078 |
| TXNDC2 | 7 | 0.016627078 |
| OR10R2 | 7 | 0.016627078 |
| PLS1 | 7 | 0.016627078 |
| SMAD9 | 7 | 0.016627078 |
| NKX2-5 | 7 | 0.016627078 |
| HGFAC | 7 | 0.016627078 |
| ZNF281 | 7 | 0.016627078 |
| EFCAB12 | 7 | 0.016627078 |
| SLC43A1 | 7 | 0.016627078 |
| KATNAL1 | 7 | 0.016627078 |
| DCDC2 | 7 | 0.016627078 |
| ARL14EP | 7 | 0.016627078 |
| DHX33 | 7 | 0.016627078 |
| CDC45 | 7 | 0.016627078 |
| ORC5 | 7 | 0.016627078 |
| ETS2 | 7 | 0.016627078 |
| KIR2DL1 | 7 | 0.016627078 |
| OR52I2 | 7 | 0.016627078 |
| PROC | 7 | 0.016627078 |
| CS | 7 | 0.016627078 |
| ZNF415 | 7 | 0.016627078 |
| PNLIPRP3 | 7 | 0.016627078 |
| TDRD15 | 7 | 0.016627078 |
| ASMT | 7 | 0.016627078 |
| VASH1 | 7 | 0.016627078 |
| BMERB1 | 7 | 0.016627078 |
| GREB1L | 7 | 0.016627078 |
| OSBPL11 | 7 | 0.016627078 |
| ZNF718 | 7 | 0.016627078 |
| LRRC42 | 7 | 0.016627078 |
| SLC22A4 | 7 | 0.016627078 |
| OR52N4 | 7 | 0.016627078 |
| HIPK4 | 7 | 0.016627078 |
| LGR6 | 7 | 0.016627078 |
| DAW1 | 7 | 0.016627078 |
| A1BG | 7 | 0.016627078 |
| H1-3 | 7 | 0.016627078 |
| TRIB2 | 7 | 0.016627078 |
| PPP2R5B | 7 | 0.016627078 |
| SLC26A2 | 7 | 0.016627078 |
| NKX6-2 | 7 | 0.016627078 |
| CYP4A11 | 7 | 0.016627078 |
| OR7G2 | 7 | 0.016627078 |
| ERAP2 | 7 | 0.016627078 |
| USP9Y | 7 | 0.016627078 |
| ZNF782 | 7 | 0.016627078 |
| ARMC5 | 7 | 0.016627078 |
| KRT32 | 7 | 0.016627078 |
| TLX2 | 7 | 0.016627078 |
| TUBAL3 | 7 | 0.016627078 |
| TMPRSS3 | 7 | 0.016627078 |
| ATG5 | 7 | 0.016627078 |
| DLGAP5 | 7 | 0.016627078 |
| TRPV4 | 7 | 0.016627078 |
| PIP4P2 | 7 | 0.016627078 |
| ATPSCKMT | 7 | 0.016627078 |
| PPM1A | 7 | 0.016627078 |
| KIFBP | 7 | 0.016627078 |
| CCDC60 | 7 | 0.016627078 |
| SLC10A4 | 7 | 0.016627078 |
| UBE3D | 7 | 0.016627078 |
| SH3BP5 | 7 | 0.016627078 |
| SIX1 | 7 | 0.016627078 |
| NAIF1 | 7 | 0.016627078 |
| EIF3D | 7 | 0.016627078 |
| ACER1 | 7 | 0.016627078 |
| DCAKD | 7 | 0.016627078 |
| TDRD1 | 7 | 0.016627078 |
| GDPD4 | 7 | 0.016627078 |
| FOXS1 | 7 | 0.016627078 |
| NLRP6 | 7 | 0.016627078 |
| DCAF7 | 7 | 0.016627078 |
| RO60 | 7 | 0.016627078 |
| CENPN | 7 | 0.016627078 |
| LUZP4 | 7 | 0.016627078 |
| GIMAP7 | 7 | 0.016627078 |
| FAM149A | 7 | 0.016627078 |
| ELF5 | 7 | 0.016627078 |
| CDK17 | 7 | 0.016627078 |
| CDC16 | 7 | 0.016627078 |
| DIPK2B | 7 | 0.016627078 |
| TIMD4 | 7 | 0.016627078 |
| CFAP57 | 7 | 0.016627078 |
| ANKEF1 | 7 | 0.016627078 |
| ZNF439 | 7 | 0.016627078 |
| PES1 | 7 | 0.016627078 |
| SP2 | 7 | 0.016627078 |
| PEPD | 7 | 0.016627078 |
| GTF2F1 | 7 | 0.016627078 |
| IRF5 | 7 | 0.016627078 |
| FZD1 | 7 | 0.016627078 |
| COPG1 | 7 | 0.016627078 |
| MYOZ2 | 7 | 0.016627078 |
| LRRC74A | 7 | 0.016627078 |
| MFSD6L | 7 | 0.016627078 |
| OR4C11 | 7 | 0.016627078 |
| KCTD9 | 7 | 0.016627078 |
| DTNB | 7 | 0.016627078 |
| LRRC39 | 7 | 0.016627078 |
| CAPN2 | 7 | 0.016627078 |
| GDPD5 | 7 | 0.016627078 |
| ZFYVE1 | 7 | 0.016627078 |
| FAM20B | 7 | 0.016627078 |
| RNASE10 | 7 | 0.016627078 |
| DUSP4 | 7 | 0.016627078 |
| SYT1 | 7 | 0.016627078 |
| TFPI | 7 | 0.016627078 |
| ZC3H7A | 7 | 0.016627078 |
| GRB7 | 7 | 0.016627078 |
| ISM2 | 7 | 0.016627078 |
| RAD54L | 7 | 0.016627078 |
| CYP1A1 | 7 | 0.016627078 |
| NELFA | 7 | 0.016627078 |
| ZNF449 | 7 | 0.016627078 |
| SLC7A9 | 7 | 0.016627078 |
| PRG3 | 7 | 0.016627078 |
| FGG | 7 | 0.016627078 |
| HS3ST2 | 7 | 0.016627078 |
| TC2N | 7 | 0.016627078 |
| MMP21 | 7 | 0.016627078 |
| REXO1 | 7 | 0.016627078 |
| KRT72 | 7 | 0.016627078 |
| NIFK | 7 | 0.016627078 |
| PPWD1 | 7 | 0.016627078 |
| TRIM26 | 7 | 0.016627078 |
| GMIP | 7 | 0.016627078 |
| NUTM2F | 7 | 0.016627078 |
| FSTL1 | 7 | 0.016627078 |
| NAB2 | 7 | 0.016627078 |
| GSDME | 7 | 0.016627078 |
| ELP2 | 7 | 0.016627078 |
| RTL8A | 7 | 0.016627078 |
| CLPB | 7 | 0.016627078 |
| CBLN3 | 7 | 0.016627078 |
| SMPD3 | 7 | 0.016627078 |
| ELANE | 7 | 0.016627078 |
| PRAMEF8 | 7 | 0.016627078 |
| MSH4 | 7 | 0.016627078 |
| DEPDC7 | 7 | 0.016627078 |
| NECAB1 | 7 | 0.016627078 |
| OPRK1 | 7 | 0.016627078 |
| SLC25A32 | 7 | 0.016627078 |
| CLP1 | 7 | 0.016627078 |
| ENAH | 7 | 0.016627078 |
| COG8 | 7 | 0.016627078 |
| LMOD3 | 7 | 0.016627078 |
| LMNB1 | 7 | 0.016627078 |
| NAXD | 7 | 0.016627078 |
| CDC25C | 7 | 0.016627078 |
| CALHM5 | 7 | 0.016627078 |
| ANLN | 7 | 0.016627078 |
| ACSL6 | 7 | 0.016627078 |
| HDAC11 | 7 | 0.016627078 |
| ZNF766 | 7 | 0.016627078 |
| CCDC172 | 7 | 0.016627078 |
| ZNF486 | 7 | 0.016627078 |
| HPS5 | 7 | 0.016627078 |
| ATP1B4 | 7 | 0.016627078 |
| NET1 | 7 | 0.016627078 |
| SLC13A4 | 7 | 0.016627078 |
| ABHD16A | 7 | 0.016627078 |
| CCT6B | 7 | 0.016627078 |
| DNAJB5 | 7 | 0.016627078 |
| TCF19 | 7 | 0.016627078 |
| FASTKD5 | 7 | 0.016627078 |
| CCN6 | 7 | 0.016627078 |
| RGL4 | 7 | 0.016627078 |
| UNC93A | 7 | 0.016627078 |
| ERP44 | 7 | 0.016627078 |
| HSF2 | 7 | 0.016627078 |
| KLC4 | 7 | 0.016627078 |
| UPK1B | 7 | 0.016627078 |
| BHLHE22 | 7 | 0.016627078 |
| SREK1IP1 | 7 | 0.016627078 |
| PKD1L2 | 7 | 0.016627078 |
| CXCR4 | 7 | 0.016627078 |
| MICALL1 | 7 | 0.016627078 |
| ZNF460 | 7 | 0.016627078 |
| CLDN8 | 7 | 0.016627078 |
| STAC2 | 7 | 0.016627078 |
| ZNF223 | 7 | 0.016627078 |
| BRMS1 | 7 | 0.016627078 |
| MFSD9 | 7 | 0.016627078 |
| ZNF18 | 7 | 0.016627078 |
| PROKR2 | 7 | 0.016627078 |
| SPHK2 | 7 | 0.016627078 |
| CHST8 | 7 | 0.016627078 |
| SLC35C2 | 7 | 0.016627078 |
| MCRS1 | 7 | 0.016627078 |
| FGFRL1 | 7 | 0.016627078 |
| CA3 | 7 | 0.016627078 |
| REM2 | 7 | 0.016627078 |
| COQ2 | 7 | 0.016627078 |
| ZDHHC11 | 7 | 0.016627078 |
| KCNJ1 | 7 | 0.016627078 |
| EPHX4 | 7 | 0.016627078 |
| FGF5 | 7 | 0.016627078 |
| FOXE1 | 7 | 0.016627078 |
| ELAC2 | 7 | 0.016627078 |
| PCSK6 | 7 | 0.016627078 |
| ULBP2 | 7 | 0.016627078 |
| SELL | 7 | 0.016627078 |
| DIO3 | 7 | 0.016627078 |
| IGKV2-24 | 7 | 0.016627078 |
| TRMU | 7 | 0.016627078 |
| SLCO2B1 | 7 | 0.016627078 |
| SLC66A1 | 7 | 0.016627078 |
| OR1L3 | 7 | 0.016627078 |
| FERMT1 | 7 | 0.016627078 |
| KLHL41 | 7 | 0.016627078 |
| ALDH6A1 | 7 | 0.016627078 |
| PPP1R16A | 7 | 0.016627078 |
| FOXD4L1 | 7 | 0.016627078 |
| KCNE5 | 7 | 0.016627078 |
| ODF3L2 | 7 | 0.016627078 |
| MDM4 | 7 | 0.016627078 |
| DVL1 | 7 | 0.016627078 |
| SERPINC1 | 7 | 0.016627078 |
| SLC9B1 | 7 | 0.016627078 |
| OR5D16 | 7 | 0.016627078 |
| GLRA1 | 7 | 0.016627078 |
| MPPED1 | 7 | 0.016627078 |
| IHO1 | 7 | 0.016627078 |
| SYTL4 | 7 | 0.016627078 |
| GGA1 | 7 | 0.016627078 |
| COPS2 | 7 | 0.016627078 |
| ADRB2 | 7 | 0.016627078 |
| MEAK7 | 7 | 0.016627078 |
| EDN3 | 7 | 0.016627078 |
| MGAT4B | 7 | 0.016627078 |
| UBA1 | 7 | 0.016627078 |
| GPR15 | 7 | 0.016627078 |
| ZNF502 | 7 | 0.016627078 |
| CCN4 | 7 | 0.016627078 |
| RGP1 | 7 | 0.016627078 |
| SART3 | 7 | 0.016627078 |
| NIPAL3 | 7 | 0.016627078 |
| AP1M2 | 7 | 0.016627078 |
| PGBD1 | 7 | 0.016627078 |
| SLC38A3 | 7 | 0.016627078 |
| OLFML3 | 7 | 0.016627078 |
| IRX3 | 7 | 0.016627078 |
| CMIP | 7 | 0.016627078 |
| ARL11 | 7 | 0.016627078 |
| KLHL7 | 7 | 0.016627078 |
| RAI2 | 7 | 0.016627078 |
| ZSCAN18 | 7 | 0.016627078 |
| ERAP1 | 7 | 0.016627078 |
| PLEKHO2 | 7 | 0.016627078 |
| IMPDH2 | 7 | 0.016627078 |
| TEAD1 | 7 | 0.016627078 |
| FRRS1L | 7 | 0.016627078 |
| NOXRED1 | 7 | 0.016627078 |
| KLHL31 | 7 | 0.016627078 |
| WSCD1 | 7 | 0.016627078 |
| CYP17A1 | 7 | 0.016627078 |
| NT5DC3 | 7 | 0.016627078 |
| PWWP2B | 7 | 0.016627078 |
| MARVELD3 | 7 | 0.016627078 |
| GALT | 7 | 0.016627078 |
| GRSF1 | 7 | 0.016627078 |
| KDM3A | 7 | 0.016627078 |
| SELENOO | 7 | 0.016627078 |
| SLAMF1 | 7 | 0.016627078 |
| TAF7L | 7 | 0.016627078 |
| OR5AP2 | 7 | 0.016627078 |
| HOXD10 | 7 | 0.016627078 |
| B4GALT3 | 7 | 0.016627078 |
| CAMK2D | 7 | 0.016627078 |
| MLC1 | 7 | 0.016627078 |
| ZNF24 | 7 | 0.016627078 |
| SLC29A3 | 7 | 0.016627078 |
| NPRL3 | 7 | 0.016627078 |
| TFAP2A | 7 | 0.016627078 |
| WRAP73 | 7 | 0.016627078 |
| KARS1 | 7 | 0.016627078 |
| PHTF2 | 7 | 0.016627078 |
| IK | 7 | 0.016627078 |
| TEKT4 | 7 | 0.016627078 |
| MLLT3 | 7 | 0.016627078 |
| ACAD9 | 7 | 0.016627078 |
| TDRP | 7 | 0.016627078 |
| SUSD2 | 7 | 0.016627078 |
| SLC16A10 | 7 | 0.016627078 |
| CCM2L | 7 | 0.016627078 |
| CLN3 | 7 | 0.016627078 |
| LCTL | 7 | 0.016627078 |
| NEUROD2 | 7 | 0.016627078 |
| SLC22A3 | 7 | 0.016627078 |
| NUAK2 | 7 | 0.016627078 |
| HNRNPA3 | 7 | 0.016627078 |
| PVRIG | 7 | 0.016627078 |
| MTCH2 | 7 | 0.016627078 |
| PLVAP | 7 | 0.016627078 |
| KRR1 | 7 | 0.016627078 |
| MAGEA11 | 7 | 0.016627078 |
| MS4A1 | 7 | 0.016627078 |
| IKBIP | 7 | 0.016627078 |
| ZBTB18 | 7 | 0.016627078 |
| CRACR2B | 7 | 0.016627078 |
| MRE11 | 7 | 0.016627078 |
| ADAM28 | 7 | 0.016627078 |
| EEFSEC | 7 | 0.016627078 |
| PLD5 | 7 | 0.016627078 |
| MAPK7 | 7 | 0.016627078 |
| SMAD1 | 7 | 0.016627078 |
| PBX2 | 7 | 0.016627078 |
| CCDC59 | 7 | 0.016627078 |
| COLQ | 7 | 0.016627078 |
| SGSM3 | 7 | 0.016627078 |
| SYN2 | 7 | 0.016627078 |
| CDK19 | 7 | 0.016627078 |
| ENOX1 | 7 | 0.016627078 |
| EMB | 7 | 0.016627078 |
| SMARCD3 | 7 | 0.016627078 |
| REN | 7 | 0.016627078 |
| SLC37A4 | 7 | 0.016627078 |
| RPN1 | 7 | 0.016627078 |
| SRSF7 | 7 | 0.016627078 |
| CORO6 | 7 | 0.016627078 |
| GNL1 | 7 | 0.016627078 |
| NTSR2 | 7 | 0.016627078 |
| SYBU | 7 | 0.016627078 |
| IRGQ | 7 | 0.016627078 |
| CH25H | 7 | 0.016627078 |
| NOP14 | 7 | 0.016627078 |
| GRAMD1A | 7 | 0.016627078 |
| AGBL2 | 7 | 0.016627078 |
| DIRAS2 | 7 | 0.016627078 |
| ESM1 | 7 | 0.016627078 |
| TRIML1 | 7 | 0.016627078 |
| PRMT9 | 7 | 0.016627078 |
| ZC3HAV1 | 7 | 0.016627078 |
| GPM6A | 7 | 0.016627078 |
| OTUD5 | 7 | 0.016627078 |
| ZBTB14 | 7 | 0.016627078 |
| DDHD2 | 7 | 0.016627078 |
| ZSCAN2 | 7 | 0.016627078 |
| TRAF3IP3 | 7 | 0.016627078 |
| NFIL3 | 7 | 0.016627078 |
| RPGR | 7 | 0.016627078 |
| ELK1 | 7 | 0.016627078 |
| LPIN2 | 7 | 0.016627078 |
| KCNMB2 | 7 | 0.016627078 |
| ABCG8 | 7 | 0.016627078 |
| ALAS1 | 7 | 0.016627078 |
| STAT3 | 7 | 0.016627078 |
| SPTLC3 | 7 | 0.016627078 |
| TRMT1L | 7 | 0.016627078 |
| BCAR1 | 7 | 0.016627078 |
| SPNS2 | 7 | 0.016627078 |
| CD200R1 | 7 | 0.016627078 |
| SYNGR4 | 7 | 0.016627078 |
| C1QL2 | 7 | 0.016627078 |
| SLC16A12 | 7 | 0.016627078 |
| FOXO1 | 7 | 0.016627078 |
| ARHGDIB | 7 | 0.016627078 |
| KCNK2 | 7 | 0.016627078 |
| GOLM1 | 7 | 0.016627078 |
| ZNF570 | 7 | 0.016627078 |
| LRRC8C | 7 | 0.016627078 |
| SLC26A11 | 7 | 0.016627078 |
| DCAF4 | 7 | 0.016627078 |
| PMEPA1 | 7 | 0.016627078 |
| SLC27A2 | 7 | 0.016627078 |
| GNL3 | 7 | 0.016627078 |
| CFAP298-TCP10L | 7 | 0.016627078 |
| SPATA31A3 | 7 | 0.016627078 |
| R3HDM4 | 7 | 0.016627078 |
| NUP54 | 7 | 0.016627078 |
| B3GALT2 | 7 | 0.016627078 |
| STRN4 | 7 | 0.016627078 |
| AGBL4 | 7 | 0.016627078 |
| GNE | 7 | 0.016627078 |
| CMTR2 | 7 | 0.016627078 |
| B3GLCT | 7 | 0.016627078 |
| OR51B4 | 7 | 0.016627078 |
| FLI1 | 7 | 0.016627078 |
| REPS2 | 7 | 0.016627078 |
| BGN | 7 | 0.016627078 |
| HIRIP3 | 7 | 0.016627078 |
| HAVCR2 | 7 | 0.016627078 |
| DENND1A | 7 | 0.016627078 |
| CTSF | 7 | 0.016627078 |
| RAVER2 | 7 | 0.016627078 |
| PSMD5 | 7 | 0.016627078 |
| IRGC | 7 | 0.016627078 |
| GRK4 | 7 | 0.016627078 |
| OR2T2 | 7 | 0.016627078 |
| HLA-G | 7 | 0.016627078 |
| ELOVL1 | 7 | 0.016627078 |
| EXOC3L2 | 7 | 0.016627078 |
| KRT17 | 7 | 0.016627078 |
| UBA7 | 7 | 0.016627078 |
| ACTL7A | 7 | 0.016627078 |
| EID3 | 7 | 0.016627078 |
| CEP68 | 7 | 0.016627078 |
| EPHX2 | 7 | 0.016627078 |
| SRPK2 | 7 | 0.016627078 |
| ZIM3 | 7 | 0.016627078 |
| MMP7 | 7 | 0.016627078 |
| CLEC4C | 7 | 0.016627078 |
| HTRA4 | 7 | 0.016627078 |
| GPR34 | 7 | 0.016627078 |
| CCKAR | 7 | 0.016627078 |
| FMNL2 | 7 | 0.016627078 |
| SEPTIN9 | 7 | 0.016627078 |
| FAM155A | 7 | 0.016627078 |
| HNRNPK | 7 | 0.016627078 |
| PCIF1 | 7 | 0.016627078 |
| APBB2 | 7 | 0.016627078 |
| ASPN | 7 | 0.016627078 |
| OR5M8 | 7 | 0.016627078 |
| TEX13B | 7 | 0.016627078 |
| PHF13 | 7 | 0.016627078 |
| SLC23A3 | 7 | 0.016627078 |
| PARP8 | 7 | 0.016627078 |
| KLC3 | 7 | 0.016627078 |
| ASAH1 | 7 | 0.016627078 |
| STRBP | 7 | 0.016627078 |
| GLYR1 | 7 | 0.016627078 |
| AC090517.4 | 7 | 0.016627078 |
| TSPAN7 | 7 | 0.016627078 |
| SC5D | 7 | 0.016627078 |
| LRRC28 | 7 | 0.016627078 |
| MMP26 | 7 | 0.016627078 |
| RIPOR2 | 7 | 0.016627078 |
| P2RY10 | 7 | 0.016627078 |
| NKX2-1 | 7 | 0.016627078 |
| CHRM5 | 7 | 0.016627078 |
| CYP4F8 | 7 | 0.016627078 |
| ACOX3 | 7 | 0.016627078 |
| RASGEF1B | 7 | 0.016627078 |
| CCDC27 | 7 | 0.016627078 |
| KLHL18 | 7 | 0.016627078 |
| GANC | 7 | 0.016627078 |
| HAGHL | 7 | 0.016627078 |
| SPOCK1 | 7 | 0.016627078 |
| ENTPD8 | 7 | 0.016627078 |
| STRN | 7 | 0.016627078 |
| SLC41A1 | 7 | 0.016627078 |
| CCZ1 | 7 | 0.016627078 |
| GALK2 | 7 | 0.016627078 |
| C1orf141 | 7 | 0.016627078 |
| TTYH3 | 7 | 0.016627078 |
| ANKRD33 | 7 | 0.016627078 |
| TMEFF2 | 7 | 0.016627078 |
| TCEA2 | 7 | 0.016627078 |
| PSMC6 | 7 | 0.016627078 |
| WIF1 | 7 | 0.016627078 |
| CLYBL | 7 | 0.016627078 |
| H6PD | 7 | 0.016627078 |
| LRIF1 | 7 | 0.016627078 |
| ELMO3 | 7 | 0.016627078 |
| NETO2 | 7 | 0.016627078 |
| OR2AT4 | 7 | 0.016627078 |
| DMBX1 | 7 | 0.016627078 |
| CARD14 | 7 | 0.016627078 |
| DOLK | 7 | 0.016627078 |
| CA9 | 7 | 0.016627078 |
| AGMO | 7 | 0.016627078 |
| INTS10 | 7 | 0.016627078 |
| APBA3 | 7 | 0.016627078 |
| ZNF71 | 7 | 0.016627078 |
| CSNK1G2 | 7 | 0.016627078 |
| OR10A6 | 7 | 0.016627078 |
| UMODL1 | 7 | 0.016627078 |
| ZMAT3 | 7 | 0.016627078 |
| ARMH4 | 7 | 0.016627078 |
| HVCN1 | 7 | 0.016627078 |
| CNKSR3 | 7 | 0.016627078 |
| NDNF | 7 | 0.016627078 |
| SLC16A14 | 7 | 0.016627078 |
| MVP | 7 | 0.016627078 |
| CA2 | 7 | 0.016627078 |
| AP002512.3 | 7 | 0.016627078 |
| CERK | 7 | 0.016627078 |
| OSM | 7 | 0.016627078 |
| CDNF | 7 | 0.016627078 |
| CCDC6 | 7 | 0.016627078 |
| CELF3 | 7 | 0.016627078 |
| CEP57 | 7 | 0.016627078 |
| GBP3 | 7 | 0.016627078 |
| SPRR3 | 7 | 0.016627078 |
| ERGIC3 | 7 | 0.016627078 |
| SNTB2 | 7 | 0.016627078 |
| SLC25A17 | 7 | 0.016627078 |
| CHRNG | 7 | 0.016627078 |
| APLNR | 7 | 0.016627078 |
| GRB10 | 7 | 0.016627078 |
| SUOX | 7 | 0.016627078 |
| PRDM11 | 7 | 0.016627078 |
| FRG2C | 7 | 0.016627078 |
| AOPEP | 7 | 0.016627078 |
| TOP1MT | 7 | 0.016627078 |
| RRP9 | 7 | 0.016627078 |
| GRAMD1C | 7 | 0.016627078 |
| IDO2 | 7 | 0.016627078 |
| RBMX2 | 7 | 0.016627078 |
| IGHV3OR16-8 | 7 | 0.016627078 |
| PGS1 | 7 | 0.016627078 |
| OR4S1 | 7 | 0.016627078 |
| ST6GALNAC2 | 7 | 0.016627078 |
| COQ8A | 7 | 0.016627078 |
| RWDD2B | 7 | 0.016627078 |
| HMX3 | 7 | 0.016627078 |
| ADRA2B | 7 | 0.016627078 |
| ENKD1 | 7 | 0.016627078 |
| BTBD10 | 7 | 0.016627078 |
| KLHL20 | 7 | 0.016627078 |
| GALNT7 | 7 | 0.016627078 |
| SLC5A10 | 7 | 0.016627078 |
| PRPF18 | 7 | 0.016627078 |
| MID2 | 7 | 0.016627078 |
| WDR37 | 7 | 0.016627078 |
| DNAH6 | 7 | 0.016627078 |
| KCNIP1 | 7 | 0.016627078 |
| SLC2A6 | 7 | 0.016627078 |
| TGIF2LX | 7 | 0.016627078 |
| AHSG | 7 | 0.016627078 |
| FAM181A | 7 | 0.016627078 |
| TDG | 7 | 0.016627078 |
| SLC36A1 | 7 | 0.016627078 |
| METAP2 | 7 | 0.016627078 |
| CCDC77 | 7 | 0.016627078 |
| CCDC157 | 7 | 0.016627078 |
| CGAS | 7 | 0.016627078 |
| OR10P1 | 7 | 0.016627078 |
| SYT9 | 7 | 0.016627078 |
| MDH1B | 7 | 0.016627078 |
| OXER1 | 7 | 0.016627078 |
| SERPINA5 | 7 | 0.016627078 |
| SLC26A4 | 7 | 0.016627078 |
| LRRTM2 | 7 | 0.016627078 |
| PPP2R5D | 7 | 0.016627078 |
| UNC45A | 7 | 0.016627078 |
| PTBP1 | 7 | 0.016627078 |
| FGF23 | 7 | 0.016627078 |
| VPS11 | 7 | 0.016627078 |
| C6orf15 | 7 | 0.016627078 |
| DDX6 | 7 | 0.016627078 |
| SRSF4 | 7 | 0.016627078 |
| IL11 | 7 | 0.016627078 |
| PAK2 | 7 | 0.016627078 |
| RASIP1 | 7 | 0.016627078 |
| FZD9 | 7 | 0.016627078 |
| HDC | 7 | 0.016627078 |
| LRCH3 | 7 | 0.016627078 |
| CHAD | 7 | 0.016627078 |
| DPY19L4 | 7 | 0.016627078 |
| MARCKSL1 | 7 | 0.016627078 |
| CBLN2 | 7 | 0.016627078 |
| POTED | 7 | 0.016627078 |
| SHC2 | 7 | 0.016627078 |
| TCHHL1 | 7 | 0.016627078 |
| KRT12 | 7 | 0.016627078 |
| SPI1 | 7 | 0.016627078 |
| VN1R5 | 7 | 0.016627078 |
| DBN1 | 7 | 0.016627078 |
| EXOC5 | 7 | 0.016627078 |
| AGXT2 | 7 | 0.016627078 |
| DHH | 7 | 0.016627078 |
| CKM | 7 | 0.016627078 |
| DKK4 | 7 | 0.016627078 |
| MKLN1 | 7 | 0.016627078 |
| NTF3 | 7 | 0.016627078 |
| FAM9B | 7 | 0.016627078 |
| RPS6KA5 | 7 | 0.016627078 |
| FAM160B1 | 7 | 0.016627078 |
| LMOD2 | 7 | 0.016627078 |
| CLCN5 | 7 | 0.016627078 |
| KCNA2 | 7 | 0.016627078 |
| PDE7B | 7 | 0.016627078 |
| DKK1 | 7 | 0.016627078 |
| C1RL | 7 | 0.016627078 |
| TCAIM | 7 | 0.016627078 |
| CCDC168 | 7 | 0.016627078 |
| C1orf94 | 7 | 0.016627078 |
| CRHR2 | 7 | 0.016627078 |
| TONSL | 7 | 0.016627078 |
| PCMTD1 | 7 | 0.016627078 |
| TBC1D12 | 7 | 0.016627078 |
| ZMAT4 | 7 | 0.016627078 |
| PPP4R3A | 7 | 0.016627078 |
| OR10K1 | 7 | 0.016627078 |
| ZNF519 | 7 | 0.016627078 |
| NLE1 | 7 | 0.016627078 |
| CCNK | 7 | 0.016627078 |
| CARD9 | 7 | 0.016627078 |
| YES1 | 7 | 0.016627078 |
| TMPRSS7 | 7 | 0.016627078 |
| NR2C1 | 7 | 0.016627078 |
| MAP4K2 | 7 | 0.016627078 |
| IGHMBP2 | 7 | 0.016627078 |
| RD3 | 7 | 0.016627078 |
| TSBP1 | 7 | 0.016627078 |
| TIGD3 | 7 | 0.016627078 |
| GTF2IRD2B | 7 | 0.016627078 |
| ZNF786 | 7 | 0.016627078 |
| MTRR | 7 | 0.016627078 |
| NFKB1 | 7 | 0.016627078 |
| CNN1 | 7 | 0.016627078 |
| TMEM79 | 7 | 0.016627078 |
| CD40LG | 7 | 0.016627078 |
| UGT1A4 | 7 | 0.016627078 |
| OR9K2 | 7 | 0.016627078 |
| ZDHHC17 | 7 | 0.016627078 |
| DDX39B | 7 | 0.016627078 |
| IP6K2 | 7 | 0.016627078 |
| GIN1 | 7 | 0.016627078 |
| LPAR3 | 7 | 0.016627078 |
| ACKR2 | 7 | 0.016627078 |
| DNAJB13 | 7 | 0.016627078 |
| ENTPD7 | 7 | 0.016627078 |
| ACTL6B | 7 | 0.016627078 |
| TGS1 | 7 | 0.016627078 |
| SEPHS1 | 6 | 0.014251781 |
| CIART | 6 | 0.014251781 |
| FOXL2 | 6 | 0.014251781 |
| SSUH2 | 6 | 0.014251781 |
| DEF6 | 6 | 0.014251781 |
| DRD2 | 6 | 0.014251781 |
| BFSP1 | 6 | 0.014251781 |
| CTPS1 | 6 | 0.014251781 |
| SOAT1 | 6 | 0.014251781 |
| NPAS1 | 6 | 0.014251781 |
| OR2J3 | 6 | 0.014251781 |
| APPBP2 | 6 | 0.014251781 |
| DHRS9 | 6 | 0.014251781 |
| FSIP1 | 6 | 0.014251781 |
| ASPHD2 | 6 | 0.014251781 |
| GPER1 | 6 | 0.014251781 |
| CALCR | 6 | 0.014251781 |
| NXN | 6 | 0.014251781 |
| OR2Z1 | 6 | 0.014251781 |
| CPNE7 | 6 | 0.014251781 |
| SGK1 | 6 | 0.014251781 |
| LY9 | 6 | 0.014251781 |
| TMEM64 | 6 | 0.014251781 |
| AC100868.1 | 6 | 0.014251781 |
| APEH | 6 | 0.014251781 |
| EMCN | 6 | 0.014251781 |
| PAQR8 | 6 | 0.014251781 |
| INPP1 | 6 | 0.014251781 |
| ATXN7L1 | 6 | 0.014251781 |
| ZNF446 | 6 | 0.014251781 |
| ARL8B | 6 | 0.014251781 |
| ACTN3 | 6 | 0.014251781 |
| PACS1 | 6 | 0.014251781 |
| UBLCP1 | 6 | 0.014251781 |
| CASP2 | 6 | 0.014251781 |
| SLC38A8 | 6 | 0.014251781 |
| TAS2R19 | 6 | 0.014251781 |
| RAP1GAP2 | 6 | 0.014251781 |
| TAF5L | 6 | 0.014251781 |
| HDAC2 | 6 | 0.014251781 |
| CDK5 | 6 | 0.014251781 |
| KRT25 | 6 | 0.014251781 |
| ZNF672 | 6 | 0.014251781 |
| GCAT | 6 | 0.014251781 |
| PSG4 | 6 | 0.014251781 |
| TRMT13 | 6 | 0.014251781 |
| RPTN | 6 | 0.014251781 |
| PRR14L | 6 | 0.014251781 |
| LBP | 6 | 0.014251781 |
| HPX | 6 | 0.014251781 |
| KCNK1 | 6 | 0.014251781 |
| ELAVL1 | 6 | 0.014251781 |
| LBH | 6 | 0.014251781 |
| RPL5 | 6 | 0.014251781 |
| PCOLCE | 6 | 0.014251781 |
| SIX2 | 6 | 0.014251781 |
| NBPF3 | 6 | 0.014251781 |
| PYM1 | 6 | 0.014251781 |
| TEAD4 | 6 | 0.014251781 |
| NFE2L2 | 6 | 0.014251781 |
| RXRB | 6 | 0.014251781 |
| FNTA | 6 | 0.014251781 |
| PALLD | 6 | 0.014251781 |
| STIM1 | 6 | 0.014251781 |
| DIPK1C | 6 | 0.014251781 |
| EPM2AIP1 | 6 | 0.014251781 |
| GSR | 6 | 0.014251781 |
| MMP8 | 6 | 0.014251781 |
| NOSTRIN | 6 | 0.014251781 |
| KIF25 | 6 | 0.014251781 |
| LLGL1 | 6 | 0.014251781 |
| RGS10 | 6 | 0.014251781 |
| C16orf72 | 6 | 0.014251781 |
| OR2B11 | 6 | 0.014251781 |
| KLK15 | 6 | 0.014251781 |
| NCEH1 | 6 | 0.014251781 |
| AFG3L2 | 6 | 0.014251781 |
| TSFM | 6 | 0.014251781 |
| ZNF367 | 6 | 0.014251781 |
| TPRX1 | 6 | 0.014251781 |
| MTMR10 | 6 | 0.014251781 |
| CHML | 6 | 0.014251781 |
| RNF151 | 6 | 0.014251781 |
| OR8B4 | 6 | 0.014251781 |
| APPL2 | 6 | 0.014251781 |
| PTAR1 | 6 | 0.014251781 |
| SLC22A8 | 6 | 0.014251781 |
| PAFAH1B1 | 6 | 0.014251781 |
| OTUD6B | 6 | 0.014251781 |
| LAP3 | 6 | 0.014251781 |
| C2orf42 | 6 | 0.014251781 |
| SERPINF2 | 6 | 0.014251781 |
| TSSK2 | 6 | 0.014251781 |
| DCAF13 | 6 | 0.014251781 |
| SETDB2 | 6 | 0.014251781 |
| CA8 | 6 | 0.014251781 |
| IGFBP3 | 6 | 0.014251781 |
| GNB3 | 6 | 0.014251781 |
| CPSF7 | 6 | 0.014251781 |
| CAST | 6 | 0.014251781 |
| PHYKPL | 6 | 0.014251781 |
| GPR85 | 6 | 0.014251781 |
| FAR1 | 6 | 0.014251781 |
| GMEB1 | 6 | 0.014251781 |
| TBX6 | 6 | 0.014251781 |
| MLF2 | 6 | 0.014251781 |
| RPS6KA3 | 6 | 0.014251781 |
| CBX3 | 6 | 0.014251781 |
| DNAJC6 | 6 | 0.014251781 |
| LINGO4 | 6 | 0.014251781 |
| CHODL | 6 | 0.014251781 |
| ENO3 | 6 | 0.014251781 |
| TCEA1 | 6 | 0.014251781 |
| LANCL1 | 6 | 0.014251781 |
| LY96 | 6 | 0.014251781 |
| PLCD3 | 6 | 0.014251781 |
| HNRNPF | 6 | 0.014251781 |
| EBPL | 6 | 0.014251781 |
| EFHD1 | 6 | 0.014251781 |
| FBXW2 | 6 | 0.014251781 |
| EEF1D | 6 | 0.014251781 |
| C6orf58 | 6 | 0.014251781 |
| CSGALNACT1 | 6 | 0.014251781 |
| IYD | 6 | 0.014251781 |
| UAP1L1 | 6 | 0.014251781 |
| TAS2R38 | 6 | 0.014251781 |
| KLHDC8A | 6 | 0.014251781 |
| MFSD13A | 6 | 0.014251781 |
| UBE2R2 | 6 | 0.014251781 |
| RGS4 | 6 | 0.014251781 |
| CMAS | 6 | 0.014251781 |
| NEFH | 6 | 0.014251781 |
| SLC19A1 | 6 | 0.014251781 |
| KRT18 | 6 | 0.014251781 |
| DRAM2 | 6 | 0.014251781 |
| TMEM184A | 6 | 0.014251781 |
| FBXL2 | 6 | 0.014251781 |
| DOK3 | 6 | 0.014251781 |
| C9orf43 | 6 | 0.014251781 |
| CHMP1A | 6 | 0.014251781 |
| ASTL | 6 | 0.014251781 |
| FAIM2 | 6 | 0.014251781 |
| PITPNC1 | 6 | 0.014251781 |
| FTSJ1 | 6 | 0.014251781 |
| AL441992.2 | 6 | 0.014251781 |
| ZNF121 | 6 | 0.014251781 |
| PPCS | 6 | 0.014251781 |
| CTIF | 6 | 0.014251781 |
| FBXO9 | 6 | 0.014251781 |
| SNX8 | 6 | 0.014251781 |
| ISM1 | 6 | 0.014251781 |
| ZNRF4 | 6 | 0.014251781 |
| TENT5B | 6 | 0.014251781 |
| GNS | 6 | 0.014251781 |
| PRR11 | 6 | 0.014251781 |
| EIF3L | 6 | 0.014251781 |
| ENPP4 | 6 | 0.014251781 |
| PIP4K2B | 6 | 0.014251781 |
| VGLL1 | 6 | 0.014251781 |
| C12orf66 | 6 | 0.014251781 |
| CD37 | 6 | 0.014251781 |
| GBP2 | 6 | 0.014251781 |
| HDHD5 | 6 | 0.014251781 |
| NUDT12 | 6 | 0.014251781 |
| HNRNPU | 6 | 0.014251781 |
| OTP | 6 | 0.014251781 |
| SVOPL | 6 | 0.014251781 |
| LGALS3BP | 6 | 0.014251781 |
| CLEC3A | 6 | 0.014251781 |
| FUT5 | 6 | 0.014251781 |
| CREBRF | 6 | 0.014251781 |
| SYT2 | 6 | 0.014251781 |
| DYNC2I2 | 6 | 0.014251781 |
| HSPA5 | 6 | 0.014251781 |
| LPAR6 | 6 | 0.014251781 |
| CD2 | 6 | 0.014251781 |
| PSTK | 6 | 0.014251781 |
| NAGK | 6 | 0.014251781 |
| MLNR | 6 | 0.014251781 |
| BMF | 6 | 0.014251781 |
| UBA2 | 6 | 0.014251781 |
| VPS51 | 6 | 0.014251781 |
| BEST4 | 6 | 0.014251781 |
| NARS2 | 6 | 0.014251781 |
| OXA1L | 6 | 0.014251781 |
| PSMC4 | 6 | 0.014251781 |
| UTP3 | 6 | 0.014251781 |
| C3AR1 | 6 | 0.014251781 |
| LHFPL6 | 6 | 0.014251781 |
| Z83844.2 | 6 | 0.014251781 |
| RAD9B | 6 | 0.014251781 |
| ZKSCAN4 | 6 | 0.014251781 |
| RPUSD2 | 6 | 0.014251781 |
| PON2 | 6 | 0.014251781 |
| PANX1 | 6 | 0.014251781 |
| WDR87 | 6 | 0.014251781 |
| ZMPSTE24 | 6 | 0.014251781 |
| RSAD2 | 6 | 0.014251781 |
| C17orf78 | 6 | 0.014251781 |
| CPEB3 | 6 | 0.014251781 |
| ZNF527 | 6 | 0.014251781 |
| AKR1B15 | 6 | 0.014251781 |
| ZNF670 | 6 | 0.014251781 |
| ATP5F1B | 6 | 0.014251781 |
| NXPE4 | 6 | 0.014251781 |
| RTF1 | 6 | 0.014251781 |
| DNAJB2 | 6 | 0.014251781 |
| GDAP1 | 6 | 0.014251781 |
| CSK | 6 | 0.014251781 |
| POLG2 | 6 | 0.014251781 |
| FANCG | 6 | 0.014251781 |
| ZC3H15 | 6 | 0.014251781 |
| UGT1A6 | 6 | 0.014251781 |
| ATXN7L3 | 6 | 0.014251781 |
| ANKDD1A | 6 | 0.014251781 |
| SLCO1C1 | 6 | 0.014251781 |
| IL5RA | 6 | 0.014251781 |
| CHGA | 6 | 0.014251781 |
| SERPINA7 | 6 | 0.014251781 |
| OR1L8 | 6 | 0.014251781 |
| TBCCD1 | 6 | 0.014251781 |
| UFL1 | 6 | 0.014251781 |
| RNF133 | 6 | 0.014251781 |
| ABCB6 | 6 | 0.014251781 |
| RAN | 6 | 0.014251781 |
| FOSB | 6 | 0.014251781 |
| SOX2 | 6 | 0.014251781 |
| ARMCX2 | 6 | 0.014251781 |
| PPOX | 6 | 0.014251781 |
| PLPPR1 | 6 | 0.014251781 |
| B4GALT6 | 6 | 0.014251781 |
| IL12RB1 | 6 | 0.014251781 |
| MBNL1 | 6 | 0.014251781 |
| APOL5 | 6 | 0.014251781 |
| ZNF621 | 6 | 0.014251781 |
| MINDY2 | 6 | 0.014251781 |
| G3BP2 | 6 | 0.014251781 |
| SNRNP70 | 6 | 0.014251781 |
| ATP6V0A1 | 6 | 0.014251781 |
| SH2B3 | 6 | 0.014251781 |
| TMEM59L | 6 | 0.014251781 |
| ALDH9A1 | 6 | 0.014251781 |
| NXPH2 | 6 | 0.014251781 |
| CCDC22 | 6 | 0.014251781 |
| ABCE1 | 6 | 0.014251781 |
| LCE2B | 6 | 0.014251781 |
| GJA4 | 6 | 0.014251781 |
| HS3ST5 | 6 | 0.014251781 |
| TARBP1 | 6 | 0.014251781 |
| SYPL2 | 6 | 0.014251781 |
| ADGRG5 | 6 | 0.014251781 |
| COPS7A | 6 | 0.014251781 |
| VSX2 | 6 | 0.014251781 |
| KCTD13 | 6 | 0.014251781 |
| PTCD2 | 6 | 0.014251781 |
| HSD17B14 | 6 | 0.014251781 |
| TSPAN2 | 6 | 0.014251781 |
| C20orf85 | 6 | 0.014251781 |
| HSF1 | 6 | 0.014251781 |
| MMP24 | 6 | 0.014251781 |
| LRTM1 | 6 | 0.014251781 |
| RRS1 | 6 | 0.014251781 |
| GABRD | 6 | 0.014251781 |
| SCPEP1 | 6 | 0.014251781 |
| ZSWIM3 | 6 | 0.014251781 |
| RAB36 | 6 | 0.014251781 |
| ZNF566 | 6 | 0.014251781 |
| KIAA1614 | 6 | 0.014251781 |
| C5orf34 | 6 | 0.014251781 |
| PLOD3 | 6 | 0.014251781 |
| SLC5A7 | 6 | 0.014251781 |
| PIH1D1 | 6 | 0.014251781 |
| KEAP1 | 6 | 0.014251781 |
| TTC31 | 6 | 0.014251781 |
| FZD8 | 6 | 0.014251781 |
| SGMS2 | 6 | 0.014251781 |
| ACKR1 | 6 | 0.014251781 |
| CUZD1 | 6 | 0.014251781 |
| SYN1 | 6 | 0.014251781 |
| SLC37A2 | 6 | 0.014251781 |
| MRPS22 | 6 | 0.014251781 |
| XKR8 | 6 | 0.014251781 |
| SLC30A3 | 6 | 0.014251781 |
| OR10Q1 | 6 | 0.014251781 |
| PLRG1 | 6 | 0.014251781 |
| ZNF875 | 6 | 0.014251781 |
| WNT3 | 6 | 0.014251781 |
| ITGB7 | 6 | 0.014251781 |
| HLA-DMB | 6 | 0.014251781 |
| OR8I2 | 6 | 0.014251781 |
| AICDA | 6 | 0.014251781 |
| OR13J1 | 6 | 0.014251781 |
| ALG6 | 6 | 0.014251781 |
| OTULIN | 6 | 0.014251781 |
| C8orf74 | 6 | 0.014251781 |
| CC2D2B | 6 | 0.014251781 |
| TULP2 | 6 | 0.014251781 |
| AC097636.1 | 6 | 0.014251781 |
| PBX1 | 6 | 0.014251781 |
| TAAR1 | 6 | 0.014251781 |
| FASTKD2 | 6 | 0.014251781 |
| TBX10 | 6 | 0.014251781 |
| TYMP | 6 | 0.014251781 |
| OR8H3 | 6 | 0.014251781 |
| SPSB4 | 6 | 0.014251781 |
| ADAMTSL5 | 6 | 0.014251781 |
| NFS1 | 6 | 0.014251781 |
| CEACAM16 | 6 | 0.014251781 |
| TLR6 | 6 | 0.014251781 |
| SIGLEC7 | 6 | 0.014251781 |
| OXSR1 | 6 | 0.014251781 |
| PSG6 | 6 | 0.014251781 |
| UQCC1 | 6 | 0.014251781 |
| AFG1L | 6 | 0.014251781 |
| TNFAIP1 | 6 | 0.014251781 |
| SERPINB3 | 6 | 0.014251781 |
| TADA2B | 6 | 0.014251781 |
| GCM1 | 6 | 0.014251781 |
| LRRC8D | 6 | 0.014251781 |
| MRPL2 | 6 | 0.014251781 |
| NEU1 | 6 | 0.014251781 |
| ASB14 | 6 | 0.014251781 |
| PARP2 | 6 | 0.014251781 |
| TESMIN | 6 | 0.014251781 |
| POU4F3 | 6 | 0.014251781 |
| RBMS1 | 6 | 0.014251781 |
| GNAI2 | 6 | 0.014251781 |
| KBTBD8 | 6 | 0.014251781 |
| FSCN1 | 6 | 0.014251781 |
| RASGRP1 | 6 | 0.014251781 |
| STK38 | 6 | 0.014251781 |
| MYOG | 6 | 0.014251781 |
| APOBEC3B | 6 | 0.014251781 |
| EYA2 | 6 | 0.014251781 |
| STXBP4 | 6 | 0.014251781 |
| MAGEA6 | 6 | 0.014251781 |
| HGD | 6 | 0.014251781 |
| CYP2W1 | 6 | 0.014251781 |
| IL6 | 6 | 0.014251781 |
| TAFA1 | 6 | 0.014251781 |
| NUP88 | 6 | 0.014251781 |
| PSMD12 | 6 | 0.014251781 |
| TTC27 | 6 | 0.014251781 |
| PRELP | 6 | 0.014251781 |
| ELMOD1 | 6 | 0.014251781 |
| GLIPR1 | 6 | 0.014251781 |
| NUDT13 | 6 | 0.014251781 |
| ARFGAP2 | 6 | 0.014251781 |
| PSMD6 | 6 | 0.014251781 |
| TMEM185A | 6 | 0.014251781 |
| ZCCHC7 | 6 | 0.014251781 |
| SLC25A39 | 6 | 0.014251781 |
| CSTF1 | 6 | 0.014251781 |
| HTR3C | 6 | 0.014251781 |
| AKR1B10 | 6 | 0.014251781 |
| SLC25A46 | 6 | 0.014251781 |
| CASP7 | 6 | 0.014251781 |
| SHH | 6 | 0.014251781 |
| LRRC4 | 6 | 0.014251781 |
| CABLES1 | 6 | 0.014251781 |
| PBX3 | 6 | 0.014251781 |
| KRT27 | 6 | 0.014251781 |
| ZPBP | 6 | 0.014251781 |
| HOXA6 | 6 | 0.014251781 |
| ATAD1 | 6 | 0.014251781 |
| FAM155B | 6 | 0.014251781 |
| FOXR1 | 6 | 0.014251781 |
| ZNF277 | 6 | 0.014251781 |
| ANKRD53 | 6 | 0.014251781 |
| ADAD2 | 6 | 0.014251781 |
| H2AC20 | 6 | 0.014251781 |
| ZNF764 | 6 | 0.014251781 |
| RUFY4 | 6 | 0.014251781 |
| CNTD1 | 6 | 0.014251781 |
| C11orf80 | 6 | 0.014251781 |
| CFHR2 | 6 | 0.014251781 |
| B3GALT4 | 6 | 0.014251781 |
| SLC22A11 | 6 | 0.014251781 |
| VSTM4 | 6 | 0.014251781 |
| RSPO4 | 6 | 0.014251781 |
| ARHGAP12 | 6 | 0.014251781 |
| OVCH2 | 6 | 0.014251781 |
| IGHV3-20 | 6 | 0.014251781 |
| PRSS54 | 6 | 0.014251781 |
| CYP11A1 | 6 | 0.014251781 |
| CD14 | 6 | 0.014251781 |
| SLC17A2 | 6 | 0.014251781 |
| COG7 | 6 | 0.014251781 |
| TFDP3 | 6 | 0.014251781 |
| GNPAT | 6 | 0.014251781 |
| CORO1A | 6 | 0.014251781 |
| RHCE | 6 | 0.014251781 |
| MSR1 | 6 | 0.014251781 |
| VN1R1 | 6 | 0.014251781 |
| LRRC30 | 6 | 0.014251781 |
| OR7D2 | 6 | 0.014251781 |
| ASB7 | 6 | 0.014251781 |
| FOXA2 | 6 | 0.014251781 |
| TRUB2 | 6 | 0.014251781 |
| BSCL2 | 6 | 0.014251781 |
| NCDN | 6 | 0.014251781 |
| TAS2R30 | 6 | 0.014251781 |
| SYNDIG1 | 6 | 0.014251781 |
| VRK1 | 6 | 0.014251781 |
| NR2E3 | 6 | 0.014251781 |
| LGALS9 | 6 | 0.014251781 |
| IRX2 | 6 | 0.014251781 |
| RNASE11 | 6 | 0.014251781 |
| PHYHIPL | 6 | 0.014251781 |
| SLC25A25 | 6 | 0.014251781 |
| HAP1 | 6 | 0.014251781 |
| TBX19 | 6 | 0.014251781 |
| CYRIA | 6 | 0.014251781 |
| BAP1 | 6 | 0.014251781 |
| MCMBP | 6 | 0.014251781 |
| BTBD9 | 6 | 0.014251781 |
| SNTA1 | 6 | 0.014251781 |
| KIF3B | 6 | 0.014251781 |
| CLUL1 | 6 | 0.014251781 |
| NOS1AP | 6 | 0.014251781 |
| ZNF696 | 6 | 0.014251781 |
| TAPT1 | 6 | 0.014251781 |
| PLSCR5 | 6 | 0.014251781 |
| RDH16 | 6 | 0.014251781 |
| PADI4 | 6 | 0.014251781 |
| FKBP8 | 6 | 0.014251781 |
| INSYN1 | 6 | 0.014251781 |
| NUFIP2 | 6 | 0.014251781 |
| NELFB | 6 | 0.014251781 |
| NPHS2 | 6 | 0.014251781 |
| CLK1 | 6 | 0.014251781 |
| ATRIP | 6 | 0.014251781 |
| MSX1 | 6 | 0.014251781 |
| TUBA1B | 6 | 0.014251781 |
| OR8K5 | 6 | 0.014251781 |
| POU3F3 | 6 | 0.014251781 |
| CLK4 | 6 | 0.014251781 |
| PCYOX1 | 6 | 0.014251781 |
| GPS2 | 6 | 0.014251781 |
| STK25 | 6 | 0.014251781 |
| NPLOC4 | 6 | 0.014251781 |
| PLSCR4 | 6 | 0.014251781 |
| FCHSD2 | 6 | 0.014251781 |
| PROCA1 | 6 | 0.014251781 |
| DDC | 6 | 0.014251781 |
| INKA2 | 6 | 0.014251781 |
| ZNF547 | 6 | 0.014251781 |
| NFKBIZ | 6 | 0.014251781 |
| FAM234A | 6 | 0.014251781 |
| PAFAH2 | 6 | 0.014251781 |
| SPRY1 | 6 | 0.014251781 |
| HOXC13 | 6 | 0.014251781 |
| JPH4 | 6 | 0.014251781 |
| TTC8 | 6 | 0.014251781 |
| NUP58 | 6 | 0.014251781 |
| ACTR6 | 6 | 0.014251781 |
| SMUG1 | 6 | 0.014251781 |
| KRTAP4-3 | 6 | 0.014251781 |
| RLBP1 | 6 | 0.014251781 |
| MMAA | 6 | 0.014251781 |
| ERO1B | 6 | 0.014251781 |
| SLC6A6 | 6 | 0.014251781 |
| BEST2 | 6 | 0.014251781 |
| SSTR3 | 6 | 0.014251781 |
| IFNA13 | 6 | 0.014251781 |
| COQ9 | 6 | 0.014251781 |
| RNF10 | 6 | 0.014251781 |
| CKAP2L | 6 | 0.014251781 |
| HTR2A | 6 | 0.014251781 |
| VEGFB | 6 | 0.014251781 |
| KRTAP6-2 | 6 | 0.014251781 |
| SERPINA11 | 6 | 0.014251781 |
| RBM4B | 6 | 0.014251781 |
| CHMP7 | 6 | 0.014251781 |
| LY6G6F-LY6G6D | 6 | 0.014251781 |
| STEAP1 | 6 | 0.014251781 |
| ENKUR | 6 | 0.014251781 |
| ATAD3A | 6 | 0.014251781 |
| CRB2 | 6 | 0.014251781 |
| DLX4 | 6 | 0.014251781 |
| TMOD4 | 6 | 0.014251781 |
| DDX20 | 6 | 0.014251781 |
| LMAN1 | 6 | 0.014251781 |
| HIC1 | 6 | 0.014251781 |
| DNAJC5B | 6 | 0.014251781 |
| ZNF440 | 6 | 0.014251781 |
| HARS1 | 6 | 0.014251781 |
| CNIH1 | 6 | 0.014251781 |
| KHDRBS1 | 6 | 0.014251781 |
| DPPA2 | 6 | 0.014251781 |
| MBD3L1 | 6 | 0.014251781 |
| OR52K1 | 6 | 0.014251781 |
| LPAR1 | 6 | 0.014251781 |
| VPS72 | 6 | 0.014251781 |
| ASB3 | 6 | 0.014251781 |
| MAPKAP1 | 6 | 0.014251781 |
| AKT1S1 | 6 | 0.014251781 |
| GIT1 | 6 | 0.014251781 |
| BPNT1 | 6 | 0.014251781 |
| CNOT6 | 6 | 0.014251781 |
| CARM1 | 6 | 0.014251781 |
| GGN | 6 | 0.014251781 |
| GPR183 | 6 | 0.014251781 |
| TBC1D20 | 6 | 0.014251781 |
| MS4A14 | 6 | 0.014251781 |
| KLF5 | 6 | 0.014251781 |
| SLC25A42 | 6 | 0.014251781 |
| ZDHHC2 | 6 | 0.014251781 |
| LIN7A | 6 | 0.014251781 |
| MISP | 6 | 0.014251781 |
| IRF4 | 6 | 0.014251781 |
| CRY1 | 6 | 0.014251781 |
| ZNF70 | 6 | 0.014251781 |
| CAMK4 | 6 | 0.014251781 |
| PJA1 | 6 | 0.014251781 |
| DTX3L | 6 | 0.014251781 |
| PRMT8 | 6 | 0.014251781 |
| SUPT20HL1 | 6 | 0.014251781 |
| MFSD1 | 6 | 0.014251781 |
| OR52N5 | 6 | 0.014251781 |
| PPM1N | 6 | 0.014251781 |
| CBX1 | 6 | 0.014251781 |
| ZNF792 | 6 | 0.014251781 |
| SLC14A1 | 6 | 0.014251781 |
| GALNT18 | 6 | 0.014251781 |
| TMC8 | 6 | 0.014251781 |
| SLC18B1 | 6 | 0.014251781 |
| RNF207 | 6 | 0.014251781 |
| AMMECR1 | 6 | 0.014251781 |
| CBX4 | 6 | 0.014251781 |
| C19orf57 | 6 | 0.014251781 |
| ENO1 | 6 | 0.014251781 |
| GLRA2 | 6 | 0.014251781 |
| DCP2 | 6 | 0.014251781 |
| HOMER2 | 6 | 0.014251781 |
| SPATC1L | 6 | 0.014251781 |
| FOXO3 | 6 | 0.014251781 |
| IL10RB | 6 | 0.014251781 |
| ZBTB12 | 6 | 0.014251781 |
| HSPA12B | 6 | 0.014251781 |
| FBXO4 | 6 | 0.014251781 |
| OR2H1 | 6 | 0.014251781 |
| ATF7IP2 | 6 | 0.014251781 |
| EXOC8 | 6 | 0.014251781 |
| NPY5R | 6 | 0.014251781 |
| RASGEF1A | 6 | 0.014251781 |
| AVPI1 | 6 | 0.014251781 |
| TRIM62 | 6 | 0.014251781 |
| SET | 6 | 0.014251781 |
| KLHL8 | 6 | 0.014251781 |
| FUT11 | 6 | 0.014251781 |
| HCAR3 | 6 | 0.014251781 |
| MIER2 | 6 | 0.014251781 |
| PPP1R15B | 6 | 0.014251781 |
| TNFRSF11A | 6 | 0.014251781 |
| PHKG1 | 6 | 0.014251781 |
| KCNJ9 | 6 | 0.014251781 |
| ZDHHC20 | 6 | 0.014251781 |
| PPP4R3B | 6 | 0.014251781 |
| NLK | 6 | 0.014251781 |
| DUSP6 | 6 | 0.014251781 |
| CPVL | 6 | 0.014251781 |
| KLK9 | 6 | 0.014251781 |
| IDO1 | 6 | 0.014251781 |
| NT5C3A | 6 | 0.014251781 |
| CDK8 | 6 | 0.014251781 |
| ULBP1 | 6 | 0.014251781 |
| MYF5 | 6 | 0.014251781 |
| SAMSN1 | 6 | 0.014251781 |
| ECHDC2 | 6 | 0.014251781 |
| RPA4 | 6 | 0.014251781 |
| RCVRN | 6 | 0.014251781 |
| BCL10 | 6 | 0.014251781 |
| MTFR1L | 6 | 0.014251781 |
| ZBTB7A | 6 | 0.014251781 |
| SH3GLB2 | 6 | 0.014251781 |
| WNT2B | 6 | 0.014251781 |
| ATXN7L2 | 6 | 0.014251781 |
| STK11 | 6 | 0.014251781 |
| IRAK1 | 6 | 0.014251781 |
| PPM1H | 6 | 0.014251781 |
| GSDMC | 6 | 0.014251781 |
| KRT38 | 6 | 0.014251781 |
| STK11IP | 6 | 0.014251781 |
| TAT | 6 | 0.014251781 |
| HCN3 | 6 | 0.014251781 |
| FBXL17 | 6 | 0.014251781 |
| MRNIP | 6 | 0.014251781 |
| SLC6A16 | 6 | 0.014251781 |
| MMS19 | 6 | 0.014251781 |
| CCDC198 | 6 | 0.014251781 |
| WDSUB1 | 6 | 0.014251781 |
| BIN1 | 6 | 0.014251781 |
| KIAA0930 | 6 | 0.014251781 |
| HSPA9 | 6 | 0.014251781 |
| STK32A | 6 | 0.014251781 |
| TOM1L2 | 6 | 0.014251781 |
| CALN1 | 6 | 0.014251781 |
| TMBIM4 | 6 | 0.014251781 |
| ICA1L | 6 | 0.014251781 |
| ZNF346 | 6 | 0.014251781 |
| PRIM1 | 6 | 0.014251781 |
| GLB1L | 6 | 0.014251781 |
| C1orf87 | 6 | 0.014251781 |
| KIF22 | 6 | 0.014251781 |
| RNGTT | 6 | 0.014251781 |
| CRISP1 | 6 | 0.014251781 |
| SNX27 | 6 | 0.014251781 |
| EML6 | 6 | 0.014251781 |
| TTC24 | 6 | 0.014251781 |
| ALDH2 | 6 | 0.014251781 |
| PARVG | 6 | 0.014251781 |
| QTRT2 | 6 | 0.014251781 |
| ZNF563 | 6 | 0.014251781 |
| ITGB5 | 6 | 0.014251781 |
| MIDN | 6 | 0.014251781 |
| ANKRD13B | 6 | 0.014251781 |
| DCLRE1C | 6 | 0.014251781 |
| SMC6 | 6 | 0.014251781 |
| CNN2 | 6 | 0.014251781 |
| MEN1 | 6 | 0.014251781 |
| HEPACAM | 6 | 0.014251781 |
| FOXP4 | 6 | 0.014251781 |
| TSPAN19 | 6 | 0.014251781 |
| DDX52 | 6 | 0.014251781 |
| VWC2L | 6 | 0.014251781 |
| ZNF770 | 6 | 0.014251781 |
| LRRC52 | 6 | 0.014251781 |
| WNT10B | 6 | 0.014251781 |
| CDH3 | 6 | 0.014251781 |
| OR52L1 | 6 | 0.014251781 |
| ARHGEF19 | 6 | 0.014251781 |
| ASB17 | 6 | 0.014251781 |
| NPEPL1 | 6 | 0.014251781 |
| AHCYL1 | 6 | 0.014251781 |
| KRT16 | 6 | 0.014251781 |
| TTLL6 | 6 | 0.014251781 |
| AMY2A | 6 | 0.014251781 |
| ZNF322 | 6 | 0.014251781 |
| FNDC4 | 6 | 0.014251781 |
| CYTH3 | 6 | 0.014251781 |
| SUSD1 | 6 | 0.014251781 |
| CCT3 | 6 | 0.014251781 |
| TMEM30A | 6 | 0.014251781 |
| LARP6 | 6 | 0.014251781 |
| P2RY2 | 6 | 0.014251781 |
| KLHDC4 | 6 | 0.014251781 |
| ZNF524 | 6 | 0.014251781 |
| GMPR | 6 | 0.014251781 |
| AWAT1 | 6 | 0.014251781 |
| SHC4 | 6 | 0.014251781 |
| FEZ1 | 6 | 0.014251781 |
| GSG1 | 6 | 0.014251781 |
| OR4C3 | 6 | 0.014251781 |
| SUPT7L | 6 | 0.014251781 |
| ORAI2 | 6 | 0.014251781 |
| GOT1 | 6 | 0.014251781 |
| ME3 | 6 | 0.014251781 |
| ELOVL4 | 6 | 0.014251781 |
| LRRN4 | 6 | 0.014251781 |
| C1QTNF1 | 6 | 0.014251781 |
| GPR182 | 6 | 0.014251781 |
| ECHDC1 | 6 | 0.014251781 |
| SLC35G5 | 6 | 0.014251781 |
| RIN1 | 6 | 0.014251781 |
| PSG3 | 6 | 0.014251781 |
| BASP1 | 6 | 0.014251781 |
| ZNF883 | 6 | 0.014251781 |
| MRPS30 | 6 | 0.014251781 |
| LHFPL3 | 6 | 0.014251781 |
| PAX4 | 6 | 0.014251781 |
| LRRC15 | 6 | 0.014251781 |
| TAS2R10 | 6 | 0.014251781 |
| LCE1A | 6 | 0.014251781 |
| OR2A12 | 6 | 0.014251781 |
| TCEAL4 | 6 | 0.014251781 |
| RRM1 | 6 | 0.014251781 |
| SLC35F1 | 6 | 0.014251781 |
| CD34 | 6 | 0.014251781 |
| LGI3 | 6 | 0.014251781 |
| SS18L1 | 6 | 0.014251781 |
| ZC4H2 | 6 | 0.014251781 |
| MORN1 | 6 | 0.014251781 |
| SPATA5L1 | 6 | 0.014251781 |
| RORC | 6 | 0.014251781 |
| NCAPH2 | 6 | 0.014251781 |
| ACVR1C | 6 | 0.014251781 |
| P2RX7 | 6 | 0.014251781 |
| CBLN4 | 6 | 0.014251781 |
| LMNTD1 | 6 | 0.014251781 |
| ZNF41 | 6 | 0.014251781 |
| NUP160 | 6 | 0.014251781 |
| ZFAND4 | 6 | 0.014251781 |
| GPR88 | 6 | 0.014251781 |
| RASSF5 | 6 | 0.014251781 |
| AKAP17A | 6 | 0.014251781 |
| LIN54 | 6 | 0.014251781 |
| ZNF713 | 6 | 0.014251781 |
| PIK3R3 | 6 | 0.014251781 |
| ZCWPW1 | 6 | 0.014251781 |
| VARS2 | 6 | 0.014251781 |
| IL20RA | 6 | 0.014251781 |
| RASL10B | 6 | 0.014251781 |
| PALD1 | 6 | 0.014251781 |
| CA7 | 6 | 0.014251781 |
| CETN3 | 6 | 0.014251781 |
| GSN | 6 | 0.014251781 |
| CAV3 | 6 | 0.014251781 |
| CAPN9 | 6 | 0.014251781 |
| TENT5A | 6 | 0.014251781 |
| CYP19A1 | 6 | 0.014251781 |
| ACSM3 | 6 | 0.014251781 |
| PPARD | 6 | 0.014251781 |
| LYZL1 | 6 | 0.014251781 |
| ESRRB | 6 | 0.014251781 |
| CD28 | 6 | 0.014251781 |
| SNX21 | 6 | 0.014251781 |
| LENG1 | 6 | 0.014251781 |
| SLX4IP | 6 | 0.014251781 |
| ARVCF | 6 | 0.014251781 |
| IKZF3 | 6 | 0.014251781 |
| GAS2L2 | 6 | 0.014251781 |
| ANXA8 | 6 | 0.014251781 |
| UROS | 6 | 0.014251781 |
| FAM186A | 6 | 0.014251781 |
| GPR6 | 6 | 0.014251781 |
| TIMM50 | 6 | 0.014251781 |
| TAAR5 | 6 | 0.014251781 |
| RFX3 | 6 | 0.014251781 |
| TLR2 | 6 | 0.014251781 |
| IFIT5 | 6 | 0.014251781 |
| CCIN | 6 | 0.014251781 |
| RSBN1L | 6 | 0.014251781 |
| DCPS | 6 | 0.014251781 |
| SH2B1 | 6 | 0.014251781 |
| CDADC1 | 6 | 0.014251781 |
| PDZK1 | 6 | 0.014251781 |
| KRT15 | 6 | 0.014251781 |
| CCDC24 | 6 | 0.014251781 |
| TASP1 | 6 | 0.014251781 |
| TRMT9B | 6 | 0.014251781 |
| CREM | 6 | 0.014251781 |
| HEXIM2 | 6 | 0.014251781 |
| SH3GL2 | 6 | 0.014251781 |
| MAGEB1 | 6 | 0.014251781 |
| CCNQ | 6 | 0.014251781 |
| XPNPEP1 | 6 | 0.014251781 |
| TSNAXIP1 | 6 | 0.014251781 |
| OLIG3 | 6 | 0.014251781 |
| ZNF157 | 6 | 0.014251781 |
| GPR173 | 6 | 0.014251781 |
| FBXO28 | 6 | 0.014251781 |
| CNDP1 | 6 | 0.014251781 |
| RLIM | 6 | 0.014251781 |
| PRSS36 | 6 | 0.014251781 |
| GPR137B | 6 | 0.014251781 |
| TRIM69 | 6 | 0.014251781 |
| TMPPE | 6 | 0.014251781 |
| EXTL1 | 6 | 0.014251781 |
| TENT4A | 6 | 0.014251781 |
| KRTAP13-1 | 6 | 0.014251781 |
| TAFA5 | 6 | 0.014251781 |
| C7orf25 | 6 | 0.014251781 |
| VAT1L | 6 | 0.014251781 |
| RAB40AL | 6 | 0.014251781 |
| RBCK1 | 6 | 0.014251781 |
| TMEM109 | 6 | 0.014251781 |
| SAG | 6 | 0.014251781 |
| COLEC11 | 6 | 0.014251781 |
| FAM71C | 6 | 0.014251781 |
| STYK1 | 6 | 0.014251781 |
| WWP2 | 6 | 0.014251781 |
| NKD1 | 6 | 0.014251781 |
| LINGO3 | 6 | 0.014251781 |
| CORO1C | 6 | 0.014251781 |
| FERD3L | 6 | 0.014251781 |
| CCM2 | 6 | 0.014251781 |
| SDF4 | 6 | 0.014251781 |
| GBP4 | 6 | 0.014251781 |
| CHN1 | 6 | 0.014251781 |
| AGXT | 6 | 0.014251781 |
| IP6K3 | 6 | 0.014251781 |
| OR56A1 | 6 | 0.014251781 |
| GPRC5C | 6 | 0.014251781 |
| ACSM5 | 6 | 0.014251781 |
| NOP56 | 6 | 0.014251781 |
| ZCWPW2 | 6 | 0.014251781 |
| ANKZF1 | 6 | 0.014251781 |
| POU2F2 | 6 | 0.014251781 |
| CHEK2 | 6 | 0.014251781 |
| CYP51A1 | 6 | 0.014251781 |
| B4GALT1 | 6 | 0.014251781 |
| AP3M2 | 6 | 0.014251781 |
| ACSS1 | 6 | 0.014251781 |
| FAM118B | 6 | 0.014251781 |
| INPP5B | 6 | 0.014251781 |
| SIAE | 6 | 0.014251781 |
| E2F3 | 6 | 0.014251781 |
| IGFBP4 | 6 | 0.014251781 |
| TRIM52 | 6 | 0.014251781 |
| EZH1 | 6 | 0.014251781 |
| SYK | 6 | 0.014251781 |
| AQP8 | 6 | 0.014251781 |
| B3GNT7 | 6 | 0.014251781 |
| ZNF461 | 6 | 0.014251781 |
| MICU3 | 6 | 0.014251781 |
| DPF2 | 6 | 0.014251781 |
| DTWD2 | 6 | 0.014251781 |
| HAPLN3 | 6 | 0.014251781 |
| KRT13 | 6 | 0.014251781 |
| GYG2 | 6 | 0.014251781 |
| OR5B17 | 6 | 0.014251781 |
| WEE2 | 6 | 0.014251781 |
| MANBA | 6 | 0.014251781 |
| OPN3 | 6 | 0.014251781 |
| BBOX1 | 6 | 0.014251781 |
| RERG | 6 | 0.014251781 |
| UBE2E2 | 6 | 0.014251781 |
| SPATA6L | 6 | 0.014251781 |
| BCKDHB | 6 | 0.014251781 |
| HNRNPA1 | 6 | 0.014251781 |
| MED15 | 6 | 0.014251781 |
| MAGIX | 6 | 0.014251781 |
| AMDHD1 | 6 | 0.014251781 |
| ITPRIPL2 | 6 | 0.014251781 |
| FMN1 | 6 | 0.014251781 |
| PDHA1 | 6 | 0.014251781 |
| TEX30 | 6 | 0.014251781 |
| RAMP3 | 6 | 0.014251781 |
| SPATA9 | 6 | 0.014251781 |
| CYP4V2 | 6 | 0.014251781 |
| SLC25A14 | 6 | 0.014251781 |
| MIPEP | 6 | 0.014251781 |
| PTGIS | 6 | 0.014251781 |
| DCN | 6 | 0.014251781 |
| NKAP | 6 | 0.014251781 |
| GOLIM4 | 6 | 0.014251781 |
| DEFB116 | 6 | 0.014251781 |
| RHBDF1 | 6 | 0.014251781 |
| ACAT1 | 6 | 0.014251781 |
| SMARCD1 | 6 | 0.014251781 |
| LIPG | 6 | 0.014251781 |
| FERMT3 | 6 | 0.014251781 |
| STXBP2 | 6 | 0.014251781 |
| ZWILCH | 6 | 0.014251781 |
| RASSF4 | 6 | 0.014251781 |
| SHISA5 | 6 | 0.014251781 |
| C5orf22 | 6 | 0.014251781 |
| RIMS3 | 6 | 0.014251781 |
| PSRC1 | 6 | 0.014251781 |
| ACAA1 | 6 | 0.014251781 |
| SLC1A5 | 6 | 0.014251781 |
| KAT2A | 6 | 0.014251781 |
| FPR1 | 6 | 0.014251781 |
| ZNF783 | 6 | 0.014251781 |
| LAMP3 | 6 | 0.014251781 |
| USH1C | 6 | 0.014251781 |
| FAHD2A | 6 | 0.014251781 |
| PPP1R3C | 6 | 0.014251781 |
| TRAF6 | 6 | 0.014251781 |
| ZNF674 | 6 | 0.014251781 |
| HOXA3 | 6 | 0.014251781 |
| FAM110C | 6 | 0.014251781 |
| ACTR5 | 6 | 0.014251781 |
| SEL1L | 6 | 0.014251781 |
| NCK2 | 6 | 0.014251781 |
| PKNOX2 | 6 | 0.014251781 |
| ZNF160 | 6 | 0.014251781 |
| DELE1 | 6 | 0.014251781 |
| GCFC2 | 6 | 0.014251781 |
| IFI35 | 6 | 0.014251781 |
| SCD | 6 | 0.014251781 |
| GPANK1 | 6 | 0.014251781 |
| AQP2 | 6 | 0.014251781 |
| CHI3L1 | 6 | 0.014251781 |
| IGHG3 | 6 | 0.014251781 |
| SLC10A5 | 6 | 0.014251781 |
| USP46 | 6 | 0.014251781 |
| TMEM245 | 6 | 0.014251781 |
| RELT | 6 | 0.014251781 |
| AMACR | 6 | 0.014251781 |
| ZNF821 | 6 | 0.014251781 |
| GAS2L3 | 6 | 0.014251781 |
| ANKRD40 | 6 | 0.014251781 |
| EXOC3L1 | 6 | 0.014251781 |
| GNA11 | 6 | 0.014251781 |
| KATNBL1 | 6 | 0.014251781 |
| TCEAL6 | 6 | 0.014251781 |
| SPON2 | 6 | 0.014251781 |
| HTRA2 | 6 | 0.014251781 |
| ZNF57 | 6 | 0.014251781 |
| NMUR1 | 6 | 0.014251781 |
| DIRAS1 | 6 | 0.014251781 |
| PMFBP1 | 6 | 0.014251781 |
| CUTC | 6 | 0.014251781 |
| CXCR5 | 6 | 0.014251781 |
| TRIML2 | 6 | 0.014251781 |
| BHMT2 | 6 | 0.014251781 |
| WBP1 | 6 | 0.014251781 |
| ANGPTL7 | 6 | 0.014251781 |
| DPAGT1 | 6 | 0.014251781 |
| MAP3K3 | 6 | 0.014251781 |
| IFNGR2 | 6 | 0.014251781 |
| CDKN2AIP | 6 | 0.014251781 |
| INPP5K | 6 | 0.014251781 |
| KCNK3 | 6 | 0.014251781 |
| DDX49 | 6 | 0.014251781 |
| SLC26A6 | 6 | 0.014251781 |
| PRKN | 6 | 0.014251781 |
| RSL1D1 | 6 | 0.014251781 |
| OR2H2 | 6 | 0.014251781 |
| CDK5RAP1 | 6 | 0.014251781 |
| BTBD8 | 6 | 0.014251781 |
| OR1E2 | 6 | 0.014251781 |
| PATZ1 | 6 | 0.014251781 |
| LILRA1 | 6 | 0.014251781 |
| EDAR | 6 | 0.014251781 |
| RECQL | 6 | 0.014251781 |
| C1GALT1C1L | 6 | 0.014251781 |
| COL26A1 | 6 | 0.014251781 |
| SSBP2 | 6 | 0.014251781 |
| AKAP8L | 6 | 0.014251781 |
| ACBD5 | 6 | 0.014251781 |
| STX19 | 6 | 0.014251781 |
| RPS6KB1 | 6 | 0.014251781 |
| SEPTIN11 | 6 | 0.014251781 |
| SPATA13 | 6 | 0.014251781 |
| GPR45 | 6 | 0.014251781 |
| ZNF596 | 6 | 0.014251781 |
| RPL3 | 6 | 0.014251781 |
| APOD | 6 | 0.014251781 |
| NEBL | 6 | 0.014251781 |
| RAD51AP1 | 6 | 0.014251781 |
| KCNK17 | 6 | 0.014251781 |
| ADA | 6 | 0.014251781 |
| WDR5B | 6 | 0.014251781 |
| CLPP | 6 | 0.014251781 |
| WT1 | 6 | 0.014251781 |
| TRIB1 | 6 | 0.014251781 |
| SMTNL1 | 6 | 0.014251781 |
| TPMT | 6 | 0.014251781 |
| ORAI1 | 6 | 0.014251781 |
| RGS11 | 6 | 0.014251781 |
| ANKMY2 | 6 | 0.014251781 |
| LAX1 | 6 | 0.014251781 |
| RBSN | 6 | 0.014251781 |
| SLC39A11 | 6 | 0.014251781 |
| CHI3L2 | 6 | 0.014251781 |
| PIM2 | 6 | 0.014251781 |
| AWAT2 | 6 | 0.014251781 |
| ZNF551 | 6 | 0.014251781 |
| RELL2 | 6 | 0.014251781 |
| ZNF383 | 6 | 0.014251781 |
| PTPMT1 | 6 | 0.014251781 |
| CPNE6 | 6 | 0.014251781 |
| SCARF2 | 6 | 0.014251781 |
| MYNN | 6 | 0.014251781 |
| TOX2 | 6 | 0.014251781 |
| SIX6 | 6 | 0.014251781 |
| KCTD15 | 6 | 0.014251781 |
| ZNF711 | 6 | 0.014251781 |
| SYTL3 | 6 | 0.014251781 |
| IGKV1-5 | 6 | 0.014251781 |
| WDR93 | 6 | 0.014251781 |
| CCDC61 | 6 | 0.014251781 |
| BTBD16 | 6 | 0.014251781 |
| GPR4 | 6 | 0.014251781 |
| SULT1C3 | 6 | 0.014251781 |
| MMP28 | 6 | 0.014251781 |
| DNAJC8 | 6 | 0.014251781 |
| GCM2 | 6 | 0.014251781 |
| CER1 | 6 | 0.014251781 |
| ATP5F1A | 6 | 0.014251781 |
| TRIM68 | 6 | 0.014251781 |
| ATE1 | 6 | 0.014251781 |
| FUT3 | 6 | 0.014251781 |
| SLC13A5 | 6 | 0.014251781 |
| ZNF846 | 6 | 0.014251781 |
| TFB1M | 6 | 0.014251781 |
| B3GALNT1 | 6 | 0.014251781 |
| MPPED2 | 6 | 0.014251781 |
| DDX51 | 6 | 0.014251781 |
| TCP1 | 6 | 0.014251781 |
| CA14 | 6 | 0.014251781 |
| ECHS1 | 6 | 0.014251781 |
| BIRC3 | 6 | 0.014251781 |
| BAX | 6 | 0.014251781 |
| P2RX2 | 6 | 0.014251781 |
| TMEM119 | 6 | 0.014251781 |
| MYCL | 6 | 0.014251781 |
| IL6R | 6 | 0.014251781 |
| ZNF639 | 6 | 0.014251781 |
| FAM220A | 6 | 0.014251781 |
| IL26 | 6 | 0.014251781 |
| TANK | 6 | 0.014251781 |
| ZNF25 | 6 | 0.014251781 |
| RERGL | 6 | 0.014251781 |
| IGHG4 | 6 | 0.014251781 |
| NR3C1 | 6 | 0.014251781 |
| CXorf58 | 6 | 0.014251781 |
| CAPSL | 6 | 0.014251781 |
| CYP2S1 | 6 | 0.014251781 |
| TRIM43 | 6 | 0.014251781 |
| VGLL3 | 6 | 0.014251781 |
| GAB2 | 6 | 0.014251781 |
| CYREN | 6 | 0.014251781 |
| MAP3K20 | 6 | 0.014251781 |
| KCNRG | 6 | 0.014251781 |
| LEPROTL1 | 6 | 0.014251781 |
| NSF | 6 | 0.014251781 |
| FAM234B | 6 | 0.014251781 |
| PI4KB | 6 | 0.014251781 |
| NUDT18 | 6 | 0.014251781 |
| RUVBL2 | 6 | 0.014251781 |
| HINFP | 6 | 0.014251781 |
| PRSS53 | 6 | 0.014251781 |
| ZNF22 | 6 | 0.014251781 |
| ABO | 6 | 0.014251781 |
| METTL25 | 6 | 0.014251781 |
| LIX1 | 6 | 0.014251781 |
| PAX8 | 6 | 0.014251781 |
| ZNF708 | 6 | 0.014251781 |
| DDX4 | 6 | 0.014251781 |
| SLC6A9 | 6 | 0.014251781 |
| TBCC | 6 | 0.014251781 |
| RABGGTB | 6 | 0.014251781 |
| SETMAR | 6 | 0.014251781 |
| CHST12 | 6 | 0.014251781 |
| COPS3 | 6 | 0.014251781 |
| OR52H1 | 6 | 0.014251781 |
| NEK9 | 6 | 0.014251781 |
| ELK4 | 6 | 0.014251781 |
| LAD1 | 6 | 0.014251781 |
| LYVE1 | 6 | 0.014251781 |
| HMBS | 6 | 0.014251781 |
| STX4 | 6 | 0.014251781 |
| VPS36 | 6 | 0.014251781 |
| HS2ST1 | 6 | 0.014251781 |
| EGLN2 | 6 | 0.014251781 |
| SLC25A3 | 6 | 0.014251781 |
| GNRH1 | 6 | 0.014251781 |
| CMTM5 | 6 | 0.014251781 |
| IRX4 | 6 | 0.014251781 |
| ZNF276 | 6 | 0.014251781 |
| KRTAP12-1 | 6 | 0.014251781 |
| LOX | 6 | 0.014251781 |
| SERPINB11 | 6 | 0.014251781 |
| UBIAD1 | 6 | 0.014251781 |
| IDI2 | 6 | 0.014251781 |
| BACE1 | 6 | 0.014251781 |
| DEPDC4 | 6 | 0.014251781 |
| XRCC1 | 6 | 0.014251781 |
| CCDC78 | 6 | 0.014251781 |
| MRPL16 | 6 | 0.014251781 |
| CD84 | 6 | 0.014251781 |
| HNRNPH1 | 6 | 0.014251781 |
| KHDC3L | 6 | 0.014251781 |
| CDK5R1 | 6 | 0.014251781 |
| SLC17A4 | 6 | 0.014251781 |
| FES | 6 | 0.014251781 |
| CEACAM5 | 6 | 0.014251781 |
| CMKLR1 | 6 | 0.014251781 |
| AP4M1 | 6 | 0.014251781 |
| A1CF | 6 | 0.014251781 |
| PKMYT1 | 6 | 0.014251781 |
| ULBP3 | 6 | 0.014251781 |
| MAGT1 | 6 | 0.014251781 |
| MEST | 6 | 0.014251781 |
| NUMBL | 6 | 0.014251781 |
| EFCAB3 | 6 | 0.014251781 |
| RBBP5 | 6 | 0.014251781 |
| CASP1 | 6 | 0.014251781 |
| YBX2 | 6 | 0.014251781 |
| SPDYE1 | 6 | 0.014251781 |
| MKS1 | 6 | 0.014251781 |
| ADIPOR2 | 6 | 0.014251781 |
| SEPTIN6 | 6 | 0.014251781 |
| LAMP2 | 6 | 0.014251781 |
| TAS2R40 | 6 | 0.014251781 |
| FAM50B | 6 | 0.014251781 |
| SPOCK2 | 6 | 0.014251781 |
| ZNF230 | 6 | 0.014251781 |
| CLEC12B | 6 | 0.014251781 |
| VPS9D1 | 6 | 0.014251781 |
| GPR153 | 6 | 0.014251781 |
| BSG | 6 | 0.014251781 |
| GFM2 | 6 | 0.014251781 |
| FOXL1 | 6 | 0.014251781 |
| ARSI | 6 | 0.014251781 |
| ZC3HC1 | 6 | 0.014251781 |
| PHYH | 6 | 0.014251781 |
| ZBED2 | 6 | 0.014251781 |
| PDZD3 | 6 | 0.014251781 |
| P2RX4 | 6 | 0.014251781 |
| OR5V1 | 6 | 0.014251781 |
| AGT | 6 | 0.014251781 |
| GDI2 | 6 | 0.014251781 |
| ZADH2 | 6 | 0.014251781 |
| FAM184B | 6 | 0.014251781 |
| OR52N1 | 6 | 0.014251781 |
| ZNF233 | 6 | 0.014251781 |
| IL27RA | 6 | 0.014251781 |
| STAMBP | 6 | 0.014251781 |
| BFSP2 | 6 | 0.014251781 |
| OPRPN | 6 | 0.014251781 |
| IL11RA | 6 | 0.014251781 |
| OR5H2 | 6 | 0.014251781 |
| WDR44 | 6 | 0.014251781 |
| MPP5 | 6 | 0.014251781 |
| TRIM72 | 6 | 0.014251781 |
| SLC39A6 | 6 | 0.014251781 |
| OLAH | 6 | 0.014251781 |
| KIAA1841 | 6 | 0.014251781 |
| CRISPLD2 | 6 | 0.014251781 |
| RASSF9 | 6 | 0.014251781 |
| ACMSD | 6 | 0.014251781 |
| SLC6A7 | 6 | 0.014251781 |
| MTERF3 | 6 | 0.014251781 |
| SPATA32 | 6 | 0.014251781 |
| GAP43 | 6 | 0.014251781 |
| OR13A1 | 6 | 0.014251781 |
| RAB3IP | 6 | 0.014251781 |
| STK40 | 6 | 0.014251781 |
| SH3D19 | 6 | 0.014251781 |
| CMTM3 | 6 | 0.014251781 |
| HOXD1 | 6 | 0.014251781 |
| DPH2 | 6 | 0.014251781 |
| SPRED3 | 6 | 0.014251781 |
| AFAP1L1 | 6 | 0.014251781 |
| POLR2F | 6 | 0.014251781 |
| FANCE | 6 | 0.014251781 |
| ZNF285 | 6 | 0.014251781 |
| UPP1 | 6 | 0.014251781 |
| TMA16 | 6 | 0.014251781 |
| TACR1 | 6 | 0.014251781 |
| DRGX | 6 | 0.014251781 |
| ADORA2A | 6 | 0.014251781 |
| TUBA1A | 6 | 0.014251781 |
| ALG5 | 6 | 0.014251781 |
| PHF21B | 6 | 0.014251781 |
| CCL14 | 6 | 0.014251781 |
| DNASE1L2 | 6 | 0.014251781 |
| ADAP2 | 6 | 0.014251781 |
| DAPK2 | 6 | 0.014251781 |
| IQCB1 | 6 | 0.014251781 |
| PLEKHO1 | 6 | 0.014251781 |
| DVL3 | 6 | 0.014251781 |
| ASTE1 | 6 | 0.014251781 |
| SERTAD4 | 6 | 0.014251781 |
| SPAST | 6 | 0.014251781 |
| TMEM259 | 6 | 0.014251781 |
| OR2A14 | 6 | 0.014251781 |
| FAM153B | 6 | 0.014251781 |
| ZNF358 | 6 | 0.014251781 |
| PATL1 | 6 | 0.014251781 |
| SULT1B1 | 6 | 0.014251781 |
| CD63 | 6 | 0.014251781 |
| LY6K | 6 | 0.014251781 |
| GLI4 | 6 | 0.014251781 |
| SULT1E1 | 6 | 0.014251781 |
| DNAJA3 | 6 | 0.014251781 |
| PHYHIP | 6 | 0.014251781 |
| HAVCR1 | 6 | 0.014251781 |
| PRR35 | 6 | 0.014251781 |
| TMEM52B | 6 | 0.014251781 |
| OR2AK2 | 6 | 0.014251781 |
| KDELR1 | 6 | 0.014251781 |
| GSTM3 | 6 | 0.014251781 |
| THBD | 6 | 0.014251781 |
| RASGRP4 | 6 | 0.014251781 |
| ABCF1 | 6 | 0.014251781 |
| ZNF324 | 6 | 0.014251781 |
| APLF | 6 | 0.014251781 |
| C17orf98 | 6 | 0.014251781 |
| TMEM183A | 6 | 0.014251781 |
| CDR1 | 6 | 0.014251781 |
| ZHX1 | 6 | 0.014251781 |
| PGD | 6 | 0.014251781 |
| ZNF253 | 6 | 0.014251781 |
| ING5 | 6 | 0.014251781 |
| GPS1 | 6 | 0.014251781 |
| SNX9 | 6 | 0.014251781 |
| TRIM65 | 6 | 0.014251781 |
| ANKRD49 | 6 | 0.014251781 |
| VDAC1 | 6 | 0.014251781 |
| TTC6 | 6 | 0.014251781 |
| TRHR | 6 | 0.014251781 |
| FGFR3 | 6 | 0.014251781 |
| RCOR2 | 6 | 0.014251781 |
| EPN3 | 6 | 0.014251781 |
| DNPEP | 6 | 0.014251781 |
| UBAP2 | 6 | 0.014251781 |
| IGBP1P2 | 6 | 0.014251781 |
| NOL9 | 6 | 0.014251781 |
| FKBPL | 6 | 0.014251781 |
| MCAM | 6 | 0.014251781 |
| TRAF1 | 6 | 0.014251781 |
| CDCP1 | 6 | 0.014251781 |
| MOGAT2 | 6 | 0.014251781 |
| ZP1 | 6 | 0.014251781 |
| UCMA | 6 | 0.014251781 |
| XPNPEP2 | 6 | 0.014251781 |
| NME9 | 6 | 0.014251781 |
| TMEM35A | 6 | 0.014251781 |
| SCRN3 | 6 | 0.014251781 |
| RTN4RL1 | 6 | 0.014251781 |
| ADK | 6 | 0.014251781 |
| MED26 | 6 | 0.014251781 |
| TCF7L1 | 6 | 0.014251781 |
| TIGD4 | 6 | 0.014251781 |
| PDLIM2 | 6 | 0.014251781 |
| MBNL2 | 6 | 0.014251781 |
| SDCBP2 | 6 | 0.014251781 |
| RUSC1 | 6 | 0.014251781 |
| HSP90AB1 | 6 | 0.014251781 |
| MUS81 | 6 | 0.014251781 |
| NXPH1 | 6 | 0.014251781 |
| NSL1 | 6 | 0.014251781 |
| TMPRSS11B | 6 | 0.014251781 |
| CCDC191 | 6 | 0.014251781 |
| RBM14 | 6 | 0.014251781 |
| CSAD | 6 | 0.014251781 |
| TDRD10 | 6 | 0.014251781 |
| ERN1 | 6 | 0.014251781 |
| NGFR | 6 | 0.014251781 |
| OR14I1 | 6 | 0.014251781 |
| OXSM | 6 | 0.014251781 |
| FAM166A | 6 | 0.014251781 |
| SPATA2L | 6 | 0.014251781 |
| TMED8 | 6 | 0.014251781 |
| OCIAD1 | 6 | 0.014251781 |
| TUBB2B | 6 | 0.014251781 |
| RIDA | 6 | 0.014251781 |
| ALLC | 6 | 0.014251781 |
| LRRC14B | 6 | 0.014251781 |
| RBM39 | 6 | 0.014251781 |
| KIR3DL2 | 6 | 0.014251781 |
| PTPN2 | 6 | 0.014251781 |
| CDKL4 | 6 | 0.014251781 |
| SEC23A | 6 | 0.014251781 |
| RNF41 | 6 | 0.014251781 |
| OR6Q1 | 6 | 0.014251781 |
| RAB11FIP5 | 6 | 0.014251781 |
| SNRNP35 | 6 | 0.014251781 |
| RSAD1 | 6 | 0.014251781 |
| STUB1 | 6 | 0.014251781 |
| FCN3 | 6 | 0.014251781 |
| H2AC1 | 6 | 0.014251781 |
| SMYD5 | 6 | 0.014251781 |
| SKAP1 | 6 | 0.014251781 |
| POLE2 | 6 | 0.014251781 |
| IGHA1 | 6 | 0.014251781 |
| MYOT | 6 | 0.014251781 |
| SERPIND1 | 6 | 0.014251781 |
| PYGO1 | 6 | 0.014251781 |
| RAB17 | 6 | 0.014251781 |
| MOCS3 | 6 | 0.014251781 |
| FGF20 | 6 | 0.014251781 |
| API5 | 6 | 0.014251781 |
| CKMT2 | 6 | 0.014251781 |
| RBM45 | 6 | 0.014251781 |
| NECTIN3 | 6 | 0.014251781 |
| CLPX | 6 | 0.014251781 |
| HNRNPCL1 | 6 | 0.014251781 |
| GNL3L | 6 | 0.014251781 |
| OR5B2 | 6 | 0.014251781 |
| TMEM135 | 6 | 0.014251781 |
| GTF2H1 | 6 | 0.014251781 |
| LPAR5 | 6 | 0.014251781 |
| MMP17 | 6 | 0.014251781 |
| CEACAM4 | 6 | 0.014251781 |
| NCL | 6 | 0.014251781 |
| ACP1 | 6 | 0.014251781 |
| ACVR1 | 6 | 0.014251781 |
| PGLYRP2 | 6 | 0.014251781 |
| PRKCE | 6 | 0.014251781 |
| SLC46A2 | 6 | 0.014251781 |
| LIMS2 | 6 | 0.014251781 |
| CDKL3 | 6 | 0.014251781 |
| RCSD1 | 6 | 0.014251781 |
| MINPP1 | 6 | 0.014251781 |
| OR5D13 | 6 | 0.014251781 |
| LPP | 6 | 0.014251781 |
| F10 | 6 | 0.014251781 |
| RALGPS2 | 6 | 0.014251781 |
| IL1B | 6 | 0.014251781 |
| ANXA11 | 6 | 0.014251781 |
| ARMC9 | 6 | 0.014251781 |
| AIM2 | 6 | 0.014251781 |
| NT5C3B | 6 | 0.014251781 |
| TSNARE1 | 6 | 0.014251781 |
| KANSL2 | 6 | 0.014251781 |
| UBE2NL | 6 | 0.014251781 |
| ATG4B | 6 | 0.014251781 |
| HMGCLL1 | 6 | 0.014251781 |
| LIAS | 6 | 0.014251781 |
| IGHV4-28 | 6 | 0.014251781 |
| H3C2 | 6 | 0.014251781 |
| WARS1 | 6 | 0.014251781 |
| EOGT | 6 | 0.014251781 |
| RFX1 | 6 | 0.014251781 |
| SP1 | 6 | 0.014251781 |
| TCTN1 | 6 | 0.014251781 |
| FZR1 | 6 | 0.014251781 |
| SLC25A24 | 6 | 0.014251781 |
| ATF7 | 6 | 0.014251781 |
| TMEM130 | 6 | 0.014251781 |
| ZNF362 | 6 | 0.014251781 |
| WDR88 | 6 | 0.014251781 |
| CCDC54 | 6 | 0.014251781 |
| LGI4 | 6 | 0.014251781 |
| GPATCH2L | 6 | 0.014251781 |
| NUTM2A | 6 | 0.014251781 |
| TFPI2 | 6 | 0.014251781 |
| MICU2 | 6 | 0.014251781 |
| TMEM171 | 6 | 0.014251781 |
| OR51I2 | 6 | 0.014251781 |
| PCED1B | 6 | 0.014251781 |
| WDFY4 | 6 | 0.014251781 |
| EFCAB14 | 6 | 0.014251781 |
| SUN3 | 6 | 0.014251781 |
| ZNF181 | 6 | 0.014251781 |
| WDR45B | 6 | 0.014251781 |
| ACADL | 6 | 0.014251781 |
| MATN1 | 6 | 0.014251781 |
| SLC25A51 | 6 | 0.014251781 |
| IL4R | 6 | 0.014251781 |
| CRTC2 | 6 | 0.014251781 |
| ACOXL | 6 | 0.014251781 |
| ATG16L1 | 6 | 0.014251781 |
| GPR151 | 6 | 0.014251781 |
| ST3GAL3 | 6 | 0.014251781 |
| TRIM36 | 6 | 0.014251781 |
| GYPA | 6 | 0.014251781 |
| SRSF5 | 6 | 0.014251781 |
| ZDHHC4 | 6 | 0.014251781 |
| TAAR2 | 6 | 0.014251781 |
| ADH1C | 6 | 0.014251781 |
| UBQLN4 | 6 | 0.014251781 |
| CYB5R4 | 6 | 0.014251781 |
| POLR3D | 6 | 0.014251781 |
| LDB2 | 6 | 0.014251781 |
| HNRNPAB | 6 | 0.014251781 |
| P3H1 | 6 | 0.014251781 |
| CAMK1G | 6 | 0.014251781 |
| GJA5 | 6 | 0.014251781 |
| HRH3 | 6 | 0.014251781 |
| METAP1D | 6 | 0.014251781 |
| EEPD1 | 6 | 0.014251781 |
| GSDMA | 6 | 0.014251781 |
| OR11L1 | 6 | 0.014251781 |
| TXLNG | 6 | 0.014251781 |
| SCML4 | 6 | 0.014251781 |
| TEPP | 5 | 0.011876485 |
| NUP43 | 5 | 0.011876485 |
| BCAS1 | 5 | 0.011876485 |
| RFPL3 | 5 | 0.011876485 |
| MFSD10 | 5 | 0.011876485 |
| HPR | 5 | 0.011876485 |
| EIF2B3 | 5 | 0.011876485 |
| MCOLN2 | 5 | 0.011876485 |
| CCDC71 | 5 | 0.011876485 |
| TMEM71 | 5 | 0.011876485 |
| BABAM1 | 5 | 0.011876485 |
| AC126283.2 | 5 | 0.011876485 |
| STKLD1 | 5 | 0.011876485 |
| C14orf180 | 5 | 0.011876485 |
| PGGHG | 5 | 0.011876485 |
| BATF3 | 5 | 0.011876485 |
| FGF3 | 5 | 0.011876485 |
| MOB3A | 5 | 0.011876485 |
| DMTF1 | 5 | 0.011876485 |
| FAM13B | 5 | 0.011876485 |
| GPR83 | 5 | 0.011876485 |
| JMJD6 | 5 | 0.011876485 |
| RUSF1 | 5 | 0.011876485 |
| C2orf73 | 5 | 0.011876485 |
| AOAH | 5 | 0.011876485 |
| TSC22D2 | 5 | 0.011876485 |
| FRMD7 | 5 | 0.011876485 |
| RSKR | 5 | 0.011876485 |
| SH3GLB1 | 5 | 0.011876485 |
| OPHN1 | 5 | 0.011876485 |
| VAX2 | 5 | 0.011876485 |
| TRIM13 | 5 | 0.011876485 |
| DXO | 5 | 0.011876485 |
| RWDD1 | 5 | 0.011876485 |
| FGGY | 5 | 0.011876485 |
| MAS1 | 5 | 0.011876485 |
| LRRC3B | 5 | 0.011876485 |
| KLRD1 | 5 | 0.011876485 |
| CDC34 | 5 | 0.011876485 |
| ABLIM1 | 5 | 0.011876485 |
| TKTL1 | 5 | 0.011876485 |
| TRIM10 | 5 | 0.011876485 |
| FAM118A | 5 | 0.011876485 |
| PKIB | 5 | 0.011876485 |
| GPR137 | 5 | 0.011876485 |
| OR8B3 | 5 | 0.011876485 |
| ST6GAL1 | 5 | 0.011876485 |
| PLEKHS1 | 5 | 0.011876485 |
| BEX1 | 5 | 0.011876485 |
| LACRT | 5 | 0.011876485 |
| IFNA16 | 5 | 0.011876485 |
| TIMM44 | 5 | 0.011876485 |
| LAIR1 | 5 | 0.011876485 |
| HYAL1 | 5 | 0.011876485 |
| TTLL13P | 5 | 0.011876485 |
| ZFY | 5 | 0.011876485 |
| HEXB | 5 | 0.011876485 |
| PREB | 5 | 0.011876485 |
| CLTA | 5 | 0.011876485 |
| JADE3 | 5 | 0.011876485 |
| PIGT | 5 | 0.011876485 |
| H1-4 | 5 | 0.011876485 |
| TMEM156 | 5 | 0.011876485 |
| CCK | 5 | 0.011876485 |
| MTFR2 | 5 | 0.011876485 |
| FRG2 | 5 | 0.011876485 |
| SRSF12 | 5 | 0.011876485 |
| FGL1 | 5 | 0.011876485 |
| POU1F1 | 5 | 0.011876485 |
| RSPH10B | 5 | 0.011876485 |
| NUCB2 | 5 | 0.011876485 |
| C12orf29 | 5 | 0.011876485 |
| OR2C1 | 5 | 0.011876485 |
| BRMS1L | 5 | 0.011876485 |
| SSX5 | 5 | 0.011876485 |
| CCT4 | 5 | 0.011876485 |
| POLR3C | 5 | 0.011876485 |
| CDC42EP4 | 5 | 0.011876485 |
| TEKT1 | 5 | 0.011876485 |
| KCTD12 | 5 | 0.011876485 |
| ERVFRD-1 | 5 | 0.011876485 |
| GFAP | 5 | 0.011876485 |
| FAM107B | 5 | 0.011876485 |
| APOBEC3G | 5 | 0.011876485 |
| PRB2 | 5 | 0.011876485 |
| OR5L1 | 5 | 0.011876485 |
| ACOT11 | 5 | 0.011876485 |
| MAP2K6 | 5 | 0.011876485 |
| TMEM121 | 5 | 0.011876485 |
| DHRS2 | 5 | 0.011876485 |
| GPR174 | 5 | 0.011876485 |
| TLX1 | 5 | 0.011876485 |
| DEDD2 | 5 | 0.011876485 |
| ZNF688 | 5 | 0.011876485 |
| FEN1 | 5 | 0.011876485 |
| RNH1 | 5 | 0.011876485 |
| C9orf72 | 5 | 0.011876485 |
| B4GALNT2 | 5 | 0.011876485 |
| BAIAP2 | 5 | 0.011876485 |
| IL9R | 5 | 0.011876485 |
| DUOXA2 | 5 | 0.011876485 |
| TIMP4 | 5 | 0.011876485 |
| OR1J2 | 5 | 0.011876485 |
| PRICKLE3 | 5 | 0.011876485 |
| CCDC185 | 5 | 0.011876485 |
| PRSS1 | 5 | 0.011876485 |
| WDR27 | 5 | 0.011876485 |
| LYL1 | 5 | 0.011876485 |
| OGFR | 5 | 0.011876485 |
| ATG101 | 5 | 0.011876485 |
| KRTAP10-4 | 5 | 0.011876485 |
| SLC43A2 | 5 | 0.011876485 |
| PDC | 5 | 0.011876485 |
| UBXN11 | 5 | 0.011876485 |
| B3GNT6 | 5 | 0.011876485 |
| VDAC2 | 5 | 0.011876485 |
| TWF1 | 5 | 0.011876485 |
| NAGS | 5 | 0.011876485 |
| RAB4B | 5 | 0.011876485 |
| ARMC8 | 5 | 0.011876485 |
| TOR1AIP2 | 5 | 0.011876485 |
| DIPK2A | 5 | 0.011876485 |
| IGKV3-15 | 5 | 0.011876485 |
| DPY19L1 | 5 | 0.011876485 |
| TEAD3 | 5 | 0.011876485 |
| UTY | 5 | 0.011876485 |
| TTC38 | 5 | 0.011876485 |
| PDCL3 | 5 | 0.011876485 |
| ECI1 | 5 | 0.011876485 |
| ZBTB25 | 5 | 0.011876485 |
| VSIG8 | 5 | 0.011876485 |
| KATNAL2 | 5 | 0.011876485 |
| SLC16A8 | 5 | 0.011876485 |
| FARS2 | 5 | 0.011876485 |
| ENTPD1 | 5 | 0.011876485 |
| RNPEPL1 | 5 | 0.011876485 |
| SPAG8 | 5 | 0.011876485 |
| FAAH2 | 5 | 0.011876485 |
| RMND1 | 5 | 0.011876485 |
| ANKRD10 | 5 | 0.011876485 |
| KDF1 | 5 | 0.011876485 |
| OR5J2 | 5 | 0.011876485 |
| DECR1 | 5 | 0.011876485 |
| ACAD10 | 5 | 0.011876485 |
| PPA2 | 5 | 0.011876485 |
| FLT3LG | 5 | 0.011876485 |
| ASPHD1 | 5 | 0.011876485 |
| CHRNE | 5 | 0.011876485 |
| IFNA8 | 5 | 0.011876485 |
| GLIS1 | 5 | 0.011876485 |
| ZNF664 | 5 | 0.011876485 |
| LRRC37B | 5 | 0.011876485 |
| STX5 | 5 | 0.011876485 |
| HSPA4 | 5 | 0.011876485 |
| MARCHF8 | 5 | 0.011876485 |
| KRT23 | 5 | 0.011876485 |
| MRI1 | 5 | 0.011876485 |
| ACR | 5 | 0.011876485 |
| NEK11 | 5 | 0.011876485 |
| SLC26A1 | 5 | 0.011876485 |
| KLK1 | 5 | 0.011876485 |
| FGF7 | 5 | 0.011876485 |
| CWH43 | 5 | 0.011876485 |
| DPF1 | 5 | 0.011876485 |
| GTPBP4 | 5 | 0.011876485 |
| KLHL28 | 5 | 0.011876485 |
| CDCA4 | 5 | 0.011876485 |
| DHX32 | 5 | 0.011876485 |
| TMEM215 | 5 | 0.011876485 |
| SLC39A14 | 5 | 0.011876485 |
| GLRA4 | 5 | 0.011876485 |
| ZSWIM1 | 5 | 0.011876485 |
| SRR | 5 | 0.011876485 |
| TIPARP | 5 | 0.011876485 |
| RAB3GAP1 | 5 | 0.011876485 |
| GMDS | 5 | 0.011876485 |
| SPINT1 | 5 | 0.011876485 |
| RCBTB1 | 5 | 0.011876485 |
| SLC1A2 | 5 | 0.011876485 |
| EDA2R | 5 | 0.011876485 |
| SLC35D3 | 5 | 0.011876485 |
| RNF126 | 5 | 0.011876485 |
| SEPTIN2 | 5 | 0.011876485 |
| SPIRE1 | 5 | 0.011876485 |
| MKRN1 | 5 | 0.011876485 |
| SCARA3 | 5 | 0.011876485 |
| WDR53 | 5 | 0.011876485 |
| ITPRIPL1 | 5 | 0.011876485 |
| CENPK | 5 | 0.011876485 |
| ATF2 | 5 | 0.011876485 |
| VMO1 | 5 | 0.011876485 |
| DHDDS | 5 | 0.011876485 |
| ETV3L | 5 | 0.011876485 |
| MACIR | 5 | 0.011876485 |
| NKAIN3 | 5 | 0.011876485 |
| MAP11 | 5 | 0.011876485 |
| OR6M1 | 5 | 0.011876485 |
| RHOT2 | 5 | 0.011876485 |
| ZNF555 | 5 | 0.011876485 |
| PDCL | 5 | 0.011876485 |
| IFIT1B | 5 | 0.011876485 |
| ERLIN1 | 5 | 0.011876485 |
| TRAF3IP2 | 5 | 0.011876485 |
| TIGD6 | 5 | 0.011876485 |
| CTSS | 5 | 0.011876485 |
| ARL2BP | 5 | 0.011876485 |
| FRRS1 | 5 | 0.011876485 |
| SORD | 5 | 0.011876485 |
| ZNF274 | 5 | 0.011876485 |
| ZCCHC13 | 5 | 0.011876485 |
| CBR3 | 5 | 0.011876485 |
| FEM1B | 5 | 0.011876485 |
| SLC25A19 | 5 | 0.011876485 |
| OR2G2 | 5 | 0.011876485 |
| POLD2 | 5 | 0.011876485 |
| RBM22 | 5 | 0.011876485 |
| NKD2 | 5 | 0.011876485 |
| ANXA4 | 5 | 0.011876485 |
| SMARCE1 | 5 | 0.011876485 |
| OR8G1 | 5 | 0.011876485 |
| TMEM92 | 5 | 0.011876485 |
| OR6C4 | 5 | 0.011876485 |
| HDDC2 | 5 | 0.011876485 |
| ASB13 | 5 | 0.011876485 |
| OR51G2 | 5 | 0.011876485 |
| MESD | 5 | 0.011876485 |
| ANKRD1 | 5 | 0.011876485 |
| CROT | 5 | 0.011876485 |
| CTSZ | 5 | 0.011876485 |
| STAM2 | 5 | 0.011876485 |
| FAM114A2 | 5 | 0.011876485 |
| OR11H6 | 5 | 0.011876485 |
| PDHX | 5 | 0.011876485 |
| EHF | 5 | 0.011876485 |
| SF3A2 | 5 | 0.011876485 |
| MORN3 | 5 | 0.011876485 |
| SDR39U1 | 5 | 0.011876485 |
| ALDH3A2 | 5 | 0.011876485 |
| OR2F2 | 5 | 0.011876485 |
| SNRNP48 | 5 | 0.011876485 |
| CRHBP | 5 | 0.011876485 |
| PHYHD1 | 5 | 0.011876485 |
| OR1B1 | 5 | 0.011876485 |
| USP49 | 5 | 0.011876485 |
| MDM2 | 5 | 0.011876485 |
| CABP5 | 5 | 0.011876485 |
| DERL1 | 5 | 0.011876485 |
| ADIPOQ | 5 | 0.011876485 |
| KRT83 | 5 | 0.011876485 |
| ARNT | 5 | 0.011876485 |
| OR1D5 | 5 | 0.011876485 |
| CXCL5 | 5 | 0.011876485 |
| IGHA2 | 5 | 0.011876485 |
| SAMD15 | 5 | 0.011876485 |
| RND2 | 5 | 0.011876485 |
| ZBTB1 | 5 | 0.011876485 |
| OR4S2 | 5 | 0.011876485 |
| GPAA1 | 5 | 0.011876485 |
| SYCE2 | 5 | 0.011876485 |
| ZPBP2 | 5 | 0.011876485 |
| ADRA1B | 5 | 0.011876485 |
| AAMP | 5 | 0.011876485 |
| APLP2 | 5 | 0.011876485 |
| RBM5 | 5 | 0.011876485 |
| SLC25A5 | 5 | 0.011876485 |
| ADH6 | 5 | 0.011876485 |
| OSGIN2 | 5 | 0.011876485 |
| TH | 5 | 0.011876485 |
| LRRC23 | 5 | 0.011876485 |
| SDR9C7 | 5 | 0.011876485 |
| ODR4 | 5 | 0.011876485 |
| DRAP1 | 5 | 0.011876485 |
| CFHR3 | 5 | 0.011876485 |
| USP12 | 5 | 0.011876485 |
| PTGER4 | 5 | 0.011876485 |
| NEIL3 | 5 | 0.011876485 |
| FBXO7 | 5 | 0.011876485 |
| TFDP2 | 5 | 0.011876485 |
| AQP4 | 5 | 0.011876485 |
| LIPH | 5 | 0.011876485 |
| CCDC89 | 5 | 0.011876485 |
| ARL6 | 5 | 0.011876485 |
| JAML | 5 | 0.011876485 |
| AMBP | 5 | 0.011876485 |
| PWWP2A | 5 | 0.011876485 |
| RGMA | 5 | 0.011876485 |
| ALKBH4 | 5 | 0.011876485 |
| FTCD | 5 | 0.011876485 |
| FASTK | 5 | 0.011876485 |
| PDCD1 | 5 | 0.011876485 |
| ARG2 | 5 | 0.011876485 |
| MCM9 | 5 | 0.011876485 |
| USP30 | 5 | 0.011876485 |
| NKX3-1 | 5 | 0.011876485 |
| SUMF1 | 5 | 0.011876485 |
| REXO4 | 5 | 0.011876485 |
| FPR2 | 5 | 0.011876485 |
| SLC24A5 | 5 | 0.011876485 |
| RHOBTB2 | 5 | 0.011876485 |
| PPP1R21 | 5 | 0.011876485 |
| ZCCHC9 | 5 | 0.011876485 |
| PPP2R3C | 5 | 0.011876485 |
| TXNIP | 5 | 0.011876485 |
| EFNA4 | 5 | 0.011876485 |
| CAT | 5 | 0.011876485 |
| UFSP1 | 5 | 0.011876485 |
| HNF4A | 5 | 0.011876485 |
| PRKAB1 | 5 | 0.011876485 |
| AC127029.3 | 5 | 0.011876485 |
| DPY19L3 | 5 | 0.011876485 |
| SDC2 | 5 | 0.011876485 |
| SLC5A11 | 5 | 0.011876485 |
| AIMP2 | 5 | 0.011876485 |
| TSPAN14 | 5 | 0.011876485 |
| FLAD1 | 5 | 0.011876485 |
| TAS2R39 | 5 | 0.011876485 |
| OR1N1 | 5 | 0.011876485 |
| TMEM214 | 5 | 0.011876485 |
| ANKRD13C | 5 | 0.011876485 |
| DCAF12 | 5 | 0.011876485 |
| RUFY2 | 5 | 0.011876485 |
| TNFAIP8L2 | 5 | 0.011876485 |
| HEY2 | 5 | 0.011876485 |
| PRSS8 | 5 | 0.011876485 |
| LNP1 | 5 | 0.011876485 |
| KRTAP19-5 | 5 | 0.011876485 |
| SIGLEC12 | 5 | 0.011876485 |
| NFKB2 | 5 | 0.011876485 |
| CALHM1 | 5 | 0.011876485 |
| GFOD2 | 5 | 0.011876485 |
| MSANTD4 | 5 | 0.011876485 |
| CA12 | 5 | 0.011876485 |
| ATP6V0D1 | 5 | 0.011876485 |
| PACC1 | 5 | 0.011876485 |
| BDKRB1 | 5 | 0.011876485 |
| MGAT4A | 5 | 0.011876485 |
| DEPTOR | 5 | 0.011876485 |
| KASH5 | 5 | 0.011876485 |
| AURKB | 5 | 0.011876485 |
| BPIFA3 | 5 | 0.011876485 |
| CCDC91 | 5 | 0.011876485 |
| MFRP | 5 | 0.011876485 |
| OR10A4 | 5 | 0.011876485 |
| PADI2 | 5 | 0.011876485 |
| TMEM74B | 5 | 0.011876485 |
| ATP6AP2 | 5 | 0.011876485 |
| ACTG1 | 5 | 0.011876485 |
| PEX5 | 5 | 0.011876485 |
| OR51F2 | 5 | 0.011876485 |
| CLDN17 | 5 | 0.011876485 |
| PODNL1 | 5 | 0.011876485 |
| RFPL4B | 5 | 0.011876485 |
| CNNM4 | 5 | 0.011876485 |
| SSTR2 | 5 | 0.011876485 |
| TRIM75P | 5 | 0.011876485 |
| ZNF165 | 5 | 0.011876485 |
| SVOP | 5 | 0.011876485 |
| SLC22A9 | 5 | 0.011876485 |
| RAB11B | 5 | 0.011876485 |
| TGFBI | 5 | 0.011876485 |
| SERPINB13 | 5 | 0.011876485 |
| DEGS1 | 5 | 0.011876485 |
| POC1B | 5 | 0.011876485 |
| ZNF32 | 5 | 0.011876485 |
| CERS6 | 5 | 0.011876485 |
| SLC19A3 | 5 | 0.011876485 |
| REG1A | 5 | 0.011876485 |
| APOL3 | 5 | 0.011876485 |
| PHLDA1 | 5 | 0.011876485 |
| PSMC3 | 5 | 0.011876485 |
| DNAJB14 | 5 | 0.011876485 |
| H2BC4 | 5 | 0.011876485 |
| NCF2 | 5 | 0.011876485 |
| THAP4 | 5 | 0.011876485 |
| GPAM | 5 | 0.011876485 |
| CYB561 | 5 | 0.011876485 |
| ACP5 | 5 | 0.011876485 |
| MINDY1 | 5 | 0.011876485 |
| OR4D1 | 5 | 0.011876485 |
| CRTAM | 5 | 0.011876485 |
| UPB1 | 5 | 0.011876485 |
| ZNF81 | 5 | 0.011876485 |
| TCTN2 | 5 | 0.011876485 |
| FAM217B | 5 | 0.011876485 |
| KLHDC7A | 5 | 0.011876485 |
| FBRS | 5 | 0.011876485 |
| SLC30A10 | 5 | 0.011876485 |
| GRHL3 | 5 | 0.011876485 |
| MUC15 | 5 | 0.011876485 |
| MB21D2 | 5 | 0.011876485 |
| AQP10 | 5 | 0.011876485 |
| GALM | 5 | 0.011876485 |
| PLEKHA1 | 5 | 0.011876485 |
| RCC1 | 5 | 0.011876485 |
| HLA-E | 5 | 0.011876485 |
| SRPRA | 5 | 0.011876485 |
| FBXO32 | 5 | 0.011876485 |
| SCAMP1 | 5 | 0.011876485 |
| TXNDC16 | 5 | 0.011876485 |
| XCR1 | 5 | 0.011876485 |
| NFAT5 | 5 | 0.011876485 |
| MS4A15 | 5 | 0.011876485 |
| TEF | 5 | 0.011876485 |
| SPINDOC | 5 | 0.011876485 |
| YIPF7 | 5 | 0.011876485 |
| DKC1 | 5 | 0.011876485 |
| TRIM48 | 5 | 0.011876485 |
| KPNA2 | 5 | 0.011876485 |
| PM20D2 | 5 | 0.011876485 |
| OR9Q1 | 5 | 0.011876485 |
| WNT4 | 5 | 0.011876485 |
| CCNE1 | 5 | 0.011876485 |
| NPNT | 5 | 0.011876485 |
| GPRC5A | 5 | 0.011876485 |
| MYOZ1 | 5 | 0.011876485 |
| BEGAIN | 5 | 0.011876485 |
| EXTL2 | 5 | 0.011876485 |
| OR5AC2 | 5 | 0.011876485 |
| TPPP2 | 5 | 0.011876485 |
| HOMER3 | 5 | 0.011876485 |
| PRR5L | 5 | 0.011876485 |
| HHLA2 | 5 | 0.011876485 |
| METRNL | 5 | 0.011876485 |
| RNF208 | 5 | 0.011876485 |
| CD1B | 5 | 0.011876485 |
| PTPRA | 5 | 0.011876485 |
| PIP5KL1 | 5 | 0.011876485 |
| IFNAR2 | 5 | 0.011876485 |
| OR13G1 | 5 | 0.011876485 |
| CHD1L | 5 | 0.011876485 |
| CSRNP2 | 5 | 0.011876485 |
| DIXDC1 | 5 | 0.011876485 |
| PAPSS1 | 5 | 0.011876485 |
| PGM2L1 | 5 | 0.011876485 |
| MYMK | 5 | 0.011876485 |
| NXPH3 | 5 | 0.011876485 |
| TRIM63 | 5 | 0.011876485 |
| CRISP3 | 5 | 0.011876485 |
| ZBTB10 | 5 | 0.011876485 |
| ILK | 5 | 0.011876485 |
| RGS17 | 5 | 0.011876485 |
| DDX3X | 5 | 0.011876485 |
| ATP6V1C1 | 5 | 0.011876485 |
| SLC47A2 | 5 | 0.011876485 |
| FDXR | 5 | 0.011876485 |
| AC008676.3 | 5 | 0.011876485 |
| SLC27A4 | 5 | 0.011876485 |
| TNNI1 | 5 | 0.011876485 |
| PDIA4 | 5 | 0.011876485 |
| CUL4A | 5 | 0.011876485 |
| FAM53A | 5 | 0.011876485 |
| POMK | 5 | 0.011876485 |
| CACNB3 | 5 | 0.011876485 |
| PPP2R2D | 5 | 0.011876485 |
| NTHL1 | 5 | 0.011876485 |
| IL13RA1 | 5 | 0.011876485 |
| RPAP3 | 5 | 0.011876485 |
| CDKN1B | 5 | 0.011876485 |
| CYP3A5 | 5 | 0.011876485 |
| TRMT10C | 5 | 0.011876485 |
| MLXIP | 5 | 0.011876485 |
| CDC37 | 5 | 0.011876485 |
| ZDHHC1 | 5 | 0.011876485 |
| SRSF1 | 5 | 0.011876485 |
| IDI1 | 5 | 0.011876485 |
| EDARADD | 5 | 0.011876485 |
| XRRA1 | 5 | 0.011876485 |
| TINF2 | 5 | 0.011876485 |
| CLEC11A | 5 | 0.011876485 |
| NTN5 | 5 | 0.011876485 |
| STX2 | 5 | 0.011876485 |
| NR1I2 | 5 | 0.011876485 |
| DUSP29 | 5 | 0.011876485 |
| FAM204A | 5 | 0.011876485 |
| ZXDC | 5 | 0.011876485 |
| PALM | 5 | 0.011876485 |
| TNFRSF9 | 5 | 0.011876485 |
| TEX13A | 5 | 0.011876485 |
| C5AR2 | 5 | 0.011876485 |
| C7orf33 | 5 | 0.011876485 |
| PTX4 | 5 | 0.011876485 |
| GHITM | 5 | 0.011876485 |
| MDH1 | 5 | 0.011876485 |
| OR10T2 | 5 | 0.011876485 |
| BCL2L13 | 5 | 0.011876485 |
| CDK20 | 5 | 0.011876485 |
| SERPINI1 | 5 | 0.011876485 |
| CCL23 | 5 | 0.011876485 |
| SERINC2 | 5 | 0.011876485 |
| OR56B1 | 5 | 0.011876485 |
| RTL10 | 5 | 0.011876485 |
| CEACAM20 | 5 | 0.011876485 |
| KCTD1 | 5 | 0.011876485 |
| ENDOD1 | 5 | 0.011876485 |
| HPS4 | 5 | 0.011876485 |
| PODXL2 | 5 | 0.011876485 |
| KRTAP10-11 | 5 | 0.011876485 |
| FAM189A2 | 5 | 0.011876485 |
| ANXA7 | 5 | 0.011876485 |
| RSRC1 | 5 | 0.011876485 |
| P2RY14 | 5 | 0.011876485 |
| CYBB | 5 | 0.011876485 |
| TMEM163 | 5 | 0.011876485 |
| SERPINA4 | 5 | 0.011876485 |
| PRDX6 | 5 | 0.011876485 |
| ATP6AP1 | 5 | 0.011876485 |
| VPS26C | 5 | 0.011876485 |
| PDE12 | 5 | 0.011876485 |
| UBAC1 | 5 | 0.011876485 |
| TAF1B | 5 | 0.011876485 |
| OR6C76 | 5 | 0.011876485 |
| SELENOP | 5 | 0.011876485 |
| GATA5 | 5 | 0.011876485 |
| PAPSS2 | 5 | 0.011876485 |
| ENPP7 | 5 | 0.011876485 |
| TCP11L1 | 5 | 0.011876485 |
| CDC14B | 5 | 0.011876485 |
| BMP15 | 5 | 0.011876485 |
| PRPF38A | 5 | 0.011876485 |
| ACAT2 | 5 | 0.011876485 |
| TM2D2 | 5 | 0.011876485 |
| MTX1 | 5 | 0.011876485 |
| CPNE3 | 5 | 0.011876485 |
| PEDS1 | 5 | 0.011876485 |
| ALOX12 | 5 | 0.011876485 |
| DDX25 | 5 | 0.011876485 |
| MYADM | 5 | 0.011876485 |
| AQP9 | 5 | 0.011876485 |
| CTSL | 5 | 0.011876485 |
| DTWD1 | 5 | 0.011876485 |
| SURF6 | 5 | 0.011876485 |
| MAGEA1 | 5 | 0.011876485 |
| NEUROG1 | 5 | 0.011876485 |
| DLAT | 5 | 0.011876485 |
| IGLON5 | 5 | 0.011876485 |
| RIC8B | 5 | 0.011876485 |
| RETREG3 | 5 | 0.011876485 |
| SWAP70 | 5 | 0.011876485 |
| CCNJ | 5 | 0.011876485 |
| HP | 5 | 0.011876485 |
| ARFGAP1 | 5 | 0.011876485 |
| PRSS55 | 5 | 0.011876485 |
| ERI2 | 5 | 0.011876485 |
| SLC16A5 | 5 | 0.011876485 |
| TULP3 | 5 | 0.011876485 |
| PEX11G | 5 | 0.011876485 |
| SLAMF7 | 5 | 0.011876485 |
| ST6GALNAC1 | 5 | 0.011876485 |
| C11orf42 | 5 | 0.011876485 |
| OR2T35 | 5 | 0.011876485 |
| TMCO5A | 5 | 0.011876485 |
| SLC6A18 | 5 | 0.011876485 |
| CYB5R2 | 5 | 0.011876485 |
| OR1D2 | 5 | 0.011876485 |
| CANT1 | 5 | 0.011876485 |
| ZNF765 | 5 | 0.011876485 |
| FH | 5 | 0.011876485 |
| DHRS7C | 5 | 0.011876485 |
| CAVIN3 | 5 | 0.011876485 |
| TNFRSF10B | 5 | 0.011876485 |
| CXXC1 | 5 | 0.011876485 |
| CDK2 | 5 | 0.011876485 |
| RTL4 | 5 | 0.011876485 |
| MATN3 | 5 | 0.011876485 |
| DCDC2B | 5 | 0.011876485 |
| ALDH5A1 | 5 | 0.011876485 |
| HMCN2 | 5 | 0.011876485 |
| DPH1 | 5 | 0.011876485 |
| TTLL10 | 5 | 0.011876485 |
| SLC22A5 | 5 | 0.011876485 |
| WDR61 | 5 | 0.011876485 |
| RIOK2 | 5 | 0.011876485 |
| CDK18 | 5 | 0.011876485 |
| CLEC12A | 5 | 0.011876485 |
| ARRB1 | 5 | 0.011876485 |
| PSEN1 | 5 | 0.011876485 |
| PLEKHF1 | 5 | 0.011876485 |
| GART | 5 | 0.011876485 |
| TSPAN18 | 5 | 0.011876485 |
| GNAT3 | 5 | 0.011876485 |
| LTV1 | 5 | 0.011876485 |
| NUP62 | 5 | 0.011876485 |
| FNBP1L | 5 | 0.011876485 |
| CLVS1 | 5 | 0.011876485 |
| ZNF3 | 5 | 0.011876485 |
| CACNG6 | 5 | 0.011876485 |
| MRPL19 | 5 | 0.011876485 |
| KRTAP24-1 | 5 | 0.011876485 |
| HS1BP3 | 5 | 0.011876485 |
| SMPDL3B | 5 | 0.011876485 |
| WDR24 | 5 | 0.011876485 |
| FCRL2 | 5 | 0.011876485 |
| AGAP6 | 5 | 0.011876485 |
| HOGA1 | 5 | 0.011876485 |
| KCNJ15 | 5 | 0.011876485 |
| OR4K17 | 5 | 0.011876485 |
| RPL10L | 5 | 0.011876485 |
| PPT2 | 5 | 0.011876485 |
| MAGEA10 | 5 | 0.011876485 |
| TMPRSS5 | 5 | 0.011876485 |
| ERP27 | 5 | 0.011876485 |
| C17orf75 | 5 | 0.011876485 |
| CCDC33 | 5 | 0.011876485 |
| CUEDC1 | 5 | 0.011876485 |
| DPF3 | 5 | 0.011876485 |
| OR5B12 | 5 | 0.011876485 |
| TCN2 | 5 | 0.011876485 |
| MFSD4A | 5 | 0.011876485 |
| SERPINH1 | 5 | 0.011876485 |
| ZBTB34 | 5 | 0.011876485 |
| SPX | 5 | 0.011876485 |
| NHLH1 | 5 | 0.011876485 |
| LRRC14 | 5 | 0.011876485 |
| SPPL2A | 5 | 0.011876485 |
| SLC40A1 | 5 | 0.011876485 |
| GDAP1L1 | 5 | 0.011876485 |
| RAB3B | 5 | 0.011876485 |
| KCNAB1 | 5 | 0.011876485 |
| PNMA5 | 5 | 0.011876485 |
| TRIM47 | 5 | 0.011876485 |
| NOC2L | 5 | 0.011876485 |
| SLC7A14 | 5 | 0.011876485 |
| LURAP1 | 5 | 0.011876485 |
| OR1J4 | 5 | 0.011876485 |
| DDN | 5 | 0.011876485 |
| LNPK | 5 | 0.011876485 |
| ANXA9 | 5 | 0.011876485 |
| KCNMB4 | 5 | 0.011876485 |
| SYNC | 5 | 0.011876485 |
| LOXL1 | 5 | 0.011876485 |
| SLC25A18 | 5 | 0.011876485 |
| UBXN8 | 5 | 0.011876485 |
| USP37 | 5 | 0.011876485 |
| ZBTB45 | 5 | 0.011876485 |
| ELN | 5 | 0.011876485 |
| CCS | 5 | 0.011876485 |
| ADH1A | 5 | 0.011876485 |
| FABP2 | 5 | 0.011876485 |
| EVA1C | 5 | 0.011876485 |
| OR51B2 | 5 | 0.011876485 |
| DNAJC2 | 5 | 0.011876485 |
| GPR31 | 5 | 0.011876485 |
| IGF1 | 5 | 0.011876485 |
| OR4D11 | 5 | 0.011876485 |
| CNPPD1 | 5 | 0.011876485 |
| FRMD4B | 5 | 0.011876485 |
| A3GALT2 | 5 | 0.011876485 |
| ANXA2 | 5 | 0.011876485 |
| HDAC3 | 5 | 0.011876485 |
| CCDC102A | 5 | 0.011876485 |
| GSTA3 | 5 | 0.011876485 |
| ADCYAP1R1 | 5 | 0.011876485 |
| C14orf93 | 5 | 0.011876485 |
| ZNF469 | 5 | 0.011876485 |
| NRN1 | 5 | 0.011876485 |
| SPAM1 | 5 | 0.011876485 |
| ST7 | 5 | 0.011876485 |
| FBXL4 | 5 | 0.011876485 |
| RCL1 | 5 | 0.011876485 |
| XPC | 5 | 0.011876485 |
| BHLHE41 | 5 | 0.011876485 |
| CALR | 5 | 0.011876485 |
| FNTB | 5 | 0.011876485 |
| FANCB | 5 | 0.011876485 |
| TNFSF14 | 5 | 0.011876485 |
| GPX5 | 5 | 0.011876485 |
| RAB6C | 5 | 0.011876485 |
| ACD | 5 | 0.011876485 |
| TMEM198 | 5 | 0.011876485 |
| PCED1A | 5 | 0.011876485 |
| UGT2B28 | 5 | 0.011876485 |
| ZNF441 | 5 | 0.011876485 |
| STEAP3 | 5 | 0.011876485 |
| EZR | 5 | 0.011876485 |
| KRTAP15-1 | 5 | 0.011876485 |
| POU3F2 | 5 | 0.011876485 |
| TEC | 5 | 0.011876485 |
| DOK4 | 5 | 0.011876485 |
| PDCD6 | 5 | 0.011876485 |
| ZNF426 | 5 | 0.011876485 |
| MEOX2 | 5 | 0.011876485 |
| SLC6A19 | 5 | 0.011876485 |
| MRPS2 | 5 | 0.011876485 |
| HS3ST3A1 | 5 | 0.011876485 |
| PIGW | 5 | 0.011876485 |
| IGKV1D-8 | 5 | 0.011876485 |
| CCNJL | 5 | 0.011876485 |
| PAN2 | 5 | 0.011876485 |
| GLMP | 5 | 0.011876485 |
| CFAP52 | 5 | 0.011876485 |
| HFE | 5 | 0.011876485 |
| TMEM140 | 5 | 0.011876485 |
| CTTNBP2NL | 5 | 0.011876485 |
| KLK12 | 5 | 0.011876485 |
| CCDC50 | 5 | 0.011876485 |
| GDF15 | 5 | 0.011876485 |
| CAP2 | 5 | 0.011876485 |
| PRKAG2 | 5 | 0.011876485 |
| KCNAB2 | 5 | 0.011876485 |
| DNAJC14 | 5 | 0.011876485 |
| PROZ | 5 | 0.011876485 |
| NEUROG3 | 5 | 0.011876485 |
| GK5 | 5 | 0.011876485 |
| PNMA3 | 5 | 0.011876485 |
| ABHD2 | 5 | 0.011876485 |
| RETREG1 | 5 | 0.011876485 |
| CAPNS2 | 5 | 0.011876485 |
| OGG1 | 5 | 0.011876485 |
| NOP53 | 5 | 0.011876485 |
| CFAP300 | 5 | 0.011876485 |
| ENDOU | 5 | 0.011876485 |
| ASS1 | 5 | 0.011876485 |
| STYXL1 | 5 | 0.011876485 |
| PIGQ | 5 | 0.011876485 |
| VTI1A | 5 | 0.011876485 |
| IFI44L | 5 | 0.011876485 |
| CRYZL1 | 5 | 0.011876485 |
| MTCH1 | 5 | 0.011876485 |
| SECISBP2 | 5 | 0.011876485 |
| MTA3 | 5 | 0.011876485 |
| RDH5 | 5 | 0.011876485 |
| NOX1 | 5 | 0.011876485 |
| HSPD1 | 5 | 0.011876485 |
| STX8 | 5 | 0.011876485 |
| ABHD14A-ACY1 | 5 | 0.011876485 |
| NPFFR1 | 5 | 0.011876485 |
| SLC25A52 | 5 | 0.011876485 |
| TOM1L1 | 5 | 0.011876485 |
| APOBEC3C | 5 | 0.011876485 |
| DDX59 | 5 | 0.011876485 |
| SLC37A3 | 5 | 0.011876485 |
| CCDC28B | 5 | 0.011876485 |
| KRT78 | 5 | 0.011876485 |
| ADCYAP1 | 5 | 0.011876485 |
| UGCG | 5 | 0.011876485 |
| AK3 | 5 | 0.011876485 |
| DNALI1 | 5 | 0.011876485 |
| ING3 | 5 | 0.011876485 |
| UBFD1 | 5 | 0.011876485 |
| RAB3D | 5 | 0.011876485 |
| ENTPD6 | 5 | 0.011876485 |
| H3-5 | 5 | 0.011876485 |
| ABCD3 | 5 | 0.011876485 |
| SETD1B | 5 | 0.011876485 |
| LRR1 | 5 | 0.011876485 |
| FAM78A | 5 | 0.011876485 |
| NSUN6 | 5 | 0.011876485 |
| LGI2 | 5 | 0.011876485 |
| PTGDR2 | 5 | 0.011876485 |
| WTAP | 5 | 0.011876485 |
| SHBG | 5 | 0.011876485 |
| UGT1A3 | 5 | 0.011876485 |
| CTSB | 5 | 0.011876485 |
| CYP2E1 | 5 | 0.011876485 |
| OR6Y1 | 5 | 0.011876485 |
| TLX3 | 5 | 0.011876485 |
| ERLEC1 | 5 | 0.011876485 |
| S100PBP | 5 | 0.011876485 |
| ACSL3 | 5 | 0.011876485 |
| MRPS9 | 5 | 0.011876485 |
| POFUT1 | 5 | 0.011876485 |
| SP8 | 5 | 0.011876485 |
| UBA5 | 5 | 0.011876485 |
| FBXO3 | 5 | 0.011876485 |
| PID1 | 5 | 0.011876485 |
| FAM181B | 5 | 0.011876485 |
| INTS9 | 5 | 0.011876485 |
| CCN5 | 5 | 0.011876485 |
| C16orf70 | 5 | 0.011876485 |
| SOCS3 | 5 | 0.011876485 |
| DUSP11 | 5 | 0.011876485 |
| FAM71D | 5 | 0.011876485 |
| RHBDL3 | 5 | 0.011876485 |
| SPAG11A | 5 | 0.011876485 |
| RIOX2 | 5 | 0.011876485 |
| RNFT2 | 5 | 0.011876485 |
| MMADHC | 5 | 0.011876485 |
| CLEC10A | 5 | 0.011876485 |
| ST6GALNAC6 | 5 | 0.011876485 |
| ZNF880 | 5 | 0.011876485 |
| CPOX | 5 | 0.011876485 |
| OR4D6 | 5 | 0.011876485 |
| LGALS8 | 5 | 0.011876485 |
| ZNF710 | 5 | 0.011876485 |
| SLC25A48 | 5 | 0.011876485 |
| MYLK4 | 5 | 0.011876485 |
| SOX21 | 5 | 0.011876485 |
| FFAR4 | 5 | 0.011876485 |
| SLC2A1 | 5 | 0.011876485 |
| CCDC63 | 5 | 0.011876485 |
| PDK1 | 5 | 0.011876485 |
| KIR3DL1 | 5 | 0.011876485 |
| HOXD11 | 5 | 0.011876485 |
| ZNF550 | 5 | 0.011876485 |
| TRMT10B | 5 | 0.011876485 |
| TAS2R8 | 5 | 0.011876485 |
| ARR3 | 5 | 0.011876485 |
| RRP15 | 5 | 0.011876485 |
| ZNF222 | 5 | 0.011876485 |
| C5AR1 | 5 | 0.011876485 |
| EEF1A1 | 5 | 0.011876485 |
| OR52E8 | 5 | 0.011876485 |
| DTX2 | 5 | 0.011876485 |
| PHETA1 | 5 | 0.011876485 |
| ADGRG1 | 5 | 0.011876485 |
| SPDEF | 5 | 0.011876485 |
| GALNT12 | 5 | 0.011876485 |
| YY2 | 5 | 0.011876485 |
| TEX9 | 5 | 0.011876485 |
| PRTN3 | 5 | 0.011876485 |
| SLC25A43 | 5 | 0.011876485 |
| UBE2L3 | 5 | 0.011876485 |
| F2RL2 | 5 | 0.011876485 |
| GLS2 | 5 | 0.011876485 |
| SELENON | 5 | 0.011876485 |
| RUNX3 | 5 | 0.011876485 |
| PCYT1A | 5 | 0.011876485 |
| TSEN2 | 5 | 0.011876485 |
| SLC35B1 | 5 | 0.011876485 |
| BSND | 5 | 0.011876485 |
| UGT1A8 | 5 | 0.011876485 |
| KRT81 | 5 | 0.011876485 |
| OR2A4 | 5 | 0.011876485 |
| KNCN | 5 | 0.011876485 |
| PGLYRP1 | 5 | 0.011876485 |
| KLF13 | 5 | 0.011876485 |
| KYNU | 5 | 0.011876485 |
| RMND5B | 5 | 0.011876485 |
| B3GNT9 | 5 | 0.011876485 |
| NBL1 | 5 | 0.011876485 |
| GTF2B | 5 | 0.011876485 |
| DPH5 | 5 | 0.011876485 |
| AC244197.3 | 5 | 0.011876485 |
| MTMR14 | 5 | 0.011876485 |
| HPN | 5 | 0.011876485 |
| ERO1A | 5 | 0.011876485 |
| NAGLU | 5 | 0.011876485 |
| DCAF15 | 5 | 0.011876485 |
| SH2D5 | 5 | 0.011876485 |
| DPCD | 5 | 0.011876485 |
| ZNF695 | 5 | 0.011876485 |
| HAS3 | 5 | 0.011876485 |
| SAMD11 | 5 | 0.011876485 |
| CD6 | 5 | 0.011876485 |
| C1D | 5 | 0.011876485 |
| DDX19B | 5 | 0.011876485 |
| SIAH2 | 5 | 0.011876485 |
| RHCG | 5 | 0.011876485 |
| SRF | 5 | 0.011876485 |
| ACKR4 | 5 | 0.011876485 |
| HOXC4 | 5 | 0.011876485 |
| CRYM | 5 | 0.011876485 |
| TNFSF11 | 5 | 0.011876485 |
| KRTAP6-3 | 5 | 0.011876485 |
| NSUN5 | 5 | 0.011876485 |
| SLC39A4 | 5 | 0.011876485 |
| HRH4 | 5 | 0.011876485 |
| MAPK9 | 5 | 0.011876485 |
| PLA2G4C | 5 | 0.011876485 |
| IL24 | 5 | 0.011876485 |
| CNP | 5 | 0.011876485 |
| TMEM192 | 5 | 0.011876485 |
| IFNA4 | 5 | 0.011876485 |
| TMEM217 | 5 | 0.011876485 |
| TSPAN17 | 5 | 0.011876485 |
| OR1S2 | 5 | 0.011876485 |
| TAF1A | 5 | 0.011876485 |
| TSLP | 5 | 0.011876485 |
| BICDL2 | 5 | 0.011876485 |
| CCDC58 | 5 | 0.011876485 |
| DEK | 5 | 0.011876485 |
| NAP1L1 | 5 | 0.011876485 |
| MIIP | 5 | 0.011876485 |
| ADGRF2 | 5 | 0.011876485 |
| KLF4 | 5 | 0.011876485 |
| GPX6 | 5 | 0.011876485 |
| MCTS1 | 5 | 0.011876485 |
| PNN | 5 | 0.011876485 |
| GORAB | 5 | 0.011876485 |
| RGS18 | 5 | 0.011876485 |
| TOR1B | 5 | 0.011876485 |
| RNF113A | 5 | 0.011876485 |
| SELPLG | 5 | 0.011876485 |
| RBM34 | 5 | 0.011876485 |
| NPEPPS | 5 | 0.011876485 |
| FAM187B | 5 | 0.011876485 |
| ADPGK | 5 | 0.011876485 |
| UBR7 | 5 | 0.011876485 |
| HEYL | 5 | 0.011876485 |
| BPNT2 | 5 | 0.011876485 |
| SUZ12 | 5 | 0.011876485 |
| ECM1 | 5 | 0.011876485 |
| LPAR2 | 5 | 0.011876485 |
| OR1F1 | 5 | 0.011876485 |
| FAM209B | 5 | 0.011876485 |
| BAAT | 5 | 0.011876485 |
| CAMK2A | 5 | 0.011876485 |
| C1orf189 | 5 | 0.011876485 |
| TOR1AIP1 | 5 | 0.011876485 |
| AKAP5 | 5 | 0.011876485 |
| SQLE | 5 | 0.011876485 |
| CRTC3 | 5 | 0.011876485 |
| HARS2 | 5 | 0.011876485 |
| RFC5 | 5 | 0.011876485 |
| C2CD2 | 5 | 0.011876485 |
| ACOT2 | 5 | 0.011876485 |
| DDIT4L | 5 | 0.011876485 |
| PLK2 | 5 | 0.011876485 |
| MNAT1 | 5 | 0.011876485 |
| PHETA2 | 5 | 0.011876485 |
| SEPTIN4 | 5 | 0.011876485 |
| RGS2 | 5 | 0.011876485 |
| KLF15 | 5 | 0.011876485 |
| ZNF584 | 5 | 0.011876485 |
| DSN1 | 5 | 0.011876485 |
| HTR6 | 5 | 0.011876485 |
| TMEM255A | 5 | 0.011876485 |
| NARF | 5 | 0.011876485 |
| ZNF8 | 5 | 0.011876485 |
| PRKCA | 5 | 0.011876485 |
| JSRP1 | 5 | 0.011876485 |
| PIF1 | 5 | 0.011876485 |
| IKZF5 | 5 | 0.011876485 |
| MS4A8 | 5 | 0.011876485 |
| LAG3 | 5 | 0.011876485 |
| STRC | 5 | 0.011876485 |
| FOXF2 | 5 | 0.011876485 |
| EXOSC9 | 5 | 0.011876485 |
| HLA-DQA2 | 5 | 0.011876485 |
| FBXW12 | 5 | 0.011876485 |
| ARID3C | 5 | 0.011876485 |
| LDHB | 5 | 0.011876485 |
| CAPZA2 | 5 | 0.011876485 |
| CYP3A43 | 5 | 0.011876485 |
| CAGE1 | 5 | 0.011876485 |
| CEPT1 | 5 | 0.011876485 |
| SLC22A1 | 5 | 0.011876485 |
| ECI2 | 5 | 0.011876485 |
| SHQ1 | 5 | 0.011876485 |
| ASCL4 | 5 | 0.011876485 |
| PRKAR1B | 5 | 0.011876485 |
| SH3GL1 | 5 | 0.011876485 |
| UNG | 5 | 0.011876485 |
| MFSD14A | 5 | 0.011876485 |
| PAQR4 | 5 | 0.011876485 |
| ADGB | 5 | 0.011876485 |
| SLC12A8 | 5 | 0.011876485 |
| RGL1 | 5 | 0.011876485 |
| VSTM2L | 5 | 0.011876485 |
| RSRC2 | 5 | 0.011876485 |
| TPM3 | 5 | 0.011876485 |
| GLTPD2 | 5 | 0.011876485 |
| SAMD12 | 5 | 0.011876485 |
| SELENOM | 5 | 0.011876485 |
| CADM4 | 5 | 0.011876485 |
| KLF14 | 5 | 0.011876485 |
| REEP5 | 5 | 0.011876485 |
| TCTE1 | 5 | 0.011876485 |
| GPAT4 | 5 | 0.011876485 |
| SYT6 | 5 | 0.011876485 |
| CDK3 | 5 | 0.011876485 |
| HNRNPC | 5 | 0.011876485 |
| STX12 | 5 | 0.011876485 |
| G6PC3 | 5 | 0.011876485 |
| SKIL | 5 | 0.011876485 |
| TMEM62 | 5 | 0.011876485 |
| TADA2A | 5 | 0.011876485 |
| PNLIPRP2 | 5 | 0.011876485 |
| B3GALT1 | 5 | 0.011876485 |
| HNMT | 5 | 0.011876485 |
| PANK3 | 5 | 0.011876485 |
| ERI3 | 5 | 0.011876485 |
| C11orf53 | 5 | 0.011876485 |
| MRPL54 | 5 | 0.011876485 |
| ENOSF1 | 5 | 0.011876485 |
| OR2T3 | 5 | 0.011876485 |
| RFTN2 | 5 | 0.011876485 |
| OR8D4 | 5 | 0.011876485 |
| SCARF1 | 5 | 0.011876485 |
| ZNF48 | 5 | 0.011876485 |
| TAS2R7 | 5 | 0.011876485 |
| ANXA1 | 5 | 0.011876485 |
| PGLYRP3 | 5 | 0.011876485 |
| CPB1 | 5 | 0.011876485 |
| GTPBP1 | 5 | 0.011876485 |
| TYRP1 | 5 | 0.011876485 |
| MRPL28 | 5 | 0.011876485 |
| FOLR1 | 5 | 0.011876485 |
| SCN3B | 5 | 0.011876485 |
| SLC7A8 | 5 | 0.011876485 |
| CAPN14 | 5 | 0.011876485 |
| HENMT1 | 5 | 0.011876485 |
| NECTIN2 | 5 | 0.011876485 |
| SYF2 | 5 | 0.011876485 |
| USP22 | 5 | 0.011876485 |
| HTR4 | 5 | 0.011876485 |
| ASPA | 5 | 0.011876485 |
| MPZL1 | 5 | 0.011876485 |
| CSTL1 | 5 | 0.011876485 |
| TKT | 5 | 0.011876485 |
| LYG1 | 5 | 0.011876485 |
| KCTD14 | 5 | 0.011876485 |
| GLP2R | 5 | 0.011876485 |
| SPNS3 | 5 | 0.011876485 |
| CCR2 | 5 | 0.011876485 |
| HOXB2 | 5 | 0.011876485 |
| OSTM1 | 5 | 0.011876485 |
| PHKG2 | 5 | 0.011876485 |
| MAPK14 | 5 | 0.011876485 |
| CFAP299 | 5 | 0.011876485 |
| OR6S1 | 5 | 0.011876485 |
| CRYBB1 | 5 | 0.011876485 |
| ART3 | 5 | 0.011876485 |
| RBM17 | 5 | 0.011876485 |
| STK35 | 5 | 0.011876485 |
| PTPN22 | 5 | 0.011876485 |
| ACHE | 5 | 0.011876485 |
| MRPL44 | 5 | 0.011876485 |
| ISY1-RAB43 | 5 | 0.011876485 |
| CASP10 | 5 | 0.011876485 |
| SSX3 | 5 | 0.011876485 |
| LSM1 | 5 | 0.011876485 |
| MACROH2A1 | 5 | 0.011876485 |
| CALB2 | 5 | 0.011876485 |
| CDK9 | 5 | 0.011876485 |
| GCKR | 5 | 0.011876485 |
| MTHFR | 5 | 0.011876485 |
| KLHDC7B | 5 | 0.011876485 |
| FUT7 | 5 | 0.011876485 |
| EXOSC8 | 5 | 0.011876485 |
| NDUFAF1 | 5 | 0.011876485 |
| MPG | 5 | 0.011876485 |
| TESK2 | 5 | 0.011876485 |
| TGFB1 | 5 | 0.011876485 |
| NUP85 | 5 | 0.011876485 |
| TERT | 5 | 0.011876485 |
| PI16 | 5 | 0.011876485 |
| C7orf57 | 5 | 0.011876485 |
| CALCRL | 5 | 0.011876485 |
| FOSL1 | 5 | 0.011876485 |
| SURF4 | 5 | 0.011876485 |
| RABEP2 | 5 | 0.011876485 |
| OR1G1 | 5 | 0.011876485 |
| SLC52A2 | 5 | 0.011876485 |
| TARS1 | 5 | 0.011876485 |
| RNFT1 | 5 | 0.011876485 |
| NAF1 | 5 | 0.011876485 |
| SEPTIN5 | 5 | 0.011876485 |
| ELMO2 | 5 | 0.011876485 |
| ZNF662 | 5 | 0.011876485 |
| RNMT | 5 | 0.011876485 |
| UGT1A9 | 5 | 0.011876485 |
| TBL3 | 5 | 0.011876485 |
| RORA | 5 | 0.011876485 |
| STX3 | 5 | 0.011876485 |
| OR7G1 | 5 | 0.011876485 |
| CEBPE | 5 | 0.011876485 |
| CRLF3 | 5 | 0.011876485 |
| VTCN1 | 5 | 0.011876485 |
| HYAL4 | 5 | 0.011876485 |
| TEFM | 5 | 0.011876485 |
| EAF2 | 5 | 0.011876485 |
| SLC41A2 | 5 | 0.011876485 |
| EXOC7 | 5 | 0.011876485 |
| GTPBP2 | 5 | 0.011876485 |
| MAPK15 | 5 | 0.011876485 |
| NCF4 | 5 | 0.011876485 |
| AGPAT3 | 5 | 0.011876485 |
| DNAJB12 | 5 | 0.011876485 |
| CDK7 | 5 | 0.011876485 |
| PRPSAP1 | 5 | 0.011876485 |
| GNB5 | 5 | 0.011876485 |
| CYB5RL | 5 | 0.011876485 |
| DAPK3 | 5 | 0.011876485 |
| PHF19 | 5 | 0.011876485 |
| PARP12 | 5 | 0.011876485 |
| SNX10 | 5 | 0.011876485 |
| OR51A2 | 5 | 0.011876485 |
| NFYC | 5 | 0.011876485 |
| PLIN3 | 5 | 0.011876485 |
| TSTD2 | 5 | 0.011876485 |
| C1GALT1 | 5 | 0.011876485 |
| CENPB | 5 | 0.011876485 |
| AP1B1 | 5 | 0.011876485 |
| SGCB | 5 | 0.011876485 |
| NEU2 | 5 | 0.011876485 |
| PPP2R5C | 5 | 0.011876485 |
| RABEP1 | 5 | 0.011876485 |
| IL17REL | 5 | 0.011876485 |
| PNO1 | 5 | 0.011876485 |
| GGT7 | 5 | 0.011876485 |
| LCAT | 5 | 0.011876485 |
| CTDSPL | 5 | 0.011876485 |
| MAGEH1 | 5 | 0.011876485 |
| HEXD | 5 | 0.011876485 |
| OR6X1 | 5 | 0.011876485 |
| RETSAT | 5 | 0.011876485 |
| TIGIT | 5 | 0.011876485 |
| PBDC1 | 5 | 0.011876485 |
| VIPAS39 | 5 | 0.011876485 |
| ACOX2 | 5 | 0.011876485 |
| DCLRE1B | 5 | 0.011876485 |
| MARCKS | 5 | 0.011876485 |
| TAZ | 5 | 0.011876485 |
| CAPZA3 | 5 | 0.011876485 |
| SCGN | 5 | 0.011876485 |
| AGA | 5 | 0.011876485 |
| ODF1 | 5 | 0.011876485 |
| ADAL | 5 | 0.011876485 |
| RRN3 | 5 | 0.011876485 |
| OR2T4 | 5 | 0.011876485 |
| TMIGD1 | 5 | 0.011876485 |
| GET1 | 5 | 0.011876485 |
| BMX | 5 | 0.011876485 |
| OR4K13 | 5 | 0.011876485 |
| TES | 5 | 0.011876485 |
| SLC9B2 | 5 | 0.011876485 |
| CLNS1A | 5 | 0.011876485 |
| KCNK5 | 5 | 0.011876485 |
| LILRA2 | 5 | 0.011876485 |
| ST8SIA2 | 5 | 0.011876485 |
| SOX15 | 5 | 0.011876485 |
| TP53I11 | 5 | 0.011876485 |
| ANKRD45 | 5 | 0.011876485 |
| ZC3H10 | 5 | 0.011876485 |
| PRSS23 | 5 | 0.011876485 |
| MAGEB3 | 5 | 0.011876485 |
| MRGPRX4 | 5 | 0.011876485 |
| OR13C9 | 5 | 0.011876485 |
| AGFG2 | 5 | 0.011876485 |
| LACTB2 | 5 | 0.011876485 |
| GK | 5 | 0.011876485 |
| PRIM2 | 5 | 0.011876485 |
| PDIA6 | 5 | 0.011876485 |
| ACTL8 | 5 | 0.011876485 |
| IDS | 5 | 0.011876485 |
| PI4K2A | 5 | 0.011876485 |
| CLDN16 | 5 | 0.011876485 |
| GALC | 5 | 0.011876485 |
| TNMD | 5 | 0.011876485 |
| SLC25A34 | 5 | 0.011876485 |
| HAT1 | 5 | 0.011876485 |
| SESN1 | 5 | 0.011876485 |
| SLC35F5 | 5 | 0.011876485 |
| CD40 | 5 | 0.011876485 |
| CLDND1 | 5 | 0.011876485 |
| RNPS1 | 5 | 0.011876485 |
| MSX2 | 5 | 0.011876485 |
| CRBN | 5 | 0.011876485 |
| ATP6V1A | 5 | 0.011876485 |
| SENP3 | 5 | 0.011876485 |
| SLC7A5 | 5 | 0.011876485 |
| VASP | 5 | 0.011876485 |
| VGF | 5 | 0.011876485 |
| ZFYVE19 | 5 | 0.011876485 |
| ASB16 | 5 | 0.011876485 |
| SERINC4 | 5 | 0.011876485 |
| GDF11 | 5 | 0.011876485 |
| TNFRSF1A | 5 | 0.011876485 |
| TMBIM1 | 5 | 0.011876485 |
| CERS4 | 5 | 0.011876485 |
| AC005324.2 | 5 | 0.011876485 |
| PPP1R2 | 5 | 0.011876485 |
| SPATS1 | 5 | 0.011876485 |
| MOGAT3 | 5 | 0.011876485 |
| BAG4 | 5 | 0.011876485 |
| PSKH1 | 5 | 0.011876485 |
| OR2K2 | 5 | 0.011876485 |
| SHMT2 | 5 | 0.011876485 |
| H2BC6 | 5 | 0.011876485 |
| GPR143 | 5 | 0.011876485 |
| ZUP1 | 5 | 0.011876485 |
| DENND2D | 5 | 0.011876485 |
| ANXA10 | 5 | 0.011876485 |
| CASQ1 | 5 | 0.011876485 |
| TM6SF2 | 5 | 0.011876485 |
| TP53INP1 | 5 | 0.011876485 |
| SUV39H1 | 5 | 0.011876485 |
| SSB | 5 | 0.011876485 |
| LRRC45 | 5 | 0.011876485 |
| HP1BP3 | 5 | 0.011876485 |
| CRKL | 5 | 0.011876485 |
| OR5P2 | 5 | 0.011876485 |
| BPIFC | 5 | 0.011876485 |
| ILRUN | 5 | 0.011876485 |
| DERA | 5 | 0.011876485 |
| ZNF681 | 5 | 0.011876485 |
| SLC25A37 | 5 | 0.011876485 |
| SLC52A1 | 5 | 0.011876485 |
| ATP6V1C2 | 5 | 0.011876485 |
| PTDSS2 | 5 | 0.011876485 |
| PPP6C | 5 | 0.011876485 |
| GTF3C5 | 5 | 0.011876485 |
| ZBTB32 | 5 | 0.011876485 |
| HYAL2 | 5 | 0.011876485 |
| FGFR4 | 5 | 0.011876485 |
| EPS8 | 5 | 0.011876485 |
| MRPL38 | 5 | 0.011876485 |
| ZNF155 | 5 | 0.011876485 |
| NT5E | 5 | 0.011876485 |
| B3GNT4 | 5 | 0.011876485 |
| OR52R1 | 5 | 0.011876485 |
| MRPS27 | 5 | 0.011876485 |
| IRF2BPL | 5 | 0.011876485 |
| RBFOX2 | 5 | 0.011876485 |
| ARSK | 5 | 0.011876485 |
| POLB | 5 | 0.011876485 |
| PHF10 | 5 | 0.011876485 |
| HDAC1 | 5 | 0.011876485 |
| C1GALT1C1 | 5 | 0.011876485 |
| DCAF17 | 5 | 0.011876485 |
| MON1A | 5 | 0.011876485 |
| GSTM5 | 5 | 0.011876485 |
| EXOSC5 | 5 | 0.011876485 |
| ZNF697 | 5 | 0.011876485 |
| ARRDC3 | 5 | 0.011876485 |
| SDC3 | 5 | 0.011876485 |
| ITPKA | 5 | 0.011876485 |
| CERS5 | 5 | 0.011876485 |
| CYTL1 | 5 | 0.011876485 |
| FAM133B | 5 | 0.011876485 |
| TMEFF1 | 5 | 0.011876485 |
| BHMT | 5 | 0.011876485 |
| AREL1 | 5 | 0.011876485 |
| OR8S1 | 5 | 0.011876485 |
| SFRP2 | 5 | 0.011876485 |
| LDHC | 5 | 0.011876485 |
| PRPF4 | 5 | 0.011876485 |
| HMGN5 | 5 | 0.011876485 |
| DNAJB9 | 5 | 0.011876485 |
| OR5K4 | 5 | 0.011876485 |
| FAM111A | 5 | 0.011876485 |
| ODF3 | 5 | 0.011876485 |
| AC011455.2 | 5 | 0.011876485 |
| CA13 | 5 | 0.011876485 |
| DMWD | 5 | 0.011876485 |
| PLP1 | 5 | 0.011876485 |
| MTF1 | 5 | 0.011876485 |
| UBE2K | 5 | 0.011876485 |
| PRMT5 | 5 | 0.011876485 |
| NUP50 | 5 | 0.011876485 |
| GPR75 | 5 | 0.011876485 |
| STAC3 | 5 | 0.011876485 |
| HSPA13 | 5 | 0.011876485 |
| CTPS2 | 5 | 0.011876485 |
| SYT17 | 5 | 0.011876485 |
| LCLAT1 | 5 | 0.011876485 |
| SOX3 | 5 | 0.011876485 |
| GAS8 | 5 | 0.011876485 |
| PPP2R1B | 5 | 0.011876485 |
| PHLDB3 | 5 | 0.011876485 |
| TAS2R50 | 5 | 0.011876485 |
| C16orf46 | 5 | 0.011876485 |
| ZNF268 | 5 | 0.011876485 |
| HMOX1 | 5 | 0.011876485 |
| SGCA | 5 | 0.011876485 |
| SERPINA9 | 5 | 0.011876485 |
| CNOT2 | 5 | 0.011876485 |
| B4GALNT3 | 5 | 0.011876485 |
| FCRL6 | 5 | 0.011876485 |
| GPR42 | 5 | 0.011876485 |
| ALDH3B1 | 5 | 0.011876485 |
| IPMK | 5 | 0.011876485 |
| ZNF506 | 5 | 0.011876485 |
| IMP4 | 5 | 0.011876485 |
| FPR3 | 5 | 0.011876485 |
| BPHL | 5 | 0.011876485 |
| C1orf122 | 5 | 0.011876485 |
| KCTD21 | 5 | 0.011876485 |
| GRAP2 | 5 | 0.011876485 |
| FAM168B | 5 | 0.011876485 |
| PLSCR3 | 5 | 0.011876485 |
| AVIL | 5 | 0.011876485 |
| HAUS1 | 5 | 0.011876485 |
| ARID3B | 5 | 0.011876485 |
| PXK | 5 | 0.011876485 |
| NAA80 | 5 | 0.011876485 |
| SLC38A7 | 5 | 0.011876485 |
| ADM | 5 | 0.011876485 |
| METTL15 | 5 | 0.011876485 |
| IFT57 | 5 | 0.011876485 |
| P2RX3 | 5 | 0.011876485 |
| NDUFV1 | 5 | 0.011876485 |
| PPP1R15A | 5 | 0.011876485 |
| BIRC2 | 5 | 0.011876485 |
| SSR1 | 5 | 0.011876485 |
| OR8D2 | 5 | 0.011876485 |
| DLX2 | 5 | 0.011876485 |
| PRR30 | 5 | 0.011876485 |
| GIMAP4 | 5 | 0.011876485 |
| DDX10 | 5 | 0.011876485 |
| CD72 | 5 | 0.011876485 |
| CCDC81 | 5 | 0.011876485 |
| SYTL2 | 5 | 0.011876485 |
| CXADR | 5 | 0.011876485 |
| OR5T3 | 5 | 0.011876485 |
| IFI44 | 5 | 0.011876485 |
| TRMT5 | 5 | 0.011876485 |
| ST3GAL1 | 5 | 0.011876485 |
| SPESP1 | 5 | 0.011876485 |
| CCNB1IP1 | 5 | 0.011876485 |
| CIBAR2 | 5 | 0.011876485 |
| OR3A3 | 5 | 0.011876485 |
| C1orf159 | 5 | 0.011876485 |
| SLC10A6 | 5 | 0.011876485 |
| CRYL1 | 5 | 0.011876485 |
| PNP | 5 | 0.011876485 |
| HSPBAP1 | 5 | 0.011876485 |
| TIMM22 | 5 | 0.011876485 |
| C2orf80 | 5 | 0.011876485 |
| POLR1G | 5 | 0.011876485 |
| DECR2 | 5 | 0.011876485 |
| TRNT1 | 5 | 0.011876485 |
| DENND2A | 5 | 0.011876485 |
| CCN3 | 5 | 0.011876485 |
| ANKRD36B | 5 | 0.011876485 |
| ABRAXAS1 | 5 | 0.011876485 |
| WDR92 | 5 | 0.011876485 |
| APTX | 5 | 0.011876485 |
| UBA3 | 5 | 0.011876485 |
| MBP | 5 | 0.011876485 |
| F11R | 5 | 0.011876485 |
| C1orf116 | 5 | 0.011876485 |
| RNF125 | 5 | 0.011876485 |
| FBXO47 | 5 | 0.011876485 |
| RPL28 | 5 | 0.011876485 |
| MANEA | 5 | 0.011876485 |
| GOPC | 5 | 0.011876485 |
| STAP2 | 5 | 0.011876485 |
| SLC35A5 | 5 | 0.011876485 |
| PNRC1 | 5 | 0.011876485 |
| FOXH1 | 5 | 0.011876485 |
| CCT5 | 5 | 0.011876485 |
| UQCRC1 | 5 | 0.011876485 |
| CHAMP1 | 5 | 0.011876485 |
| NGEF | 5 | 0.011876485 |
| NCLN | 5 | 0.011876485 |
| SEC13 | 5 | 0.011876485 |
| CHCHD5 | 5 | 0.011876485 |
| PRPF19 | 5 | 0.011876485 |
| TM4SF19 | 5 | 0.011876485 |
| UTP18 | 5 | 0.011876485 |
| POU5F1 | 5 | 0.011876485 |
| CAMLG | 5 | 0.011876485 |
| PROCR | 5 | 0.011876485 |
| RHD | 5 | 0.011876485 |
| CFAP221 | 5 | 0.011876485 |
| EFEMP2 | 5 | 0.011876485 |
| ZNF182 | 5 | 0.011876485 |
| BIN2 | 5 | 0.011876485 |
| TXNL1 | 5 | 0.011876485 |
| CGB1 | 5 | 0.011876485 |
| MAGEB6 | 5 | 0.011876485 |
| IPCEF1 | 5 | 0.011876485 |
| MYOZ3 | 5 | 0.011876485 |
| KCTD20 | 5 | 0.011876485 |
| POLR1E | 5 | 0.011876485 |
| FNDC8 | 5 | 0.011876485 |
| ZNF410 | 5 | 0.011876485 |
| PLSCR1 | 5 | 0.011876485 |
| SPN | 5 | 0.011876485 |
| E2F4 | 5 | 0.011876485 |
| MLEC | 5 | 0.011876485 |
| PPP1R42 | 5 | 0.011876485 |
| SMYD4 | 5 | 0.011876485 |
| ASPDH | 5 | 0.011876485 |
| CHST13 | 5 | 0.011876485 |
| REEP2 | 5 | 0.011876485 |
| CAMK1 | 5 | 0.011876485 |
| SLC52A3 | 5 | 0.011876485 |
| TOLLIP | 5 | 0.011876485 |
| LRRC10 | 5 | 0.011876485 |
| PMEL | 5 | 0.011876485 |
| MIP | 5 | 0.011876485 |
| PRPS1L1 | 5 | 0.011876485 |
| TEX45 | 5 | 0.011876485 |
| ENPP6 | 5 | 0.011876485 |
| HLA-DPB1 | 5 | 0.011876485 |
| NAPSA | 5 | 0.011876485 |
| SLC39A8 | 5 | 0.011876485 |
| POC1A | 5 | 0.011876485 |
| MCUB | 5 | 0.011876485 |
| GZMH | 5 | 0.011876485 |
| HNRNPR | 5 | 0.011876485 |
| ZNF707 | 5 | 0.011876485 |
| SIAH3 | 5 | 0.011876485 |
| OR52B2 | 5 | 0.011876485 |
| MYOC | 5 | 0.011876485 |
| DDX21 | 5 | 0.011876485 |
| CXXC5 | 5 | 0.011876485 |
| DMAP1 | 5 | 0.011876485 |
| GPD1L | 5 | 0.011876485 |
| SYCE1 | 5 | 0.011876485 |
| PRAMEF19 | 5 | 0.011876485 |
| CCDC124 | 5 | 0.011876485 |
| RPIA | 5 | 0.011876485 |
| ZBTB2 | 5 | 0.011876485 |
| RAD51C | 5 | 0.011876485 |
| TMCO4 | 5 | 0.011876485 |
| DUSP9 | 5 | 0.011876485 |
| SPRY4 | 5 | 0.011876485 |
| IGF2BP2 | 5 | 0.011876485 |
| ETNK1 | 5 | 0.011876485 |
| TRIT1 | 5 | 0.011876485 |
| DERL2 | 5 | 0.011876485 |
| U2AF2 | 5 | 0.011876485 |
| ZNF565 | 5 | 0.011876485 |
| DNAAF2 | 5 | 0.011876485 |
| ZNF75A | 5 | 0.011876485 |
| PTN | 5 | 0.011876485 |
| MLPH | 5 | 0.011876485 |
| MAP1LC3C | 5 | 0.011876485 |
| TMEM184C | 5 | 0.011876485 |
| CLEC2B | 5 | 0.011876485 |
| LRCH4 | 5 | 0.011876485 |
| SLC20A1 | 5 | 0.011876485 |
| NARS1 | 5 | 0.011876485 |
| PNOC | 5 | 0.011876485 |
| PORCN | 5 | 0.011876485 |
| ATP6V1E2 | 5 | 0.011876485 |
| MTRF1L | 5 | 0.011876485 |
| PEBP4 | 5 | 0.011876485 |
| OLA1 | 5 | 0.011876485 |
| ZSCAN25 | 5 | 0.011876485 |
| ONECUT3 | 5 | 0.011876485 |
| MAP1LC3B2 | 5 | 0.011876485 |
| KAT8 | 5 | 0.011876485 |
| MPPE1 | 5 | 0.011876485 |
| OR2F1 | 5 | 0.011876485 |
| XYLB | 5 | 0.011876485 |
| GPR152 | 5 | 0.011876485 |
| WNT10A | 5 | 0.011876485 |
| ATP5F1C | 5 | 0.011876485 |
| NCKIPSD | 5 | 0.011876485 |
| EHD1 | 5 | 0.011876485 |
| OTX1 | 5 | 0.011876485 |
| ACP4 | 5 | 0.011876485 |
| PCMTD2 | 5 | 0.011876485 |
| OR7A5 | 5 | 0.011876485 |
| OR13C5 | 5 | 0.011876485 |
| LIPJ | 5 | 0.011876485 |
| STARD3 | 5 | 0.011876485 |
| HABP2 | 5 | 0.011876485 |
| PNPLA2 | 5 | 0.011876485 |
| DAGLB | 5 | 0.011876485 |
| EMD | 5 | 0.011876485 |
| TMEM147 | 5 | 0.011876485 |
| RGS7BP | 5 | 0.011876485 |
| CYP39A1 | 5 | 0.011876485 |
| FOXD3 | 5 | 0.011876485 |
| NPVF | 5 | 0.011876485 |
| KRT7 | 5 | 0.011876485 |
| CSTF2 | 5 | 0.011876485 |
| HMGXB4 | 5 | 0.011876485 |
| BTG4 | 5 | 0.011876485 |
| RRM2 | 5 | 0.011876485 |
| SYT7 | 5 | 0.011876485 |
| CNOT6L | 5 | 0.011876485 |
| CREB3L4 | 5 | 0.011876485 |
| SPATA22 | 5 | 0.011876485 |
| UGT2B7 | 5 | 0.011876485 |
| OR1M1 | 5 | 0.011876485 |
| CYP4F11 | 5 | 0.011876485 |
| NAPRT | 5 | 0.011876485 |
| LRRC27 | 5 | 0.011876485 |
| STOML1 | 5 | 0.011876485 |
| PABPC4L | 5 | 0.011876485 |
| LEXM | 5 | 0.011876485 |
| ZNF215 | 5 | 0.011876485 |
| P2RY6 | 5 | 0.011876485 |
| PRAMEF4 | 5 | 0.011876485 |
| RBMS2 | 5 | 0.011876485 |
| CCDC130 | 5 | 0.011876485 |
| VEZF1 | 5 | 0.011876485 |
| TUFT1 | 5 | 0.011876485 |
| LAS1L | 5 | 0.011876485 |
| HOXA7 | 5 | 0.011876485 |
| UTS2R | 5 | 0.011876485 |
| KPTN | 5 | 0.011876485 |
| MAPK4 | 5 | 0.011876485 |
| ECD | 5 | 0.011876485 |
| MYLK2 | 5 | 0.011876485 |
| PELI1 | 5 | 0.011876485 |
| RBM4 | 5 | 0.011876485 |
| SPIB | 5 | 0.011876485 |
| GSTZ1 | 5 | 0.011876485 |
| GLUD1 | 5 | 0.011876485 |
| HOXD8 | 5 | 0.011876485 |
| NOXO1 | 5 | 0.011876485 |
| GXYLT2 | 5 | 0.011876485 |
| OTOG | 5 | 0.011876485 |
| LUC7L2 | 5 | 0.011876485 |
| BUB1B | 5 | 0.011876485 |
| PCK2 | 5 | 0.011876485 |
| TNFRSF13B | 5 | 0.011876485 |
| BLMH | 5 | 0.011876485 |
| BRAT1 | 5 | 0.011876485 |
| MC4R | 5 | 0.011876485 |
| NRSN2 | 5 | 0.011876485 |
| LACC1 | 5 | 0.011876485 |
| PNPLA1 | 5 | 0.011876485 |
| MEX3A | 5 | 0.011876485 |
| ZNF655 | 5 | 0.011876485 |
| DUS3L | 5 | 0.011876485 |
| DDX56 | 5 | 0.011876485 |
| PITHD1 | 5 | 0.011876485 |
| ABHD12 | 5 | 0.011876485 |
| OIT3 | 5 | 0.011876485 |
| SULT4A1 | 5 | 0.011876485 |
| IQCG | 5 | 0.011876485 |
| NOL12 | 5 | 0.011876485 |
| FBXW8 | 5 | 0.011876485 |
| METTL1 | 5 | 0.011876485 |
| NOTUM | 5 | 0.011876485 |
| TRIP13 | 5 | 0.011876485 |
| GEMIN8 | 5 | 0.011876485 |
| GADL1 | 5 | 0.011876485 |
| MAPKAPK2 | 5 | 0.011876485 |
| CFAP97 | 5 | 0.011876485 |
| TMEM120A | 5 | 0.011876485 |
| KCNIP2 | 5 | 0.011876485 |
| FBXO8 | 5 | 0.011876485 |
| GPR132 | 5 | 0.011876485 |
| C3orf38 | 5 | 0.011876485 |
| OLFM3 | 5 | 0.011876485 |
| KLHDC3 | 5 | 0.011876485 |
| KCNK7 | 5 | 0.011876485 |
| ARRDC1 | 5 | 0.011876485 |
| KRT35 | 5 | 0.011876485 |
| HSF5 | 5 | 0.011876485 |
| CACNG8 | 5 | 0.011876485 |
| LDHAL6A | 5 | 0.011876485 |
| NOL7 | 5 | 0.011876485 |
| SLC29A2 | 5 | 0.011876485 |
| RPP40 | 5 | 0.011876485 |
| VSTM1 | 5 | 0.011876485 |
| PROSER2 | 5 | 0.011876485 |
| B4GALT2 | 5 | 0.011876485 |
| SGMS1 | 5 | 0.011876485 |
| NAPB | 5 | 0.011876485 |
| KIAA1328 | 5 | 0.011876485 |
| HPCA | 5 | 0.011876485 |
| SNX17 | 5 | 0.011876485 |
| BPIFA1 | 4 | 0.009501188 |
| CD200 | 4 | 0.009501188 |
| CFAP92 | 4 | 0.009501188 |
| PAX9 | 4 | 0.009501188 |
| PAGE2 | 4 | 0.009501188 |
| ABHD4 | 4 | 0.009501188 |
| HBP1 | 4 | 0.009501188 |
| RSPH14 | 4 | 0.009501188 |
| DNASE2 | 4 | 0.009501188 |
| TMEM225 | 4 | 0.009501188 |
| LRRC75B | 4 | 0.009501188 |
| SLC25A35 | 4 | 0.009501188 |
| USF1 | 4 | 0.009501188 |
| SLC35G6 | 4 | 0.009501188 |
| TMEM266 | 4 | 0.009501188 |
| CBLL1 | 4 | 0.009501188 |
| PNKP | 4 | 0.009501188 |
| OS9 | 4 | 0.009501188 |
| GTF3C6 | 4 | 0.009501188 |
| THUMPD2 | 4 | 0.009501188 |
| GPSM3 | 4 | 0.009501188 |
| AIRE | 4 | 0.009501188 |
| FAM209A | 4 | 0.009501188 |
| PEX16 | 4 | 0.009501188 |
| ACADS | 4 | 0.009501188 |
| WBP11 | 4 | 0.009501188 |
| DDAH1 | 4 | 0.009501188 |
| KLRF1 | 4 | 0.009501188 |
| RHOQ | 4 | 0.009501188 |
| CCDC3 | 4 | 0.009501188 |
| MGST1 | 4 | 0.009501188 |
| OR1Q1 | 4 | 0.009501188 |
| TTC22 | 4 | 0.009501188 |
| ALG1 | 4 | 0.009501188 |
| CELF6 | 4 | 0.009501188 |
| EIF3I | 4 | 0.009501188 |
| PREPL | 4 | 0.009501188 |
| ALPG | 4 | 0.009501188 |
| DNASE1L3 | 4 | 0.009501188 |
| PGGT1B | 4 | 0.009501188 |
| C9orf64 | 4 | 0.009501188 |
| SQOR | 4 | 0.009501188 |
| CLEC4A | 4 | 0.009501188 |
| NRBF2 | 4 | 0.009501188 |
| AMFR | 4 | 0.009501188 |
| GALNT1 | 4 | 0.009501188 |
| OR4E2 | 4 | 0.009501188 |
| HBG2 | 4 | 0.009501188 |
| STYX | 4 | 0.009501188 |
| FECH | 4 | 0.009501188 |
| COASY | 4 | 0.009501188 |
| MPZ | 4 | 0.009501188 |
| SPATA18 | 4 | 0.009501188 |
| VIP | 4 | 0.009501188 |
| SMAD5 | 4 | 0.009501188 |
| RBM38 | 4 | 0.009501188 |
| LRRN2 | 4 | 0.009501188 |
| PCBP2 | 4 | 0.009501188 |
| MS4A5 | 4 | 0.009501188 |
| BCO1 | 4 | 0.009501188 |
| NUDT11 | 4 | 0.009501188 |
| OR2A25 | 4 | 0.009501188 |
| TMEM19 | 4 | 0.009501188 |
| FGF9 | 4 | 0.009501188 |
| ZNF207 | 4 | 0.009501188 |
| NOMO2 | 4 | 0.009501188 |
| PARM1 | 4 | 0.009501188 |
| MOB4 | 4 | 0.009501188 |
| DUSP8 | 4 | 0.009501188 |
| CSN1S1 | 4 | 0.009501188 |
| ARMCX3 | 4 | 0.009501188 |
| SEPTIN14 | 4 | 0.009501188 |
| TBXA2R | 4 | 0.009501188 |
| PSMA3 | 4 | 0.009501188 |
| NPY | 4 | 0.009501188 |
| RFWD3 | 4 | 0.009501188 |
| SNX11 | 4 | 0.009501188 |
| MINDY3 | 4 | 0.009501188 |
| FAM162B | 4 | 0.009501188 |
| HCAR2 | 4 | 0.009501188 |
| GGH | 4 | 0.009501188 |
| ARL14 | 4 | 0.009501188 |
| GDF7 | 4 | 0.009501188 |
| EGLN1 | 4 | 0.009501188 |
| H1-1 | 4 | 0.009501188 |
| RNF19B | 4 | 0.009501188 |
| IGHV5-51 | 4 | 0.009501188 |
| ING4 | 4 | 0.009501188 |
| ZWINT | 4 | 0.009501188 |
| ETFB | 4 | 0.009501188 |
| KRT36 | 4 | 0.009501188 |
| METTL7A | 4 | 0.009501188 |
| RALA | 4 | 0.009501188 |
| GFRA4 | 4 | 0.009501188 |
| MTARC2 | 4 | 0.009501188 |
| EPPIN | 4 | 0.009501188 |
| GLDN | 4 | 0.009501188 |
| CCDC103 | 4 | 0.009501188 |
| RPRD1B | 4 | 0.009501188 |
| FOS | 4 | 0.009501188 |
| TGFB3 | 4 | 0.009501188 |
| SNAI1 | 4 | 0.009501188 |
| ZNF579 | 4 | 0.009501188 |
| SHC3 | 4 | 0.009501188 |
| ARL10 | 4 | 0.009501188 |
| NEMP1 | 4 | 0.009501188 |
| DDHD1 | 4 | 0.009501188 |
| FAF2 | 4 | 0.009501188 |
| YIPF4 | 4 | 0.009501188 |
| ETF1 | 4 | 0.009501188 |
| GIMAP6 | 4 | 0.009501188 |
| HDHD3 | 4 | 0.009501188 |
| ZNF280C | 4 | 0.009501188 |
| RS1 | 4 | 0.009501188 |
| NAGA | 4 | 0.009501188 |
| TRIM27 | 4 | 0.009501188 |
| ATXN3 | 4 | 0.009501188 |
| DDX5 | 4 | 0.009501188 |
| GHDC | 4 | 0.009501188 |
| RCOR1 | 4 | 0.009501188 |
| TMEM252 | 4 | 0.009501188 |
| LAYN | 4 | 0.009501188 |
| RNF167 | 4 | 0.009501188 |
| RNF115 | 4 | 0.009501188 |
| SINHCAF | 4 | 0.009501188 |
| AKIRIN2 | 4 | 0.009501188 |
| SEC11C | 4 | 0.009501188 |
| AKR1D1 | 4 | 0.009501188 |
| WLS | 4 | 0.009501188 |
| SH3BP5L | 4 | 0.009501188 |
| CAPN11 | 4 | 0.009501188 |
| MBTD1 | 4 | 0.009501188 |
| GNA15 | 4 | 0.009501188 |
| OXCT1 | 4 | 0.009501188 |
| TMEM38B | 4 | 0.009501188 |
| NNMT | 4 | 0.009501188 |
| GTF2I | 4 | 0.009501188 |
| SULT2B1 | 4 | 0.009501188 |
| ZNF77 | 4 | 0.009501188 |
| KRTAP4-5 | 4 | 0.009501188 |
| SERPINA6 | 4 | 0.009501188 |
| CAMKK1 | 4 | 0.009501188 |
| TK1 | 4 | 0.009501188 |
| ZFP69 | 4 | 0.009501188 |
| CELA3B | 4 | 0.009501188 |
| MRPL32 | 4 | 0.009501188 |
| TAS2R41 | 4 | 0.009501188 |
| TMEM72 | 4 | 0.009501188 |
| GPR157 | 4 | 0.009501188 |
| HOXD9 | 4 | 0.009501188 |
| FOXD4L6 | 4 | 0.009501188 |
| YIPF3 | 4 | 0.009501188 |
| WEE1 | 4 | 0.009501188 |
| SEPHS2 | 4 | 0.009501188 |
| RPP38 | 4 | 0.009501188 |
| TAAR8 | 4 | 0.009501188 |
| ACADVL | 4 | 0.009501188 |
| DNASE1 | 4 | 0.009501188 |
| DPEP3 | 4 | 0.009501188 |
| ITGBL1 | 4 | 0.009501188 |
| TACR2 | 4 | 0.009501188 |
| ATP1B3 | 4 | 0.009501188 |
| RNF152 | 4 | 0.009501188 |
| TYW3 | 4 | 0.009501188 |
| TANGO2 | 4 | 0.009501188 |
| CDH15 | 4 | 0.009501188 |
| CPLX4 | 4 | 0.009501188 |
| AZIN2 | 4 | 0.009501188 |
| RCC1L | 4 | 0.009501188 |
| CCDC65 | 4 | 0.009501188 |
| MAP1LC3A | 4 | 0.009501188 |
| RDH8 | 4 | 0.009501188 |
| DNAJC18 | 4 | 0.009501188 |
| DDX53 | 4 | 0.009501188 |
| ARHGAP1 | 4 | 0.009501188 |
| SLC9A6 | 4 | 0.009501188 |
| NR2F1 | 4 | 0.009501188 |
| TRABD2A | 4 | 0.009501188 |
| RIPK2 | 4 | 0.009501188 |
| GPR84 | 4 | 0.009501188 |
| PRSS57 | 4 | 0.009501188 |
| ABRA | 4 | 0.009501188 |
| RASSF10 | 4 | 0.009501188 |
| CARD8 | 4 | 0.009501188 |
| OTOS | 4 | 0.009501188 |
| UBE2J2 | 4 | 0.009501188 |
| PGC | 4 | 0.009501188 |
| TRIM66 | 4 | 0.009501188 |
| RBPMS | 4 | 0.009501188 |
| RETREG2 | 4 | 0.009501188 |
| H2BC18 | 4 | 0.009501188 |
| MKNK2 | 4 | 0.009501188 |
| IGLV3-10 | 4 | 0.009501188 |
| PLEKHA3 | 4 | 0.009501188 |
| C2orf66 | 4 | 0.009501188 |
| CTSA | 4 | 0.009501188 |
| ALDH7A1 | 4 | 0.009501188 |
| ANAPC4 | 4 | 0.009501188 |
| KLHL35 | 4 | 0.009501188 |
| SPP2 | 4 | 0.009501188 |
| WDR31 | 4 | 0.009501188 |
| DCTN4 | 4 | 0.009501188 |
| GMPR2 | 4 | 0.009501188 |
| PROK2 | 4 | 0.009501188 |
| TMEM88 | 4 | 0.009501188 |
| TSEN54 | 4 | 0.009501188 |
| SDF2 | 4 | 0.009501188 |
| ZNF620 | 4 | 0.009501188 |
| RAB5A | 4 | 0.009501188 |
| HMOX2 | 4 | 0.009501188 |
| ITPK1 | 4 | 0.009501188 |
| CETN1 | 4 | 0.009501188 |
| PNLIPRP1 | 4 | 0.009501188 |
| SFTPB | 4 | 0.009501188 |
| SERGEF | 4 | 0.009501188 |
| HMBOX1 | 4 | 0.009501188 |
| ERVW-1 | 4 | 0.009501188 |
| BUB3 | 4 | 0.009501188 |
| GNAL | 4 | 0.009501188 |
| STK17A | 4 | 0.009501188 |
| CST4 | 4 | 0.009501188 |
| PRCP | 4 | 0.009501188 |
| RSU1 | 4 | 0.009501188 |
| GPR25 | 4 | 0.009501188 |
| BRAP | 4 | 0.009501188 |
| NR1I3 | 4 | 0.009501188 |
| C4BPA | 4 | 0.009501188 |
| MSRA | 4 | 0.009501188 |
| PIR | 4 | 0.009501188 |
| CD9 | 4 | 0.009501188 |
| THEM4 | 4 | 0.009501188 |
| BDKRB2 | 4 | 0.009501188 |
| TBX1 | 4 | 0.009501188 |
| FAM117A | 4 | 0.009501188 |
| FAM124B | 4 | 0.009501188 |
| C19orf18 | 4 | 0.009501188 |
| DDIT3 | 4 | 0.009501188 |
| NR1H2 | 4 | 0.009501188 |
| NMNAT2 | 4 | 0.009501188 |
| INHBB | 4 | 0.009501188 |
| F12 | 4 | 0.009501188 |
| NDUFB10 | 4 | 0.009501188 |
| IDH3A | 4 | 0.009501188 |
| ZNF581 | 4 | 0.009501188 |
| RNF212 | 4 | 0.009501188 |
| IGFBP1 | 4 | 0.009501188 |
| CALCOCO2 | 4 | 0.009501188 |
| VIPR1 | 4 | 0.009501188 |
| SKAP2 | 4 | 0.009501188 |
| FAM83A | 4 | 0.009501188 |
| ZNF691 | 4 | 0.009501188 |
| C1orf115 | 4 | 0.009501188 |
| EIF2AK2 | 4 | 0.009501188 |
| MLX | 4 | 0.009501188 |
| ELP6 | 4 | 0.009501188 |
| RASL11A | 4 | 0.009501188 |
| TPPP | 4 | 0.009501188 |
| RNF34 | 4 | 0.009501188 |
| CCR4 | 4 | 0.009501188 |
| PI4K2B | 4 | 0.009501188 |
| NUDCD3 | 4 | 0.009501188 |
| LYPLA1 | 4 | 0.009501188 |
| PIH1D2 | 4 | 0.009501188 |
| SAAL1 | 4 | 0.009501188 |
| KLK6 | 4 | 0.009501188 |
| RAB20 | 4 | 0.009501188 |
| ICAM1 | 4 | 0.009501188 |
| FGF17 | 4 | 0.009501188 |
| THG1L | 4 | 0.009501188 |
| EMX2 | 4 | 0.009501188 |
| H4C7 | 4 | 0.009501188 |
| PELO | 4 | 0.009501188 |
| NECTIN4 | 4 | 0.009501188 |
| ASB4 | 4 | 0.009501188 |
| LRWD1 | 4 | 0.009501188 |
| FAM216A | 4 | 0.009501188 |
| ZSCAN9 | 4 | 0.009501188 |
| AQP1 | 4 | 0.009501188 |
| NIPAL4 | 4 | 0.009501188 |
| OR10G3 | 4 | 0.009501188 |
| APEX2 | 4 | 0.009501188 |
| NSMCE1 | 4 | 0.009501188 |
| PSMC5 | 4 | 0.009501188 |
| COQ4 | 4 | 0.009501188 |
| OR2AG1 | 4 | 0.009501188 |
| CX3CL1 | 4 | 0.009501188 |
| THEM5 | 4 | 0.009501188 |
| DMKN | 4 | 0.009501188 |
| RNF130 | 4 | 0.009501188 |
| MMP20 | 4 | 0.009501188 |
| SEPTIN7 | 4 | 0.009501188 |
| ARHGEF33 | 4 | 0.009501188 |
| AGPAT5 | 4 | 0.009501188 |
| KLRC2 | 4 | 0.009501188 |
| SYT8 | 4 | 0.009501188 |
| FAM71E1 | 4 | 0.009501188 |
| H4C12 | 4 | 0.009501188 |
| RASSF1 | 4 | 0.009501188 |
| NUDT16L1 | 4 | 0.009501188 |
| LRG1 | 4 | 0.009501188 |
| FRZB | 4 | 0.009501188 |
| HEMGN | 4 | 0.009501188 |
| ERCC1 | 4 | 0.009501188 |
| CFAP20 | 4 | 0.009501188 |
| C6orf136 | 4 | 0.009501188 |
| SLC47A1 | 4 | 0.009501188 |
| ATG4A | 4 | 0.009501188 |
| TPPP3 | 4 | 0.009501188 |
| ETFDH | 4 | 0.009501188 |
| SPATA19 | 4 | 0.009501188 |
| RSRP1 | 4 | 0.009501188 |
| GALR2 | 4 | 0.009501188 |
| POC1B-GALNT4 | 4 | 0.009501188 |
| OR51F1 | 4 | 0.009501188 |
| STK16 | 4 | 0.009501188 |
| KCTD10 | 4 | 0.009501188 |
| POLR2E | 4 | 0.009501188 |
| CXorf65 | 4 | 0.009501188 |
| BMT2 | 4 | 0.009501188 |
| SBK2 | 4 | 0.009501188 |
| GNAT2 | 4 | 0.009501188 |
| AGMAT | 4 | 0.009501188 |
| VSIG1 | 4 | 0.009501188 |
| GPX2 | 4 | 0.009501188 |
| NANOS3 | 4 | 0.009501188 |
| SARS1 | 4 | 0.009501188 |
| CCSAP | 4 | 0.009501188 |
| TOMM34 | 4 | 0.009501188 |
| ATG7 | 4 | 0.009501188 |
| SAP30BP | 4 | 0.009501188 |
| RILP | 4 | 0.009501188 |
| GINS1 | 4 | 0.009501188 |
| ALKBH5 | 4 | 0.009501188 |
| GPATCH2 | 4 | 0.009501188 |
| ACTR8 | 4 | 0.009501188 |
| H2BC5 | 4 | 0.009501188 |
| CCR10 | 4 | 0.009501188 |
| MAPK8IP2 | 4 | 0.009501188 |
| NEK2 | 4 | 0.009501188 |
| CYGB | 4 | 0.009501188 |
| XAGE2 | 4 | 0.009501188 |
| ZNF582 | 4 | 0.009501188 |
| METTL8 | 4 | 0.009501188 |
| VPS35 | 4 | 0.009501188 |
| PPIL2 | 4 | 0.009501188 |
| FAM131C | 4 | 0.009501188 |
| BNIP2 | 4 | 0.009501188 |
| NMNAT1 | 4 | 0.009501188 |
| DHRS7 | 4 | 0.009501188 |
| ZFP69B | 4 | 0.009501188 |
| GOSR1 | 4 | 0.009501188 |
| MTSS2 | 4 | 0.009501188 |
| SLC6A14 | 4 | 0.009501188 |
| USP51 | 4 | 0.009501188 |
| GAL3ST1 | 4 | 0.009501188 |
| ANKRD2 | 4 | 0.009501188 |
| OR4X2 | 4 | 0.009501188 |
| OR51G1 | 4 | 0.009501188 |
| MNT | 4 | 0.009501188 |
| PLEKHF2 | 4 | 0.009501188 |
| THAP5 | 4 | 0.009501188 |
| MRPL9 | 4 | 0.009501188 |
| TRMT44 | 4 | 0.009501188 |
| LHX8 | 4 | 0.009501188 |
| PIGS | 4 | 0.009501188 |
| CA4 | 4 | 0.009501188 |
| CREB3L3 | 4 | 0.009501188 |
| SLC34A3 | 4 | 0.009501188 |
| DENND6B | 4 | 0.009501188 |
| TFB2M | 4 | 0.009501188 |
| VENTX | 4 | 0.009501188 |
| PIGA | 4 | 0.009501188 |
| NEU4 | 4 | 0.009501188 |
| PTGDR | 4 | 0.009501188 |
| RSC1A1 | 4 | 0.009501188 |
| PRKAB2 | 4 | 0.009501188 |
| KRT80 | 4 | 0.009501188 |
| AUH | 4 | 0.009501188 |
| GRAMD2B | 4 | 0.009501188 |
| SPANXB1 | 4 | 0.009501188 |
| PPP1R7 | 4 | 0.009501188 |
| GPRIN2 | 4 | 0.009501188 |
| PRKCZ | 4 | 0.009501188 |
| KIAA0895L | 4 | 0.009501188 |
| FGFBP1 | 4 | 0.009501188 |
| ZNF225 | 4 | 0.009501188 |
| ASAH2B | 4 | 0.009501188 |
| NRG2 | 4 | 0.009501188 |
| C4A | 4 | 0.009501188 |
| ZC3H14 | 4 | 0.009501188 |
| SYT11 | 4 | 0.009501188 |
| PELI2 | 4 | 0.009501188 |
| MICU1 | 4 | 0.009501188 |
| BSDC1 | 4 | 0.009501188 |
| RAC1 | 4 | 0.009501188 |
| DYDC2 | 4 | 0.009501188 |
| CCDC113 | 4 | 0.009501188 |
| CYP27A1 | 4 | 0.009501188 |
| OR7A10 | 4 | 0.009501188 |
| PROX2 | 4 | 0.009501188 |
| LPCAT2 | 4 | 0.009501188 |
| FUZ | 4 | 0.009501188 |
| RPS7 | 4 | 0.009501188 |
| NUDT10 | 4 | 0.009501188 |
| CTRL | 4 | 0.009501188 |
| PIM1 | 4 | 0.009501188 |
| OR13H1 | 4 | 0.009501188 |
| CNOT8 | 4 | 0.009501188 |
| OPN5 | 4 | 0.009501188 |
| THRA | 4 | 0.009501188 |
| PITX2 | 4 | 0.009501188 |
| IGHV3-16 | 4 | 0.009501188 |
| GDPD1 | 4 | 0.009501188 |
| SFTPD | 4 | 0.009501188 |
| ETV3 | 4 | 0.009501188 |
| GNPTG | 4 | 0.009501188 |
| PRMT6 | 4 | 0.009501188 |
| NXPE3 | 4 | 0.009501188 |
| MED8 | 4 | 0.009501188 |
| GMCL1 | 4 | 0.009501188 |
| TM4SF5 | 4 | 0.009501188 |
| SPG21 | 4 | 0.009501188 |
| OR4K14 | 4 | 0.009501188 |
| HINT3 | 4 | 0.009501188 |
| CCDC190 | 4 | 0.009501188 |
| ZNF488 | 4 | 0.009501188 |
| ERG28 | 4 | 0.009501188 |
| TMEM190 | 4 | 0.009501188 |
| LIN9 | 4 | 0.009501188 |
| LLPH | 4 | 0.009501188 |
| GPR108 | 4 | 0.009501188 |
| SERTAD1 | 4 | 0.009501188 |
| RAMP1 | 4 | 0.009501188 |
| PENK | 4 | 0.009501188 |
| AK1 | 4 | 0.009501188 |
| CILK1 | 4 | 0.009501188 |
| C10orf88 | 4 | 0.009501188 |
| CATIP | 4 | 0.009501188 |
| SERPINB9 | 4 | 0.009501188 |
| PTCD3 | 4 | 0.009501188 |
| SAE1 | 4 | 0.009501188 |
| H4C13 | 4 | 0.009501188 |
| STRA6 | 4 | 0.009501188 |
| ATG14 | 4 | 0.009501188 |
| JUNB | 4 | 0.009501188 |
| AKAP10 | 4 | 0.009501188 |
| OR14J1 | 4 | 0.009501188 |
| CTBS | 4 | 0.009501188 |
| SENP8 | 4 | 0.009501188 |
| OR1A1 | 4 | 0.009501188 |
| NFATC2IP | 4 | 0.009501188 |
| RABL2A | 4 | 0.009501188 |
| KCNJ14 | 4 | 0.009501188 |
| CCDC117 | 4 | 0.009501188 |
| CTDSP1 | 4 | 0.009501188 |
| PMP22 | 4 | 0.009501188 |
| TAS2R14 | 4 | 0.009501188 |
| DYRK2 | 4 | 0.009501188 |
| FRMD5 | 4 | 0.009501188 |
| SULT1A1 | 4 | 0.009501188 |
| OR52B4 | 4 | 0.009501188 |
| RCHY1 | 4 | 0.009501188 |
| IGHV4-34 | 4 | 0.009501188 |
| RDM1 | 4 | 0.009501188 |
| RAB27B | 4 | 0.009501188 |
| MARCHF11 | 4 | 0.009501188 |
| MPI | 4 | 0.009501188 |
| TDRD9 | 4 | 0.009501188 |
| RSPH3 | 4 | 0.009501188 |
| ZSCAN22 | 4 | 0.009501188 |
| RTL8B | 4 | 0.009501188 |
| FIGNL2 | 4 | 0.009501188 |
| FAM83F | 4 | 0.009501188 |
| CAMKMT | 4 | 0.009501188 |
| MRPS15 | 4 | 0.009501188 |
| DMRTA1 | 4 | 0.009501188 |
| VPS39 | 4 | 0.009501188 |
| PIGN | 4 | 0.009501188 |
| OR2T34 | 4 | 0.009501188 |
| FCRLA | 4 | 0.009501188 |
| ISL2 | 4 | 0.009501188 |
| NACAD | 4 | 0.009501188 |
| ATG10 | 4 | 0.009501188 |
| IL32 | 4 | 0.009501188 |
| RPL24 | 4 | 0.009501188 |
| YBX1 | 4 | 0.009501188 |
| SPATS2L | 4 | 0.009501188 |
| CDO1 | 4 | 0.009501188 |
| SH2B2 | 4 | 0.009501188 |
| SIRPB2 | 4 | 0.009501188 |
| AKR1C1 | 4 | 0.009501188 |
| AMZ2 | 4 | 0.009501188 |
| SLC16A11 | 4 | 0.009501188 |
| CELF1 | 4 | 0.009501188 |
| YWHAE | 4 | 0.009501188 |
| SGTA | 4 | 0.009501188 |
| C19orf47 | 4 | 0.009501188 |
| CCDC51 | 4 | 0.009501188 |
| POLR2J | 4 | 0.009501188 |
| NEIL2 | 4 | 0.009501188 |
| RASSF6 | 4 | 0.009501188 |
| SLC25A33 | 4 | 0.009501188 |
| RARS1 | 4 | 0.009501188 |
| MRPS31 | 4 | 0.009501188 |
| ABI2 | 4 | 0.009501188 |
| H4C4 | 4 | 0.009501188 |
| SPACA5B | 4 | 0.009501188 |
| SLAMF9 | 4 | 0.009501188 |
| RHBDL1 | 4 | 0.009501188 |
| OR1L6 | 4 | 0.009501188 |
| TPST1 | 4 | 0.009501188 |
| THAP2 | 4 | 0.009501188 |
| WDR48 | 4 | 0.009501188 |
| TSR1 | 4 | 0.009501188 |
| PRRC1 | 4 | 0.009501188 |
| OGFOD3 | 4 | 0.009501188 |
| SLAIN2 | 4 | 0.009501188 |
| NXF3 | 4 | 0.009501188 |
| NDUFAF4 | 4 | 0.009501188 |
| RNASE7 | 4 | 0.009501188 |
| MRPS7 | 4 | 0.009501188 |
| LYZL2 | 4 | 0.009501188 |
| MYL2 | 4 | 0.009501188 |
| HSPA14 | 4 | 0.009501188 |
| PNPLA4 | 4 | 0.009501188 |
| COQ7 | 4 | 0.009501188 |
| KRTAP10-9 | 4 | 0.009501188 |
| TNFSF15 | 4 | 0.009501188 |
| WASF1 | 4 | 0.009501188 |
| VEGFD | 4 | 0.009501188 |
| DUSP10 | 4 | 0.009501188 |
| SHOX2 | 4 | 0.009501188 |
| B3GNT3 | 4 | 0.009501188 |
| TIA1 | 4 | 0.009501188 |
| UGT1A1 | 4 | 0.009501188 |
| SHPK | 4 | 0.009501188 |
| ENDOG | 4 | 0.009501188 |
| H3C6 | 4 | 0.009501188 |
| BAG2 | 4 | 0.009501188 |
| TOR1A | 4 | 0.009501188 |
| ARHGAP26 | 4 | 0.009501188 |
| MTMR8 | 4 | 0.009501188 |
| MSRB3 | 4 | 0.009501188 |
| NEU3 | 4 | 0.009501188 |
| KRTAP5-8 | 4 | 0.009501188 |
| STX18 | 4 | 0.009501188 |
| DHCR7 | 4 | 0.009501188 |
| PRXL2A | 4 | 0.009501188 |
| AC231657.3 | 4 | 0.009501188 |
| ZKSCAN8 | 4 | 0.009501188 |
| TAF8 | 4 | 0.009501188 |
| ZNF12 | 4 | 0.009501188 |
| FAM122C | 4 | 0.009501188 |
| MS4A7 | 4 | 0.009501188 |
| IL18 | 4 | 0.009501188 |
| EVI2B | 4 | 0.009501188 |
| PAIP1 | 4 | 0.009501188 |
| CES2 | 4 | 0.009501188 |
| SCNN1D | 4 | 0.009501188 |
| C4B | 4 | 0.009501188 |
| TCTN3 | 4 | 0.009501188 |
| SGPL1 | 4 | 0.009501188 |
| DCAF8 | 4 | 0.009501188 |
| USP39 | 4 | 0.009501188 |
| CD48 | 4 | 0.009501188 |
| TMEM178A | 4 | 0.009501188 |
| FCGR3A | 4 | 0.009501188 |
| SNAPC3 | 4 | 0.009501188 |
| MANF | 4 | 0.009501188 |
| LCN12 | 4 | 0.009501188 |
| MZT2A | 4 | 0.009501188 |
| MC1R | 4 | 0.009501188 |
| COG3 | 4 | 0.009501188 |
| SEC22C | 4 | 0.009501188 |
| PRKAG3 | 4 | 0.009501188 |
| BRD9 | 4 | 0.009501188 |
| RNF14 | 4 | 0.009501188 |
| ZDHHC24 | 4 | 0.009501188 |
| AASDHPPT | 4 | 0.009501188 |
| ARHGEF39 | 4 | 0.009501188 |
| MRPL3 | 4 | 0.009501188 |
| OR6C2 | 4 | 0.009501188 |
| LRFN1 | 4 | 0.009501188 |
| ANKH | 4 | 0.009501188 |
| BDNF | 4 | 0.009501188 |
| CCDC127 | 4 | 0.009501188 |
| PDP1 | 4 | 0.009501188 |
| OR2T11 | 4 | 0.009501188 |
| GALNT16 | 4 | 0.009501188 |
| OR9G4 | 4 | 0.009501188 |
| GLYATL1 | 4 | 0.009501188 |
| PSMC2 | 4 | 0.009501188 |
| SERTAD2 | 4 | 0.009501188 |
| ACAD8 | 4 | 0.009501188 |
| ATP6V1H | 4 | 0.009501188 |
| CREBZF | 4 | 0.009501188 |
| ASB9 | 4 | 0.009501188 |
| SMPX | 4 | 0.009501188 |
| MC5R | 4 | 0.009501188 |
| MSS51 | 4 | 0.009501188 |
| CD27 | 4 | 0.009501188 |
| DHFR2 | 4 | 0.009501188 |
| BBOF1 | 4 | 0.009501188 |
| CD79A | 4 | 0.009501188 |
| ATG13 | 4 | 0.009501188 |
| DPY30 | 4 | 0.009501188 |
| RAD23B | 4 | 0.009501188 |
| AP3M1 | 4 | 0.009501188 |
| KCNIP4 | 4 | 0.009501188 |
| TARDBP | 4 | 0.009501188 |
| ZNF485 | 4 | 0.009501188 |
| PPP2CB | 4 | 0.009501188 |
| KIAA2013 | 4 | 0.009501188 |
| LYPD6B | 4 | 0.009501188 |
| TAF11 | 4 | 0.009501188 |
| KCMF1 | 4 | 0.009501188 |
| APOE | 4 | 0.009501188 |
| C17orf80 | 4 | 0.009501188 |
| RNASE9 | 4 | 0.009501188 |
| C11orf54 | 4 | 0.009501188 |
| METTL27 | 4 | 0.009501188 |
| ABRAXAS2 | 4 | 0.009501188 |
| GPATCH1 | 4 | 0.009501188 |
| SPACA3 | 4 | 0.009501188 |
| PIAS2 | 4 | 0.009501188 |
| AKT2 | 4 | 0.009501188 |
| NDUFS3 | 4 | 0.009501188 |
| GATA4 | 4 | 0.009501188 |
| FUCA2 | 4 | 0.009501188 |
| SLC25A6 | 4 | 0.009501188 |
| DENND1B | 4 | 0.009501188 |
| NR6A1 | 4 | 0.009501188 |
| CTSO | 4 | 0.009501188 |
| TMEM161A | 4 | 0.009501188 |
| RARRES1 | 4 | 0.009501188 |
| MARCHF5 | 4 | 0.009501188 |
| ZFP14 | 4 | 0.009501188 |
| VWDE | 4 | 0.009501188 |
| APCS | 4 | 0.009501188 |
| NPBWR2 | 4 | 0.009501188 |
| LHFPL2 | 4 | 0.009501188 |
| CALHM6 | 4 | 0.009501188 |
| VPS28 | 4 | 0.009501188 |
| SEPTIN10 | 4 | 0.009501188 |
| TSG101 | 4 | 0.009501188 |
| TPD52L1 | 4 | 0.009501188 |
| FZD5 | 4 | 0.009501188 |
| SCN2B | 4 | 0.009501188 |
| PPM1B | 4 | 0.009501188 |
| PEX11B | 4 | 0.009501188 |
| ADRB1 | 4 | 0.009501188 |
| AC004593.2 | 4 | 0.009501188 |
| NLGN2 | 4 | 0.009501188 |
| MAPKAPK5 | 4 | 0.009501188 |
| ELOVL5 | 4 | 0.009501188 |
| GOLPH3 | 4 | 0.009501188 |
| CEP43 | 4 | 0.009501188 |
| FAM167A | 4 | 0.009501188 |
| ZCCHC4 | 4 | 0.009501188 |
| DLST | 4 | 0.009501188 |
| MGP | 4 | 0.009501188 |
| TREM1 | 4 | 0.009501188 |
| PKIA | 4 | 0.009501188 |
| C12orf56 | 4 | 0.009501188 |
| TMEM33 | 4 | 0.009501188 |
| MAGEA3 | 4 | 0.009501188 |
| B3GALT5 | 4 | 0.009501188 |
| SIL1 | 4 | 0.009501188 |
| MPP7 | 4 | 0.009501188 |
| IL23R | 4 | 0.009501188 |
| FAM178B | 4 | 0.009501188 |
| GSS | 4 | 0.009501188 |
| CABP7 | 4 | 0.009501188 |
| RXYLT1 | 4 | 0.009501188 |
| TESK1 | 4 | 0.009501188 |
| SENP1 | 4 | 0.009501188 |
| NUBP1 | 4 | 0.009501188 |
| FBXW4 | 4 | 0.009501188 |
| TPM2 | 4 | 0.009501188 |
| FANK1 | 4 | 0.009501188 |
| MID1 | 4 | 0.009501188 |
| ADPRHL1 | 4 | 0.009501188 |
| EED | 4 | 0.009501188 |
| PRR5 | 4 | 0.009501188 |
| XRCC3 | 4 | 0.009501188 |
| TSSK4 | 4 | 0.009501188 |
| TTI2 | 4 | 0.009501188 |
| CATSPER2 | 4 | 0.009501188 |
| BCCIP | 4 | 0.009501188 |
| JKAMP | 4 | 0.009501188 |
| AMY1C | 4 | 0.009501188 |
| PCBP3 | 4 | 0.009501188 |
| CLHC1 | 4 | 0.009501188 |
| MIA | 4 | 0.009501188 |
| ZNF414 | 4 | 0.009501188 |
| DDX39A | 4 | 0.009501188 |
| TRMT61A | 4 | 0.009501188 |
| EMC3 | 4 | 0.009501188 |
| ARF4 | 4 | 0.009501188 |
| GNB1 | 4 | 0.009501188 |
| OR10A7 | 4 | 0.009501188 |
| NANOG | 4 | 0.009501188 |
| ZNF114 | 4 | 0.009501188 |
| BTN3A2 | 4 | 0.009501188 |
| PSMA2 | 4 | 0.009501188 |
| OR2T29 | 4 | 0.009501188 |
| STX1B | 4 | 0.009501188 |
| HOXB1 | 4 | 0.009501188 |
| TIMM17B | 4 | 0.009501188 |
| ARF1 | 4 | 0.009501188 |
| BEND7 | 4 | 0.009501188 |
| ELAC1 | 4 | 0.009501188 |
| NSDHL | 4 | 0.009501188 |
| LARGE2 | 4 | 0.009501188 |
| WIPI1 | 4 | 0.009501188 |
| OR5AU1 | 4 | 0.009501188 |
| DUSP12 | 4 | 0.009501188 |
| PRPS2 | 4 | 0.009501188 |
| WRNIP1 | 4 | 0.009501188 |
| OGFOD2 | 4 | 0.009501188 |
| UEVLD | 4 | 0.009501188 |
| ZNF169 | 4 | 0.009501188 |
| CTBP2 | 4 | 0.009501188 |
| ETFA | 4 | 0.009501188 |
| SPR | 4 | 0.009501188 |
| IFRD1 | 4 | 0.009501188 |
| PRRX1 | 4 | 0.009501188 |
| KREMEN1 | 4 | 0.009501188 |
| GOLGA6A | 4 | 0.009501188 |
| INHBC | 4 | 0.009501188 |
| GOLT1B | 4 | 0.009501188 |
| FOXP3 | 4 | 0.009501188 |
| DEDD | 4 | 0.009501188 |
| TMEM186 | 4 | 0.009501188 |
| SPACA1 | 4 | 0.009501188 |
| C9orf135 | 4 | 0.009501188 |
| ZNF213 | 4 | 0.009501188 |
| RNF146 | 4 | 0.009501188 |
| PRR5-ARHGAP8 | 4 | 0.009501188 |
| SDHD | 4 | 0.009501188 |
| TBKBP1 | 4 | 0.009501188 |
| E2F2 | 4 | 0.009501188 |
| KATNA1 | 4 | 0.009501188 |
| PPM1M | 4 | 0.009501188 |
| GTPBP8 | 4 | 0.009501188 |
| DGLUCY | 4 | 0.009501188 |
| TTLL11 | 4 | 0.009501188 |
| PRG2 | 4 | 0.009501188 |
| KMT5C | 4 | 0.009501188 |
| GPR82 | 4 | 0.009501188 |
| SSMEM1 | 4 | 0.009501188 |
| SUCNR1 | 4 | 0.009501188 |
| ARL6IP6 | 4 | 0.009501188 |
| PLK3 | 4 | 0.009501188 |
| CHRFAM7A | 4 | 0.009501188 |
| CCRL2 | 4 | 0.009501188 |
| BCL2L11 | 4 | 0.009501188 |
| ZNF385C | 4 | 0.009501188 |
| EGFL8 | 4 | 0.009501188 |
| HRAS | 4 | 0.009501188 |
| RGS19 | 4 | 0.009501188 |
| SLC22A23 | 4 | 0.009501188 |
| ACTR10 | 4 | 0.009501188 |
| SCTR | 4 | 0.009501188 |
| IFNG | 4 | 0.009501188 |
| GC | 4 | 0.009501188 |
| STC1 | 4 | 0.009501188 |
| NRF1 | 4 | 0.009501188 |
| ADRA2A | 4 | 0.009501188 |
| GCG | 4 | 0.009501188 |
| SMU1 | 4 | 0.009501188 |
| HOXC12 | 4 | 0.009501188 |
| DCUN1D1 | 4 | 0.009501188 |
| CENPV | 4 | 0.009501188 |
| RHOC | 4 | 0.009501188 |
| AP1S3 | 4 | 0.009501188 |
| MRPL46 | 4 | 0.009501188 |
| LIPA | 4 | 0.009501188 |
| TEDC1 | 4 | 0.009501188 |
| TCTEX1D1 | 4 | 0.009501188 |
| NAPG | 4 | 0.009501188 |
| H4-16 | 4 | 0.009501188 |
| CRYGB | 4 | 0.009501188 |
| COX6A2 | 4 | 0.009501188 |
| CHKA | 4 | 0.009501188 |
| OR1I1 | 4 | 0.009501188 |
| PFDN4 | 4 | 0.009501188 |
| WFDC5 | 4 | 0.009501188 |
| CAPG | 4 | 0.009501188 |
| FGF1 | 4 | 0.009501188 |
| ACRV1 | 4 | 0.009501188 |
| SH3RF2 | 4 | 0.009501188 |
| CUEDC2 | 4 | 0.009501188 |
| ACTRT3 | 4 | 0.009501188 |
| SCNN1A | 4 | 0.009501188 |
| RIPK3 | 4 | 0.009501188 |
| SPATA2 | 4 | 0.009501188 |
| SGK3 | 4 | 0.009501188 |
| KCNG3 | 4 | 0.009501188 |
| CMTM1 | 4 | 0.009501188 |
| BNIPL | 4 | 0.009501188 |
| C16orf89 | 4 | 0.009501188 |
| PEDS1-UBE2V1 | 4 | 0.009501188 |
| IGHV1-46 | 4 | 0.009501188 |
| SLC30A2 | 4 | 0.009501188 |
| NNT | 4 | 0.009501188 |
| LGALS13 | 4 | 0.009501188 |
| DTX3 | 4 | 0.009501188 |
| CCZ1B | 4 | 0.009501188 |
| HSD17B10 | 4 | 0.009501188 |
| PSMF1 | 4 | 0.009501188 |
| CD177 | 4 | 0.009501188 |
| SAXO1 | 4 | 0.009501188 |
| TYMS | 4 | 0.009501188 |
| CYB5R3 | 4 | 0.009501188 |
| PCK1 | 4 | 0.009501188 |
| TMEM43 | 4 | 0.009501188 |
| CAV2 | 4 | 0.009501188 |
| OR2M7 | 4 | 0.009501188 |
| AGPS | 4 | 0.009501188 |
| NIT1 | 4 | 0.009501188 |
| BMP6 | 4 | 0.009501188 |
| ZBTB44 | 4 | 0.009501188 |
| CHAF1B | 4 | 0.009501188 |
| GSTA5 | 4 | 0.009501188 |
| MSTN | 4 | 0.009501188 |
| SLC3A1 | 4 | 0.009501188 |
| ZNF787 | 4 | 0.009501188 |
| NDUFS2 | 4 | 0.009501188 |
| SLC25A44 | 4 | 0.009501188 |
| ATP4B | 4 | 0.009501188 |
| UPK1A | 4 | 0.009501188 |
| H3C4 | 4 | 0.009501188 |
| TEX264 | 4 | 0.009501188 |
| CREB3L2 | 4 | 0.009501188 |
| PKDCC | 4 | 0.009501188 |
| SSH3 | 4 | 0.009501188 |
| TPD52L3 | 4 | 0.009501188 |
| PARK7 | 4 | 0.009501188 |
| CMA1 | 4 | 0.009501188 |
| MPV17L2 | 4 | 0.009501188 |
| ZDHHC3 | 4 | 0.009501188 |
| CD7 | 4 | 0.009501188 |
| IL4 | 4 | 0.009501188 |
| PLAU | 4 | 0.009501188 |
| CZIB | 4 | 0.009501188 |
| IRAG2 | 4 | 0.009501188 |
| EIF2D | 4 | 0.009501188 |
| ADM2 | 4 | 0.009501188 |
| PDK4 | 4 | 0.009501188 |
| U2AF1L4 | 4 | 0.009501188 |
| CIDEC | 4 | 0.009501188 |
| SPACA7 | 4 | 0.009501188 |
| KRT14 | 4 | 0.009501188 |
| TRIM14 | 4 | 0.009501188 |
| AC013477.1 | 4 | 0.009501188 |
| CROCC2 | 4 | 0.009501188 |
| ANKRD34B | 4 | 0.009501188 |
| WNT7B | 4 | 0.009501188 |
| PRB4 | 4 | 0.009501188 |
| NSUN4 | 4 | 0.009501188 |
| PMPCA | 4 | 0.009501188 |
| FBXO44 | 4 | 0.009501188 |
| MED4 | 4 | 0.009501188 |
| DKK3 | 4 | 0.009501188 |
| DNAJB4 | 4 | 0.009501188 |
| KAT5 | 4 | 0.009501188 |
| RBMX | 4 | 0.009501188 |
| VPREB1 | 4 | 0.009501188 |
| WDFY2 | 4 | 0.009501188 |
| PIK3IP1 | 4 | 0.009501188 |
| BCL2L1 | 4 | 0.009501188 |
| ING2 | 4 | 0.009501188 |
| SLC7A7 | 4 | 0.009501188 |
| LRRC37A | 4 | 0.009501188 |
| EMILIN3 | 4 | 0.009501188 |
| SEC22A | 4 | 0.009501188 |
| CMSS1 | 4 | 0.009501188 |
| CD79B | 4 | 0.009501188 |
| MTG2 | 4 | 0.009501188 |
| DYNAP | 4 | 0.009501188 |
| ZYX | 4 | 0.009501188 |
| IFNA5 | 4 | 0.009501188 |
| TPGS2 | 4 | 0.009501188 |
| FAM81A | 4 | 0.009501188 |
| TMEM38A | 4 | 0.009501188 |
| ITPA | 4 | 0.009501188 |
| WASF2 | 4 | 0.009501188 |
| NT5DC1 | 4 | 0.009501188 |
| EIF2B4 | 4 | 0.009501188 |
| SLC35E1 | 4 | 0.009501188 |
| SLC36A2 | 4 | 0.009501188 |
| SYT10 | 4 | 0.009501188 |
| OSBP2 | 4 | 0.009501188 |
| SLBP | 4 | 0.009501188 |
| CACNG4 | 4 | 0.009501188 |
| PIGM | 4 | 0.009501188 |
| NMT2 | 4 | 0.009501188 |
| NSMF | 4 | 0.009501188 |
| LRRC8B | 4 | 0.009501188 |
| NAT2 | 4 | 0.009501188 |
| KCP | 4 | 0.009501188 |
| TAFA4 | 4 | 0.009501188 |
| NFYA | 4 | 0.009501188 |
| HACD4 | 4 | 0.009501188 |
| FAM174A | 4 | 0.009501188 |
| HERPUD1 | 4 | 0.009501188 |
| DUSP3 | 4 | 0.009501188 |
| GADD45GIP1 | 4 | 0.009501188 |
| CCDC90B | 4 | 0.009501188 |
| TTC26 | 4 | 0.009501188 |
| CSNK1G1 | 4 | 0.009501188 |
| OR6C74 | 4 | 0.009501188 |
| BTBD1 | 4 | 0.009501188 |
| PCGF5 | 4 | 0.009501188 |
| TLE6 | 4 | 0.009501188 |
| SCRN2 | 4 | 0.009501188 |
| CORO2A | 4 | 0.009501188 |
| BCL2L14 | 4 | 0.009501188 |
| NINJ1 | 4 | 0.009501188 |
| PSMG2 | 4 | 0.009501188 |
| CST11 | 4 | 0.009501188 |
| PRKAR2B | 4 | 0.009501188 |
| USP50 | 4 | 0.009501188 |
| CTLA4 | 4 | 0.009501188 |
| ALG2 | 4 | 0.009501188 |
| VEGFA | 4 | 0.009501188 |
| SPAG4 | 4 | 0.009501188 |
| SIRT7 | 4 | 0.009501188 |
| VAX1 | 4 | 0.009501188 |
| TSC22D4 | 4 | 0.009501188 |
| BCLAF3 | 4 | 0.009501188 |
| VSIG4 | 4 | 0.009501188 |
| DNASE2B | 4 | 0.009501188 |
| OMA1 | 4 | 0.009501188 |
| TMIGD3 | 4 | 0.009501188 |
| DEF8 | 4 | 0.009501188 |
| RMDN2 | 4 | 0.009501188 |
| ZMAT2 | 4 | 0.009501188 |
| CD47 | 4 | 0.009501188 |
| EBNA1BP2 | 4 | 0.009501188 |
| IL21 | 4 | 0.009501188 |
| SGK2 | 4 | 0.009501188 |
| NEK6 | 4 | 0.009501188 |
| RTN4RL2 | 4 | 0.009501188 |
| SPSB1 | 4 | 0.009501188 |
| DUS1L | 4 | 0.009501188 |
| CAPZA1 | 4 | 0.009501188 |
| FZD4 | 4 | 0.009501188 |
| FOXR2 | 4 | 0.009501188 |
| WBP1L | 4 | 0.009501188 |
| KLF1 | 4 | 0.009501188 |
| ANKRD20A1 | 4 | 0.009501188 |
| ASPSCR1 | 4 | 0.009501188 |
| TAL1 | 4 | 0.009501188 |
| CDCA8 | 4 | 0.009501188 |
| FCMR | 4 | 0.009501188 |
| PXMP2 | 4 | 0.009501188 |
| SOWAHD | 4 | 0.009501188 |
| PIEZO1 | 4 | 0.009501188 |
| PTGER2 | 4 | 0.009501188 |
| XPA | 4 | 0.009501188 |
| PEX12 | 4 | 0.009501188 |
| SLC15A4 | 4 | 0.009501188 |
| KRBA2 | 4 | 0.009501188 |
| HYKK | 4 | 0.009501188 |
| RPS5 | 4 | 0.009501188 |
| GPR63 | 4 | 0.009501188 |
| IL2RB | 4 | 0.009501188 |
| BAMBI | 4 | 0.009501188 |
| CD55 | 4 | 0.009501188 |
| HACD3 | 4 | 0.009501188 |
| SCAMP5 | 4 | 0.009501188 |
| TMPRSS11F | 4 | 0.009501188 |
| TMEM121B | 4 | 0.009501188 |
| SERPINF1 | 4 | 0.009501188 |
| SPACA9 | 4 | 0.009501188 |
| CCDC106 | 4 | 0.009501188 |
| NFIX | 4 | 0.009501188 |
| ZNF823 | 4 | 0.009501188 |
| MOB3C | 4 | 0.009501188 |
| MCEE | 4 | 0.009501188 |
| MTRES1 | 4 | 0.009501188 |
| TTC9 | 4 | 0.009501188 |
| PRDM8 | 4 | 0.009501188 |
| PTPDC1 | 4 | 0.009501188 |
| TUBB4B | 4 | 0.009501188 |
| GINS3 | 4 | 0.009501188 |
| OR8G5 | 4 | 0.009501188 |
| LRRC61 | 4 | 0.009501188 |
| DCP1A | 4 | 0.009501188 |
| CHIC2 | 4 | 0.009501188 |
| LCK | 4 | 0.009501188 |
| SPP1 | 4 | 0.009501188 |
| NAT8L | 4 | 0.009501188 |
| FPGS | 4 | 0.009501188 |
| TRIM7 | 4 | 0.009501188 |
| COA8 | 4 | 0.009501188 |
| MIPOL1 | 4 | 0.009501188 |
| PYROXD1 | 4 | 0.009501188 |
| STK38L | 4 | 0.009501188 |
| IGHD | 4 | 0.009501188 |
| PAQR5 | 4 | 0.009501188 |
| ATF1 | 4 | 0.009501188 |
| WDR86 | 4 | 0.009501188 |
| MOSPD1 | 4 | 0.009501188 |
| FBXL6 | 4 | 0.009501188 |
| BCL2L2 | 4 | 0.009501188 |
| TMEM164 | 4 | 0.009501188 |
| ASB8 | 4 | 0.009501188 |
| MRPL13 | 4 | 0.009501188 |
| CNBD2 | 4 | 0.009501188 |
| IBA57 | 4 | 0.009501188 |
| FUCA1 | 4 | 0.009501188 |
| C16orf96 | 4 | 0.009501188 |
| CPN2 | 4 | 0.009501188 |
| FNDC11 | 4 | 0.009501188 |
| SMYD2 | 4 | 0.009501188 |
| BST1 | 4 | 0.009501188 |
| PDCD4 | 4 | 0.009501188 |
| SLC29A1 | 4 | 0.009501188 |
| CLPTM1L | 4 | 0.009501188 |
| QPRT | 4 | 0.009501188 |
| CYRIB | 4 | 0.009501188 |
| PSMC1 | 4 | 0.009501188 |
| PCGF3 | 4 | 0.009501188 |
| RALGPS1 | 4 | 0.009501188 |
| NIPAL1 | 4 | 0.009501188 |
| ZNF576 | 4 | 0.009501188 |
| PPP1CC | 4 | 0.009501188 |
| MREG | 4 | 0.009501188 |
| C11orf65 | 4 | 0.009501188 |
| SGPP2 | 4 | 0.009501188 |
| SHD | 4 | 0.009501188 |
| NAPA | 4 | 0.009501188 |
| ZFP91 | 4 | 0.009501188 |
| PRXL2C | 4 | 0.009501188 |
| CSF1 | 4 | 0.009501188 |
| SLC35F4 | 4 | 0.009501188 |
| RRP1 | 4 | 0.009501188 |
| DUSP5 | 4 | 0.009501188 |
| IFIT3 | 4 | 0.009501188 |
| PAOX | 4 | 0.009501188 |
| ABHD8 | 4 | 0.009501188 |
| FCRLB | 4 | 0.009501188 |
| GPR146 | 4 | 0.009501188 |
| NSUN3 | 4 | 0.009501188 |
| OR4C12 | 4 | 0.009501188 |
| LTB | 4 | 0.009501188 |
| OR52B6 | 4 | 0.009501188 |
| OR6C1 | 4 | 0.009501188 |
| MLF1 | 4 | 0.009501188 |
| ACP3 | 4 | 0.009501188 |
| FIBIN | 4 | 0.009501188 |
| TNFSF9 | 4 | 0.009501188 |
| CCR8 | 4 | 0.009501188 |
| EMC2 | 4 | 0.009501188 |
| TSSK6 | 4 | 0.009501188 |
| RNF215 | 4 | 0.009501188 |
| MID1IP1 | 4 | 0.009501188 |
| SOST | 4 | 0.009501188 |
| PIMREG | 4 | 0.009501188 |
| NXPE1 | 4 | 0.009501188 |
| METTL7B | 4 | 0.009501188 |
| GRHL1 | 4 | 0.009501188 |
| OR10A2 | 4 | 0.009501188 |
| AFMID | 4 | 0.009501188 |
| GYPB | 4 | 0.009501188 |
| EIF5 | 4 | 0.009501188 |
| THOC3 | 4 | 0.009501188 |
| HAX1 | 4 | 0.009501188 |
| DAP3 | 4 | 0.009501188 |
| FBXO6 | 4 | 0.009501188 |
| PLEK2 | 4 | 0.009501188 |
| CLEC4E | 4 | 0.009501188 |
| GET1-SH3BGR | 4 | 0.009501188 |
| NUDT9 | 4 | 0.009501188 |
| CMC1 | 4 | 0.009501188 |
| TP53I13 | 4 | 0.009501188 |
| FAM53C | 4 | 0.009501188 |
| UBAC2 | 4 | 0.009501188 |
| ADSS2 | 4 | 0.009501188 |
| SH3YL1 | 4 | 0.009501188 |
| AC068580.4 | 4 | 0.009501188 |
| TP53TG5 | 4 | 0.009501188 |
| PGAM1 | 4 | 0.009501188 |
| RXFP4 | 4 | 0.009501188 |
| MMP14 | 4 | 0.009501188 |
| OR5AK2 | 4 | 0.009501188 |
| TFEB | 4 | 0.009501188 |
| CTDNEP1 | 4 | 0.009501188 |
| GPSM1 | 4 | 0.009501188 |
| DRG1 | 4 | 0.009501188 |
| SCLY | 4 | 0.009501188 |
| IL20 | 4 | 0.009501188 |
| SLC7A13 | 4 | 0.009501188 |
| TMEM47 | 4 | 0.009501188 |
| ULK3 | 4 | 0.009501188 |
| GLT1D1 | 4 | 0.009501188 |
| PARP11 | 4 | 0.009501188 |
| KCNE4 | 4 | 0.009501188 |
| ORM2 | 4 | 0.009501188 |
| OR7C2 | 4 | 0.009501188 |
| RFPL1 | 4 | 0.009501188 |
| ZNF552 | 4 | 0.009501188 |
| VXN | 4 | 0.009501188 |
| GATA6 | 4 | 0.009501188 |
| ZNF76 | 4 | 0.009501188 |
| BORA | 4 | 0.009501188 |
| C14orf28 | 4 | 0.009501188 |
| IL22RA2 | 4 | 0.009501188 |
| ZNF2 | 4 | 0.009501188 |
| PRRX2 | 4 | 0.009501188 |
| AMY2B | 4 | 0.009501188 |
| SRY | 4 | 0.009501188 |
| CALCOCO1 | 4 | 0.009501188 |
| DALRD3 | 4 | 0.009501188 |
| STX7 | 4 | 0.009501188 |
| AKR1E2 | 4 | 0.009501188 |
| SLC25A53 | 4 | 0.009501188 |
| HLA-DMA | 4 | 0.009501188 |
| RGS8 | 4 | 0.009501188 |
| PLEKHB1 | 4 | 0.009501188 |
| TAPBP | 4 | 0.009501188 |
| RTN4R | 4 | 0.009501188 |
| CEP57L1 | 4 | 0.009501188 |
| CFP | 4 | 0.009501188 |
| OR11A1 | 4 | 0.009501188 |
| MFF | 4 | 0.009501188 |
| SUGCT | 4 | 0.009501188 |
| KIN | 4 | 0.009501188 |
| KRTAP1-5 | 4 | 0.009501188 |
| SLC35E3 | 4 | 0.009501188 |
| PLIN5 | 4 | 0.009501188 |
| AGAP4 | 4 | 0.009501188 |
| USB1 | 4 | 0.009501188 |
| MAL | 4 | 0.009501188 |
| RPF1 | 4 | 0.009501188 |
| ZPLD1 | 4 | 0.009501188 |
| CXCL16 | 4 | 0.009501188 |
| G6PC | 4 | 0.009501188 |
| PTF1A | 4 | 0.009501188 |
| ORC4 | 4 | 0.009501188 |
| RND1 | 4 | 0.009501188 |
| EGLN3 | 4 | 0.009501188 |
| H2AC6 | 4 | 0.009501188 |
| FRG2B | 4 | 0.009501188 |
| UGT2B17 | 4 | 0.009501188 |
| NBPF1 | 4 | 0.009501188 |
| EIF3M | 4 | 0.009501188 |
| AATF | 4 | 0.009501188 |
| ATP6V0A2 | 4 | 0.009501188 |
| ITIH4 | 4 | 0.009501188 |
| LRRC58 | 4 | 0.009501188 |
| ARL13A | 4 | 0.009501188 |
| KLK8 | 4 | 0.009501188 |
| EIF3H | 4 | 0.009501188 |
| SCP2 | 4 | 0.009501188 |
| HSD3B7 | 4 | 0.009501188 |
| OR5P3 | 4 | 0.009501188 |
| RPE | 4 | 0.009501188 |
| RAB7A | 4 | 0.009501188 |
| RDH13 | 4 | 0.009501188 |
| ZNF23 | 4 | 0.009501188 |
| SENP2 | 4 | 0.009501188 |
| RAB8B | 4 | 0.009501188 |
| RTBDN | 4 | 0.009501188 |
| CGA | 4 | 0.009501188 |
| SLC30A6 | 4 | 0.009501188 |
| UBD | 4 | 0.009501188 |
| HACL1 | 4 | 0.009501188 |
| RCAN2 | 4 | 0.009501188 |
| DRAXIN | 4 | 0.009501188 |
| SETD7 | 4 | 0.009501188 |
| WSB2 | 4 | 0.009501188 |
| CCNA2 | 4 | 0.009501188 |
| LRRC25 | 4 | 0.009501188 |
| FN3K | 4 | 0.009501188 |
| LZTFL1 | 4 | 0.009501188 |
| GPN2 | 4 | 0.009501188 |
| SFRP5 | 4 | 0.009501188 |
| WDR74 | 4 | 0.009501188 |
| AJUBA | 4 | 0.009501188 |
| TMEM150C | 4 | 0.009501188 |
| IFNL2 | 4 | 0.009501188 |
| MMP1 | 4 | 0.009501188 |
| HSD17B4 | 4 | 0.009501188 |
| KCNK4 | 4 | 0.009501188 |
| B3GNT8 | 4 | 0.009501188 |
| CYP2U1 | 4 | 0.009501188 |
| STARD6 | 4 | 0.009501188 |
| TBC1D13 | 4 | 0.009501188 |
| PATE1 | 4 | 0.009501188 |
| MORF4L2 | 4 | 0.009501188 |
| PIFO | 4 | 0.009501188 |
| ACTR3 | 4 | 0.009501188 |
| AP5B1 | 4 | 0.009501188 |
| CTNNAL1 | 4 | 0.009501188 |
| TMEM165 | 4 | 0.009501188 |
| STRAP | 4 | 0.009501188 |
| KCNC4 | 4 | 0.009501188 |
| IFT46 | 4 | 0.009501188 |
| TSGA13 | 4 | 0.009501188 |
| PKNOX1 | 4 | 0.009501188 |
| MAZ | 4 | 0.009501188 |
| MAMSTR | 4 | 0.009501188 |
| ADH1B | 4 | 0.009501188 |
| GPR119 | 4 | 0.009501188 |
| PLAGL1 | 4 | 0.009501188 |
| LETM2 | 4 | 0.009501188 |
| PLEKHA8 | 4 | 0.009501188 |
| ZDHHC9 | 4 | 0.009501188 |
| GSTM2 | 4 | 0.009501188 |
| HOXD13 | 4 | 0.009501188 |
| RPL35 | 4 | 0.009501188 |
| NPY1R | 4 | 0.009501188 |
| BHLHE40 | 4 | 0.009501188 |
| GSC | 4 | 0.009501188 |
| HELT | 4 | 0.009501188 |
| NOXA1 | 4 | 0.009501188 |
| ERAL1 | 4 | 0.009501188 |
| ZNF501 | 4 | 0.009501188 |
| ZNF195 | 4 | 0.009501188 |
| STK32C | 4 | 0.009501188 |
| FBLN5 | 4 | 0.009501188 |
| FOXJ3 | 4 | 0.009501188 |
| CXCR3 | 4 | 0.009501188 |
| CIAPIN1 | 4 | 0.009501188 |
| CPA3 | 4 | 0.009501188 |
| PTPN18 | 4 | 0.009501188 |
| TMEM37 | 4 | 0.009501188 |
| CHAC1 | 4 | 0.009501188 |
| RPL7 | 4 | 0.009501188 |
| AAR2 | 4 | 0.009501188 |
| RAB3IL1 | 4 | 0.009501188 |
| SLC16A3 | 4 | 0.009501188 |
| A4GNT | 4 | 0.009501188 |
| NUDT14 | 4 | 0.009501188 |
| ALDH3B2 | 4 | 0.009501188 |
| STX1A | 4 | 0.009501188 |
| BROX | 4 | 0.009501188 |
| GRPR | 4 | 0.009501188 |
| TMEM237 | 4 | 0.009501188 |
| HEY1 | 4 | 0.009501188 |
| TMC7 | 4 | 0.009501188 |
| HGH1 | 4 | 0.009501188 |
| ACBD3 | 4 | 0.009501188 |
| LGALS14 | 4 | 0.009501188 |
| TRMO | 4 | 0.009501188 |
| KLK11 | 4 | 0.009501188 |
| DFFA | 4 | 0.009501188 |
| SLC35G1 | 4 | 0.009501188 |
| NDUFB5 | 4 | 0.009501188 |
| CLEC1A | 4 | 0.009501188 |
| SLC1A4 | 4 | 0.009501188 |
| ATG4C | 4 | 0.009501188 |
| FAM8A1 | 4 | 0.009501188 |
| SDC1 | 4 | 0.009501188 |
| BOD1 | 4 | 0.009501188 |
| LYSMD2 | 4 | 0.009501188 |
| SBK1 | 4 | 0.009501188 |
| DLL4 | 4 | 0.009501188 |
| ZNF705A | 4 | 0.009501188 |
| KLHL15 | 4 | 0.009501188 |
| CCDC197 | 4 | 0.009501188 |
| MEOX1 | 4 | 0.009501188 |
| FASLG | 4 | 0.009501188 |
| IFNGR1 | 4 | 0.009501188 |
| RRH | 4 | 0.009501188 |
| UBE2A | 4 | 0.009501188 |
| LSM10 | 4 | 0.009501188 |
| TSPAN12 | 4 | 0.009501188 |
| MDFIC | 4 | 0.009501188 |
| PTGES3L-AARSD1 | 4 | 0.009501188 |
| C17orf58 | 4 | 0.009501188 |
| NUS1 | 4 | 0.009501188 |
| TYW5 | 4 | 0.009501188 |
| RAD23A | 4 | 0.009501188 |
| DHRS1 | 4 | 0.009501188 |
| OSTN | 4 | 0.009501188 |
| FTHL17 | 4 | 0.009501188 |
| NTAQ1 | 4 | 0.009501188 |
| DMRTC2 | 4 | 0.009501188 |
| IMPACT | 4 | 0.009501188 |
| SERPINB4 | 4 | 0.009501188 |
| LGALS9B | 4 | 0.009501188 |
| DCBLD1 | 4 | 0.009501188 |
| EYA3 | 4 | 0.009501188 |
| OTUB1 | 4 | 0.009501188 |
| SNX30 | 4 | 0.009501188 |
| WDR4 | 4 | 0.009501188 |
| DTNBP1 | 4 | 0.009501188 |
| CCDC142 | 4 | 0.009501188 |
| CLDN12 | 4 | 0.009501188 |
| XG | 4 | 0.009501188 |
| C18orf25 | 4 | 0.009501188 |
| MICOS13 | 4 | 0.009501188 |
| OR52M1 | 4 | 0.009501188 |
| PPP4R2 | 4 | 0.009501188 |
| CD38 | 4 | 0.009501188 |
| GSTM4 | 4 | 0.009501188 |
| MDH2 | 4 | 0.009501188 |
| TEKT2 | 4 | 0.009501188 |
| UQCRB | 4 | 0.009501188 |
| LSG1 | 4 | 0.009501188 |
| SLC22A12 | 4 | 0.009501188 |
| PLEKHG7 | 4 | 0.009501188 |
| ADH5 | 4 | 0.009501188 |
| DDX28 | 4 | 0.009501188 |
| CENPBD1 | 4 | 0.009501188 |
| ELP5 | 4 | 0.009501188 |
| MTFR1 | 4 | 0.009501188 |
| PLAAT2 | 4 | 0.009501188 |
| ZNF627 | 4 | 0.009501188 |
| AC139530.2 | 4 | 0.009501188 |
| ABI1 | 4 | 0.009501188 |
| RPS6KB2 | 4 | 0.009501188 |
| KCNJ13 | 4 | 0.009501188 |
| LFNG | 4 | 0.009501188 |
| CDK11A | 4 | 0.009501188 |
| PSMD4 | 4 | 0.009501188 |
| SLC25A10 | 4 | 0.009501188 |
| GSTA1 | 4 | 0.009501188 |
| ESRP2 | 4 | 0.009501188 |
| POU5F2 | 4 | 0.009501188 |
| WDR43 | 4 | 0.009501188 |
| YARS1 | 4 | 0.009501188 |
| PNCK | 4 | 0.009501188 |
| GBX2 | 4 | 0.009501188 |
| RP9 | 4 | 0.009501188 |
| KNSTRN | 4 | 0.009501188 |
| EIF3F | 4 | 0.009501188 |
| CCNB2 | 4 | 0.009501188 |
| UQCRC2 | 4 | 0.009501188 |
| TM9SF4 | 4 | 0.009501188 |
| MALRD1 | 4 | 0.009501188 |
| GIMAP5 | 4 | 0.009501188 |
| GYPC | 4 | 0.009501188 |
| SQSTM1 | 4 | 0.009501188 |
| GARS1 | 4 | 0.009501188 |
| DAOA | 4 | 0.009501188 |
| CD8B | 4 | 0.009501188 |
| MAP2K5 | 4 | 0.009501188 |
| IL7 | 4 | 0.009501188 |
| AMH | 4 | 0.009501188 |
| CEP85 | 4 | 0.009501188 |
| OR11H12 | 4 | 0.009501188 |
| ODAPH | 4 | 0.009501188 |
| TNFRSF6B | 4 | 0.009501188 |
| LRRC55 | 4 | 0.009501188 |
| CRX | 4 | 0.009501188 |
| DNAJC5G | 4 | 0.009501188 |
| ZNF75D | 4 | 0.009501188 |
| PPID | 4 | 0.009501188 |
| RAB4A | 4 | 0.009501188 |
| B4GALT7 | 4 | 0.009501188 |
| PGM3 | 4 | 0.009501188 |
| SFT2D1 | 4 | 0.009501188 |
| TCEAL1 | 4 | 0.009501188 |
| ZNHIT1 | 4 | 0.009501188 |
| GTPBP6 | 4 | 0.009501188 |
| WNT2 | 4 | 0.009501188 |
| HNRNPA2B1 | 4 | 0.009501188 |
| BCAS2 | 4 | 0.009501188 |
| PSTPIP2 | 4 | 0.009501188 |
| GPR150 | 4 | 0.009501188 |
| TTPAL | 4 | 0.009501188 |
| MBOAT7 | 4 | 0.009501188 |
| IFNA14 | 4 | 0.009501188 |
| UTP23 | 4 | 0.009501188 |
| S100A7 | 4 | 0.009501188 |
| FLOT2 | 4 | 0.009501188 |
| PLAAT1 | 4 | 0.009501188 |
| ZNF682 | 4 | 0.009501188 |
| RFT1 | 4 | 0.009501188 |
| ZNF394 | 4 | 0.009501188 |
| TMEM169 | 4 | 0.009501188 |
| PTPN7 | 4 | 0.009501188 |
| KDM5D | 4 | 0.009501188 |
| VNN2 | 4 | 0.009501188 |
| NSG2 | 4 | 0.009501188 |
| ZNF613 | 4 | 0.009501188 |
| TM6SF1 | 4 | 0.009501188 |
| CENPQ | 4 | 0.009501188 |
| TTC39B | 4 | 0.009501188 |
| MBD2 | 4 | 0.009501188 |
| IMPA2 | 4 | 0.009501188 |
| NUTM2D | 4 | 0.009501188 |
| ATXN10 | 4 | 0.009501188 |
| SHB | 4 | 0.009501188 |
| REP15 | 4 | 0.009501188 |
| KCNMB1 | 4 | 0.009501188 |
| DLK2 | 4 | 0.009501188 |
| AP5S1 | 4 | 0.009501188 |
| FOXJ2 | 4 | 0.009501188 |
| B3GNT5 | 4 | 0.009501188 |
| SSC4D | 4 | 0.009501188 |
| SAT1 | 4 | 0.009501188 |
| HSD17B13 | 4 | 0.009501188 |
| IMMP2L | 4 | 0.009501188 |
| MROH2A | 4 | 0.009501188 |
| GPR176 | 4 | 0.009501188 |
| TEX37 | 4 | 0.009501188 |
| NAA11 | 4 | 0.009501188 |
| CTHRC1 | 4 | 0.009501188 |
| TADA1 | 4 | 0.009501188 |
| FAM71F1 | 4 | 0.009501188 |
| PPP1CB | 4 | 0.009501188 |
| FATE1 | 4 | 0.009501188 |
| TSPO2 | 4 | 0.009501188 |
| RAB42 | 4 | 0.009501188 |
| TMEM176B | 4 | 0.009501188 |
| RNF138 | 4 | 0.009501188 |
| OR10H4 | 4 | 0.009501188 |
| H3C11 | 4 | 0.009501188 |
| PNMA1 | 4 | 0.009501188 |
| GGT1 | 4 | 0.009501188 |
| ESD | 4 | 0.009501188 |
| H1-5 | 4 | 0.009501188 |
| GZMB | 4 | 0.009501188 |
| GCNT3 | 4 | 0.009501188 |
| OR52N2 | 4 | 0.009501188 |
| TMEM115 | 4 | 0.009501188 |
| SLC25A16 | 4 | 0.009501188 |
| ABHD13 | 4 | 0.009501188 |
| GTF2H3 | 4 | 0.009501188 |
| NPIPB15 | 4 | 0.009501188 |
| MRPL58 | 4 | 0.009501188 |
| NTMT1 | 4 | 0.009501188 |
| MS4A10 | 4 | 0.009501188 |
| TMPRSS11E | 4 | 0.009501188 |
| EDN1 | 4 | 0.009501188 |
| ARMCX5-GPRASP2 | 4 | 0.009501188 |
| PSG5 | 4 | 0.009501188 |
| AL031777.2 | 4 | 0.009501188 |
| DFFB | 4 | 0.009501188 |
| GPRC5B | 4 | 0.009501188 |
| COPS4 | 4 | 0.009501188 |
| CCDC97 | 4 | 0.009501188 |
| PDP2 | 4 | 0.009501188 |
| C1QC | 4 | 0.009501188 |
| GLIS2 | 4 | 0.009501188 |
| POTEM | 4 | 0.009501188 |
| TMEM97 | 4 | 0.009501188 |
| POGLUT3 | 4 | 0.009501188 |
| CLEC18C | 4 | 0.009501188 |
| RPL4 | 4 | 0.009501188 |
| APOL6 | 4 | 0.009501188 |
| CYTH1 | 4 | 0.009501188 |
| RPS23 | 4 | 0.009501188 |
| ARX | 4 | 0.009501188 |
| PISD | 4 | 0.009501188 |
| METTL21C | 4 | 0.009501188 |
| SLC5A1 | 4 | 0.009501188 |
| COTL1 | 4 | 0.009501188 |
| MCM2 | 4 | 0.009501188 |
| IFNL3 | 4 | 0.009501188 |
| RALY | 4 | 0.009501188 |
| CANX | 4 | 0.009501188 |
| PRDX2 | 4 | 0.009501188 |
| CLECL1 | 4 | 0.009501188 |
| SIRT3 | 4 | 0.009501188 |
| NEK7 | 4 | 0.009501188 |
| IRF2BP2 | 4 | 0.009501188 |
| CD46 | 4 | 0.009501188 |
| AGER | 4 | 0.009501188 |
| JAZF1 | 4 | 0.009501188 |
| IGHV3-11 | 4 | 0.009501188 |
| C11orf68 | 4 | 0.009501188 |
| DNAJB11 | 4 | 0.009501188 |
| STING1 | 4 | 0.009501188 |
| DRICH1 | 4 | 0.009501188 |
| RAB35 | 4 | 0.009501188 |
| COL10A1 | 4 | 0.009501188 |
| BCS1L | 4 | 0.009501188 |
| FKBP5 | 4 | 0.009501188 |
| TRAPPC6A | 4 | 0.009501188 |
| DNAJC27 | 4 | 0.009501188 |
| EIF4H | 4 | 0.009501188 |
| BAIAP2L2 | 4 | 0.009501188 |
| PRR19 | 4 | 0.009501188 |
| PCYOX1L | 4 | 0.009501188 |
| PSMD3 | 4 | 0.009501188 |
| RUNDC3A | 4 | 0.009501188 |
| SDHB | 4 | 0.009501188 |
| SLC25A41 | 4 | 0.009501188 |
| DOK2 | 4 | 0.009501188 |
| SETD4 | 4 | 0.009501188 |
| C11orf1 | 4 | 0.009501188 |
| CHMP6 | 4 | 0.009501188 |
| GDPGP1 | 4 | 0.009501188 |
| GLCCI1 | 4 | 0.009501188 |
| RHOXF2 | 4 | 0.009501188 |
| UFSP2 | 4 | 0.009501188 |
| CTAGE6 | 4 | 0.009501188 |
| PRADC1 | 4 | 0.009501188 |
| HIBADH | 4 | 0.009501188 |
| IRF8 | 4 | 0.009501188 |
| PEX13 | 4 | 0.009501188 |
| MRM3 | 4 | 0.009501188 |
| OR51I1 | 4 | 0.009501188 |
| MROH6 | 4 | 0.009501188 |
| UGT1A5 | 4 | 0.009501188 |
| SRRM3 | 4 | 0.009501188 |
| GGPS1 | 4 | 0.009501188 |
| GAS2 | 4 | 0.009501188 |
| POPDC2 | 4 | 0.009501188 |
| PPTC7 | 4 | 0.009501188 |
| APEX1 | 4 | 0.009501188 |
| PPP6R1 | 4 | 0.009501188 |
| ACTC1 | 4 | 0.009501188 |
| RBM44 | 4 | 0.009501188 |
| RTP2 | 4 | 0.009501188 |
| TNNI3 | 4 | 0.009501188 |
| CD320 | 4 | 0.009501188 |
| OR7A17 | 4 | 0.009501188 |
| FAM110D | 4 | 0.009501188 |
| MTERF1 | 4 | 0.009501188 |
| FABP6 | 4 | 0.009501188 |
| RNF175 | 4 | 0.009501188 |
| TSN | 4 | 0.009501188 |
| RHBDD3 | 4 | 0.009501188 |
| LHFPL5 | 4 | 0.009501188 |
| MEGF11 | 4 | 0.009501188 |
| RSPH1 | 4 | 0.009501188 |
| RLN2 | 4 | 0.009501188 |
| ZKSCAN3 | 4 | 0.009501188 |
| METTL24 | 4 | 0.009501188 |
| REG1B | 4 | 0.009501188 |
| FBXL16 | 4 | 0.009501188 |
| IGHV3-23 | 4 | 0.009501188 |
| ACCS | 4 | 0.009501188 |
| FCAR | 4 | 0.009501188 |
| FOXD4 | 4 | 0.009501188 |
| C1orf74 | 4 | 0.009501188 |
| CWC25 | 4 | 0.009501188 |
| TERF1 | 4 | 0.009501188 |
| TPM1 | 4 | 0.009501188 |
| SDCCAG8 | 4 | 0.009501188 |
| ARRDC2 | 4 | 0.009501188 |
| C1QA | 4 | 0.009501188 |
| KLF12 | 4 | 0.009501188 |
| HSD17B6 | 4 | 0.009501188 |
| NEURL1 | 4 | 0.009501188 |
| COPE | 4 | 0.009501188 |
| PUS1 | 4 | 0.009501188 |
| MRPS11 | 4 | 0.009501188 |
| BPIFB3 | 4 | 0.009501188 |
| RBPJ | 4 | 0.009501188 |
| UNC93B1 | 4 | 0.009501188 |
| CNTF | 4 | 0.009501188 |
| RPL18A | 4 | 0.009501188 |
| HNRNPH3 | 4 | 0.009501188 |
| HDAC8 | 4 | 0.009501188 |
| NTN3 | 4 | 0.009501188 |
| PPP3CC | 4 | 0.009501188 |
| M1AP | 4 | 0.009501188 |
| ALOX15 | 4 | 0.009501188 |
| GABPB1 | 4 | 0.009501188 |
| MAPK3 | 4 | 0.009501188 |
| FAM151B | 4 | 0.009501188 |
| TIGD5 | 4 | 0.009501188 |
| ADORA3 | 4 | 0.009501188 |
| PSMB8 | 4 | 0.009501188 |
| CSF3 | 4 | 0.009501188 |
| SP7 | 4 | 0.009501188 |
| NDUFB9 | 4 | 0.009501188 |
| TNNT3 | 4 | 0.009501188 |
| APOBEC2 | 4 | 0.009501188 |
| TNFRSF10C | 4 | 0.009501188 |
| KLF7 | 4 | 0.009501188 |
| STARD10 | 4 | 0.009501188 |
| MTURN | 4 | 0.009501188 |
| SMAP2 | 4 | 0.009501188 |
| MCM8 | 4 | 0.009501188 |
| TFCP2 | 4 | 0.009501188 |
| SLC39A13 | 4 | 0.009501188 |
| PPP1R14A | 4 | 0.009501188 |
| FRMD1 | 4 | 0.009501188 |
| XKR9 | 4 | 0.009501188 |
| METTL14 | 4 | 0.009501188 |
| PFKFB4 | 4 | 0.009501188 |
| FAM167B | 4 | 0.009501188 |
| HNRNPLL | 4 | 0.009501188 |
| C1orf56 | 4 | 0.009501188 |
| PLBD2 | 4 | 0.009501188 |
| SEPSECS | 4 | 0.009501188 |
| EEF2KMT | 4 | 0.009501188 |
| COX18 | 4 | 0.009501188 |
| ZNF202 | 4 | 0.009501188 |
| FKRP | 4 | 0.009501188 |
| C7orf26 | 4 | 0.009501188 |
| PSAT1 | 4 | 0.009501188 |
| GOT2 | 4 | 0.009501188 |
| KRTAP19-3 | 4 | 0.009501188 |
| MTERF2 | 4 | 0.009501188 |
| HOXC9 | 4 | 0.009501188 |
| BCL7C | 4 | 0.009501188 |
| FCGR1A | 4 | 0.009501188 |
| SRPX2 | 4 | 0.009501188 |
| NCBP2L | 4 | 0.009501188 |
| CCDC183 | 4 | 0.009501188 |
| MIOX | 4 | 0.009501188 |
| OR2A2 | 4 | 0.009501188 |
| PPM1K | 4 | 0.009501188 |
| C11orf24 | 4 | 0.009501188 |
| PITX1 | 4 | 0.009501188 |
| STMN4 | 4 | 0.009501188 |
| SERPING1 | 4 | 0.009501188 |
| CEACAM19 | 4 | 0.009501188 |
| ARCN1 | 4 | 0.009501188 |
| H4C8 | 4 | 0.009501188 |
| SMR3A | 4 | 0.009501188 |
| DEGS2 | 4 | 0.009501188 |
| SHARPIN | 4 | 0.009501188 |
| TBCEL | 4 | 0.009501188 |
| AKR1C4 | 4 | 0.009501188 |
| RP2 | 4 | 0.009501188 |
| GNAI1 | 4 | 0.009501188 |
| SERPINA1 | 4 | 0.009501188 |
| PLSCR2 | 4 | 0.009501188 |
| LURAP1L | 4 | 0.009501188 |
| S100A8 | 4 | 0.009501188 |
| OSGEP | 4 | 0.009501188 |
| MOGAT1 | 4 | 0.009501188 |
| P2RY12 | 4 | 0.009501188 |
| FEZ2 | 4 | 0.009501188 |
| MRPL47 | 4 | 0.009501188 |
| FFAR2 | 4 | 0.009501188 |
| ITM2A | 4 | 0.009501188 |
| TMEM100 | 4 | 0.009501188 |
| NFKBID | 4 | 0.009501188 |
| UQCRFS1 | 4 | 0.009501188 |
| CD1D | 4 | 0.009501188 |
| CPNE1 | 4 | 0.009501188 |
| CSN3 | 4 | 0.009501188 |
| PPP4C | 4 | 0.009501188 |
| MADCAM1 | 4 | 0.009501188 |
| PRB3 | 4 | 0.009501188 |
| NDUFAF7 | 4 | 0.009501188 |
| MRPL1 | 4 | 0.009501188 |
| TRMT61B | 4 | 0.009501188 |
| CDK5RAP3 | 4 | 0.009501188 |
| SLC6A8 | 4 | 0.009501188 |
| BTD | 4 | 0.009501188 |
| ELOVL3 | 4 | 0.009501188 |
| ABHD6 | 4 | 0.009501188 |
| FGF2 | 4 | 0.009501188 |
| XKRX | 4 | 0.009501188 |
| IFI6 | 4 | 0.009501188 |
| MIF4GD | 4 | 0.009501188 |
| FBXO48 | 4 | 0.009501188 |
| TRIP10 | 4 | 0.009501188 |
| APH1B | 4 | 0.009501188 |
| PEX14 | 4 | 0.009501188 |
| TAF13 | 4 | 0.009501188 |
| DRD3 | 4 | 0.009501188 |
| NUDC | 4 | 0.009501188 |
| GFOD1 | 4 | 0.009501188 |
| TAS2R4 | 4 | 0.009501188 |
| SPDYE3 | 4 | 0.009501188 |
| EVA1A | 4 | 0.009501188 |
| VPS4B | 4 | 0.009501188 |
| SMYD3 | 4 | 0.009501188 |
| SLC25A36 | 4 | 0.009501188 |
| PRM3 | 4 | 0.009501188 |
| GABPB2 | 4 | 0.009501188 |
| SEPTIN8 | 4 | 0.009501188 |
| FAM126B | 4 | 0.009501188 |
| OR11H4 | 4 | 0.009501188 |
| P3H4 | 4 | 0.009501188 |
| C11orf49 | 4 | 0.009501188 |
| NME7 | 4 | 0.009501188 |
| GH2 | 4 | 0.009501188 |
| SULT1C2 | 4 | 0.009501188 |
| DONSON | 4 | 0.009501188 |
| LACTB | 4 | 0.009501188 |
| CALU | 4 | 0.009501188 |
| SNRPA | 4 | 0.009501188 |
| KMT5A | 4 | 0.009501188 |
| RAB6A | 4 | 0.009501188 |
| UTP11 | 4 | 0.009501188 |
| AC018630.4 | 4 | 0.009501188 |
| NDRG1 | 4 | 0.009501188 |
| B9D2 | 4 | 0.009501188 |
| PGAP3 | 4 | 0.009501188 |
| CNMD | 4 | 0.009501188 |
| YTHDF1 | 4 | 0.009501188 |
| OR4D9 | 4 | 0.009501188 |
| DEPDC1B | 4 | 0.009501188 |
| RSPO1 | 4 | 0.009501188 |
| DQX1 | 4 | 0.009501188 |
| SPDYA | 4 | 0.009501188 |
| SPOP | 4 | 0.009501188 |
| CPA6 | 4 | 0.009501188 |
| MBLAC2 | 3 | 0.007125891 |
| CBX7 | 3 | 0.007125891 |
| CHMP3 | 3 | 0.007125891 |
| GNPDA2 | 3 | 0.007125891 |
| TNFSF18 | 3 | 0.007125891 |
| LIPN | 3 | 0.007125891 |
| KLF2 | 3 | 0.007125891 |
| RHO | 3 | 0.007125891 |
| IGLV5-45 | 3 | 0.007125891 |
| PRR15L | 3 | 0.007125891 |
| NTPCR | 3 | 0.007125891 |
| PSMB2 | 3 | 0.007125891 |
| P4HTM | 3 | 0.007125891 |
| FN3KRP | 3 | 0.007125891 |
| ZNF732 | 3 | 0.007125891 |
| ARL15 | 3 | 0.007125891 |
| HSCB | 3 | 0.007125891 |
| ENHO | 3 | 0.007125891 |
| C2orf69 | 3 | 0.007125891 |
| CKMT1B | 3 | 0.007125891 |
| IL15RA | 3 | 0.007125891 |
| IL22 | 3 | 0.007125891 |
| PQBP1 | 3 | 0.007125891 |
| RNASEH2B | 3 | 0.007125891 |
| KDM8 | 3 | 0.007125891 |
| SFRP1 | 3 | 0.007125891 |
| KREMEN2 | 3 | 0.007125891 |
| DNAL4 | 3 | 0.007125891 |
| ZNF239 | 3 | 0.007125891 |
| SLC39A2 | 3 | 0.007125891 |
| CARHSP1 | 3 | 0.007125891 |
| KRTAP26-1 | 3 | 0.007125891 |
| BOLL | 3 | 0.007125891 |
| ZNF146 | 3 | 0.007125891 |
| SLC2A12 | 3 | 0.007125891 |
| TATDN3 | 3 | 0.007125891 |
| HOXB13 | 3 | 0.007125891 |
| FOXE3 | 3 | 0.007125891 |
| SLC7A6 | 3 | 0.007125891 |
| EMP3 | 3 | 0.007125891 |
| GNA13 | 3 | 0.007125891 |
| DCTN3 | 3 | 0.007125891 |
| RIMKLA | 3 | 0.007125891 |
| CARD16 | 3 | 0.007125891 |
| VCPKMT | 3 | 0.007125891 |
| HAND1 | 3 | 0.007125891 |
| METTL9 | 3 | 0.007125891 |
| TBX21 | 3 | 0.007125891 |
| YIPF5 | 3 | 0.007125891 |
| OXGR1 | 3 | 0.007125891 |
| ZFAND3 | 3 | 0.007125891 |
| DUOXA1 | 3 | 0.007125891 |
| ANXA3 | 3 | 0.007125891 |
| NRARP | 3 | 0.007125891 |
| CTXN3 | 3 | 0.007125891 |
| HCRTR1 | 3 | 0.007125891 |
| FAM136A | 3 | 0.007125891 |
| ZDHHC19 | 3 | 0.007125891 |
| TUBB1 | 3 | 0.007125891 |
| AC097637.1 | 3 | 0.007125891 |
| C1QL1 | 3 | 0.007125891 |
| HSD17B12 | 3 | 0.007125891 |
| SDHAF2 | 3 | 0.007125891 |
| UPK3A | 3 | 0.007125891 |
| SCAMP3 | 3 | 0.007125891 |
| TAPBPL | 3 | 0.007125891 |
| CASP9 | 3 | 0.007125891 |
| CCDC149 | 3 | 0.007125891 |
| ETFRF1 | 3 | 0.007125891 |
| TBRG1 | 3 | 0.007125891 |
| ABHD5 | 3 | 0.007125891 |
| ZNF396 | 3 | 0.007125891 |
| HUS1 | 3 | 0.007125891 |
| SCRT1 | 3 | 0.007125891 |
| PIGZ | 3 | 0.007125891 |
| C2orf15 | 3 | 0.007125891 |
| CD33 | 3 | 0.007125891 |
| TOM1 | 3 | 0.007125891 |
| ARGLU1 | 3 | 0.007125891 |
| OR10G2 | 3 | 0.007125891 |
| SPATA6 | 3 | 0.007125891 |
| TMED2 | 3 | 0.007125891 |
| RTL8C | 3 | 0.007125891 |
| CDC37L1 | 3 | 0.007125891 |
| MYBPHL | 3 | 0.007125891 |
| RACGAP1 | 3 | 0.007125891 |
| PGA5 | 3 | 0.007125891 |
| PFN2 | 3 | 0.007125891 |
| MRPS5 | 3 | 0.007125891 |
| DTX1 | 3 | 0.007125891 |
| KCTD6 | 3 | 0.007125891 |
| SLC33A1 | 3 | 0.007125891 |
| ESAM | 3 | 0.007125891 |
| TACSTD2 | 3 | 0.007125891 |
| PRKAR1A | 3 | 0.007125891 |
| TBC1D28 | 3 | 0.007125891 |
| SART1 | 3 | 0.007125891 |
| SCNM1 | 3 | 0.007125891 |
| KRTAP19-1 | 3 | 0.007125891 |
| TREX2 | 3 | 0.007125891 |
| FAM24A | 3 | 0.007125891 |
| POU4F1 | 3 | 0.007125891 |
| MRPL15 | 3 | 0.007125891 |
| PPM1F | 3 | 0.007125891 |
| UBTD2 | 3 | 0.007125891 |
| ATP6V1B2 | 3 | 0.007125891 |
| KRT24 | 3 | 0.007125891 |
| LRP11 | 3 | 0.007125891 |
| PARD6B | 3 | 0.007125891 |
| RGPD4 | 3 | 0.007125891 |
| UIMC1 | 3 | 0.007125891 |
| IST1 | 3 | 0.007125891 |
| TSEN34 | 3 | 0.007125891 |
| AC136428.1 | 3 | 0.007125891 |
| OTUB2 | 3 | 0.007125891 |
| KRTAP4-1 | 3 | 0.007125891 |
| TOPAZ1 | 3 | 0.007125891 |
| SSU72P8 | 3 | 0.007125891 |
| BUD13 | 3 | 0.007125891 |
| FBXO2 | 3 | 0.007125891 |
| PLXDC1 | 3 | 0.007125891 |
| RBIS | 3 | 0.007125891 |
| RAB1B | 3 | 0.007125891 |
| SORBS3 | 3 | 0.007125891 |
| PBK | 3 | 0.007125891 |
| ORAI3 | 3 | 0.007125891 |
| CHRNA10 | 3 | 0.007125891 |
| COX19 | 3 | 0.007125891 |
| MRO | 3 | 0.007125891 |
| OR7G3 | 3 | 0.007125891 |
| KRTAP5-1 | 3 | 0.007125891 |
| ABCF2 | 3 | 0.007125891 |
| HOATZ | 3 | 0.007125891 |
| S1PR2 | 3 | 0.007125891 |
| RER1 | 3 | 0.007125891 |
| PGAM5 | 3 | 0.007125891 |
| GPT2 | 3 | 0.007125891 |
| TRIB3 | 3 | 0.007125891 |
| CRLF1 | 3 | 0.007125891 |
| FAM207A | 3 | 0.007125891 |
| PSMA4 | 3 | 0.007125891 |
| SHROOM1 | 3 | 0.007125891 |
| STAP1 | 3 | 0.007125891 |
| DCAF10 | 3 | 0.007125891 |
| CHKB | 3 | 0.007125891 |
| RBP4 | 3 | 0.007125891 |
| ARL6IP5 | 3 | 0.007125891 |
| TMEM174 | 3 | 0.007125891 |
| COX4I1 | 3 | 0.007125891 |
| CIDEA | 3 | 0.007125891 |
| CD1C | 3 | 0.007125891 |
| PRKACB | 3 | 0.007125891 |
| GALK1 | 3 | 0.007125891 |
| UCP3 | 3 | 0.007125891 |
| TAS2R60 | 3 | 0.007125891 |
| RNASE4 | 3 | 0.007125891 |
| SLPI | 3 | 0.007125891 |
| CYTH2 | 3 | 0.007125891 |
| C9orf152 | 3 | 0.007125891 |
| WDR89 | 3 | 0.007125891 |
| GRINA | 3 | 0.007125891 |
| ALG14 | 3 | 0.007125891 |
| PLIN1 | 3 | 0.007125891 |
| ADAP1 | 3 | 0.007125891 |
| GGCT | 3 | 0.007125891 |
| RGPD3 | 3 | 0.007125891 |
| HIBCH | 3 | 0.007125891 |
| ANXA5 | 3 | 0.007125891 |
| GNPDA1 | 3 | 0.007125891 |
| CNPY3 | 3 | 0.007125891 |
| INSL3 | 3 | 0.007125891 |
| CCR6 | 3 | 0.007125891 |
| RHOU | 3 | 0.007125891 |
| GAL3ST3 | 3 | 0.007125891 |
| UBA52 | 3 | 0.007125891 |
| ATP6V1G1 | 3 | 0.007125891 |
| C19orf54 | 3 | 0.007125891 |
| RAD51D | 3 | 0.007125891 |
| OR8D1 | 3 | 0.007125891 |
| TAS2R16 | 3 | 0.007125891 |
| CPN1 | 3 | 0.007125891 |
| PTGR1 | 3 | 0.007125891 |
| LRP8 | 3 | 0.007125891 |
| SOX8 | 3 | 0.007125891 |
| ZFP62 | 3 | 0.007125891 |
| HTR1D | 3 | 0.007125891 |
| ROPN1L | 3 | 0.007125891 |
| KCNE1 | 3 | 0.007125891 |
| OPTC | 3 | 0.007125891 |
| GPR107 | 3 | 0.007125891 |
| CHCHD6 | 3 | 0.007125891 |
| GSTT2B | 3 | 0.007125891 |
| IGHV2-70 | 3 | 0.007125891 |
| FBXW9 | 3 | 0.007125891 |
| SLC22A18 | 3 | 0.007125891 |
| FCER1A | 3 | 0.007125891 |
| COQ6 | 3 | 0.007125891 |
| PSMA5 | 3 | 0.007125891 |
| CCNY | 3 | 0.007125891 |
| IDUA | 3 | 0.007125891 |
| LYSMD4 | 3 | 0.007125891 |
| ARPC2 | 3 | 0.007125891 |
| C8orf58 | 3 | 0.007125891 |
| IL20RB | 3 | 0.007125891 |
| CFAP36 | 3 | 0.007125891 |
| TPD52 | 3 | 0.007125891 |
| PLPPR3 | 3 | 0.007125891 |
| IMPA1 | 3 | 0.007125891 |
| SERTAD3 | 3 | 0.007125891 |
| TPRA1 | 3 | 0.007125891 |
| TRMT2A | 3 | 0.007125891 |
| PSMD13 | 3 | 0.007125891 |
| JOSD1 | 3 | 0.007125891 |
| VPS26B | 3 | 0.007125891 |
| AIPL1 | 3 | 0.007125891 |
| C1orf131 | 3 | 0.007125891 |
| AUP1 | 3 | 0.007125891 |
| CD99L2 | 3 | 0.007125891 |
| RMND5A | 3 | 0.007125891 |
| NUDT6 | 3 | 0.007125891 |
| COA6 | 3 | 0.007125891 |
| FANCD2OS | 3 | 0.007125891 |
| PF4 | 3 | 0.007125891 |
| IRF9 | 3 | 0.007125891 |
| LINC02210-CRHR1 | 3 | 0.007125891 |
| CCL16 | 3 | 0.007125891 |
| CCN2 | 3 | 0.007125891 |
| AKAP1 | 3 | 0.007125891 |
| TMEM39B | 3 | 0.007125891 |
| SGTB | 3 | 0.007125891 |
| CNNM3 | 3 | 0.007125891 |
| C5orf15 | 3 | 0.007125891 |
| SMAP1 | 3 | 0.007125891 |
| CYSLTR2 | 3 | 0.007125891 |
| TMEM87A | 3 | 0.007125891 |
| ST13 | 3 | 0.007125891 |
| RPS6KL1 | 3 | 0.007125891 |
| OR52E6 | 3 | 0.007125891 |
| RTRAF | 3 | 0.007125891 |
| LANCL3 | 3 | 0.007125891 |
| ZNF79 | 3 | 0.007125891 |
| APOH | 3 | 0.007125891 |
| CKS1B | 3 | 0.007125891 |
| TMEM205 | 3 | 0.007125891 |
| C1QTNF12 | 3 | 0.007125891 |
| LMO4 | 3 | 0.007125891 |
| PCP2 | 3 | 0.007125891 |
| SRP54 | 3 | 0.007125891 |
| APOL4 | 3 | 0.007125891 |
| CAPN13 | 3 | 0.007125891 |
| KLHL29 | 3 | 0.007125891 |
| CALHM2 | 3 | 0.007125891 |
| ELOVL7 | 3 | 0.007125891 |
| H2BC3 | 3 | 0.007125891 |
| ZDHHC6 | 3 | 0.007125891 |
| C1orf52 | 3 | 0.007125891 |
| ZBTB47 | 3 | 0.007125891 |
| KLF11 | 3 | 0.007125891 |
| LAMTOR1 | 3 | 0.007125891 |
| ETNK2 | 3 | 0.007125891 |
| TRAPPC2 | 3 | 0.007125891 |
| TERF2 | 3 | 0.007125891 |
| UBXN10 | 3 | 0.007125891 |
| RSPH9 | 3 | 0.007125891 |
| MRPL50 | 3 | 0.007125891 |
| ATG3 | 3 | 0.007125891 |
| FOXA3 | 3 | 0.007125891 |
| MAGEB5 | 3 | 0.007125891 |
| SYCP3 | 3 | 0.007125891 |
| C12orf54 | 3 | 0.007125891 |
| RNF26 | 3 | 0.007125891 |
| NDUFB3 | 3 | 0.007125891 |
| OR10A5 | 3 | 0.007125891 |
| ADPRH | 3 | 0.007125891 |
| MUC1 | 3 | 0.007125891 |
| PMP2 | 3 | 0.007125891 |
| LYG2 | 3 | 0.007125891 |
| TMEM255B | 3 | 0.007125891 |
| PXYLP1 | 3 | 0.007125891 |
| ADPRM | 3 | 0.007125891 |
| IVD | 3 | 0.007125891 |
| GJB7 | 3 | 0.007125891 |
| SHISA2 | 3 | 0.007125891 |
| PTCRA | 3 | 0.007125891 |
| NPM2 | 3 | 0.007125891 |
| WBP2 | 3 | 0.007125891 |
| GPI | 3 | 0.007125891 |
| SDHAF3 | 3 | 0.007125891 |
| CD300C | 3 | 0.007125891 |
| CABLES2 | 3 | 0.007125891 |
| OCLN | 3 | 0.007125891 |
| ELMOD2 | 3 | 0.007125891 |
| CLDN19 | 3 | 0.007125891 |
| RPF2 | 3 | 0.007125891 |
| LDHD | 3 | 0.007125891 |
| ERMAP | 3 | 0.007125891 |
| CSNK1A1 | 3 | 0.007125891 |
| QPCT | 3 | 0.007125891 |
| PHOSPHO1 | 3 | 0.007125891 |
| RASL12 | 3 | 0.007125891 |
| TRIM77 | 3 | 0.007125891 |
| NPPA | 3 | 0.007125891 |
| C8orf33 | 3 | 0.007125891 |
| PLAA | 3 | 0.007125891 |
| S100A2 | 3 | 0.007125891 |
| RAB11FIP3 | 3 | 0.007125891 |
| ZMYND12 | 3 | 0.007125891 |
| IRF3 | 3 | 0.007125891 |
| CEACAM3 | 3 | 0.007125891 |
| LSMEM1 | 3 | 0.007125891 |
| SULT1A2 | 3 | 0.007125891 |
| NRL | 3 | 0.007125891 |
| OR52A1 | 3 | 0.007125891 |
| PACRGL | 3 | 0.007125891 |
| STX16 | 3 | 0.007125891 |
| ASF1B | 3 | 0.007125891 |
| KRT8 | 3 | 0.007125891 |
| OMP | 3 | 0.007125891 |
| MAPK13 | 3 | 0.007125891 |
| CTTN | 3 | 0.007125891 |
| CASP14 | 3 | 0.007125891 |
| TMEM101 | 3 | 0.007125891 |
| MC2R | 3 | 0.007125891 |
| GRPEL1 | 3 | 0.007125891 |
| MITD1 | 3 | 0.007125891 |
| FBXL15 | 3 | 0.007125891 |
| DLX3 | 3 | 0.007125891 |
| YOD1 | 3 | 0.007125891 |
| ZNF260 | 3 | 0.007125891 |
| GLS | 3 | 0.007125891 |
| ZNF343 | 3 | 0.007125891 |
| LRRC19 | 3 | 0.007125891 |
| BMI1 | 3 | 0.007125891 |
| CENPO | 3 | 0.007125891 |
| COA1 | 3 | 0.007125891 |
| METTL21A | 3 | 0.007125891 |
| UBAP1 | 3 | 0.007125891 |
| IGKV3D-11 | 3 | 0.007125891 |
| TAF9B | 3 | 0.007125891 |
| SLC25A40 | 3 | 0.007125891 |
| SNU13 | 3 | 0.007125891 |
| DPPA4 | 3 | 0.007125891 |
| ATP1B1 | 3 | 0.007125891 |
| ZNF263 | 3 | 0.007125891 |
| PRSS16 | 3 | 0.007125891 |
| C4orf17 | 3 | 0.007125891 |
| RETN | 3 | 0.007125891 |
| NOA1 | 3 | 0.007125891 |
| STPG1 | 3 | 0.007125891 |
| LYPLA2 | 3 | 0.007125891 |
| ATP5PB | 3 | 0.007125891 |
| TCEAL9 | 3 | 0.007125891 |
| LCE2D | 3 | 0.007125891 |
| WNT5B | 3 | 0.007125891 |
| HTATIP2 | 3 | 0.007125891 |
| NCBP3 | 3 | 0.007125891 |
| RIOK3 | 3 | 0.007125891 |
| SOWAHB | 3 | 0.007125891 |
| OR7D4 | 3 | 0.007125891 |
| IGFBP2 | 3 | 0.007125891 |
| STAR | 3 | 0.007125891 |
| TRIM11 | 3 | 0.007125891 |
| FXN | 3 | 0.007125891 |
| TM7SF2 | 3 | 0.007125891 |
| HACD1 | 3 | 0.007125891 |
| IFI27L2 | 3 | 0.007125891 |
| UCKL1 | 3 | 0.007125891 |
| SDE2 | 3 | 0.007125891 |
| GCSAML | 3 | 0.007125891 |
| ARMC1 | 3 | 0.007125891 |
| C7orf61 | 3 | 0.007125891 |
| UBXN2A | 3 | 0.007125891 |
| PILRA | 3 | 0.007125891 |
| TAS2R43 | 3 | 0.007125891 |
| PBX4 | 3 | 0.007125891 |
| NOL11 | 3 | 0.007125891 |
| EIF4E3 | 3 | 0.007125891 |
| TRIM28 | 3 | 0.007125891 |
| RAB23 | 3 | 0.007125891 |
| EFCAB8 | 3 | 0.007125891 |
| APOBEC3H | 3 | 0.007125891 |
| LOXHD1 | 3 | 0.007125891 |
| TPD52L2 | 3 | 0.007125891 |
| CLRN2 | 3 | 0.007125891 |
| H4C9 | 3 | 0.007125891 |
| DEFB106B | 3 | 0.007125891 |
| SLC22A25 | 3 | 0.007125891 |
| CCDC184 | 3 | 0.007125891 |
| TEX55 | 3 | 0.007125891 |
| CRHR1 | 3 | 0.007125891 |
| CD53 | 3 | 0.007125891 |
| FST | 3 | 0.007125891 |
| PRSS48 | 3 | 0.007125891 |
| OR9I1 | 3 | 0.007125891 |
| PAAF1 | 3 | 0.007125891 |
| F3 | 3 | 0.007125891 |
| EIF2B1 | 3 | 0.007125891 |
| TOR3A | 3 | 0.007125891 |
| PDZD11 | 3 | 0.007125891 |
| MYB | 3 | 0.007125891 |
| SH2D4A | 3 | 0.007125891 |
| RPSA | 3 | 0.007125891 |
| VAPA | 3 | 0.007125891 |
| KRTAP12-3 | 3 | 0.007125891 |
| MEX3D | 3 | 0.007125891 |
| FKBP11 | 3 | 0.007125891 |
| C2CD2L | 3 | 0.007125891 |
| APOBEC3F | 3 | 0.007125891 |
| C1orf194 | 3 | 0.007125891 |
| FLVCR1 | 3 | 0.007125891 |
| CBLN1 | 3 | 0.007125891 |
| GPR148 | 3 | 0.007125891 |
| UCK2 | 3 | 0.007125891 |
| C1orf146 | 3 | 0.007125891 |
| H1-6 | 3 | 0.007125891 |
| RBFA | 3 | 0.007125891 |
| CLDN4 | 3 | 0.007125891 |
| MACROD1 | 3 | 0.007125891 |
| MANSC1 | 3 | 0.007125891 |
| TRIM44 | 3 | 0.007125891 |
| SIM2 | 3 | 0.007125891 |
| GUCA1A | 3 | 0.007125891 |
| TCP11L2 | 3 | 0.007125891 |
| GCLM | 3 | 0.007125891 |
| CRYGC | 3 | 0.007125891 |
| ZNF703 | 3 | 0.007125891 |
| OR2AG2 | 3 | 0.007125891 |
| SIRT6 | 3 | 0.007125891 |
| FANCF | 3 | 0.007125891 |
| ANKS4B | 3 | 0.007125891 |
| GRP | 3 | 0.007125891 |
| TMEM18 | 3 | 0.007125891 |
| NAP1L4 | 3 | 0.007125891 |
| LSMEM2 | 3 | 0.007125891 |
| CTDSPL2 | 3 | 0.007125891 |
| VRK3 | 3 | 0.007125891 |
| PLPPR2 | 3 | 0.007125891 |
| KPNA5 | 3 | 0.007125891 |
| RIPPLY2 | 3 | 0.007125891 |
| PRAMEF10 | 3 | 0.007125891 |
| TTC5 | 3 | 0.007125891 |
| CTSE | 3 | 0.007125891 |
| SLC10A7 | 3 | 0.007125891 |
| KLRG1 | 3 | 0.007125891 |
| CD151 | 3 | 0.007125891 |
| TOMM40L | 3 | 0.007125891 |
| PRSS58 | 3 | 0.007125891 |
| TRMT11 | 3 | 0.007125891 |
| RIBC1 | 3 | 0.007125891 |
| GPR18 | 3 | 0.007125891 |
| NEUROG2 | 3 | 0.007125891 |
| PLBD1 | 3 | 0.007125891 |
| NIPA1 | 3 | 0.007125891 |
| ITFG2 | 3 | 0.007125891 |
| GJC2 | 3 | 0.007125891 |
| CST7 | 3 | 0.007125891 |
| KLRC3 | 3 | 0.007125891 |
| NIPSNAP1 | 3 | 0.007125891 |
| GRXCR2 | 3 | 0.007125891 |
| ABHD17C | 3 | 0.007125891 |
| IGHV3-53 | 3 | 0.007125891 |
| GDI1 | 3 | 0.007125891 |
| PLP2 | 3 | 0.007125891 |
| PDLIM4 | 3 | 0.007125891 |
| ABHD15 | 3 | 0.007125891 |
| COMMD10 | 3 | 0.007125891 |
| BCDIN3D | 3 | 0.007125891 |
| LELP1 | 3 | 0.007125891 |
| CLDN9 | 3 | 0.007125891 |
| GABARAPL1 | 3 | 0.007125891 |
| IGKV1D-16 | 3 | 0.007125891 |
| TDP1 | 3 | 0.007125891 |
| RANBP1 | 3 | 0.007125891 |
| C3orf62 | 3 | 0.007125891 |
| GPR89B | 3 | 0.007125891 |
| TMEM150A | 3 | 0.007125891 |
| SNAP25 | 3 | 0.007125891 |
| LIPK | 3 | 0.007125891 |
| OR1S1 | 3 | 0.007125891 |
| PDPN | 3 | 0.007125891 |
| TNFRSF25 | 3 | 0.007125891 |
| HSDL1 | 3 | 0.007125891 |
| MIS18A | 3 | 0.007125891 |
| STN1 | 3 | 0.007125891 |
| MBL2 | 3 | 0.007125891 |
| GPR135 | 3 | 0.007125891 |
| FAM168A | 3 | 0.007125891 |
| RFFL | 3 | 0.007125891 |
| HIF1AN | 3 | 0.007125891 |
| TST | 3 | 0.007125891 |
| UHMK1 | 3 | 0.007125891 |
| CPSF3 | 3 | 0.007125891 |
| AC134980.2 | 3 | 0.007125891 |
| PRRT1 | 3 | 0.007125891 |
| NOB1 | 3 | 0.007125891 |
| DNAJC25 | 3 | 0.007125891 |
| CTRC | 3 | 0.007125891 |
| YIF1B | 3 | 0.007125891 |
| CPNE5 | 3 | 0.007125891 |
| GKN1 | 3 | 0.007125891 |
| MUC7 | 3 | 0.007125891 |
| IPP | 3 | 0.007125891 |
| NDRG4 | 3 | 0.007125891 |
| GJB4 | 3 | 0.007125891 |
| OR5A1 | 3 | 0.007125891 |
| FAM228A | 3 | 0.007125891 |
| LTA4H | 3 | 0.007125891 |
| PICK1 | 3 | 0.007125891 |
| YBEY | 3 | 0.007125891 |
| TIFAB | 3 | 0.007125891 |
| GBX1 | 3 | 0.007125891 |
| LENG9 | 3 | 0.007125891 |
| BARX1 | 3 | 0.007125891 |
| ZNF280D | 3 | 0.007125891 |
| WDR18 | 3 | 0.007125891 |
| SNX16 | 3 | 0.007125891 |
| METTL4 | 3 | 0.007125891 |
| OSER1 | 3 | 0.007125891 |
| MRS2 | 3 | 0.007125891 |
| CLEC7A | 3 | 0.007125891 |
| RAD52 | 3 | 0.007125891 |
| LCN9 | 3 | 0.007125891 |
| POMC | 3 | 0.007125891 |
| THUMPD3 | 3 | 0.007125891 |
| LRRC2 | 3 | 0.007125891 |
| SECTM1 | 3 | 0.007125891 |
| CENPW | 3 | 0.007125891 |
| KRTAP4-12 | 3 | 0.007125891 |
| SLC30A7 | 3 | 0.007125891 |
| ACER2 | 3 | 0.007125891 |
| TUBB6 | 3 | 0.007125891 |
| FHL2 | 3 | 0.007125891 |
| FAM153A | 3 | 0.007125891 |
| NDUFA12 | 3 | 0.007125891 |
| POC5 | 3 | 0.007125891 |
| KRTAP10-5 | 3 | 0.007125891 |
| OR2D3 | 3 | 0.007125891 |
| HHIPL1 | 3 | 0.007125891 |
| PTTG1IP | 3 | 0.007125891 |
| AMD1 | 3 | 0.007125891 |
| DCAF16 | 3 | 0.007125891 |
| UPF3B | 3 | 0.007125891 |
| NPPB | 3 | 0.007125891 |
| ZFP1 | 3 | 0.007125891 |
| CHMP2B | 3 | 0.007125891 |
| GGTLC1 | 3 | 0.007125891 |
| AGR2 | 3 | 0.007125891 |
| ARID3A | 3 | 0.007125891 |
| TOR4A | 3 | 0.007125891 |
| TMEM144 | 3 | 0.007125891 |
| TMEM106C | 3 | 0.007125891 |
| MEMO1 | 3 | 0.007125891 |
| HMSD | 3 | 0.007125891 |
| CFLAR | 3 | 0.007125891 |
| URB1 | 3 | 0.007125891 |
| FAXC | 3 | 0.007125891 |
| DCP1B | 3 | 0.007125891 |
| ANGPTL6 | 3 | 0.007125891 |
| MAPK1 | 3 | 0.007125891 |
| SF3A3 | 3 | 0.007125891 |
| MUC19 | 3 | 0.007125891 |
| PMM1 | 3 | 0.007125891 |
| NCS1 | 3 | 0.007125891 |
| DNAH14 | 3 | 0.007125891 |
| ACADM | 3 | 0.007125891 |
| RAC3 | 3 | 0.007125891 |
| UTP15 | 3 | 0.007125891 |
| ESPN | 3 | 0.007125891 |
| HYI | 3 | 0.007125891 |
| SIRPG | 3 | 0.007125891 |
| KY | 3 | 0.007125891 |
| KDSR | 3 | 0.007125891 |
| PPP1CA | 3 | 0.007125891 |
| CD36 | 3 | 0.007125891 |
| TSPAN6 | 3 | 0.007125891 |
| SCD5 | 3 | 0.007125891 |
| NDFIP1 | 3 | 0.007125891 |
| LAPTM4A | 3 | 0.007125891 |
| DCUN1D3 | 3 | 0.007125891 |
| PINX1 | 3 | 0.007125891 |
| TSPYL1 | 3 | 0.007125891 |
| SLFN14 | 3 | 0.007125891 |
| NICN1 | 3 | 0.007125891 |
| SNRNP27 | 3 | 0.007125891 |
| RNPEP | 3 | 0.007125891 |
| IFNB1 | 3 | 0.007125891 |
| H2AC4 | 3 | 0.007125891 |
| UROD | 3 | 0.007125891 |
| CDC6 | 3 | 0.007125891 |
| ARRDC5 | 3 | 0.007125891 |
| CEACAM7 | 3 | 0.007125891 |
| FEM1C | 3 | 0.007125891 |
| EXOSC3 | 3 | 0.007125891 |
| OR10H3 | 3 | 0.007125891 |
| IL2RA | 3 | 0.007125891 |
| HINT2 | 3 | 0.007125891 |
| TENT5C | 3 | 0.007125891 |
| IFT43 | 3 | 0.007125891 |
| EIF4A3 | 3 | 0.007125891 |
| TIAF1 | 3 | 0.007125891 |
| DMRTA2 | 3 | 0.007125891 |
| ELOB | 3 | 0.007125891 |
| COLEC10 | 3 | 0.007125891 |
| LMNB2 | 3 | 0.007125891 |
| PRPH | 3 | 0.007125891 |
| RILPL2 | 3 | 0.007125891 |
| SCMH1 | 3 | 0.007125891 |
| ERAS | 3 | 0.007125891 |
| CTDSP2 | 3 | 0.007125891 |
| IFITM1 | 3 | 0.007125891 |
| CLEC2D | 3 | 0.007125891 |
| C3orf33 | 3 | 0.007125891 |
| CXCL9 | 3 | 0.007125891 |
| CMTM8 | 3 | 0.007125891 |
| IGKV2D-28 | 3 | 0.007125891 |
| REPIN1 | 3 | 0.007125891 |
| FOXF1 | 3 | 0.007125891 |
| ISCA1 | 3 | 0.007125891 |
| NPTXR | 3 | 0.007125891 |
| APOL2 | 3 | 0.007125891 |
| WDR25 | 3 | 0.007125891 |
| OXT | 3 | 0.007125891 |
| ALKBH8 | 3 | 0.007125891 |
| LMO3 | 3 | 0.007125891 |
| HSD17B1 | 3 | 0.007125891 |
| GNAI3 | 3 | 0.007125891 |
| PDHB | 3 | 0.007125891 |
| ARMCX6 | 3 | 0.007125891 |
| MAP3K8 | 3 | 0.007125891 |
| RAP1GAP | 3 | 0.007125891 |
| CFAP410 | 3 | 0.007125891 |
| MEF2D | 3 | 0.007125891 |
| CDCA7 | 3 | 0.007125891 |
| TRIM38 | 3 | 0.007125891 |
| VDR | 3 | 0.007125891 |
| ADTRP | 3 | 0.007125891 |
| UPP2 | 3 | 0.007125891 |
| NT5M | 3 | 0.007125891 |
| VCX3B | 3 | 0.007125891 |
| ZNF143 | 3 | 0.007125891 |
| KLRC4 | 3 | 0.007125891 |
| DMAC2 | 3 | 0.007125891 |
| C6orf89 | 3 | 0.007125891 |
| NR2F6 | 3 | 0.007125891 |
| MXI1 | 3 | 0.007125891 |
| KIR2DL3 | 3 | 0.007125891 |
| EGR4 | 3 | 0.007125891 |
| CCR9 | 3 | 0.007125891 |
| TMEM196 | 3 | 0.007125891 |
| ESRRA | 3 | 0.007125891 |
| FBXO46 | 3 | 0.007125891 |
| TRAF4 | 3 | 0.007125891 |
| EAPP | 3 | 0.007125891 |
| DNAJB6 | 3 | 0.007125891 |
| SPATA25 | 3 | 0.007125891 |
| OR52E2 | 3 | 0.007125891 |
| USP14 | 3 | 0.007125891 |
| CALML3 | 3 | 0.007125891 |
| PDGFRL | 3 | 0.007125891 |
| CHIC1 | 3 | 0.007125891 |
| DGAT2L6 | 3 | 0.007125891 |
| NCBP2 | 3 | 0.007125891 |
| TMEM236 | 3 | 0.007125891 |
| TMEM218 | 3 | 0.007125891 |
| CPSF4 | 3 | 0.007125891 |
| MED31 | 3 | 0.007125891 |
| PSMA7 | 3 | 0.007125891 |
| ZPR1 | 3 | 0.007125891 |
| SGSH | 3 | 0.007125891 |
| NSG1 | 3 | 0.007125891 |
| MAPRE1 | 3 | 0.007125891 |
| SSU72 | 3 | 0.007125891 |
| AQP3 | 3 | 0.007125891 |
| NTS | 3 | 0.007125891 |
| RBM11 | 3 | 0.007125891 |
| EIF2S2 | 3 | 0.007125891 |
| ZNF468 | 3 | 0.007125891 |
| ASCC1 | 3 | 0.007125891 |
| CHDH | 3 | 0.007125891 |
| ARHGAP11B | 3 | 0.007125891 |
| CD226 | 3 | 0.007125891 |
| MLYCD | 3 | 0.007125891 |
| FOSL2 | 3 | 0.007125891 |
| SMURF2 | 3 | 0.007125891 |
| COMMD2 | 3 | 0.007125891 |
| ALG12 | 3 | 0.007125891 |
| NGF | 3 | 0.007125891 |
| COX15 | 3 | 0.007125891 |
| PGBD2 | 3 | 0.007125891 |
| IL17F | 3 | 0.007125891 |
| HDHD2 | 3 | 0.007125891 |
| TCEAL5 | 3 | 0.007125891 |
| CAPNS1 | 3 | 0.007125891 |
| PIGU | 3 | 0.007125891 |
| PRUNE1 | 3 | 0.007125891 |
| CD160 | 3 | 0.007125891 |
| PPP1R17 | 3 | 0.007125891 |
| KRTAP20-1 | 3 | 0.007125891 |
| SLC35E4 | 3 | 0.007125891 |
| DNAJC24 | 3 | 0.007125891 |
| SLC35B4 | 3 | 0.007125891 |
| H2AX | 3 | 0.007125891 |
| CD300LB | 3 | 0.007125891 |
| SSBP1 | 3 | 0.007125891 |
| LCE2C | 3 | 0.007125891 |
| OR51D1 | 3 | 0.007125891 |
| SKP2 | 3 | 0.007125891 |
| GPRC5D | 3 | 0.007125891 |
| CD5 | 3 | 0.007125891 |
| RAET1L | 3 | 0.007125891 |
| CWC15 | 3 | 0.007125891 |
| PRELID3A | 3 | 0.007125891 |
| BBS5 | 3 | 0.007125891 |
| OR6A2 | 3 | 0.007125891 |
| LTB4R2 | 3 | 0.007125891 |
| TSSC4 | 3 | 0.007125891 |
| CALB1 | 3 | 0.007125891 |
| JUN | 3 | 0.007125891 |
| SLC10A1 | 3 | 0.007125891 |
| PPM1J | 3 | 0.007125891 |
| DGUOK | 3 | 0.007125891 |
| PAQR6 | 3 | 0.007125891 |
| TMEM241 | 3 | 0.007125891 |
| ITM2C | 3 | 0.007125891 |
| SLFN12L | 3 | 0.007125891 |
| TIMP2 | 3 | 0.007125891 |
| GNG7 | 3 | 0.007125891 |
| OR8B2 | 3 | 0.007125891 |
| CRISP2 | 3 | 0.007125891 |
| L2HGDH | 3 | 0.007125891 |
| SPC25 | 3 | 0.007125891 |
| METTL2B | 3 | 0.007125891 |
| ST7L | 3 | 0.007125891 |
| CD247 | 3 | 0.007125891 |
| CCDC122 | 3 | 0.007125891 |
| NPTX1 | 3 | 0.007125891 |
| ARPC5 | 3 | 0.007125891 |
| GPR27 | 3 | 0.007125891 |
| TP53I3 | 3 | 0.007125891 |
| TMEM50A | 3 | 0.007125891 |
| TMX1 | 3 | 0.007125891 |
| MRGPRE | 3 | 0.007125891 |
| CNRIP1 | 3 | 0.007125891 |
| CBR1 | 3 | 0.007125891 |
| TMX4 | 3 | 0.007125891 |
| CCNYL1 | 3 | 0.007125891 |
| HES1 | 3 | 0.007125891 |
| TTC23 | 3 | 0.007125891 |
| CENPA | 3 | 0.007125891 |
| UBE2G2 | 3 | 0.007125891 |
| TPI1 | 3 | 0.007125891 |
| CYB5D1 | 3 | 0.007125891 |
| DPH6 | 3 | 0.007125891 |
| IGKV6D-41 | 3 | 0.007125891 |
| RALB | 3 | 0.007125891 |
| KRTAP13-2 | 3 | 0.007125891 |
| MRPL18 | 3 | 0.007125891 |
| ADPRS | 3 | 0.007125891 |
| THUMPD1 | 3 | 0.007125891 |
| UBC | 3 | 0.007125891 |
| H1-7 | 3 | 0.007125891 |
| NBPF9 | 3 | 0.007125891 |
| GDF1 | 3 | 0.007125891 |
| TMEM216 | 3 | 0.007125891 |
| SH3BGRL2 | 3 | 0.007125891 |
| OR52I1 | 3 | 0.007125891 |
| MCAT | 3 | 0.007125891 |
| NMS | 3 | 0.007125891 |
| RAB31 | 3 | 0.007125891 |
| LLCFC1 | 3 | 0.007125891 |
| CPLX2 | 3 | 0.007125891 |
| PSMB3 | 3 | 0.007125891 |
| FJX1 | 3 | 0.007125891 |
| SPDYC | 3 | 0.007125891 |
| SPHK1 | 3 | 0.007125891 |
| LDLRAD2 | 3 | 0.007125891 |
| ATP6V1E1 | 3 | 0.007125891 |
| ITLN1 | 3 | 0.007125891 |
| ARFIP2 | 3 | 0.007125891 |
| GUCA2B | 3 | 0.007125891 |
| TVP23B | 3 | 0.007125891 |
| SEC61B | 3 | 0.007125891 |
| TIRAP | 3 | 0.007125891 |
| DAPL1 | 3 | 0.007125891 |
| PGAM2 | 3 | 0.007125891 |
| RBM20 | 3 | 0.007125891 |
| TRAPPC3 | 3 | 0.007125891 |
| BATF2 | 3 | 0.007125891 |
| MFAP3 | 3 | 0.007125891 |
| TNFRSF18 | 3 | 0.007125891 |
| UBE2T | 3 | 0.007125891 |
| PEF1 | 3 | 0.007125891 |
| DKKL1 | 3 | 0.007125891 |
| SEPTIN1 | 3 | 0.007125891 |
| GLRX2 | 3 | 0.007125891 |
| FUNDC2 | 3 | 0.007125891 |
| FAAH | 3 | 0.007125891 |
| ALG3 | 3 | 0.007125891 |
| ALDOA | 3 | 0.007125891 |
| ARMC12 | 3 | 0.007125891 |
| SEC23B | 3 | 0.007125891 |
| MSI2 | 3 | 0.007125891 |
| ANKRD20A4P | 3 | 0.007125891 |
| AKR1C3 | 3 | 0.007125891 |
| IGKV1D-13 | 3 | 0.007125891 |
| TBPL1 | 3 | 0.007125891 |
| OR1K1 | 3 | 0.007125891 |
| SNAPC2 | 3 | 0.007125891 |
| GIMAP2 | 3 | 0.007125891 |
| HPDL | 3 | 0.007125891 |
| ANKRD16 | 3 | 0.007125891 |
| GSTA4 | 3 | 0.007125891 |
| C22orf15 | 3 | 0.007125891 |
| GPHB5 | 3 | 0.007125891 |
| RNF121 | 3 | 0.007125891 |
| PCTP | 3 | 0.007125891 |
| WAS | 3 | 0.007125891 |
| CYP2C9 | 3 | 0.007125891 |
| ZFYVE21 | 3 | 0.007125891 |
| H2BC7 | 3 | 0.007125891 |
| GAPDHS | 3 | 0.007125891 |
| HMGXB3 | 3 | 0.007125891 |
| MAD2L1BP | 3 | 0.007125891 |
| RAP2B | 3 | 0.007125891 |
| CLDN7 | 3 | 0.007125891 |
| H3C13 | 3 | 0.007125891 |
| LCMT1 | 3 | 0.007125891 |
| HOMER1 | 3 | 0.007125891 |
| SLC35A2 | 3 | 0.007125891 |
| MS4A2 | 3 | 0.007125891 |
| N6AMT1 | 3 | 0.007125891 |
| CHST7 | 3 | 0.007125891 |
| CCDC137 | 3 | 0.007125891 |
| NCCRP1 | 3 | 0.007125891 |
| DDO | 3 | 0.007125891 |
| HSD17B3 | 3 | 0.007125891 |
| TMEM219 | 3 | 0.007125891 |
| RHEX | 3 | 0.007125891 |
| ADH7 | 3 | 0.007125891 |
| GJB6 | 3 | 0.007125891 |
| NREP | 3 | 0.007125891 |
| OR5K1 | 3 | 0.007125891 |
| SPRING1 | 3 | 0.007125891 |
| RPL10A | 3 | 0.007125891 |
| TMED5 | 3 | 0.007125891 |
| AANAT | 3 | 0.007125891 |
| CTAGE9 | 3 | 0.007125891 |
| PHF11 | 3 | 0.007125891 |
| HPD | 3 | 0.007125891 |
| CELA2B | 3 | 0.007125891 |
| TTC25 | 3 | 0.007125891 |
| HMG20B | 3 | 0.007125891 |
| FOXK2 | 3 | 0.007125891 |
| H4C6 | 3 | 0.007125891 |
| EBLN2 | 3 | 0.007125891 |
| EIF4EBP2 | 3 | 0.007125891 |
| C8G | 3 | 0.007125891 |
| CERS1 | 3 | 0.007125891 |
| GPATCH3 | 3 | 0.007125891 |
| FOXC2 | 3 | 0.007125891 |
| PIGP | 3 | 0.007125891 |
| OR4F6 | 3 | 0.007125891 |
| NQO1 | 3 | 0.007125891 |
| UFC1 | 3 | 0.007125891 |
| CCNDBP1 | 3 | 0.007125891 |
| C4orf46 | 3 | 0.007125891 |
| OR2V2 | 3 | 0.007125891 |
| KPNA7 | 3 | 0.007125891 |
| OR5T1 | 3 | 0.007125891 |
| RGPD2 | 3 | 0.007125891 |
| AP4S1 | 3 | 0.007125891 |
| ANKRD29 | 3 | 0.007125891 |
| N4BP2L1 | 3 | 0.007125891 |
| PRPF39 | 3 | 0.007125891 |
| RNF170 | 3 | 0.007125891 |
| MGARP | 3 | 0.007125891 |
| VDAC3 | 3 | 0.007125891 |
| TSSK3 | 3 | 0.007125891 |
| RBM18 | 3 | 0.007125891 |
| RRAGC | 3 | 0.007125891 |
| CD209 | 3 | 0.007125891 |
| RRAS | 3 | 0.007125891 |
| DCTPP1 | 3 | 0.007125891 |
| PSMB5 | 3 | 0.007125891 |
| THOC6 | 3 | 0.007125891 |
| SLAMF8 | 3 | 0.007125891 |
| UBE2G1 | 3 | 0.007125891 |
| ZNF558 | 3 | 0.007125891 |
| PRMT1 | 3 | 0.007125891 |
| PPIB | 3 | 0.007125891 |
| LIM2 | 3 | 0.007125891 |
| SLC16A4 | 3 | 0.007125891 |
| OGFOD1 | 3 | 0.007125891 |
| EIF2S3 | 3 | 0.007125891 |
| PTK6 | 3 | 0.007125891 |
| FOXI2 | 3 | 0.007125891 |
| FIBP | 3 | 0.007125891 |
| IGLV1-50 | 3 | 0.007125891 |
| KHDC4 | 3 | 0.007125891 |
| S1PR3 | 3 | 0.007125891 |
| TAS2R3 | 3 | 0.007125891 |
| CABCOCO1 | 3 | 0.007125891 |
| PTGDS | 3 | 0.007125891 |
| CLEC9A | 3 | 0.007125891 |
| GDA | 3 | 0.007125891 |
| LY6G6C | 3 | 0.007125891 |
| PANX3 | 3 | 0.007125891 |
| H2BC1 | 3 | 0.007125891 |
| PJVK | 3 | 0.007125891 |
| KCTD2 | 3 | 0.007125891 |
| C16orf87 | 3 | 0.007125891 |
| MCM5 | 3 | 0.007125891 |
| BTF3L4 | 3 | 0.007125891 |
| HBD | 3 | 0.007125891 |
| STOM | 3 | 0.007125891 |
| LGALS3 | 3 | 0.007125891 |
| NSMCE4A | 3 | 0.007125891 |
| SHISA4 | 3 | 0.007125891 |
| PEMT | 3 | 0.007125891 |
| SEC62 | 3 | 0.007125891 |
| CEACAM8 | 3 | 0.007125891 |
| HAO2 | 3 | 0.007125891 |
| PDX1 | 3 | 0.007125891 |
| AKR7A3 | 3 | 0.007125891 |
| ZRSR2 | 3 | 0.007125891 |
| HOXC5 | 3 | 0.007125891 |
| DNAJC9 | 3 | 0.007125891 |
| CCT2 | 3 | 0.007125891 |
| CMTM7 | 3 | 0.007125891 |
| FAM76B | 3 | 0.007125891 |
| HS3ST1 | 3 | 0.007125891 |
| R3HDML | 3 | 0.007125891 |
| ZNF80 | 3 | 0.007125891 |
| RENBP | 3 | 0.007125891 |
| WNT8A | 3 | 0.007125891 |
| PFN4 | 3 | 0.007125891 |
| DEFB119 | 3 | 0.007125891 |
| MOSPD2 | 3 | 0.007125891 |
| TNFAIP6 | 3 | 0.007125891 |
| PDGFC | 3 | 0.007125891 |
| AARD | 3 | 0.007125891 |
| NKIRAS2 | 3 | 0.007125891 |
| PAK1IP1 | 3 | 0.007125891 |
| EXT2 | 3 | 0.007125891 |
| BTG3 | 3 | 0.007125891 |
| RCN2 | 3 | 0.007125891 |
| ZBTB3 | 3 | 0.007125891 |
| FGL2 | 3 | 0.007125891 |
| PAQR3 | 3 | 0.007125891 |
| ZSCAN16 | 3 | 0.007125891 |
| VKORC1L1 | 3 | 0.007125891 |
| CLEC3B | 3 | 0.007125891 |
| RAB40B | 3 | 0.007125891 |
| TAGLN | 3 | 0.007125891 |
| PSCA | 3 | 0.007125891 |
| FOXA1 | 3 | 0.007125891 |
| ZDHHC13 | 3 | 0.007125891 |
| RRP7A | 3 | 0.007125891 |
| TBC1D21 | 3 | 0.007125891 |
| FBP1 | 3 | 0.007125891 |
| RNF182 | 3 | 0.007125891 |
| H2AC13 | 3 | 0.007125891 |
| PTPN20 | 3 | 0.007125891 |
| POLR3H | 3 | 0.007125891 |
| C2orf88 | 3 | 0.007125891 |
| PSMD7 | 3 | 0.007125891 |
| FOXD4L3 | 3 | 0.007125891 |
| BMP8A | 3 | 0.007125891 |
| EIF4E1B | 3 | 0.007125891 |
| JAM2 | 3 | 0.007125891 |
| TOB2 | 3 | 0.007125891 |
| OR8B8 | 3 | 0.007125891 |
| CXorf56 | 3 | 0.007125891 |
| TMUB2 | 3 | 0.007125891 |
| SAPCD2 | 3 | 0.007125891 |
| HLA-DQB1 | 3 | 0.007125891 |
| ACSF2 | 3 | 0.007125891 |
| APOBEC3D | 3 | 0.007125891 |
| SYT15 | 3 | 0.007125891 |
| TMEM143 | 3 | 0.007125891 |
| ABHD17B | 3 | 0.007125891 |
| GABRP | 3 | 0.007125891 |
| DMC1 | 3 | 0.007125891 |
| NMU | 3 | 0.007125891 |
| NOL10 | 3 | 0.007125891 |
| KRTAP3-1 | 3 | 0.007125891 |
| CSRP1 | 3 | 0.007125891 |
| CCNO | 3 | 0.007125891 |
| IGFLR1 | 3 | 0.007125891 |
| HRH1 | 3 | 0.007125891 |
| APOBEC3A | 3 | 0.007125891 |
| CFHR1 | 3 | 0.007125891 |
| ZNF799 | 3 | 0.007125891 |
| TMEM202 | 3 | 0.007125891 |
| RIC8A | 3 | 0.007125891 |
| PRSS21 | 3 | 0.007125891 |
| HIGD1B | 3 | 0.007125891 |
| FKBP14 | 3 | 0.007125891 |
| PGK1 | 3 | 0.007125891 |
| SELENOV | 3 | 0.007125891 |
| RAB5C | 3 | 0.007125891 |
| HSPA1B | 3 | 0.007125891 |
| SFXN4 | 3 | 0.007125891 |
| MAK16 | 3 | 0.007125891 |
| RFXAP | 3 | 0.007125891 |
| HHATL | 3 | 0.007125891 |
| MYLIP | 3 | 0.007125891 |
| C16orf71 | 3 | 0.007125891 |
| AMDHD2 | 3 | 0.007125891 |
| SPATA7 | 3 | 0.007125891 |
| KXD1 | 3 | 0.007125891 |
| CBARP | 3 | 0.007125891 |
| CELA3A | 3 | 0.007125891 |
| C2 | 3 | 0.007125891 |
| ANP32B | 3 | 0.007125891 |
| HHLA1 | 3 | 0.007125891 |
| HPCAL1 | 3 | 0.007125891 |
| TRAPPC5 | 3 | 0.007125891 |
| ZNF500 | 3 | 0.007125891 |
| UBE2E1 | 3 | 0.007125891 |
| KRTAP4-2 | 3 | 0.007125891 |
| DIMT1 | 3 | 0.007125891 |
| REEP3 | 3 | 0.007125891 |
| ITFG1 | 3 | 0.007125891 |
| IFNA17 | 3 | 0.007125891 |
| SRRD | 3 | 0.007125891 |
| CCNE2 | 3 | 0.007125891 |
| ZNF397 | 3 | 0.007125891 |
| OR4B1 | 3 | 0.007125891 |
| TNNC2 | 3 | 0.007125891 |
| CARD19 | 3 | 0.007125891 |
| TEX35 | 3 | 0.007125891 |
| ZDHHC23 | 3 | 0.007125891 |
| CLVS2 | 3 | 0.007125891 |
| LHB | 3 | 0.007125891 |
| OLFM2 | 3 | 0.007125891 |
| IGLV3-9 | 3 | 0.007125891 |
| POLR2D | 3 | 0.007125891 |
| RNASE3 | 3 | 0.007125891 |
| AMOTL2 | 3 | 0.007125891 |
| GNPNAT1 | 3 | 0.007125891 |
| SACM1L | 3 | 0.007125891 |
| TERB2 | 3 | 0.007125891 |
| CATSPERG | 3 | 0.007125891 |
| MRGPRF | 3 | 0.007125891 |
| MAOA | 3 | 0.007125891 |
| ZNF474 | 3 | 0.007125891 |
| CFAP126 | 3 | 0.007125891 |
| KRTAP10-2 | 3 | 0.007125891 |
| OR10V1 | 3 | 0.007125891 |
| TAF7 | 3 | 0.007125891 |
| GPR21 | 3 | 0.007125891 |
| LRIT3 | 3 | 0.007125891 |
| ICAM4 | 3 | 0.007125891 |
| MUTYH | 3 | 0.007125891 |
| RBM7 | 3 | 0.007125891 |
| RASGEF1C | 3 | 0.007125891 |
| DCUN1D4 | 3 | 0.007125891 |
| EFNA1 | 3 | 0.007125891 |
| CCDC25 | 3 | 0.007125891 |
| ODC1 | 3 | 0.007125891 |
| PIP4P1 | 3 | 0.007125891 |
| ATF4 | 3 | 0.007125891 |
| LMNTD2 | 3 | 0.007125891 |
| TFAM | 3 | 0.007125891 |
| REEP4 | 3 | 0.007125891 |
| CA10 | 3 | 0.007125891 |
| OR5M10 | 3 | 0.007125891 |
| FAM72A | 3 | 0.007125891 |
| DNAAF6 | 3 | 0.007125891 |
| LMAN2 | 3 | 0.007125891 |
| IL1A | 3 | 0.007125891 |
| ZNF781 | 3 | 0.007125891 |
| C1orf50 | 3 | 0.007125891 |
| CELA1 | 3 | 0.007125891 |
| ISCA2 | 3 | 0.007125891 |
| LYPD3 | 3 | 0.007125891 |
| ORC6 | 3 | 0.007125891 |
| OR8U1 | 3 | 0.007125891 |
| FAM222A | 3 | 0.007125891 |
| GIPC2 | 3 | 0.007125891 |
| YWHAQ | 3 | 0.007125891 |
| TMEM151A | 3 | 0.007125891 |
| CLEC4M | 3 | 0.007125891 |
| MXD1 | 3 | 0.007125891 |
| H4C5 | 3 | 0.007125891 |
| HABP4 | 3 | 0.007125891 |
| PSMB11 | 3 | 0.007125891 |
| FAM43A | 3 | 0.007125891 |
| PNKD | 3 | 0.007125891 |
| KLF10 | 3 | 0.007125891 |
| TEX26 | 3 | 0.007125891 |
| PSMD14 | 3 | 0.007125891 |
| DHRSX | 3 | 0.007125891 |
| TTL | 3 | 0.007125891 |
| RASA4 | 3 | 0.007125891 |
| PPA1 | 3 | 0.007125891 |
| WTIP | 3 | 0.007125891 |
| SPRY2 | 3 | 0.007125891 |
| NAE1 | 3 | 0.007125891 |
| SPCS3 | 3 | 0.007125891 |
| WNT9B | 3 | 0.007125891 |
| CA1 | 3 | 0.007125891 |
| FOXD2 | 3 | 0.007125891 |
| AKTIP | 3 | 0.007125891 |
| METTL18 | 3 | 0.007125891 |
| CRYBB3 | 3 | 0.007125891 |
| MARCHF1 | 3 | 0.007125891 |
| EFS | 3 | 0.007125891 |
| ATL3 | 3 | 0.007125891 |
| NACA2 | 3 | 0.007125891 |
| PCNA | 3 | 0.007125891 |
| LYSMD3 | 3 | 0.007125891 |
| ALKBH1 | 3 | 0.007125891 |
| GPR65 | 3 | 0.007125891 |
| BLOC1S2 | 3 | 0.007125891 |
| HEXIM1 | 3 | 0.007125891 |
| HSPB11 | 3 | 0.007125891 |
| TWF2 | 3 | 0.007125891 |
| LRRC59 | 3 | 0.007125891 |
| PAWR | 3 | 0.007125891 |
| ZBTB17 | 3 | 0.007125891 |
| CMC4 | 3 | 0.007125891 |
| NAMPT | 3 | 0.007125891 |
| PEX3 | 3 | 0.007125891 |
| INKA1 | 3 | 0.007125891 |
| FGF16 | 3 | 0.007125891 |
| ZFAND5 | 3 | 0.007125891 |
| INHBE | 3 | 0.007125891 |
| BAK1 | 3 | 0.007125891 |
| SNX4 | 3 | 0.007125891 |
| LAGE3 | 3 | 0.007125891 |
| IGHV3-38 | 3 | 0.007125891 |
| STK26 | 3 | 0.007125891 |
| EIF5A2 | 3 | 0.007125891 |
| RAB11A | 3 | 0.007125891 |
| RFLNB | 3 | 0.007125891 |
| TRAFD1 | 3 | 0.007125891 |
| ARSH | 3 | 0.007125891 |
| SPIC | 3 | 0.007125891 |
| OR4L1 | 3 | 0.007125891 |
| CLIC3 | 3 | 0.007125891 |
| SH2D4B | 3 | 0.007125891 |
| PPP3R1 | 3 | 0.007125891 |
| PEG10 | 3 | 0.007125891 |
| RAPSN | 3 | 0.007125891 |
| CPLX1 | 3 | 0.007125891 |
| BBS10 | 3 | 0.007125891 |
| TUBB | 3 | 0.007125891 |
| CCDC47 | 3 | 0.007125891 |
| SMIM21 | 3 | 0.007125891 |
| H2BC8 | 3 | 0.007125891 |
| ARHGAP42 | 3 | 0.007125891 |
| PSG11 | 3 | 0.007125891 |
| CACYBP | 3 | 0.007125891 |
| PKM | 3 | 0.007125891 |
| RAB14 | 3 | 0.007125891 |
| SELENOI | 3 | 0.007125891 |
| OR5M11 | 3 | 0.007125891 |
| CTAG2 | 3 | 0.007125891 |
| ODAM | 3 | 0.007125891 |
| NOVA2 | 3 | 0.007125891 |
| TWIST1 | 3 | 0.007125891 |
| SOX1 | 3 | 0.007125891 |
| AAGAB | 3 | 0.007125891 |
| FCGR2B | 3 | 0.007125891 |
| EID1 | 3 | 0.007125891 |
| HMGB2 | 3 | 0.007125891 |
| TMEM138 | 3 | 0.007125891 |
| SRC | 3 | 0.007125891 |
| P4HA3 | 3 | 0.007125891 |
| OTULINL | 3 | 0.007125891 |
| THAP8 | 3 | 0.007125891 |
| COX6C | 3 | 0.007125891 |
| ELOVL6 | 3 | 0.007125891 |
| TMED10 | 3 | 0.007125891 |
| SOX4 | 3 | 0.007125891 |
| EEF1AKMT1 | 3 | 0.007125891 |
| CFDP1 | 3 | 0.007125891 |
| CLEC4G | 3 | 0.007125891 |
| TMEM200B | 3 | 0.007125891 |
| ADRA2C | 3 | 0.007125891 |
| IMPDH1 | 3 | 0.007125891 |
| SPIN2B | 3 | 0.007125891 |
| NUDT15 | 3 | 0.007125891 |
| SMTNL2 | 3 | 0.007125891 |
| TMEM86A | 3 | 0.007125891 |
| ZNRF1 | 3 | 0.007125891 |
| CTAGE15 | 3 | 0.007125891 |
| SUFU | 3 | 0.007125891 |
| IFI30 | 3 | 0.007125891 |
| MAFK | 3 | 0.007125891 |
| MIER1 | 3 | 0.007125891 |
| MSL1 | 3 | 0.007125891 |
| TMEM184B | 3 | 0.007125891 |
| TMBIM6 | 3 | 0.007125891 |
| SERPINA3 | 3 | 0.007125891 |
| SFTPA1 | 3 | 0.007125891 |
| GRAMD2A | 3 | 0.007125891 |
| FUS | 3 | 0.007125891 |
| DCTD | 3 | 0.007125891 |
| CDC42EP1 | 3 | 0.007125891 |
| PDRG1 | 3 | 0.007125891 |
| LYSMD1 | 3 | 0.007125891 |
| NPM1 | 3 | 0.007125891 |
| CCDC74A | 3 | 0.007125891 |
| ACTR1A | 3 | 0.007125891 |
| TMEM222 | 3 | 0.007125891 |
| FBXO27 | 3 | 0.007125891 |
| HDAC10 | 3 | 0.007125891 |
| APOF | 3 | 0.007125891 |
| TAS2R46 | 3 | 0.007125891 |
| IL2 | 3 | 0.007125891 |
| LYNX1 | 3 | 0.007125891 |
| CYP2B6 | 3 | 0.007125891 |
| FUOM | 3 | 0.007125891 |
| DEFB118 | 3 | 0.007125891 |
| FAM193B | 3 | 0.007125891 |
| UBXN2B | 3 | 0.007125891 |
| TMEM141 | 3 | 0.007125891 |
| HUS1B | 3 | 0.007125891 |
| GPR39 | 3 | 0.007125891 |
| SKI | 3 | 0.007125891 |
| IL37 | 3 | 0.007125891 |
| NPTN | 3 | 0.007125891 |
| NME6 | 3 | 0.007125891 |
| CYBC1 | 3 | 0.007125891 |
| NHLH2 | 3 | 0.007125891 |
| TREML2 | 3 | 0.007125891 |
| CST2 | 3 | 0.007125891 |
| GTF3A | 3 | 0.007125891 |
| GALE | 3 | 0.007125891 |
| STARD3NL | 3 | 0.007125891 |
| TNFSF8 | 3 | 0.007125891 |
| RNF144A | 3 | 0.007125891 |
| KANK3 | 3 | 0.007125891 |
| GNG11 | 3 | 0.007125891 |
| BCL2L15 | 3 | 0.007125891 |
| EARS2 | 3 | 0.007125891 |
| KRTAP11-1 | 3 | 0.007125891 |
| GRK7 | 3 | 0.007125891 |
| KRT77 | 3 | 0.007125891 |
| RIOX1 | 3 | 0.007125891 |
| TM4SF20 | 3 | 0.007125891 |
| SLC25A26 | 3 | 0.007125891 |
| CDHR4 | 3 | 0.007125891 |
| HMCES | 3 | 0.007125891 |
| LAMP5 | 3 | 0.007125891 |
| CFL1 | 3 | 0.007125891 |
| SMS | 3 | 0.007125891 |
| GTSE1 | 3 | 0.007125891 |
| RDH10 | 3 | 0.007125891 |
| SIRT2 | 3 | 0.007125891 |
| IFT22 | 3 | 0.007125891 |
| TSEN15 | 3 | 0.007125891 |
| RTN2 | 3 | 0.007125891 |
| CCDC126 | 3 | 0.007125891 |
| TECTB | 3 | 0.007125891 |
| OLR1 | 3 | 0.007125891 |
| FAM86B1 | 3 | 0.007125891 |
| VASH2 | 3 | 0.007125891 |
| ARHGEF16 | 3 | 0.007125891 |
| ZDHHC21 | 3 | 0.007125891 |
| KIAA0513 | 3 | 0.007125891 |
| IZUMO1R | 3 | 0.007125891 |
| LRSAM1 | 3 | 0.007125891 |
| CKLF | 3 | 0.007125891 |
| CRADD | 3 | 0.007125891 |
| ARF5 | 3 | 0.007125891 |
| BRCC3 | 3 | 0.007125891 |
| SIGMAR1 | 3 | 0.007125891 |
| CCND1 | 3 | 0.007125891 |
| IGHV3-21 | 3 | 0.007125891 |
| NAT8 | 3 | 0.007125891 |
| DHPS | 3 | 0.007125891 |
| NCF1 | 3 | 0.007125891 |
| NRG4 | 3 | 0.007125891 |
| RAC2 | 3 | 0.007125891 |
| LUC7L | 3 | 0.007125891 |
| COPS7B | 3 | 0.007125891 |
| TMEM61 | 3 | 0.007125891 |
| CRTAP | 3 | 0.007125891 |
| TCEA3 | 3 | 0.007125891 |
| RASL11B | 3 | 0.007125891 |
| ADRM1 | 3 | 0.007125891 |
| NMBR | 3 | 0.007125891 |
| NPY4R | 3 | 0.007125891 |
| FOXC1 | 3 | 0.007125891 |
| P2RY1 | 3 | 0.007125891 |
| SPPL3 | 3 | 0.007125891 |
| CERS2 | 3 | 0.007125891 |
| BBS4 | 3 | 0.007125891 |
| POLR3F | 3 | 0.007125891 |
| CTRB1 | 3 | 0.007125891 |
| RAB3A | 3 | 0.007125891 |
| HAPLN2 | 3 | 0.007125891 |
| DBNDD2 | 3 | 0.007125891 |
| UCN2 | 3 | 0.007125891 |
| RHNO1 | 3 | 0.007125891 |
| TMEM207 | 3 | 0.007125891 |
| TMEM182 | 3 | 0.007125891 |
| CSAG2 | 3 | 0.007125891 |
| PPIL6 | 3 | 0.007125891 |
| BPI | 3 | 0.007125891 |
| PITPNB | 3 | 0.007125891 |
| STK17B | 3 | 0.007125891 |
| PCMT1 | 3 | 0.007125891 |
| GET4 | 3 | 0.007125891 |
| PLPP7 | 3 | 0.007125891 |
| FAM163A | 3 | 0.007125891 |
| CENPT | 3 | 0.007125891 |
| LARS2 | 3 | 0.007125891 |
| MSANTD2 | 3 | 0.007125891 |
| DPEP1 | 3 | 0.007125891 |
| ABHD1 | 3 | 0.007125891 |
| ODF4 | 3 | 0.007125891 |
| OR5B21 | 3 | 0.007125891 |
| RBBP9 | 3 | 0.007125891 |
| PDE6D | 3 | 0.007125891 |
| FAM219A | 3 | 0.007125891 |
| KRBOX4 | 3 | 0.007125891 |
| DPM1 | 3 | 0.007125891 |
| FRA10AC1 | 3 | 0.007125891 |
| MAGEB10 | 3 | 0.007125891 |
| OAS1 | 3 | 0.007125891 |
| TBPL2 | 3 | 0.007125891 |
| KIR2DS4 | 3 | 0.007125891 |
| ODF3L1 | 3 | 0.007125891 |
| H2AB1 | 3 | 0.007125891 |
| ALAD | 3 | 0.007125891 |
| ZBTB8A | 3 | 0.007125891 |
| NMI | 3 | 0.007125891 |
| ZBED6CL | 3 | 0.007125891 |
| ANKRD30B | 3 | 0.007125891 |
| GPX1 | 3 | 0.007125891 |
| OVOL1 | 3 | 0.007125891 |
| SFTPA2 | 3 | 0.007125891 |
| OSCAR | 3 | 0.007125891 |
| SAA2-SAA4 | 3 | 0.007125891 |
| AIP | 3 | 0.007125891 |
| GPX4 | 3 | 0.007125891 |
| KLF6 | 3 | 0.007125891 |
| PIGV | 3 | 0.007125891 |
| CLBA1 | 3 | 0.007125891 |
| ATAD3B | 3 | 0.007125891 |
| SP110 | 3 | 0.007125891 |
| LRRC34 | 3 | 0.007125891 |
| CFI | 3 | 0.007125891 |
| CDK10 | 3 | 0.007125891 |
| TNIP2 | 3 | 0.007125891 |
| TXNDC5 | 3 | 0.007125891 |
| KDM1A | 3 | 0.007125891 |
| VSIG10L | 3 | 0.007125891 |
| ANKRD46 | 3 | 0.007125891 |
[truncated: 108,119 more chars]
