# Supplementary material for: The pivotal role of SFRP2 in promoting glycolysis and progression in the high-risk group based on the glycometabolism prognostic model for colorectal cancer
Source: J Gastroenterol. 2025 Jul 29;60(11):1400–13. doi: 10.1007/s00535-025-02281-5 (PMC12549743; doi:10.1007/s00535-025-02281-5)
Supplement: Supplementary file 20 — Supplementary file20 (PDF 49 KB) [file 535_2025_2281_MOESM20_ESM.pdf]

Table S11. Primer sequences used in the study.

Primer name	Primer sequences	Length	T_m	Location
ANKZF1 sense:	5'-ATGCTCCGGTCTTTCAGGG-3'	19	61.4	65-83
ANKZF1 antisense:	5'-GGTCTGGTCACAAGTTGAACAAA-3'	23	61.1	240-218
CHST1 sense:	5'-ACCTGGCTCGGAACCCTAT-3'	19	62.0	911-929
CHST1 antisense:	5'-CGGTGCCGTATTTGTGCTTG-3'	20	62.2	1048-1029
CLDN9 sense:	5'-CGGCTGCACTGCTTATGCT-3'	19	63.0	509-527
CLDN9 antisense:	5'-GAGGGGATGGAGTAGCCCA-3'	19	62.1	611-593
FKBP4 sense:	5'-GAAGGCGTGCTGAAGGTCAT-3'	20	62.5	91-110
FKBP4 antisense:	5'-TGCCATCTAATAGCCAGCCAG-3'	21	61.7	193-173
GLCE sense:	5'-GCAGCTCGGGTCAACTATAAG-3'	21	60.2	13-33
GLCE antisense:	5'-GAACGCCGTGGAAACTGGA-3'	19	62.5	128-110
GPC1 sense:	5'-TGAAGCTGGTCTACTGTGCTC-3'	21	61.5	752-772
GPC1 antisense:	5'-CCCAGAACTTGTCGGTGATGA-3'	21	61.7	916-896
IDUA sense:	5'-CAGGAGATACATCGGTAGGTACG-3'	23	61.1	480-502
IDUA antisense:	5'-TCATGGAGACGTTGTCAAAGTC-3'	22	60.2	580-559
NOL3 sense:	5'-GACCGCAGCTATGACCCTC-3'	19	61.9	294-312
NOL3 antisense:	5'-CTCCGGTTCAGCCTCTTTAGA-3'	21	60.9	494-474
P4HA1 sense:	5'-AGTACAGCGACAAAAGATCCAG-3'	22	60.0	202-223
P4HA1 antisense:	5'-CTCCAACTCACTCCACTCAGTA-3'	22	60.5	297-276
PMM2 sense:	5'-CTTCGACGTGGATGGGACC-3'	19	62.1	30-48
PMM2 antisense:	5'-CGCCTACCACTCCGATTTTG-3'	20	60.8	136-117
PPP2CB sense:	5'-CTGAACGAGAACCAAGTGCG-3'	20	61.3	67-86
PPP2CB antisense:	5'-ACGAACCTCTTGCACATTTGA-3'	21	60.2	147-127
RBCK1 sense:	5'-TGCTCAGATGCACACCGTC-3'	19	62.3	189-207
RBCK1 antisense:	5'-CAAGACTGGTGGGAAGCCATA-3'	21	61.5	294-274
SPAG4 sense:	5'-TCTCCAGTAGTCTCTGAGGAGC-3'	22	61.8	316-337
SPAG4antisense:	5'-CGGATGGAACAGACCTCCC-3'	19	61.4	494-476
STC2 sense:	5'-GGGTGTGGCGTGTTTGAATG-3'	20	62.1	190-209
STC2 antisense:	5'-TTTCCAGCGTTGTGCAGAAAA-3'	21	61.3	281-261
ALDH1A3 sense:	5'-TGAATGGCACGAATCCAAGAG-3'	21	60.4	114-134
ALDH1A3 antisense:	5'-CACGTCGGGCTTATCTCCT-3'	19	60.8	213-195
ENO3 sense:	5'-GGCTGGTTACCCAGACAAGG-3'	20	62.2	570-589
ENO3 antisense:	5'-TCGTACTTCCCATTGCGATAGAA-3'	23	61.2	644-622
G6PC2 sense:	5'-CAGAAGGACTACCGAGCTTACT-3'	22	60.6	46-67
G6PC2 antisense:	5'-CCAATCCCCAATGACTGCTAC-3'	21	60.4	198-178
NDC1 sense:	5'-AGGTCGCGGGACATACTGT-3'	19	62.7	34-52
NDC1 antisense:	5'-TGCAGATGGGTAGAAATAGCACT-3'	23	61.1	118-96
SLC2A3 sense:	5'-GCTGGGCATCGTTGTTGGA-3'	19	62.9	477-495
SLC2A3 antisense:	5'-GCACTTTGTAGGATAGCAGGAAG-3'	23	60.7	599-577
WNT10A sense:	5'-GGTCAGCACCCAATGACATTC-3'	21	61.3	113-133
WNT10A antisense:	5'-TGGATGGCGATCTGGATGC-3'	19	61.9	281-263
CDKN2A sense:	5'-GATCCAGGTGGGTAGAAGGTC-3'	21	61.0	144-164

CDKN2A antisense:	5'-CCCCTGCAAACTTCGTCCT-3'	19	61.9	217-199
SERPINE1 sense:	5'-ACCGCAACGTGGTTTTCTCA-3'	20	62.6	110-129
SERPINE1 antisense:	5'-TTGAATCCCATAGCTGCTTGAAT-3'	23	60.0	218-196
KREMEN2 sense:	5'-AGGGCATCTACTGGCGCTA-3'	19	62.5	320-338
KREMEN2 antisense:	5'-CTGAGTCCACAAAGCATCCCA-3'	21	62.0	394-374
CALB2 sense:	5'-ACTTTGACGCAGACGGAAATG-3'	21	61.2	80-100
CALB2 antisense:	5'-GAAGTTCTCTTCGGTTGGCAG-3'	21	60.9	294-274
UCHL1 sense:	5'-CCTGTGGCACAATCGGACTTA-3'	21	62.1	266-286
UCHL1 antisense:	5'-CATCTACCCGACATTGGCCTT-3'	21	61.9	466-446
FABP4 sense:	5'-ACTGGGCCAGGAATTTGACG-3'	20	62.5	198-217
FABP4 antisense:	5'-CTCGTGGAAGTGACGCCTT-3'	19	61.9	380-362
SFRP2 sense:	5'-ACGGCATCGAATACCAGAACA-3'	21	61.5	152-172
SFRP2 antisense:	5'-CTCGTCTAGGTCATCGAGGCA-3'	21	62.8	327-307
CLCA1 sense:	5'-ACAACAATGGCTATGAAGGCA-3'	21	60.0	83-103
CLCA1 antisense:	5'-GGTCTCAAGTTTTGGTCTCACAT-3'	23	60.4	294-272
CLCA4 sense:	5'-CCTGCTGCACCAGTCAAATAC-3'	21	61.2	42-62
CLCA4 antisense:	5'-ACGTAGAAGCTGTAGTCACCATA-3'	23	60.3	184-162
UGT2B7 sense:	5'-GATCCCAACAACTCATCCGCT-3'	21	62.2	193-213
UGT2B7 antisense:	5'-CAGCAGCTCACTACAGGGAA-3'	20	61.2	480-461
SFRP2 sense:	5'-ACGGCATCGAATACCAGAACA-3'	21	61.5	152-172
SFRP2 antisense:	5'-CTCGTCTAGGTCATCGAGGCA-3'	21	62.8	327-307
ENO2 sense:	5'-AGCCTCTACGGGCATCTATGA-3'	21	62.0	114-134
ENO2 antisense:	5'-TTCTCAGTCCCATCCAACTCC-3'	21	60.9	305-285
Cyclin D1 sense:	5'-GCTGCGAAGTGGAAACCATC-3'	20	61.6	20-39
Cyclin D1 antisense:	5'-CAGCAGCTCACTACAGGGAA-3'	22	60.8	154-133
C-MYC sense:	5'-GGCTCCTGGCAAAAGGTCA-3'	19	62.2	858-876
C-MYC antisense:	5'-CTGCGTAGTTGTGCTGATGT-3'	20	60.4	976-957
ACTB sense:	5’-CATGTACGTTGCTATCCAGGC-3’	21	60.8	393-413
ACTB antisense:	5’-CTCCTTAATGTCACGCACGAT-3’	21	60.2	642-622
