# Supplementary material for: The pivotal role of SFRP2 in promoting glycolysis and progression in the high-risk group based on the glycometabolism prognostic model for colorectal cancer
Source: J Gastroenterol. 2025 Jul 29;60(11):1400–13. doi: 10.1007/s00535-025-02281-5 (PMC12549743; doi:10.1007/s00535-025-02281-5)
Supplement: Supplementary file 21 — Supplementary file21 (DOCX 29 KB) [file 535_2025_2281_MOESM21_ESM.docx]

**Supplementary material**

**Supplementary methods and materials**

**Screening of differences in the GRG signature between normal and CRC tissues**

A total of 297 GRGs were identified from the Molecular Signatures Database (MSigDB). Transcriptome data and clinical information were obtained from The Cancer Genome Atlas (TCGA-COAD) (https://portal.gdc.cancer.) and Gene Expression Omnibus (GEO, GSE39582, GSE14333) databases (https://www. ncbi.nlm.nih.gov/gds/). After the batch effect was tested, the dplyr and sva R packages were used to normalize and analyse the gene expression data. Differential gene expression was identified through the limma R package. P values were adjusted using the false discovery rate (FDR) method. Genes with adjusted *P* < 0.05 and |log2-fold change (FC)|>1.0 were considered DEGs in the combined analysis. Finally, the maftools R package was used to analyse the MAF and TMB information.

**Cell culture and cell lines**

SW480, CaCo2, LoVo and T84 cell lines were purchased from the Chinese Academy of Science. SW480 and T84 cells were cultured in RPMI 1640 medium (Eallbio). LoVo cells were cultured in Ham's F12 and Kaighn's Modification media (Cytiva). CaCo2 cells were cultured in MEM supplemented with 10% fetal bovine serum (OPCELL), 100 U/mL penicillin, and 100 μg/mL streptomycin (Solarbio) and incubated at 37 °C and 5% CO_2_.

**Immune Infiltration Analysis**

The associations between the risk score and tumor-infiltrating immune cell subtypes were analysed using TIMER. In addition, CIBERSORT analysis was conducted to estimate the fractions of immune infiltrates. Pathology HE-stained sections obtained from the TCGA database were used to validate the immune infiltrates in the Risk^H^ and Risk^L^ tumors.

**Immune checkpoint expression and TIDE score**

The differences in the expression of checkpoint molecules between the Risk^H^ and Risk^L^ samples were analysed via the Wilcoxon signed-rank test. The TIDE score was utilized to assess tumor immune escape and dysfunction. The ggplot2 R package was used to visualize all TIMER and CIBERSORT results.

**Transwell migration assay**

A total of 5000 cells were seeded in the upper chambers of a 24-well plate (Corning, USA) in 200 μl of medium lacking serum. The lower chamber was filled with 500 µl of medium supplemented with 10% FBS. After 24 h of incubation at 37 °C, the cells invading the lower chambers were fixed with methanol and stained with crystal violet. Images were taken with an inverted microscope (Leica, Germany) and analysed using Image J software.

**Western blotting**

Total proteins were extracted from cells with RIPA lysis buffer (Biyuntian) according to the manufacturer’s instructions. Equal amounts of protein from each sample were separated by 12.5% SDS‒PAGE and transferred onto PVDF membranes. After blocking with 5% skim milk, the blots were incubated with primary antibodies against SFRP2 (Abcam, ab137560), E-cadherin (Abcam, ab76319), vimentin (Abcam, ab92547), Twist (Abcam, ab175430), β-catenin (Abcam, ab32572), GSK-3β (Abcam, ab93926), Cyclin D1 (Abcam, ab134175), C-Myc (Abcam, ab185655), and ENO2 (Cell Signaling Technology, #8171) overnight at 4 °C and then incubated with a horseradish peroxidase-conjugated IgG secondary antibody (Earthox) for 2 h at room temperature. The protein bands were visualized with an enhanced chemiluminescence detection reagent and analysed with a Bio-Rad ChemiDoc XRS system (Bio-Rad, Hercules, CA, USA).

**Real-time quantitative polymerase chain reaction (qRT‒PCR)**

We extracted total RNA from cells using TRNzol Universal (Ambion, Beijing, China) to synthesize complementary DNA (cDNA) using the FastQuant First-Strand cDNA Synthesis Kit (Takara). The reaction mixture consisted of 1 μg of cDNA, 5 μl of SYBR Green/Fluorescein qPCR Master Mix and a primer mixture containing both upstream and downstream primers at a concentration of 2 μM. Diethylpyrocarbonate (DEPC) water was added to attain the desired volume, resulting in a final volume of 10 μl. We performed qRT‒PCR using a 7500 Fast Real-Time PCR data analysis system, with ACTIN as the internal reference. We calculated the expression of transcripts using the 2^−△△ct^ method. The primer sequences are listed in Table S11.

**Immunohistochemistry**

All antibodies used are described in the Supplementary Information. Immunohistochemical staining was conducted on tissue microarrays containing samples from colorectal cancer patients with and without metastasis, as well as from patients with high and low SFRP2 expression, and survival data were collected. Paraffin-embedded sections were rehydrated through a xylene and alcohol series, rinsed in H_2_O and washed in PBS. Antigen retrieval was performed using target EDTA (Vector Labs, Burlingame, CA) and steamed for 20 min. Samples were then blocked in peroxidase blocking buffer (Thermo Scientific) for 10 min, blocked with protein (Thermo Scientific) for 20 min, and incubated with the appropriate primary antibodies against SFRP2 (Abcam, ab137560) and ENO2 (Proteintech, 55235-1-AP) diluted in antibody diluent (S0809, Dako) at 4 °C overnight in a humidified chamber. Subsequently, the sections were washed with PBS three times, and the secondary antibody was applied for 15 min. Then, the sections were developed with horseradish peroxidase (HRP) and diaminobenzidine (DAB), counterstained with hematoxylin, differentiated with hydrochloric acid ethanol, rinsed with running water, hydrated with gradient alcohol and xylene, and mounted with neutral balsam. After that, a microscopic examination was performed.

**Metabolism assays**

The Seahorse XFe96 Analyzer was used to measure mitochondrial respiration and glycolysis. Briefly, CRC cells were plated at a density of 2×10^4^ cells per well in Agilent Seahorse XF96 Cell Culture Microplate. The glycolysis stress test which were initially cultured in glucose-free medium and ECAR was measured following 10 mM glucose (Glu), 1.5μM oligomycin (Oligo), and 50 mM 2-Deoxy-D-glucose (2-DG) injections in the indicated cells. Box plots depict Glycolysis rates (ECAR after glucose addition subtracted from baseline ECAR before the first injection), Glycolytic Capacity (ECAR after oligomycin infection subtracted from basal ECAR) and Glycolytic Reserve (glycolytic capacity minus basal glycolysis) calculated from the ECAR curve.

The OCRs were first measured on 2 × 10^4^ cells of each cell line under basal conditions and then sequentially added to oligomycin (1.0 µM), FCCP (0.5 µM), rotenone (1.0 µM), and antimycin A (1.0 µM) at the indicated times to determine the different parameters of mitochondrial functions. Graphs show the basal OCR, ATP-linked OCR, proton leak OCR, maximal OCR, reserve capacity, and non-mitochondrial OCR among the indicated cell lines. The non-mitochondrial OCR was determined as the OCR after rotenone/antimycin A treatment. The basal OCR was determined as the OCR before oligomycin minus the OCR after rotenone/antimycin A treatment. The ATP-linked OCR was determined as the OCR before oligomycin minus the OCR after oligomycin. The proton leak OCR was determined as the basal OCR minus the ATP-linked OCR. The maximal OCR was determined as the OCR after FCCP minus the non-mitochondrial OCR. Reserve capacity was defined as the difference between the maximal OCR after FCCP minus the basal OCR. Statistical significance was determined using paired t-tests. **P* < 0.05, ns, not significant.

**Functional enrichment**

[Gene Ontology](https://www.sciencedirect.com/topics/biochemistry-genetics-and-molecular-biology/gene-ontology)Gene Ontology (GO) enrichment analysis and Kyoto Encyclopedia of Genes and Genomes (KEGG) enrichment analysis were performed using the R package with *P*<0.05 and a normalized enrichment score>1 ([http://www.bioconductor.org/packages/release/bioc/html/clusterProfiler.html](http://www.bioconductor.org/packages/release/bioc/html/clusterProfiler.html)).

**Protein‒protein interaction (PPI) network construction**

Molecular complex detection (MCODE) identifies proteins of nodes in a network based on their connectivity patterns and uses a scoring system to assess the density and strength of connections within proteins. The biological network data were loaded into Cytoscape, and the MCODE algorithm was applied to the loaded network data. Visualization tools within Cytoscape were utilized to facilitate the interpretation of the results.

**Statistical analysis**

Statistical significance was set at probability values of *P* < 0.05. Statistical analysis was performed with the statistical software packages SPSS 25.0, GraphPad Prism 9.0 and ImageJ. K‒M survival analysis and the log rank test were used to plot the survival curves and compare the survival times; the significance level was set at 0.05. Comparisons between the means of two independent samples were performed using the *t* test; ANOVA was performed to compare the differences in means between multiple groups, and *P* < 0.05 was considered to indicate statistical significance.

**Fig.S1. Expression of the GRG signature in normal and CRC tissues.** (A) RT‒qPCR was used to determine the expression of 19 GRG signature in ten pairs of CRC and normal colorectal mucosa. (B) Paired comparisons of the expression levels of 19 GRG genes between CRC and normal controls from TCGA datasets.

**Fig.S2.** The prognostic value of 19 GRG signature genes in CRC patients based on TCGA data.

**Fig.S3.** Correlations between the risk score and clinicopathological parameters. T, T classification; N, lymphatic metastasis; M, distant metastasis. **P* < 0.05; ***P* < 0.01; ****P* < 0.001

**Fig.S4. GRG signature validation in the test cohort GSE39582.** (A) Patient outcomes based on the risk score. The risk score for each CRC patient (top). Survival rates and survival times of patients in different groups (bottom). (B) Kaplan‒Meier survival curves for the overall survival and progression-free survival of CRC patients in the Risk^H^ and Risk^L^ groups. (C)PCA of two groups using whole transcriptome data. (D) ROC curves showing the prognostic value of the GRG signature and clinicopathologic characteristics. (E-F) Forest plot showing the univariate and multivariate Cox analyses of the risk model for overall survival in CRC patients. (G) Boxplots representing the changes in risk scores with clinicopathological parameters. (H) Correlation between risk score and clinicopathological parameters. T, T classification; N, lymphatic metastasis; M, distant metastasis. **P* < 0.05; ***P* < 0.01; ****P* < 0.001

**Fig.S5.** (A) Heatmap showing the expression patterns of 19 alvcometabolism-related genes defined in the prognostic model. (B) GSVA showed the enriched pathways between the Risk^H^ and Risk^L^ groups.

**Fig.S6. SFRP2 overexpression indicates poor prognosis and immunosuppression in CRC patients.** (A-C) Correlations between clinicopathologic features and SFRP2 expression. (D) Relative abundance fractions of the immune cell population in the SFRP2^H^ and SFRP2^L^ groups determined using the CIBERSORT tool. (E) Correlation analysis of SFRP2 expression and immune cell infiltration. (F) Correlations between SFRP2 expression and immune-related genes. (G) Gene‒gene correlations between the SFRP2 related genes.

**Fig.S7.** **Analysis of glycolysis in CRC cells in response to SFRP2.** (A, C, E, G) the Glycolysis Stress test which were initially cultured in glucose-free medium and ECAR was measured following glucose (Glu), oligomycin (Oligo), and 2-Deoxy-D-glucose (2-DG) injections in the indicated cells. Box plots depict Glycolysis rates, Glycolytic Capacity and Glycolytic Reserve calculated from the ECAR curve. Statistical significance was determined using paired t-tests. **P* < 0.05, ns, not significant. (B, D, F, H) An analysis of O_2_ consumption rate in the various cell lines sequentially added to oligomycin (1.0 µM), FCCP (0.5 µM), rotenone (1.0 µM), and antimycin A (1.0 µM). Graphs show the basal OCR, ATP-linked OCR, proton leak OCR, maximal OCR, reserve capacity, and non-mitochondrial OCR among the indicated cell lines.

**Fig.S8.** (A-B) Boxplots representing the changes in risk scores with external beam radiotherapy (XRT) and Cyclophosphamide (CTX) from the cohort GSE14333. (C) Boxplots representing the changes in risk scores with tumor location from the cohort GSE14333. (D) Boxplots representing the changes in risk scores with chemotherapy type from the cohort GSE39582. (E) Boxplots representing the changes in risk scores with tumor location from the cohort GSE39582.

**Fig.S9. Comparison of the DNA alterations between the Risk^H^ and Risk^L^ groups.** (A) Comparison of the somatic mutation rate between the Risk^H^ and Risk^L^ groups using MAF and oncoplots. (B) Correlations between mutation frequency and GRG risk score. (C) Boxplots showing the difference in risk scores between patients with wild-type and mutant isoforms of the indicated gene.
